# Supplementary material for: Systems Genomics Reveals microRNA Regulation of ICS Response in Childhood Asthma
Source: Cells. 2023 May 29;12(11):1505. doi: 10.3390/cells12111505 (PMC10309175; doi:10.3390/cells12111505)
Supplement: Supplementary file 1 [file cells-12-01505-s001.zip › cells-2332255-supplementary-SI.pdf]

Systems Genomics Reveals microRNA Regulation of ICS Response in Childhood Asthma

Rinku Sharma[1], Anshul Tiwari[1,2], Alvin T. Kho[1,3], Juan C. Celedón[4], Scott T. Weiss[1], Kelan G. Tantisira[5], Michael J. McGeachie[1,*]

1. Channing Division of Network Medicine, Brigham and Women's Hospital, Harvard Medical School, Boston, MA 02115, USA

2. Department of Molecular Physiology and Biophysics, Vanderbilt University, Nashville, TN 37235, USA

3. Computational Health Informatics Program, Boston Children's Hospital, Boston, MA 02115, USA

4. Division of Pediatric Pulmonary Medicine, UPMC Children's Hospital of Pittsburgh, University of Pittsburgh, Pittsburgh, PA 15260, USA

5. Division of Pediatric Respiratory Medicine, University of California San Diego, Rady Children's Hospital, San Diego, CA 92123, USA

* Correspondence: remmg@channing.harvard.edu; Tel: +617-525-2272; Fax: +617-731-1541

Supplemental Materials

Supplemental Figures.

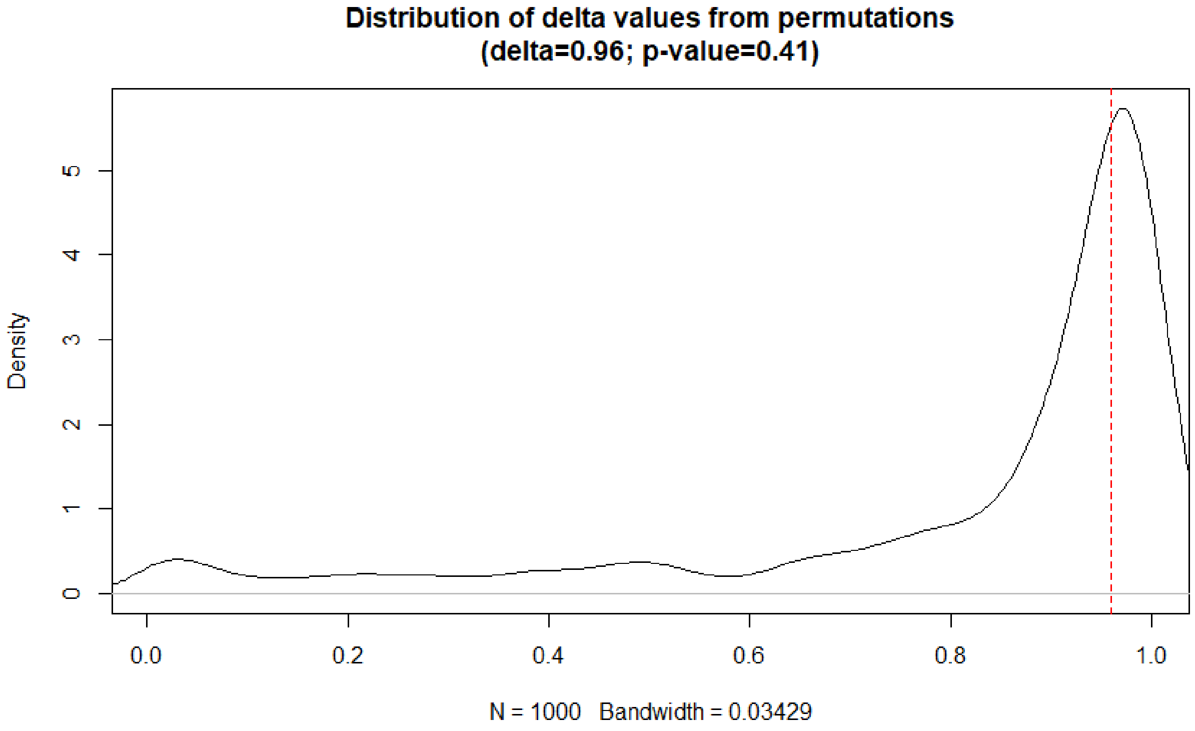

Figure S1. **GACRS batch effect check**

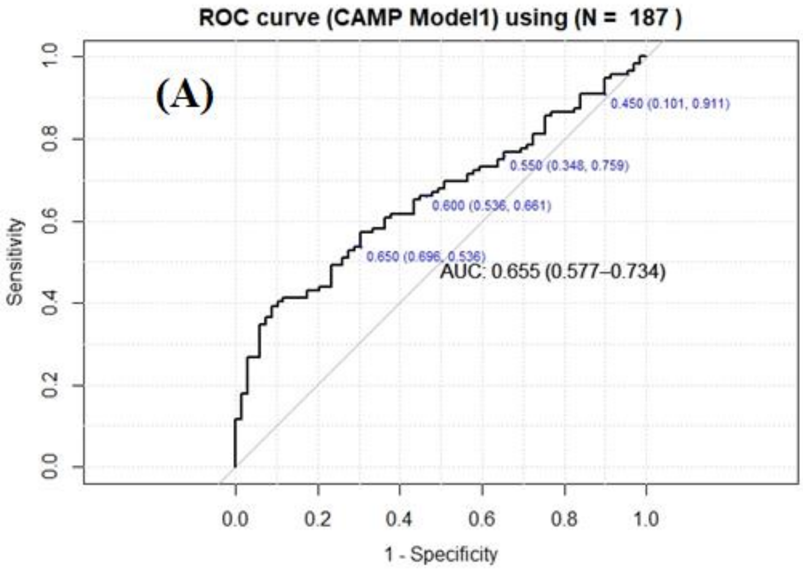

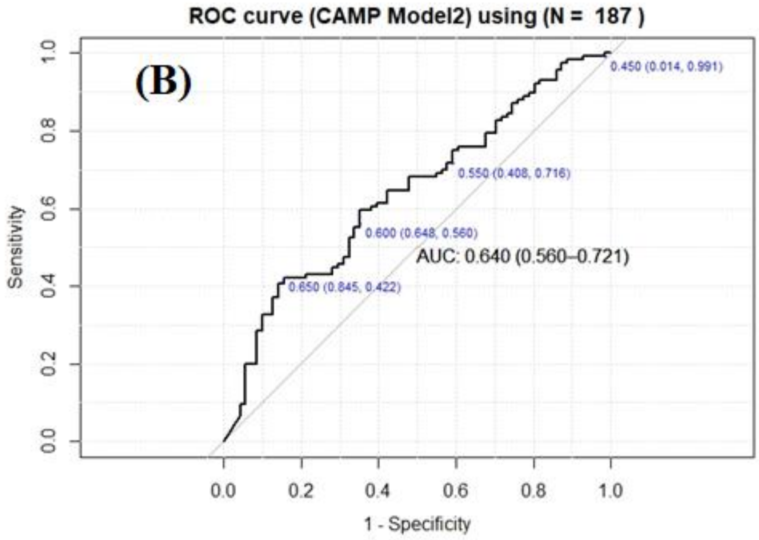

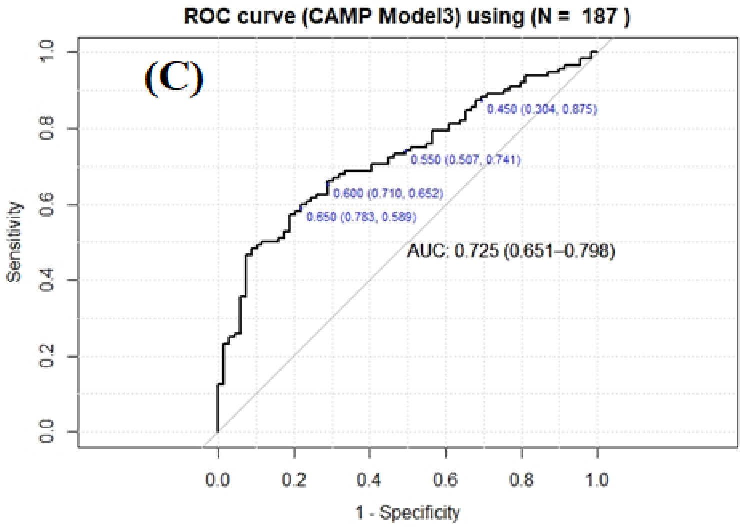

Figure S2. **ICS response prediction using logistic regression in replication cohort (CAMP)** (a) Model1 including age, sex, race/ethnicity, height, weight,BMI, log10 IgE, log10 Eosinophil, vitamin, smoking and asthma severity; (b) Model2: three miRNAs (miR-28-5p, miR-339-3p, miR-432-5p); (c) Model3: Variables of Model1 and Model2

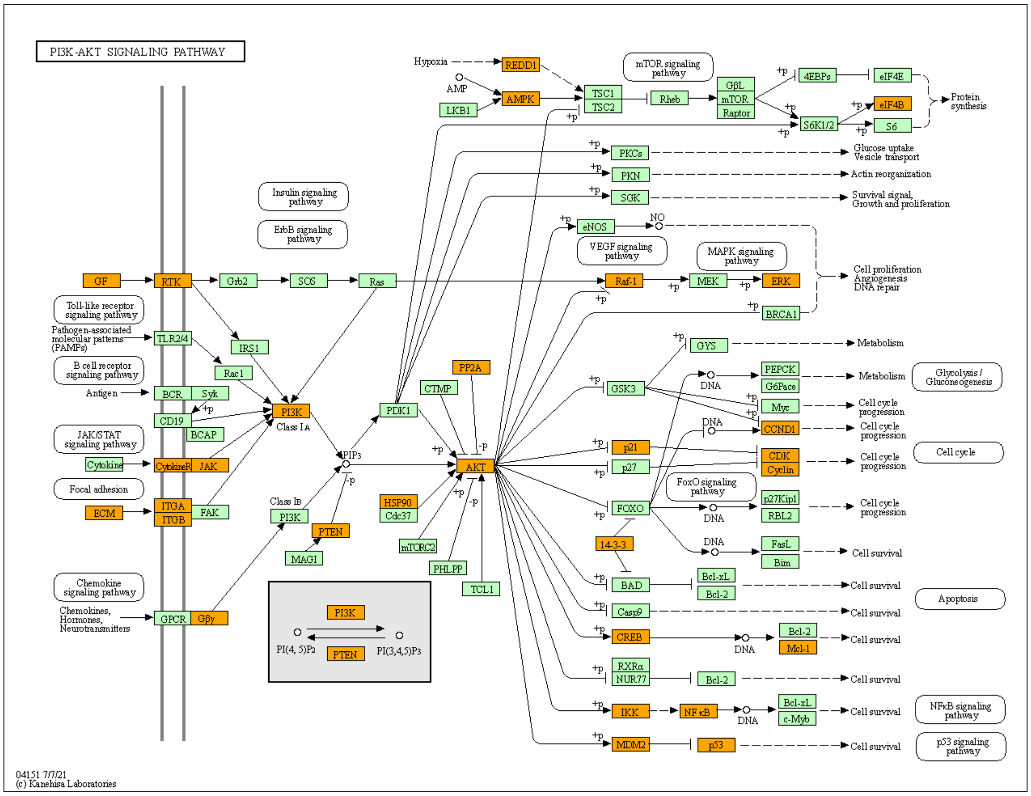

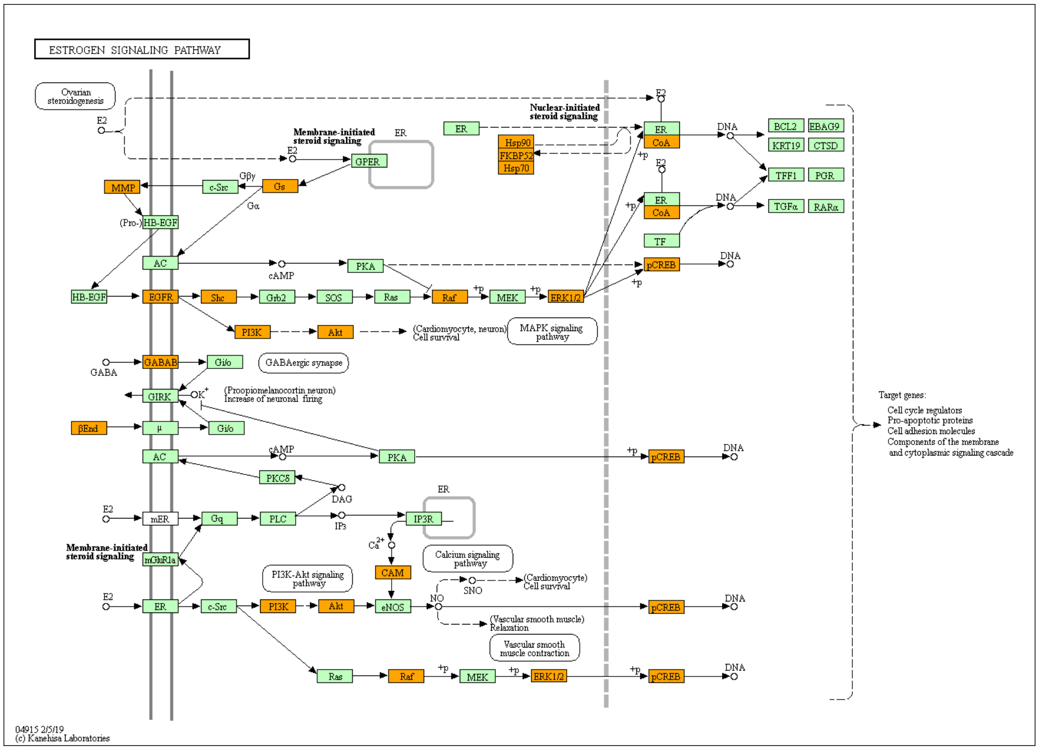

Figure S3. **Target genes of replicated miRs enriched in PI3K-AKT and estrogen signaling pathways.** Orange color box represents enriched target genes.

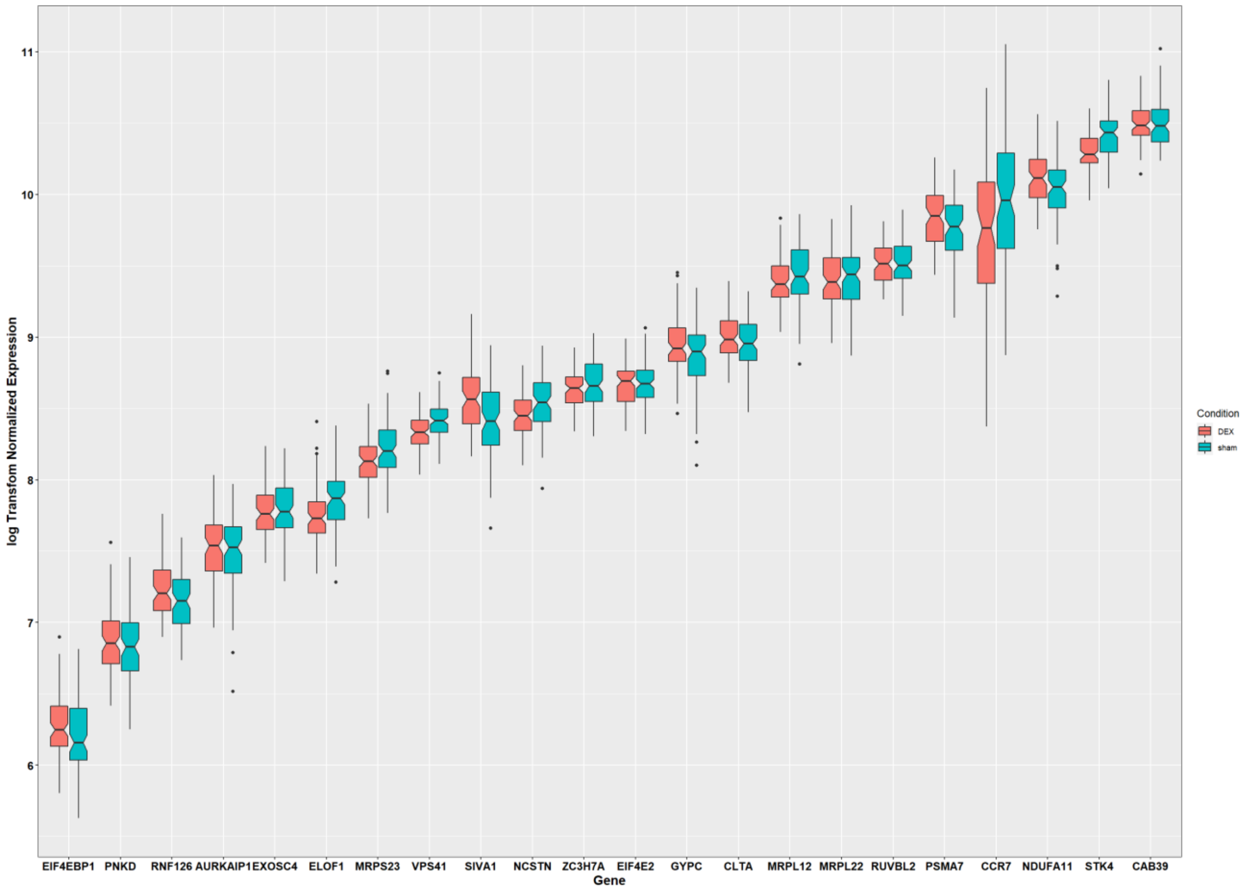

Figure S4. **Boxplot showing expression under DEX and Sham condition for 22 differentially expressed genes associated with replicated miRNAs.**

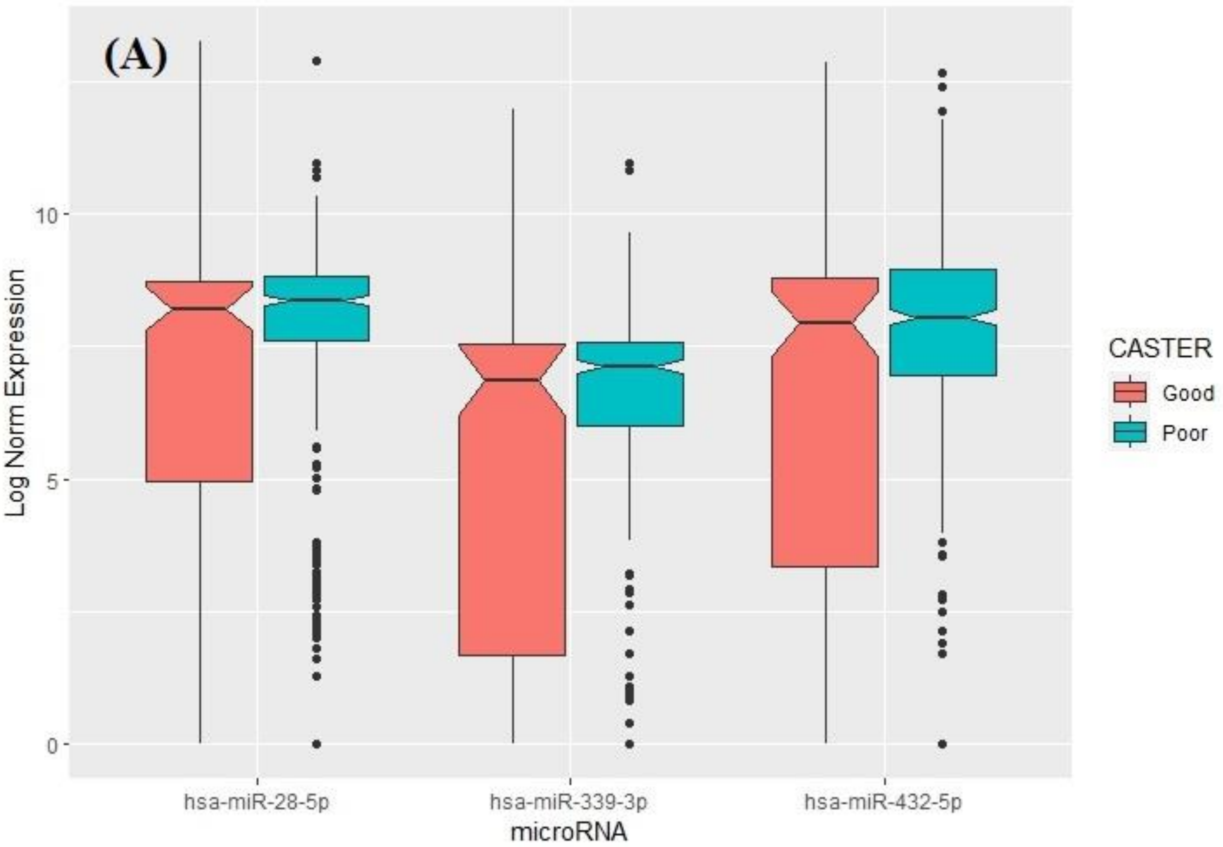

(A)
Log Norm Expression
10
5
0
hsa-miR-28-5p
hsa-miR-339-3p
hsa-miR-432-5p
microRNA
CASTER
Good
Poor

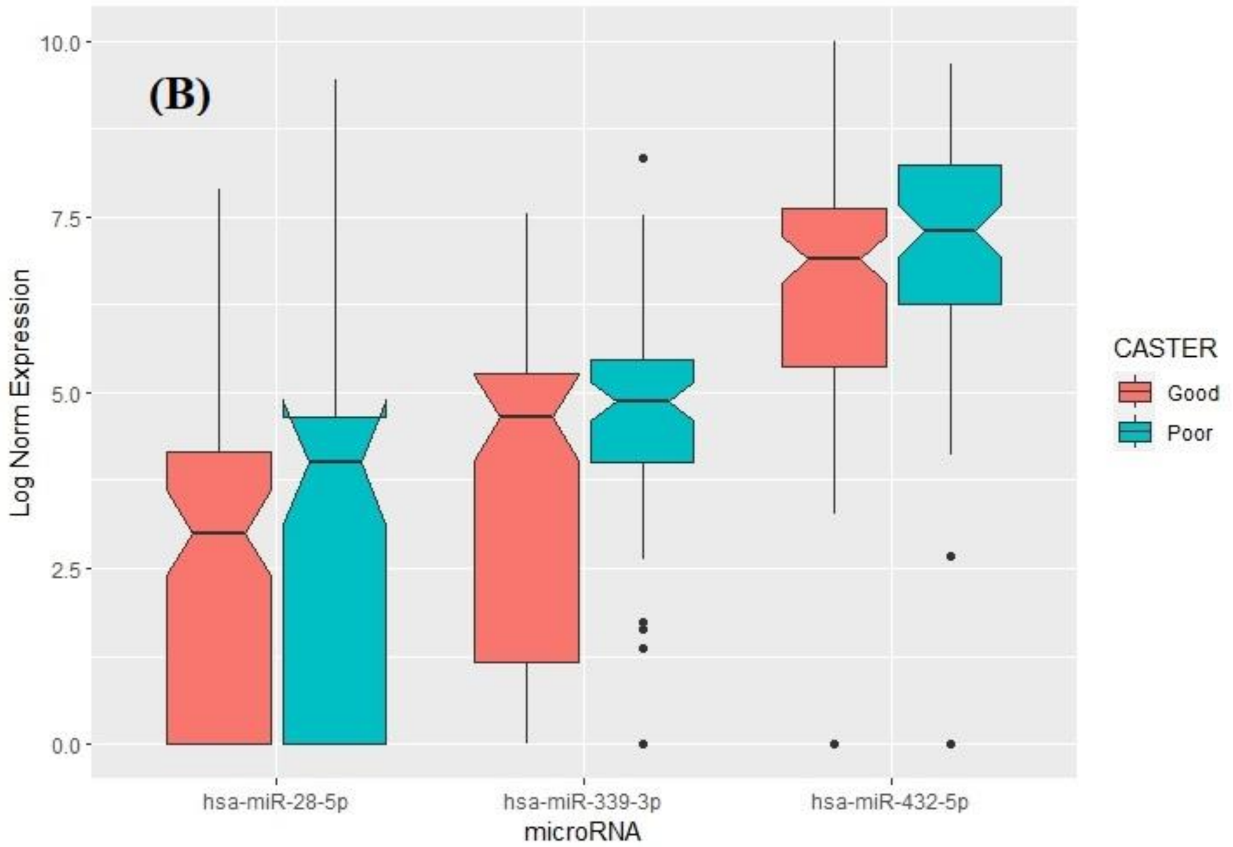

Figure S5. BoxPlot showing expression in poor and good responder group for three replicated miRNAs in (A) GACRS and (B) CAMP.

Supplemental Tables.

Table S1. miRNAs associated with ICS response in GACRS cohort.

Term	Beta	Z	P.Value	OR	ORlower	Orupper	FDR
has-miR-28-5p	0.1275	3.5813	0.0003	1.136	1.0594	1.2181	0.0011
has-miR-432-5p	0.0803	2.9883	0.0028	1.0836	1.028	1.1422	0.0082
has-miR-339-3p	0.0643	2.238	0.0252	1.0664	1.008	1.1282	0.0703
has-mir-151b	0.0908	2.1385	0.0325	1.095	1.0076	1.19	0.0887
has-miR-486-5p	-0.2412	-3.5344	0.0004	0.7857	0.6873	0.8981	0.0012
has-miR-874-3p	0.1031	3.5162	0.0004	1.1086	1.0467	1.1741	0.0013
has-miR-152-3p	0.1701	3.3312	0.0009	1.1854	1.0725	1.3102	0.0026
has-miR-23b-5p	0.0944	3.0297	0.0024	1.099	1.0339	1.1682	0.0072
has-miR-193a-5p	0.088	3.02	0.0025	1.092	1.0314	1.1562	0.0074
has-miR-340-5p	0.081	2.7563	0.0058	1.0843	1.0237	1.1486	0.0171
has-miR-340-3p	0.0775	2.6981	0.007	1.0805	1.0214	1.1431	0.0203

has-miR-221-5p	0.1197	2.6243	0.0087	1.1272	1.0308	1.2326	0.0253
has-miR-744-5p	0.0941	2.6116	0.009	1.0987	1.0238	1.179	0.0261
has-miR-15b-5p	0.0894	2.6092	0.0091	1.0935	1.0225	1.1695	0.0262
has-miR-181a-2-3p	0.0739	2.5998	0.0093	1.0767	1.0183	1.1383	0.0269
has-let-7c-5p	0.1305	2.5869	0.0097	1.1394	1.0321	1.2579	0.0278
has-miR-374b-5p	0.0705	2.4317	0.015	1.073	1.0138	1.1358	0.043
has-miR-577	0.0682	2.4306	0.0151	1.0706	1.0133	1.1311	0.0431
has-miR-195-5p	0.069	2.3885	0.0169	1.0714	1.0125	1.1338	0.0482
has-miR-451a	-0.166	-2.3615	0.0182	0.847	0.738	0.9722	0.0517
has-miR-200a-3p	0.0616	2.3107	0.0209	1.0635	1.0094	1.1205	0.059
has-miR-199a-5p	0.1178	2.3015	0.0214	1.125	1.0176	1.2436	0.0603
has-miR-199b-5p	0.0593	2.2692	0.0233	1.0611	1.0081	1.1168	0.0654
has-miR-151a-5p	0.157	2.263	0.0236	1.17	1.0213	1.3405	0.0663
has-miR-181a-3p	0.0657	2.2579	0.0239	1.0679	1.0087	1.1306	0.067
has-miR-130b-5p	0.073	2.2307	0.0257	1.0758	1.0089	1.1471	0.0715
has-miR-423-5p	-0.1456	-2.2276	0.0259	0.8645	0.7606	0.9827	0.0718
has-miR-181b-5p	0.0682	2.2152	0.0267	1.0705	1.0079	1.1371	0.0739
has-miR-142-3p	0.0616	2.1986	0.0279	1.0635	1.0067	1.1236	0.0769
has-let-7b-3p	0.0612	2.1477	0.0317	1.0632	1.0054	1.1243	0.0872
has-miR-339-5p	0.0597	2.1457	0.0319	1.0616	1.0052	1.1211	0.0874
has-miR-223-3p	0.0698	2.1149	0.0344	1.0723	1.0051	1.1441	0.0938
has-miR-11400	0.0606	2.1139	0.0345	1.0625	1.0044	1.1239	0.0938
has-miR-125b-2-3p	0.0589	2.1116	0.0347	1.0606	1.0042	1.1202	0.0941
has-miR-494-3p	0.0529	2.1012	0.0356	1.0543	1.0036	1.1076	0.0962
has-miR-181c-5p	0.0588	2.0915	0.0365	1.0606	1.0037	1.1207	0.0983

Table S2. Details of genes from LCL cell line used for differential expression analysis and WGCNA.

SYMBOL	EntrezID	Definition	logFC	AveExpr	P.Value	adj.P.Val	Module
IL2RG	3561	interleukin 2 receptor, gamma (severe combined immunodeficiency) (IL2RG), mRNA.	-0.02	9.58	3.03E-01	3.47E-01	magenta
PTEN	5728	phosphatase and tensin homolog (mutated in multiple advanced cancers 1) (PTEN), mRNA.	-0.10	7.26	3.37E-14	1.07E-13	black

CDK4	1019	cyclin-dependent kinase 4 (CDK4), mRNA.	0.15	8.54	1.30E-19	5.44E-19	magenta
JAK1	3716	Janus kinase 1 (a protein tyrosine kinase) (JAK1), mRNA.	0.02	7.05	2.80E-01	3.23E-01	blue
MCL1	4170	myeloid cell leukemia sequence 1 (BCL2-related) (MCL1), transcript variant 1, mRNA.	0.11	7.17	2.52E-11	6.82E-11	blue
POMC	5443	proopiomelanocortin (adrenocorticotropin/ beta-lipotropin/ alpha-melanocyte stimulating hormone/ beta-melanocyte stimulating hormone/ beta-endorphin) (POMC), transcript variant 2, mRNA.	0.21	10.60	1.88E-31	1.30E-30	grey
CDKN1A	1026	cyclin-dependent kinase inhibitor 1A (p21, Cip1) (CDKN1A), transcript variant 2, mRNA.	-0.02	8.55	1.73E-01	2.07E-01	red
FSCN1	6624	fascin homolog 1, actin-bundling protein (Strongylocentrotus purpuratus) (FSCN1), mRNA.	0.01	9.26	7.27E-01	7.60E-01	tan
HSP90B1	7184	heat shock protein 90kDa beta (Grp94), member 1 (HSP90B1), mRNA.	0.12	8.46	4.12E-21	1.85E-20	tan
CCND1	595	cyclin D1 (CCND1), mRNA.	-0.17	6.01	6.26E-20	2.67E-19	turquoise
FOXO1	2308	forkhead box O1A (rhabdomyosarcoma) (FOXO1A), mRNA.	0.06	7.51	3.56E-05	6.45E-05	turquoise

HSPA8	3312	heat shock 70kDa protein 8 (HSPA8), transcript variant 2, mRNA.	0.11	8.02	1.65E-09	4.02E-09	turquoise
ACLY	47	ATP citrate lyase (ACLY), transcript variant 1, mRNA.	0.05	11.01	4.74E-04	7.78E-04	black
ACP5	54	acid phosphatase 5, tartrate resistant (ACP5), mRNA.	0.04	8.94	5.69E-03	8.32E-03	black
ANAPC11	51529	APC11 anaphase promoting complex subunit 11 homolog (yeast) (ANAPC11), transcript variant 1, mRNA.	0.08	12.48	3.47E-08	7.78E-08	black
ANKRD39	51239	ankyrin repeat domain 39 (ANKRD39), mRNA.	-0.15	10.79	3.77E-11	1.01E-10	black
APRT	353	adenine phosphoribosyltransferase (APRT), transcript variant 1, mRNA.	0.10	6.63	3.72E-08	8.33E-08	black
ARF5	381	ADP-ribosylation factor 5 (ARF5), mRNA.	0.06	7.06	9.48E-04	1.50E-03	black
ATIC	471	5-aminoimidazole-4-carboxamide ribonucleotide formyltransferase/IMP cyclohydrolase (ATIC), mRNA.	-0.20	7.53	1.71E-30	1.13E-29	black
ATOX1	475	ATX1 antioxidant protein 1 homolog (yeast) (ATOX1), mRNA.	0.36	8.88	3.10E-34	2.40E-33	black
ATP2C1	27032	ATPase, Ca++ transporting, type 2C, member 1 (ATP2C1), transcript variant 1, mRNA.	-0.12	11.57	7.15E-16	2.49E-15	black

ATP5G1	516	ATP synthase, H+ transporting, mitochondrial F0 complex, subunit C1 (subunit 9) (ATP5G1), nuclear gene encoding mitochondrial protein, transcript variant 2, mRNA.	-0.17	7.29	6.28E-28	3.82E-27	black
ATP5I	521	ATP synthase, H+ transporting, mitochondrial F0 complex, subunit E (ATP5I), nuclear gene encoding mitochondrial protein, mRNA.	-0.13	8.94	3.11E-07	6.56E-07	black
ATP5J2	9551	ATP synthase, H+ transporting, mitochondrial F0 complex, subunit F2 (ATP5J2), nuclear gene encoding mitochondrial protein, transcript variant 3, mRNA.	0.04	7.74	5.92E-04	9.61E-04	black
ATP5L	10632	ATP synthase, H+ transporting, mitochondrial F0 complex, subunit G (ATP5L), nuclear gene encoding mitochondrial protein, mRNA.	0.00	7.25	8.79E-01	8.97E-01	black
ATP6V1B2	526	ATPase, H+ transporting, lysosomal 56/58kDa, V1 subunit B2 (ATP6V1B2), mRNA.	0.02	7.73	1.78E-01	2.13E-01	black
ATP6V1F	9296	ATPase, H+ transporting, lysosomal 14kDa, V1 subunit F (ATP6V1F), mRNA.	0.30	8.90	1.78E-26	1.02E-25	black

B4GALT6	9331	UDP-Gal:betaGlcNAc beta 1,4- galactosyltransferase, polypeptide 6 (B4GALT6), mRNA.	-0.09	6.85	3.95E-06	7.74E-06	black
BCL2	596	B-cell CLL/lymphoma 2 (BCL2), nuclear gene encoding mitochondrial protein, transcript variant alpha, mRNA.	0.01	9.32	4.76E-01	5.21E-01	black
BCS1L	617	BCS1-like (yeast) (BCS1L), nuclear gene encoding mitochondrial protein, transcript variant 2, mRNA.	-0.08	6.46	3.07E-05	5.59E-05	black
BFSP2	8419	beaded filament structural protein 2, phakinin (BFSP2), mRNA.	-0.16	6.47	6.55E-19	2.66E-18	black
BLOC1S1	2647	biogenesis of lysosome-related organelles complex-1, subunit 1 (BLOC1S1), mRNA.	-0.03	8.11	1.48E-02	2.07E-02	black
C16orf13	84326	hypothetical protein MGC13114 (MGC13114), transcript variant 7, mRNA.	-0.03	9.09	4.75E-02	6.22E-02	black
C19orf48	84798	chromosome 19 open reading frame 48 (C19orf48), mRNA.	0.04	11.03	3.45E-03	5.15E-03	black
C19orf53	28974	chromosome 19 open reading frame 53 (C19orf53), mRNA.	-0.21	7.71	1.31E-33	9.79E-33	black
C19orf70	125988	hypothetical protein P117 (P117), mRNA.	-0.03	8.04	1.12E-02	1.59E-02	black

C5orf22	55322	chromosome 5 open reading frame 22 (C5orf22), mRNA.	-0.01	7.75	3.62E-01	4.08E-01	black
C7orf55	154791	hypothetical protein HSPC268 (HSPC268), mRNA.	0.04	6.96	9.05E-02	1.14E-01	black
CAB39	51719	calcium binding protein 39 (CAB39), mRNA.	0.06	7.51	1.14E-02	1.61E-02	black
CAND1	55832	cullin-associated and neddylation-dissociated 1 (CAND1), mRNA.	-0.07	7.74	1.35E-04	2.32E-04	black
CCDC167	154467	chromosome 6 open reading frame 129 (C6orf129), mRNA.	0.69	10.38	4.41E-88	7.00E-86	black
CD2AP	23607	CD2-associated protein (CD2AP), mRNA.	-0.07	7.73	1.57E-06	3.15E-06	black
CD46	4179	CD46 molecule, complement regulatory protein (CD46), transcript variant d, mRNA.	0.00	12.33	9.89E-01	9.91E-01	black
CDKN2A	1029	cyclin-dependent kinase inhibitor 2A (melanoma, p16, inhibits CDK4) (CDKN2A), transcript variant 1, mRNA.	0.11	7.92	4.24E-09	1.01E-08	black
CGGBP1	8545	CGG triplet repeat binding protein 1 (CGGBP1), transcript variant 1, mRNA.	-0.05	5.77	1.03E-03	1.63E-03	black
CHCHD1	118487	coiled-coil-helix-coiled-coil-helix domain containing 1 (CHCHD1), mRNA.	0.20	8.36	3.97E-32	2.83E-31	black
CHCHD5	84269	coiled-coil-helix-coiled-coil-helix domain containing 5 (CHCHD5), mRNA.	0.01	9.08	3.76E-01	4.21E-01	black

CHMP4A	29082	chromatin modifying protein 4A (CHMP4A), mRNA.	-0.13	7.16	1.37E-13	4.20E-13	black
CIB1	10519	calcium and integrin binding 1 (calmyrin) (CIB1), mRNA.	0.41	7.58	9.01E-59	2.32E-57	black
CLPP	8192	ClpP caseinolytic peptidase, ATP-dependent, proteolytic subunit homolog (E. coli) (CLPP), nuclear gene encoding mitochondrial protein, mRNA.	0.10	5.45	2.18E-07	4.66E-07	black
CLTB	1212	clathrin, light chain (Lcb) (CLTB), transcript variant 1, mRNA.	-0.35	8.54	1.12E-29	7.21E-29	black
CNTNAP1	8506	contactin associated protein 1 (CNTNAP1), mRNA.	-0.13	8.09	1.32E-17	5.03E-17	black
COA4	51287	coiled-coil-helix-coiled-coil-helix domain containing 8 (CHCHD8), mRNA.	0.08	6.65	4.09E-06	8.00E-06	black
COMMD1	150684	copper metabolism (Murr1) domain containing 1 (COMMD1), mRNA.	-0.05	6.41	5.36E-03	7.86E-03	black
COMTD1	118881	catechol-O-methyltransferase domain containing 1 (COMTD1), mRNA.	-0.06	5.87	1.54E-05	2.89E-05	black
COX14	84987	chromosome 12 open reading frame 62 (C12orf62), mRNA.	-0.01	8.84	6.00E-01	6.41E-01	black

COX17	10063	COX17 cytochrome c oxidase assembly homolog (S. cerevisiae) (COX17), nuclear gene encoding mitochondrial protein, mRNA.	-0.03	7.93	2.63E-02	3.55E-02	black
COX5B	1329	cytochrome c oxidase subunit Vb (COX5B), mRNA.	-0.06	6.63	1.87E-05	3.48E-05	black
COX6A1	1337	cytochrome c oxidase subunit VIa polypeptide 1 (COX6A1), nuclear gene encoding mitochondrial protein, mRNA.	0.02	8.01	1.29E-01	1.58E-01	black
COX8A	1351	cytochrome c oxidase subunit 8A (ubiquitous) (COX8A), mRNA.	0.00	12.33	7.64E-01	7.94E-01	black
CRYZ	1429	crystallin, zeta (quinone reductase) (CRYZ), mRNA.	-0.25	5.97	5.13E-32	3.64E-31	black
CYBA	1535	cytochrome b-245, alpha polypeptide (CYBA), mRNA.	-0.06	8.02	1.34E-04	2.31E-04	black
CYC1	1537	cytochrome c-1 (CYC1), mRNA.	0.00	7.34	9.62E-01	9.68E-01	black
DCTN3	11258	dynactin 3 (p22) (DCTN3), transcript variant 1, mRNA.	-0.05	5.97	6.71E-03	9.75E-03	black
DCXR	51181	dicarbonyl/L-xylulose reductase (DCXR), mRNA.	0.17	7.39	6.43E-24	3.30E-23	black
DDRGK1	65992	chromosome 20 open reading frame 116 (C20orf116), mRNA.	-0.02	7.33	3.78E-01	4.23E-01	black
DNPH1	10591	chromosome 6 open reading frame 108 (C6orf108), transcript variant 1, mRNA.	-0.04	10.78	2.07E-02	2.83E-02	black

DPM3	54344	dolichyl-phosphate mannosyltransferase polypeptide 3 (DPM3), transcript variant 1, mRNA.	0.23	8.17	3.32E-31	2.25E-30	black
DYNLL2	140735	dynein, light chain, LC8-type 2 (DYNLL2), mRNA.	-0.03	10.18	3.40E-02	4.52E-02	black
DYRK4	8798	dual-specificity tyrosine-(Y)-phosphorylation regulated kinase 4 (DYRK4), mRNA.	-0.03	9.90	7.18E-02	9.18E-02	black
EBP	10682	emopamil binding protein (sterol isomerase) (EBP), mRNA.	-0.45	8.90	4.21E-56	8.93E-55	black
EBPL	84650	emopamil binding protein-like (EBPL), mRNA.	-0.01	11.85	6.26E-01	6.66E-01	black
EDF1	8721	endothelial differentiation-related factor 1 (EDF1), transcript variant alpha, mRNA.	0.14	9.50	6.79E-16	2.36E-15	black
EIF4E2	9470	eukaryotic translation initiation factor 4E family member 2 (EIF4E2), mRNA.	-0.06	8.91	2.88E-05	5.27E-05	black
EIF5A	1984	eukaryotic translation initiation factor 5A (EIF5A), mRNA.	0.02	7.73	3.04E-01	3.48E-01	black
ELF1	1997	E74-like factor 1 (ets domain transcription factor) (ELF1), mRNA.	0.32	6.78	4.86E-43	5.66E-42	black
EPB41L4A-AS1	114915	TIGA1 (TIGA1), mRNA.	-0.43	8.31	5.98E-70	2.71E-68	black
EPN1	29924	epsin 1 (EPN1), mRNA.	0.02	9.00	3.53E-02	4.69E-02	black
EXOSC5	56915	exosome component 5 (EXOSC5), mRNA.	-0.33	7.88	2.81E-52	4.81E-51	black

FAM173A	65990	chromosome 16 open reading frame 24 (C16orf24), mRNA.	-0.04	7.63	4.16E-02	5.48E-02	black
FAM96B	51647	family with sequence similarity 96, member B (FAM96B), mRNA.	-0.02	6.42	9.13E-02	1.15E-01	black
FAU	2197	Finkel-Biskis-Reilly murine sarcoma virus (FBR-MuSV) ubiquitously expressed (fox derived); ribosomal protein S30 (FAU), mRNA.	-0.24	7.60	4.73E-42	5.27E-41	black
FDPS	2224	farnesyl diphosphate synthase (farnesyl pyrophosphate synthetase, dimethylallyltranstransferase, geranyltranstransferase) (FDPS), mRNA.	-0.01	12.32	5.82E-01	6.25E-01	black
FDX1L	112812	similar to RIKEN cDNA B230118G17 gene (MGC19604), transcript variant 1, mRNA.	0.07	5.90	1.35E-04	2.31E-04	black
FIS1	51024	fission 1 (mitochondrial outer membrane) homolog (S. cerevisiae) (FIS1), mRNA.	0.41	7.74	2.84E-55	5.78E-54	black
FRG1B	284802	similar to FRG1 protein (FSHD region gene 1 protein) (MGC72104), mRNA.	-0.14	7.67	5.62E-20	2.40E-19	black
FUOM	282969	chromosome 10 open reading frame 125 (C10orf125), mRNA.	-0.09	11.90	8.65E-13	2.54E-12	black

G3BP2	9908	GTPase activating protein (SH3 domain) binding protein 2 (G3BP2), transcript variant 1, mRNA.	-0.09	7.30	1.53E-08	3.53E-08	black
GABPB1	2553	GA binding protein transcription factor, beta subunit 2 (GABPB2), transcript variant gamma-2, mRNA.	0.05	6.43	5.27E-03	7.73E-03	black
GCA	25801	grancalcin, EF-hand calcium binding protein (GCA), mRNA.	-0.02	7.32	4.58E-01	5.04E-01	black
GCLM	2730	glutamate-cysteine ligase, modifier subunit (GCLM), mRNA.	0.02	10.16	1.91E-01	2.28E-01	black
GMFG	9535	glia maturation factor, gamma (GMFG), mRNA.	-0.10	6.79	6.59E-11	1.75E-10	black
GMPS	8833	guanine monphosphate synthetase (GMPS), mRNA.	-0.03	5.85	2.87E-02	3.84E-02	black
GNGT2	2793	guanine nucleotide binding protein (G protein), gamma transducing activity polypeptide 2 (GNGT2), mRNA.	0.41	5.99	8.27E-34	6.25E-33	black
GNPTG	84572	N-acetylglucosamine-1-phosphate transferase, gamma subunit (GNPTG), mRNA.	0.03	8.65	9.05E-03	1.29E-02	black
GOLGB1	2804	golgi autoantigen, golgin subfamily b, macrogolgin (with transmembrane signal), 1 (GOLGB1), mRNA.	-0.06	7.47	3.61E-04	5.98E-04	black

GPX1	2876	glutathione peroxidase 1 (GPX1), transcript variant 1, mRNA.	-0.04	10.01	5.38E-04	8.77E-04	black
GSTK1	373156	glutathione S-transferase kappa 1 (GSTK1), mRNA.	0.08	7.18	1.71E-07	3.68E-07	black
GSTO1	9446	glutathione S-transferase omega 1 (GSTO1), mRNA.	0.00	9.01	7.09E-01	7.44E-01	black
GSTP1	2950	glutathione S-transferase pi (GSTP1), mRNA.	-0.16	8.73	4.87E-25	2.64E-24	black
GUK1	2987	guanylate kinase 1 (GUK1), mRNA.	-0.05	8.32	2.64E-03	3.98E-03	black
GYPC	2995	glycophorin C (Gerbich blood group) (GYPC), transcript variant 1, mRNA.	-0.20	7.59	3.07E-23	1.53E-22	black
HAUS4	54930	chromosome 14 open reading frame 94 (C14orf94), mRNA.	0.03	7.27	2.66E-02	3.59E-02	black
HAX1	10456	HCLS1 associated protein X-1 (HAX1), transcript variant 2, mRNA.	-0.04	8.76	6.38E-04	1.03E-03	black
HDDC3	374659	HD domain containing 3 (HDDC3), mRNA.	0.03	10.77	1.07E-01	1.33E-01	black
HIGD2A	192286	HIG1 domain family, member 2A (HIGD2A), mRNA.	0.03	8.39	4.51E-02	5.91E-02	black
HINT2	84681	histidine triad nucleotide binding protein 2 (HINT2), mRNA.	-0.13	8.21	1.84E-15	6.24E-15	black
HNRNPAB	3182	heterogeneous nuclear ribonucleoprotein A/B (HNRPAB), transcript variant 1, mRNA.	1.17	6.66	2.40E-112	2.03E-109	black

HNRNPD	3184	heterogeneous nuclear ribonucleoprotein D (AU-rich element RNA binding protein 1, 37kDa) (HNRPD), transcript variant 4, mRNA.	0.06	8.02	7.94E-04	1.27E-03	black
HRAS	3265	v-Ha-ras Harvey rat sarcoma viral oncogene homolog (HRAS), transcript variant 1, mRNA.	-0.03	9.45	4.06E-02	5.35E-02	black
HSD17B8	7923	hydroxysteroid (17-beta) dehydrogenase 8 (HSD17B8), mRNA.	-0.06	10.34	8.88E-10	2.20E-09	black
HSPA1A	3303	heat shock 70kDa protein 1A (HSPA1A), mRNA.	-0.02	6.44	2.05E-01	2.43E-01	black
HSPA1B	3304	heat shock 70kDa protein 1B (HSPA1B), mRNA.	-0.22	11.00	5.39E-50	8.45E-49	black
IARS	3376	isoleucyl-tRNA synthetase (IARS), transcript variant short, mRNA.	0.06	8.08	6.36E-06	1.23E-05	black
IFI27L1	122509	family with sequence similarity 14, member B (FAM14B), mRNA.	0.09	7.24	2.18E-11	5.92E-11	black
IFI27L2	83982	family with sequence similarity 14, member A (FAM14A), mRNA.	-0.05	10.77	5.17E-05	9.22E-05	black
IMP4	92856	IMP4, U3 small nucleolar ribonucleoprotein, homolog (yeast) (IMP4), mRNA.	0.43	6.43	1.29E-47	1.88E-46	black
KAT8	84148	MYST histone acetyltransferase 1 (MYST1), mRNA.	-0.01	9.60	6.00E-01	6.41E-01	black

KRT10	3858	keratin 10 (epidermolytic hyperkeratosis; keratosis palmaris et plantaris) (KRT10), mRNA.	-0.08	8.88	1.58E-09	3.87E-09	black
KRTCAP2	200185	keratinocyte associated protein 2 (KRTCAP2), mRNA.	-0.31	10.88	3.13E-54	5.98E-53	black
LAGE3	8270	L antigen family, member 3 (LAGE3), mRNA.	-0.13	6.82	8.04E-12	2.24E-11	black
LAMTOR2	28956	mitogen-activated protein-binding protein-interacting protein (MAPBPIP), mRNA.	0.05	8.53	2.04E-03	3.13E-03	black
LAMTOR4	389541	similar to CG14977-PA (LOC389541), mRNA.	-0.03	5.79	9.05E-02	1.14E-01	black
LANCL1	10314	LanC lantibiotic synthetase component C-like 1 (bacterial) (LANCL1), mRNA.	0.12	8.64	4.02E-13	1.21E-12	black
LAS1L	81887	LAS1-like (S. cerevisiae) (LAS1L), mRNA.	-0.03	5.76	4.73E-02	6.19E-02	black
LBHD1	79081	chromosome 11 open reading frame 48 (C11orf48), mRNA.	-0.07	5.78	3.98E-05	7.18E-05	black
LINC00116	205251	PREDICTED: LOC205251 (LOC205251), misc RNA.	0.13	9.48	6.81E-17	2.51E-16	black
LRPPRC	10128	leucine-rich PPR-motif containing (LRPPRC), mRNA.	-0.22	10.60	1.37E-45	1.81E-44	black
LSM7	51690	LSM7 homolog, U6 small nuclear RNA associated (S. cerevisiae) (LSM7), mRNA.	0.22	5.89	1.58E-26	9.09E-26	black

MAD2L2	10459	MAD2 mitotic arrest deficient-like 2 (yeast) (MAD2L2), mRNA.	0.51	5.93	1.35E-35	1.12E-34	black
MAPRE1	22919	microtubule-associated protein, RP/EB family, member 1 (MAPRE1), mRNA.	0.02	8.81	6.84E-02	8.76E-02	black
MGMT	4255	O-6-methylguanine-DNA methyltransferase (MGMT), mRNA.	0.02	6.64	3.35E-01	3.80E-01	black
MIDN	90007	midnolin (MIDN), mRNA.	0.07	5.60	2.35E-04	3.96E-04	black
MIF	4282	macrophage migration inhibitory factor (glycosylation-inhibiting factor) (MIF), mRNA.	0.09	8.96	1.59E-09	3.89E-09	black
MIIP	60672	invasion inhibitory protein 45 (IIP45), transcript variant 1, mRNA.	0.02	6.48	1.30E-01	1.60E-01	black
MPC1	51660	brain protein 44-like (BRP44L), mRNA.	-0.25	9.33	1.73E-38	1.65E-37	black
MRPL14	64928	mitochondrial ribosomal protein L14 (MRPL14), nuclear gene encoding mitochondrial protein, mRNA.	0.08	8.44	4.06E-07	8.49E-07	black
MRPL23	6150	mitochondrial ribosomal protein L23 (MRPL23), nuclear gene encoding mitochondrial protein, mRNA.	-0.01	6.23	6.32E-01	6.71E-01	black

MRPL27	51264	mitochondrial ribosomal protein L27 (MRPL27), nuclear gene encoding mitochondrial protein, transcript variant 1, mRNA.	0.00	7.55	8.45E-01	8.68E-01	black
MRPL34	64981	mitochondrial ribosomal protein L34 (MRPL34), nuclear gene encoding mitochondrial protein, mRNA.	-0.02	9.86	1.30E-01	1.59E-01	black
MRPL36	64979	mitochondrial ribosomal protein L36 (MRPL36), nuclear gene encoding mitochondrial protein, mRNA.	-0.01	8.88	4.47E-01	4.94E-01	black
MRPL41	64975	mitochondrial ribosomal protein L41 (MRPL41), nuclear gene encoding mitochondrial protein, mRNA.	0.13	7.47	1.55E-12	4.50E-12	black
MRPL53	116540	mitochondrial ribosomal protein L53 (MRPL53), nuclear gene encoding mitochondrial protein, mRNA.	0.00	12.38	9.14E-01	9.28E-01	black
MRPL54	116541	mitochondrial ribosomal protein L54 (MRPL54), nuclear gene encoding mitochondrial protein, mRNA.	0.46	7.15	5.28E-55	1.05E-53	black

MRPL55	128308	mitochondrial ribosomal protein L55 (MRPL55), nuclear gene encoding mitochondrial protein, transcript variant 8, mRNA.	0.00	7.90	9.16E-01	9.29E-01	black
MRPS11	64963	mitochondrial ribosomal protein S11 (MRPS11), nuclear gene encoding mitochondrial protein, transcript variant 1, mRNA.	0.08	8.22	4.42E-08	9.85E-08	black
MRPS18A	55168	mitochondrial ribosomal protein S18A (MRPS18A), nuclear gene encoding mitochondrial protein, mRNA.	0.09	8.24	1.56E-06	3.13E-06	black
MRPS23	51649	mitochondrial ribosomal protein S23 (MRPS23), nuclear gene encoding mitochondrial protein, mRNA.	-0.19	9.38	1.56E-20	6.85E-20	black
MRPS34	65993	mitochondrial ribosomal protein S34 (MRPS34), nuclear gene encoding mitochondrial protein, mRNA.	0.03	7.33	9.26E-02	1.16E-01	black
MSRB2	22921	methionine sulfoxide reductase B2 (MSRB2), mRNA.	0.05	9.13	1.54E-04	2.63E-04	black
MTMR4	9110	myotubularin related protein 4 (MTMR4), mRNA.	0.03	5.87	2.68E-02	3.61E-02	black
MYL12B	103910	myosin regulatory light chain MRLC2 (MRLC2), mRNA.	0.58	7.21	3.39E-74	1.87E-72	black

MYL6	4637	myosin, light chain 6, alkali, smooth muscle and non-muscle (MYL6), transcript variant 2, mRNA.	-0.01	7.28	7.41E-01	7.72E-01	black
MYOM2	9172	myomesin (M-protein) 2, 165kDa (MYOM2), mRNA.	0.09	8.98	2.21E-09	5.34E-09	black
NA	654483	bolA homolog 2B (E. coli) (BOLA2B), mRNA.	-0.35	6.58	3.36E-53	5.95E-52	black
NA	10094	actin related protein 2/3 complex, subunit 3, 21kDa (ARPC3), mRNA.	-0.21	8.42	7.83E-24	4.01E-23	black
NA	95	aminoacylase 1 (ACY1), mRNA.	-0.13	8.07	2.85E-22	1.35E-21	black
NA	79005	sodium channel modifier 1 (SCNM1), transcript variant 2, mRNA.	0.07	9.79	2.58E-04	4.33E-04	black
NA	151230	kelch-like 23 (Drosophila) (KLHL23), mRNA.	-0.04	7.51	7.92E-03	1.14E-02	black
NA	51025	mitochondria-associated protein involved in granulocyte-macrophage colony-stimulating factor signal transduction (Magmas), nuclear gene encoding mitochondrial protein, mRNA.	0.04	9.49	8.89E-03	1.27E-02	black
NA	10169	small EDRK-rich factor 2 (SERF2), mRNA.	0.02	7.74	2.07E-01	2.45E-01	black
NA	64951	mitochondrial ribosomal protein S24 (MRPS24), nuclear gene encoding mitochondrial protein, mRNA.	-0.01	7.90	5.21E-01	5.65E-01	black

NA	552900	bolA homolog 2 (E. coli) (BOLA2), transcript variant 2, mRNA.	0.00	6.88	8.05E-01	8.30E-01	black
NAA10	8260	ARD1 homolog A, N-acetyltransferase (S. cerevisiae) (ARD1A), mRNA.	0.11	9.77	3.04E-08	6.85E-08	black
NAA38	84316	LSM domain containing 1 (LSMD1), mRNA.	0.05	6.81	6.39E-06	1.23E-05	black
NARF	26502	nuclear prelamin A recognition factor (NARF), transcript variant 1, mRNA.	0.08	7.87	3.70E-07	7.75E-07	black
NCBP2	22916	nuclear cap binding protein subunit 2, 20kDa (NCBP2), transcript variant 2, mRNA.	-0.08	7.43	1.60E-08	3.68E-08	black
NDUFA11	126328	NADH dehydrogenase (ubiquinone) 1 alpha subcomplex, 11, 14.7kDa (NDUFA11), mRNA.	-0.15	8.89	2.22E-23	1.11E-22	black
NDUFA13	51079	NADH dehydrogenase (ubiquinone) 1 alpha subcomplex, 13 (NDUFA13), mRNA.	-0.03	5.95	1.38E-02	1.93E-02	black
NDUFA2	4695	NADH dehydrogenase (ubiquinone) 1 alpha subcomplex, 2, 8kDa (NDUFA2), mRNA.	-0.05	7.33	1.34E-02	1.88E-02	black
NDUFA3	4696	NADH dehydrogenase (ubiquinone) 1 alpha subcomplex, 3, 9kDa (NDUFA3), mRNA.	-0.02	9.83	1.37E-01	1.67E-01	black

NDUFA7	4701	NADH dehydrogenase (ubiquinone) 1 alpha subcomplex, 7, 14.5kDa (NDUFA7), mRNA.	0.23	7.71	3.63E-29	2.28E-28	black
NDUFB11	54539	NADH dehydrogenase (ubiquinone) 1 beta subcomplex, 11, 17.3kDa (NDUFB11), mRNA.	0.06	7.61	5.50E-04	8.96E-04	black
NDUFB2	4708	NADH dehydrogenase (ubiquinone) 1 beta subcomplex, 2, 8kDa (NDUFB2), nuclear gene encoding mitochondrial protein, mRNA.	0.11	6.53	5.83E-10	1.46E-09	black
NDUFB6	4712	NADH dehydrogenase (ubiquinone) 1 beta subcomplex, 6, 17kDa (NDUFB6), nuclear gene encoding mitochondrial protein, transcript variant 2, mRNA.	-0.05	7.66	7.65E-03	1.10E-02	black
NDUFB7	4713	NADH dehydrogenase (ubiquinone) 1 beta subcomplex, 7, 18kDa (NDUFB7), nuclear gene encoding mitochondrial protein, mRNA.	0.00	7.03	9.10E-01	9.25E-01	black
NDUFB9	4715	NADH dehydrogenase (ubiquinone) 1 beta subcomplex, 9, 22kDa (NDUFB9), mRNA.	-0.31	8.90	8.73E-40	8.65E-39	black

NETO2	81831	neuropilin (NRP) and tolloid (TLL)-like 2 (NETO2), mRNA.	0.04	8.31	4.73E-03	6.97E-03	black
NHP2	55651	nucleolar protein family A, member 2 (H/ACA small nucleolar RNPs) (NOLA2), transcript variant 1, mRNA.	-0.10	5.43	6.73E-09	1.59E-08	black
NME3	4832	non-metastatic cells 3, protein expressed in (NME3), mRNA.	-0.27	5.77	1.11E-35	9.17E-35	black
NOP10	55505	nucleolar protein family A, member 3 (H/ACA small nucleolar RNPs) (NOLA3), mRNA.	0.00	7.19	9.80E-01	9.83E-01	black
NRM	11270	nurim (nuclear envelope membrane protein) (NRM), mRNA.	-0.27	6.08	3.51E-34	2.70E-33	black
NUDT2	318	nudix (nucleoside diphosphate linked moiety X)-type motif 2 (NUDT2), transcript variant 2, mRNA.	0.03	6.26	7.55E-02	9.61E-02	black
NUDT21	11051	nudix (nucleoside diphosphate linked moiety X)-type motif 21 (NUDT21), mRNA.	-0.02	9.20	4.23E-01	4.69E-01	black
NUDT8	254552	nudix (nucleoside diphosphate linked moiety X)-type motif 8 (NUDT8), mRNA.	-0.04	7.47	1.76E-02	2.43E-02	black
NUTF2	10204	nuclear transport factor 2 (NUTF2), mRNA.	0.55	8.22	4.13E-67	1.63E-65	black
OCIAD2	132299	OCIA domain containing 2 (OCIAD2), transcript variant 1, mRNA.	-0.13	8.42	1.49E-14	4.81E-14	black

OSGEP	55644	O-sialoglycoprotein endopeptidase (OSGEP), mRNA.	-0.05	9.38	1.21E-03	1.90E-03	black
PAFAH1B3	5050	platelet-activating factor acetylhydrolase, isoform Ib, gamma subunit 29kDa (PAFAH1B3), mRNA.	0.04	6.58	6.92E-03	1.00E-02	black
PAICS	10606	phosphoribosylaminoimidazole carboxylase, phosphoribosylaminoimidazole succinocarboxamide synthetase (PAICS), transcript variant 3, mRNA.	-0.03	9.41	4.12E-02	5.43E-02	black
PFDN6	10471	prefoldin subunit 6 (PFDN6), mRNA.	-0.72	7.84	2.71E-47	3.90E-46	black
PHF20L1	51105	PHD finger protein 20-like 1 (PHF20L1), transcript variant 1, mRNA.	-0.08	8.83	4.43E-06	8.64E-06	black
PHPT1	29085	phosphohistidine phosphatase 1 (PHPT1), mRNA.	-0.19	7.85	1.38E-27	8.33E-27	black
PI4K2B	55300	phosphatidylinositol 4-kinase type 2 beta (PI4K2B), mRNA.	0.00	9.32	9.62E-01	9.68E-01	black
PJA2	9867	praja 2, RING-H2 motif containing (PJA2), mRNA.	-0.15	8.93	9.48E-20	4.00E-19	black
PNKD	25953	paroxysmal nonkinesiogenic dyskinesia (PNKD), transcript variant 1, mRNA.	-0.08	7.21	7.34E-11	1.94E-10	black
POLR2F	5435	polymerase (RNA) II (DNA directed) polypeptide F (POLR2F), mRNA.	-0.08	6.77	1.95E-04	3.30E-04	black

POLR2I	5438	polymerase (RNA) II (DNA directed) polypeptide I, 14.5kDa (POLR2I), mRNA.	-0.11	9.18	1.74E-08	3.98E-08	black
PRDX5	25824	peroxiredoxin 5 (PRDX5), nuclear gene encoding mitochondrial protein, transcript variant 1, mRNA.	0.00	8.77	9.50E-01	9.57E-01	black
PRPS1	5631	phosphoribosyl pyrophosphate synthetase 1 (PRPS1), mRNA.	0.43	5.82	3.87E-38	3.64E-37	black
PSMA7	5688	proteasome (prosome, macropain) subunit, alpha type, 7 (PSMA7), mRNA.	0.05	8.38	1.49E-03	2.31E-03	black
PSMB8	5696	proteasome (prosome, macropain) subunit, beta type, 8 (large multifunctional peptidase 7) (PSMB8), transcript variant 2, mRNA.	0.05	8.56	3.09E-04	5.15E-04	black
PSMD13	5719	proteasome (prosome, macropain) 26S subunit, non-ATPase, 13 (PSMD13), transcript variant 1, mRNA.	-0.11	6.92	7.27E-13	2.15E-12	black
PSMG3	84262	chromosome 7 open reading frame 48 (C7orf48), mRNA.	-0.01	10.55	5.32E-01	5.77E-01	black
PTBP1	5725	polypyrimidine tract binding protein 1 (PTBP1), transcript variant 2, mRNA.	-0.40	7.59	2.14E-66	8.11E-65	black

PTBP3	9991	ROD1 regulator of differentiation 1 (S. pombe) (ROD1), mRNA.	-0.03	10.63	1.67E-02	2.31E-02	black
PVRIG	79037	poliovirus receptor related immunoglobulin domain containing (PVRIG), mRNA.	0.02	8.01	9.98E-02	1.25E-01	black
PWP1	11137	PWP1 homolog (S. cerevisiae) (PWP1), mRNA.	0.06	7.82	1.03E-04	1.79E-04	black
PXMP2	5827	peroxisomal membrane protein 2, 22kDa (PXMP2), mRNA.	-0.01	6.57	4.25E-01	4.71E-01	black
PYCARD	29108	PYD and CARD domain containing (PYCARD), transcript variant 1, mRNA.	0.12	8.31	4.93E-15	1.63E-14	black
RBMX	27316	RNA binding motif protein, X-linked (RBMX), mRNA.	0.08	9.17	6.20E-08	1.37E-07	black
RHOA	387	ras homolog gene family, member A (RHOA), mRNA.	-0.02	5.84	1.02E-01	1.28E-01	black
RIOK3	8780	RIO kinase 3 (yeast) (RIOK3), transcript variant 2, mRNA.	-0.08	8.24	1.31E-07	2.83E-07	black
RNASE6	6039	ribonuclease, RNase A family, k6 (RNASE6), mRNA.	0.03	7.40	9.49E-02	1.19E-01	black
RNASEH2A	10535	ribonuclease H2, subunit A (RNASEH2A), mRNA.	0.00	7.04	8.19E-01	8.44E-01	black
RNF181	51255	hypothetical protein LOC51255 (LOC51255), mRNA.	0.90	6.66	1.10E-64	3.93E-63	black
RNF20	56254	ring finger protein 20 (RNF20), mRNA.	0.10	7.09	3.49E-11	9.36E-11	black

ROMO1	140823	chromosome 20 open reading frame 52 (C20orf52), nuclear gene encoding mitochondrial protein, mRNA.	0.54	6.98	2.53E-42	2.87E-41	black
RPL35	441246	PREDICTED: similar to 60S ribosomal protein L35, transcript variant 5 (LOC441246), mRNA.	0.04	7.84	4.95E-02	6.46E-02	black
RPL36	25873	ribosomal protein L36 (RPL36), transcript variant 1, mRNA.	-0.15	6.21	1.43E-21	6.56E-21	black
RPS19BP1	91582	ribosomal protein S19 binding protein 1 (RPS19BP1), mRNA.	0.14	7.36	7.97E-14	2.48E-13	black
RPS5	6193	ribosomal protein S5 (RPS5), mRNA.	0.09	9.42	6.37E-12	1.78E-11	black
RPS9	6203	ribosomal protein S9 (RPS9), mRNA.	0.01	7.26	6.71E-01	7.07E-01	black
SCAND1	51282	SCAN domain containing 1 (SCAND1), transcript variant 1, mRNA.	-0.02	6.48	3.01E-01	3.45E-01	black
SDHB	6390	succinate dehydrogenase complex, subunit B, iron sulfur (Ip) (SDHB), mRNA.	0.23	8.09	8.72E-38	8.06E-37	black
SDHC	6391	succinate dehydrogenase complex, subunit C, integral membrane protein, 15kDa (SDHC), nuclear gene encoding mitochondrial protein, mRNA.	0.05	8.31	2.30E-04	3.88E-04	black
SEC61B	10952	Sec61 beta subunit (SEC61B), mRNA.	0.04	8.06	1.37E-05	2.57E-05	black

SF3B5	83443	splicing factor 3b, subunit 5, 10kDa (SF3B5), mRNA.	-0.12	8.47	1.88E-19	7.81E-19	black
SIVA1	10572	SIVA1, apoptosis-inducing factor (SIVA1), transcript variant 1, mRNA.	-0.18	6.60	5.90E-25	3.19E-24	black
SLC27A5	10998	solute carrier family 27 (fatty acid transporter), member 5 (SLC27A5), mRNA.	-0.11	7.97	1.85E-07	3.97E-07	black
SMARCD2	6603	SWI/SNF related, matrix associated, actin dependent regulator of chromatin, subfamily d, member 2 (SMARCD2), mRNA.	0.12	9.04	9.35E-13	2.74E-12	black
SOD2	6648	superoxide dismutase 2, mitochondrial (SOD2), nuclear gene encoding mitochondrial protein, transcript variant 2, mRNA.	0.03	6.83	5.13E-02	6.68E-02	black
SPAG7	9552	sperm associated antigen 7 (SPAG7), mRNA.	-0.17	5.88	6.32E-22	2.94E-21	black
SRBD1	55133	S1 RNA binding domain 1 (SRBD1), mRNA.	0.01	7.48	4.21E-01	4.67E-01	black
SRP54	6729	signal recognition particle 54kDa (SRP54), mRNA.	0.05	9.26	1.33E-03	2.07E-03	black
SRSF1	6426	splicing factor, arginine/serine-rich 1 (splicing factor 2, alternate splicing factor) (SFRS1), transcript variant 2, mRNA.	0.01	7.71	3.25E-01	3.70E-01	black

SSR4	6748	signal sequence receptor, delta (translocon-associated protein delta) (SSR4), mRNA.	0.35	6.20	1.99E-24	1.05E-23	black
SUSD3	203328	sushi domain containing 3 (SUSD3), mRNA.	0.02	6.50	1.51E-01	1.83E-01	black
SWAP70	23075	SWAP-70 protein (SWAP70), mRNA.	0.03	6.92	1.44E-01	1.75E-01	black
SYNCRIP	10492	synaptotagmin binding, cytoplasmic RNA interacting protein (SYNCRIP), mRNA.	0.07	10.47	6.28E-07	1.30E-06	black
TCEB2	6923	transcription elongation factor B (SIII), polypeptide 2 (18kDa, elongin B) (TCEB2), transcript variant 2, mRNA.	-0.09	8.44	5.97E-06	1.15E-05	black
TCTEX1D2	255758	hypothetical protein MGC33212 (MGC33212), mRNA.	0.10	7.53	2.64E-14	8.41E-14	black
TFDP1	7027	transcription factor Dp-1 (TFDP1), mRNA.	0.91	5.82	1.08E-46	1.50E-45	black
TFPT	29844	TCF3 (E2A) fusion partner (in childhood Leukemia) (TFPT), mRNA.	-0.09	6.04	1.99E-11	5.42E-11	black
TFRC	7037	transferrin receptor (p90, CD71) (TFRC), mRNA.	-0.21	9.23	2.25E-30	1.47E-29	black
TIMM17B	10245	translocase of inner mitochondrial membrane 17 homolog B (yeast) (TIMM17B), mRNA.	0.00	11.06	8.99E-01	9.15E-01	black

TIMM8B	26521	translocase of inner mitochondrial membrane 8 homolog B (yeast) (TIMM8B), mRNA.	0.07	9.38	3.65E-05	6.61E-05	black
TMA7	51372	coiled-coil domain containing 72 (CCDC72), mRNA.	-0.11	5.50	6.31E-12	1.77E-11	black
TMEM101	84336	transmembrane protein 101 (TMEM101), mRNA.	0.29	6.95	2.12E-37	1.91E-36	black
TMEM141	85014	transmembrane protein 141 (TMEM141), mRNA.	0.34	8.56	2.93E-43	3.44E-42	black
TMEM160	54958	transmembrane protein 160 (TMEM160), mRNA.	0.13	6.26	5.47E-13	1.63E-12	black
TMEM208	29100	HSPC171 protein (HSPC171), mRNA.	0.11	6.90	1.58E-14	5.08E-14	black
TMEM256	254863	chromosome 17 open reading frame 61 (C17orf61), mRNA.	0.05	7.49	1.97E-05	3.65E-05	black
TNFSF4	7292	tumor necrosis factor (ligand) superfamily, member 4 (tax-transcriptionally activated glycoprotein 1, 34kDa) (TNFSF4), mRNA.	0.08	8.56	2.26E-04	3.82E-04	black
TPT1	7178	tumor protein, translationally-controlled 1 (TPT1), mRNA.	-0.02	8.11	2.17E-01	2.56E-01	black
TRAPPC1	58485	trafficking protein particle complex 1 (TRAPPC1), mRNA.	0.13	8.86	2.59E-09	6.25E-09	black

TRIM33	51592	tripartite motif-containing 33 (TRIM33), transcript variant a, mRNA.	0.21	6.32	2.48E-23	1.24E-22	black
TRMT112	51504	hypothetical protein HSPC152 (HSPC152), mRNA.	0.14	5.83	1.25E-18	5.02E-18	black
TRPT1	83707	tRNA phosphotransferase 1 (TRPT1), transcript variant 1, mRNA.	-0.06	6.80	1.20E-05	2.27E-05	black
UBL5	59286	ubiquitin-like 5 (UBL5), transcript variant 1, mRNA.	0.17	7.84	2.50E-18	9.80E-18	black
UFC1	51506	ubiquitin-fold modifier conjugating enzyme 1 (UFC1), mRNA.	0.00	12.56	8.03E-01	8.29E-01	black
UQCC2	84300	chromosome 6 open reading frame 125 (C6orf125), mRNA.	-0.01	9.15	2.23E-01	2.63E-01	black
UQCR10	29796	ubiquinol-cytochrome c reductase complex (7.2 kD) (UCRC), transcript variant 2, mRNA.	0.02	6.97	1.11E-01	1.38E-01	black
UQCRQ	27089	ubiquinol-cytochrome c reductase, complex III subunit VII, 9.5kDa (UQCRQ), nuclear gene encoding mitochondrial protein, mRNA.	-0.32	8.44	1.82E-39	1.78E-38	black
WAS	7454	Wiskott-Aldrich syndrome (eczema-thrombocytopenia) (WAS), mRNA.	-0.05	6.80	3.80E-03	5.65E-03	black
WDR83OS	51398	chromosome 19 open reading frame 56 (C19orf56), mRNA.	-0.05	9.08	2.57E-03	3.89E-03	black

XPOT	441228	PREDICTED: similar to Exportin-T (tRNA exportin) (Exportin(tRNA)) (LOC441228), mRNA.	0.07	7.82	1.19E-04	2.05E-04	black
XRCC1	7515	X-ray repair complementing defective repair in Chinese hamster cells 1 (XRCC1), mRNA.	-0.05	7.67	8.82E-04	1.41E-03	black
YBEY	54059	chromosome 21 open reading frame 57 (C21orf57), transcript variant 1, mRNA.	0.39	6.91	6.69E-61	1.93E-59	black
ZC3H7A	29066	zinc finger CCCH-type containing 7A (ZC3H7A), mRNA.	0.15	6.61	2.85E-17	1.07E-16	black
ZNF330	27309	zinc finger protein 330 (ZNF330), mRNA.	-0.13	8.27	3.60E-11	9.66E-11	black
ZNHIT1	10467	zinc finger, HIT type 1 (ZNHIT1), mRNA.	0.05	10.22	7.10E-05	1.25E-04	black
ZNRD1	30834	zinc ribbon domain containing 1 (ZNRD1), transcript variant a, mRNA.	-0.16	7.54	1.43E-17	5.46E-17	black
MARCH6	10299	membrane-associated ring finger (C3HC4) 6 (MARCH6), mRNA.	0.07	8.67	2.23E-03	3.39E-03	blue
ABCB6	10058	ATP-binding cassette, sub-family B (MDR/TAP), member 6 (ABCB6), nuclear gene encoding mitochondrial protein, mRNA.	-0.39	8.41	3.03E-74	1.71E-72	blue

ACO2	50	aconitase 2, mitochondrial (ACO2), nuclear gene encoding mitochondrial protein, mRNA.	0.48	8.73	3.31E-68	1.37E-66	blue
ACOT2	10965	acyl-CoA thioesterase 2 (ACOT2), mRNA.	-0.08	9.81	1.63E-09	3.99E-09	blue
ACP1	52	acid phosphatase 1, soluble (ACP1), transcript variant 2, mRNA.	0.21	7.98	1.71E-35	1.40E-34	blue
ACTA2	59	actin, alpha 2, smooth muscle, aorta (ACTA2), mRNA.	-0.24	7.91	8.67E-33	6.33E-32	blue
ACTG1	71	actin, gamma 1 (ACTG1), mRNA.	0.08	8.62	9.89E-13	2.90E-12	blue
ACTR2	10097	ARP2 actin-related protein 2 homolog (yeast) (ACTR2), transcript variant 2, mRNA.	0.13	9.79	1.82E-15	6.17E-15	blue
ADA	100	adenosine deaminase (ADA), mRNA.	0.01	6.06	5.66E-01	6.09E-01	blue
ADD3	120	adducin 3 (gamma) (ADD3), transcript variant 1, mRNA.	-0.02	7.30	1.74E-01	2.09E-01	blue
ADK	132	adenosine kinase (ADK), transcript variant ADK-long, mRNA.	-0.02	7.92	1.64E-02	2.27E-02	blue
ADSS	159	adenylosuccinate synthase (ADSS), mRNA.	0.03	8.33	8.08E-02	1.02E-01	blue
AFMID	125061	arylformamidase (AFMID), mRNA.	-0.09	8.19	5.76E-12	1.62E-11	blue
AGAP3	116988	centaurin, gamma 3 (CENTG3), transcript variant 1, mRNA.	0.02	7.99	7.44E-02	9.48E-02	blue
AGFG1	3267	HIV-1 Rev binding protein (HRB), mRNA.	0.01	8.18	2.66E-01	3.08E-01	blue

AGPAT2	10555	1-acylglycerol-3-phosphate O-acyltransferase 2 (lysophosphatidic acid acyltransferase, beta) (AGPAT2), transcript variant 1, mRNA.	0.17	7.68	4.65E-24	2.41E-23	blue
AGPAT3	56894	1-acylglycerol-3-phosphate O-acyltransferase 3 (AGPAT3), transcript variant 2, mRNA.	0.22	6.70	2.88E-26	1.64E-25	blue
AGPS	8540	alkylglycerone phosphate synthase (AGPS), mRNA.	0.04	8.85	6.24E-04	1.01E-03	blue
AHCYL1	10768	S-adenosylhomocysteine hydrolase-like 1 (AHCYL1), mRNA.	-0.04	8.43	2.48E-02	3.37E-02	blue
AIDA	64853	chromosome 1 open reading frame 80 (C1orf80), mRNA.	-0.13	7.82	3.44E-06	6.76E-06	blue
AIFM1	9131	apoptosis-inducing factor, mitochondrion-associated, 1 (AIFM1), nuclear gene encoding mitochondrial protein, transcript variant 1, mRNA.	0.09	7.86	3.84E-09	9.21E-09	blue
AIP	9049	aryl hydrocarbon receptor interacting protein (AIP), mRNA.	0.52	8.13	5.35E-79	4.52E-77	blue
AK4	387851	adenylate kinase 3-like 2 (AK3L2), mRNA.	-0.01	7.30	3.99E-01	4.45E-01	blue
AKR1B1	231	aldo-keto reductase family 1, member B1 (aldose reductase) (AKR1B1), mRNA.	0.12	5.72	6.93E-14	2.17E-13	blue

ALDH6A1	4329	aldehyde dehydrogenase 6 family, member A1 (ALDH6A1), nuclear gene encoding mitochondrial protein, mRNA.	0.02	12.05	1.47E-01	1.79E-01	blue
ALDOA	226	aldolase A, fructose-bisphosphate (ALDOA), transcript variant 2, mRNA.	0.05	9.70	1.54E-05	2.89E-05	blue
AMD1	262	adenosylmethionine decarboxylase 1 (AMD1), transcript variant 1, mRNA.	-0.23	9.83	3.70E-37	3.28E-36	blue
AMFR	267	autocrine motility factor receptor (AMFR), mRNA.	-0.08	6.12	4.68E-08	1.04E-07	blue
ANP32C	23520	acidic (leucine-rich) nuclear phosphoprotein 32 family, member C (ANP32C), mRNA.	0.69	5.98	8.84E-63	2.84E-61	blue
ANXA2	302	annexin A2 (ANXA2), transcript variant 2, mRNA.	-0.13	7.07	7.98E-11	2.10E-10	blue
AP1S1	1174	adaptor-related protein complex 1, sigma 1 subunit (AP1S1), transcript variant 1, mRNA.	0.32	6.38	6.89E-45	8.76E-44	blue
AP1S2	8905	adaptor-related protein complex 1, sigma 2 subunit (AP1S2), mRNA.	-0.03	6.74	1.31E-02	1.84E-02	blue
AP2S1	1175	adaptor-related protein complex 2, sigma 1 subunit (AP2S1), transcript variant AP17, mRNA.	-0.34	10.41	1.62E-45	2.14E-44	blue
APIP	51074	APAF1 interacting protein (APIP), mRNA.	0.02	7.93	2.81E-01	3.24E-01	blue

APOBEC3B	9582	apolipoprotein B mRNA editing enzyme, catalytic polypeptide-like 3B (APOBEC3B), mRNA.	0.21	8.09	2.95E-18	1.15E-17	blue
ARF3	377	ADP-ribosylation factor 3 (ARF3), mRNA.	-0.23	6.38	9.47E-23	4.62E-22	blue
ARL6IP6	151188	ADP-ribosylation-like factor 6 interacting protein 6 (ARL6IP6), mRNA.	-0.01	9.41	3.87E-01	4.33E-01	blue
ARMC8	25852	armadillo repeat containing 8 (ARMC8), transcript variant 3, mRNA.	-0.02	12.64	1.01E-01	1.26E-01	blue
ARMCX6	54470	armadillo repeat containing, X-linked 6 (ARMCX6), transcript variant 1, mRNA.	-0.10	8.29	1.39E-09	3.42E-09	blue
ARPC2	10109	actin related protein 2/3 complex, subunit 2, 34kDa (ARPC2), transcript variant 1, mRNA.	-0.02	8.50	2.65E-01	3.07E-01	blue
ARPC4	10093	actin related protein 2/3 complex, subunit 4, 20kDa (ARPC4), transcript variant 2, mRNA.	0.10	9.48	4.42E-12	1.25E-11	blue
ARSD	414	arylsulfatase D (ARSD), transcript variant 1, mRNA.	-0.14	8.36	1.03E-21	4.74E-21	blue
ASNSD1	54529	asparagine synthetase domain containing 1 (ASNSD1), mRNA.	0.08	6.70	4.31E-08	9.60E-08	blue
ASS1	445	argininosuccinate synthetase 1 (ASS1), transcript variant 1, mRNA.	-0.13	5.56	1.25E-11	3.45E-11	blue

ASXL2	55252	additional sex combs like 2 (Drosophila) (ASXL2), mRNA.	0.10	7.08	5.41E-15	1.79E-14	blue
ATP1B3	483	ATPase, Na+/K+ transporting, beta 3 polypeptide (ATP1B3), mRNA. XM_945518	-0.52	7.99	1.77E-72	9.06E-71	blue
ATP2A2	488	ATPase, Ca++ transporting, cardiac muscle, slow twitch 2 (ATP2A2), transcript variant 2, mRNA.	-0.51	6.46	9.82E-63	3.13E-61	blue
ATP5F1	515	ATP synthase, H+ transporting, mitochondrial F0 complex, subunit B1 (ATP5F1), nuclear gene encoding mitochondrial protein, mRNA.	0.21	7.40	4.49E-32	3.19E-31	blue
ATP6V0E1	8992	ATPase, H+ transporting, lysosomal 9kDa, V0 subunit e1 (ATP6V0E1), mRNA.	0.09	7.55	9.62E-10	2.37E-09	blue
ATRAID	51374	chromosome 2 open reading frame 28 (C2orf28), transcript variant 1, mRNA.	-0.08	9.48	2.62E-13	7.93E-13	blue
AUP1	550	ancient ubiquitous protein 1 (AUP1), mRNA.	0.87	8.05	1.05E-105	5.34E-103	blue
AURKAIP1	727877	PREDICTED: similar to Cyclin-L2 (Paneth cell-enhanced expression protein), transcript variant 2 (LOC727877), mRNA.	-0.08	8.23	2.05E-14	6.56E-14	blue

B3GNT2	10678	UDP-GlcNAc:betaGal beta-1,3-N-acetylglucosaminyltransferase 2 (B3GNT2), mRNA.	0.12	7.51	3.99E-11	1.07E-10	blue
BCAS4	55653	breast carcinoma amplified sequence 4 (BCAS4), transcript variant 3, mRNA.	0.00	9.17	7.02E-01	7.36E-01	blue
BCAT2	587	branched chain aminotransferase 2, mitochondrial (BCAT2), mRNA.	0.13	7.83	1.74E-16	6.23E-16	blue
BICD2	23299	bicaudal D homolog 2 (Drosophila) (BICD2), transcript variant 2, mRNA.	-1.49	8.88	5.09E-107	2.87E-104	blue
BPNT1	10380	3'(2'), 5'-bisphosphate nucleotidase 1 (BPNT1), mRNA.	-0.08	7.85	1.33E-10	3.44E-10	blue
BRMS1	25855	breast cancer metastasis suppressor 1 (BRMS1), transcript variant 3, mRNA.	-0.19	7.74	3.87E-31	2.61E-30	blue
BTBD1	53339	BTB (POZ) domain containing 1 (BTBD1), transcript variant 1, mRNA.	0.17	7.50	7.72E-18	2.97E-17	blue
C11orf73	51501	chromosome 11 open reading frame 73 (C11orf73), mRNA.	-0.02	9.73	1.03E-01	1.29E-01	blue
C1orf43	25912	chromosome 1 open reading frame 43 (C1orf43), transcript variant 1, mRNA.	0.32	9.20	9.36E-50	1.45E-48	blue

C21orf33	8209	chromosome 21 open reading frame 33 (C21orf33), nuclear gene encoding mitochondrial protein, transcript variant 1, mRNA.	0.18	6.58	7.33E-23	3.60E-22	blue
C9orf85	138241	chromosome 9 open reading frame 85 (C9orf85), mRNA.	0.00	9.00	6.51E-01	6.89E-01	blue
CACYBP	27101	calcyclin binding protein (CACYBP), transcript variant 1, mRNA.	-0.07	6.17	3.04E-07	6.40E-07	blue
CALU	813	calumenin (CALU), mRNA.	-0.07	6.03	4.35E-07	9.07E-07	blue
CANX	821	calnexin (CANX), transcript variant 1, mRNA.	0.04	8.41	7.11E-03	1.03E-02	blue
CAPRIN1	4076	GPI-anchored membrane protein 1 (GPIAP1), transcript variant 2, mRNA.	-0.06	6.58	1.36E-03	2.12E-03	blue
CAT	847	catalase (CAT), mRNA.	0.00	7.54	6.24E-01	6.64E-01	blue
CCM2	83605	cerebral cavernous malformation 2 (CCM2), transcript variant 2, mRNA.	0.19	5.73	5.11E-21	2.28E-20	blue
CCNK	8812	cyclin K (CCNK), mRNA.	-0.12	8.11	1.59E-19	6.65E-19	blue
CCS	9973	copper chaperone for superoxide dismutase (CCS), mRNA.	-0.04	8.71	3.22E-04	5.36E-04	blue
CCT7	10574	chaperonin containing TCP1, subunit 7 (eta) (CCT7), transcript variant 1, mRNA.	-0.32	9.44	7.30E-45	9.27E-44	blue
CCZ1	51622	chromosome 7 open reading frame 28A (C7orf28A), mRNA.	0.13	6.50	1.04E-11	2.87E-11	blue

CD151	977	CD151 molecule (Raph blood group) (CD151), transcript variant 1, mRNA.	0.30	7.05	5.16E-47	7.36E-46	blue
CD79A	973	CD79a molecule, immunoglobulin-associated alpha (CD79A), transcript variant 1, mRNA.	-0.38	9.31	1.26E-53	2.28E-52	blue
CD79B	974	CD79b molecule, immunoglobulin-associated beta (CD79B), transcript variant 1, mRNA.	0.07	9.56	2.00E-05	3.71E-05	blue
CDAN1	146059	congenital dyserythropoietic anemia, type I (CDAN1), mRNA.	0.32	8.39	3.03E-52	5.17E-51	blue
CDC26	246184	cell division cycle 26 homolog (S. cerevisiae) (CDC26), mRNA.	-0.01	5.63	4.54E-01	5.00E-01	blue
CDC42	998	cell division cycle 42 (GTP binding protein, 25kDa) (CDC42), transcript variant 2, mRNA.	-0.15	10.85	3.00E-29	1.89E-28	blue
CDC42SE2	56990	CDC42 small effector 2 (CDC42SE2), transcript variant 1, mRNA.	0.05	7.66	1.10E-05	2.09E-05	blue
CDC45	8318	CDC45 cell division cycle 45-like (S. cerevisiae) (CDC45L), mRNA.	-0.01	9.39	7.15E-01	7.48E-01	blue
CDK14	5218	PFTAIRE protein kinase 1 (PFTK1), mRNA.	-0.19	7.02	3.38E-34	2.61E-33	blue
CDK2	1017	cyclin-dependent kinase 2 (CDK2), transcript variant 1, mRNA.	1.05	8.60	1.27E-77	9.06E-76	blue
CDKN1B	1027	cyclin-dependent kinase inhibitor 1B (p27, Kip1) (CDKN1B), mRNA.	0.11	7.10	4.16E-08	9.28E-08	blue

CDKN2AIP	55602	CDKN2A interacting protein (CDKN2AIP), mRNA.	-0.02	8.49	3.59E-02	4.77E-02	blue
CEBPG	1054	CCAAT/enhancer binding protein (C/EBP), gamma (CEBPG), mRNA.	0.07	6.16	2.42E-09	5.84E-09	blue
CENPM	79019	centromere protein M (CENPM), transcript variant 1, mRNA.	-0.22	11.13	2.02E-36	1.74E-35	blue
CHD4	1108	chromodomain helicase DNA binding protein 4 (CHD4), mRNA.	0.11	7.90	2.36E-21	1.07E-20	blue
CHD8	57680	chromodomain helicase DNA binding protein 8 (CHD8), mRNA.	-0.01	6.04	6.31E-01	6.71E-01	blue
CHI3L2	1117	chitinase 3-like 2 (CHI3L2), transcript variant 3, mRNA.	0.23	8.17	1.34E-21	6.18E-21	blue
CHMP1B	57132	chromatin modifying protein 1B (CHMP1B), mRNA.	0.05	6.41	7.92E-03	1.14E-02	blue
CHMP2A	27243	chromatin modifying protein 2A (CHMP2A), transcript variant 1, mRNA.	-0.38	8.99	4.84E-59	1.30E-57	blue
CKAP5	9793	cytoskeleton associated protein 5 (CKAP5), transcript variant 1, mRNA.	0.11	8.22	1.73E-09	4.22E-09	blue
CLK3	1198	CDC-like kinase 3 (CLK3), transcript variant phclk3, mRNA.	-0.13	7.74	3.88E-16	1.36E-15	blue
CNIH1	10175	cornichon homolog (Drosophila) (CNIH), transcript variant 2, mRNA.	-0.04	7.59	2.72E-03	4.09E-03	blue
CNN2	1265	calponin 2 (CNN2), transcript variant 1, mRNA.	-0.10	7.32	9.73E-12	2.70E-11	blue

CNN3	1266	calponin 3, acidic (CNN3), mRNA.	-0.02	9.13	1.17E-01	1.45E-01	blue
CNOT10	25904	CCR4-NOT transcription complex, subunit 10 (CNOT10), mRNA.	0.12	11.72	3.72E-14	1.18E-13	blue
CNPY3	10695	trinucleotide repeat containing 5 (TNRC5), transcript variant 1, mRNA.	0.25	7.85	1.75E-35	1.43E-34	blue
COPZ1	22818	coatomer protein complex, subunit zeta 1 (COPZ1), mRNA.	0.06	8.70	8.11E-07	1.66E-06	blue
CRTAP	10491	PREDICTED: cartilage associated protein (CRTAP), mRNA.	0.34	6.19	2.41E-41	2.56E-40	blue
CRY2	1408	cryptochrome 2 (photolyase-like) (CRY2), mRNA.	0.02	9.44	3.54E-01	3.99E-01	blue
CTNNB1	1499	catenin (cadherin-associated protein), beta 1, 88kDa (CTNNB1), mRNA. XM_945653 XM_945654 XM_945655 XM_945657	-0.05	6.43	2.31E-03	3.51E-03	blue
CXorf56	63932	chromosome X open reading frame 56 (CXorf56), mRNA.	-0.06	9.61	1.78E-06	3.57E-06	blue
CYP4V2	285440	cytochrome P450, family 4, subfamily V, polypeptide 2 (CYP4V2), mRNA.	-0.05	8.39	4.99E-07	1.03E-06	blue
DAZAP1	26528	DAZ associated protein 1 (DAZAP1), transcript variant 2, mRNA.	-0.09	7.24	2.32E-04	3.91E-04	blue
DCK	1633	deoxycytidine kinase (DCK), mRNA.	1.42	6.83	2.21E-99	8.64E-97	blue

DCUN1D1	54165	DCN1, defective in cullin neddylation 1, domain containing 1 (S. cerevisiae) (DCUN1D1), mRNA.	0.00	7.87	8.11E-01	8.37E-01	blue
DDX17	10521	DEAD (Asp-Glu-Ala-Asp) box polypeptide 17 (DDX17), transcript variant 1, mRNA.	-0.08	9.27	8.18E-08	1.79E-07	blue
DDX47	51202	DEAD (Asp-Glu-Ala-Asp) box polypeptide 47 (DDX47), transcript variant 1, mRNA.	-0.05	8.83	3.09E-04	5.15E-04	blue
DEPDC1B	55789	DEP domain containing 1B (DEPDC1B), mRNA.	0.00	6.66	9.39E-01	9.48E-01	blue
DHRS1	115817	dehydrogenase/reductase (SDR family) member 1 (DHRS1), mRNA.	-0.01	6.98	6.68E-01	7.05E-01	blue
DHRS4	10901	dehydrogenase/reductase (SDR family) member 4 (DHRS4), mRNA.	-0.04	7.17	7.00E-03	1.02E-02	blue
DHRS4L2	317749	dehydrogenase/reductase (SDR family) member 4 like 2 (DHRS4L2), mRNA.	-0.04	8.66	5.51E-03	8.08E-03	blue
DHX33	56919	DEAH (Asp-Glu-Ala-His) box polypeptide 33 (DHX33), mRNA.	-0.09	7.78	1.53E-06	3.08E-06	blue
DHX9	1660	DEAH (Asp-Glu-Ala-His) box polypeptide 9 (DHX9), mRNA.	0.44	8.39	6.61E-62	2.01E-60	blue

DICER1	23405	Dicer1, Dcr-1 homolog (Drosophila) (DICER1), transcript variant 1, mRNA.	-0.02	9.07	3.27E-02	4.35E-02	blue
DNAJC7	7266	DnaJ (Hsp40) homolog, subfamily C, member 7 (DNAJC7), mRNA.	-0.21	10.10	6.85E-44	8.22E-43	blue
DPM1	8813	dolichyl-phosphate mannosyltransferase polypeptide 1, catalytic subunit (DPM1), mRNA.	0.38	7.44	6.84E-54	1.28E-52	blue
DPP3	10072	dipeptidyl-peptidase 3 (DPP3), transcript variant 2, mRNA.	0.07	7.66	3.26E-04	5.42E-04	blue
DPP7	29952	dipeptidyl-peptidase 7 (DPP7), mRNA.	-0.03	8.98	2.17E-03	3.31E-03	blue
DTYMK	1841	deoxythymidylate kinase (thymidylate kinase) (DTYMK), mRNA.	-0.08	8.92	1.97E-09	4.78E-09	blue
DUS3L	56931	dihydrouridine synthase 3-like (S. cerevisiae) (DUS3L), mRNA.	-0.06	10.88	5.67E-08	1.26E-07	blue
DUT	1854	dUTP pyrophosphatase (DUT), nuclear gene encoding mitochondrial protein, transcript variant 1, mRNA.	0.10	8.58	2.01E-08	4.59E-08	blue
DYNC2LI1	51626	dynein, cytoplasmic 2, light intermediate chain 1 (DYNC2LI1), transcript variant 2, mRNA.	0.00	6.13	8.65E-01	8.86E-01	blue
DYNLRB1	83658	dynein, light chain, roadblock-type 1 (DYNLRB1), mRNA.	0.62	6.03	3.36E-56	7.17E-55	blue

ECD	11319	ecdysoneless homolog (Drosophila) (ECD), mRNA.	0.62	7.26	2.76E-78	2.12E-76	blue
ECH1	1891	enoyl Coenzyme A hydratase 1, peroxisomal (ECH1), mRNA.	0.61	6.74	2.43E-78	1.93E-76	blue
EEF1A1	1915	eukaryotic translation elongation factor 1 alpha 1 (EEF1A1), mRNA.	0.00	6.41	8.16E-01	8.41E-01	blue
EEF1B2	1933	eukaryotic translation elongation factor 1 beta 2 (EEF1B2), transcript variant 1, mRNA.	0.12	6.97	7.55E-14	2.35E-13	blue
EIF3F	8665	eukaryotic translation initiation factor 3, subunit 5 epsilon, 47kDa (EIF3S5), mRNA.	0.13	6.49	1.04E-16	3.81E-16	blue
EIF3M	10480	PCI domain containing 1 (herpesvirus entry mediator) (PCID1), mRNA.	-0.01	8.55	4.97E-01	5.42E-01	blue
EIF4H	653994	PREDICTED: similar to eukaryotic translation initiation factor 4H isoform 2, transcript variant 5 (LOC653994), mRNA.	-0.05	10.20	1.43E-06	2.89E-06	blue
EIF4H	7458	eukaryotic translation initiation factor 4H (EIF4H), transcript variant 1, mRNA.	-0.03	6.59	5.43E-02	7.04E-02	blue
EIF5	1983	eukaryotic translation initiation factor 5 (EIF5), transcript variant 1, mRNA.	0.00	8.13	9.94E-01	9.95E-01	blue

ELMO1	9844	engulfment and cell motility 1 (ELMO1), transcript variant 1, mRNA.	-0.29	7.58	7.12E-44	8.52E-43	blue
EML2	24139	echinoderm microtubule associated protein like 2 (EML2), mRNA.	-0.02	7.92	2.50E-01	2.91E-01	blue
ENO2	2026	enolase 2 (gamma, neuronal) (ENO2), mRNA.	0.04	6.48	1.13E-02	1.60E-02	blue
ENSA	2029	endosulfine alpha (ENSA), transcript variant 8, mRNA.	-0.05	8.01	4.69E-03	6.92E-03	blue
ERCC1	2067	excision repair cross-complementing rodent repair deficiency, complementation group 1 (includes overlapping antisense sequence) (ERCC1), transcript variant 2, mRNA.	0.02	8.58	3.69E-01	4.15E-01	blue
ETFB	2109	electron-transfer-flavoprotein, beta polypeptide (ETFB), transcript variant 1, mRNA.	0.07	5.98	1.27E-06	2.57E-06	blue
ETV6	2120	ets variant gene 6 (TEL oncogene) (ETV6), mRNA.	0.02	7.33	2.16E-01	2.56E-01	blue
EWSR1	2130	Ewing sarcoma breakpoint region 1 (EWSR1), transcript variant EWS, mRNA.	0.11	6.91	1.78E-15	6.03E-15	blue
EZH2	2146	enhancer of zeste homolog 2 (Drosophila) (EZH2), transcript variant 1, mRNA.	0.64	6.71	1.64E-79	1.43E-77	blue
FAF1	11124	Fas (TNFRSF6) associated factor 1 (FAF1), mRNA.	0.31	10.03	4.99E-39	4.80E-38	blue

FAHD1	81889	fumarylacetoacetate hydrolase domain containing 1 (FAHD1), transcript variant 1, mRNA.	0.10	9.15	4.82E-14	1.52E-13	blue
FAM172A	83989	chromosome 5 open reading frame 21 (C5orf21), mRNA.	-0.51	7.62	3.12E-71	1.48E-69	blue
FAM174A	345757	transmembrane protein 157 (TMEM157), mRNA.	0.12	9.13	1.21E-10	3.15E-10	blue
FAM195A	84331	chromosome 16 open reading frame 14 (C16orf14), mRNA.	0.06	7.54	1.58E-06	3.18E-06	blue
FAM213A	84293	chromosome 10 open reading frame 58 (C10orf58), mRNA.	0.05	9.35	1.48E-03	2.30E-03	blue
FAM3C	10447	family with sequence similarity 3, member C (FAM3C), transcript variant 1, mRNA.	0.03	6.76	6.56E-02	8.43E-02	blue
FBXO22	26263	F-box protein 22 (FBXO22), transcript variant 2, mRNA.	-0.02	7.81	1.67E-01	2.01E-01	blue
FBXO7	25793	F-box protein 7 (FBXO7), transcript variant 1, mRNA.	0.28	6.34	5.21E-38	4.86E-37	blue
FEM1C	56929	fem-1 homolog c (C. elegans) (FEM1C), mRNA.	0.09	10.39	2.53E-13	7.66E-13	blue
FEN1	2237	flap structure-specific endonuclease 1 (FEN1), mRNA.	0.07	7.18	1.82E-03	2.81E-03	blue
FEZ1	9638	fasciculation and elongation protein zeta 1 (zygin I) (FEZ1), transcript variant 1, mRNA.	-0.07	8.70	6.80E-07	1.40E-06	blue

FIGNL1	63979	fidgetin-like 1 (FIGNL1), transcript variant 2, mRNA.	0.03	5.81	7.33E-02	9.35E-02	blue
FNTA	2339	farnesyltransferase, CAAX box, alpha (FNTA), transcript variant 1, mRNA.	0.06	7.25	2.48E-04	4.17E-04	blue
FOCAD	54914	KIAA1797 (KIAA1797), mRNA.	0.16	6.04	4.60E-22	2.16E-21	blue
FTL	2512	ferritin, light polypeptide (FTL), mRNA.	0.00	7.85	9.46E-01	9.54E-01	blue
GALK2	2585	galactokinase 2 (GALK2), transcript variant 1, mRNA.	0.34	6.25	1.37E-52	2.36E-51	blue
GALNT10	55568	UDP-N-acetyl-alpha-D-galactosamine:polypeptide N-acetylgalactosaminyltransferase 10 (GalNAc-T10) (GALNT10), transcript variant 2, mRNA.	-0.15	9.72	1.34E-23	6.79E-23	blue
GANAB	23193	glucosidase, alpha; neutral AB (GANAB), transcript variant 2, mRNA.	0.07	9.55	4.13E-05	7.43E-05	blue
GAR1	54433	nucleolar protein family A, member 1 (H/ACA small nucleolar RNPs) (NOLA1), transcript variant 1, mRNA.	-0.14	7.79	1.60E-11	4.37E-11	blue

GART	2618	phosphoribosylglycinamide formyltransferase, phosphoribosylglycinamide synthetase, phosphoribosylaminoimidazole synthetase (GART), transcript variant 2, mRNA.	0.02	5.96	2.77E-01	3.20E-01	blue
GATM	2628	glycine amidinotransferase (L-arginine:glycine amidinotransferase) (GATM), nuclear gene encoding mitochondrial protein, mRNA.	0.09	9.61	1.21E-06	2.45E-06	blue
GFM2	84340	G elongation factor, mitochondrial 2 (GFM2), nuclear gene encoding mitochondrial protein, transcript variant 3, mRNA.	-0.15	6.96	5.65E-21	2.52E-20	blue
GGCT	79017	chromosome 7 open reading frame 24 (C7orf24), mRNA.	-0.08	8.50	4.33E-13	1.30E-12	blue
GLCCI1	113263	glucocorticoid induced transcript 1 (GLCCI1), mRNA.	-0.04	8.92	1.04E-01	1.30E-01	blue
GNG10	2790	guanine nucleotide binding protein (G protein), gamma 10 (GNG10), mRNA.	-0.04	8.05	2.22E-03	3.38E-03	blue
GNG2	54331	guanine nucleotide binding protein (G protein), gamma 2 (GNG2), mRNA.	-0.01	9.68	6.94E-01	7.28E-01	blue
GOSR2	9570	golgi SNAP receptor complex member 2 (GOSR2), transcript variant B, mRNA.	0.34	7.79	6.38E-61	1.85E-59	blue

GPAA1	8733	glycosylphosphatidylinositol anchor attachment protein 1 homolog (yeast) (GPAA1), mRNA.	-0.03	11.71	2.22E-02	3.03E-02	blue
GPR15	2838	G protein-coupled receptor 15 (GPR15), mRNA.	0.05	10.85	3.48E-05	6.31E-05	blue
GPX7	2882	glutathione peroxidase 7 (GPX7), mRNA.	-0.08	5.88	2.39E-07	5.09E-07	blue
GRAP	391157	PREDICTED: similar to GRB2-related adapter protein (LOC391157), mRNA.	-0.06	7.97	2.74E-06	5.41E-06	blue
GRB2	2885	growth factor receptor-bound protein 2 (GRB2), transcript variant 1, mRNA.	-0.06	6.04	4.36E-04	7.19E-04	blue
GRN	2896	granulin (GRN), mRNA.	-0.09	7.93	8.88E-08	1.94E-07	blue
GSR	2936	glutathione reductase (GSR), mRNA.	0.02	6.91	4.86E-02	6.35E-02	blue
GTF2H2	2966	general transcription factor IIH, polypeptide 2, 44kDa (GTF2H2), mRNA.	-0.14	6.27	3.68E-18	1.44E-17	blue
GTF2H3	2967	general transcription factor IIH, polypeptide 3, 34kDa (GTF2H3), mRNA.	0.08	7.74	2.04E-07	4.36E-07	blue
GUCY1A3	2982	guanylate cyclase 1, soluble, alpha 3 (GUCY1A3), mRNA.	0.00	7.10	7.25E-01	7.57E-01	blue
H3F3C	440093	similar to H3 histone, family 3B (LOC440093), mRNA.	-0.03	10.40	2.23E-02	3.04E-02	blue

HAT1	8520	histone acetyltransferase 1 (HAT1), transcript variant 1, mRNA.	-0.11	9.80	7.04E-17	2.59E-16	blue
HAUS1	115106	coiled-coil domain containing 5 (spindle associated) (CCDC5), mRNA.	0.08	8.47	2.13E-04	3.60E-04	blue
HELLS	3070	helicase, lymphoid-specific (HELLS), mRNA.	0.02	10.47	1.29E-01	1.58E-01	blue
HIBADH	11112	3-hydroxyisobutyrate dehydrogenase (HIBADH), mRNA.	0.01	7.62	5.82E-01	6.24E-01	blue
HIF1A	3091	hypoxia-inducible factor 1, alpha subunit (basic helix-loop-helix transcription factor) (HIF1A), transcript variant 1, mRNA.	0.04	7.80	7.81E-03	1.13E-02	blue
HLA-C	3107	major histocompatibility complex, class I, C (HLA-C), mRNA.	0.08	6.50	2.18E-08	4.95E-08	blue
HLA-F	3134	major histocompatibility complex, class I, F (HLA-F), mRNA.	-0.04	8.81	7.21E-03	1.04E-02	blue
HM13	81502	histocompatibility (minor) 13 (HM13), transcript variant 1, mRNA.	0.01	6.30	6.92E-01	7.27E-01	blue
HMBS	3145	hydroxymethylbilane synthase (HMBS), transcript variant 1, mRNA.	-0.15	7.10	3.62E-21	1.63E-20	blue
HMGN2	3151	high-mobility group nucleosomal binding domain 2 (HMGN2), mRNA.	0.03	12.22	6.24E-02	8.03E-02	blue
HMHB1	57824	histocompatibility (minor) HB-1 (HMHB1), mRNA.	-0.11	7.57	1.68E-11	4.60E-11	blue

HNRNPA1	3178	heterogeneous nuclear ribonucleoprotein A1 (HNRPA1), transcript variant 1, mRNA.	-0.31	7.50	4.83E-44	5.87E-43	blue
HNRNPA3	220988	heterogeneous nuclear ribonucleoprotein A3 (HNRPA3), mRNA.	0.04	10.55	5.06E-03	7.43E-03	blue
HNRNPH1	3187	heterogeneous nuclear ribonucleoprotein H1 (H) (HNRPH1), mRNA.	-0.15	8.64	9.07E-14	2.81E-13	blue
HNRNPK	3190	heterogeneous nuclear ribonucleoprotein K (HNRPK), transcript variant 3, mRNA.	-0.13	7.57	9.26E-11	2.42E-10	blue
HNRNPM	4670	heterogeneous nuclear ribonucleoprotein M (HNRPM), transcript variant 1, mRNA.	-0.07	6.37	1.67E-07	3.58E-07	blue
HOXC4	3221	homeobox C4 (HOXC4), transcript variant 2, mRNA.	-0.09	9.57	1.22E-08	2.82E-08	blue
HSD17B11	51170	hydroxysteroid (17-beta) dehydrogenase 11 (HSD17B11), mRNA.	-0.18	8.04	2.15E-29	1.36E-28	blue
HSPBP1	23640	hsp70-interacting protein (HSPBP1), mRNA.	0.03	10.70	2.27E-02	3.09E-02	blue
HSPD1	3329	heat shock 60kDa protein 1 (chaperonin) (HSPD1), nuclear gene encoding mitochondrial protein, transcript variant 1, mRNA.	-0.04	7.51	1.33E-02	1.86E-02	blue

HTRA2	27429	HtrA serine peptidase 2 (HTRA2), nuclear gene encoding mitochondrial protein, transcript variant 1, mRNA.	-0.05	6.63	3.09E-04	5.15E-04	blue
ICAM2	3384	intercellular adhesion molecule 2 (ICAM2), mRNA.	0.01	8.79	1.70E-01	2.05E-01	blue
IDH1	3417	isocitrate dehydrogenase 1 (NADP+), soluble (IDH1), mRNA.	-0.21	9.88	6.04E-16	2.11E-15	blue
IFIT3	3437	interferon-induced protein with tetratricopeptide repeats 3 (IFIT3), mRNA.	-0.31	8.13	4.47E-42	5.02E-41	blue
IKZF3	22806	IKAROS family zinc finger 3 (Aiolos) (IKZF3), transcript variant 4, mRNA.	-0.38	6.73	1.79E-35	1.46E-34	blue
IL10RB	3588	interleukin 10 receptor, beta (IL10RB), mRNA.	0.03	8.56	1.43E-02	2.00E-02	blue
ILF3	3609	interleukin enhancer binding factor 3, 90kDa (ILF3), transcript variant 2, mRNA.	-0.24	7.32	1.58E-35	1.30E-34	blue
INPP5B	3633	inositol polyphosphate-5-phosphatase, 75kDa (INPP5B), nuclear gene encoding mitochondrial protein, mRNA.	0.00	10.10	7.82E-01	8.11E-01	blue
INTS10	55174	integrator complex subunit 10 (INTS10), mRNA.	-0.20	7.47	2.87E-25	1.57E-24	blue
IP6K2	51447	inositol hexaphosphate kinase 2 (IHPK2), transcript variant 4, mRNA.	0.17	9.94	1.31E-22	6.32E-22	blue

IPO7	10527	importin 7 (IPO7), mRNA.	-0.14	9.87	4.34E-23	2.15E-22	blue
ITGB1	3688	integrin, beta 1 (fibronectin receptor, beta polypeptide, antigen CD29 includes MDF2, MSK12) (ITGB1), transcript variant 1E, mRNA.	-0.11	7.57	7.23E-10	1.80E-09	blue
ITPK1	3705	inositol 1,3,4-triphosphate 5/6 kinase (ITPK1), mRNA.	0.09	6.92	9.96E-10	2.45E-09	blue
KAT6A	7994	MYST histone acetyltransferase (monocytic leukemia) 3 (MYST3), mRNA.	0.12	9.00	9.94E-18	3.81E-17	blue
KIAA1551	55196	chromosome 12 open reading frame 35 (C12orf35), mRNA.	0.08	7.65	2.53E-07	5.36E-07	blue
KIF1B	23095	kinesin family member 1B (KIF1B), transcript variant 1, mRNA.	0.07	6.25	1.05E-05	1.99E-05	blue
KIF3B	9371	kinesin family member 3B (KIF3B), mRNA.	-0.09	11.45	9.07E-14	2.81E-13	blue
KIF5B	3799	kinesin family member 5B (KIF5B), mRNA.	-0.02	6.80	1.86E-01	2.22E-01	blue
KNSTRN	90417	chromosome 15 open reading frame 23 (C15orf23), mRNA.	0.98	8.51	3.23E-87	4.97E-85	blue
KYNU	8942	kynureninase (L-kynurenine hydrolase) (KYNU), transcript variant 1, mRNA.	0.07	10.55	2.04E-05	3.79E-05	blue
LGALS3	3958	lectin, galactoside-binding, soluble, 3 (LGALS3), transcript variant 1, mRNA.	-0.05	9.51	3.07E-04	5.12E-04	blue

LIMS1	3987	LIM and senescent cell antigen-like domains 1 (LIMS1), mRNA.	-0.17	10.29	4.18E-29	2.62E-28	blue
LLGL1	3996	lethal giant larvae homolog 1 (Drosophila) (LLGL1), mRNA.	-0.01	7.24	3.98E-01	4.44E-01	blue
LLPH	84298	chromosome 12 open reading frame 31 (C12orf31), mRNA.	0.06	7.88	1.68E-04	2.86E-04	blue
LMAN1	3998	lectin, mannose-binding, 1 (LMAN1), mRNA.	0.11	7.81	1.19E-09	2.92E-09	blue
LMNB2	84823	lamin B2 (LMNB2), mRNA.	-0.10	8.57	7.33E-12	2.04E-11	blue
LRFN4	78999	leucine rich repeat and fibronectin type III domain containing 4 (LRFN4), mRNA.	0.00	8.27	7.51E-01	7.82E-01	blue
LRRC40	55631	leucine rich repeat containing 40 (LRRC40), mRNA.	-0.07	7.24	3.81E-06	7.47E-06	blue
LSP1	4046	lymphocyte-specific protein 1 (LSP1), transcript variant 1, mRNA.	-0.13	9.08	1.63E-12	4.71E-12	blue
LTBR	4055	lymphotoxin beta receptor (TNFR superfamily, member 3) (LTBR), mRNA.	0.27	5.76	1.16E-37	1.06E-36	blue
LY9	4063	lymphocyte antigen 9 (LY9), transcript variant 1, mRNA.	-0.06	7.81	2.05E-07	4.37E-07	blue
LYPLA1	10434	lysophospholipase I (LYPLA1), mRNA.	-0.39	10.86	8.08E-39	7.75E-38	blue
LYRM7	90624	hypothetical protein LOC90624 (LOC90624), mRNA.	-0.08	9.89	2.48E-11	6.70E-11	blue
M6PR	4074	mannose-6-phosphate receptor (cation dependent) (M6PR), mRNA.	0.40	5.52	4.06E-23	2.01E-22	blue

MAD1L1	8379	MAD1 mitotic arrest deficient-like 1 (yeast) (MAD1L1), transcript variant 3, mRNA.	0.01	5.73	6.92E-01	7.27E-01	blue
MAD2L1BP	9587	MAD2L1 binding protein (MAD2L1BP), transcript variant 2, mRNA.	-0.01	8.88	4.04E-01	4.49E-01	blue
MAEA	10296	macrophage erythroblast attacher (MAEA), transcript variant 1, mRNA.	0.14	8.11	1.64E-19	6.84E-19	blue
MAP1LC3B	81631	microtubule-associated protein 1 light chain 3 beta (MAP1LC3B), mRNA.	-0.08	8.48	2.07E-13	6.31E-13	blue
MAPKAP1	79109	mitogen-activated protein kinase associated protein 1 (MAPKAP1), transcript variant 6, mRNA.	-0.16	5.81	3.75E-18	1.47E-17	blue
MAT2B	27430	methionine adenosyltransferase II, beta (MAT2B), transcript variant 2, mRNA.	0.18	10.20	1.44E-30	9.47E-30	blue
MBD2	8932	methyl-CpG binding domain protein 2 (MBD2), transcript variant testis-specific, mRNA.	0.07	8.14	6.10E-04	9.87E-04	blue
MCAT	27349	malonyl CoA:ACP acyltransferase (mitochondrial) (MCAT), nuclear gene encoding mitochondrial protein, transcript variant 1, mRNA.	0.07	7.90	1.87E-04	3.18E-04	blue

MCRS1	10445	microspherule protein 1 (MCRS1), transcript variant 1, mRNA.	-0.12	7.45	2.61E-10	6.70E-10	blue
MDM1	56890	Mdm4, transformed 3T3 cell double minute 1, p53 binding protein (mouse) (MDM1), transcript variant 1, mRNA.	0.05	9.39	6.43E-04	1.04E-03	blue
ME2	4200	malic enzyme 2, NAD(+)-dependent, mitochondrial (ME2), nuclear gene encoding mitochondrial protein, mRNA.	-0.14	8.02	3.19E-29	2.01E-28	blue
MED16	10025	thyroid hormone receptor associated protein 5 (THRAP5), mRNA.	0.08	8.00	1.55E-06	3.12E-06	blue
METTL21B	25895	family with sequence similarity 119, member B (FAM119B), transcript variant 1, mRNA.	-0.06	8.89	6.80E-08	1.50E-07	blue
MFGE8	4240	milk fat globule-EGF factor 8 protein (MFGE8), mRNA.	-0.05	7.64	1.90E-03	2.92E-03	blue
MGAT2	4247	mannosyl (alpha-1,6-)-glycoprotein beta-1,2-N-acetylglucosaminyltransferase (MGAT2), transcript variant 1, mRNA.	-0.15	7.76	1.52E-18	6.07E-18	blue
MGST1	4257	microsomal glutathione S-transferase 1 (MGST1), transcript variant 1c, mRNA.	-0.13	11.50	1.52E-16	5.48E-16	blue

MICAL1	64780	microtubule associated monoxygenase, calponin and LIM domain containing 1 (MICAL1), mRNA.	0.01	5.93	6.95E-01	7.29E-01	blue
MINA	84864	MYC induced nuclear antigen (MINA), transcript variant 3, mRNA.	0.15	7.35	2.48E-19	1.03E-18	blue
MIS18BP1	55320	chromosome 14 open reading frame 106 (C14orf106), mRNA.	0.28	9.93	6.25E-40	6.24E-39	blue
MOCS2	4338	molybdenum cofactor synthesis 2 (MOCS2), transcript variant 1, mRNA.	-0.28	7.81	7.85E-37	6.91E-36	blue
MORF4L2	9643	mortality factor 4 like 2 (MORF4L2), mRNA.	-0.02	7.96	1.23E-01	1.52E-01	blue
MPHOSPH6	10200	M-phase phosphoprotein 6 (MPHOSPH6), mRNA.	0.04	10.62	2.67E-04	4.47E-04	blue
MR1	3140	major histocompatibility complex, class I-related (MR1), mRNA.	1.34	6.05	5.87E-59	1.57E-57	blue
MRFAP1	93621	Mof4 family associated protein 1 (MRFAP1), mRNA.	0.13	7.20	1.31E-15	4.47E-15	blue
MRPL18	29074	mitochondrial ribosomal protein L18 (MRPL18), nuclear gene encoding mitochondrial protein, mRNA.	0.01	6.62	5.90E-01	6.32E-01	blue

MRPL30	51263	mitochondrial ribosomal protein L30 (MRPL30), nuclear gene encoding mitochondrial protein, transcript variant 3, mRNA.	0.04	7.36	1.21E-02	1.71E-02	blue
MRPL39	54148	mitochondrial ribosomal protein L39 (MRPL39), nuclear gene encoding mitochondrial protein, transcript variant 1, mRNA.	0.07	8.45	2.73E-07	5.78E-07	blue
MRPL45	84311	mitochondrial ribosomal protein L45 (MRPL45), nuclear gene encoding mitochondrial protein, mRNA.	0.01	6.47	7.13E-01	7.47E-01	blue
MSC	9242	musculin (activated B-cell factor-1) (MSC), mRNA.	0.05	8.32	6.95E-05	1.23E-04	blue
MSMO1	6307	sterol-C4-methyl oxidase-like (SC4MOL), transcript variant 1, mRNA.	0.12	7.75	4.19E-19	1.72E-18	blue
MTAP	4507	methylthioadenosine phosphorylase (MTAP), mRNA.	0.05	7.37	1.28E-03	2.00E-03	blue
MTRR	4552	5-methyltetrahydrofolate-homocysteine methyltransferase reductase (MTRR), transcript variant 2, mRNA.	0.09	6.08	7.20E-09	1.69E-08	blue
MTX1	4580	metaxin 1 (MTX1), transcript variant 1, mRNA.	-0.09	8.89	8.61E-09	2.01E-08	blue

MYL5	84179	PREDICTED: major facilitator superfamily domain containing 7 (MFSD7), mRNA.	0.06	9.48	2.94E-04	4.92E-04	blue
MYO9B	4650	myosin IXB (MYO9B), mRNA.	-0.25	10.22	9.49E-32	6.65E-31	blue
NA	85002	family with sequence similarity 86, member B1 (FAM86B1), mRNA.	0.64	6.33	4.28E-67	1.67E-65	blue
NA	11039	SMA4 (SMA4), mRNA.	0.57	7.34	8.17E-66	2.98E-64	blue
NA	440926	PREDICTED: H3 histone, family 3A pseudogene, transcript variant 3 (LOC440926), misc RNA.	0.49	7.55	1.75E-61	5.27E-60	blue
NA	79008	GIY-YIG domain containing 2 (GIYD2), transcript variant 1, mRNA.	-0.39	7.76	1.88E-60	5.30E-59	blue
NA	6638	small nuclear ribonucleoprotein polypeptide N (SNRPN), transcript variant 5, mRNA.	-0.44	6.78	1.80E-59	4.90E-58	blue
NA	51030	family with sequence similarity 18, member B (FAM18B), mRNA.	0.39	8.46	5.28E-47	7.51E-46	blue
NA	57461	ISY1 splicing factor homolog (S. cerevisiae) (ISY1), mRNA.	-0.26	8.96	3.72E-46	5.08E-45	blue
NA	4831	non-metastatic cells 2, protein (NM23B) expressed in (NME2), transcript variant 3, mRNA.	-0.27	7.27	5.14E-45	6.55E-44	blue

NA	10384	butyrophilin, subfamily 3, member A3 (BTN3A3), transcript variant 1, mRNA.	-0.13	9.82	1.31E-14	4.24E-14	blue
NA	10137	RNA binding motif protein 12 (RBM12), transcript variant 2, mRNA.	-0.11	7.44	3.57E-14	1.13E-13	blue
NA	200316	apolipoprotein B mRNA editing enzyme, catalytic polypeptide-like 3F (APOBEC3F), transcript variant 2, mRNA.	-0.10	7.19	1.19E-13	3.67E-13	blue
NA	4830	non-metastatic cells 1, protein (NM23A) expressed in (NME1), transcript variant 1, mRNA.	0.09	8.10	3.99E-10	1.01E-09	blue
NA	908	chaperonin containing TCP1, subunit 6A (zeta 1) (CCT6A), transcript variant 1, mRNA.	0.09	10.03	9.92E-09	2.31E-08	blue
NA	442535	similar to T-cell receptor gamma chain V region PT-gamma-1/2 precursor (LOC442535), mRNA.	0.05	8.76	1.41E-03	2.19E-03	blue
NA	5901	RAN, member RAS oncogene family (RAN), mRNA.	0.06	8.20	2.35E-03	3.57E-03	blue
NA	375260	CXYorf1-related protein (MGC52000), mRNA.	-0.05	5.94	5.02E-03	7.38E-03	blue
NA	164022	peptidylprolyl isomerase A (cyclophilin A)-like 4 (PPIAL4), mRNA.	0.03	7.74	5.60E-02	7.25E-02	blue

NA	84278	hippocampus abundant gene transcript-like 2 (HIATL2) on chromosome 9.	0.02	8.20	6.43E-02	8.27E-02	blue
NA	653635	PREDICTED: similar to CXYorf1-related protein (LOC653635), mRNA.	-0.02	7.04	1.51E-01	1.82E-01	blue
NA	728554	PREDICTED: similar to THO complex 3 (LOC728554), mRNA.	0.00	5.82	8.96E-01	9.13E-01	blue
NACA	4666	nascent-polypeptide-associated complex alpha polypeptide (NACA), mRNA.	-0.10	6.37	2.05E-09	4.97E-09	blue
NCAPD2	9918	non-SMC condensin I complex, subunit D2 (NCAPD2), mRNA.	-0.04	6.60	4.76E-02	6.23E-02	blue
NCF1	653361	neutrophil cytosolic factor 1, (chronic granulomatous disease, autosomal 1) (NCF1), mRNA.	-0.13	7.04	1.18E-15	4.05E-15	blue
NCL	4691	nucleolin (NCL), mRNA.	0.11	9.68	2.47E-16	8.79E-16	blue
NDUFV3	4731	NADH dehydrogenase (ubiquinone) flavoprotein 3, 10kDa (NDUFV3), nuclear gene encoding mitochondrial protein, transcript variant 1, mRNA.	0.04	12.16	1.07E-02	1.52E-02	blue
NEMF	9147	serologically defined colon cancer antigen 1 (SDCCAG1), mRNA.	0.04	9.36	3.16E-02	4.22E-02	blue

NFKBIB	4793	nuclear factor of kappa light polypeptide gene enhancer in B-cells inhibitor, beta (NFKBIB), transcript variant 1, mRNA.	-0.13	6.47	8.14E-19	3.28E-18	blue
NGRN	51335	neugrin, neurite outgrowth associated (NGRN), transcript variant 1, mRNA.	-0.34	8.59	3.37E-50	5.32E-49	blue
NOC4L	79050	nucleolar complex associated 4 homolog (S. cerevisiae) (NOC4L), mRNA.	0.13	7.87	1.57E-13	4.79E-13	blue
NOMO1	23420	NODAL modulator 1 (NOMO1), mRNA.	-0.17	7.01	4.22E-26	2.39E-25	blue
NRDE2	55051	chromosome 14 open reading frame 102 (C14orf102), transcript variant 1, mRNA.	-0.07	7.95	4.73E-05	8.47E-05	blue
NUP35	129401	nucleoporin 35kDa (NUP35), transcript variant 1, mRNA.	0.00	7.28	9.93E-01	9.94E-01	blue
NUP50	10762	nucleoporin 50kDa (NUP50), transcript variant 3, mRNA.	0.08	7.24	2.00E-07	4.29E-07	blue
OAS2	4939	2'-5'-oligoadenylate synthetase 2, 69/71kDa (OAS2), transcript variant 1, mRNA.	0.10	8.27	1.83E-11	4.99E-11	blue
OPN3	23596	opsin 3 (encephalopsin, panopsin) (OPN3), transcript variant 1, mRNA.	-0.10	8.80	2.62E-09	6.31E-09	blue
ORC5	5001	origin recognition complex, subunit 5-like (yeast) (ORC5L), transcript variant 1, mRNA.	-0.09	8.17	1.14E-12	3.34E-12	blue

ORC6	23594	origin recognition complex, subunit 6 like (yeast) (ORC6L), mRNA.	-0.05	8.75	2.24E-06	4.46E-06	blue
OSTC	58505	DC2 protein (DC2), mRNA.	0.04	7.08	5.41E-02	7.02E-02	blue
OSTF1	26578	osteoclast stimulating factor 1 (OSTF1), mRNA.	0.05	7.67	9.06E-05	1.58E-04	blue
PAAF1	80227	WD repeat domain 71 (WDR71), mRNA.	-0.77	7.45	4.01E-82	3.99E-80	blue
PABPC3	5042	poly(A) binding protein, cytoplasmic 3 (PABPC3), mRNA.	0.10	8.65	5.30E-09	1.26E-08	blue
PAG1	55824	phosphoprotein associated with glycosphingolipid microdomains 1 (PAG1), mRNA.	-0.06	11.57	1.54E-05	2.89E-05	blue
PARVB	29780	parvin, beta (PARVB), transcript variant 2, mRNA.	-0.22	7.06	4.12E-28	2.52E-27	blue
PCBP2	5094	poly(rC) binding protein 2 (PCBP2), transcript variant 2, mRNA.	0.10	7.89	1.40E-07	3.02E-07	blue
PCGF6	84108	polycomb group ring finger 6 (PCGF6), transcript variant 1, mRNA.	0.00	5.86	7.84E-01	8.12E-01	blue
PDCD10	11235	programmed cell death 10 (PDCD10), transcript variant 2, mRNA.	0.08	6.77	1.88E-11	5.13E-11	blue
PDCD5	9141	programmed cell death 5 (PDCD5), mRNA.	-0.21	6.75	1.07E-36	9.31E-36	blue
PDIA6	10130	protein disulfide isomerase family A, member 6 (PDIA6), mRNA.	0.14	10.41	2.37E-24	1.25E-23	blue

PDPK1	5170	3-phosphoinositide dependent protein kinase-1 (PDPK1), transcript variant 1, mRNA.	-0.45	8.04	1.09E-75	6.65E-74	blue
PDXDC1	23042	PREDICTED: KIAA0251 protein (KIAA0251), mRNA.	-0.13	10.47	5.43E-13	1.62E-12	blue
PEMT	10400	phosphatidylethanolamine N-methyltransferase (PEMT), nuclear gene encoding mitochondrial protein, transcript variant 1, mRNA.	0.06	8.27	6.70E-07	1.38E-06	blue
PEX6	5190	peroxisomal biogenesis factor 6 (PEX6), mRNA.	0.15	8.34	1.17E-22	5.65E-22	blue
PFN1	5216	profilin 1 (PFN1), mRNA.	0.03	10.46	7.50E-03	1.08E-02	blue
PGAM1	643576	PREDICTED: similar to Phosphoglycerate mutase 1 (Phosphoglycerate mutase isozyme B) (PGAM-B) (BPG-dependent PGAM 1), transcript variant 1 (LOC643576), mRNA.	-0.31	6.79	1.59E-47	2.32E-46	blue
PGAM4	728188	PREDICTED: similar to Probable phosphoglycerate mutase 4 (LOC728188), mRNA.	0.13	8.50	3.44E-12	9.79E-12	blue
PGD	5226	phosphogluconate dehydrogenase (PGD), mRNA.	0.02	7.62	3.01E-01	3.45E-01	blue
PGK1	5230	phosphoglycerate kinase 1 (PGK1), mRNA.	-0.05	9.06	1.81E-03	2.79E-03	blue
PGM1	5236	phosphoglucomutase 1 (PGM1), mRNA.	-0.01	7.66	4.61E-01	5.07E-01	blue

PHAX	51808	RNA U, small nuclear RNA export adaptor (phosphorylation regulated) (RNUXA), mRNA.	-0.06	9.92	6.83E-07	1.41E-06	blue
PHF5A	84844	PHD finger protein 5A (PHF5A), mRNA.	-0.03	7.61	5.35E-02	6.94E-02	blue
PI4KB	5298	phosphatidylinositol 4-kinase, catalytic, beta polypeptide (PIK4CB), mRNA.	-0.28	8.73	3.60E-39	3.48E-38	blue
PIGK	10026	phosphatidylinositol glycan anchor biosynthesis, class K (PIGK), mRNA.	-0.03	8.65	3.34E-02	4.44E-02	blue
PKMYT1	9088	protein kinase, membrane associated tyrosine/threonine 1 (PKMYT1), transcript variant 1, mRNA.	0.04	9.68	1.32E-02	1.85E-02	blue
PLEKHB2	55041	pleckstrin homology domain containing, family B (evectins) member 2 (PLEKHB2), transcript variant 1, mRNA.	0.12	6.43	3.16E-15	1.06E-14	blue
PLIN3	10226	mannose-6-phosphate receptor binding protein 1 (M6PRBP1), mRNA.	0.45	8.19	4.59E-51	7.49E-50	blue
PMPCA	23203	peptidase (mitochondrial processing) alpha (PMPCA), nuclear gene encoding mitochondrial protein, mRNA.	0.04	6.08	9.09E-03	1.30E-02	blue
POGK	57645	pogo transposable element with KRAB domain (POGK), mRNA.	-0.04	8.22	2.13E-03	3.25E-03	blue

POGLUT1	56983	KTEL (Lys-Tyr-Glu-Leu) containing 1 (KTELC1), mRNA.	0.00	7.72	8.26E-01	8.50E-01	blue
POLD3	10714	polymerase (DNA-directed), delta 3, accessory subunit (POLD3), mRNA.	-0.04	9.34	9.07E-04	1.45E-03	blue
POLE4	56655	polymerase (DNA-directed), epsilon 4 (p12 subunit) (POLE4), mRNA.	-0.41	8.81	5.09E-51	8.25E-50	blue
POLR1E	64425	polymerase (RNA) I polypeptide E, 53kDa (POLR1E), mRNA.	0.22	6.05	6.09E-26	3.42E-25	blue
POLR2E	5434	polymerase (RNA) II (DNA directed) polypeptide E, 25kDa (POLR2E), mRNA.	0.05	9.55	2.66E-04	4.46E-04	blue
POLR2J3	548644	RPB11b2alpha protein (POLR2J3), mRNA.	-0.15	8.73	3.44E-27	2.04E-26	blue
POMP	51371	proteasome maturation protein (POMP), mRNA.	-0.11	7.06	3.23E-17	1.20E-16	blue
POTEKP	440915	actin-like protein (FKSG30), mRNA.	0.00	5.68	9.48E-01	9.55E-01	blue
PPIA	5478	peptidylprolyl isomerase A (cyclophilin A) (PPIA), mRNA.	-0.12	6.09	7.40E-13	2.19E-12	blue
PPID	5481	peptidylprolyl isomerase D (cyclophilin D) (PPID), mRNA.	0.14	6.60	1.60E-19	6.67E-19	blue
PPIE	10450	peptidylprolyl isomerase E (cyclophilin E) (PPIE), transcript variant 2, mRNA.	-0.14	7.23	1.56E-24	8.28E-24	blue
PPIL3	53938	peptidylprolyl isomerase (cyclophilin)-like 3 (PPIL3), transcript variant PPIL3c, mRNA.	0.03	8.75	2.33E-02	3.17E-02	blue

PPM1K	152926	protein phosphatase 1K (PP2C domain containing) (PPM1K), mRNA.	-0.09	7.94	5.52E-09	1.31E-08	blue
PPP1CA	5499	protein phosphatase 1, catalytic subunit, alpha isoform (PPP1CA), transcript variant 3, mRNA.	0.21	7.62	3.32E-16	1.17E-15	blue
PPP1R2	5504	protein phosphatase 1, regulatory (inhibitor) subunit 2 (PPP1R2), mRNA.	0.25	5.77	1.02E-22	4.98E-22	blue
PPP1R7	5510	protein phosphatase 1, regulatory (inhibitor) subunit 7 (PPP1R7), mRNA.	0.00	8.96	7.14E-01	7.48E-01	blue
PPP2R2A	5520	protein phosphatase 2 (formerly 2A), regulatory subunit B, alpha isoform (PPP2R2A), mRNA.	-0.01	8.65	2.01E-01	2.39E-01	blue
PPP2R2D	55844	protein phosphatase 2, regulatory subunit B, delta isoform (PPP2R2D), transcript variant 1, mRNA.	-0.01	8.16	6.20E-01	6.61E-01	blue
PPP2R5C	5527	protein phosphatase 2, regulatory subunit B', gamma isoform (PPP2R5C), transcript variant 4, mRNA.	-0.01	8.52	2.09E-01	2.47E-01	blue
PPP4R2	151987	protein phosphatase 4, regulatory subunit 2 (PPP4R2), mRNA.	0.05	8.89	3.04E-05	5.56E-05	blue
PPTC7	160760	PTC7 protein phosphatase homolog (S. cerevisiae) (PPTC7), mRNA.	0.01	8.30	4.67E-01	5.13E-01	blue

PRDX3	10935	peroxiredoxin 3 (PRDX3), nuclear gene encoding mitochondrial protein, transcript variant 2, mRNA.	0.05	7.90	2.18E-05	4.01E-05	blue
PRKAG2	51422	protein kinase, AMP-activated, gamma 2 non-catalytic subunit (PRKAG2), transcript variant b, mRNA.	-0.01	7.65	3.76E-01	4.21E-01	blue
PRKAR1B	5575	protein kinase, cAMP-dependent, regulatory, type I, beta (PRKAR1B), mRNA.	-0.19	6.19	5.04E-30	3.26E-29	blue
PRKCB	5579	protein kinase C, beta 1 (PRKCB1), transcript variant 1, mRNA.	0.10	7.28	3.06E-10	7.78E-10	blue
PRPF6	24148	PRP6 pre-mRNA processing factor 6 homolog (S. cerevisiae) (PRPF6), mRNA.	-0.48	6.41	1.31E-53	2.35E-52	blue
PRR11	55771	proline rich 11 (PRR11), mRNA.	0.02	6.32	1.16E-01	1.44E-01	blue
PRR3	80742	proline rich 3 (PRR3), transcript variant 1, mRNA.	0.04	7.79	6.01E-04	9.74E-04	blue
PRRC2C	23215	BAT2 domain containing 1 (BAT2D1), mRNA.	-0.04	8.05	1.04E-04	1.81E-04	blue
PRSS21	10942	protease, serine, 21 (testisin) (PRSS21), transcript variant 2, mRNA.	0.04	8.06	4.49E-02	5.90E-02	blue
PRTFDC1	56952	phosphoribosyl transferase domain containing 1 (PRTFDC1), mRNA.	0.09	7.85	1.00E-11	2.77E-11	blue

PSMA1	5682	proteasome (prosome, macropain) subunit, alpha type, 1 (PSMA1), transcript variant 2, mRNA.	-0.21	7.79	6.85E-18	2.65E-17	blue
PSMC4	5704	proteasome (prosome, macropain) 26S subunit, ATPase, 4 (PSMC4), transcript variant 2, mRNA.	0.17	7.08	3.78E-20	1.64E-19	blue
PSMD6	9861	proteasome (prosome, macropain) 26S subunit, non-ATPase, 6 (PSMD6), mRNA.	0.03	8.24	8.99E-02	1.13E-01	blue
PSPH	5723	phosphoserine phosphatase (PSPH), mRNA.	-0.03	8.01	5.53E-02	7.16E-02	blue
PTP4A1	7803	protein tyrosine phosphatase type IVA, member 1 (PTP4A1), mRNA.	-0.03	8.57	4.50E-03	6.65E-03	blue
PTPRC	5788	protein tyrosine phosphatase, receptor type, C (PTPRC), transcript variant 4, mRNA.	1.16	7.08	9.34E-77	6.08E-75	blue
PTTG1	9232	pituitary tumor-transforming 1 (PTTG1), mRNA.	0.05	9.89	1.19E-03	1.86E-03	blue
PTTG1IP	754	pituitary tumor-transforming 1 interacting protein (PTTG1IP), mRNA.	-0.01	8.27	5.15E-01	5.60E-01	blue
RAB28	9364	RAB28, member RAS oncogene family (RAB28), transcript variant 2, mRNA.	0.13	6.31	9.39E-13	2.75E-12	blue
RABL3	285282	RAB, member of RAS oncogene family-like 3 (RABL3), mRNA.	0.40	10.51	5.88E-46	7.91E-45	blue

RAC2	5880	ras-related C3 botulinum toxin substrate 2 (rho family, small GTP binding protein Rac2) (RAC2), mRNA.	-0.11	10.02	2.29E-11	6.22E-11	blue
RAD21	5885	RAD21 homolog (S. pombe) (RAD21), mRNA.	-0.05	8.82	4.04E-04	6.67E-04	blue
RAD51	5888	RAD51 homolog (RecA homolog, E. coli) (S. cerevisiae) (RAD51), transcript variant 1, mRNA.	0.68	6.42	8.99E-52	1.51E-50	blue
RAD51C	5889	RAD51 homolog C (S. cerevisiae) (RAD51C), transcript variant 2, mRNA.	0.32	5.49	2.08E-16	7.44E-16	blue
RAE1	8480	RAE1 RNA export 1 homolog (S. pombe) (RAE1), transcript variant 1, mRNA.	-0.15	6.93	7.09E-19	2.88E-18	blue
RALB	5899	v-ral simian leukemia viral oncogene homolog B (ras related; GTP binding protein) (RALB), mRNA.	-0.21	9.14	5.89E-28	3.59E-27	blue
RANBP1	5902	RAN binding protein 1 (RANBP1), mRNA.	0.13	6.93	2.45E-08	5.55E-08	blue
RBBP5	5929	retinoblastoma binding protein 5 (RBBP5), mRNA.	-0.13	9.17	6.62E-16	2.30E-15	blue
RBM3	5935	RNA binding motif (RNP1, RRM) protein 3 (RBM3), transcript variant 3, mRNA.	-0.13	6.92	2.29E-15	7.71E-15	blue
RBM4	5936	RNA binding motif protein 4 (RBM4), mRNA.	-0.11	6.11	1.62E-11	4.45E-11	blue

RECQL	5965	RecQ protein-like (DNA helicase Q1-like) (RECQL), transcript variant 2, mRNA.	0.12	7.50	1.65E-17	6.26E-17	blue
RFC2	5982	replication factor C (activator 1) 2, 40kDa (RFC2), transcript variant 1, mRNA.	-0.66	6.26	3.48E-63	1.15E-61	blue
RFC3	5983	replication factor C (activator 1) 3, 38kDa (RFC3), transcript variant 2, mRNA.	-0.51	8.17	9.15E-56	1.92E-54	blue
RFXANK	8625	regulatory factor X-associated ankyrin-containing protein (RFXANK), transcript variant 1, mRNA.	0.01	7.61	5.80E-01	6.23E-01	blue
RHPN2	85415	rhophilin, Rho GTPase binding protein 2 (RHPN2), mRNA.	0.05	5.74	1.16E-03	1.82E-03	blue
RILPL2	728069	PREDICTED: similar to T-box 1 isoform C (LOC728069), mRNA.	0.23	6.59	1.99E-36	1.71E-35	blue
RIOK1	83732	RIO kinase 1 (yeast) (RIOK1), transcript variant 1, mRNA.	-0.60	6.70	1.31E-83	1.51E-81	blue
RMDN1	642197	PREDICTED: similar to Protein FAM82B (LOC642197), mRNA.	0.03	5.70	1.28E-01	1.58E-01	blue
RNF130	55819	ring finger protein 130 (RNF130), mRNA.	0.09	7.50	1.25E-06	2.53E-06	blue
RNF4	6047	ring finger protein 4 (RNF4), mRNA.	0.12	8.36	4.25E-20	1.83E-19	blue

ROCK1	6093	Rho-associated, coiled-coil containing protein kinase 1 (ROCK1), mRNA.	0.00	10.29	7.30E-01	7.62E-01	blue
RPA1	6117	replication protein A1, 70kDa (RPA1), mRNA.	0.01	7.97	3.37E-01	3.82E-01	blue
RPL10A	4736	ribosomal protein L10a (RPL10A), mRNA.	0.01	7.48	7.01E-01	7.35E-01	blue
RPL13	6137	ribosomal protein L13 (RPL13), transcript variant 2, mRNA.	-0.10	9.80	1.60E-11	4.38E-11	blue
RPL13A	23521	ribosomal protein L13a (RPL13A), mRNA.	0.10	9.05	2.77E-10	7.07E-10	blue
RPL15	6138	ribosomal protein L15 (RPL15), mRNA.	-0.04	8.39	7.07E-03	1.02E-02	blue
RPL17	6139	ribosomal protein L17 (RPL17), transcript variant 1, mRNA.	0.15	6.27	6.91E-14	2.16E-13	blue
RPL18	441775	PREDICTED: similar to 60S ribosomal protein L18 (LOC441775), mRNA.	-0.18	6.24	2.66E-16	9.46E-16	blue
RPL22	6146	ribosomal protein L22 (RPL22), mRNA.	0.37	8.93	1.66E-77	1.17E-75	blue
RPL24	731365	PREDICTED: similar to ribosomal protein L24 (LOC731365), mRNA.	-0.32	9.44	7.89E-59	2.10E-57	blue
RPL7L1	731390	PREDICTED: similar to ribosomal protein L7-like 1 (LOC731390), mRNA.	-0.12	9.20	1.91E-12	5.49E-12	blue
RPL8	6132	ribosomal protein L8 (RPL8), transcript variant 2, mRNA.	0.00	8.52	8.49E-01	8.72E-01	blue

RPLP0	6175	ribosomal protein, large, P0 (RPLP0), transcript variant 1, mRNA.	0.09	6.85	2.46E-11	6.66E-11	blue
RPS4X	6191	ribosomal protein S4, X-linked (RPS4X), mRNA.	0.00	10.10	8.03E-01	8.29E-01	blue
RPS6KB2	6199	ribosomal protein S6 kinase, 70kDa, polypeptide 2 (RPS6KB2), transcript variant 2, mRNA.	-0.04	5.71	5.33E-03	7.81E-03	blue
RRAS2	22800	related RAS viral (r-ras) oncogene homolog 2 (RRAS2), mRNA.	0.26	8.88	1.23E-37	1.13E-36	blue
RRM2	6241	ribonucleotide reductase M2 polypeptide (RRM2), mRNA.	-0.15	9.56	3.40E-26	1.93E-25	blue
RRP7A	27341	CGI-96 protein (CGI-96), mRNA.	0.42	7.62	1.19E-59	3.27E-58	blue
RSRC1	51319	arginine/serine-rich coiled-coil 1 (RSRC1), mRNA.	-0.14	7.99	3.32E-23	1.65E-22	blue
RSU1	6251	Ras suppressor protein 1 (RSU1), transcript variant 1, mRNA.	2.11	8.93	3.21E-113	3.26E-110	blue
RTN3	10313	reticulon 3 (RTN3), transcript variant 1, mRNA.	-0.39	8.74	1.21E-54	2.36E-53	blue
S100A13	6284	S100 calcium binding protein A13 (S100A13), transcript variant 2, mRNA.	-0.05	7.92	1.07E-02	1.51E-02	blue
SDAD1	55153	SDA1 domain containing 1 (SDAD1), mRNA.	-0.09	7.76	9.98E-06	1.90E-05	blue

SDHA	6389	succinate dehydrogenase complex, subunit A, flavoprotein (Fp) (SDHA), nuclear gene encoding mitochondrial protein, mRNA.	-0.06	7.59	1.30E-04	2.23E-04	blue
SDHAF3	57001	ACN9 homolog (S. cerevisiae) (ACN9), mRNA.	0.03	6.71	2.76E-02	3.72E-02	blue
SEH1L	81929	SEH1-like (S. cerevisiae) (SEH1L), transcript variant 2, mRNA.	0.11	9.63	1.07E-12	3.15E-12	blue
SERBP1	26135	SERPINE1 mRNA binding protein 1 (SERBP1), transcript variant 3, mRNA.	-0.03	7.73	4.35E-02	5.73E-02	blue
SETD3	84193	SET domain containing 3 (SETD3), transcript variant 2, mRNA.	0.29	8.66	3.63E-25	1.98E-24	blue
SF3B1	23451	splicing factor 3b, subunit 1, 155kDa (SF3B1), transcript variant 2, mRNA.	-0.26	7.51	1.34E-29	8.50E-29	blue
SFR1	119392	chromosome 10 open reading frame 78 (C10orf78), transcript variant 2, mRNA.	0.29	6.01	2.68E-32	1.93E-31	blue
SKP1	6500	S-phase kinase-associated protein 1A (p19A) (SKP1A), transcript variant 2, mRNA.	-0.08	7.62	7.27E-11	1.92E-10	blue
SLC10A7	84068	solute carrier family 10 (sodium/bile acid cotransporter family), member 7 (SLC10A7), transcript variant 2, mRNA.	-0.07	6.13	3.73E-06	7.32E-06	blue

SLC25A15	10166	solute carrier family 25 (mitochondrial carrier; ornithine transporter) member 15 (SLC25A15), nuclear gene encoding mitochondrial protein, mRNA.	-0.22	8.06	1.60E-37	1.45E-36	blue
SLC25A20	788	solute carrier family 25 (carnitine/acylcarnitine translocase), member 20 (SLC25A20), nuclear gene encoding mitochondrial protein, mRNA.	-0.25	6.51	9.27E-38	8.54E-37	blue
SLC25A26	115286	solute carrier family 25, member 26 (SLC25A26), mRNA.	-0.02	6.56	7.09E-02	9.07E-02	blue
SLC25A3	5250	solute carrier family 25 (mitochondrial carrier; phosphate carrier), member 3 (SLC25A3), nuclear gene encoding mitochondrial protein, transcript variant 3, mRNA.	0.09	9.11	1.77E-08	4.06E-08	blue
SLC25A36	55186	solute carrier family 25, member 36 (SLC25A36), mRNA.	0.05	8.08	1.32E-02	1.85E-02	blue
SLC30A7	148867	solute carrier family 30 (zinc transporter), member 7 (SLC30A7), mRNA.	-0.02	5.55	2.21E-01	2.61E-01	blue

SLC35A1	10559	solute carrier family 35 (CMP-sialic acid transporter), member A1 (SLC35A1), mRNA.	0.04	11.50	7.07E-05	1.25E-04	blue
SLC39A3	29985	solute carrier family 39 (zinc transporter), member 3 (SLC39A3), transcript variant 1, mRNA.	0.11	8.51	3.03E-11	8.15E-11	blue
SLC39A6	25800	solute carrier family 39 (zinc transporter), member 6 (SLC39A6), mRNA.	-0.05	8.55	6.10E-04	9.87E-04	blue
SLC3A2	6520	solute carrier family 3 (activators of dibasic and neutral amino acid transport), member 2 (SLC3A2), transcript variant 6, mRNA.	-0.18	6.70	1.07E-25	5.97E-25	blue
SLC50A1	55974	recombination activating gene 1 activating protein 1 (RAG1AP1), mRNA.	0.02	9.54	5.86E-02	7.57E-02	blue
SLC5A6	8884	solute carrier family 5 (sodium-dependent vitamin transporter), member 6 (SLC5A6), mRNA.	-0.05	7.14	6.05E-05	1.07E-04	blue
SLFN11	91607	schlafen family member 11 (SLFN11), mRNA.	0.07	9.22	9.91E-04	1.57E-03	blue
SLFN5	162394	schlafen family member 5 (SLFN5), mRNA.	0.04	8.46	2.17E-02	2.96E-02	blue

SLTM	79811	SAFB-like, transcription modulator (SLTM), transcript variant 1, mRNA.	-0.10	7.42	7.08E-09	1.67E-08	blue
SMARCE1	6605	SWI/SNF related, matrix associated, actin dependent regulator of chromatin, subfamily e, member 1 (SMARCE1), mRNA.	-0.06	6.83	3.49E-04	5.79E-04	blue
SMS	646347	PREDICTED: similar to spermine synthase (LOC646347), mRNA.	0.14	6.04	9.90E-17	3.62E-16	blue
SMU1	55234	smu-1 suppressor of mec-8 and unc-52 homolog (C. elegans) (SMU1), mRNA.	0.03	8.12	9.59E-03	1.37E-02	blue
SMYD2	56950	SET and MYND domain containing 2 (SMYD2), mRNA.	-0.07	10.13	2.00E-08	4.57E-08	blue
SNAP23	8773	synaptosomal-associated protein, 23kDa (SNAP23), transcript variant 1, mRNA.	-0.17	9.35	2.02E-20	8.79E-20	blue
SNRPG	6637	small nuclear ribonucleoprotein polypeptide G (SNRPG), mRNA.	-0.16	9.66	1.61E-22	7.74E-22	blue
SNX5	27131	sorting nexin 5 (SNX5), transcript variant 1, mRNA.	0.23	8.53	6.73E-31	4.49E-30	blue
SP140	11262	SP140 nuclear body protein (SP140), transcript variant 2, mRNA.	-0.13	8.92	2.16E-13	6.56E-13	blue
SPACA3	124912	sperm acrosome associated 3 (SPACA3), mRNA.	0.02	7.73	4.98E-02	6.49E-02	blue

SPATA18	132671	spermatogenesis associated 18 homolog (rat) (SPATA18), mRNA.	-0.20	8.87	2.95E-37	2.63E-36	blue
SPATA20	64847	spermatogenesis associated 20 (SPATA20), mRNA.	-0.15	7.54	1.08E-20	4.75E-20	blue
SPATC1L	84221	chromosome 21 open reading frame 56 (C21orf56), mRNA.	-0.09	6.55	2.52E-09	6.09E-09	blue
SPRED2	200734	sprouty-related, EVH1 domain containing 2 (SPRED2), mRNA.	0.01	8.42	2.38E-01	2.78E-01	blue
SPTBN1	6711	spectrin, beta, non-erythrocytic 1 (SPTBN1), transcript variant 1, mRNA.	-0.31	9.29	1.13E-47	1.66E-46	blue
SPTLC1	10558	serine palmitoyltransferase, long chain base subunit 1 (SPTLC1), transcript variant 1, mRNA.	-0.10	8.16	7.45E-08	1.64E-07	blue
SQLE	6713	squalene epoxidase (SQLE), mRNA.	0.02	9.49	3.04E-02	4.07E-02	blue
SRP9	6726	signal recognition particle 9kDa (SRP9), mRNA.	-0.03	10.20	3.75E-03	5.58E-03	blue
SRPK2	6733	SFRS protein kinase 2 (SRPK2), transcript variant 2, mRNA.	-0.37	9.10	9.48E-58	2.25E-56	blue
SRSF10	10772	FUS interacting protein (serine/arginine-rich) 1 (FUSIP1), transcript variant 1, mRNA.	-0.32	7.43	5.03E-55	1.01E-53	blue
SRSF7	6432	splicing factor, arginine/serine-rich 7, 35kDa (SFRS7), mRNA.	0.03	7.70	8.10E-02	1.03E-01	blue

SS18	6760	synovial sarcoma translocation, chromosome 18 (SS18), transcript variant 1, mRNA.	-0.08	7.68	7.85E-05	1.38E-04	blue
SSR2	6746	signal sequence receptor, beta (translocon-associated protein beta) (SSR2), mRNA.	-0.03	8.80	1.25E-01	1.54E-01	blue
SSR3	6747	signal sequence receptor, gamma (translocon-associated protein gamma) (SSR3), mRNA.	0.01	7.66	6.56E-01	6.94E-01	blue
SSSCA1	10534	Sjogren's syndrome/scleroderma autoantigen 1 (SSSCA1), mRNA.	0.08	10.43	9.12E-07	1.86E-06	blue
STAT6	6778	signal transducer and activator of transcription 6, interleukin-4 induced (STAT6), mRNA.	-0.04	9.01	7.66E-03	1.11E-02	blue
STRN3	29966	striatin, calmodulin binding protein 3 (STRN3), mRNA.	0.03	7.84	3.51E-02	4.66E-02	blue
STT3B	201595	STT3, subunit of the oligosaccharyltransferase complex, homolog B (S. cerevisiae) (STT3B), mRNA.	-0.08	7.32	1.80E-09	4.38E-09	blue
SUMF2	25870	sulfatase modifying factor 2 (SUMF2), transcript variant 3, mRNA.	0.16	6.58	2.04E-21	9.27E-21	blue
SUZ12	23512	suppressor of zeste 12 homolog (Drosophila) (SUZ12), mRNA.	-0.02	6.71	3.77E-01	4.23E-01	blue
SYPL1	6856	synaptophysin-like 1 (SYPL1), transcript variant 2, mRNA.	0.04	8.45	8.95E-03	1.28E-02	blue

TAF5L	27097	TAF5-like RNA polymerase II, p300/CBP-associated factor (PCAF)-associated factor, 65kDa (TAF5L), transcript variant 1, mRNA.	0.02	6.17	1.68E-01	2.02E-01	blue
TAGLN2	8407	transgelin 2 (TAGLN2), mRNA.	-0.11	7.19	1.66E-14	5.35E-14	blue
TAMM41	132001	chromosome 3 open reading frame 31 (C3orf31), mRNA.	-0.02	7.66	2.90E-01	3.33E-01	blue
TAOK1	57551	TAO kinase 1 (TAOK1), mRNA.	0.04	8.82	2.58E-03	3.90E-03	blue
TAP2	6891	transporter 2, ATP-binding cassette, sub-family B (MDR/TAP) (TAP2), transcript variant 2, mRNA.	0.05	6.84	3.52E-03	5.26E-03	blue
TCEA1	6917	transcription elongation factor A (SII), 1 (TCEA1), transcript variant 1, mRNA.	-0.14	7.36	2.00E-15	6.72E-15	blue
TCFL5	10732	transcription factor-like 5 (basic helix-loop-helix) (TCFL5), mRNA.	0.00	6.55	9.33E-01	9.44E-01	blue
TCP1	6950	t-complex 1 (TCP1), transcript variant 1, mRNA.	-0.32	6.13	2.48E-49	3.82E-48	blue
TCTN3	26123	chromosome 10 open reading frame 61 (C10orf61), transcript variant 1, mRNA.	0.22	8.07	3.17E-26	1.80E-25	blue
TECR	9524	glycoprotein, synaptic 2 (GPSN2), mRNA.	0.10	6.16	1.80E-08	4.12E-08	blue
TES	26136	testis derived transcript (3 LIM domains) (TES), transcript variant 2, mRNA.	0.04	5.75	1.02E-02	1.44E-02	blue

TEX2	55852	testis expressed sequence 2 (TEX2), mRNA.	-0.07	8.54	2.37E-06	4.72E-06	blue
THAP1	55145	THAP domain containing, apoptosis associated protein 1 (THAP1), transcript variant 1, mRNA.	0.02	7.86	2.02E-01	2.40E-01	blue
TIA1	7072	TIA1 cytotoxic granule-associated RNA binding protein (TIA1), transcript variant 1, mRNA.	0.12	8.65	1.22E-10	3.17E-10	blue
TMED10	10972	transmembrane emp24-like trafficking protein 10 (yeast) (TMED10), mRNA.	-0.07	7.01	1.41E-05	2.66E-05	blue
TMED4	222068	transmembrane emp24 protein transport domain containing 4 (TMED4), mRNA.	0.04	8.20	1.97E-03	3.02E-03	blue
TMEM106C	79022	transmembrane protein 106C (TMEM106C), mRNA.	0.16	7.34	1.44E-17	5.48E-17	blue
TMEM14A	28978	transmembrane protein 14A (TMEM14A), mRNA.	0.02	11.53	1.45E-01	1.76E-01	blue
TMEM156	80008	transmembrane protein 156 (TMEM156), mRNA.	0.00	5.87	7.90E-01	8.17E-01	blue
TMEM30A	55754	transmembrane protein 30A (TMEM30A), mRNA.	0.01	7.81	5.16E-01	5.60E-01	blue
TMEM38B	55151	transmembrane protein 38B (TMEM38B), mRNA.	-0.21	9.78	3.88E-35	3.12E-34	blue
TMEM69	51249	transmembrane protein 69 (TMEM69), mRNA.	-0.11	5.64	2.25E-11	6.11E-11	blue

TMPRSS3	64699	transmembrane protease, serine 3 (TMPRSS3), transcript variant D, mRNA.	-0.23	6.40	1.66E-28	1.03E-27	blue
TNFRSF1A	7132	tumor necrosis factor receptor superfamily, member 1A (TNFRSF1A), mRNA.	-0.19	7.84	1.22E-21	5.63E-21	blue
TNPO1	3842	transportin 1 (TNPO1), transcript variant 2, mRNA.	0.03	11.00	8.48E-03	1.22E-02	blue
TNPO3	23534	transportin 3 (TNPO3), mRNA.	0.08	8.16	1.89E-06	3.78E-06	blue
TOP1	7150	topoisomerase (DNA) I (TOP1), mRNA.	0.05	9.11	1.07E-05	2.04E-05	blue
TOP2B	7155	topoisomerase (DNA) II beta 180kDa (TOP2B), mRNA.	0.04	10.42	6.83E-02	8.76E-02	blue
TPI1	7167	triosephosphate isomerase 1 (TPI1), mRNA.	0.13	7.88	5.89E-12	1.65E-11	blue
TPM3P9	147804	tropomyosin 3 pseudogene (LOC147804) on chromosome 19.	-0.05	8.52	5.83E-03	8.50E-03	blue
TRIM5	85363	tripartite motif-containing 5 (TRIM5), transcript variant alpha, mRNA.	0.14	8.50	1.03E-12	3.00E-12	blue
TRIM61	391712	tripartite motif-containing 61 (TRIM61), mRNA.	0.12	7.15	2.24E-12	6.44E-12	blue
TRIP13	9319	thyroid hormone receptor interactor 13 (TRIP13), mRNA.	0.01	8.98	3.62E-01	4.07E-01	blue
TSFM	10102	Ts translation elongation factor, mitochondrial (TSFM), mRNA.	-0.14	6.29	1.77E-21	8.07E-21	blue

TSNAX	7257	translin-associated factor X (TSNAX), mRNA.	-0.09	7.96	3.20E-10	8.13E-10	blue
TSPAN3	10099	tetraspanin 3 (TSPAN3), transcript variant 1, mRNA.	0.40	7.93	1.73E-64	6.08E-63	blue
TTYH3	80727	tweety homolog 3 (Drosophila) (TTYH3), mRNA.	-0.17	6.30	1.75E-17	6.63E-17	blue
TUBA1A	7846	tubulin, alpha 1a (TUBA1A), mRNA.	-0.06	7.62	9.79E-05	1.71E-04	blue
TUBB	203068	tubulin, beta (TUBB), mRNA.	0.32	8.81	1.22E-40	1.27E-39	blue
TUBB6	84617	tubulin, beta 6 (TUBB6), mRNA.	0.18	8.54	1.08E-13	3.32E-13	blue
TWSG1	57045	twisted gastrulation homolog 1 (Drosophila) (TWSG1), mRNA.	-0.07	6.33	3.81E-07	7.98E-07	blue
TXNDC5	81567	thioredoxin domain containing 5 (TXNDC5), transcript variant 1, mRNA.	0.04	6.98	1.91E-02	2.63E-02	blue
TYMP	1890	endothelial cell growth factor 1 (platelet-derived) (ECGF1), mRNA.	0.18	7.33	1.62E-18	6.44E-18	blue
UBB	7314	ubiquitin B (UBB), mRNA.	-0.04	9.84	6.81E-03	9.88E-03	blue
UBE2H	7328	ubiquitin-conjugating enzyme E2H (UBC8 homolog, yeast) (UBE2H), transcript variant 1, mRNA.	0.05	7.18	2.52E-03	3.81E-03	blue
UBE2L6	9246	ubiquitin-conjugating enzyme E2L 6 (UBE2L6), transcript variant 1, mRNA.	0.14	6.46	1.98E-13	6.04E-13	blue
UBE2O	63893	ubiquitin-conjugating enzyme E2O (UBE2O), mRNA.	0.05	7.43	2.55E-04	4.28E-04	blue

UBL7	84993	ubiquitin-like 7 (bone marrow stromal cell-derived) (UBL7), transcript variant 1, mRNA.	-0.67	7.44	9.30E-77	6.08E-75	blue
UBP1	7342	upstream binding protein 1 (LBP-1a) (UBP1), mRNA.	-0.02	8.71	1.36E-01	1.66E-01	blue
UBR4	23352	zinc finger, UBR1 type 1 (ZUBR1), mRNA.	0.16	8.36	5.95E-15	1.96E-14	blue
UBXN6	80700	UBX domain containing 1 (UBXD1), mRNA.	-0.30	10.17	9.76E-55	1.90E-53	blue
UFM1	51569	ubiquitin-fold modifier 1 (UFM1), mRNA.	0.06	7.70	2.53E-03	3.84E-03	blue
UPF2	26019	UPF2 regulator of nonsense transcripts homolog (yeast) (UPF2), transcript variant 2, mRNA.	0.08	7.40	3.94E-05	7.11E-05	blue
USF1	7391	upstream transcription factor 1 (USF1), transcript variant 2, mRNA.	0.02	6.84	7.12E-02	9.11E-02	blue
USP10	9100	ubiquitin specific peptidase 10 (USP10), mRNA.	-0.06	10.24	2.19E-08	4.98E-08	blue
UTP14A	10813	UTP14, U3 small nucleolar ribonucleoprotein, homolog A (yeast) (UTP14A), mRNA.	0.15	8.84	1.05E-19	4.44E-19	blue
VOPP1	81552	EGFR-coamplified and overexpressed protein (ECOP), mRNA.	0.04	8.63	6.88E-04	1.11E-03	blue
VPS25	84313	vacuolar protein sorting 25 homolog (S. cerevisiae) (VPS25), mRNA.	-0.03	8.16	1.53E-02	2.13E-02	blue
VWA9	81556	chromosome 15 open reading frame 44 (C15orf44), mRNA.	0.01	9.46	3.33E-01	3.78E-01	blue

WASH3P	375690	CXYorf1-related protein (FLJ00038), mRNA.	0.05	6.87	3.86E-05	6.98E-05	blue
WDR1	9948	WD repeat domain 1 (WDR1), transcript variant 1, mRNA.	0.26	7.30	2.66E-28	1.64E-27	blue
WDR74	54663	WD repeat domain 74 (WDR74), mRNA.	-0.02	5.51	2.18E-01	2.57E-01	blue
WDR75	84128	WD repeat domain 75 (WDR75), mRNA.	-0.01	9.07	5.07E-01	5.52E-01	blue
WNK1	65125	WNK lysine deficient protein kinase 1 (WNK1), mRNA.	-0.05	6.56	1.17E-03	1.83E-03	blue
WRAP73	49856	WD repeat domain 8 (WDR8), mRNA.	0.07	9.11	4.14E-11	1.11E-10	blue
WTAP	9589	Wilms tumor 1 associated protein (WTAP), transcript variant 1, mRNA.	0.05	10.01	7.36E-06	1.41E-05	blue
WTAP	646517	PREDICTED: similar to Wilms tumour 1-associating protein (LOC646517), mRNA.	-0.05	10.22	4.51E-04	7.41E-04	blue
XPO6	23214	exportin 6 (XPO6), mRNA.	-0.04	9.99	1.65E-03	2.55E-03	blue
XRCC6	2547	X-ray repair complementing defective repair in Chinese hamster cells 6 (Ku autoantigen, 70kDa) (XRCC6), mRNA.	0.06	6.92	1.03E-04	1.79E-04	blue
XRN2	22803	5'-3' exoribonuclease 2 (XRN2), mRNA.	-0.09	6.82	5.59E-07	1.16E-06	blue
YBX1	646531	PREDICTED: similar to nuclease sensitive element binding protein 1 (LOC646531), mRNA.	-0.24	6.77	8.69E-36	7.23E-35	blue

YME1L1	10730	YME1-like 1 (S. cerevisiae) (YME1L1), nuclear gene encoding mitochondrial protein, transcript variant 2, mRNA.	-0.07	7.60	7.06E-06	1.36E-05	blue
YWHAE	7531	tyrosine 3-monooxygenase/tryptophan 5-monooxygenase activation protein, epsilon polypeptide (YWHAE), mRNA.	0.01	7.41	6.77E-01	7.12E-01	blue
YWHAZ	7534	tyrosine 3-monooxygenase/tryptophan 5-monooxygenase activation protein, zeta polypeptide (YWHAZ), transcript variant 1, mRNA.	0.28	7.30	5.31E-36	4.45E-35	blue
ZAK	51776	sterile alpha motif and leucine zipper containing kinase AZK (ZAK), transcript variant 1, mRNA.	-0.12	8.34	5.05E-15	1.67E-14	blue
ZCCHC14	23174	zinc finger, CCHC domain containing 14 (ZCCHC14), mRNA.	-0.06	9.43	1.11E-04	1.93E-04	blue
ZFAND6	54469	zinc finger, AN1-type domain 6 (ZFAND6), mRNA.	0.03	6.20	8.09E-02	1.03E-01	blue
ZHX1	11244	zinc fingers and homeoboxes 1 (ZHX1), transcript variant 1, mRNA.	-0.09	8.70	2.16E-15	7.28E-15	blue
ZMAT3	64393	zinc finger, matrin type 3 (ZMAT3), transcript variant 2, mRNA.	-0.57	8.15	2.66E-73	1.41E-71	blue
ZNF217	7764	zinc finger protein 217 (ZNF217), mRNA.	-0.02	6.55	2.18E-01	2.58E-01	blue

ZSWIM8	23053	KIAA0913 (KIAA0913), mRNA.	-0.20	7.24	1.09E-35	9.06E-35	blue
ZWILCH	55055	Zwilch, kinetochore associated, homolog (Drosophila) (ZWILCH), transcript variant 1, mRNA.	0.01	10.66	6.44E-01	6.83E-01	blue
SEPT2	4735	septin 2 (SEPT2), transcript variant 3, mRNA.	-0.06	9.14	5.09E-05	9.09E-05	brown
SEPT9	10801	septin 9 (SEPT9), mRNA.	0.03	9.43	1.96E-02	2.70E-02	brown
AARS2	57505	alanyl-tRNA synthetase 2, mitochondrial (putative) (AARS2), mRNA.	-0.29	7.84	1.31E-29	8.37E-29	brown
ABCB7	22	ATP-binding cassette, sub-family B (MDR/TAP), member 7 (ABCB7), nuclear gene encoding mitochondrial protein, mRNA.	-0.11	9.25	2.52E-20	1.09E-19	brown
ABCF1	23	ATP-binding cassette, sub-family F (GCN20), member 1 (ABCF1), transcript variant 2, mRNA.	-0.06	8.95	8.81E-03	1.26E-02	brown
ABHD14B	84836	abhydrolase domain containing 14B (ABHD14B), mRNA.	0.01	11.26	4.80E-01	5.26E-01	brown
ABHD8	79575	abhydrolase domain containing 8 (ABHD8), mRNA.	-0.08	7.42	1.30E-10	3.38E-10	brown
ABL1	25	v-abl Abelson murine leukemia viral oncogene homolog 1 (ABL1), transcript variant b, mRNA.	-0.06	10.77	3.44E-10	8.73E-10	brown

ABR	29	active BCR-related gene (ABR), transcript variant 1, mRNA.	-0.18	11.70	1.70E-31	1.17E-30	brown
ACAD9	28976	acyl-Coenzyme A dehydrogenase family, member 9 (ACAD9), mRNA.	-0.04	9.37	5.10E-02	6.64E-02	brown
ACOT13	55856	thioesterase superfamily member 2 (THEM2), mRNA.	-0.01	10.81	3.69E-01	4.15E-01	brown
ACSL5	51703	acyl-CoA synthetase long-chain family member 5 (ACSL5), transcript variant 1, mRNA.	-0.09	9.43	1.27E-12	3.71E-12	brown
ACTR1A	10121	ARP1 actin-related protein 1 homolog A, centractin alpha (yeast) (ACTR1A), mRNA.	-0.02	10.04	2.62E-01	3.04E-01	brown
ADCY3	109	adenylate cyclase 3 (ADCY3), mRNA.	-0.14	9.26	1.76E-27	1.05E-26	brown
AES	166	amino-terminal enhancer of split (AES), transcript variant 2, mRNA.	0.12	7.15	1.90E-10	4.91E-10	brown
AGO2	27161	eukaryotic translation initiation factor 2C, 2 (EIF2C2), mRNA.	-0.13	6.77	2.84E-16	1.01E-15	brown
AIMP2	7965	JTV1 gene (JTV1), mRNA.	0.20	7.58	2.66E-31	1.82E-30	brown
ALDH4A1	8659	aldehyde dehydrogenase 4 family, member A1 (ALDH4A1), nuclear gene encoding mitochondrial protein, transcript variant P5CDhL, mRNA.	0.42	7.32	7.77E-61	2.23E-59	brown

ALKBH3	221120	alkB, alkylation repair homolog 3 (E. coli) (ALKBH3), mRNA.	0.11	6.36	1.97E-13	6.02E-13	brown
ALKBH5	54890	alkB, alkylation repair homolog 5 (E. coli) (ALKBH5), mRNA.	0.29	7.36	3.40E-58	8.33E-57	brown
AP1B1	162	adaptor-related protein complex 1, beta 1 subunit (AP1B1), transcript variant 1, mRNA.	0.03	8.95	1.10E-01	1.36E-01	brown
AP1M1	8907	adaptor-related protein complex 1, mu 1 subunit (AP1M1), mRNA.	0.05	9.35	1.51E-03	2.35E-03	brown
AP4B1	10717	adaptor-related protein complex 4, beta 1 subunit (AP4B1), mRNA.	0.24	8.00	6.27E-36	5.24E-35	brown
APBA3	9546	amyloid beta (A4) precursor protein-binding, family A, member 3 (X11-like 2) (APBA3), mRNA.	-0.26	9.80	4.72E-21	2.11E-20	brown
APBB3	10307	amyloid beta (A4) precursor protein-binding, family B, member 3 (APBB3), transcript variant 4, mRNA.	-0.06	11.86	5.84E-05	1.04E-04	brown
APEX2	27301	APEX nuclease (apurinic/apyrimidinic endonuclease) 2 (APEX2), nuclear gene encoding mitochondrial protein, mRNA.	0.66	8.71	1.43E-83	1.61E-81	brown
ARF4	378	ADP-ribosylation factor 4 (ARF4), mRNA.	-0.11	5.95	9.58E-09	2.23E-08	brown
ARFGAP2	84364	zinc finger protein 289, ID1 regulated (ZNF289), mRNA.	0.03	6.02	2.03E-02	2.78E-02	brown

ARHGAP17	55114	Rho GTPase activating protein 17 (ARHGAP17), transcript variant 1, mRNA.	0.09	7.74	6.26E-09	1.48E-08	brown
ARHGAP33	115703	sorting nexin 26 (SNX26), mRNA.	-0.24	5.83	4.99E-26	2.82E-25	brown
ARID3B	10620	AT rich interactive domain 3B (BRIGHT-like) (ARID3B), mRNA.	0.14	6.85	1.06E-15	3.65E-15	brown
ARIH2	10425	ariadne homolog 2 (Drosophila) (ARIH2), mRNA.	0.02	9.24	2.71E-01	3.13E-01	brown
ASCC2	84164	activating signal cointegrator 1 complex subunit 2 (ASCC2), mRNA.	-0.14	9.39	8.58E-25	4.62E-24	brown
ATG7	10533	ATG7 autophagy related 7 homolog (S. cerevisiae) (ATG7), mRNA.	-0.02	7.81	2.34E-01	2.74E-01	brown
ATP5H	10476	ATP synthase, H+ transporting, mitochondrial F0 complex, subunit d (ATP5H), nuclear gene encoding mitochondrial protein, transcript variant 1, mRNA.	0.36	6.86	8.81E-40	8.72E-39	brown
ATP6AP1	537	ATPase, H+ transporting, lysosomal accessory protein 1 (ATP6AP1), mRNA.	-0.09	8.91	1.67E-15	5.65E-15	brown
ATP6V1E1	529	ATPase, H+ transporting, lysosomal 31kDa, V1 subunit E1 (ATP6V1E1), transcript variant 1, mRNA.	-0.01	7.90	4.05E-01	4.50E-01	brown
ATXN2	6311	ataxin 2 (ATXN2), mRNA.	0.05	8.49	1.25E-04	2.15E-04	brown

BFAR	51283	bifunctional apoptosis regulator (BFAR), mRNA.	0.00	7.67	9.45E-01	9.53E-01	brown
BMS1	9790	BMS1-like, ribosome assembly protein (yeast) (BMS1L), mRNA.	0.03	6.65	3.28E-02	4.37E-02	brown
BRAT1	221927	chromosome 7 open reading frame 27 (C7orf27), mRNA.	0.01	8.98	5.94E-01	6.36E-01	brown
BRD2	6046	bromodomain containing 2 (BRD2), mRNA.	0.04	11.82	3.13E-03	4.70E-03	brown
BRD9	65980	bromodomain containing 9 (BRD9), transcript variant 1, mRNA.	0.89	6.83	2.03E-103	9.36E-101	brown
BRI3	25798	brain protein I3 (BRI3), mRNA.	0.03	8.57	1.06E-01	1.32E-01	brown
BUD31	8896	BUD31 homolog (S. cerevisiae) (BUD31), mRNA.	-0.10	7.20	3.34E-11	8.96E-11	brown
C14orf166	51637	chromosome 14 open reading frame 166 (C14orf166), mRNA.	0.14	8.57	8.82E-16	3.05E-15	brown
C14orf80	283643	chromosome 14 open reading frame 80 (C14orf80), mRNA.	0.63	7.66	4.84E-77	3.27E-75	brown
C16orf59	80178	chromosome 16 open reading frame 59 (C16orf59), mRNA.	-0.57	8.62	4.23E-60	1.17E-58	brown
C17orf70	80233	chromosome 17 open reading frame 70 (C17orf70), mRNA.	-0.11	7.16	7.40E-11	1.96E-10	brown
C1orf112	55732	chromosome 1 open reading frame 112 (C1orf112), mRNA.	0.06	6.82	5.12E-07	1.06E-06	brown
C1orf86	199990	chromosome 1 open reading frame 86 (C1orf86), mRNA.	0.07	12.20	3.82E-07	8.00E-07	brown

C1QBP	708	complement component 1, q subcomponent binding protein (C1QBP), nuclear gene encoding mitochondrial protein, mRNA.	0.37	8.02	3.16E-36	2.70E-35	brown
C2CD2	25966	chromosome 21 open reading frame 25 (C21orf25), transcript variant 1, mRNA.	-0.65	8.28	2.42E-83	2.67E-81	brown
C4orf27	54969	chromosome 4 open reading frame 27 (C4orf27), mRNA.	-0.17	8.41	4.38E-15	1.45E-14	brown
CARD11	84433	caspase recruitment domain family, member 11 (CARD11), mRNA.	0.07	8.64	3.13E-08	7.03E-08	brown
CASZ1	54897	castor zinc finger 1 (CASZ1), transcript variant 2, mRNA.	0.42	7.38	6.73E-66	2.48E-64	brown
CBFA2T3	863	core-binding factor, runt domain, alpha subunit 2; translocated to, 3 (CBFA2T3), transcript variant 1, mRNA.	0.03	7.26	5.10E-02	6.64E-02	brown
CCDC130	81576	coiled-coil domain containing 130 (CCDC130), mRNA.	0.04	7.70	2.79E-03	4.20E-03	brown
CCNI	10983	cyclin I (CCNI), mRNA.	0.16	6.57	8.06E-18	3.10E-17	brown
CD69	969	CD69 molecule (CD69), mRNA.	1.14	7.93	1.87E-109	1.36E-106	brown
CDC16	8881	cell division cycle 16 homolog (S. cerevisiae) (CDC16), transcript variant 1, mRNA.	0.08	6.03	4.80E-07	9.98E-07	brown
CDC20	991	cell division cycle 20 homolog (S. cerevisiae) (CDC20), mRNA.	0.54	5.74	3.60E-44	4.39E-43	brown

CDC37	11140	cell division cycle 37 homolog (S. cerevisiae) (CDC37), mRNA.	-0.21	7.07	8.16E-37	7.16E-36	brown
CDCA7	83879	cell division cycle associated 7 (CDCA7), transcript variant 2, mRNA.	-0.11	8.20	1.87E-05	3.48E-05	brown
CDK5RAP2	55755	CDK5 regulatory subunit associated protein 2 (CDK5RAP2), transcript variant 1, mRNA.	-0.54	6.44	4.33E-62	1.33E-60	brown
CECR5	27440	cat eye syndrome chromosome region, candidate 5 (CECR5), transcript variant 2, mRNA.	0.50	7.47	5.45E-69	2.34E-67	brown
CEP131	22994	5-azacytidine induced 1 (AZI1), transcript variant 2, mRNA.	-0.05	8.28	2.28E-04	3.84E-04	brown
CEP85	64793	coiled-coil domain containing 21 (CCDC21), mRNA.	0.00	6.24	8.77E-01	8.96E-01	brown
CHAF1B	8208	chromatin assembly factor 1, subunit B (p60) (CHAF1B), mRNA.	-0.20	6.40	2.03E-30	1.33E-29	brown
CHCHD10	400916	chromosome 22 open reading frame 16 (C22orf16), mRNA.	-0.08	9.89	4.74E-05	8.48E-05	brown
CHCHD4	131474	coiled-coil-helix-coiled-coil-helix domain containing 4 (CHCHD4), mRNA.	0.00	6.13	9.19E-01	9.32E-01	brown
CHP1	11261	calcium binding protein P22 (CHP), mRNA.	-0.02	9.56	8.82E-02	1.11E-01	brown
CKAP2L	150468	cytoskeleton associated protein 2-like (CKAP2L), mRNA.	-0.04	9.11	9.09E-04	1.45E-03	brown
CLCN7	1186	chloride channel 7 (CLCN7), mRNA.	0.11	6.35	1.30E-16	4.72E-16	brown

CLEC16A	23274	KIAA0350 (KIAA0350), mRNA.	-0.11	6.03	1.39E-04	2.38E-04	brown
CMC4	4515	mature T-cell proliferation 1 (MTCP1), nuclear gene encoding mitochondrial protein, transcript variant B1, mRNA.	0.00	7.36	9.31E-01	9.42E-01	brown
CNOT11	55571	chromosome 2 open reading frame 29 (C2orf29), mRNA.	-0.07	10.36	1.52E-11	4.17E-11	brown
COA5	493753	hypothetical protein MGC52110 (MGC52110), mRNA.	-0.22	6.02	1.07E-22	5.19E-22	brown
COMMD8	54951	COMM domain containing 8 (COMMD8), mRNA.	0.04	10.44	1.11E-02	1.57E-02	brown
COMT	1312	catechol-O-methyltransferase (COMT), transcript variant S-COMT, mRNA.	0.09	7.39	1.53E-06	3.09E-06	brown
COX7A2L	9167	cytochrome c oxidase subunit VIIa polypeptide 2 like (COX7A2L), nuclear gene encoding mitochondrial protein, mRNA.	-0.09	7.90	4.45E-07	9.28E-07	brown
CPSF1	29894	cleavage and polyadenylation specific factor 1, 160kDa (CPSF1), mRNA.	0.53	10.11	3.54E-71	1.66E-69	brown
CSNK2A1	1457	casein kinase 2, alpha 1 polypeptide (CSNK2A1), transcript variant 3, mRNA.	0.08	7.09	6.06E-04	9.82E-04	brown

CTDSP2	10106	CTD (carboxy-terminal domain, RNA polymerase II, polypeptide A) small phosphatase 2 (CTDSP2), mRNA.	-0.13	8.37	2.31E-17	8.70E-17	brown
CTNS	1497	cystinosis, nephropathic (CTNS), transcript variant 1, mRNA.	0.01	8.65	6.86E-01	7.21E-01	brown
CTSD	1509	cathepsin D (CTSD), mRNA.	-0.09	9.75	5.95E-10	1.49E-09	brown
CXXC1	30827	CXXC finger 1 (PHD domain) (CXXC1), mRNA.	0.16	5.92	2.29E-18	9.01E-18	brown
CYFIP2	26999	cytoplasmic FMR1 interacting protein 2 (CYFIP2), transcript variant 3, mRNA.	0.25	7.48	3.66E-27	2.17E-26	brown
DAP	1611	death-associated protein (DAP), mRNA.	-0.13	8.68	9.93E-10	2.45E-09	brown
DBNL	28988	drebrin-like (DBNL), transcript variant 2, mRNA.	0.10	6.76	3.08E-15	1.03E-14	brown
DDX27	55661	DEAD (Asp-Glu-Ala-Asp) box polypeptide 27 (DDX27), mRNA.	-0.05	6.51	4.08E-03	6.06E-03	brown
DDX46	9879	DEAD (Asp-Glu-Ala-Asp) box polypeptide 46 (DDX46), mRNA.	0.08	8.75	3.95E-07	8.27E-07	brown
DENND1A	57706	DENN/MADD domain containing 1A (DENND1A), transcript variant 2, mRNA.	0.07	5.89	4.66E-07	9.71E-07	brown
DMAP1	55929	DNA methyltransferase 1 associated protein 1 (DMAP1), transcript variant 1, mRNA.	-0.03	7.43	4.20E-02	5.53E-02	brown

DNAAF5	54919	HEAT repeat containing 2 (HEATR2), mRNA. XM_935824 XM_935825	-0.09	7.88	3.26E-12	9.28E-12	brown
DNAJC8	22826	DnaJ (Hsp40) homolog, subfamily C, member 8 (DNAJC8), mRNA.	-0.14	8.14	2.87E-15	9.62E-15	brown
DNMT1	1786	DNA (cytosine-5-)-methyltransferase 1 (DNMT1), mRNA.	-0.04	7.45	4.08E-03	6.05E-03	brown
DOCK2	1794	dedicator of cytokinesis 2 (DOCK2), mRNA.	-0.02	5.99	4.40E-01	4.86E-01	brown
DPYD	1806	dihydropyrimidine dehydrogenase (DPYD), mRNA.	0.02	9.68	3.36E-01	3.81E-01	brown
DRAP1	10589	DR1-associated protein 1 (negative cofactor 2 alpha) (DRAP1), mRNA.	0.08	9.03	4.56E-06	8.89E-06	brown
DUS2	54920	dihydrouridine synthase 2-like, SMM1 homolog (S. cerevisiae) (DUS2L), mRNA.	0.00	7.91	8.53E-01	8.75E-01	brown
DYNLT3	6990	dynein, light chain, Tctex-type 3 (DYNLT3), mRNA.	0.24	7.91	1.28E-23	6.47E-23	brown
E2F1	1869	E2F transcription factor 1 (E2F1), mRNA.	0.01	8.37	4.63E-01	5.09E-01	brown
E2F4	1874	E2F transcription factor 4, p107/p130-binding (E2F4), mRNA.	-0.05	6.72	5.96E-03	8.70E-03	brown
E4F1	1877	E4F transcription factor 1 (E4F1), mRNA.	-0.08	9.01	1.44E-08	3.32E-08	brown
ECHDC2	55268	enoyl Coenzyme A hydratase domain containing 2 (ECHDC2), mRNA.	-0.25	9.80	4.54E-33	3.34E-32	brown
EDC4	23644	enhancer of mRNA decapping 4 (EDC4), mRNA.	-0.23	9.39	2.40E-41	2.55E-40	brown

EI24	9538	etoposide induced 2.4 mRNA (EI24), transcript variant 2, mRNA.	0.28	9.31	7.69E-36	6.41E-35	brown
EIF2AK1	27102	eukaryotic translation initiation factor 2-alpha kinase 1 (EIF2AK1), mRNA.	-0.12	8.01	2.29E-14	7.32E-14	brown
EIF2B2	8892	eukaryotic translation initiation factor 2B, subunit 2 beta, 39kDa (EIF2B2), mRNA.	0.12	8.75	2.78E-10	7.10E-10	brown
EIF2B5	8893	eukaryotic translation initiation factor 2B, subunit 5 epsilon, 82kDa (EIF2B5), mRNA.	0.20	6.53	7.05E-20	2.99E-19	brown
EIF4G3	8672	eukaryotic translation initiation factor 4 gamma, 3 (EIF4G3), mRNA.	0.03	5.51	5.20E-02	6.76E-02	brown
EML3	256364	echinoderm microtubule associated protein like 3 (EML3), mRNA.	-0.04	7.22	3.23E-02	4.30E-02	brown
ENO3	2027	enolase 3 (beta, muscle) (ENO3), transcript variant 2, mRNA.	0.27	6.55	5.67E-26	3.19E-25	brown
EVI5L	115704	ecotropic viral integration site 5-like (EVI5L), mRNA.	-0.05	9.95	3.17E-04	5.28E-04	brown
EXOSC10	5394	exosome component 10 (EXOSC10), transcript variant 1, mRNA.	-0.28	7.23	3.47E-34	2.67E-33	brown
FAF2	23197	UBX domain containing 8 (UBXD8), mRNA.	-0.30	7.17	1.75E-34	1.37E-33	brown
FAM120A	23196	family with sequence similarity 120A (FAM120A), mRNA.	0.07	7.13	2.09E-08	4.75E-08	brown

FAM136A	84908	hypothetical protein FLJ14668 (FLJ14668), mRNA.	0.54	8.11	9.06E-66	3.29E-64	brown
FAM53C	51307	family with sequence similarity 53, member C (FAM53C), mRNA.	0.05	11.21	1.31E-04	2.25E-04	brown
FAM96A	84191	family with sequence similarity 96, member A (FAM96A), transcript variant 1, mRNA.	-0.04	5.93	6.36E-03	9.26E-03	brown
FAN1	54893	myotubularin related protein 10 (MTMR10), mRNA.	0.03	11.41	8.04E-02	1.02E-01	brown
FBXO18	84893	F-box protein, helicase, 18 (FBXO18), transcript variant 1, mRNA.	0.07	7.24	1.29E-06	2.62E-06	brown
FDXR	2232	ferredoxin reductase (FDXR), nuclear gene encoding mitochondrial protein, transcript variant 1, mRNA.	-0.01	9.42	4.35E-01	4.82E-01	brown
FEM1A	55527	fem-1 homolog a (C. elegans) (FEM1A), mRNA.	0.10	7.56	1.25E-08	2.89E-08	brown
FGD3	89846	FYVE, RhoGEF and PH domain containing 3 (FGD3), mRNA.	-0.03	9.27	3.23E-02	4.31E-02	brown
FKBP1A	2280	FK506 binding protein 1A, 12kDa (FKBP1A), transcript variant 12B, mRNA.	-0.23	5.62	1.70E-22	8.13E-22	brown
FKBP3	2287	FK506 binding protein 3, 25kDa (FKBP3), mRNA.	0.49	8.18	5.59E-69	2.38E-67	brown
FKBP4	2288	FK506 binding protein 4, 59kDa (FKBP4), mRNA.	0.02	10.78	1.12E-01	1.39E-01	brown

FLAD1	80308	FAD1 flavin adenine dinucleotide synthetase homolog (S. cerevisiae) (FLAD1), transcript variant 2, mRNA.	-0.34	6.13	1.73E-43	2.05E-42	brown
FOXJ2	55810	forkhead box J2 (FOXJ2), mRNA.	-0.07	5.99	2.62E-06	5.18E-06	brown
FRMD8	83786	FKSG44 gene (FKSG44), mRNA.	0.03	8.78	1.00E-01	1.26E-01	brown
FUK	197258	fucokinase (FUK), mRNA.	-0.10	11.07	5.78E-13	1.72E-12	brown
GAK	2580	cyclin G associated kinase (GAK), mRNA. XM_943600 XM_943603	0.00	6.75	9.15E-01	9.29E-01	brown
GATAD2A	54815	GATA zinc finger domain containing 2A (GATAD2A), mRNA.	-0.22	7.70	6.77E-31	4.52E-30	brown
GATAD2B	57459	GATA zinc finger domain containing 2B (GATAD2B), mRNA.	-0.04	8.13	1.28E-02	1.80E-02	brown
GATB	5188	PET112-like (yeast) (PET112L), mRNA.	0.04	7.81	1.15E-03	1.81E-03	brown
GCN1L1	10985	GCN1 general control of amino-acid synthesis 1-like 1 (yeast) (GCN1L1), mRNA.	0.08	7.27	6.68E-06	1.28E-05	brown
GEMIN2	8487	survival of motor neuron protein interacting protein 1 (SIP1), transcript variant beta, mRNA.	-0.08	11.32	4.47E-06	8.72E-06	brown
GEMIN4	50628	gem (nuclear organelle) associated protein 4 (GEMIN4), mRNA.	0.04	7.01	2.45E-02	3.32E-02	brown

GGH	8836	gamma-glutamyl hydrolase (conjugase, folylpolygammaglutamyl hydrolase) (GGH), mRNA.	-0.14	9.87	1.09E-23	5.54E-23	brown
GLRX	2745	glutaredoxin (thioltransferase) (GLRX), mRNA.	0.06	9.24	1.94E-05	3.60E-05	brown
GNG5	2787	guanine nucleotide binding protein (G protein), gamma 5 (GNG5), mRNA.	-0.09	6.73	7.28E-14	2.27E-13	brown
GNL1	2794	guanine nucleotide binding protein-like 1 (GNL1), mRNA.	-0.03	6.52	2.09E-01	2.47E-01	brown
GPHN	10243	gephyrin (GPHN), transcript variant 2, mRNA.	0.23	6.96	9.40E-27	5.46E-26	brown
GPI	2821	glucose phosphate isomerase (GPI), mRNA.	0.49	7.97	4.90E-72	2.41E-70	brown
GPR137	56834	G protein-coupled receptor 137 (GPR137), mRNA.	-0.01	6.47	5.84E-01	6.27E-01	brown
GPS1	2873	G protein pathway suppressor 1 (GPS1), transcript variant 1, mRNA.	0.01	9.84	3.43E-01	3.88E-01	brown
GPSM3	63940	G-protein signalling modulator 3 (AGS3-like, C. elegans) (GPSM3), mRNA.	0.46	8.56	2.92E-45	3.78E-44	brown
GRAMD1A	57655	GRAM domain containing 1A (GRAMD1A), mRNA.	0.06	8.32	1.85E-05	3.46E-05	brown
GTPBP6	8225	GTP binding protein 6 (putative) (GTPBP6), mRNA.	-0.02	7.88	5.44E-02	7.05E-02	brown
GUCD1	83606	chromosome 22 open reading frame 13 (C22orf13), mRNA.	-0.05	7.02	9.35E-03	1.34E-02	brown

HADHA	3030	hydroxyacyl-Coenzyme A dehydrogenase/3-ketoacyl-Coenzyme A thiolase/enoyl-Coenzyme A hydratase (trifunctional protein), alpha subunit (HADHA), nuclear gene encoding mitochondrial protein, mRNA.	0.07	11.49	1.48E-10	3.83E-10	brown
HARS	3035	histidyl-tRNA synthetase (HARS), mRNA.	-0.04	7.66	8.20E-03	1.18E-02	brown
HCFC1	3054	host cell factor C1 (VP16-accessory protein) (HCFC1), mRNA.	0.06	11.70	3.98E-06	7.79E-06	brown
HDAC1	3065	histone deacetylase 1 (HDAC1), mRNA.	-0.08	9.52	4.93E-07	1.02E-06	brown
HGS	9146	hepatocyte growth factor-regulated tyrosine kinase substrate (HGS), mRNA.	-0.10	7.86	1.38E-09	3.37E-09	brown
HIRIP3	8479	HIRA interacting protein 3 (HIRIP3), mRNA.	-0.24	7.49	1.11E-26	6.44E-26	brown
HMHA1	23526	histocompatibility (minor) HA-1 (HMHA1), mRNA.	0.00	5.98	8.49E-01	8.71E-01	brown
HNRNPDL	9987	heterogeneous nuclear ribonucleoprotein D-like (HNRPDL), transcript variant 3, transcribed RNA.	-0.48	10.81	1.15E-77	8.31E-76	brown
HSBP1	3281	heat shock factor binding protein 1 (HSBP1), mRNA.	0.29	7.21	2.20E-38	2.08E-37	brown
IGBP1	3476	immunoglobulin (CD79A) binding protein 1 (IGBP1), mRNA.	-0.10	7.19	3.16E-09	7.60E-09	brown

IGF2R	3482	insulin-like growth factor 2 receptor (IGF2R), mRNA.	-0.01	6.22	6.79E-01	7.14E-01	brown
INO80E	283899	coiled-coil domain containing 95 (CCDC95), mRNA.	0.10	8.09	1.05E-07	2.30E-07	brown
INPPL1	3636	inositol polyphosphate phosphatase-like 1 (INPPL1), mRNA.	-0.08	9.20	7.93E-07	1.63E-06	brown
INTS6	26512	integrator complex subunit 6 (INTS6), transcript variant 1, mRNA.	0.62	8.62	2.68E-64	9.33E-63	brown
INTS9	55756	integrator complex subunit 9 (RC74), mRNA.	0.04	6.45	6.50E-02	8.36E-02	brown
IPO11	51194	importin 11 (IPO11), mRNA.	-0.17	9.65	1.02E-21	4.72E-21	brown
ITGB1BP1	9270	integrin beta 1 binding protein 1 (ITGB1BP1), transcript variant 2, mRNA.	-0.23	7.38	2.31E-41	2.46E-40	brown
ITPR3	3710	inositol 1,4,5-triphosphate receptor, type 3 (ITPR3), mRNA.	0.00	5.86	9.82E-01	9.85E-01	brown
JADE1	79960	PHD finger protein 17 (PHF17), transcript variant S, mRNA.	-0.07	8.16	1.21E-05	2.29E-05	brown
JMJD8	339123	hypothetical LOC339123 (LOC339123), mRNA.	-0.18	7.40	4.05E-20	1.75E-19	brown
KANSL2	54934	chromosome 12 open reading frame 41 (C12orf41), mRNA.	-0.08	9.91	2.73E-10	7.00E-10	brown
KAT2A	2648	GCN5 general control of amino-acid synthesis 5-like 2 (yeast) (GCN5L2), mRNA.	0.02	8.66	1.14E-01	1.42E-01	brown

KDELR2	11014	KDEL (Lys-Asp-Glu-Leu) endoplasmic reticulum protein retention receptor 2 (KDELR2), mRNA.	-0.06	9.18	9.82E-08	2.14E-07	brown
KDM5A	5927	Jumonji, AT rich interactive domain 1A (RBBP2-like) (JARID1A), mRNA.	0.02	7.91	1.72E-01	2.07E-01	brown
KHSRP	8570	KH-type splicing regulatory protein (FUSE binding protein 2) (KHSRP), mRNA.	-0.16	8.96	1.24E-30	8.18E-30	brown
KIAA0195	9772	KIAA0195 (KIAA0195), mRNA.	0.47	6.23	3.76E-36	3.17E-35	brown
KIAA0391	9692	KIAA0391 (KIAA0391), mRNA.	0.15	6.23	5.09E-14	1.60E-13	brown
KIAA1279	26128	KIAA1279 (KIAA1279), mRNA.	0.05	7.69	1.95E-04	3.30E-04	brown
KLHL22	84861	kelch-like 22 (Drosophila) (KLHL22), mRNA.	-0.09	10.44	1.52E-12	4.41E-12	brown
KRBA1	84626	KRAB-A domain containing 1 (KRBA1), mRNA.	0.06	6.33	4.37E-05	7.84E-05	brown
KXD1	79036	chromosome 19 open reading frame 50 (C19orf50), mRNA.	-0.02	9.15	2.33E-01	2.73E-01	brown
LDHA	3939	lactate dehydrogenase A (LDHA), mRNA.	0.01	7.56	6.65E-01	7.01E-01	brown
LIPA	3988	lipase A, lysosomal acid, cholesterol esterase (Wolman disease) (LIPA), mRNA.	-0.07	8.38	5.01E-09	1.19E-08	brown
LITAF	9516	lipopolysaccharide-induced TNF factor (LITAF), mRNA.	0.12	7.75	4.21E-10	1.06E-09	brown

LRR1	122769	peptidylprolyl isomerase (cyclophilin)-like 5 (PPIL5), transcript variant 1, mRNA.	-0.14	6.58	9.51E-10	2.35E-09	brown
LRRC14	9684	leucine rich repeat containing 14 (LRRC14), mRNA.	0.18	7.14	1.02E-25	5.72E-25	brown
LRRC45	201255	leucine rich repeat containing 45 (LRRC45), mRNA.	0.05	7.50	1.43E-03	2.22E-03	brown
LRSAM1	90678	leucine rich repeat and sterile alpha motif containing 1 (LRSAM1), transcript variant 3, mRNA.	0.00	7.00	8.25E-01	8.49E-01	brown
LRWD1	222229	hypothetical protein DKFZp434K1815 (DKFZp434K1815), mRNA.	0.18	8.06	3.09E-23	1.54E-22	brown
LSM5	23658	LSM5 homolog, U6 small nuclear RNA associated (S. cerevisiae) (LSM5), mRNA.	0.07	8.28	2.37E-05	4.36E-05	brown
LTB4R	1241	leukotriene B4 receptor (LTB4R), mRNA.	0.08	9.57	4.61E-08	1.03E-07	brown
MAD2L1	4085	MAD2 mitotic arrest deficient-like 1 (yeast) (MAD2L1), mRNA.	-0.02	8.52	6.80E-02	8.73E-02	brown
MAP4K2	5871	mitogen-activated protein kinase kinase kinase kinase 2 (MAP4K2), mRNA.	0.09	8.76	6.22E-08	1.37E-07	brown
MBP	4155	myelin basic protein (MBP), transcript variant 8, mRNA.	-0.29	9.91	1.22E-31	8.49E-31	brown

MCM7	4176	MCM7 minichromosome maintenance deficient 7 (S. cerevisiae) (MCM7), transcript variant 1, mRNA.	-0.41	6.81	2.65E-44	3.27E-43	brown
MCOLN2	255231	mucolipin 2 (MCOLN2), mRNA.	-0.28	8.38	5.57E-50	8.70E-49	brown
MCUR1	63933	coiled-coil domain containing 90A (CCDC90A), mRNA.	-0.05	7.06	6.26E-03	9.12E-03	brown
MED22	6837	surfeit 5 (SURF5), transcript variant a, mRNA.	0.13	9.54	1.11E-15	3.83E-15	brown
MEF2D	4209	MADS box transcription enhancer factor 2, polypeptide D (myocyte enhancer factor 2D) (MEF2D), mRNA.	-0.07	5.93	7.77E-03	1.12E-02	brown
MEPCE	56257	bin3, bicoid-interacting 3, homolog (Drosophila) (BCDIN3), mRNA.	-0.06	6.28	4.34E-05	7.80E-05	brown
METTL1	4234	methyltransferase like 1 (METTL1), transcript variant 1, mRNA.	0.00	8.48	9.68E-01	9.73E-01	brown
METTL5	29081	methyltransferase like 5 (METTL5), mRNA.	0.13	10.94	4.02E-18	1.57E-17	brown
MFSD3	113655	major facilitator superfamily domain containing 3 (MFSD3), mRNA.	-0.09	7.57	7.32E-09	1.72E-08	brown
MGME1	92667	chromosome 20 open reading frame 72 (C20orf72), mRNA.	0.00	5.52	7.91E-01	8.18E-01	brown

MIEF1	54471	Smith-Magenis syndrome chromosome region, candidate 7-like (SMCR7L), mRNA.	0.02	10.06	2.76E-01	3.19E-01	brown
MMACHC	25974	methylmalonic aciduria (cobalamin deficiency) cblC type, with homocystinuria (MMACHC), mRNA.	-0.01	8.94	5.39E-01	5.84E-01	brown
MOAP1	64112	modulator of apoptosis 1 (MOAP1), mRNA.	-0.01	6.08	6.10E-01	6.51E-01	brown
MOB3A	126308	MOB1, Mps One Binder kinase activator-like 2A (yeast) (MOBKL2A), mRNA.	0.08	6.32	2.35E-08	5.32E-08	brown
MOB3C	148932	MOB1, Mps One Binder kinase activator-like 2C (yeast) (MOBKL2C), transcript variant 1, mRNA.	0.01	11.60	4.00E-01	4.46E-01	brown
MORC2	22880	MORC family CW-type zinc finger 2 (MORC2), mRNA.	-0.28	7.73	5.11E-48	7.64E-47	brown
MRPL3	11222	mitochondrial ribosomal protein L3 (MRPL3), nuclear gene encoding mitochondrial protein, mRNA.	0.12	10.10	2.61E-10	6.69E-10	brown
MRPL35	51318	mitochondrial ribosomal protein L35 (MRPL35), nuclear gene encoding mitochondrial protein, transcript variant 1, mRNA.	0.00	6.75	8.88E-01	9.06E-01	brown

MRPL37	51253	mitochondrial ribosomal protein L37 (MRPL37), nuclear gene encoding mitochondrial protein, mRNA.	-0.11	6.85	8.05E-11	2.12E-10	brown
MRPS10	55173	mitochondrial ribosomal protein S10 (MRPS10), nuclear gene encoding mitochondrial protein, mRNA.	-0.28	9.79	1.42E-43	1.70E-42	brown
MTFR1	9650	mitochondrial fission regulator 1 (MTFR1), mRNA.	-0.03	8.13	4.39E-02	5.78E-02	brown
MYH9	4627	myosin, heavy chain 9, non-muscle (MYH9), mRNA.	0.10	8.65	4.24E-08	9.45E-08	brown
NA	730316	PREDICTED: similar to Nuclear envelope pore membrane protein POM 121 (Pore membrane protein of 121 kDa) (P145) (LOC730316), mRNA.	-0.24	8.99	4.59E-46	6.23E-45	brown
NA	84992	phosphatidylinositol glycan anchor biosynthesis, class Y (PIGY), transcript variant 2, mRNA.	0.27	7.04	4.90E-32	3.48E-31	brown
NA	79716	aminopeptidase-like 1 (NPEPL1), mRNA.	0.11	8.82	8.55E-08	1.87E-07	brown
NA	729843	PREDICTED: similar to WW domain binding protein 1 (LOC729843), mRNA.	0.06	5.98	2.88E-04	4.82E-04	brown
NA	84129	acyl-Coenzyme A dehydrogenase family, member 11 (ACAD11), mRNA.	-0.04	8.22	4.91E-03	7.22E-03	brown

NA	10588	5,10-methenyltetrahydrofolate synthetase (5-formyltetrahydrofolate cyclo-ligase) (MTHFS), mRNA.	-0.04	8.89	3.07E-02	4.11E-02	brown
NA	26580	Bernardinelli-Seip congenital lipodystrophy 2 (seipin) (BSCL2), mRNA.	0.03	5.80	4.59E-02	6.02E-02	brown
NAT10	55226	N-acetyltransferase 10 (NAT10), mRNA.	0.11	6.33	1.57E-12	4.55E-12	brown
NCLN	56926	nicalin homolog (zebrafish) (NCLN), mRNA.	-0.10	9.32	5.26E-19	2.15E-18	brown
NCSTN	23385	nicastrin (NCSTN), mRNA.	-0.06	8.44	9.35E-06	1.78E-05	brown
NDE1	54820	nudE nuclear distribution gene E homolog 1 (A. nidulans) (NDE1), mRNA.	0.05	5.85	4.49E-05	8.05E-05	brown
NDNL2	56160	necdin-like 2 (NDNL2), mRNA.	-0.05	8.72	3.25E-05	5.91E-05	brown
NDUFC1	4717	NADH dehydrogenase (ubiquinone) 1, subcomplex unknown, 1, 6kDa (NDUFC1), mRNA.	0.08	7.59	5.13E-06	9.96E-06	brown
NDUFV2	4729	NADH dehydrogenase (ubiquinone) flavoprotein 2, 24kDa (NDUFV2), mRNA.	0.06	6.59	1.31E-02	1.84E-02	brown
NFIC	4782	nuclear factor I/C (CCAAT-binding transcription factor) (NFIC), transcript variant 2, mRNA.	-0.21	8.06	2.50E-29	1.58E-28	brown

NFKB1	4790	nuclear factor of kappa light polypeptide gene enhancer in B-cells 1 (p105) (NFKB1), mRNA.	-0.11	5.96	7.28E-10	1.81E-09	brown
NFU1	27247	NFU1 iron-sulfur cluster scaffold homolog (S. cerevisiae) (NFU1), transcript variant 4, mRNA.	0.04	8.08	9.55E-03	1.36E-02	brown
NIPSNAP1	8508	nipsnap homolog 1 (C. elegans) (NIPSNAP1), mRNA.	-0.01	10.16	6.25E-01	6.65E-01	brown
NMT1	4836	N-myristoyltransferase 1 (NMT1), mRNA.	0.04	8.78	7.04E-03	1.02E-02	brown
NOL12	79159	hypothetical protein MGC3731 (MGC3731), mRNA.	-0.05	8.72	3.78E-03	5.62E-03	brown
NOP2	4839	nucleolar protein 1, 120kDa (NOL1), transcript variant 2, mRNA.	-0.06	8.94	5.11E-08	1.14E-07	brown
NOTCH1	4851	Notch homolog 1, translocation-associated (Drosophila) (NOTCH1), mRNA.	0.09	6.64	1.19E-06	2.42E-06	brown
NRBF2	29982	nuclear receptor binding factor 2 (NRBF2), mRNA.	-0.08	10.07	8.51E-09	1.99E-08	brown
NSA2	10412	TGF beta-inducible nuclear protein 1 (TINP1), mRNA.	0.14	7.19	8.37E-17	3.06E-16	brown
NSMF	26012	nasal embryonic LHRH factor (NELF), mRNA.	-0.31	8.26	2.30E-41	2.45E-40	brown
NTPCR	84284	chromosome 1 open reading frame 57 (C1orf57), mRNA.	-0.47	6.47	6.68E-58	1.59E-56	brown

NUP62	23636	nucleoporin 62kDa (NUP62), transcript variant 4, mRNA.	0.06	8.08	3.38E-05	6.15E-05	brown
NVL	4931	nuclear VCP-like (NVL), transcript variant 2, mRNA.	-0.13	6.40	1.22E-10	3.18E-10	brown
OIP5	11339	Opa interacting protein 5 (OIP5), mRNA.	-0.04	9.20	3.53E-03	5.28E-03	brown
OSBP	5007	oxysterol binding protein (OSBP), mRNA.	-0.13	7.80	2.28E-12	6.54E-12	brown
OTUD5	55593	OTU domain containing 5 (OTUD5), mRNA.	0.06	6.97	2.90E-07	6.12E-07	brown
PACSIN2	11252	protein kinase C and casein kinase substrate in neurons 2 (PACSIN2), mRNA.	-0.11	6.64	9.72E-14	3.00E-13	brown
PAF1	54623	Paf1, RNA polymerase II associated factor, homolog (S. cerevisiae) (PAF1), mRNA.	-0.18	8.18	3.53E-24	1.84E-23	brown
PAPD5	64282	PAP associated domain containing 5 (PAPD5), transcript variant 1, mRNA.	-0.10	7.65	3.21E-11	8.62E-11	brown
PARP6	56965	poly (ADP-ribose) polymerase family, member 6 (PARP6), mRNA.	-0.09	7.45	4.05E-13	1.21E-12	brown
PBK	55872	PDZ binding kinase (PBK), mRNA.	-0.17	7.36	6.54E-31	4.37E-30	brown
PBRM1	55193	polybromo 1 (PB1), transcript variant 2, mRNA.	0.54	9.60	1.09E-52	1.89E-51	brown
PCK2	5106	phosphoenolpyruvate carboxykinase 2 (mitochondrial) (PCK2), nuclear gene encoding mitochondrial protein, transcript variant 1, mRNA.	-0.15	9.61	1.30E-32	9.45E-32	brown

PCNX	22990	pecanex homolog (Drosophila) (PCNX), mRNA.	-0.05	9.41	4.67E-05	8.37E-05	brown
PDCL3	79031	phosducin-like 3 (PDCL3), mRNA.	-0.10	7.29	1.35E-13	4.16E-13	brown
PDDC1	347862	Parkinson disease 7 domain containing 1 (PDDC1), mRNA.	0.12	8.70	2.17E-11	5.92E-11	brown
PDXK	8566	pyridoxal (pyridoxine, vitamin B6) kinase (PDXK), mRNA.	-0.21	8.24	7.97E-32	5.60E-31	brown
PDXP	57026	pyridoxal (pyridoxine, vitamin B6) phosphatase (PDXP), mRNA.	-0.06	9.91	1.38E-05	2.60E-05	brown
PEBP1	5037	phosphatidylethanolamine binding protein 1 (PEBP1), mRNA.	0.16	6.26	4.83E-20	2.07E-19	brown
PES1	23481	pescadillo homolog 1, containing BRCT domain (zebrafish) (PES1), mRNA.	-0.38	8.25	8.30E-61	2.37E-59	brown
PEX2	5828	peroxisomal membrane protein 3, 35kDa (Zellweger syndrome) (PXMP3), transcript variant 2, mRNA.	0.07	5.16	1.04E-04	1.81E-04	brown
PEX26	55670	peroxisome biogenesis factor 26 (PEX26), mRNA.	-0.06	7.85	2.52E-05	4.63E-05	brown
PHB	5245	prohibitin (PHB), mRNA.	0.19	6.60	1.45E-17	5.52E-17	brown
PHRF1	57661	CTD-binding SR-like protein rA9 (KIAA1542), mRNA.	-0.10	6.96	7.53E-10	1.87E-09	brown
PI4KA	5297	phosphatidylinositol 4-kinase, catalytic, alpha polypeptide (PIK4CA), transcript variant 2, mRNA.	0.48	10.00	6.70E-66	2.48E-64	brown

PIAS3	10401	protein inhibitor of activated STAT, 3 (PIAS3), mRNA.	-0.19	7.78	7.17E-29	4.47E-28	brown
PIAS4	51588	protein inhibitor of activated STAT, 4 (PIAS4), mRNA.	0.01	6.90	5.37E-01	5.82E-01	brown
PIDD1	55367	leucine-rich repeats and death domain containing (LRDD), transcript variant 2, mRNA.	0.05	7.37	3.73E-04	6.18E-04	brown
PIGF	5281	phosphatidylinositol glycan, class F (PIGF), transcript variant 2, mRNA.	0.00	7.70	7.38E-01	7.69E-01	brown
PIGP	51227	phosphatidylinositol glycan anchor biosynthesis, class P (PIGP), transcript variant 2, mRNA.	-0.03	10.17	9.64E-03	1.37E-02	brown
PIGQ	9091	phosphatidylinositol glycan, class Q (PIGQ), transcript variant 2, mRNA.	-0.14	9.05	4.27E-14	1.35E-13	brown
PIK3R2	5296	phosphoinositide-3-kinase, regulatory subunit 2 (p85 beta) (PIK3R2), mRNA.	-0.07	7.54	2.97E-07	6.28E-07	brown
PLOD1	5351	procollagen-lysine 1, 2-oxoglutarate 5-dioxygenase 1 (PLOD1), mRNA.	0.14	8.58	5.84E-22	2.72E-21	brown
PNMAL1	55228	hypothetical protein FLJ10781 (FLJ10781), mRNA.	-0.01	10.02	3.03E-01	3.47E-01	brown
POLA2	23649	polymerase (DNA directed), alpha 2 (70kD subunit) (POLA2), mRNA.	-0.10	7.93	9.00E-16	3.11E-15	brown
POLD2	5425	polymerase (DNA directed), delta 2, regulatory subunit 50kDa (POLD2), mRNA.	-0.06	5.82	2.31E-04	3.89E-04	brown
POLE	5426	polymerase (DNA directed), epsilon (POLE), mRNA.	-0.05	9.57	4.91E-05	8.77E-05	brown

POLG	5428	polymerase (DNA directed), gamma (POLG), mRNA.	0.00	5.61	7.47E-01	7.78E-01	brown
POLR2A	5430	polymerase (RNA) II (DNA directed) polypeptide A, 220kDa (POLR2A), mRNA.	0.05	7.87	2.11E-05	3.89E-05	brown
POU2AF1	5450	POU domain, class 2, associating factor 1 (POU2AF1), mRNA.	0.00	7.89	8.24E-01	8.48E-01	brown
PPDPF	79144	chromosome 20 open reading frame 149 (C20orf149), mRNA.	0.08	8.59	1.59E-08	3.65E-08	brown
PPP2R5D	5528	protein phosphatase 2, regulatory subunit B', delta isoform (PPP2R5D), transcript variant 2, mRNA.	0.02	10.46	9.95E-02	1.25E-01	brown
PPRC1	23082	peroxisome proliferator-activated receptor gamma, coactivator-related 1 (PPRC1), mRNA.	-0.02	5.42	3.54E-01	4.00E-01	brown
PRDM4	11108	PR domain containing 4 (PRDM4), mRNA.	-0.14	8.04	2.74E-18	1.07E-17	brown
PRKD2	25865	protein kinase D2 (PRKD2), transcript variant 4, mRNA.	-0.46	10.10	1.96E-58	4.93E-57	brown
PRKDC	5591	protein kinase, DNA-activated, catalytic polypeptide (PRKDC), transcript variant 2, mRNA.	0.03	9.26	2.11E-02	2.89E-02	brown
PRMT7	54496	protein arginine methyltransferase 7 (PRMT7), mRNA.	0.12	9.11	1.25E-14	4.05E-14	brown

PRPF19	27339	PRP19/PSO4 pre-mRNA processing factor 19 homolog (S. cerevisiae) (PRPF19), mRNA.	0.05	8.81	1.80E-05	3.35E-05	brown
PRR14	78994	proline rich 14 (PRR14), mRNA.	0.07	9.16	1.65E-04	2.81E-04	brown
PSAP	5660	prosaposin (variant Gaucher disease and variant metachromatic leukodystrophy) (PSAP), transcript variant 3, mRNA.	0.05	8.88	4.96E-04	8.12E-04	brown
PSMC3	5702	proteasome (prosome, macropain) 26S subunit, ATPase, 3 (PSMC3), mRNA.	0.01	8.12	7.40E-01	7.71E-01	brown
PSMD8	5714	proteasome (prosome, macropain) 26S subunit, non-ATPase, 8 (PSMD8), mRNA.	0.08	6.68	4.90E-06	9.53E-06	brown
PUF60	22827	fuse-binding protein-interacting repressor (SIAHBP1), transcript variant 2, mRNA.	-0.03	6.72	8.84E-02	1.12E-01	brown
PUS1	80324	pseudouridylate synthase 1 (PUS1), transcript variant 1, mRNA.	-0.40	6.51	2.74E-45	3.55E-44	brown
RAB11FIP3	9727	RAB11 family interacting protein 3 (class II) (RAB11FIP3), mRNA.	0.13	8.92	8.37E-12	2.32E-11	brown
RAB35	11021	RAB35, member RAS oncogene family (RAB35), mRNA.	0.00	7.19	8.78E-01	8.97E-01	brown
RABEPK	10244	Rab9 effector protein with kelch motifs (RABEPK), mRNA.	0.05	9.31	8.27E-05	1.45E-04	brown

RABGAP1	23637	RAB GTPase activating protein 1 (RABGAP1), mRNA.	0.10	8.16	1.07E-07	2.34E-07	brown
RABGGTA	5875	Rab geranylgeranyltransferase, alpha subunit (RABGGTA), transcript variant 1, mRNA.	0.03	8.34	5.89E-02	7.61E-02	brown
RALGDS	5900	ral guanine nucleotide dissociation stimulator (RALGDS), transcript variant 2, mRNA.	-0.12	7.22	1.08E-14	3.52E-14	brown
RANGAP1	5905	Ran GTPase activating protein 1 (RANGAP1), mRNA.	0.40	7.12	5.70E-46	7.69E-45	brown
RAPGEF1	2889	Rap guanine nucleotide exchange factor (GEF) 1 (RAPGEF1), transcript variant 2, mRNA.	0.04	8.06	2.09E-02	2.85E-02	brown
RBM14	10432	RNA binding motif protein 14 (RBM14), mRNA.	-0.10	9.37	1.81E-14	5.83E-14	brown
RBM22	55696	RNA binding motif protein 22 (RBM22), mRNA.	0.05	7.38	4.16E-03	6.17E-03	brown
RCC2	55920	regulator of chromosome condensation 2 (RCC2), mRNA.	0.09	8.27	1.35E-08	3.12E-08	brown
REXO4	57109	REX4, RNA exonuclease 4 homolog (S. cerevisiae) (REXO4), mRNA.	-0.20	7.89	5.04E-27	2.96E-26	brown
RFWD3	55159	ring finger and WD repeat domain 3 (RFWD3), mRNA.	-0.11	8.89	9.95E-21	4.39E-20	brown

RHBDF2	79651	rhomboid 5 homolog 2 (Drosophila) (RHBDF2), transcript variant 2, mRNA.	-0.12	7.55	5.33E-18	2.07E-17	brown
RNF115	27246	zinc finger protein 364 (ZNF364), mRNA.	0.00	7.56	8.70E-01	8.90E-01	brown
RNMTL1	55178	RNA methyltransferase like 1 (RNMTL1), mRNA.	0.17	8.22	2.64E-19	1.09E-18	brown
RNPEP	6051	arginyl aminopeptidase (aminopeptidase B) (RNPEP), mRNA.	0.07	8.69	2.92E-05	5.34E-05	brown
RP9	6100	retinitis pigmentosa 9 (autosomal dominant) (RP9), mRNA.	-0.14	5.80	4.15E-20	1.79E-19	brown
RPA3	6119	replication protein A3, 14kDa (RPA3), mRNA.	-0.08	7.55	2.56E-09	6.18E-09	brown
RPL23A	6147	ribosomal protein L23a (RPL23A), mRNA.	-0.02	6.92	1.95E-01	2.31E-01	brown
RSPRY1	89970	ring finger and SPRY domain containing 1 (RSPRY1), mRNA.	0.05	10.41	8.69E-03	1.25E-02	brown
RSRC2	65117	arginine/serine-rich coiled-coil 2 (RSRC2), transcript variant 1, mRNA.	-0.08	10.31	1.07E-07	2.33E-07	brown
RTCA	8634	RNA terminal phosphate cyclase domain 1 (RTCD1), mRNA.	0.01	9.13	7.13E-01	7.47E-01	brown
RTCB	51493	chromosome 22 open reading frame 28 (C22orf28), mRNA.	0.02	7.89	3.47E-01	3.93E-01	brown
RUNX3	864	runt-related transcription factor 3 (RUNX3), transcript variant 2, mRNA.	0.02	8.18	1.76E-01	2.11E-01	brown

RXRB	6257	retinoid X receptor, beta (RXRB), mRNA.	-0.04	8.84	4.42E-02	5.81E-02	brown
SAFB	6294	scaffold attachment factor B (SAFB), mRNA.	0.08	6.63	3.23E-08	7.27E-08	brown
SCAMP3	10067	secretory carrier membrane protein 3 (SCAMP3), transcript variant 2, mRNA.	0.02	7.13	2.84E-01	3.26E-01	brown
SCARB1	949	scavenger receptor class B, member 1 (SCARB1), mRNA.	-0.35	6.28	1.00E-36	8.78E-36	brown
SCRIB	23513	scribbled homolog (Drosophila) (SCRIB), transcript variant 1, mRNA.	0.06	10.55	2.90E-07	6.13E-07	brown
SDCBP	6386	syndecan binding protein (syntenin) (SDCBP), transcript variant 5, mRNA.	0.02	10.10	1.55E-01	1.88E-01	brown
SDCCAG3	10807	serologically defined colon cancer antigen 3 (SDCCAG3), transcript variant 2, mRNA.	-0.01	10.17	5.76E-01	6.19E-01	brown
SF3A3	10946	splicing factor 3a, subunit 3, 60kDa (SF3A3), mRNA.	0.11	7.78	1.23E-12	3.59E-12	brown
SF3B2	10992	splicing factor 3b, subunit 2, 145kDa (SF3B2), mRNA.	-0.06	11.17	2.21E-05	4.09E-05	brown
SFMBT2	57713	Scm-like with four mbt domains 2 (SFMBT2), mRNA.	0.42	7.10	7.14E-51	1.15E-49	brown
SGSM2	9905	RUN and TBC1 domain containing 1 (RUTBC1), mRNA.	0.02	5.89	8.14E-02	1.03E-01	brown
SH3BGRL	6451	SH3 domain binding glutamic acid-rich protein like (SH3BGRL), mRNA.	-0.09	9.40	1.40E-13	4.29E-13	brown

SHMT1	6470	serine hydroxymethyltransferase 1 (soluble) (SHMT1), transcript variant 1, mRNA.	0.00	9.89	8.66E-01	8.87E-01	brown
SIPA1	6494	signal-induced proliferation-associated gene 1 (SIPA1), transcript variant 2, mRNA.	-0.23	8.64	8.60E-23	4.20E-22	brown
SIPA1L1	26037	signal-induced proliferation-associated 1 like 1 (SIPA1L1), mRNA.	-0.13	7.09	8.69E-18	3.34E-17	brown
SIRT7	51547	sirtuin (silent mating type information regulation 2 homolog) 7 (S. cerevisiae) (SIRT7), mRNA.	0.01	7.85	4.52E-01	4.99E-01	brown
SKAP2	8935	src kinase associated phosphoprotein 2 (SKAP2), mRNA.	-0.47	9.17	2.21E-58	5.49E-57	brown
SLC12A9	56996	solute carrier family 12 (potassium/chloride transporters), member 9 (SLC12A9), mRNA.	0.11	6.14	3.98E-16	1.40E-15	brown
SLC25A10	1468	solute carrier family 25 (mitochondrial carrier; dicarboxylate transporter), member 10 (SLC25A10), mRNA.	-0.01	9.75	5.79E-01	6.23E-01	brown

SLC25A19	60386	solute carrier family 25 (mitochondrial thiamine pyrophosphate carrier), member 19 (SLC25A19), mRNA.	0.39	9.52	3.27E-39	3.17E-38	brown
SLC25A5	292	solute carrier family 25 (mitochondrial carrier; adenine nucleotide translocator), member 5 (SLC25A5), mRNA.	0.03	8.49	4.40E-02	5.79E-02	brown
SLC26A6	65010	solute carrier family 26, member 6 (SLC26A6), transcript variant 1, mRNA.	0.02	9.85	1.46E-01	1.77E-01	brown
SLC35B2	347734	solute carrier family 35, member B2 (SLC35B2), mRNA.	-0.02	7.58	2.02E-01	2.40E-01	brown
SLC9A1	6548	solute carrier family 9 (sodium/hydrogen exchanger), member 1 (antiporter, Na+/H+, amiloride sensitive) (SLC9A1), mRNA.	0.01	9.98	4.53E-01	4.99E-01	brown
SLX4	84464	BTB (POZ) domain containing 12 (BTBD12), mRNA.	0.06	8.31	4.26E-06	8.32E-06	brown
SMARCA4	6597	SWI/SNF related, matrix associated, actin dependent regulator of chromatin, subfamily a, member 4 (SMARCA4), mRNA.	0.00	10.69	9.25E-01	9.36E-01	brown

SNAPC4	6621	small nuclear RNA activating complex, polypeptide 4, 190kDa (SNAPC4), mRNA.	-0.16	8.66	5.32E-27	3.12E-26	brown
SNRPB	6628	small nuclear ribonucleoprotein polypeptides B and B1 (SNRPB), transcript variant 1, mRNA.	0.03	6.87	4.65E-02	6.09E-02	brown
SNX27	81609	sorting nexin family member 27 (SNX27), mRNA.	-0.12	7.74	5.24E-10	1.32E-09	brown
SPCS1	28972	signal peptidase complex subunit 1 homolog (S. cerevisiae) (SPCS1), mRNA.	0.32	7.06	2.82E-41	2.98E-40	brown
SPECC1L	23384	SPECC1-like (SPECC1L), mRNA.	-0.02	9.53	8.23E-02	1.04E-01	brown
SPG7	6687	spastic paraplegia 7, paraplegin (pure and complicated autosomal recessive) (SPG7), nuclear gene encoding mitochondrial protein, transcript variant 1, mRNA.	-0.05	8.30	1.48E-03	2.30E-03	brown
SPHK2	56848	sphingosine kinase 2 (SPHK2), mRNA.	0.08	6.70	1.24E-06	2.51E-06	brown
SPNS1	83985	spinster (SPIN1), mRNA.	-0.22	5.84	4.43E-24	2.30E-23	brown
SPRYD3	84926	SPRY domain containing 3 (SPRYD3), mRNA.	-0.07	7.13	9.55E-06	1.82E-05	brown
SRSF5	6430	splicing factor, arginine/serine-rich 5 (SFRS5), transcript variant 1, mRNA.	0.05	8.55	6.47E-03	9.42E-03	brown
SRSF6	6431	splicing factor, arginine/serine-rich 6 (SFRS6), mRNA.	-0.11	9.75	1.45E-15	4.96E-15	brown

ST3GAL2	6483	ST3 beta-galactoside alpha-2,3-sialyltransferase 2 (ST3GAL2), mRNA.	-0.09	8.39	4.75E-10	1.20E-09	brown
STEAP3	55240	STEAP family member 3 (STEAP3), transcript variant 3, mRNA.	-0.06	7.28	2.91E-04	4.87E-04	brown
STK25	10494	serine/threonine kinase 25 (STE20 homolog, yeast) (STK25), mRNA.	-0.24	8.99	4.36E-37	3.86E-36	brown
STRADA	92335	protein kinase LYK5 (LYK5), transcript variant 2, mRNA.	-0.03	7.43	1.05E-01	1.31E-01	brown
STX16	8675	syntaxin 16 (STX16), transcript variant 2, mRNA.	0.08	5.46	8.12E-05	1.43E-04	brown
TACC1	6867	transforming, acidic coiled-coil containing protein 1 (TACC1), mRNA.	0.03	6.39	2.43E-02	3.30E-02	brown
TAF15	8148	TAF15 RNA polymerase II, TATA box binding protein (TBP)-associated factor, 68kDa (TAF15), transcript variant 1, mRNA.	0.20	9.10	3.97E-26	2.25E-25	brown
TAPBPL	55080	TAP binding protein-like (TAPBPL), mRNA.	0.01	8.37	6.19E-01	6.59E-01	brown
TATDN1	83940	TatD DNase domain containing 1 (TATDN1), mRNA.	-0.11	7.07	3.14E-14	9.98E-14	brown
TBL3	10607	transducin (beta)-like 3 (TBL3), mRNA.	0.43	8.31	2.78E-57	6.42E-56	brown
TCF25	22980	transcription factor 25 (basic helix-loop-helix) (TCF25), mRNA.	0.00	7.12	9.48E-01	9.55E-01	brown

TELO2	9894	TEL2, telomere maintenance 2, homolog (S. cerevisiae) (TELO2), mRNA.	0.17	9.21	1.39E-21	6.38E-21	brown
THEM6	51337	chromosome 8 open reading frame 55 (C8orf55), mRNA.	0.03	8.12	6.92E-02	8.87E-02	brown
THOC5	8563	THO complex 5 (THOC5), transcript variant 3, mRNA.	0.03	11.03	3.99E-02	5.27E-02	brown
TICAM1	148022	toll-like receptor adaptor molecule 1 (TICAM1), transcript variant 2, mRNA.	0.08	5.84	1.40E-07	3.03E-07	brown
TIMM22	29928	translocase of inner mitochondrial membrane 22 homolog (yeast) (TIMM22), mRNA.	0.09	10.08	3.67E-12	1.04E-11	brown
TIMM23	10431	translocase of inner mitochondrial membrane 23 homolog (yeast) (TIMM23), mRNA.	0.04	8.96	3.97E-02	5.24E-02	brown
TJAP1	93643	tight junction associated protein 1 (peripheral) (TJAP1), mRNA.	0.06	7.22	1.22E-04	2.10E-04	brown
TMED3	23423	transmembrane emp24 protein transport domain containing 3 (TMED3), mRNA.	0.11	8.76	8.35E-09	1.95E-08	brown
TMEM147	10430	transmembrane protein 147 (TMEM147), mRNA.	0.01	8.67	5.62E-01	6.06E-01	brown
TMEM175	84286	transmembrane protein 175 (TMEM175), mRNA.	-0.35	11.21	1.64E-69	7.32E-68	brown
TMEM219	124446	hypothetical protein BC017488 (LOC124446), mRNA.	-0.06	5.78	2.09E-05	3.87E-05	brown

TMEM87A	25963	transmembrane protein 87A (TMEM87A), mRNA.	0.09	7.09	2.43E-09	5.87E-09	brown
TNFRSF10B	8795	tumor necrosis factor receptor superfamily, member 10b (TNFRSF10B), transcript variant 1, mRNA.	-0.12	9.20	6.37E-15	2.10E-14	brown
TNFRSF13C	115650	tumor necrosis factor receptor superfamily, member 13C (TNFRSF13C), mRNA.	-0.11	7.58	2.89E-06	5.72E-06	brown
TNFRSF14	8764	tumor necrosis factor receptor superfamily, member 14 (herpesvirus entry mediator) (TNFRSF14), mRNA.	0.15	8.34	1.25E-15	4.27E-15	brown
TNFRSF1B	7133	tumor necrosis factor receptor superfamily, member 1B (TNFRSF1B), mRNA.	-0.03	10.48	9.57E-03	1.36E-02	brown
TOMM34	10953	translocase of outer mitochondrial membrane 34 (TOMM34), nuclear gene encoding mitochondrial protein, mRNA.	-0.10	6.68	2.28E-08	5.17E-08	brown
TOMM40	10452	translocase of outer mitochondrial membrane 40 homolog (yeast) (TOMM40), mRNA.	0.19	8.36	1.49E-22	7.18E-22	brown
TOP3B	8940	topoisomerase (DNA) III beta (TOP3B), mRNA.	0.03	8.57	9.95E-03	1.42E-02	brown

TPD52L2	7165	tumor protein D52-like 2 (TPD52L2), transcript variant 4, mRNA.	-0.10	7.79	7.00E-15	2.30E-14	brown
TPK1	27010	thiamin pyrophosphokinase 1 (TPK1), transcript variant 1, mRNA.	-0.01	7.06	5.95E-01	6.37E-01	brown
TRABD	80305	TraB domain containing (TRABD), mRNA.	-0.05	6.69	1.81E-03	2.79E-03	brown
TRIM24	8805	tripartite motif-containing 24 (TRIM24), transcript variant 1, mRNA.	-0.01	10.37	3.89E-01	4.35E-01	brown
TRMT1	55621	TRM1 tRNA methyltransferase 1 homolog (S. cerevisiae) (TRMT1), mRNA.	0.00	8.97	8.90E-01	9.07E-01	brown
TSPAN33	340348	tetraspanin 33 (TSPAN33), mRNA.	0.04	7.19	5.10E-04	8.34E-04	brown
TUBA1B	10376	tubulin, alpha 1b (TUBA1B), mRNA.	0.01	8.65	4.49E-01	4.95E-01	brown
TUBA1C	84790	tubulin, alpha 1c (TUBA1C), mRNA.	-0.28	8.11	3.26E-44	3.99E-43	brown
TUBG1	7283	tubulin, gamma 1 (TUBG1), mRNA.	0.11	9.75	3.16E-16	1.12E-15	brown
TXLNA	200081	taxilin alpha (TXLNA), mRNA.	-0.07	8.95	1.81E-05	3.38E-05	brown
TYK2	7297	tyrosine kinase 2 (TYK2), mRNA.	-0.30	7.51	2.21E-27	1.32E-26	brown
TYSND1	219743	trypsin domain containing 1 (TYSND1), transcript variant 2, mRNA.	-0.03	5.41	5.17E-02	6.73E-02	brown
UBAP2	55833	ubiquitin associated protein 2 (UBAP2), mRNA.	0.24	7.13	1.66E-36	1.43E-35	brown
UBE2L3	7332	ubiquitin-conjugating enzyme E2L 3 (UBE2L3), transcript variant 1, mRNA.	-0.18	9.49	7.71E-27	4.49E-26	brown

UBN1	29855	ubinuclein 1 (UBN1), transcript variant 1, mRNA.	0.03	7.78	1.01E-01	1.26E-01	brown
UCHL5	51377	ubiquitin carboxyl-terminal hydrolase L5 (UCHL5), mRNA.	0.08	6.51	2.56E-06	5.06E-06	brown
UCP2	7351	uncoupling protein 2 (mitochondrial, proton carrier) (UCP2), nuclear gene encoding mitochondrial protein, mRNA.	-0.16	6.01	2.60E-21	1.18E-20	brown
UIMC1	51720	ubiquitin interaction motif containing 1 (UIMC1), mRNA.	0.16	8.62	2.21E-18	8.69E-18	brown
ULK1	8408	unc-51-like kinase 1 (C. elegans) (ULK1), mRNA.	-0.07	6.50	9.22E-06	1.76E-05	brown
VARS2	57176	valyl-tRNA synthetase 2, mitochondrial (putative) (VARS2), mRNA.	0.06	8.12	2.09E-05	3.86E-05	brown
VBP1	7411	von Hippel-Lindau binding protein 1 (VBP1), mRNA.	-0.01	5.40	4.59E-01	5.05E-01	brown
VDAC2	7417	voltage-dependent anion channel 2 (VDAC2), mRNA.	0.13	10.13	1.14E-15	3.93E-15	brown
VEGFB	7423	vascular endothelial growth factor B (VEGFB), mRNA.	0.11	5.68	4.17E-08	9.30E-08	brown
VPS16	64601	vacuolar protein sorting 16 homolog (S. cerevisiae) (VPS16), transcript variant 2, mRNA.	0.02	10.47	7.27E-02	9.29E-02	brown
VPS39	23339	vacuolar protein sorting 39 homolog (S. cerevisiae) (VPS39), mRNA.	-0.22	7.02	5.37E-31	3.60E-30	brown
VWCE	220001	von Willebrand factor C and EGF domains (VWCE), mRNA.	-0.04	6.37	4.18E-03	6.19E-03	brown

WBP2	23558	WW domain binding protein 2 (WBP2), mRNA.	0.02	7.23	1.92E-01	2.28E-01	brown
WDR18	57418	WD repeat domain 18 (WDR18), mRNA.	0.14	7.07	1.07E-16	3.92E-16	brown
WDR60	55112	WD repeat domain 60 (WDR60), mRNA.	0.02	7.62	2.77E-01	3.20E-01	brown
WRB	7485	tryptophan rich basic protein (WRB), mRNA.	0.11	9.05	1.68E-16	6.04E-16	brown
XRCC6BP1	91419	XRCC6 binding protein 1 (XRCC6BP1), mRNA.	0.05	6.91	1.47E-02	2.05E-02	brown
YARS	8565	tyrosyl-tRNA synthetase (YARS), mRNA.	0.04	6.42	1.17E-02	1.65E-02	brown
YIPF4	84272	Yip1 domain family, member 4 (YIPF4), mRNA.	-0.02	7.60	1.17E-01	1.45E-01	brown
YY1	7528	YY1 transcription factor (YY1), mRNA.	0.03	9.01	2.65E-02	3.58E-02	brown
ZCCHC6	79670	zinc finger, CCHC domain containing 6 (ZCCHC6), mRNA.	-0.05	7.49	4.22E-05	7.59E-05	brown
ZDHHC16	84287	zinc finger, DHHC-type containing 16 (ZDHHC16), transcript variant 5, mRNA.	0.04	11.17	8.46E-03	1.22E-02	brown
ZDHHC5	25921	zinc finger, DHHC-type containing 5 (ZDHHC5), mRNA.	0.21	9.69	2.35E-25	1.29E-24	brown
ZDHHC8	29801	zinc finger, DHHC-type containing 8 (ZDHHC8), mRNA.	-0.02	8.94	2.10E-01	2.48E-01	brown
ZGPAT	84619	zinc finger, CCCH-type with G patch domain (ZGPAT), transcript variant 2, mRNA.	0.02	8.10	3.10E-01	3.55E-01	brown
ZMIZ2	83637	zinc finger, MIZ-type containing 2 (ZMIZ2), transcript variant 1, mRNA.	0.05	8.91	4.63E-03	6.84E-03	brown

ZMYND19	116225	zinc finger, MYND-type containing 19 (ZMYND19), mRNA.	-0.04	8.60	2.47E-03	3.75E-03	brown
ZNF142	7701	zinc finger protein 142 (ZNF142), mRNA.	0.18	7.44	1.42E-27	8.55E-27	brown
ZNF329	79673	zinc finger protein 329 (ZNF329), mRNA.	-0.18	9.22	1.34E-19	5.62E-19	brown
ZNF428	126299	zinc finger protein 428 (ZNF428), mRNA.	-0.64	8.14	1.42E-81	1.38E-79	brown
ZNF512	84450	zinc finger protein 512 (ZNF512), mRNA.	-0.03	9.42	7.23E-02	9.24E-02	brown
ZNF672	79894	zinc finger protein 672 (ZNF672), mRNA.	-0.23	8.10	3.67E-36	3.11E-35	brown
ZNF689	115509	zinc finger protein 689 (ZNF689), mRNA.	-0.04	10.11	3.24E-03	4.86E-03	brown
ZNF777	27153	zinc finger protein 777 (ZNF777), mRNA.	0.12	9.11	8.91E-13	2.62E-12	brown
ZSCAN21	7589	zinc finger and SCAN domain containing 21 (ZSCAN21), mRNA.	-0.01	6.53	5.97E-01	6.38E-01	brown
ZSWIM1	90204	zinc finger, SWIM-type containing 1 (ZSWIM1), mRNA.	0.00	7.18	8.88E-01	9.06E-01	brown
ACYP1	97	acylphosphatase 1, erythrocyte (common) type (ACYP1), transcript variant 2, mRNA.	-0.14	6.26	7.78E-17	2.85E-16	cyan
ADGRE5	976	CD97 molecule (CD97), transcript variant 3, mRNA.	0.16	9.81	3.72E-31	2.52E-30	cyan
AK2	204	adenylate kinase 2 (AK2), transcript variant AK2A, mRNA.	0.24	9.71	2.31E-34	1.80E-33	cyan

APOA1BP	128240	apolipoprotein A-I binding protein (APOA1BP), mRNA.	0.10	11.34	6.51E-13	1.93E-12	cyan
APOOL	139322	apolipoprotein O-like (APOOL), mRNA.	0.03	7.00	6.72E-02	8.63E-02	cyan
ATP5J	522	ATP synthase, H+ transporting, mitochondrial F0 complex, subunit F6 (ATP5J), nuclear gene encoding mitochondrial protein, transcript variant 1, mRNA.	0.39	8.17	4.17E-47	5.99E-46	cyan
BOLA3	388962	bolA homolog 3 (E. coli) (BOLA3), transcript variant 2, mRNA.	0.02	7.31	1.68E-01	2.02E-01	cyan
BTF3	689	basic transcription factor 3 (BTF3), transcript variant 2, mRNA.	-0.21	6.90	4.48E-28	2.74E-27	cyan
C14orf2	9556	chromosome 14 open reading frame 2 (C14orf2), mRNA.	0.45	11.41	1.83E-82	1.85E-80	cyan
CCDC23	374969	coiled-coil domain containing 23 (CCDC23), mRNA.	-0.01	12.05	4.81E-01	5.26E-01	cyan
CCDC58	131076	coiled-coil domain containing 58 (CCDC58), mRNA.	-0.18	6.42	8.20E-21	3.63E-20	cyan
CDV3	55573	CDV3 homolog (mouse) (CDV3), mRNA. XM_945284 XM_945286 XM_945287	0.15	8.26	8.69E-31	5.78E-30	cyan
CHFR	55743	checkpoint with forkhead and ring finger domains (CHFR), mRNA.	-0.50	9.01	1.90E-75	1.13E-73	cyan
CKS1B	1163	CDC28 protein kinase regulatory subunit 1B (CKS1B), mRNA.	0.12	8.15	8.13E-09	1.90E-08	cyan

CKS2	1164	CDC28 protein kinase regulatory subunit 2 (CKS2), mRNA.	0.07	8.04	1.97E-03	3.02E-03	cyan
CLPTM1L	81037	CLPTM1-like (CLPTM1L), mRNA.	0.05	8.23	1.08E-03	1.70E-03	cyan
CMC1	152100	hypothetical protein MGC61571 (MGC61571), mRNA.	0.11	9.83	5.98E-12	1.68E-11	cyan
CMSS1	84319	chromosome 3 open reading frame 26 (C3orf26), mRNA.	0.12	8.22	1.71E-12	4.94E-12	cyan
CNIH4	29097	cornichon homolog 4 (Drosophila) (CNIH4), mRNA.	-0.08	8.05	7.06E-10	1.76E-09	cyan
COMMD6	170622	COMM domain containing 6 (COMMD6), transcript variant 2, mRNA.	0.03	9.22	6.65E-02	8.53E-02	cyan
COQ3	51805	coenzyme Q3 homolog, methyltransferase (S. cerevisiae) (COQ3), mRNA.	0.01	8.34	4.89E-01	5.34E-01	cyan
COX6B1	1340	cytochrome c oxidase subunit Vib polypeptide 1 (ubiquitous) (COX6B1), mRNA.	0.04	6.83	1.01E-01	1.26E-01	cyan
COX7A2	1347	cytochrome c oxidase subunit VIIa polypeptide 2 (liver) (COX7A2), mRNA.	0.14	8.19	3.93E-14	1.24E-13	cyan
COX7B	1349	cytochrome c oxidase subunit VIIb (COX7B), nuclear gene encoding mitochondrial protein, mRNA.	0.02	9.22	3.01E-01	3.45E-01	cyan

COX7C	1350	cytochrome c oxidase subunit VIIc (COX7C), nuclear gene encoding mitochondrial protein, mRNA.	-0.01	6.79	3.94E-01	4.39E-01	cyan
CSTF3	1479	cleavage stimulation factor, 3' pre-RNA, subunit 3, 77kDa (CSTF3), transcript variant 2, mRNA.	0.08	8.16	1.29E-05	2.43E-05	cyan
DDX23	9416	DEAD (Asp-Glu-Ala-Asp) box polypeptide 23 (DDX23), mRNA.	0.02	5.54	1.31E-01	1.61E-01	cyan
DPY30	84661	dpy-30-like protein (LOC84661), mRNA.	0.21	8.05	5.02E-52	8.52E-51	cyan
EEF2	1938	eukaryotic translation elongation factor 2 (EEF2), mRNA.	-0.06	10.04	2.93E-05	5.37E-05	cyan
ENO1	2023	enolase 1, (alpha) (ENO1), mRNA.	0.10	7.81	3.44E-11	9.22E-11	cyan
ERH	2079	enhancer of rudimentary homolog (Drosophila) (ERH), mRNA.	-0.02	7.87	1.07E-01	1.33E-01	cyan
FAM217B	63939	chromosome 20 open reading frame 177 (C20orf177), mRNA.	0.10	8.75	6.86E-09	1.62E-08	cyan
GCDH	2639	glutaryl-Coenzyme A dehydrogenase (GCDH), nuclear gene encoding mitochondrial protein, transcript variant 1, mRNA.	0.04	9.04	1.32E-03	2.06E-03	cyan
GEMIN6	79833	gem (nuclear organelle) associated protein 6 (GEMIN6), mRNA.	-0.13	8.58	5.23E-12	1.47E-11	cyan
GOLIM4	27333	golgi phosphoprotein 4 (GOLPH4), mRNA.	-0.02	8.00	3.26E-01	3.71E-01	cyan

GTF2H5	404672	general transcription factor IIH, polypeptide 5 (GTF2H5), mRNA.	0.08	7.16	4.19E-05	7.54E-05	cyan
HCLS1	3059	hematopoietic cell-specific Lyn substrate 1 (HCLS1), mRNA.	-0.09	7.57	4.85E-07	1.01E-06	cyan
HINT1	3094	histidine triad nucleotide binding protein 1 (HINT1), mRNA.	-0.01	7.36	6.82E-01	7.17E-01	cyan
HIST1H4C	8364	histone cluster 1, H4c (HIST1H4C), mRNA.	-0.16	8.42	4.34E-15	1.44E-14	cyan
HLA-B	3106	major histocompatibility complex, class I, B (HLA-B), mRNA.	-0.14	8.24	2.79E-13	8.45E-13	cyan
HNRNPUL1	11100	heterogeneous nuclear ribonucleoprotein U-like 1 (HNRPUL1), transcript variant 1, mRNA.	-0.07	8.97	1.08E-08	2.52E-08	cyan
IMMP1L	196294	IMP1 inner mitochondrial membrane peptidase-like (S. cerevisiae) (IMMP1L), mRNA.	0.05	8.11	5.36E-04	8.74E-04	cyan
IMPDH2	3615	IMP (inosine monophosphate) dehydrogenase 2 (IMPDH2), mRNA.	-0.09	7.97	1.69E-12	4.89E-12	cyan
INO80C	125476	chromosome 18 open reading frame 37 (C18orf37), mRNA.	-0.08	7.53	1.55E-06	3.11E-06	cyan
INPP4B	8821	inositol polyphosphate-4-phosphatase, type II, 105kDa (INPP4B), mRNA.	0.05	10.11	1.47E-02	2.04E-02	cyan

KIAA0101	9768	KIAA0101 (KIAA0101), transcript variant 1, mRNA.	-0.39	7.70	2.94E-44	3.60E-43	cyan
LSM3	27258	LSM3 homolog, U6 small nuclear RNA associated (S. cerevisiae) (LSM3), mRNA.	0.11	6.85	7.40E-09	1.74E-08	cyan
LY96	23643	lymphocyte antigen 96 (LY96), mRNA.	0.07	8.87	1.20E-07	2.62E-07	cyan
LYPLAL1	127018	lysophospholipase-like 1 (LYPLAL1), mRNA.	0.21	7.05	8.73E-23	4.26E-22	cyan
LYRM2	57226	LYR motif containing 2 (LYRM2), mRNA.	0.12	7.81	2.02E-13	6.14E-13	cyan
MAGOH	4116	mago-nashi homolog, proliferation-associated (Drosophila) (MAGOH), mRNA.	0.00	7.86	9.37E-01	9.47E-01	cyan
MELK	9833	maternal embryonic leucine zipper kinase (MELK), mRNA.	0.06	9.24	2.41E-06	4.79E-06	cyan
MOB4	25843	preimplantation protein 3 (PREI3), transcript variant 1, mRNA.	-0.28	7.42	8.18E-42	8.95E-41	cyan
MRPL22	29093	mitochondrial ribosomal protein L22 (MRPL22), nuclear gene encoding mitochondrial protein, transcript variant 1, mRNA.	0.29	6.75	2.87E-44	3.54E-43	cyan
MRPL33	9553	mitochondrial ribosomal protein L33 (MRPL33), nuclear gene encoding mitochondrial protein, transcript variant 2, mRNA.	-0.14	6.00	2.25E-12	6.44E-12	cyan

MRPL40	64976	mitochondrial ribosomal protein L40 (MRPL40), nuclear gene encoding mitochondrial protein, mRNA.	-0.13	7.10	1.57E-20	6.88E-20	cyan
MRPL48	51642	mitochondrial ribosomal protein L48 (MRPL48), nuclear gene encoding mitochondrial protein, mRNA.	0.03	11.35	4.59E-02	6.01E-02	cyan
MRPS17	51373	mitochondrial ribosomal protein S17 (MRPS17), nuclear gene encoding mitochondrial protein, mRNA.	0.01	8.46	6.05E-01	6.46E-01	cyan
MRPS18C	51023	mitochondrial ribosomal protein S18C (MRPS18C), nuclear gene encoding mitochondrial protein, mRNA.	0.40	7.78	3.11E-55	6.29E-54	cyan
MRPS21	54460	mitochondrial ribosomal protein S21 (MRPS21), nuclear gene encoding mitochondrial protein, transcript variant 1, mRNA.	-0.50	7.56	3.76E-63	1.24E-61	cyan
MRPS33	51650	mitochondrial ribosomal protein S33 (MRPS33), nuclear gene encoding mitochondrial protein, transcript variant 2, mRNA.	-0.06	7.78	6.79E-05	1.20E-04	cyan
NA	56617	small EDRK-rich factor 1B (centromeric) (SERF1B), mRNA.	-0.40	7.80	1.42E-67	5.62E-66	cyan

NA	514	ATP synthase, H+ transporting, mitochondrial F1 complex, epsilon subunit (ATP5E), nuclear gene encoding mitochondrial protein, mRNA.	-0.05	7.73	2.13E-03	3.25E-03	cyan
NA	6161	ribosomal protein L32 (RPL32), transcript variant 3, mRNA.	-0.04	8.28	2.25E-02	3.07E-02	cyan
NA	4738	neural precursor cell expressed, developmentally down-regulated 8 (NEDD8), mRNA.	-0.03	7.69	1.56E-01	1.89E-01	cyan
NDUFA1	4694	NADH dehydrogenase (ubiquinone) 1 alpha subcomplex, 1, 7.5kDa (NDUFA1), nuclear gene encoding mitochondrial protein, mRNA.	-0.03	7.11	1.33E-01	1.63E-01	cyan
NDUFA4	4697	NADH dehydrogenase (ubiquinone) 1 alpha subcomplex, 4, 9kDa (NDUFA4), nuclear gene encoding mitochondrial protein, mRNA.	0.04	11.96	7.72E-04	1.24E-03	cyan
NDUFAF2	91942	NDUFA12-like (NDUFA12L), mRNA.	-0.19	8.22	7.94E-23	3.89E-22	cyan
NDUFB3	4709	NADH dehydrogenase (ubiquinone) 1 beta subcomplex, 3, 12kDa (NDUFB3), mRNA.	0.11	6.66	5.81E-09	1.38E-08	cyan

NDUFS4	4724	NADH dehydrogenase (ubiquinone) Fe-S protein 4, 18kDa (NADH-coenzyme Q reductase) (NDUFS4), mRNA.	0.00	9.64	8.74E-01	8.94E-01	cyan
NDUFS5	4725	NADH dehydrogenase (ubiquinone) Fe-S protein 5, 15kDa (NADH-coenzyme Q reductase) (NDUFS5), mRNA.	0.02	9.77	5.52E-02	7.16E-02	cyan
NELFCD	51497	TH1-like (Drosophila) (TH1L), transcript variant 1, mRNA.	-0.23	8.54	4.50E-43	5.26E-42	cyan
NHP2L1	4809	NHP2 non-histone chromosome protein 2-like 1 (S. cerevisiae) (NHP2L1), transcript variant 2, mRNA.	0.13	7.29	1.59E-15	5.42E-15	cyan
NIT2	56954	nitrilase family, member 2 (NIT2), mRNA.	-0.21	6.95	1.03E-28	6.44E-28	cyan
PABPC1	26986	poly(A) binding protein, cytoplasmic 1 (PABPC1), mRNA.	0.05	8.56	5.30E-03	7.77E-03	cyan
PFDN5	5204	prefoldin subunit 5 (PFDN5), transcript variant 1, mRNA.	-0.04	7.90	1.74E-03	2.69E-03	cyan
POLR2H	5437	polymerase (RNA) II (DNA directed) polypeptide H (POLR2H), mRNA.	0.17	8.93	2.09E-19	8.67E-19	cyan
PTRHD1	391356	similar to CG14903-PA (LOC391356), mRNA.	-0.10	9.71	1.65E-17	6.27E-17	cyan
RBX1	9978	ring-box 1 (RBX1), mRNA.	0.16	9.65	5.54E-39	5.33E-38	cyan
RPL26	6154	ribosomal protein L26 (RPL26), mRNA.	0.10	7.25	2.55E-06	5.05E-06	cyan

RPL26L1	51121	ribosomal protein L26-like 1 (RPL26L1), mRNA.	0.03	8.26	5.92E-02	7.64E-02	cyan
RPL30	6156	ribosomal protein L30 (RPL30), mRNA.	-0.05	7.75	1.43E-03	2.22E-03	cyan
RPL31	6160	ribosomal protein L31 (RPL31), mRNA.	0.06	7.72	1.40E-03	2.18E-03	cyan
RPL34	6164	ribosomal protein L34 (RPL34), transcript variant 2, mRNA.	0.04	6.69	4.67E-02	6.11E-02	cyan
RPL35A	6165	ribosomal protein L35a (RPL35A), mRNA.	-0.05	7.04	2.04E-03	3.13E-03	cyan
RPL36AL	6166	ribosomal protein L36a-like (RPL36AL), mRNA.	-0.09	10.80	4.66E-18	1.81E-17	cyan
RPL39	6170	ribosomal protein L39 (RPL39), mRNA.	-0.07	7.46	1.69E-05	3.16E-05	cyan
RPL5	6125	ribosomal protein L5 (RPL5), mRNA.	0.09	10.89	2.03E-07	4.34E-07	cyan
RPP40	10799	ribonuclease P 40kDa subunit (RPP40), mRNA.	0.00	10.75	7.93E-01	8.19E-01	cyan
RPS13	6207	ribosomal protein S13 (RPS13), mRNA.	-0.12	8.99	6.00E-13	1.78E-12	cyan
RPS15	6209	ribosomal protein S15 (RPS15), mRNA.	-0.10	9.12	6.08E-18	2.35E-17	cyan
RPS16	6217	ribosomal protein S16 (RPS16), mRNA.	0.61	8.24	2.29E-88	3.98E-86	cyan
RPS17	6218	ribosomal protein S17 (RPS17), mRNA.	0.20	8.32	2.68E-21	1.21E-20	cyan
RPS21	6227	ribosomal protein S21 (RPS21), mRNA.	0.01	12.50	4.58E-01	5.04E-01	cyan
RPS27A	6233	ribosomal protein S27a (RPS27A), mRNA.	-0.05	7.41	1.86E-03	2.87E-03	cyan

RPS27L	51065	ribosomal protein S27-like (RPS27L), mRNA.	0.11	9.66	1.30E-10	3.38E-10	cyan
RPS7	6201	ribosomal protein S7 (RPS7), mRNA.	0.05	8.02	6.58E-04	1.06E-03	cyan
RPS8	6202	ribosomal protein S8 (RPS8), mRNA.	0.49	7.60	3.48E-61	1.02E-59	cyan
SEC61G	23480	Sec61 gamma subunit (SEC61G), transcript variant 2, mRNA.	0.02	8.07	1.73E-01	2.07E-01	cyan
SF3B6	51639	splicing factor 3B, 14 kDa subunit (SF3B14), mRNA.	-0.15	6.92	4.18E-13	1.25E-12	cyan
SHFM1	7979	split hand/foot malformation (ectrodactyly) type 1 (SHFM1), mRNA.	0.02	6.08	3.04E-01	3.48E-01	cyan
SLIRP	81892	chromosome 14 open reading frame 156 (C14orf156), mRNA.	0.31	9.01	2.64E-46	3.63E-45	cyan
SNRPB2	6629	small nuclear ribonucleoprotein polypeptide B" (SNRPB2), transcript variant 1, mRNA.	0.05	6.24	2.82E-02	3.79E-02	cyan
SNRPD2	6633	small nuclear ribonucleoprotein D2 polypeptide 16.5kDa (SNRPD2), transcript variant 1, mRNA.	-0.13	8.85	1.38E-25	7.70E-25	cyan
SNRPF	6636	small nuclear ribonucleoprotein polypeptide F (SNRPF), mRNA.	0.23	7.46	7.26E-26	4.07E-25	cyan
STX8	9482	syntaxin 8 (STX8), mRNA.	-0.04	8.78	1.11E-03	1.75E-03	cyan
TCEB1	6921	transcription elongation factor B (SIII), polypeptide 1 (15kDa, elongin C) (TCEB1), mRNA.	0.09	9.97	9.17E-13	2.69E-12	cyan

TKT	7086	transketolase (Wernicke-Korsakoff syndrome) (TKT), mRNA.	-0.08	9.44	1.42E-05	2.66E-05	cyan
TMBIM4	51643	transmembrane BAX inhibitor motif containing 4 (TMBIM4), mRNA.	0.46	8.34	6.43E-42	7.10E-41	cyan
TMEM258	746	chromosome 11 open reading frame 10 (C11orf10), mRNA.	-0.02	7.27	1.68E-01	2.02E-01	cyan
TNIK	23043	TRAF2 and NCK interacting kinase (TNIK), mRNA.	-0.14	7.09	1.59E-20	6.96E-20	cyan
TOMM22	56993	translocase of outer mitochondrial membrane 22 homolog (yeast) (TOMM22), nuclear gene encoding mitochondrial protein, mRNA.	0.11	10.48	6.75E-10	1.68E-09	cyan
TOMM6	29964	chromosome 6 open reading frame 49 (C6orf49), mRNA.	-0.15	8.41	3.43E-16	1.21E-15	cyan
TOMM7	54543	translocase of outer mitochondrial membrane 7 homolog (yeast) (TOMM7), mRNA.	-0.17	6.57	1.73E-30	1.13E-29	cyan
TPRKB	51002	TP53RK binding protein (TPRKB), mRNA.	0.13	8.29	7.14E-19	2.89E-18	cyan
TXN	7295	thioredoxin (TXN), mRNA.	-0.21	8.75	1.56E-24	8.27E-24	cyan
UQCRB	7381	ubiquinol-cytochrome c reductase binding protein (UQCRB), mRNA.	0.04	7.17	3.66E-03	5.46E-03	cyan
VPS29	51699	vacuolar protein sorting 29 (yeast) (VPS29), transcript variant 1, mRNA.	0.11	10.06	8.00E-13	2.36E-12	cyan

ZBTB43	23099	zinc finger and BTB domain containing 43 (ZBTB43), mRNA.	0.06	6.30	2.32E-06	4.61E-06	cyan
ZNF585A	199704	zinc finger protein 585A (ZNF585A), transcript variant 1, mRNA.	-0.04	8.02	9.37E-03	1.34E-02	cyan
MARCH7	64844	membrane-associated ring finger (C3HC4) 7 (MARCH7), mRNA.	-0.11	9.98	3.80E-09	9.11E-09	green
SEPT7	989	septin 7 (SEPT7), transcript variant 2, mRNA.	-0.07	5.84	1.08E-06	2.19E-06	green
SEPT11	55752	septin 11 (SEPT11), mRNA.	0.02	7.00	1.97E-01	2.35E-01	green
SEP15	9403	15 kDa selenoprotein (SEP15), transcript variant 2, mRNA.	-0.09	7.90	7.90E-13	2.33E-12	green
ABCA5	23461	ATP-binding cassette, sub-family A (ABC1), member 5 (ABCA5), transcript variant 2, mRNA.	-0.02	7.75	1.31E-01	1.61E-01	green
ABCE1	6059	ATP-binding cassette, sub-family E (OABP), member 1 (ABCE1), transcript variant 1, mRNA.	0.20	9.29	1.56E-27	9.36E-27	green
ABHD17B	51104	chromosome 9 open reading frame 77 (C9orf77), transcript variant 2, mRNA.	0.02	8.94	1.55E-01	1.88E-01	green
ACAA1	30	acetyl-Coenzyme A acyltransferase 1 (peroxisomal 3-oxoacyl-Coenzyme A thiolase) (ACAA1), nuclear gene encoding mitochondrial protein, mRNA.	-0.06	5.98	3.60E-05	6.52E-05	green

ACADVL	37	acyl-Coenzyme A dehydrogenase, very long chain (ACADVL), nuclear gene encoding mitochondrial protein, transcript variant 2, mRNA.	0.01	7.79	6.37E-01	6.76E-01	green
ACSL1	2180	acyl-CoA synthetase long-chain family member 1 (ACSL1), mRNA.	-0.19	8.07	8.40E-22	3.89E-21	green
ACTR3	10096	ARP3 actin-related protein 3 homolog (yeast) (ACTR3), mRNA.	0.53	7.25	2.71E-58	6.71E-57	green
ADNP	23394	activity-dependent neuroprotector (ADNP), transcript variant 2, mRNA.	0.66	5.96	2.92E-56	6.31E-55	green
AFTPH	54812	aftiphilin (AFTPH), transcript variant 3, mRNA.	-0.02	7.23	3.48E-01	3.93E-01	green
AGPAT5	55326	1-acylglycerol-3-phosphate O-acyltransferase 5 (lysophosphatidic acid acyltransferase, epsilon) (AGPAT5), mRNA.	0.04	7.50	1.58E-02	2.20E-02	green
AHCTF1	25909	AT hook containing transcription factor 1 (AHCTF1), mRNA.	0.00	9.55	7.42E-01	7.73E-01	green
ALG5	29880	asparagine-linked glycosylation 5 homolog (S. cerevisiae, dolichyl-phosphate beta-glucosyltransferase) (ALG5), mRNA.	-0.38	6.45	2.90E-42	3.28E-41	green
ANXA4	307	annexin A4 (ANXA4), mRNA.	0.15	9.25	4.41E-11	1.18E-10	green

AP1AR	55435	chromosome 4 open reading frame 16 (C4orf16), mRNA.	-0.04	5.63	2.70E-03	4.07E-03	green
ARL8B	55207	ADP-ribosylation factor-like 8B (ARL8B), mRNA.	-0.05	8.69	1.10E-03	1.74E-03	green
ARMC1	55156	armadillo repeat containing 1 (ARMC1), mRNA.	0.11	7.44	3.38E-08	7.59E-08	green
ASUN	55726	chromosome 12 open reading frame 11 (C12orf11), mRNA.	0.08	7.92	5.65E-08	1.25E-07	green
ATF4	468	activating transcription factor 4 (tax-responsive enhancer element B67) (ATF4), transcript variant 1, mRNA.	-0.23	9.38	3.87E-32	2.76E-31	green
AVEN	57099	apoptosis, caspase activation inhibitor (AVEN), mRNA.	-0.11	7.97	2.86E-11	7.72E-11	green
BAZ2B	29994	bromodomain adjacent to zinc finger domain, 2B (BAZ2B), mRNA.	-0.09	6.30	1.94E-08	4.43E-08	green
BTAF1	9044	BTAF1 RNA polymerase II, B-TFIID transcription factor-associated, 170kDa (Mot1 homolog, S. cerevisiae) (BTAF1), mRNA.	-0.02	8.58	2.43E-01	2.84E-01	green
C2orf76	130355	hypothetical protein LOC130355 (LOC130355), mRNA.	0.07	6.57	3.50E-07	7.35E-07	green
CAMSAP2	23271	calmodulin regulated spectrin-associated protein 1-like 1 (CAMSAP1L1), mRNA.	0.02	7.99	2.67E-01	3.09E-01	green

CAPZA1	829	capping protein (actin filament) muscle Z-line, alpha 1 (CAPZA1), mRNA.	-0.01	5.97	5.66E-01	6.10E-01	green
CCAR1	55749	cell division cycle and apoptosis regulator 1 (CCAR1), mRNA.	0.06	7.24	2.28E-04	3.85E-04	green
CCBL2	56267	cysteine conjugate-beta lyase 2 (CCBL2), transcript variant 2, mRNA.	-0.03	9.07	7.64E-02	9.72E-02	green
CCDC50	152137	coiled-coil domain containing 50 (CCDC50), transcript variant 1, mRNA.	0.08	7.19	2.89E-05	5.29E-05	green
CCNB1	891	cyclin B1 (CCNB1), mRNA.	-0.11	6.75	1.73E-10	4.47E-10	green
CCNG1	900	cyclin G1 (CCNG1), transcript variant 1, mRNA.	0.16	6.70	1.09E-16	3.98E-16	green
CCP110	9738	CP110 protein (CP110), mRNA.	0.02	10.13	3.68E-02	4.88E-02	green
CCT3	7203	chaperonin containing TCP1, subunit 3 (gamma) (CCT3), transcript variant 2, mRNA.	-0.83	7.82	1.71E-89	3.21E-87	green
CD164	8763	CD164 molecule, sialomucin (CD164), mRNA.	0.02	7.14	1.30E-01	1.60E-01	green
CD58	965	CD58 molecule (CD58), mRNA.	-0.03	6.20	1.46E-01	1.78E-01	green
CDC5L	988	CDC5 cell division cycle 5-like (S. pombe) (CDC5L), mRNA.	0.73	9.25	1.44E-79	1.28E-77	green
CDC7	8317	cell division cycle 7 homolog (S. cerevisiae) (CDC7), mRNA.	0.05	6.68	8.06E-05	1.42E-04	green

CDK1	983	cell division cycle 2, G1 to S and G2 to M (CDC2), transcript variant 1, mRNA.	-0.10	8.12	2.99E-11	8.05E-11	green
CEBPZ	10153	CCAAT/enhancer binding protein zeta (CEBPZ), mRNA.	0.04	8.96	1.28E-04	2.22E-04	green
CENPJ	55835	centromere protein J (CENPJ), mRNA.	-0.25	8.88	1.42E-46	1.98E-45	green
CENPQ	55166	centromere protein Q (CENPQ), mRNA.	-0.28	9.81	3.14E-34	2.42E-33	green
CENPW	387103	chromosome 6 open reading frame 173 (C6orf173), mRNA.	-0.05	6.97	8.91E-03	1.28E-02	green
CHAC2	494143	ChaC, cation transport regulator homolog 2 (E. coli) (CHAC2), mRNA.	0.24	5.97	3.40E-33	2.51E-32	green
CHD1	1105	chromodomain helicase DNA binding protein 1 (CHD1), mRNA.	-0.73	8.48	5.43E-70	2.48E-68	green
CHMP2B	25978	chromatin modifying protein 2B (CHMP2B), mRNA.	-0.19	7.13	1.85E-22	8.79E-22	green
CISD1	55847	zinc finger, CDGSH-type domain 1 (ZCD1), mRNA.	-0.06	8.44	5.71E-03	8.34E-03	green
CLIC4	25932	chloride intracellular channel 4 (CLIC4), mRNA.	-0.25	8.26	2.14E-32	1.55E-31	green
CLTC	1213	clathrin, heavy chain (Hc) (CLTC), mRNA.	-0.05	7.94	2.17E-03	3.31E-03	green
CMPK1	51727	cytidylate kinase (CMPK), mRNA.	-0.06	7.56	3.25E-05	5.92E-05	green
COMMD10	51397	COMM domain containing 10 (COMMD10), mRNA.	0.06	8.00	1.32E-02	1.85E-02	green

COPS2	9318	COP9 constitutive photomorphogenic homolog subunit 2 (Arabidopsis) (COPS2), mRNA.	0.46	8.62	5.44E-63	1.77E-61	green
CTNNA1	1495	catenin (cadherin-associated protein), alpha 1, 102kDa (CTNNA1), mRNA.	-0.01	5.57	6.08E-01	6.49E-01	green
CTSO	1519	cathepsin O (CTSO), mRNA.	-0.03	7.49	2.19E-02	2.99E-02	green
CTTN	2017	cortactin (CTTN), transcript variant 1, mRNA.	-0.05	8.43	1.44E-04	2.47E-04	green
CWF19L2	143884	CWF19-like 2, cell cycle control (S. pombe) (CWF19L2), mRNA.	-0.19	7.70	1.13E-36	9.80E-36	green
CYB5R4	51167	cytochrome b5 reductase 4 (CYB5R4), mRNA.	0.13	9.86	5.46E-20	2.34E-19	green
CYP51A1	1595	cytochrome P450, family 51, subfamily A, polypeptide 1 (CYP51A1), mRNA.	-0.29	6.52	5.95E-42	6.59E-41	green
DCAF13	25879	WD repeats and SOF1 domain containing (WDSOF1), mRNA.	-0.07	9.11	1.35E-04	2.31E-04	green
DCP2	167227	DCP2 decapping enzyme homolog (S. cerevisiae) (DCP2), mRNA.	0.26	6.52	3.72E-35	3.00E-34	green
DCTN4	51164	dynactin 4 (p62) (DCTN4), mRNA.	0.06	6.68	7.42E-05	1.31E-04	green
DDX3Y	8653	DEAD (Asp-Glu-Ala-Asp) box polypeptide 3, Y-linked (DDX3Y), mRNA.	0.06	7.77	3.33E-06	6.56E-06	green
DENND6A	201627	hypothetical protein FLJ34969 (FLJ34969), mRNA.	0.09	7.85	2.67E-10	6.83E-10	green

DENND6B	414918	family with sequence similarity 116, member B (FAM116B), mRNA.	-0.41	9.01	7.31E-54	1.35E-52	green
DLAT	1737	dihydrolipoamide S-acetyltransferase (E2 component of pyruvate dehydrogenase complex) (DLAT), mRNA.	-0.07	9.37	1.34E-05	2.53E-05	green
DLD	1738	dihydrolipoamide dehydrogenase (DLD), mRNA.	0.60	5.80	4.57E-42	5.12E-41	green
DMXL1	1657	Dmx-like 1 (DMXL1), mRNA.	0.43	6.36	1.35E-43	1.61E-42	green
DNAAF2	55172	chromosome 14 open reading frame 104 (C14orf104), mRNA.	0.03	9.34	2.25E-02	3.07E-02	green
DNAJA2	10294	DnaJ (Hsp40) homolog, subfamily A, member 2 (DNAJA2), mRNA.	0.04	7.93	2.44E-02	3.32E-02	green
DNAJB14	79982	DnaJ (Hsp40) homolog, subfamily B, member 14 (DNAJB14), transcript variant 2, mRNA.	0.66	6.71	5.52E-78	4.12E-76	green
DNM1L	10059	dynamin 1-like (DNM1L), transcript variant 3, mRNA.	0.07	7.37	1.40E-07	3.03E-07	green
DOCK10	55619	dedicator of cytokinesis 10 (DOCK10), mRNA.	-0.14	6.23	9.50E-14	2.94E-13	green
DOCK8	81704	dedicator of cytokinesis 8 (DOCK8), mRNA.	0.04	8.40	2.00E-01	2.37E-01	green
DPM2	8818	dolichyl-phosphate mannosyltransferase polypeptide 2, regulatory subunit (DPM2), mRNA.	-0.09	5.44	3.36E-06	6.61E-06	green

DR1	1810	down-regulator of transcription 1, TBP-binding (negative cofactor 2) (DR1), mRNA.	-0.04	10.53	3.76E-03	5.60E-03	green
EEF2KMT	196483	family with sequence similarity 86, member A (FAM86A), transcript variant 1, mRNA.	-0.03	6.89	9.36E-02	1.18E-01	green
EIF1AX	1964	eukaryotic translation initiation factor 1A, X-linked (EIF1AX), mRNA.	-0.04	8.32	1.74E-03	2.68E-03	green
EIF3A	8661	eukaryotic translation initiation factor 3, subunit 10 theta, 150/170kDa (EIF3S10), mRNA.	-0.09	10.98	8.18E-12	2.27E-11	green
EIF3F	83880	IFP38 (IFP38), mRNA.	-0.14	9.08	1.50E-13	4.58E-13	green
EIF5B	9669	eukaryotic translation initiation factor 5B (EIF5B), mRNA.	0.05	5.64	2.17E-03	3.31E-03	green
ELF4	2000	E74-like factor 4 (ets domain transcription factor) (ELF4), mRNA.	1.02	6.20	5.07E-87	7.57E-85	green
ELOVL5	60481	ELOVL family member 5, elongation of long chain fatty acids (FEN1/Elo2, SUR4/Elo3-like, yeast) (ELOVL5), mRNA.	-0.08	10.71	1.19E-10	3.10E-10	green
EML4	27436	echinoderm microtubule associated protein like 4 (EML4), mRNA.	-0.08	8.86	5.72E-09	1.36E-08	green
ENOSF1	55556	enolase superfamily member 1 (ENOSF1), mRNA.	-0.01	9.78	5.66E-01	6.09E-01	green
ERGIC2	51290	ERGIC and golgi 2 (ERGIC2), mRNA.	-0.12	9.52	1.65E-10	4.26E-10	green

ESCO2	157570	establishment of cohesion 1 homolog 2 (S. cerevisiae) (ESCO2), mRNA.	0.02	6.98	2.72E-01	3.14E-01	green
EVI2A	2123	ecotropic viral integration site 2A (EVI2A), transcript variant 2, mRNA.	0.07	7.56	5.11E-06	9.92E-06	green
FABP5	387934	PREDICTED: similar to Fatty acid-binding protein, epidermal (E-FABP) (Psoriasis-associated fatty acid-binding protein homolog) (PA-FABP) (LOC387934), mRNA.	-0.11	11.40	5.47E-16	1.91E-15	green
FAM117B	150864	amyotrophic lateral sclerosis 2 (juvenile) chromosome region, candidate 13 (ALS2CR13), mRNA.	-0.06	7.29	4.64E-05	8.31E-05	green
FAM122B	159090	family with sequence similarity 122B (FAM122B), mRNA.	-0.02	8.98	2.13E-01	2.52E-01	green
FAM162A	26355	chromosome 3 open reading frame 28 (C3orf28), mRNA.	-0.01	6.59	5.63E-01	6.06E-01	green
FAM49B	51571	family with sequence similarity 49, member B (FAM49B), mRNA.	0.01	8.66	7.48E-01	7.78E-01	green
FAM98A	25940	family with sequence similarity 98, member A (FAM98A), mRNA.	-0.07	8.97	1.01E-05	1.92E-05	green
FANCI	55215	KIAA1794 (KIAA1794), mRNA.	-0.52	8.22	1.64E-63	5.53E-62	green

FDFT1	2222	farnesyl-diphosphate farnesyltransferase 1 (FDFT1), mRNA.	0.06	9.22	6.20E-05	1.10E-04	green
FH	2271	fumarate hydratase (FH), nuclear gene encoding mitochondrial protein, mRNA.	-0.10	7.74	8.57E-09	2.00E-08	green
FLI1	2313	Friend leukemia virus integration 1 (FLI1), mRNA.	0.31	7.14	2.07E-40	2.13E-39	green
FRG1	2483	FSHD region gene 1 (FRG1), mRNA.	-0.06	7.91	7.39E-06	1.42E-05	green
FYTTD1	84248	forty-two-three domain containing 1 (FYTTD1), transcript variant 2, mRNA.	0.20	8.32	1.57E-34	1.23E-33	green
G6PC3	92579	glucose 6 phosphatase, catalytic, 3 (G6PC3), mRNA.	-0.01	6.94	6.58E-01	6.96E-01	green
GBAS	2631	glioblastoma amplified sequence (GBAS), mRNA.	0.02	9.50	7.82E-02	9.94E-02	green
GDI2	2665	GDP dissociation inhibitor 2 (GDI2), mRNA.	0.05	8.16	1.38E-03	2.16E-03	green
GLRX2	51022	glutaredoxin 2 (GLRX2), transcript variant 2, mRNA.	-0.13	7.90	2.50E-24	1.31E-23	green
GMNN	51053	geminin, DNA replication inhibitor (GMNN), mRNA.	0.11	7.52	1.33E-08	3.07E-08	green
GOLT1B	51026	golgi transport 1 homolog B (S. cerevisiae) (GOLT1B), mRNA.	-0.04	10.19	1.55E-02	2.15E-02	green
GTF2H1	2965	general transcription factor IIH, polypeptide 1 (62kD subunit) (GTF2H1), mRNA.	-0.07	6.91	3.35E-07	7.05E-07	green

GTF3C6	644563	PREDICTED: hypothetical LOC644563 (LOC644563), mRNA.	-0.08	10.94	7.26E-10	1.81E-09	green
HDAC2	3066	histone deacetylase 2 (HDAC2), mRNA.	0.03	7.18	1.53E-02	2.13E-02	green
HLA-DQB1	3119	major histocompatibility complex, class II, DQ beta 1 (HLA-DQB1), mRNA.	-0.23	7.44	1.12E-47	1.66E-46	green
HLA-G	3135	HLA-G histocompatibility antigen, class I, G (HLA-G), mRNA.	-0.44	7.24	7.95E-59	2.10E-57	green
HLTF	6596	helicase-like transcription factor (HLTF), transcript variant 2, mRNA.	-0.18	7.42	9.87E-31	6.54E-30	green
HMGB1	731809	PREDICTED: similar to High mobility group protein B1 (High mobility group protein 1) (HMG-1) (Amphoterin) (Heparin-binding protein p30) (LOC731809), mRNA.	-0.02	6.60	2.41E-01	2.81E-01	green
HMGB1	10357	high-mobility group (nonhistone chromosomal) protein 1-like 1 (HMG1L1), mRNA.	0.01	6.55	7.53E-01	7.83E-01	green
HMGN3	9324	high mobility group nucleosomal binding domain 3 (HMGN3), transcript variant 2, mRNA.	0.08	6.21	3.17E-06	6.24E-06	green

HNRNPH3	3189	heterogeneous nuclear ribonucleoprotein H3 (2H9) (HNRPH3), transcript variant 2H9, mRNA.	1.09	5.94	5.31E-68	2.16E-66	green
HNRNPL	3191	heterogeneous nuclear ribonucleoprotein L (HNRPL), transcript variant 1, mRNA.	-0.14	11.14	2.73E-25	1.50E-24	green
HSPA4	3308	heat shock 70kDa protein 4 (HSPA4), transcript variant 1, mRNA.	-0.06	8.88	7.90E-06	1.51E-05	green
HSPB11	51668	chromosome 1 open reading frame 41 (C1orf41), mRNA.	-0.66	6.06	5.95E-63	1.92E-61	green
IBTK	25998	inhibitor of Bruton agammaglobulinemia tyrosine kinase (IBTK), mRNA.	-0.18	9.24	1.44E-28	8.91E-28	green
IER3IP1	51124	immediate early response 3 interacting protein 1 (IER3IP1), mRNA.	-0.04	8.96	8.38E-03	1.20E-02	green
IFT74	80173	intraflagellar transport 74 homolog (Chlamydomonas) (IFT74), mRNA.	0.03	7.91	6.66E-02	8.55E-02	green
IMPA1	3612	inositol(myo)-1(or 4)-monophosphatase 1 (IMPA1), mRNA.	0.08	8.45	3.02E-03	4.54E-03	green
INTS8	55656	integrator complex subunit 8 (INTS8), mRNA.	-0.05	5.65	3.41E-03	5.10E-03	green
ITGB3BP	23421	integrin beta 3 binding protein (beta3-endonexin) (ITGB3BP), mRNA.	-0.15	8.30	2.09E-17	7.89E-17	green

KATNA1	11104	katanin p60 (ATPase-containing) subunit A 1 (KATNA1), mRNA.	0.67	8.68	9.57E-69	4.01E-67	green
KBTBD8	84541	kelch repeat and BTB (POZ) domain containing 8 (KBTBD8), mRNA.	0.11	6.35	1.65E-09	4.02E-09	green
KDM3A	55818	jumonji domain containing 1A (JMJD1A), mRNA.	0.07	9.81	3.83E-08	8.56E-08	green
KHDRBS1	10657	KH domain containing, RNA binding, signal transduction associated 1 (KHDRBS1), mRNA.	-0.40	6.45	1.75E-70	8.09E-69	green
KIAA1033	23325	KIAA1033 (KIAA1033), mRNA.	-0.03	9.50	1.13E-01	1.41E-01	green
KIAA1524	57650	KIAA1524 (KIAA1524), mRNA.	-0.08	7.11	7.28E-07	1.49E-06	green
KLC1	3831	kinesin 2 (KNS2), transcript variant 1, mRNA.	0.55	7.57	1.95E-69	8.60E-68	green
KLHL2	11275	kelch-like 2, Mayven (Drosophila) (KLHL2), mRNA.	-0.11	7.76	7.22E-14	2.26E-13	green
KPNA3	3839	karyopherin alpha 3 (importin alpha 4) (KPNA3), mRNA.	-0.10	7.50	9.73E-12	2.70E-11	green
KPNA4	3840	karyopherin alpha 4 (importin alpha 3) (KPNA4), mRNA.	-0.25	9.06	6.99E-42	7.69E-41	green
LCLAT1	253558	lysocardiolipin acyltransferase (LYCAT), transcript variant 2, mRNA.	0.09	9.07	6.99E-12	1.95E-11	green
LDHB	3945	lactate dehydrogenase B (LDHB), mRNA.	0.08	6.22	7.01E-07	1.44E-06	green
LMNB1	4001	lamin B1 (LMNB1), mRNA.	-0.25	6.38	1.95E-31	1.34E-30	green

LSM1	27257	LSM1 homolog, U6 small nuclear RNA associated (S. cerevisiae) (LSM1), mRNA.	0.07	6.37	3.65E-05	6.61E-05	green
LSM4	25804	LSM4 homolog, U6 small nuclear RNA associated (S. cerevisiae) (LSM4), mRNA.	0.02	8.64	1.83E-01	2.19E-01	green
LSM6	11157	LSM6 homolog, U6 small nuclear RNA associated (S. cerevisiae) (LSM6), mRNA.	0.24	6.03	5.98E-29	3.74E-28	green
MATR3	9782	matrin 3 (MATR3), transcript variant 2, mRNA.	0.00	8.90	9.59E-01	9.65E-01	green
MBNL1	4154	muscleblind-like (Drosophila) (MBNL1), transcript variant 3, mRNA.	-0.01	6.39	6.71E-01	7.08E-01	green
MCTS1	28985	malignant T cell amplified sequence 1 (MCTS1), mRNA.	-0.04	6.65	9.12E-04	1.45E-03	green
MED1	5469	PPAR binding protein (PPARBP), mRNA.	-0.08	7.08	2.70E-08	6.11E-08	green
MED23	9439	cofactor required for Sp1 transcriptional activation, subunit 3, 130kDa (CRSP3), transcript variant 1, mRNA.	-0.16	6.57	1.80E-18	7.13E-18	green
MEX3C	51320	ring finger and KH domain containing 2 (RKHD2), mRNA.	-0.64	6.33	9.31E-56	1.94E-54	green
MICU2	221154	EF-hand domain family, member A1 (EFHA1), mRNA.	0.08	10.31	2.90E-05	5.31E-05	green
MINPP1	9562	multiple inositol polyphosphate histidine phosphatase, 1 (MINPP1), mRNA.	-0.34	8.61	1.61E-41	1.73E-40	green

MND1	84057	meiotic nuclear divisions 1 homolog (S. cerevisiae) (MND1), mRNA.	-0.23	7.78	2.76E-32	1.98E-31	green
MOB1B	92597	MOB1, Mps One Binder kinase activator-like 1A (yeast) (MOBKL1A), mRNA.	0.02	7.92	2.79E-01	3.21E-01	green
MORF4L1	10933	mortality factor 4 like 1 (MORF4L1), transcript variant 1, mRNA.	0.04	6.74	4.62E-03	6.83E-03	green
MRPL47	57129	mitochondrial ribosomal protein L47 (MRPL47), nuclear gene encoding mitochondrial protein, transcript variant 1, mRNA.	-0.16	8.96	9.22E-21	4.08E-20	green
MRPL51	51258	mitochondrial ribosomal protein L51 (MRPL51), nuclear gene encoding mitochondrial protein, mRNA.	0.06	7.18	1.91E-04	3.24E-04	green
MRPS28	28957	mitochondrial ribosomal protein S28 (MRPS28), nuclear gene encoding mitochondrial protein, mRNA.	-0.09	8.77	5.78E-09	1.37E-08	green
MSH2	4436	mutS homolog 2, colon cancer, nonpolyposis type 1 (E. coli) (MSH2), mRNA.	0.09	6.61	2.00E-05	3.71E-05	green
MTF2	22823	metal response element binding transcription factor 2 (MTF2), mRNA.	0.05	9.05	5.14E-06	9.96E-06	green

MTMR6	9107	myotubularin related protein 6 (MTMR6), mRNA.	-0.11	6.19	3.54E-09	8.50E-09	green
NA	221960	chromosome 7 open reading frame 28B (C7orf28B), mRNA.	-0.25	8.06	1.26E-50	2.01E-49	green
NA	278	amylase, alpha 1C; salivary (AMY1C), mRNA.	0.36	6.41	2.89E-44	3.56E-43	green
NA	51119	Shwachman-Bodian-Diamond syndrome (SBDS), mRNA.	-0.21	7.72	1.07E-25	5.97E-25	green
NA	6880	TAF9 RNA polymerase II, TATA box binding protein (TBP)-associated factor, 32kDa (TAF9), transcript variant 3, mRNA.	0.04	8.94	7.83E-02	9.94E-02	green
NARS	4677	asparaginyl-tRNA synthetase (NARS), mRNA.	0.08	6.26	3.40E-06	6.68E-06	green
NDC80	10403	NDC80 homolog, kinetochore complex component (S. cerevisiae) (NDC80), mRNA.	0.18	11.13	3.24E-42	3.65E-41	green
NDUFAF5	79133	chromosome 20 open reading frame 7 (C20orf7), transcript variant 2, mRNA.	0.00	10.31	9.06E-01	9.21E-01	green
NFE2L2	4780	nuclear factor (erythroid-derived 2)-like 2 (NFE2L2), mRNA.	0.02	6.25	2.33E-01	2.73E-01	green
NIN	51199	ninein (GSK3B interacting protein) (NIN), transcript variant 2, mRNA.	-0.07	9.84	1.34E-07	2.90E-07	green
NMD3	51068	NMD3 homolog (S. cerevisiae) (NMD3), mRNA.	0.08	6.71	4.78E-07	9.93E-07	green

NPTN	27020	neuroplastin (NPTN), transcript variant beta, mRNA.	0.02	8.53	2.72E-01	3.15E-01	green
NQO2	4835	NAD(P)H dehydrogenase, quinone 2 (NQO2), mRNA.	0.09	9.71	1.89E-10	4.89E-10	green
NUCKS1	64710	nuclear casein kinase and cyclin-dependent kinase substrate 1 (NUCKS1), mRNA.	-0.17	7.53	2.43E-24	1.27E-23	green
NUDCD2	134492	NudC domain containing 2 (NUDCD2), mRNA.	0.07	8.03	8.40E-04	1.34E-03	green
NUF2	83540	NUF2, NDC80 kinetochore complex component, homolog (S. cerevisiae) (NUF2), transcript variant 1, mRNA.	-0.54	7.68	2.19E-54	4.22E-53	green
NUP54	53371	nucleoporin 54kDa (NUP54), mRNA.	-0.03	7.93	2.88E-02	3.86E-02	green
NXT2	55916	nuclear transport factor 2-like export factor 2 (NXT2), mRNA.	-0.03	6.35	1.41E-01	1.71E-01	green
OFD1	8481	oral-facial-digital syndrome 1 (OFD1), mRNA.	-0.02	8.13	1.46E-01	1.77E-01	green
OGFOD1	55239	2-oxoglutarate and iron-dependent oxygenase domain containing 1 (OGFOD1), mRNA.	-0.17	9.76	8.83E-27	5.14E-26	green
ORC3	23595	origin recognition complex, subunit 3-like (yeast) (ORC3L), transcript variant 1, mRNA.	-0.02	7.95	1.39E-01	1.69E-01	green
OSBPL8	114882	oxysterol binding protein-like 8 (OSBPL8), transcript variant 2, mRNA.	-0.03	7.18	1.17E-01	1.45E-01	green

PAPOLA	10914	poly(A) polymerase alpha (PAPOLA), mRNA.	-0.01	7.34	4.02E-01	4.47E-01	green
PARPBP	55010	chromosome 12 open reading frame 48 (C12orf48), mRNA.	0.07	8.37	4.27E-07	8.93E-07	green
PBDC1	51260	chromosome X open reading frame 26 (CXorf26), mRNA.	-0.04	7.60	1.66E-02	2.30E-02	green
PCM1	5108	pericentriolar material 1 (PCM1), mRNA.	0.15	10.83	8.18E-19	3.29E-18	green
PCNA	5111	proliferating cell nuclear antigen (PCNA), transcript variant 1, mRNA.	-0.18	5.68	7.51E-17	2.76E-16	green
PEX3	8504	peroxisomal biogenesis factor 3 (PEX3), mRNA.	-0.14	7.63	7.91E-14	2.46E-13	green
PHIP	55023	pleckstrin homology domain interacting protein (PHIP), mRNA.	-0.02	6.33	1.97E-01	2.34E-01	green
PHTF2	57157	putative homeodomain transcription factor 2 (PHTF2), mRNA.	-0.14	7.49	2.69E-15	9.04E-15	green
PIK3AP1	118788	phosphoinositide-3-kinase adaptor protein 1 (PIK3AP1), mRNA.	0.62	9.97	7.98E-81	7.64E-79	green
PKIA	5569	protein kinase (cAMP-dependent, catalytic) inhibitor alpha (PKIA), transcript variant 6, mRNA.	-0.05	8.46	6.57E-03	9.55E-03	green
PM20D2	135293	aminoacylase 1-like 2 (ACY1L2), mRNA.	-0.03	7.41	1.16E-02	1.64E-02	green
PMS1	5378	PMS1 postmeiotic segregation increased 1 (S. cerevisiae) (PMS1), mRNA.	-0.11	6.26	2.50E-14	7.99E-14	green

PNKP	11284	polynucleotide kinase 3'-phosphatase (PNKP), mRNA.	-0.13	7.84	7.25E-17	2.66E-16	green
POC1B	282809	WD repeat domain 51B (WDR51B), mRNA.	-0.19	8.57	7.36E-38	6.84E-37	green
PPA2	27068	pyrophosphatase (inorganic) 2 (PPA2), nuclear gene encoding mitochondrial protein, transcript variant 1, mRNA.	0.20	6.47	2.36E-20	1.02E-19	green
PPIL1	51645	peptidylprolyl isomerase (cyclophilin)-like 1 (PPIL1), mRNA.	-0.04	6.38	1.80E-02	2.47E-02	green
PPT1	5538	palmitoyl-protein thioesterase 1 (ceroid-lipofuscinosis, neuronal 1, infantile) (PPT1), mRNA.	0.20	8.16	4.30E-36	3.61E-35	green
PRDX2	7001	peroxiredoxin 2 (PRDX2), nuclear gene encoding mitochondrial protein, transcript variant 3, mRNA.	-0.03	6.73	1.54E-02	2.14E-02	green
PRKRA	8575	protein kinase, interferon-inducible double stranded RNA dependent activator (PRKRA), mRNA.	-0.04	7.18	2.71E-03	4.09E-03	green
PSIP1	11168	PC4 and SFRS1 interacting protein 1 (PSIP1), transcript variant 2, mRNA.	-0.02	10.03	1.97E-01	2.34E-01	green
PSMA6	5687	proteasome (prosome, macropain) subunit, alpha type, 6 (PSMA6), mRNA.	0.18	5.42	5.71E-13	1.70E-12	green

PSMD14	10213	proteasome (prosome, macropain) 26S subunit, non-ATPase, 14 (PSMD14), mRNA.	0.06	9.40	1.99E-05	3.68E-05	green
PSMG1	8624	Down syndrome critical region gene 2 (DSCR2), transcript variant 2, mRNA.	0.30	8.21	2.90E-53	5.16E-52	green
PTGES3	10728	prostaglandin E synthase 3 (cytosolic) (PTGES3), mRNA.	0.06	7.38	9.96E-04	1.58E-03	green
PTMA	5757	prothymosin, alpha (gene sequence 28) (PTMA), mRNA.	0.04	8.73	3.76E-03	5.60E-03	green
PUS7	54517	pseudouridylate synthase 7 homolog (S. cerevisiae) (PUS7), mRNA.	-0.03	6.94	8.43E-02	1.07E-01	green
RAB33B	83452	RAB33B, member RAS oncogene family (RAB33B), mRNA.	0.08	9.17	6.25E-07	1.29E-06	green
RABGAP1L	9910	RAB GTPase activating protein 1-like (RABGAP1L), mRNA.	0.11	6.67	1.13E-11	3.12E-11	green
RAP1B	5908	RAP1B, member of RAS oncogene family (RAP1B), transcript variant 2, mRNA.	-0.10	7.20	4.59E-14	1.45E-13	green
RBBP7	5931	retinoblastoma binding protein 7 (RBBP7), mRNA.	0.04	7.87	2.42E-02	3.29E-02	green
RBM34	23029	RNA binding motif protein 34 (RBM34), mRNA.	-0.23	10.11	2.39E-47	3.44E-46	green
RBM7	10179	RNA binding motif protein 7 (RBM7), mRNA.	-0.15	7.96	9.28E-21	4.10E-20	green

REV3L	5980	REV3-like, catalytic subunit of DNA polymerase zeta (yeast) (REV3L), mRNA.	0.11	8.55	9.69E-08	2.12E-07	green
RNASEH2B	79621	ribonuclease H2, subunit B (RNASEH2B), mRNA.	-0.03	5.76	1.18E-01	1.46E-01	green
RNF138	51444	ring finger protein 138 (RNF138), transcript variant 2, mRNA.	0.48	8.44	6.00E-66	2.24E-64	green
RNF219	79596	chromosome 13 open reading frame 7 (C13orf7), mRNA.	0.03	11.79	1.82E-03	2.81E-03	green
RPL23	9349	ribosomal protein L23 (RPL23), mRNA.	0.04	7.04	1.64E-03	2.54E-03	green
RPL27A	6157	ribosomal protein L27a (RPL27A), mRNA.	0.08	9.93	2.31E-09	5.59E-09	green
RPL7	6129	ribosomal protein L7 (RPL7), mRNA.	0.00	7.47	9.68E-01	9.73E-01	green
RPL7A	6130	ribosomal protein L7a (RPL7A), mRNA.	0.06	8.01	5.67E-05	1.01E-04	green
RPLP1	729416	hCG1641617 (LOC729416), mRNA.	-0.13	6.40	3.49E-19	1.43E-18	green
RPS28	6234	ribosomal protein S28 (RPS28), mRNA.	1.04	9.75	3.08E-115	5.21E-112	green
RPS3A	644972	PREDICTED: similar to 40S ribosomal protein S3a (V-fos transformation effector protein) (LOC644972), mRNA.	-0.06	9.07	4.85E-06	9.45E-06	green
RRN3	54700	RRN3 RNA polymerase I transcription factor homolog (S. cerevisiae) (RRN3), mRNA.	0.03	7.90	1.03E-02	1.46E-02	green

RSF1	51773	remodeling and spacing factor 1 (RSF1), mRNA.	0.23	10.75	2.67E-36	2.28E-35	green
RSL24D1	51187	chromosome 15 open reading frame 15 (C15orf15), mRNA.	0.01	8.77	5.58E-01	6.02E-01	green
RSPH1	89765	testis specific A2 homolog (mouse) (TSGA2), mRNA.	0.17	7.38	7.89E-22	3.66E-21	green
S1PR1	1901	endothelial differentiation, sphingolipid G-protein-coupled receptor, 1 (EDG1), mRNA.	0.46	7.53	2.02E-77	1.38E-75	green
SACM1L	22908	SAC1 suppressor of actin mutations 1-like (yeast) (SACM1L), mRNA.	-0.03	7.53	9.86E-03	1.40E-02	green
SAR1A	56681	SAR1 gene homolog A (S. cerevisiae) (SAR1A), mRNA.	-0.41	9.66	4.22E-57	9.60E-56	green
SCOC	60592	short coiled-coil protein (SCOC), mRNA.	0.04	6.69	1.62E-02	2.24E-02	green
SDHD	6392	succinate dehydrogenase complex, subunit D, integral membrane protein (SDHD), nuclear gene encoding mitochondrial protein, mRNA.	0.10	8.44	3.80E-15	1.26E-14	green
SFPQ	6421	splicing factor proline/glutamine-rich (polypyrimidine tract binding protein associated) (SFPQ), mRNA.	-0.03	9.84	2.31E-02	3.15E-02	green
SH3RF1	57630	SH3 domain containing ring finger 1 (SH3RF1), mRNA.	0.33	8.91	8.94E-59	2.32E-57	green

SIRT1	23411	sirtuin (silent mating type information regulation 2 homolog) 1 (S. cerevisiae) (SIRT1), mRNA.	-0.06	7.29	3.79E-05	6.84E-05	green
SKIV2L2	23517	superkiller viralicidic activity 2-like 2 (S. cerevisiae) (SKIV2L2), mRNA.	0.79	10.82	3.19E-93	9.53E-91	green
SLC25A13	10165	solute carrier family 25, member 13 (citrin) (SLC25A13), mRNA.	0.23	6.40	4.31E-37	3.81E-36	green
SLC37A1	54020	solute carrier family 37 (glycerol-3-phosphate transporter), member 1 (SLC37A1), mRNA.	-0.12	8.92	1.60E-20	6.98E-20	green
SLC39A8	64116	solute carrier family 39 (zinc transporter), member 8 (SLC39A8), mRNA.	0.10	8.91	2.07E-16	7.41E-16	green
SLK	9748	STE20-like kinase (yeast) (SLK), mRNA.	0.14	7.81	2.33E-14	7.45E-14	green
SMARCA5	8467	SWI/SNF related, matrix associated, actin dependent regulator of chromatin, subfamily a, member 5 (SMARCA5), mRNA.	0.11	6.78	2.65E-10	6.79E-10	green
SMNDC1	10285	survival motor neuron domain containing 1 (SMNDC1), mRNA.	-0.12	7.98	5.81E-18	2.25E-17	green

SND1	27044	staphylococcal nuclease and tudor domain containing 1 (SND1), mRNA.	-0.05	6.30	1.55E-02	2.15E-02	green
SP3	6670	Sp3 transcription factor (SP3), transcript variant 1, mRNA.	0.05	6.80	2.21E-03	3.37E-03	green
SP4	6671	Sp4 transcription factor (SP4), mRNA.	0.10	8.02	2.07E-08	4.70E-08	green
SPIN4	139886	hypothetical protein LOC139886 (LOC139886), mRNA.	0.12	10.41	7.64E-22	3.55E-21	green
SREK1	140890	splicing factor, arginine/serine-rich 12 (SFRS12), transcript variant 2, mRNA.	0.08	8.24	1.32E-08	3.06E-08	green
SRSF10	727922	PREDICTED: similar to FUS-interacting serine-arginine-rich protein 1 (TLS-associated protein with Ser-Arg repeats) (TLS-associated protein with SR repeats) (TASR) (TLS-associated serine-arginine protein) (TLS-associated SR protein) (Neural-specific SR protein..., transcript variant 5 (LOC727922), mRNA.	0.18	8.99	3.53E-27	2.09E-26	green
SRSF11	9295	splicing factor, arginine/serine-rich 11 (SFRS11), mRNA.	0.35	10.44	5.11E-57	1.15E-55	green
SRSF3	6428	splicing factor, arginine/serine-rich 3 (SFRS3), mRNA.	0.08	9.71	5.62E-08	1.25E-07	green

STAG2	10735	stromal antigen 2 (STAG2), transcript variant 3, mRNA.	-0.02	6.66	1.21E-01	1.49E-01	green
STARD3NL	83930	STARD3 N-terminal like (STARD3NL), mRNA.	-0.04	7.37	7.56E-04	1.21E-03	green
STK26	51765	serine/threonine protein kinase MST4 (MST4), transcript variant 3, mRNA.	0.17	7.46	3.75E-28	2.29E-27	green
SUB1	10923	SUB1 homolog (S. cerevisiae) (SUB1), mRNA.	0.04	9.84	1.23E-05	2.33E-05	green
SUMO2	6613	SMT3 suppressor of mif two 3 homolog 2 (S. cerevisiae) (SUMO2), transcript variant 1, mRNA.	0.05	8.98	2.62E-05	4.80E-05	green
SYAP1	94056	synapse associated protein 1, SAP47 homolog (Drosophila) (SYAP1), mRNA.	-0.17	8.55	3.80E-15	1.26E-14	green
TCERG1	10915	transcription elongation regulator 1 (TCERG1), transcript variant 2, mRNA.	0.14	9.16	3.34E-12	9.50E-12	green
TCF12	6938	transcription factor 12 (HTF4, helix-loop-helix transcription factors 4) (TCF12), transcript variant 1, mRNA.	-0.19	7.96	2.93E-35	2.37E-34	green
TEX30	93081	chromosome 13 open reading frame 27 (C13orf27), mRNA.	-0.18	8.24	5.13E-23	2.53E-22	green
TIMM21	29090	chromosome 18 open reading frame 55 (C18orf55), mRNA.	0.06	7.33	4.54E-04	7.46E-04	green

TIPRL	261726	TIP41, TOR signalling pathway regulator-like (S. cerevisiae) (TIPRL), transcript variant 1, mRNA.	-0.03	5.75	8.90E-02	1.12E-01	green
TLK1	9874	tousled-like kinase 1 (TLK1), mRNA.	0.00	10.03	8.24E-01	8.48E-01	green
TMA16	55319	hypothetical protein FLJ11184 (FLJ11184), mRNA.	0.09	9.26	8.64E-06	1.65E-05	green
TMEM14B	81853	transmembrane protein 14B (TMEM14B), mRNA.	-0.17	8.75	2.01E-21	9.14E-21	green
TMEM167A	153339	transmembrane protein 167 (TMEM167), mRNA.	-0.05	9.15	9.75E-05	1.70E-04	green
TMEM263	90488	chromosome 12 open reading frame 23 (C12orf23), mRNA.	-0.01	7.87	5.95E-01	6.36E-01	green
TPM3	7170	tropomyosin 3 (TPM3), transcript variant 3, mRNA.	0.54	8.53	4.29E-49	6.56E-48	green
TRAF3IP2	10758	TRAF3 interacting protein 2 (TRAF3IP2), transcript variant 1, mRNA.	0.17	10.61	5.56E-14	1.75E-13	green
TRIP12	9320	thyroid hormone receptor interactor 12 (TRIP12), mRNA.	0.08	9.22	1.35E-08	3.12E-08	green
TRIT1	54802	tRNA isopentenyltransferase 1 (TRIT1), mRNA.	-0.05	8.33	6.13E-07	1.27E-06	green
TRMT10C	54931	RNA (guanine-9-) methyltransferase domain containing 1 (RG9MTD1), mRNA.	-0.23	8.10	1.07E-31	7.48E-31	green

TRNT1	51095	tRNA nucleotidyl transferase, CCA-adding, 1 (TRNT1), transcript variant 2, mRNA.	0.58	7.43	3.12E-62	9.79E-61	green
TSPYL5	85453	TSPY-like 5 (TSPYL5), mRNA.	-0.06	6.91	2.40E-05	4.43E-05	green
TSR2	90121	TSR2, 20S rRNA accumulation, homolog (S. cerevisiae) (TSR2), mRNA.	-0.23	7.00	4.59E-34	3.51E-33	green
TTC33	23548	tetratricopeptide repeat domain 33 (TTC33), mRNA.	0.01	9.56	6.62E-01	6.99E-01	green
TUBA4A	7277	tubulin, alpha 4a (TUBA4A), mRNA.	-0.03	7.53	1.35E-01	1.65E-01	green
TUBD1	51174	tubulin, delta 1 (TUBD1), mRNA.	0.05	7.74	5.08E-04	8.31E-04	green
TXNDC9	10190	thioredoxin domain containing 9 (TXNDC9), mRNA.	-0.06	7.14	2.37E-06	4.72E-06	green
TXNL1	9352	thioredoxin-like 1 (TXNL1), mRNA.	-0.06	6.84	2.19E-03	3.33E-03	green
UBA6	55236	ubiquitin-activating enzyme E1-like 2 (UBE1L2), mRNA.	0.23	8.56	1.71E-29	1.08E-28	green
UBE2V2	7336	ubiquitin-conjugating enzyme E2 variant 2 (UBE2V2), mRNA.	-0.03	6.61	2.50E-02	3.38E-02	green
UBQLN2	29978	ubiquilin 2 (UBQLN2), mRNA.	0.19	7.74	2.80E-24	1.46E-23	green
UGP2	7360	UDP-glucose pyrophosphorylase 2 (UGP2), transcript variant 2, mRNA.	-0.15	7.49	4.44E-20	1.91E-19	green
UQCRC2	7385	ubiquinol-cytochrome c reductase core protein II (UQCRC2), mRNA.	0.08	8.90	7.09E-09	1.67E-08	green
UQCRH	7388	ubiquinol-cytochrome c reductase hinge protein (UQCRH), mRNA.	-0.01	10.24	2.59E-01	3.01E-01	green

USP1	7398	ubiquitin specific peptidase 1 (USP1), transcript variant 3, mRNA.	0.14	7.70	1.06E-15	3.66E-15	green
USP33	23032	ubiquitin specific peptidase 33 (USP33), transcript variant 3, mRNA.	0.09	7.64	6.17E-08	1.36E-07	green
UTP11L	51118	UTP11-like, U3 small nucleolar ribonucleoprotein, (yeast) (UTP11L), mRNA.	-0.04	6.39	3.47E-02	4.61E-02	green
UTP18	51096	UTP18, small subunit (SSU) processome component, homolog (yeast) (UTP18), mRNA.	-0.03	7.76	8.96E-02	1.13E-01	green
UTP3	57050	disrupter of silencing 10 (SAS10), mRNA.	0.07	8.29	1.82E-04	3.09E-04	green
VMA21	203547	hypothetical protein LOC203547 (LOC203547), mRNA.	0.16	9.62	4.38E-23	2.17E-22	green
VRK3	51231	vaccinia related kinase 3 (VRK3), transcript variant 1, mRNA.	0.03	6.03	2.84E-02	3.81E-02	green
VWA8	23078	KIAA0564 protein (KIAA0564), transcript variant 1, mRNA.	-0.14	11.47	6.14E-20	2.61E-19	green
WDR48	57599	WD repeat domain 48 (WDR48), mRNA.	-0.04	10.01	6.52E-03	9.49E-03	green
WIPF1	7456	WAS/WASL interacting protein family, member 1 (WIPF1), transcript variant 1, mRNA.	0.19	8.01	2.60E-19	1.07E-18	green
WSB1	26118	WD repeat and SOCS box-containing 1 (WSB1), transcript variant 1, mRNA.	0.32	7.13	9.74E-42	1.06E-40	green

WSB2	55884	WD repeat and SOCS box-containing 2 (WSB2), mRNA.	0.05	8.43	2.58E-04	4.33E-04	green
XPO1	7514	exportin 1 (CRM1 homolog, yeast) (XPO1), mRNA.	0.12	7.06	1.61E-07	3.46E-07	green
YEATS4	8089	YEATS domain containing 4 (YEATS4), mRNA.	-0.01	10.85	4.91E-01	5.36E-01	green
YWHAG	7532	tyrosine 3-monooxygenase/tryptophan 5-monooxygenase activation protein, gamma polypeptide (YWHAG), mRNA.	-0.06	5.62	6.00E-04	9.74E-04	green
ZBTB33	10009	zinc finger and BTB domain containing 33 (ZBTB33), mRNA.	0.28	9.52	2.55E-31	1.74E-30	green
ZFR	51663	zinc finger RNA binding protein (ZFR), mRNA.	0.00	7.53	9.20E-01	9.32E-01	green
ZGRF1	91431	prematurely terminated mRNA decay factor-like (LOC91431), mRNA.	-0.07	8.76	1.35E-08	3.12E-08	green
ZNF274	10782	zinc finger protein 274 (ZNF274), transcript variant ZNF274a, mRNA.	0.00	6.89	9.74E-01	9.79E-01	green
ZNF518B	85460	KIAA1729 protein (KIAA1729), mRNA.	0.18	7.30	3.11E-23	1.55E-22	green
ZNF721	170960	zinc finger protein 721 (ZNF721), mRNA.	-0.01	8.85	6.16E-01	6.57E-01	green
ZNF91	7644	zinc finger protein 91 (ZNF91), mRNA.	-0.01	6.31	5.12E-01	5.57E-01	green
ZNHIT3	9326	zinc finger, HIT type 3 (ZNHIT3), transcript variant 1, mRNA.	0.07	7.90	5.69E-04	9.24E-04	green

MOSC2	54996	MOCO sulphurase C-terminal domain containing 2 (MOSC2), mRNA.	-0.47	5.86	2.35E-40	2.42E-39	greenyellow
SEPT10	151011	septin 10 (SEPT10), transcript variant 2, mRNA.	0.20	6.06	7.16E-18	2.76E-17	greenyellow
ABCG1	9619	ATP-binding cassette, sub-family G (WHITE), member 1 (ABCG1), transcript variant 6, mRNA.	0.02	8.33	3.12E-01	3.57E-01	greenyellow
ABLIM1	3983	actin binding LIM protein 1 (ABLIM1), transcript variant 4, mRNA.	0.02	9.32	1.50E-01	1.82E-01	greenyellow
ACSS2	55902	acyl-CoA synthetase short-chain family member 2 (ACSS2), transcript variant 1, mRNA.	-0.12	6.99	4.96E-10	1.25E-09	greenyellow
ADAM23	8745	ADAM metallopeptidase domain 23 (ADAM23), mRNA.	0.02	9.55	1.44E-01	1.75E-01	greenyellow
ADARB1	104	adenosine deaminase, RNA-specific, B1 (RED1 homolog rat) (ADARB1), transcript variant 3, mRNA.	-0.19	7.93	4.42E-25	2.41E-24	greenyellow
ADGRE1	2015	egf-like module containing, mucin-like, hormone receptor-like 1 (EMR1), mRNA.	0.03	6.54	9.52E-02	1.19E-01	greenyellow
ADM	133	adrenomedullin (ADM), mRNA.	0.09	6.13	1.55E-11	4.25E-11	greenyellow

AFAP1L2	84632	actin filament associated protein 1-like 2 (AFAP1L2), transcript variant 1, mRNA.	-0.09	6.99	2.19E-06	4.37E-06	greenyellow
ALPK2	115701	heart alpha-kinase (HAK), mRNA.	0.00	9.82	8.39E-01	8.63E-01	greenyellow
ANXA1	301	annexin A1 (ANXA1), mRNA.	-0.20	8.14	1.11E-23	5.65E-23	greenyellow
ARHGAP25	9938	Rho GTPase activating protein 25 (ARHGAP25), transcript variant 1, mRNA.	0.03	7.91	7.20E-02	9.20E-02	greenyellow
ARHGAP44	9912	KIAA0672 gene product (KIAA0672), mRNA.	-0.10	6.83	2.77E-09	6.67E-09	greenyellow
ATG4C	84938	ATG4 autophagy related 4 homolog C (S. cerevisiae) (ATG4C), transcript variant 8, mRNA.	-0.11	9.48	2.55E-14	8.14E-14	greenyellow
ATP6V1G1	9550	ATPase, H+ transporting, lysosomal 13kDa, V1 subunit G1 (ATP6V1G1), mRNA.	-0.02	7.96	1.37E-01	1.67E-01	greenyellow
AUTS2	26053	autism susceptibility candidate 2 (AUTS2), mRNA.	-0.06	7.66	4.92E-04	8.07E-04	greenyellow
BAG3	9531	BCL2-associated athanogene 3 (BAG3), mRNA.	-0.81	6.54	4.22E-52	7.20E-51	greenyellow
BANK1	55024	B-cell scaffold protein with ankyrin repeats 1 (BANK1), mRNA.	0.41	5.72	9.75E-47	1.37E-45	greenyellow
BASP1	10409	brain abundant, membrane attached signal protein 1 (BASP1), mRNA.	1.16	8.03	1.31E-89	2.56E-87	greenyellow
BCAT1	586	branched chain aminotransferase 1, cytosolic (BCAT1), mRNA.	-0.03	8.09	1.22E-02	1.72E-02	greenyellow

BMP4	652	bone morphogenetic protein 4 (BMP4), transcript variant 2, mRNA.	0.07	9.42	5.94E-05	1.06E-04	greenyellow
BZW2	28969	basic leucine zipper and W2 domains 2 (BZW2), mRNA.	0.17	7.17	1.26E-27	7.63E-27	greenyellow
C11orf80	79703	hypothetical protein FLJ22531 (FLJ22531), mRNA.	0.02	6.14	2.29E-01	2.68E-01	greenyellow
C17orf58	284018	chromosome 17 open reading frame 58 (C17orf58), transcript variant 1, mRNA.	0.12	8.35	2.04E-10	5.26E-10	greenyellow
C1orf115	79762	chromosome 1 open reading frame 115 (C1orf115), mRNA.	0.20	8.24	1.34E-29	8.52E-29	greenyellow
CAMK1	8536	calcium/calmodulin-dependent protein kinase I (CAMK1), mRNA.	0.24	9.94	8.60E-30	5.54E-29	greenyellow
CBFB	865	core-binding factor, beta subunit (CBFB), transcript variant 1, mRNA.	0.03	7.87	2.00E-02	2.74E-02	greenyellow
CBLB	868	Cas-Br-M (murine) ecotropic retroviral transforming sequence b (CBLB), mRNA.	-0.15	7.82	2.52E-21	1.14E-20	greenyellow
CCDC53	51019	coiled-coil domain containing 53 (CCDC53), mRNA.	0.07	6.10	9.47E-10	2.34E-09	greenyellow
CCDC74A	90557	coiled-coil domain containing 74A (CCDC74A), mRNA.	-0.20	8.69	1.27E-26	7.32E-26	greenyellow
CCDC74B	91409	coiled-coil domain containing 74B (CCDC74B), mRNA.	-0.07	9.57	1.03E-06	2.10E-06	greenyellow

CD2BP2	10421	CD2 (cytoplasmic tail) binding protein 2 (CD2BP2), mRNA.	0.01	9.09	6.71E-01	7.08E-01	greenyellow
CD47	961	CD47 molecule (CD47), transcript variant 2, mRNA.	0.03	9.61	2.76E-02	3.71E-02	greenyellow
CLIC6	54102	chloride intracellular channel 6 (CLIC6), mRNA.	-0.13	7.06	8.69E-15	2.85E-14	greenyellow
CLYBL	171425	citrate lyase beta like (CLYBL), transcript variant 2, mRNA.	0.24	9.38	5.22E-44	6.30E-43	greenyellow
CORO1B	57175	coronin, actin binding protein, 1B (CORO1B), transcript variant 2, mRNA.	0.73	6.96	1.98E-76	1.27E-74	greenyellow
CPA4	51200	carboxypeptidase A4 (CPA4), mRNA.	0.02	6.44	1.34E-01	1.64E-01	greenyellow
CREB5	9586	cAMP responsive element binding protein 5 (CREB5), transcript variant 3, mRNA.	0.05	8.58	2.82E-06	5.58E-06	greenyellow
CREG1	8804	cellular repressor of E1A-stimulated genes 1 (CREG1), mRNA.	0.16	7.08	2.18E-24	1.15E-23	greenyellow
CRY1	1407	cryptochrome 1 (photolyase-like) (CRY1), mRNA.	-0.04	7.73	4.50E-03	6.65E-03	greenyellow
CYSTM1	84418	chromosome 5 open reading frame 32 (C5orf32), mRNA.	0.04	9.40	5.63E-03	8.23E-03	greenyellow
DCBLD2	131566	discoidin, CUB and LCCL domain containing 2 (DCBLD2), mRNA.	-0.05	9.20	1.01E-05	1.92E-05	greenyellow
DENND2D	79961	DENN/MADD domain containing 2D (DENND2D), mRNA.	-0.12	6.85	4.13E-12	1.17E-11	greenyellow

DENND5A	23258	RAB6 interacting protein 1 (RAB6IP1), mRNA.	-0.02	6.16	1.50E-01	1.82E-01	greenyellow
DNAJA4	55466	DnaJ (Hsp40) homolog, subfamily A, member 4 (DNAJA4), mRNA.	0.03	6.14	6.39E-02	8.22E-02	greenyellow
DSC2	1824	desmocollin 2 (DSC2), transcript variant Dsc2b, mRNA.	-0.03	7.56	1.13E-01	1.41E-01	greenyellow
DSG2	1829	desmoglein 2 (DSG2), mRNA.	0.10	10.39	5.98E-13	1.78E-12	greenyellow
DUSP23	54935	dual specificity phosphatase 23 (DUSP23), mRNA.	0.33	10.08	7.11E-53	1.24E-51	greenyellow
DYNLT1	6993	dynein, light chain, Tctex-type 1 (DYNLT1), mRNA.	-0.21	8.33	4.40E-26	2.49E-25	greenyellow
ECHDC3	79746	enoyl Coenzyme A hydratase domain containing 3 (ECHDC3), mRNA.	0.16	6.72	2.91E-28	1.79E-27	greenyellow
FAH	2184	fumarylacetoacetate hydrolase (fumarylacetoacetase) (FAH), mRNA.	0.03	6.74	1.05E-01	1.31E-01	greenyellow
FAM101B	359845	family with sequence similarity 101, member B (FAM101B), mRNA.	-0.08	8.12	5.52E-10	1.38E-09	greenyellow
FAM167A	83648	chromosome 8 open reading frame 13 (C8orf13), mRNA.	-0.16	8.42	9.96E-21	4.39E-20	greenyellow
FAM43A	131583	family with sequence similarity 43, member A (FAM43A), mRNA.	0.08	9.80	2.07E-12	5.94E-12	greenyellow
FES	2242	feline sarcoma oncogene (FES), mRNA.	-0.12	8.91	6.34E-17	2.34E-16	greenyellow
FHOD3	80206	formin homology 2 domain containing 3 (FHOD3), mRNA.	0.05	5.89	5.40E-03	7.91E-03	greenyellow

FRMD4A	55691	FERM domain containing 4A (FRMD4A), mRNA.	-0.23	6.57	6.52E-26	3.66E-25	greenyellow
GAP43	2596	growth associated protein 43 (GAP43), mRNA.	0.03	9.85	3.09E-01	3.53E-01	greenyellow
GCSAM	257144	germinal center expressed transcript 2 (GCET2), transcript variant 1, mRNA.	-0.19	9.50	1.10E-37	1.01E-36	greenyellow
GIMAP1	170575	GTPase, IMAP family member 1 (GIMAP1), mRNA.	-0.05	8.63	2.24E-03	3.40E-03	greenyellow
GIMAP2	26157	GTPase, IMAP family member 2 (GIMAP2), mRNA.	-0.15	5.72	1.79E-19	7.46E-19	greenyellow
GIMAP4	55303	GTPase, IMAP family member 4 (GIMAP4), mRNA.	-0.05	7.01	3.96E-04	6.54E-04	greenyellow
GIMAP6	474344	GTPase, IMAP family member 6 (GIMAP6), transcript variant 1, mRNA.	0.70	6.08	1.12E-58	2.83E-57	greenyellow
GIMAP7	168537	GTPase, IMAP family member 7 (GIMAP7), mRNA.	-0.03	7.86	6.07E-02	7.83E-02	greenyellow
GLB1L2	89944	hypothetical protein BC008326 (LOC89944), mRNA.	0.02	7.82	2.82E-01	3.25E-01	greenyellow
GPD1L	23171	glycerol-3-phosphate dehydrogenase 1-like (GPD1L), mRNA.	-0.17	6.27	7.23E-17	2.66E-16	greenyellow
GPR18	2841	G protein-coupled receptor 18 (GPR18), mRNA.	-0.02	7.10	9.39E-02	1.18E-01	greenyellow
GPR183	1880	Epstein-Barr virus induced gene 2 (lymphocyte-specific G protein-coupled receptor) (EBI2), mRNA.	0.01	7.85	4.18E-01	4.64E-01	greenyellow

GRAP2	9402	GRB2-related adaptor protein 2 (GRAP2), mRNA.	0.03	7.77	7.30E-02	9.32E-02	greenyellow
HDGFRP3	50810	hepatoma-derived growth factor, related protein 3 (HDGFRP3), mRNA.	-0.01	7.45	1.33E-01	1.63E-01	greenyellow
HECW2	57520	HECT, C2 and WW domain containing E3 ubiquitin protein ligase 2 (HECW2), mRNA.	0.02	11.04	4.95E-02	6.46E-02	greenyellow
HK1	3098	hexokinase 1 (HK1), nuclear gene encoding mitochondrial protein, transcript variant 1, mRNA.	0.09	7.85	6.12E-07	1.27E-06	greenyellow
HOMER2	9455	homer homolog 2 (Drosophila) (HOMER2), transcript variant 3, mRNA.	0.28	7.60	1.97E-33	1.46E-32	greenyellow
IL18R1	8809	interleukin 18 receptor 1 (IL18R1), mRNA.	0.03	7.52	1.07E-01	1.33E-01	greenyellow
IL18RAP	8807	interleukin 18 receptor accessory protein (IL18RAP), mRNA.	-0.10	8.45	2.76E-08	6.24E-08	greenyellow
ISCU	23479	iron-sulfur cluster scaffold homolog (E. coli) (ISCU), nuclear gene encoding mitochondrial protein, transcript variant 1, mRNA.	-0.07	9.16	9.98E-06	1.90E-05	greenyellow
JUP	3728	junction plakoglobin (JUP), transcript variant 1, mRNA.	-0.07	6.41	5.90E-06	1.14E-05	greenyellow
KDM1A	23028	amine oxidase (flavin containing) domain 2 (AOF2), transcript variant 2, mRNA.	0.00	7.32	9.18E-01	9.30E-01	greenyellow

LCP2	3937	lymphocyte cytosolic protein 2 (SH2 domain containing leukocyte protein of 76kDa) (LCP2), mRNA.	0.07	9.18	6.48E-09	1.53E-08	greenyellow
LGALSL	29094	galectin-related protein (HSPC159), mRNA.	-0.23	5.97	4.80E-31	3.23E-30	greenyellow
LHFPL2	10184	lipoma HMGIC fusion partner-like 2 (LHFPL2), mRNA.	0.00	6.89	8.25E-01	8.49E-01	greenyellow
LIMA1	51474	LIM domain and actin binding 1 (LIMA1), mRNA.	-0.10	8.88	7.16E-13	2.12E-12	greenyellow
LOXL3	84695	lysyl oxidase-like 3 (LOXL3), mRNA.	-0.05	8.67	5.05E-06	9.81E-06	greenyellow
LRIG3	121227	leucine-rich repeats and immunoglobulin-like domains 3 (LRIG3), mRNA.	-0.21	5.72	1.06E-14	3.46E-14	greenyellow
LRRC28	123355	leucine rich repeat containing 28 (LRRC28), mRNA.	0.01	7.16	3.15E-01	3.59E-01	greenyellow
LRRC34	151827	leucine rich repeat containing 34 (LRRC34), mRNA.	0.28	8.24	5.43E-34	4.13E-33	greenyellow
LRRC4C	57689	leucine rich repeat containing 4C (LRRC4C), mRNA.	-0.27	6.90	9.31E-45	1.17E-43	greenyellow
LTA4H	4048	leukotriene A4 hydrolase (LTA4H), mRNA.	0.13	7.31	7.17E-19	2.91E-18	greenyellow
MS4A1	931	membrane-spanning 4-domains, subfamily A, member 1 (MS4A1), transcript variant 1, mRNA.	0.02	6.37	6.35E-02	8.17E-02	greenyellow
NA	389206	coiled-coil domain containing 4 (CCDC4), mRNA.	0.86	6.33	8.54E-59	2.23E-57	greenyellow

NA	7784	zona pellucida glycoprotein 3 (sperm receptor) (ZP3), mRNA.	0.24	9.59	7.58E-45	9.59E-44	greenyellow
NA	55027	hypothetical protein FLJ20718 (FLJ20718), transcript variant 1, mRNA.	0.17	6.27	1.92E-21	8.77E-21	greenyellow
NA	55340	GTPase, IMAP family member 5 (GIMAP5), mRNA.	-0.04	6.22	2.08E-02	2.85E-02	greenyellow
NCKAP1	10787	NCK-associated protein 1 (NCKAP1), transcript variant 1, mRNA.	0.05	8.75	3.23E-04	5.38E-04	greenyellow
NKG7	4818	natural killer cell group 7 sequence (NKG7), mRNA.	-0.18	8.18	9.77E-23	4.76E-22	greenyellow
NPL	80896	N-acetylneuraminate pyruvate lyase (dihydrodipicolinate synthase) (NPL), mRNA.	0.04	6.98	1.13E-01	1.41E-01	greenyellow
NPNT	255743	nephronectin (NPNT), mRNA.	-0.07	5.59	5.87E-06	1.14E-05	greenyellow
NUFIP2	57532	nuclear fragile X mental retardation protein interacting protein 2 (NUFIP2), mRNA.	0.03	6.48	1.13E-02	1.60E-02	greenyellow
NUP62CL	54830	nucleoporin 62kDa C-terminal like (NUP62CL), mRNA.	0.06	6.68	2.10E-03	3.22E-03	greenyellow
OAT	4942	ornithine aminotransferase (gyrate atrophy) (OAT), nuclear gene encoding mitochondrial protein, mRNA.	-0.56	7.74	2.78E-72	1.39E-70	greenyellow
P3H2	55214	leprecan-like 1 (LEPREL1), mRNA.	-0.43	8.26	5.98E-58	1.44E-56	greenyellow

PASK	23178	PAS domain containing serine/threonine kinase (PASK), mRNA.	0.34	10.15	2.02E-38	1.91E-37	greenyellow
PHTF1	10745	putative homeodomain transcription factor 1 (PHTF1), mRNA.	0.01	9.33	5.55E-01	5.98E-01	greenyellow
PIEZO2	63895	family with sequence similarity 38, member B (FAM38B), mRNA.	0.33	6.50	1.23E-40	1.28E-39	greenyellow
PLS3	5358	plastin 3 (T isoform) (PLS3), mRNA.	0.02	9.74	1.46E-01	1.77E-01	greenyellow
POMZP3	22932	POM (POM121 homolog, rat) and ZP3 fusion (POMZP3), transcript variant 1, mRNA.	-0.12	8.53	2.30E-11	6.24E-11	greenyellow
PPARG	5468	peroxisome proliferator-activated receptor gamma (PPARG), transcript variant 3, mRNA.	0.01	9.39	4.49E-01	4.95E-01	greenyellow
PPP2R4	5524	protein phosphatase 2A activator, regulatory subunit 4 (PPP2R4), transcript variant 2, mRNA.	-0.05	9.56	1.13E-06	2.30E-06	greenyellow
PRCP	5547	prolylcarboxypeptidase (angiotensinase C) (PRCP), transcript variant 2, mRNA.	0.04	5.50	1.96E-02	2.69E-02	greenyellow
PRKCH	5583	protein kinase C, eta (PRKCH), mRNA.	-0.02	7.66	9.83E-02	1.23E-01	greenyellow

PRNP	5621	prion protein (p27-30) (Creutzfeldt-Jakob disease, Gerstmann-Strausler-Scheinker syndrome, fatal familial insomnia) (PRNP), transcript variant 3, mRNA.	-0.07	9.19	2.45E-05	4.50E-05	greenyellow
PSMB9	5698	proteasome (prosome, macropain) subunit, beta type, 9 (large multifunctional peptidase 2) (PSMB9), transcript variant 2, mRNA.	0.01	9.32	3.98E-01	4.44E-01	greenyellow
PTK2	5747	PTK2 protein tyrosine kinase 2 (PTK2), transcript variant 2, mRNA.	0.10	6.95	5.04E-10	1.27E-09	greenyellow
PTP4A3	11156	protein tyrosine phosphatase type IVA, member 3 (PTP4A3), transcript variant 2, mRNA.	-0.04	6.76	2.26E-02	3.08E-02	greenyellow
PTPN7	5778	protein tyrosine phosphatase, non-receptor type 7 (PTPN7), transcript variant 1, mRNA.	0.11	8.03	8.74E-10	2.16E-09	greenyellow
PTRF	284119	polymerase I and transcript release factor (PTRF), mRNA.	0.05	10.15	2.43E-03	3.69E-03	greenyellow
PYROXD2	84795	chromosome 10 open reading frame 33 (C10orf33), mRNA.	-0.17	7.60	6.39E-22	2.97E-21	greenyellow
QPCT	25797	glutaminyl-peptide cyclotransferase (glutaminyl cyclase) (QPCT), mRNA.	0.04	9.87	7.54E-05	1.33E-04	greenyellow

RABGEF1	27342	RAB guanine nucleotide exchange factor (GEF) 1 (RABGEF1), mRNA.	0.04	8.99	4.75E-03	6.99E-03	greenyellow
RAI14	26064	retinoic acid induced 14 (RAI14), mRNA.	-0.16	5.80	1.75E-23	8.79E-23	greenyellow
RB1	5925	retinoblastoma 1 (including osteosarcoma) (RB1), mRNA.	-0.13	9.24	7.22E-16	2.51E-15	greenyellow
RCAN1	1827	Down syndrome critical region gene 1 (DSCR1), transcript variant 2, mRNA.	0.00	5.51	9.23E-01	9.35E-01	greenyellow
REXO2	25996	REX2, RNA exonuclease 2 homolog (S. cerevisiae) (REXO2), mRNA.	0.06	6.22	4.73E-04	7.76E-04	greenyellow
RHBDD1	84236	rhomboid domain containing 1 (RHBDD1), mRNA.	-0.16	8.90	6.99E-24	3.59E-23	greenyellow
RIMBP2	23504	RIMS binding protein 2 (RIMBP2), mRNA.	-0.04	8.45	1.07E-03	1.70E-03	greenyellow
RNF149	284996	ring finger protein 149 (RNF149), mRNA.	0.21	9.75	3.31E-40	3.37E-39	greenyellow
RPP25L	138716	chromosome 9 open reading frame 23 (C9orf23), transcript variant 2, mRNA.	0.21	6.69	1.14E-22	5.50E-22	greenyellow
SAV1	60485	salvador homolog 1 (Drosophila) (SAV1), mRNA.	0.02	8.17	2.16E-01	2.55E-01	greenyellow
SDC1	6382	syndecan 1 (SDC1), transcript variant 2, mRNA.	0.00	7.92	9.73E-01	9.77E-01	greenyellow

SEMA6A	57556	sema domain, transmembrane domain (TM), and cytoplasmic domain, (semaphorin) 6A (SEMA6A), mRNA.	-0.09	9.45	3.01E-10	7.66E-10	greenyellow
SERPINB1	1992	serpin peptidase inhibitor, clade B (ovalbumin), member 1 (SERPINB1), mRNA.	-0.23	7.16	1.49E-25	8.29E-25	greenyellow
SERPINB6	5269	serpin peptidase inhibitor, clade B (ovalbumin), member 6 (SERPINB6), mRNA.	-0.08	9.51	9.58E-11	2.50E-10	greenyellow
SGCE	8910	sarcoglycan, epsilon (SGCE), mRNA.	0.23	10.04	1.17E-29	7.51E-29	greenyellow
SIK3	23387	KIAA0999 protein (KIAA0999), mRNA.	-0.08	6.84	3.64E-06	7.14E-06	greenyellow
SLC23A2	9962	solute carrier family 23 (nucleobase transporters), member 2 (SLC23A2), transcript variant 1, mRNA.	0.06	6.87	2.53E-04	4.26E-04	greenyellow
SNTB1	6641	syntrophin, beta 1 (dystrophin-associated protein A1, 59kDa, basic component 1) (SNTB1), mRNA.	-0.02	7.58	2.80E-01	3.23E-01	greenyellow
SOWAHC	65124	ankyrin repeat domain 57 (ANKRD57), mRNA.	0.09	7.88	8.04E-08	1.77E-07	greenyellow
ST7	7982	suppression of tumorigenicity 7 (ST7), transcript variant a, mRNA.	-0.10	9.65	8.59E-14	2.67E-13	greenyellow

STAT4	6775	signal transducer and activator of transcription 4 (STAT4), mRNA.	0.02	9.41	1.45E-01	1.77E-01	greenyellow
SV2B	9899	synaptic vesicle glycoprotein 2B (SV2B), mRNA.	-0.01	7.20	6.37E-01	6.76E-01	greenyellow
SYK	6850	spleen tyrosine kinase (SYK), mRNA.	0.19	9.08	6.38E-24	3.28E-23	greenyellow
SYNJ2	8871	synaptojanin 2 (SYNJ2), mRNA.	-0.05	6.20	8.85E-04	1.41E-03	greenyellow
TANC1	85461	tetratricopeptide repeat, ankyrin repeat and coiled-coil containing 1 (TANC1), mRNA.	-0.03	8.28	3.06E-02	4.09E-02	greenyellow
TBC1D4	9882	TBC1 domain family, member 4 (TBC1D4), mRNA.	-0.15	6.48	5.13E-20	2.20E-19	greenyellow
TBL1X	6907	transducin (beta)-like 1X-linked (TBL1X), mRNA.	0.05	10.16	1.38E-04	2.37E-04	greenyellow
TESC	54997	tescalcin (TESC), mRNA.	0.06	6.73	6.01E-04	9.75E-04	greenyellow
TMEM144	55314	transmembrane protein 144 (TMEM144), mRNA.	0.13	8.65	7.61E-12	2.12E-11	greenyellow
TMX4	56255	thioredoxin domain containing 13 (TXNDC13), mRNA.	-0.11	6.89	1.28E-13	3.95E-13	greenyellow
TNFRSF19	55504	tumor necrosis factor receptor superfamily, member 19 (TNFRSF19), transcript variant 2, mRNA.	0.10	8.73	5.72E-13	1.70E-12	greenyellow
TRAK1	22906	trafficking protein, kinesin binding 1 (TRAK1), transcript variant 2, mRNA.	0.06	7.45	1.50E-03	2.33E-03	greenyellow
TTF2	8458	transcription termination factor, RNA polymerase II (TTF2), mRNA.	0.00	11.61	7.87E-01	8.15E-01	greenyellow

UBAC2	337867	phosphoglycerate dehydrogenase like 1 (PHGDHL1), mRNA.	-0.11	7.07	4.47E-10	1.13E-09	greenyellow
UBQLN1	29979	ubiquilin 1 (UBQLN1), transcript variant 1, mRNA.	-0.12	8.81	8.78E-20	3.71E-19	greenyellow
UST	10090	uronyl-2-sulfotransferase (UST), mRNA.	0.11	7.50	1.29E-14	4.18E-14	greenyellow
VIM	7431	vimentin (VIM), mRNA.	0.03	12.44	1.18E-01	1.46E-01	greenyellow
VRK2	7444	vaccinia related kinase 2 (VRK2), mRNA.	-0.05	7.56	5.71E-04	9.28E-04	greenyellow
WDR59	79726	WD repeat domain 59 (WDR59), mRNA.	-0.01	8.94	3.29E-01	3.73E-01	greenyellow
WDR91	29062	HSPC049 protein (HSPC049), mRNA.	-0.77	8.13	1.45E-85	1.94E-83	greenyellow
ZMIZ1	57178	zinc finger, MIZ-type containing 1 (ZMIZ1), mRNA.	-0.03	5.79	2.83E-02	3.80E-02	greenyellow
ZNF185	7739	zinc finger protein 185 (LIM domain) (ZNF185), mRNA.	-0.01	5.67	5.35E-01	5.80E-01	greenyellow
ZNF608	57507	zinc finger protein 608 (ZNF608), mRNA.	0.12	6.12	1.72E-10	4.44E-10	greenyellow
BEX5	340542	NGFRAP1-like 1 (NGFRAP1L1), mRNA.	0.01	8.21	6.38E-01	6.77E-01	grey
CD163L1	283316	CD163 molecule-like 1 (CD163L1), mRNA.	-0.45	6.32	2.64E-46	3.63E-45	grey
CD55	1604	CD55 molecule, decay accelerating factor for complement (Cromer blood group) (CD55), mRNA.	0.01	9.44	4.56E-01	5.02E-01	grey
DAPK1	1612	death-associated protein kinase 1 (DAPK1), mRNA.	-0.12	7.84	8.66E-12	2.40E-11	grey

DPYSL4	10570	dihydropyrimidinase-like 4 (DPYSL4), mRNA.	0.02	5.52	2.62E-01	3.04E-01	grey
EIF1AY	9086	eukaryotic translation initiation factor 1A, Y-linked (EIF1AY), mRNA.	0.04	9.08	1.75E-03	2.71E-03	grey
EYA2	2139	eyes absent homolog 2 (Drosophila) (EYA2), transcript variant 5, mRNA.	-0.06	8.02	6.48E-07	1.34E-06	grey
FHL1	2273	four and a half LIM domains 1 (FHL1), mRNA.	-0.01	8.00	6.22E-01	6.62E-01	grey
GALE	2582	UDP-galactose-4-epimerase (GALE), transcript variant 1, mRNA.	-0.65	7.23	1.06E-75	6.55E-74	grey
GALNT18	374378	UDP-N-acetyl-alpha-D-galactosamine:polypeptide N-acetylgalactosaminyltransferase-like 4 (GALNTL4), mRNA.	-0.26	7.61	7.43E-31	4.94E-30	grey
GSTM1	2944	glutathione S-transferase M1 (GSTM1), transcript variant 1, mRNA.	0.20	9.33	5.06E-37	4.46E-36	grey
GTSF1	121355	family with sequence similarity 112, member B (FAM112B), mRNA.	0.06	8.01	4.99E-04	8.17E-04	grey
HDHD1	8226	haloacid dehalogenase-like hydrolase domain containing 1A (HDHD1A), mRNA.	-0.13	6.59	2.59E-11	7.02E-11	grey
HLA-DRB5	3127	major histocompatibility complex, class II, DR beta 5 (HLA-DRB5), mRNA.	-0.13	9.37	1.49E-15	5.08E-15	grey

IPO8	10526	importin 8 (IPO8), mRNA.	0.04	7.74	1.16E-03	1.83E-03	grey
ISX	91464	intestine-specific homeobox (ISX), mRNA.	0.00	8.91	8.72E-01	8.92E-01	grey
KDM5D	8284	jumonji, AT rich interactive domain 1D (JARID1D), mRNA.	-0.15	9.68	2.93E-21	1.32E-20	grey
LMCD1	29995	LIM and cysteine-rich domains 1 (LMCD1), mRNA.	-0.05	6.65	1.54E-03	2.39E-03	grey
NA	401233	similar to HIV TAT specific factor 1; cofactor required for Tat activation of HIV-1 transcription (LOC401233), mRNA.	0.27	6.27	2.72E-30	1.77E-29	grey
NA	389833	similar to hypothetical protein MGC27019 (LOC389833), mRNA.	-0.22	7.70	4.15E-29	2.61E-28	grey
NA	283755	hypothetical protein LOC283755 (LOC283755), mRNA.	0.03	6.97	3.61E-02	4.79E-02	grey
NDUFAF1	51103	NADH dehydrogenase (ubiquinone) 1 alpha subcomplex, assembly factor 1 (NDUFAF1), mRNA.	-0.05	8.29	1.43E-03	2.23E-03	grey
NEFH	4744	neurofilament, heavy polypeptide 200kDa (NEFH), mRNA.	-0.10	9.13	1.25E-12	3.65E-12	grey
NKD2	85409	naked cuticle homolog 2 (Drosophila) (NKD2), mRNA.	0.05	7.83	2.47E-06	4.90E-06	grey
NLGN4Y	22829	neuroligin 4, Y-linked (NLGN4Y), mRNA.	0.14	7.30	4.63E-18	1.80E-17	grey

NLRP2	55655	NLR family, pyrin domain containing 2 (NLRP2), mRNA.	-0.32	7.04	1.20E-41	1.29E-40	grey
PIGR	5284	polymeric immunoglobulin receptor (PIGR), mRNA.	0.10	9.36	2.18E-21	9.91E-21	grey
PKHD1L1	93035	polycystic kidney and hepatic disease 1 (autosomal recessive)-like 1 (PKHD1L1), mRNA.	0.11	6.34	4.33E-09	1.03E-08	grey
PLBD1	79887	hypothetical protein FLJ22662 (FLJ22662), mRNA.	-0.07	5.56	1.51E-05	2.84E-05	grey
PTER	9317	phosphotriesterase related (PTER), transcript variant 1, mRNA.	0.02	7.32	3.25E-01	3.70E-01	grey
RAB34	83871	RAB34, member RAS oncogene family (RAB34), mRNA.	0.00	10.83	7.97E-01	8.24E-01	grey
RASIP1	54922	Ras interacting protein 1 (RASIP1), mRNA.	0.20	7.63	4.42E-31	2.98E-30	grey
ROBO1	6091	roundabout, axon guidance receptor, homolog 1 (Drosophila) (ROBO1), transcript variant 1, mRNA.	-0.01	7.84	6.53E-01	6.91E-01	grey
RPS24	6229	ribosomal protein S24 (RPS24), transcript variant 2, mRNA.	-0.03	8.42	1.29E-01	1.58E-01	grey
RPS4Y1	6192	ribosomal protein S4, Y-linked 1 (RPS4Y1), mRNA.	-0.02	7.95	1.25E-01	1.54E-01	grey
SFRP1	6422	secreted frizzled-related protein 1 (SFRP1), mRNA.	-0.04	5.71	5.30E-02	6.89E-02	grey

SLC25A47	283600	chromosome 14 open reading frame 68 (C14orf68), mRNA.	0.01	9.84	5.48E-01	5.92E-01	grey
SPINT2	10653	serine peptidase inhibitor, Kunitz type, 2 (SPINT2), mRNA.	0.02	6.51	4.65E-01	5.11E-01	grey
TBXAS1	6916	thromboxane A synthase 1 (platelet, cytochrome P450, family 5, subfamily A) (TBXAS1), transcript variant TXS-I, mRNA.	-0.24	9.15	3.50E-34	2.69E-33	grey
THNSL1	79896	threonine synthase-like 1 (bacterial) (THNSL1), mRNA.	0.03	6.93	1.22E-01	1.50E-01	grey
TMSB4Y	9087	thymosin, beta 4, Y-linked (TMSB4Y), mRNA.	-0.03	7.06	5.56E-02	7.20E-02	grey
TPM2	7169	tropomyosin 2 (beta) (TPM2), transcript variant 2, mRNA.	0.06	8.98	2.46E-03	3.74E-03	grey
TRAPPC4	51399	trafficking protein particle complex 4 (TRAPPC4), mRNA.	0.00	7.89	9.42E-01	9.50E-01	grey
TXLNGY	246126	chromosome Y open reading frame 15A (CYorf15A), mRNA.	-0.24	9.16	3.71E-34	2.85E-33	grey
TXLNGY	84663	chromosome Y open reading frame 15B (CYorf15B), mRNA.	-0.03	9.87	2.09E-03	3.19E-03	grey
USP9Y	8287	ubiquitin specific peptidase 9, Y-linked (fat facets-like, Drosophila) (USP9Y), mRNA.	0.18	9.04	9.10E-15	2.97E-14	grey

UTY	7404	ubiquitously transcribed tetratricopeptide repeat gene, Y-linked (UTY), transcript variant 3, mRNA.	-0.07	5.48	4.34E-05	7.79E-05	grey
ABCA3	21	ATP-binding cassette, sub-family A (ABC1), member 3 (ABCA3), mRNA.	0.06	7.67	8.41E-05	1.48E-04	grey60
ADTRP	84830	chromosome 6 open reading frame 105 (C6orf105), mRNA.	-0.51	8.41	4.63E-69	2.03E-67	grey60
ARL6IP5	10550	ADP-ribosylation-like factor 6 interacting protein 5 (ARL6IP5), mRNA.	-0.03	6.90	2.26E-02	3.08E-02	grey60
ARPC5	10092	actin related protein 2/3 complex, subunit 5, 16kDa (ARPC5), mRNA.	0.03	5.77	1.70E-02	2.34E-02	grey60
ATP8B2	57198	ATPase, Class I, type 8B, member 2 (ATP8B2), transcript variant 1, mRNA.	0.03	7.01	1.13E-01	1.40E-01	grey60
BHLHE40	8553	basic helix-loop-helix domain containing, class B, 2 (BHLHB2), mRNA.	0.04	7.48	1.74E-02	2.40E-02	grey60
C10orf10	11067	chromosome 10 open reading frame 10 (C10orf10), mRNA.	0.04	10.16	1.49E-02	2.07E-02	grey60
CAPN2	824	calpain 2, (m/II) large subunit (CAPN2), mRNA.	-0.09	6.02	1.55E-09	3.78E-09	grey60
CCL20	6364	chemokine (C-C motif) ligand 20 (CCL20), mRNA.	0.38	7.95	3.38E-58	8.33E-57	grey60
CCR6	1235	chemokine (C-C motif) receptor 6 (CCR6), transcript variant 1, mRNA.	-0.12	9.31	1.40E-18	5.58E-18	grey60

CCR7	1236	chemokine (C-C motif) receptor 7 (CCR7), mRNA.	-0.09	6.68	8.39E-10	2.08E-09	grey60
CD74	972	CD74 molecule, major histocompatibility complex, class II invariant chain (CD74), transcript variant 1, mRNA.	0.07	6.19	1.40E-05	2.63E-05	grey60
CHST7	56548	carbohydrate (N-acetylglucosamine 6-O) sulfotransferase 7 (CHST7), mRNA.	-0.07	8.51	4.16E-06	8.12E-06	grey60
CIITA	4261	class II, major histocompatibility complex, transactivator (CIITA), mRNA.	-0.31	8.18	1.85E-43	2.19E-42	grey60
CMTM3	123920	CKLF-like MARVEL transmembrane domain containing 3 (CMTM3), transcript variant 3, mRNA.	0.10	8.09	1.54E-12	4.49E-12	grey60
CSRNP2	81566	family with sequence similarity 130, member A1 (FAM130A1), mRNA.	0.09	9.02	7.23E-11	1.91E-10	grey60
CTSC	1075	cathepsin C (CTSC), transcript variant 1, mRNA.	0.14	7.13	5.62E-16	1.96E-15	grey60
EGR1	1958	early growth response 1 (EGR1), mRNA.	0.05	6.69	7.32E-03	1.06E-02	grey60
FAM102B	284611	family with sequence similarity 102, member B (FAM102B), mRNA.	0.03	7.82	1.69E-01	2.03E-01	grey60
FAM129A	116496	chromosome 1 open reading frame 24 (C1orf24), transcript variant 2, mRNA.	-0.23	10.73	3.18E-37	2.83E-36	grey60

FAM171A1	221061	chromosome 10 open reading frame 38 (C10orf38), mRNA.	-0.05	10.05	5.58E-04	9.07E-04	grey60
FAM64A	54478	family with sequence similarity 64, member A (FAM64A), mRNA.	0.08	8.31	1.01E-06	2.06E-06	grey60
GCAT	23464	glycine C-acetyltransferase (2-amino-3-ketobutyrate coenzyme A ligase) (GCAT), nuclear gene encoding mitochondrial protein, mRNA.	-0.07	9.69	1.07E-07	2.34E-07	grey60
GLTP	51228	glycolipid transfer protein (GLTP), mRNA.	-0.04	5.43	1.64E-02	2.27E-02	grey60
H2AFJ	55766	H2A histone family, member J (H2AFJ), transcript variant 2, mRNA.	0.11	8.31	1.13E-11	3.12E-11	grey60
HIST1H1C	3006	histone cluster 1, H1c (HIST1H1C), mRNA.	-0.06	6.94	2.40E-03	3.65E-03	grey60
HIST1H2AC	8334	histone cluster 1, H2ac (HIST1H2AC), mRNA.	-0.01	5.72	2.69E-01	3.11E-01	grey60
HIST1H2BD	3017	histone cluster 1, H2bd (HIST1H2BD), transcript variant 2, mRNA.	2.06	6.75	3.95E-94	1.25E-91	grey60
HIST1H2BF	8343	histone cluster 1, H2bf (HIST1H2BF), mRNA.	0.02	9.39	8.44E-02	1.07E-01	grey60
HIST1H2BG	8339	histone cluster 1, H2bg (HIST1H2BG), mRNA.	0.11	6.49	1.62E-09	3.96E-09	grey60
HIST1H2BH	8345	histone cluster 1, H2bh (HIST1H2BH), mRNA.	0.15	11.01	9.15E-20	3.87E-19	grey60

HIST1H2BJ	8970	histone cluster 1, H2bj (HIST1H2BJ), mRNA.	-0.44	7.13	2.44E-54	4.70E-53	grey60
HIST1H2BK	85236	histone cluster 1, H2bk (HIST1H2BK), mRNA.	0.00	8.94	9.04E-01	9.19E-01	grey60
HIST1H3D	8351	histone cluster 1, H3d (HIST1H3D), mRNA.	0.09	9.32	2.24E-08	5.09E-08	grey60
HIST1H4H	8365	histone cluster 1, H4h (HIST1H4H), mRNA.	0.12	6.55	1.74E-16	6.24E-16	grey60
HIST2H2AC	8338	histone cluster 2, H2ac (HIST2H2AC), mRNA.	0.04	7.77	1.85E-02	2.54E-02	grey60
HLA-DMA	3108	major histocompatibility complex, class II, DM alpha (HLA-DMA), mRNA.	-0.14	6.27	2.03E-16	7.27E-16	grey60
HLA-DOA	3111	major histocompatibility complex, class II, DO alpha (HLA-DOA), mRNA.	-0.60	7.99	9.03E-54	1.65E-52	grey60
HLA-DPA1	3113	major histocompatibility complex, class II, DP alpha 1 (HLA-DPA1), mRNA.	-0.03	8.22	3.01E-02	4.02E-02	grey60
HLA-DPB1	3115	major histocompatibility complex, class II, DP beta 1 (HLA-DPB1), mRNA.	0.05	9.04	4.78E-04	7.83E-04	grey60
HLA-DRA	3122	major histocompatibility complex, class II, DR alpha (HLA-DRA), mRNA.	0.17	7.73	2.80E-19	1.16E-18	grey60
HLA-DRB3	3125	major histocompatibility complex, class II, DR beta 3 (HLA-DRB3), mRNA.	-0.12	7.51	3.68E-09	8.81E-09	grey60

HSPB1	3315	heat shock 27kDa protein 1 (HSPB1), mRNA.	-0.21	7.38	2.17E-30	1.42E-29	grey60
IGSF3	3321	immunoglobulin superfamily, member 3 (IGSF3), transcript variant 2, mRNA.	-0.12	5.77	2.17E-10	5.57E-10	grey60
IL10	3586	interleukin 10 (IL10), mRNA.	-0.22	8.65	1.95E-30	1.27E-29	grey60
IL1R2	7850	interleukin 1 receptor, type II (IL1R2), transcript variant 2, mRNA.	0.03	6.64	2.99E-02	4.00E-02	grey60
JAM2	58494	junctional adhesion molecule 2 (JAM2), mRNA.	-0.19	10.22	4.72E-32	3.36E-31	grey60
KDELC2	143888	KDEL (Lys-Asp-Glu-Leu) containing 2 (KDELC2), mRNA.	0.78	8.24	4.10E-93	1.16E-90	grey60
MAL	4118	mal, T-cell differentiation protein (MAL), transcript variant a, mRNA.	-0.05	8.65	1.38E-05	2.60E-05	grey60
NA	8337	histone cluster 2, H2aa3 (HIST2H2AA3), mRNA.	0.41	6.33	7.44E-54	1.37E-52	grey60
NA	8362	histone cluster 1, H4k (HIST1H4K), mRNA.	-0.35	8.12	1.10E-45	1.47E-44	grey60
NA	51192	chemokine-like factor (CKLF), transcript variant 3, mRNA.	-0.09	8.51	9.29E-11	2.43E-10	grey60
NA	8544	pirin (iron-binding nuclear protein) (PIR), transcript variant 1, mRNA.	-0.04	5.63	5.39E-03	7.91E-03	grey60
NA	8370	histone cluster 2, H4a (HIST2H4A), mRNA.	0.02	8.04	3.85E-01	4.31E-01	grey60

NRCAM	4897	neuronal cell adhesion molecule (NRCAM), transcript variant 1, mRNA.	-0.06	9.97	4.13E-08	9.22E-08	grey60
PHACTR2	9749	phosphatase and actin regulator 2 (PHACTR2), mRNA.	-0.02	8.21	2.25E-01	2.64E-01	grey60
PITPNC1	26207	phosphatidylinositol transfer protein, cytoplasmic 1 (PITPNC1), transcript variant 2, mRNA.	0.18	9.24	3.13E-34	2.42E-33	grey60
PON2	5445	paraoxonase 2 (PON2), transcript variant 2, mRNA.	0.09	8.04	4.61E-10	1.16E-09	grey60
PRRX1	5396	paired related homeobox 1 (PRRX1), transcript variant pmx-1a, mRNA.	-0.04	11.59	4.14E-05	7.46E-05	grey60
RPS6KA2	6196	ribosomal protein S6 kinase, 90kDa, polypeptide 2 (RPS6KA2), transcript variant 2, mRNA.	-0.14	7.36	1.01E-13	3.11E-13	grey60
RRAGD	58528	Ras-related GTP binding D (RRAGD), mRNA.	-0.15	7.43	9.91E-15	3.24E-14	grey60
SETD9	133383	chromosome 5 open reading frame 35 (C5orf35), mRNA.	-0.06	7.32	2.51E-03	3.80E-03	grey60
TCEAL2	140597	transcription elongation factor A (SII)-like 2 (TCEAL2), mRNA.	-0.03	9.72	1.02E-02	1.45E-02	grey60
TCL1A	8115	T-cell leukemia/lymphoma 1A (TCL1A), mRNA.	-0.02	7.77	1.99E-01	2.37E-01	grey60
TGM5	9333	transglutaminase 5 (TGM5), transcript variant 2, mRNA.	-0.12	6.53	1.10E-14	3.57E-14	grey60

TMBIM6	7009	testis enhanced gene transcript (BAX inhibitor 1) (TEGT), mRNA.	0.15	6.03	3.27E-17	1.22E-16	grey60
TMEM136	219902	transmembrane protein 136 (TMEM136), mRNA.	0.00	7.72	9.75E-01	9.79E-01	grey60
TMEM200A	114801	KIAA1913 (KIAA1913), mRNA.	0.03	6.96	7.66E-02	9.73E-02	grey60
TSPO	706	translocator protein (18kDa) (TSPO), transcript variant PBR, mRNA.	0.18	6.53	8.90E-24	4.54E-23	grey60
WWC3	55841	WWC family member 3 (WWC3), mRNA.	-0.41	5.88	1.38E-47	2.01E-46	grey60
ZCCHC11	23318	zinc finger, CCHC domain containing 11 (ZCCHC11), transcript variant 3, mRNA.	0.77	8.66	1.56E-66	5.97E-65	grey60
ACCS	84680	1-aminocyclopropane-1-carboxylate synthase (PHACS), mRNA.	-0.04	8.34	2.57E-03	3.89E-03	lightcyan
ACOT9	23597	acyl-CoA thioesterase 9 (ACOT9), transcript variant 2, mRNA.	0.40	6.24	7.02E-54	1.31E-52	lightcyan
AHSA2	130872	AHA1, activator of heat shock 90kDa protein ATPase homolog 2 (yeast) (AHSA2), mRNA.	-0.01	9.20	4.01E-01	4.47E-01	lightcyan
ANAPC1	64682	anaphase promoting complex subunit 1 (ANAPC1), mRNA.	0.01	7.47	6.04E-01	6.46E-01	lightcyan
ANKRD12	23253	ankyrin repeat domain 12 (ANKRD12), mRNA.	0.40	5.85	1.53E-46	2.12E-45	lightcyan
ARHGAP4	393	Rho GTPase activating protein 4 (ARHGAP4), mRNA.	0.03	8.17	2.60E-02	3.51E-02	lightcyan

ARHGEF7	8874	Rho guanine nucleotide exchange factor (GEF) 7 (ARHGEF7), transcript variant 1, mRNA.	-0.02	7.26	3.65E-01	4.10E-01	lightcyan
BRPF3	27154	bromodomain and PHD finger containing, 3 (BRPF3), mRNA.	-0.30	10.65	2.89E-54	5.54E-53	lightcyan
C2CD5	9847	KIAA0528 (KIAA0528), mRNA.	-0.25	6.77	4.04E-23	2.00E-22	lightcyan
CAPZB	832	capping protein (actin filament) muscle Z-line, beta (CAPZB), mRNA.	-0.01	8.29	4.23E-01	4.69E-01	lightcyan
CCDC14	64770	coiled-coil domain containing 14 (CCDC14), mRNA.	0.25	6.38	4.12E-27	2.44E-26	lightcyan
CCDC84	338657	coiled-coil domain containing 84 (CCDC84), mRNA.	-0.10	7.84	1.70E-11	4.64E-11	lightcyan
CENPF	1063	centromere protein F, 350/400ka (mitosin) (CENPF), mRNA.	0.28	8.71	3.48E-42	3.91E-41	lightcyan
CEP192	55125	centrosomal protein 192kDa (CEP192), mRNA.	0.12	9.08	4.31E-13	1.29E-12	lightcyan
CEP83	51134	coiled-coil domain containing 41 (CCDC41), transcript variant 2, mRNA.	0.01	11.17	4.30E-01	4.76E-01	lightcyan
CHAMP1	283489	chromosome 13 open reading frame 8 (C13orf8), mRNA.	-0.07	7.06	1.45E-06	2.93E-06	lightcyan
CLDN15	24146	claudin 15 (CLDN15), transcript variant 1, mRNA.	0.21	6.38	2.15E-23	1.08E-22	lightcyan

CNOT1	23019	CCR4-NOT transcription complex, subunit 1 (CNOT1), transcript variant 1, mRNA.	0.21	8.20	4.24E-36	3.57E-35	lightcyan
CRIPAK	285464	cysteine-rich PAK1 inhibitor (CRIPAK), mRNA.	0.02	7.33	2.96E-01	3.40E-01	lightcyan
DDX55	57696	DEAD (Asp-Glu-Ala-Asp) box polypeptide 55 (DDX55), mRNA.	-0.01	7.50	3.71E-01	4.16E-01	lightcyan
DIAPH1	1729	diaphanous homolog 1 (Drosophila) (DIAPH1), transcript variant 2, mRNA.	-0.06	7.82	2.34E-03	3.55E-03	lightcyan
DNAJB2	3300	DnaJ (Hsp40) homolog, subfamily B, member 2 (DNAJB2), transcript variant 1, mRNA.	-0.05	9.10	1.37E-04	2.35E-04	lightcyan
DYNC1LI2	1783	dynein, cytoplasmic 1, light intermediate chain 2 (DYNC1LI2), mRNA.	-0.02	9.92	1.28E-01	1.57E-01	lightcyan
EIF4A2	1974	eukaryotic translation initiation factor 4A, isoform 2 (EIF4A2), mRNA.	0.20	6.55	3.06E-21	1.38E-20	lightcyan
EXOC7	23265	exocyst complex component 7 (EXOC7), transcript variant 2, mRNA.	0.00	7.97	9.48E-01	9.55E-01	lightcyan
FAM13B	51306	chromosome 5 open reading frame 5 (C5orf5), mRNA.	-0.22	8.22	3.93E-31	2.65E-30	lightcyan
FNBP4	23360	formin binding protein 4 (FNBP4), mRNA.	-0.05	7.07	6.85E-04	1.10E-03	lightcyan
GFOD1	54438	glucose-fructose oxidoreductase domain containing 1 (GFOD1), mRNA.	-0.02	9.71	7.39E-02	9.43E-02	lightcyan

GSE1	23199	KIAA0182 (KIAA0182), mRNA.	-0.01	7.83	6.87E-01	7.22E-01	lightcyan
GTPBP3	84705	GTP binding protein 3 (mitochondrial) (GTPBP3), transcript variant V, mRNA.	-0.06	8.84	4.50E-04	7.41E-04	lightcyan
HEATR1	55127	HEAT repeat containing 1 (HEATR1), mRNA.	-0.02	7.79	1.79E-01	2.14E-01	lightcyan
HMBOX1	79618	homeobox containing 1 (HMBOX1), mRNA.	0.06	10.04	1.31E-08	3.04E-08	lightcyan
ILK	3611	integrin-linked kinase (ILK), transcript variant 3, mRNA.	0.16	5.66	5.35E-17	1.98E-16	lightcyan
KANSL3	55683	hypothetical protein FLJ10081 (FLJ10081), mRNA.	0.29	8.37	5.99E-44	7.22E-43	lightcyan
KBTBD2	25948	kelch repeat and BTB (POZ) domain containing 2 (KBTBD2), mRNA.	0.02	9.36	1.37E-01	1.67E-01	lightcyan
KDM2B	84678	F-box and leucine-rich repeat protein 10 (FBXL10), transcript variant 1, mRNA.	0.01	10.16	3.02E-01	3.47E-01	lightcyan
KNTC1	9735	kinetochore associated 1 (KNTC1), mRNA.	0.07	7.78	3.85E-06	7.54E-06	lightcyan
KPNA6	23633	karyopherin alpha 6 (importin alpha 7) (KPNA6), mRNA.	0.11	7.86	7.35E-07	1.51E-06	lightcyan
LAMA5	3911	laminin, alpha 5 (LAMA5), mRNA.	-0.03	6.27	4.03E-02	5.32E-02	lightcyan
LARP1	23367	La ribonucleoprotein domain family, member 1 (LARP1), transcript variant 2, mRNA.	-0.15	8.17	1.96E-17	7.40E-17	lightcyan

LRP5L	91355	low density lipoprotein receptor-related protein 5-like (LRP5L), mRNA.	-0.10	6.50	6.46E-09	1.53E-08	lightcyan
LUC7L3	51747	cisplatin resistance-associated overexpressed protein (CROP), transcript variant 1, mRNA.	0.10	10.60	7.17E-07	1.47E-06	lightcyan
LZTR1	8216	leucine-zipper-like transcription regulator 1 (LZTR1), mRNA.	0.01	8.03	6.11E-01	6.52E-01	lightcyan
MACF1	23499	microtubule-actin crosslinking factor 1 (MACF1), transcript variant 1, mRNA.	-0.01	6.06	6.89E-01	7.24E-01	lightcyan
MAT2A	4144	methionine adenosyltransferase II, alpha (MAT2A), mRNA.	-0.07	7.00	1.93E-05	3.58E-05	lightcyan
MBD6	114785	methyl-CpG binding domain protein 6 (MBD6), mRNA.	0.09	7.78	2.18E-04	3.69E-04	lightcyan
MDC1	9656	mediator of DNA damage checkpoint 1 (MDC1), mRNA.	0.00	8.23	9.22E-01	9.34E-01	lightcyan
MLLT6	4302	myeloid/lymphoid or mixed-lineage leukemia (trithorax homolog, Drosophila); translocated to, 6 (MLLT6), mRNA.	0.08	7.92	6.89E-09	1.62E-08	lightcyan
MPPE1	65258	metallophosphoesterase 1 (MPPE1), mRNA.	0.03	9.99	5.81E-02	7.51E-02	lightcyan
MRPS25	64432	mitochondrial ribosomal protein S25 (MRPS25), nuclear gene encoding mitochondrial protein, mRNA.	0.01	7.16	4.21E-01	4.67E-01	lightcyan

MUM1	84939	melanoma associated antigen (mutated) 1 (MUM1), mRNA.	0.05	7.77	9.52E-03	1.36E-02	lightcyan
NA	54441	DKFZp434A0131 protein (DKFZP434A0131), transcript variant 1, mRNA.	0.17	8.56	9.05E-38	8.36E-37	lightcyan
NA	9284	nuclear pore complex interacting protein (NPIP), mRNA.	-0.16	9.88	2.63E-27	1.57E-26	lightcyan
NA	8681	phospholipase A2, group IVB (cytosolic) (PLA2G4B), mRNA.	0.15	7.52	1.63E-15	5.54E-15	lightcyan
NA	22995	centrosomal protein 152kDa (CEP152), mRNA.	0.08	7.05	3.60E-06	7.08E-06	lightcyan
NA	442578	similar to Cohesin subunit SA-3 (Stromal antigen 3) (Stromalin 3) (SCC3 homolog 3) (LOC442578), mRNA.	0.01	9.26	2.39E-01	2.79E-01	lightcyan
NA	400986	protein immuno-reactive with anti-PTH polyclonal antibodies (LOC400986), mRNA.	0.00	8.71	8.27E-01	8.50E-01	lightcyan
NCKAP1L	3071	NCK-associated protein 1-like (NCKAP1L), mRNA.	-0.26	7.41	2.39E-30	1.56E-29	lightcyan
NCOA6	23054	nuclear receptor coactivator 6 (NCOA6), mRNA.	-0.12	8.35	3.30E-16	1.17E-15	lightcyan
NISCH	11188	nischarin (NISCH), mRNA.	0.06	11.01	1.19E-05	2.26E-05	lightcyan
NKTR	4820	natural killer-tumor recognition sequence (NKTR), transcript variant 1, mRNA.	0.08	9.32	2.57E-08	5.82E-08	lightcyan

NPIPB9	728734	PREDICTED: similar to kidney-specific protein (KS), transcript variant 2 (LOC728734), mRNA.	-0.04	7.39	2.99E-03	4.50E-03	lightcyan
OGT	8473	O-linked N-acetylglucosamine (GlcNAc) transferase (UDP-N-acetylglucosamine:polypeptide-N-acetylglucosaminyl transferase) (OGT), transcript variant 2, mRNA.	0.13	5.90	1.65E-15	5.60E-15	lightcyan
OSBPL7	114881	oxysterol binding protein-like 7 (OSBPL7), transcript variant 1, mRNA.	-0.07	8.44	1.98E-08	4.52E-08	lightcyan
PCNT	5116	pericentrin (kendrin) (PCNT), mRNA.	0.00	9.29	8.23E-01	8.48E-01	lightcyan
PDCD11	22984	programmed cell death 11 (PDCD11), mRNA.	0.04	8.58	1.58E-03	2.44E-03	lightcyan
PHF19	26147	PHD finger protein 19 (PHF19), transcript variant 1, mRNA.	-0.09	7.12	2.74E-10	7.02E-10	lightcyan
PIEZO1	9780	family with sequence similarity 38, member A (FAM38A), mRNA.	0.04	7.18	9.39E-04	1.49E-03	lightcyan
POLQ	10721	polymerase (DNA directed), theta (POLQ), mRNA.	-0.09	10.29	5.17E-05	9.22E-05	lightcyan
PSME4	23198	proteasome (prosome, macropain) activator subunit 4 (PSME4), mRNA.	0.03	7.48	8.46E-03	1.22E-02	lightcyan
QSOX2	169714	quiescin Q6-like 1 (QSCN6L1), mRNA.	0.02	8.43	2.09E-01	2.47E-01	lightcyan

RAD54L2	23132	RAD54-like 2 (S. cerevisiae) (RAD54L2), mRNA.	-0.04	9.33	7.65E-04	1.23E-03	lightcyan
RBM25	58517	RNA binding motif protein 25 (RBM25), mRNA.	-0.03	9.24	1.66E-02	2.29E-02	lightcyan
RBM33	155435	RNA binding motif protein 33 (RBM33), mRNA.	0.08	7.34	3.63E-04	6.02E-04	lightcyan
RBM5	10181	RNA binding motif protein 5 (RBM5), mRNA.	0.03	7.79	1.72E-01	2.07E-01	lightcyan
RNASEH1	246243	ribonuclease H1 (RNASEH1), mRNA.	-0.09	7.60	3.57E-07	7.51E-07	lightcyan
RNF19A	25897	ring finger protein 19 (RNF19), transcript variant 2, mRNA.	-0.21	7.33	1.15E-30	7.63E-30	lightcyan
RPS6KA1	6195	ribosomal protein S6 kinase, 90kDa, polypeptide 1 (RPS6KA1), transcript variant 2, mRNA.	-0.07	9.87	8.66E-10	2.15E-09	lightcyan
RSRP1	57035	chromosome 1 open reading frame 63 (C1orf63), mRNA.	0.06	9.31	5.59E-04	9.10E-04	lightcyan
SCAP	22937	SREBF chaperone (SCAP), mRNA.	0.19	7.24	5.36E-27	3.14E-26	lightcyan
SETD2	29072	SET domain containing 2 (SETD2), mRNA.	-0.04	6.62	2.11E-02	2.89E-02	lightcyan
SF3B3	23450	splicing factor 3b, subunit 3, 130kDa (SF3B3), mRNA.	-0.01	9.30	5.41E-01	5.86E-01	lightcyan
SKIV2L	6499	superkiller viralicidic activity 2-like (S. cerevisiae) (SKIV2L), mRNA.	0.21	8.17	2.24E-39	2.19E-38	lightcyan
SMG1	23049	PI-3-kinase-related kinase SMG-1 (SMG1), mRNA.	0.01	7.57	4.89E-01	5.35E-01	lightcyan

SND1-IT1	27099	nasopharyngeal carcinoma associated gene protein-8 (NAG8), mRNA.	-0.36	8.46	4.21E-62	1.30E-60	lightcyan
SNORA25	684959	small nucleolar RNA, H/ACA box 25 (SNORA25) on chromosome 11.	0.18	7.56	1.49E-23	7.51E-23	lightcyan
SPEN	23013	spen homolog, transcriptional regulator (Drosophila) (SPEN), mRNA.	0.01	6.95	5.23E-01	5.67E-01	lightcyan
STK35	140901	serine/threonine kinase 35 (STK35), mRNA.	0.77	7.42	1.16E-57	2.72E-56	lightcyan
STK36	27148	serine/threonine kinase 36, fused homolog (Drosophila) (STK36), mRNA.	-0.15	7.84	5.50E-17	2.04E-16	lightcyan
SUGP2	10147	splicing factor, arginine/serine-rich 14 (SFRS14), transcript variant 2, mRNA.	0.12	11.19	4.29E-11	1.14E-10	lightcyan
TAF1C	9013	TATA box binding protein (TBP)-associated factor, RNA polymerase I, C, 110kDa (TAF1C), transcript variant 1, mRNA.	-0.33	8.21	1.74E-32	1.26E-31	lightcyan
TARBP1	6894	Tar (HIV-1) RNA binding protein 1 (TARBP1), mRNA.	0.01	10.76	5.41E-01	5.86E-01	lightcyan
TRRAP	8295	transformation/transcription domain-associated protein (TRRAP), mRNA.	0.22	7.46	4.17E-50	6.58E-49	lightcyan
UBE2G2	7327	ubiquitin-conjugating enzyme E2G 2 (UBC7 homolog, yeast) (UBE2G2), transcript variant 1, mRNA.	-0.02	7.80	1.44E-01	1.76E-01	lightcyan

USB1	79650	chromosome 16 open reading frame 57 (C16orf57), mRNA.	0.06	9.16	1.16E-02	1.63E-02	lightcyan
VAMP1	6843	vesicle-associated membrane protein 1 (synaptobrevin 1) (VAMP1), transcript variant 3, mRNA.	0.07	9.15	2.09E-05	3.87E-05	lightcyan
VEZF1	7716	vascular endothelial zinc finger 1 (VEZF1), mRNA.	0.17	10.54	1.37E-12	3.99E-12	lightcyan
WDR19	57728	WD repeat domain 19 (WDR19), mRNA.	-0.39	8.08	7.47E-67	2.87E-65	lightcyan
XPO4	64328	exportin 4 (XPO4), mRNA.	0.88	5.81	1.30E-56	2.85E-55	lightcyan
ZFHX3	463	AT-binding transcription factor 1 (ATBF1), mRNA.	0.01	6.27	7.22E-01	7.55E-01	lightcyan
AGA	175	aspartylglucosaminidase (AGA), mRNA.	-0.01	9.19	5.32E-01	5.77E-01	lightgreen
ALCAM	214	activated leukocyte cell adhesion molecule (ALCAM), mRNA.	-0.29	5.95	4.31E-34	3.29E-33	lightgreen
ARID5B	84159	AT rich interactive domain 5B (MRF1-like) (ARID5B), mRNA.	0.10	6.55	2.62E-08	5.93E-08	lightgreen
BPHL	670	biphenyl hydrolase-like (serine hydrolase; breast epithelial mucin-associated antigen) (BPHL), mRNA.	-0.40	6.20	4.89E-57	1.10E-55	lightgreen
C1GALT1	56913	core 1 synthase, glycoprotein-N-acetylgalactosamine 3-beta-galactosyltransferase, 1 (C1GALT1), mRNA.	-0.29	6.80	2.17E-45	2.84E-44	lightgreen
CCDC12	151903	coiled-coil domain containing 12 (CCDC12), mRNA.	-0.08	6.99	1.51E-07	3.25E-07	lightgreen

CCNC	892	cyclin C (CCNC), transcript variant 1, mRNA.	-0.20	8.71	8.09E-28	4.92E-27	lightgreen
CENPV	201161	proline rich 6 (PRR6), mRNA.	0.06	7.78	1.16E-06	2.35E-06	lightgreen
CLEC2D	29121	C-type lectin domain family 2, member D (CLEC2D), transcript variant 2, mRNA.	-0.25	7.82	5.60E-35	4.46E-34	lightgreen
CMBL	134147	carboxymethylenebutenolidase homolog (Pseudomonas) (CMBL), mRNA.	0.12	5.99	2.21E-11	6.01E-11	lightgreen
COPRS	55352	chromosome 17 open reading frame 79 (C17orf79), mRNA.	-0.04	5.94	1.13E-03	1.78E-03	lightgreen
DERL1	79139	Der1-like domain family, member 1 (DERL1), mRNA.	0.10	9.64	2.88E-16	1.02E-15	lightgreen
EIF2AK3	9451	eukaryotic translation initiation factor 2-alpha kinase 3 (EIF2AK3), mRNA.	-0.08	7.19	1.49E-07	3.21E-07	lightgreen
EMC7	56851	chromosome 15 open reading frame 24 (C15orf24), mRNA.	0.14	9.64	8.08E-13	2.38E-12	lightgreen
FNDC3A	22862	fibronectin type III domain containing 3A (FNDC3A), transcript variant 2, mRNA.	-0.11	6.55	1.17E-12	3.43E-12	lightgreen
FUT8	2530	fucosyltransferase 8 (alpha (1,6) fucosyltransferase) (FUT8), transcript variant 2, mRNA.	-0.05	7.47	7.05E-05	1.25E-04	lightgreen
GNAI3	2773	guanine nucleotide binding protein (G protein), alpha inhibiting activity polypeptide 3 (GNAI3), mRNA.	0.13	7.43	4.37E-16	1.53E-15	lightgreen

GNG11	2791	guanine nucleotide binding protein (G protein), gamma 11 (GNG11), mRNA.	-0.02	8.78	1.17E-01	1.44E-01	lightgreen
GOLPH3L	55204	golgi phosphoprotein 3-like (GOLPH3L), mRNA.	0.60	8.24	5.80E-64	2.00E-62	lightgreen
GSTA4	2941	glutathione S-transferase A4 (GSTA4), mRNA.	-0.04	5.99	2.94E-02	3.94E-02	lightgreen
HNRNPLL	92906	heterogeneous nuclear ribonucleoprotein L-like (HNRPLL), mRNA.	-0.02	5.93	2.44E-01	2.84E-01	lightgreen
HS2ST1	9653	heparan sulfate 2-O-sulfotransferase 1 (HS2ST1), mRNA.	0.07	5.99	3.72E-05	6.72E-05	lightgreen
ICA1	3382	islet cell autoantigen 1, 69kDa (ICA1), transcript variant 1, mRNA.	0.00	8.58	9.43E-01	9.52E-01	lightgreen
INSIG2	51141	insulin induced gene 2 (INSIG2), mRNA.	-0.01	7.14	3.86E-01	4.32E-01	lightgreen
IRF8	3394	interferon regulatory factor 8 (IRF8), mRNA.	0.25	5.68	3.35E-39	3.24E-38	lightgreen
ITM2A	9452	integral membrane protein 2A (ITM2A), mRNA.	0.26	10.11	1.05E-37	9.64E-37	lightgreen
KIAA0922	23240	KIAA0922 (KIAA0922), mRNA.	-0.08	6.69	8.94E-11	2.35E-10	lightgreen
LMBRD1	55788	LMBR1 domain containing 1 (LMBRD1), mRNA.	-1.30	7.55	4.70E-86	6.63E-84	lightgreen
MBNL2	10150	muscleblind-like 2 (Drosophila) (MBNL2), transcript variant 1, mRNA.	0.43	8.36	3.16E-56	6.76E-55	lightgreen

MGST2	4258	microsomal glutathione S-transferase 2 (MGST2), mRNA.	-0.01	9.09	5.11E-01	5.56E-01	lightgreen
MTDH	92140	metadherin (MTDH), mRNA.	0.00	8.63	8.16E-01	8.41E-01	lightgreen
NA	124944	chromosome 17 open reading frame 49 (C17orf49), mRNA.	0.04	8.45	8.62E-03	1.24E-02	lightgreen
NASP	4678	nuclear autoantigenic sperm protein (histone-binding) (NASP), transcript variant 1, mRNA.	-0.07	8.21	2.25E-07	4.79E-07	lightgreen
NME4	4833	non-metastatic cells 4, protein expressed in (NME4), mRNA.	-0.57	9.58	5.42E-68	2.18E-66	lightgreen
NR2F6	2063	nuclear receptor subfamily 2, group F, member 6 (NR2F6), mRNA.	0.22	7.98	3.54E-32	2.53E-31	lightgreen
NRD1	4898	nardilysin (N-arginine dibasic convertase) (NRD1), mRNA.	0.01	7.22	4.69E-01	5.14E-01	lightgreen
NUCB2	4925	nucleobindin 2 (NUCB2), mRNA.	-0.63	6.36	1.95E-62	6.14E-61	lightgreen
PAPSS1	9061	3'-phosphoadenosine 5'-phosphosulfate synthase 1 (PAPSS1), mRNA.	0.02	7.99	1.08E-01	1.34E-01	lightgreen
PELI1	57162	pellino homolog 1 (Drosophila) (PELI1), mRNA.	-0.20	7.60	2.19E-25	1.21E-24	lightgreen
PLEKHF1	79156	pleckstrin homology domain containing, family F (with FYVE domain) member 1 (PLEKHF1), mRNA.	-0.15	6.33	6.85E-12	1.91E-11	lightgreen

PRKRIR	5612	protein-kinase, interferon-inducible double stranded RNA dependent inhibitor, repressor of (P58 repressor) (PRKRIR), mRNA.	1.08	6.25	1.16E-63	3.96E-62	lightgreen
PRRC1	133619	proline-rich coiled-coil 1 (PRRC1), mRNA.	-0.13	8.89	1.67E-21	7.62E-21	lightgreen
RAD51AP1	10635	RAD51 associated protein 1 (RAD51AP1), mRNA.	0.21	10.20	9.15E-35	7.19E-34	lightgreen
RPN2	6185	ribophorin II (RPN2), mRNA.	0.14	7.93	4.53E-17	1.68E-16	lightgreen
SAR1B	51128	SAR1 gene homolog B (S. cerevisiae) (SAR1B), transcript variant 2, mRNA.	-0.07	8.99	1.59E-05	2.98E-05	lightgreen
SARAF	51669	transmembrane protein 66 (TMEM66), mRNA.	-0.03	9.18	9.27E-02	1.17E-01	lightgreen
SCCPDH	51097	saccharopine dehydrogenase (putative) (SCCPDH), mRNA.	0.01	10.20	3.40E-01	3.84E-01	lightgreen
SEC11C	90701	SEC11 homolog C (S. cerevisiae) (SEC11C), mRNA.	-0.12	9.46	6.72E-14	2.10E-13	lightgreen
SEC24D	9871	SEC24 related gene family, member D (S. cerevisiae) (SEC24D), mRNA.	-0.41	9.11	2.28E-60	6.39E-59	lightgreen
SERP1	27230	stress-associated endoplasmic reticulum protein 1 (SERP1), mRNA.	0.10	7.04	3.23E-12	9.20E-12	lightgreen
SLC25A46	91137	solute carrier family 25, member 46 (SLC25A46), mRNA.	0.11	6.68	1.48E-08	3.40E-08	lightgreen

SLC30A5	64924	solute carrier family 30 (zinc transporter), member 5 (SLC30A5), transcript variant 1, mRNA.	-0.40	10.43	1.67E-42	1.92E-41	lightgreen
SLC33A1	9197	solute carrier family 33 (acetyl-CoA transporter), member 1 (SLC33A1), mRNA.	-0.14	6.05	1.08E-13	3.32E-13	lightgreen
SRP72	6731	signal recognition particle 72kDa (SRP72), mRNA.	-0.23	7.20	3.04E-22	1.43E-21	lightgreen
STARD10	10809	START domain containing 10 (STARD10), mRNA.	-0.11	7.21	2.99E-09	7.19E-09	lightgreen
STT3A	3703	STT3, subunit of the oligosaccharyltransferase complex, homolog A (S. cerevisiae) (STT3A), mRNA.	-0.07	7.20	2.19E-04	3.70E-04	lightgreen
TBC1D7	51256	TBC1 domain family, member 7 (TBC1D7), mRNA.	0.29	7.88	9.27E-47	1.30E-45	lightgreen
TIMP1	7076	TIMP metallopeptidase inhibitor 1 (TIMP1), mRNA.	0.12	5.40	5.44E-11	1.44E-10	lightgreen
TMED2	10959	transmembrane emp24 domain trafficking protein 2 (TMED2), mRNA.	0.48	7.76	1.35E-67	5.38E-66	lightgreen
TMED7	51014	transmembrane emp24 protein transport domain containing 7 (TMED7), mRNA.	-0.21	6.93	5.81E-32	4.11E-31	lightgreen
TMEM154	201799	transmembrane protein 154 (TMEM154), mRNA.	-0.26	6.70	1.52E-22	7.28E-22	lightgreen

TNIP1	10318	TNFAIP3 interacting protein 1 (TNIP1), mRNA.	0.14	7.95	3.89E-13	1.17E-12	lightgreen
TRAM1	23471	translocation associated membrane protein 1 (TRAM1), mRNA.	0.07	7.61	1.84E-05	3.43E-05	lightgreen
TTC37	9652	KIAA0372 (KIAA0372), mRNA.	0.01	6.52	3.44E-01	3.89E-01	lightgreen
UAP1	6675	UDP-N-acteylglucosamine pyrophosphorylase 1 (UAP1), mRNA.	0.04	9.51	9.20E-05	1.61E-04	lightgreen
UBAC1	10422	ubiquitin associated domain containing 1 (UBADC1), mRNA.	-0.01	12.63	6.61E-01	6.98E-01	lightgreen
UBR5	51366	E3 ubiquitin protein ligase, HECT domain containing, 1 (EDD1), mRNA.	-0.06	5.73	6.75E-05	1.19E-04	lightgreen
UBTD2	92181	ubiquitin domain containing 2 (UBTD2), mRNA.	0.07	6.63	2.51E-06	4.97E-06	lightgreen
UBXN1	51035	SAPK substrate protein 1 (LOC51035), mRNA.	0.00	9.52	8.82E-01	8.99E-01	lightgreen
USO1	8615	vesicle docking protein p115 (VDP), mRNA.	-0.12	6.94	9.88E-18	3.79E-17	lightgreen
VGLL4	9686	vestigial like 4 (Drosophila) (VGLL4), mRNA.	0.03	9.24	1.71E-02	2.36E-02	lightgreen
AK3	50808	adenylate kinase 3 (AK3), mRNA.	-0.03	9.26	7.39E-02	9.43E-02	lightyellow
ALDH16A1	126133	aldehyde dehydrogenase 16 family, member A1 (ALDH16A1), mRNA.	0.03	7.28	5.72E-02	7.40E-02	lightyellow
ASPM	259266	asp (abnormal spindle) homolog, microcephaly associated (Drosophila) (ASPM), mRNA.	0.00	6.76	9.67E-01	9.73E-01	lightyellow

AURKA	6790	aurora kinase A (AURKA), transcript variant 5, mRNA.	-0.16	6.61	3.82E-24	1.99E-23	lightyellow
BORA	79866	chromosome 13 open reading frame 34 (C13orf34), mRNA.	0.04	9.05	5.61E-03	8.21E-03	lightyellow
BUB1	699	BUB1 budding uninhibited by benzimidazoles 1 homolog (yeast) (BUB1), mRNA.	0.09	7.86	1.24E-05	2.34E-05	lightyellow
BUB1B	701	BUB1 budding uninhibited by benzimidazoles 1 homolog beta (yeast) (BUB1B), mRNA.	-0.09	6.74	6.27E-06	1.21E-05	lightyellow
C12orf43	64897	chromosome 12 open reading frame 43 (C12orf43), mRNA.	0.12	7.64	2.24E-05	4.13E-05	lightyellow
CCL17	6361	chemokine (C-C motif) ligand 17 (CCL17), mRNA.	0.12	9.30	2.64E-11	7.14E-11	lightyellow
CCNA2	890	cyclin A2 (CCNA2), mRNA.	-0.02	7.60	1.60E-01	1.93E-01	lightyellow
CCNB2	9133	cyclin B2 (CCNB2), mRNA.	0.01	8.25	5.10E-01	5.55E-01	lightyellow
CCNF	899	cyclin F (CCNF), mRNA.	0.03	9.59	1.13E-02	1.60E-02	lightyellow
CDCA3	83461	cell division cycle associated 3 (CDCA3), mRNA.	0.06	8.60	2.12E-05	3.91E-05	lightyellow
CDKN3	1033	cyclin-dependent kinase inhibitor 3 (CDK2-associated dual specificity phosphatase) (CDKN3), mRNA.	0.31	6.18	8.57E-40	8.51E-39	lightyellow
CENPA	1058	centromere protein A (CENPA), transcript variant 1, mRNA.	-0.01	7.40	4.79E-01	5.24E-01	lightyellow
CENPE	1062	centromere protein E, 312kDa (CENPE), mRNA.	-0.09	7.38	1.69E-09	4.11E-09	lightyellow

CEP55	55165	centrosomal protein 55kDa (CEP55), mRNA.	-0.05	7.12	2.98E-04	4.97E-04	lightyellow
CGN	57530	cingulin (CGN), mRNA.	0.05	8.23	2.87E-04	4.81E-04	lightyellow
DLGAP5	9787	discs, large homolog 7 (Drosophila) (DLG7), mRNA.	0.05	8.53	5.57E-04	9.07E-04	lightyellow
DOCK11	139818	dedicator of cytokinesis 11 (DOCK11), mRNA.	0.05	9.24	1.15E-02	1.62E-02	lightyellow
HLA-DRB4	3126	major histocompatibility complex, class II, DR beta 4 (HLA-DRB4), mRNA.	-0.06	5.38	1.03E-03	1.62E-03	lightyellow
HMMR	3161	hyaluronan-mediated motility receptor (RHAMM) (HMMR), transcript variant 2, mRNA.	-0.02	9.60	1.09E-01	1.36E-01	lightyellow
ITPRIP	85450	KIAA1754 (KIAA1754), mRNA.	-0.01	6.58	6.15E-01	6.56E-01	lightyellow
KIF11	3832	kinesin family member 11 (KIF11), mRNA.	-0.02	7.21	3.08E-01	3.52E-01	lightyellow
KIF14	9928	kinesin family member 14 (KIF14), mRNA.	-0.01	12.27	7.09E-01	7.43E-01	lightyellow
KIF15	56992	kinesin family member 15 (KIF15), mRNA.	0.00	12.48	7.59E-01	7.89E-01	lightyellow
KIF20A	10112	kinesin family member 20A (KIF20A), mRNA.	-0.13	6.61	2.72E-16	9.64E-16	lightyellow
LBR	3930	lamin B receptor (LBR), transcript variant 1, mRNA.	0.12	6.63	8.79E-08	1.92E-07	lightyellow
MLH1	4292	mutL homolog 1, colon cancer, nonpolyposis type 2 (E. coli) (MLH1), mRNA.	-0.03	6.96	1.90E-01	2.27E-01	lightyellow

NCAPG	64151	non-SMC condensin I complex, subunit G (NCAPG), mRNA.	0.05	11.07	8.23E-04	1.32E-03	lightyellow
NDC1	55706	transmembrane protein 48 (TMEM48), mRNA.	0.06	9.18	5.59E-04	9.10E-04	lightyellow
NUSAP1	51203	nucleolar and spindle associated protein 1 (NUSAP1), transcript variant 1, mRNA.	-0.08	8.44	3.48E-07	7.32E-07	lightyellow
PEX11B	8799	peroxisomal biogenesis factor 11B (PEX11B), mRNA.	-0.21	6.22	2.62E-26	1.49E-25	lightyellow
PLK1	5347	polo-like kinase 1 (Drosophila) (PLK1), mRNA.	-0.08	7.23	1.30E-05	2.44E-05	lightyellow
PLK4	10733	polo-like kinase 4 (Drosophila) (PLK4), mRNA.	-0.02	10.59	2.07E-01	2.45E-01	lightyellow
PRC1	9055	protein regulator of cytokinesis 1 (PRC1), transcript variant 1, mRNA.	0.07	8.32	9.88E-07	2.01E-06	lightyellow
SORBS2	8470	sorbin and SH3 domain containing 2 (SORBS2), transcript variant 2, mRNA.	-0.11	6.09	1.88E-09	4.56E-09	lightyellow
SPAG5	10615	sperm associated antigen 5 (SPAG5), mRNA.	0.04	10.95	1.68E-02	2.32E-02	lightyellow
SPRYD4	283377	SPRY domain containing 4 (SPRYD4), mRNA.	0.05	8.30	1.44E-06	2.91E-06	lightyellow
STIL	6491	SCL/TAL1 interrupting locus (STIL), transcript variant 1, mRNA.	-0.01	9.22	6.70E-01	7.06E-01	lightyellow
TOP2A	7153	topoisomerase (DNA) II alpha 170kDa (TOP2A), mRNA.	-0.15	8.70	4.03E-21	1.81E-20	lightyellow
TTK	7272	TTK protein kinase (TTK), mRNA.	0.02	5.58	1.86E-01	2.22E-01	lightyellow

UBE2C	11065	ubiquitin-conjugating enzyme E2C (UBE2C), transcript variant 6, mRNA.	0.14	7.55	6.77E-10	1.69E-09	lightyellow
AAAS	8086	achalasia, adrenocortical insufficiency, alacrimia (Allgrove, triple-A) (AAAS), mRNA.	-0.04	8.33	1.67E-03	2.58E-03	magenta
ACTB	60	actin, beta (ACTB), mRNA.	1.54	10.19	2.62E-116	6.64E-113	magenta
AKR1A1	10327	aldo-keto reductase family 1, member A1 (aldehyde reductase) (AKR1A1), transcript variant 1, mRNA.	-0.09	8.78	6.81E-07	1.40E-06	magenta
AP2B1	163	adaptor-related protein complex 2, beta 1 subunit (AP2B1), transcript variant 1, mRNA.	0.19	8.00	1.41E-23	7.14E-23	magenta
AP2M1	1173	adaptor-related protein complex 2, mu 1 subunit (AP2M1), transcript variant 2, mRNA.	-0.14	10.00	7.35E-15	2.41E-14	magenta
AP3B1	8546	adaptor-related protein complex 3, beta 1 subunit (AP3B1), mRNA.	0.02	8.38	1.80E-02	2.47E-02	magenta
APH1A	51107	anterior pharynx defective 1 homolog A (C. elegans) (APH1A), transcript variant 2, mRNA.	-0.31	6.51	5.06E-55	1.01E-53	magenta
AQR	9716	aquarius homolog (mouse) (AQR), mRNA.	0.10	7.19	3.01E-09	7.24E-09	magenta
ARPP19	10776	cyclic AMP phosphoprotein, 19 kD (ARPP-19), mRNA.	0.03	7.54	3.46E-02	4.61E-02	magenta

ATG101	60673	chromosome 12 open reading frame 44 (C12orf44), mRNA.	0.04	9.56	3.22E-02	4.29E-02	magenta
ATP5D	513	ATP synthase, H+ transporting, mitochondrial F1 complex, delta subunit (ATP5D), nuclear gene encoding mitochondrial protein, transcript variant 2, mRNA.	0.94	7.92	6.19E-77	4.13E-75	magenta
ATP6V0D1	9114	ATPase, H+ transporting, lysosomal 38kDa, V0 subunit d1 (ATP6V0D1), mRNA.	-0.01	9.37	3.39E-01	3.84E-01	magenta
AURKB	9212	aurora kinase B (AURKB), mRNA.	-0.17	6.41	4.52E-22	2.12E-21	magenta
BABAM1	29086	HSPC142 protein (HSPC142), transcript variant 1, mRNA.	-0.01	6.17	6.57E-01	6.95E-01	magenta
BAD	572	BCL2-antagonist of cell death (BAD), transcript variant 2, mRNA.	0.07	6.33	3.27E-05	5.94E-05	magenta
BAG6	7917	HLA-B associated transcript 3 (BAT3), transcript variant 1, mRNA.	-0.12	8.50	1.43E-12	4.17E-12	magenta
BAK1	578	BCL2-antagonist/killer 1 (BAK1), mRNA.	0.01	8.66	6.27E-01	6.67E-01	magenta
C14orf169	79697	chromosome 14 open reading frame 169 (C14orf169), mRNA.	0.11	7.99	5.37E-11	1.43E-10	magenta
C17orf97	400566	hypothetical gene supported by AK128660 (LOC400566), mRNA.	-0.12	6.86	4.15E-12	1.17E-11	magenta

C19orf24	55009	chromosome 19 open reading frame 24 (C19orf24), mRNA.	-0.14	9.19	1.15E-26	6.64E-26	magenta
C19orf43	79002	chromosome 19 open reading frame 43 (C19orf43), mRNA.	-0.14	7.87	6.04E-18	2.34E-17	magenta
C21orf59	56683	chromosome 21 open reading frame 59 (C21orf59), mRNA.	-0.02	7.58	5.55E-02	7.19E-02	magenta
C2orf42	54980	chromosome 2 open reading frame 42 (C2orf42), mRNA.	0.36	8.95	5.75E-47	8.16E-46	magenta
C6orf106	64771	chromosome 6 open reading frame 106 (C6orf106), transcript variant 2, mRNA.	0.12	5.69	6.11E-13	1.81E-12	magenta
C9orf16	79095	chromosome 9 open reading frame 16 (C9orf16), mRNA.	-0.02	8.49	1.21E-01	1.50E-01	magenta
CAMK1D	57118	calcium/calmodulin-dependent protein kinase ID (CAMK1D), transcript variant 2, mRNA.	0.05	8.05	8.36E-04	1.34E-03	magenta
CAPNS1	826	calpain, small subunit 1 (CAPNS1), transcript variant 2, mRNA.	-0.03	6.77	2.60E-02	3.51E-02	magenta
CBX1	10951	chromobox homolog 1 (HP1 beta homolog Drosophila) (CBX1), mRNA.	0.04	8.17	2.46E-02	3.34E-02	magenta
CBX4	8535	chromobox homolog 4 (Pc class homolog, Drosophila) (CBX4), mRNA.	-0.10	7.18	8.27E-08	1.81E-07	magenta

CCDC124	115098	coiled-coil domain containing 124 (CCDC124), mRNA.	0.00	7.45	8.59E-01	8.80E-01	magenta
CD27	939	CD27 molecule (CD27), mRNA.	-0.11	10.01	1.27E-19	5.36E-19	magenta
CDK2AP2	10263	CDK2-associated protein 2 (CDK2AP2), mRNA.	-0.15	6.45	1.11E-18	4.45E-18	magenta
CHCHD3	54927	coiled-coil-helix-coiled-coil-helix domain containing 3 (CHCHD3), mRNA.	-0.22	6.80	2.13E-31	1.46E-30	magenta
CINP	51550	cyclin-dependent kinase 2-interacting protein (CINP), mRNA.	-0.06	6.38	1.02E-02	1.44E-02	magenta
CLTA	1211	clathrin, light chain (Lca) (CLTA), transcript variant 1, mRNA.	0.22	8.83	2.27E-16	8.10E-16	magenta
COPE	11316	coatomer protein complex, subunit epsilon (COPE), transcript variant 2, mRNA.	-0.06	7.34	2.55E-05	4.69E-05	magenta
COPG2	26958	coatomer protein complex, subunit gamma 2 (COPG2), mRNA.	-0.12	9.38	3.27E-08	7.36E-08	magenta
CORO1A	11151	coronin, actin binding protein, 1A (CORO1A), mRNA.	-0.11	6.42	3.46E-10	8.79E-10	magenta
CPS1	1373	carbamoyl-phosphate synthetase 1, mitochondrial (CPS1), mRNA.	0.01	7.30	2.71E-01	3.13E-01	magenta
CPSF3L	54973	cleavage and polyadenylation specific factor 3-like (CPSF3L), mRNA.	-0.04	9.41	6.79E-03	9.86E-03	magenta
CSNK2B	1460	casein kinase 2, beta polypeptide (CSNK2B), mRNA.	0.01	9.68	4.53E-01	4.99E-01	magenta

CTSH	1512	cathepsin H (CTSH), transcript variant 2, mRNA.	0.06	8.08	9.64E-04	1.53E-03	magenta
DCPS	28960	decapping enzyme, scavenger (DCPS), mRNA.	0.05	5.79	5.50E-05	9.78E-05	magenta
DDX41	51428	DEAD (Asp-Glu-Ala-Asp) box polypeptide 41 (DDX41), mRNA.	0.08	6.14	1.95E-05	3.62E-05	magenta
DFFA	1676	DNA fragmentation factor, 45kDa, alpha polypeptide (DFFA), transcript variant 1, mRNA.	0.23	7.56	7.55E-38	7.01E-37	magenta
DHPS	1725	deoxyhypusine synthase (DHPS), transcript variant 3, mRNA.	-0.05	7.61	1.15E-03	1.80E-03	magenta
DLST	1743	dihydrolipoamide S-succinyltransferase (E2 component of 2-oxo-glutarate complex) (DLST), mRNA.	0.03	6.62	3.71E-02	4.92E-02	magenta
DSCC1	79075	defective in sister chromatid cohesion homolog 1 (S. cerevisiae) (DCC1), mRNA.	-0.04	6.37	5.90E-02	7.61E-02	magenta
DTD1	92675	D-tyrosyl-tRNA deacylase 1 homolog (S. cerevisiae) (DTD1), mRNA.	0.03	6.31	4.55E-02	5.97E-02	magenta
DUSP3	1845	dual specificity phosphatase 3 (vaccinia virus phosphatase VH1-related) (DUSP3), mRNA.	-0.01	9.91	3.75E-01	4.20E-01	magenta

EEF1D	1936	eukaryotic translation elongation factor 1 delta (guanine nucleotide exchange protein) (EEF1D), transcript variant 2, mRNA.	-0.01	10.78	1.19E-01	1.47E-01	magenta
EIF3G	8666	eukaryotic translation initiation factor 3, subunit 4 delta, 44kDa (EIF3S4), mRNA.	0.17	6.53	2.12E-19	8.79E-19	magenta
EIF3H	8667	eukaryotic translation initiation factor 3, subunit 3 gamma, 40kDa (EIF3S3), mRNA.	0.16	6.24	1.73E-22	8.26E-22	magenta
EIF4EBP1	1978	eukaryotic translation initiation factor 4E binding protein 1 (EIF4EBP1), mRNA.	0.04	8.59	1.41E-02	1.97E-02	magenta
EIF6	3692	integrin beta 4 binding protein (ITGB4BP), transcript variant 4, mRNA.	-0.02	5.51	1.75E-01	2.10E-01	magenta
ELOF1	84337	elongation factor 1 homolog (S. cerevisiae) (ELOF1), mRNA.	0.09	6.60	5.19E-08	1.15E-07	magenta
EXOSC4	54512	exosome component 4 (EXOSC4), mRNA.	-0.04	8.12	1.62E-04	2.77E-04	magenta
FAHD2B	731002	PREDICTED: similar to fumarylacetoacetate hydrolase domain containing 2A (LOC731002), mRNA.	-0.01	7.70	6.45E-01	6.83E-01	magenta
FAM104A	84923	family with sequence similarity 104, member A (FAM104A), mRNA.	0.01	5.54	6.67E-01	7.04E-01	magenta

FAM175B	23172	KIAA0157 (KIAA0157), mRNA.	-0.04	5.65	6.89E-03	9.99E-03	magenta
FAM195B	348262	hypothetical protein LOC348262 (LOC348262), mRNA.	-0.06	8.66	5.88E-06	1.14E-05	magenta
FARSA	2193	phenylalanyl-tRNA synthetase, alpha subunit (FARSA), mRNA.	-0.03	5.44	7.43E-02	9.47E-02	magenta
FASTK	10922	Fas-activated serine/threonine kinase (FASTK), transcript variant 1, mRNA.	-0.22	7.03	1.55E-42	1.78E-41	magenta
FKBP8	23770	FK506 binding protein 8, 38kDa (FKBP8), mRNA.	0.07	8.38	6.20E-06	1.20E-05	magenta
FLOT1	10211	flotillin 1 (FLOT1), mRNA.	-0.46	10.23	2.12E-47	3.07E-46	magenta
GAPDH	2597	glyceraldehyde-3-phosphate dehydrogenase (GAPDH), mRNA.	-0.01	5.61	5.16E-01	5.60E-01	magenta
GNAS	2778	GNAS complex locus (GNAS), transcript variant 1, mRNA.	0.03	7.57	2.00E-02	2.74E-02	magenta
GNB2	2783	guanine nucleotide binding protein (G protein), beta polypeptide 2 (GNB2), mRNA.	0.46	6.20	6.83E-54	1.28E-52	magenta
GRK6	2870	G protein-coupled receptor kinase 6 (GRK6), transcript variant 2, mRNA.	-0.14	6.81	1.16E-23	5.90E-23	magenta
GSPT1	2935	G1 to S phase transition 1 (GSPT1), mRNA.	0.05	10.41	5.86E-04	9.52E-04	magenta
HADH	3033	hydroxyacyl-Coenzyme A dehydrogenase (HADH), nuclear gene encoding mitochondrial protein, mRNA.	-0.09	6.84	1.21E-08	2.81E-08	magenta

HDGF	3068	hepatoma-derived growth factor (high-mobility group protein 1-like) (HDGF), mRNA.	-0.05	8.25	1.10E-02	1.56E-02	magenta
HDHD3	81932	haloacid dehalogenase-like hydrolase domain containing 3 (HDHD3), mRNA.	0.11	6.41	2.89E-07	6.11E-07	magenta
HLA-A	3105	major histocompatibility complex, class I, A (HLA-A), mRNA.	0.26	7.59	5.41E-34	4.12E-33	magenta
HLA-E	3133	major histocompatibility complex, class I, E (HLA-E), mRNA.	0.05	8.94	1.02E-02	1.44E-02	magenta
IDH2	3418	isocitrate dehydrogenase 2 (NADP+), mitochondrial (IDH2), mRNA.	-0.05	8.85	9.04E-04	1.44E-03	magenta
IDH3G	3421	isocitrate dehydrogenase 3 (NAD+) gamma (IDH3G), nuclear gene encoding mitochondrial protein, transcript variant 1, mRNA.	0.01	6.35	5.32E-01	5.77E-01	magenta
IFRD2	7866	interferon-related developmental regulator 2 (IFRD2), mRNA.	-0.28	7.05	5.05E-40	5.06E-39	magenta
ILDR1	286676	immunoglobulin-like domain containing receptor 1 (ILDR1), mRNA.	-0.14	8.35	3.45E-21	1.55E-20	magenta
KARS	3735	lysyl-tRNA synthetase (KARS), mRNA.	-0.02	10.76	6.81E-02	8.74E-02	magenta
KIAA1191	57179	KIAA1191 (KIAA1191), transcript variant 3, mRNA.	-0.05	5.58	5.20E-04	8.49E-04	magenta

KIF22	3835	kinesin family member 22 (KIF22), mRNA.	0.00	12.07	9.39E-01	9.48E-01	magenta
KLHL3	26249	kelch-like 3 (Drosophila) (KLHL3), mRNA.	0.05	9.05	2.54E-02	3.44E-02	magenta
KRCC1	51315	lysine-rich coiled-coil 1 (KRCC1), mRNA.	-0.04	8.87	1.91E-04	3.25E-04	magenta
LPAR5	57121	G protein-coupled receptor 92 (GPR92), mRNA.	0.00	9.15	9.76E-01	9.80E-01	magenta
LYST	1130	lysosomal trafficking regulator (LYST), transcript variant 1, mRNA.	0.01	7.00	4.42E-01	4.88E-01	magenta
MAF1	84232	MAF1 homolog (S. cerevisiae) (MAF1), mRNA.	0.07	8.66	4.28E-10	1.08E-09	magenta
MAN2B1	4125	mannosidase, alpha, class 2B, member 1 (MAN2B1), mRNA.	-0.05	7.38	3.57E-03	5.33E-03	magenta
MAP2K2	5605	mitogen-activated protein kinase kinase 2 (MAP2K2), mRNA.	-0.05	7.29	9.57E-04	1.52E-03	magenta
MBD3	53615	methyl-CpG binding domain protein 3 (MBD3), mRNA.	0.14	9.40	3.98E-15	1.32E-14	magenta
MCM5	4174	MCM5 minichromosome maintenance deficient 5, cell division cycle 46 (S. cerevisiae) (MCM5), mRNA.	0.00	7.50	9.67E-01	9.72E-01	magenta
MIPEP	4285	mitochondrial intermediate peptidase (MIPEP), nuclear gene encoding mitochondrial protein, mRNA.	-0.16	7.51	3.61E-36	3.06E-35	magenta
MKRN1	23608	makorin, ring finger protein, 1 (MKRN1), mRNA.	-0.21	6.66	4.83E-33	3.55E-32	magenta

MPV17	4358	MpV17 mitochondrial inner membrane protein (MPV17), nuclear gene encoding mitochondrial protein, mRNA.	0.14	8.45	1.83E-18	7.26E-18	magenta
MRPL12	6182	mitochondrial ribosomal protein L12 (MRPL12), nuclear gene encoding mitochondrial protein, mRNA.	-0.10	9.99	7.40E-15	2.43E-14	magenta
MRPL28	10573	mitochondrial ribosomal protein L28 (MRPL28), nuclear gene encoding mitochondrial protein, mRNA.	-0.11	6.59	1.66E-16	5.97E-16	magenta
MRPS18B	28973	mitochondrial ribosomal protein S18B (MRPS18B), nuclear gene encoding mitochondrial protein, mRNA.	0.13	8.80	2.33E-08	5.28E-08	magenta
MRTO4	51154	mRNA turnover 4 homolog (S. cerevisiae) (MRTO4), mRNA.	-0.14	7.43	4.15E-19	1.70E-18	magenta
MSH3	4437	mutS homolog 3 (E. coli) (MSH3), mRNA.	-0.09	8.68	9.48E-09	2.21E-08	magenta
MTFP1	51537	mitochondrial protein 18 kDa (MTP18), nuclear gene encoding mitochondrial protein, transcript variant 1, mRNA.	-0.30	6.98	1.47E-56	3.20E-55	magenta
MVD	4597	mevalonate (diphospho) decarboxylase (MVD), mRNA.	-0.49	6.91	1.83E-57	4.27E-56	magenta

MVP	9961	major vault protein (MVP), transcript variant 2, mRNA.	0.01	7.29	5.47E-01	5.91E-01	magenta
MYLIP	29116	myosin regulatory light chain interacting protein (MYLIP), mRNA.	-0.88	8.73	2.53E-61	7.56E-60	magenta
NA	283820	NODAL modulator 2 (NOMO2), transcript variant 2, mRNA.	0.74	8.46	1.04E-83	1.22E-81	magenta
NA	51491	hypothetical protein HSPC111 (HSPC111), mRNA.	0.13	7.10	5.50E-13	1.64E-12	magenta
NA	440400	similar to DNA segment, Chr 11, Brigham & Womens Genetics 0434 expressed (MGC71993), mRNA.	0.08	7.64	1.03E-09	2.53E-09	magenta
NA	1415	crystallin, beta B2 (CRYBB2), mRNA.	-0.05	6.64	4.63E-03	6.84E-03	magenta
NA	731986	PREDICTED: similar to cytochrome P450 monooxygenase CYP2T1 (LOC731986), mRNA.	0.04	7.26	1.78E-02	2.46E-02	magenta
NA	84833	upregulated during skeletal muscle growth 5 homolog (mouse) (USMG5), mRNA.	0.03	5.44	3.77E-02	4.99E-02	magenta
NDUFA10	732160	PREDICTED: similar to NADH dehydrogenase (ubiquinone) 1 alpha subcomplex, 10, 42kDa precursor (LOC732160), mRNA.	0.04	8.94	2.81E-03	4.23E-03	magenta

NFATC3	4775	nuclear factor of activated T-cells, cytoplasmic, calcineurin-dependent 3 (NFATC3), transcript variant 2, mRNA.	0.01	6.83	4.73E-01	5.18E-01	magenta
NME1-NME2	654364	NM23-LV (NME1-NME2), mRNA.	0.14	6.74	4.55E-17	1.69E-16	magenta
NOB1	28987	NIN1/RPN12 binding protein 1 homolog (S. cerevisiae) (NOB1), mRNA.	-0.20	8.30	1.51E-19	6.31E-19	magenta
NONO	4841	non-POU domain containing, octamer-binding (NONO), mRNA.	-0.06	7.75	5.98E-05	1.06E-04	magenta
NUBP1	4682	nucleotide binding protein 1 (MinD homolog, E. coli) (NUBP1), mRNA.	-0.13	10.26	1.75E-19	7.29E-19	magenta
NUDC	10726	nuclear distribution gene C homolog (A. nidulans) (NUDC), mRNA.	-0.03	7.52	4.46E-02	5.86E-02	magenta
NUDT22	84304	nudix (nucleoside diphosphate linked moiety X)-type motif 22 (NUDT22), mRNA.	0.06	8.34	1.15E-04	1.98E-04	magenta
OTP	23440	orthopedia homeobox (OTP), mRNA.	-0.39	8.95	1.81E-53	3.24E-52	magenta
OXA1L	5018	oxidase (cytochrome c) assembly 1-like (OXA1L), mRNA.	-0.08	7.29	3.01E-06	5.94E-06	magenta
PAGR1	79447	chromosome 16 open reading frame 53 (C16orf53), mRNA.	0.07	7.67	4.91E-07	1.02E-06	magenta
PAK1	5058	p21/Cdc42/Rac1-activated kinase 1 (STE20 homolog, yeast) (PAK1), mRNA.	-0.04	9.12	1.43E-02	2.00E-02	magenta

PCGF2	7703	polycomb group ring finger 2 (PCGF2), mRNA.	0.05	7.81	3.29E-04	5.47E-04	magenta
PGLS	25796	6-phosphogluconolactonase (PGLS), mRNA.	0.01	6.99	3.67E-01	4.12E-01	magenta
PHACTR4	65979	phosphatase and actin regulator 4 (PHACTR4), transcript variant 1, mRNA.	0.07	6.70	1.40E-05	2.63E-05	magenta
PLP2	5355	proteolipid protein 2 (colonic epithelium-enriched) (PLP2), mRNA.	0.07	10.20	3.52E-05	6.38E-05	magenta
PMVK	10654	phosphomevalonate kinase (PMVK), mRNA.	-0.11	6.26	1.25E-09	3.06E-09	magenta
POLD4	57804	polymerase (DNA-directed), delta 4 (POLD4), mRNA.	-0.01	6.65	5.69E-01	6.13E-01	magenta
PPIH	10465	peptidylprolyl isomerase H (cyclophilin H) (PPIH), mRNA.	0.01	5.63	5.07E-01	5.52E-01	magenta
PPP2R5A	5525	protein phosphatase 2, regulatory subunit B', alpha isoform (PPP2R5A), mRNA.	0.06	10.31	3.53E-05	6.40E-05	magenta
PQLC3	130814	PQ loop repeat containing 3 (PQLC3), mRNA.	0.00	9.30	9.29E-01	9.40E-01	magenta
PRCC	5546	papillary renal cell carcinoma (translocation-associated) (PRCC), transcript variant 1, mRNA.	0.00	6.20	7.73E-01	8.02E-01	magenta
PRKCSH	5589	protein kinase C substrate 80K-H (PRKCSH), transcript variant 1, mRNA.	0.04	7.42	6.93E-02	8.87E-02	magenta

PSENEN	55851	presenilin enhancer 2 homolog (C. elegans) (PSENEN), mRNA.	-0.21	10.32	2.01E-35	1.64E-34	magenta
PSMD3	5709	proteasome (prosome, macropain) 26S subunit, non-ATPase, 3 (PSMD3), mRNA.	-0.01	9.84	3.57E-01	4.03E-01	magenta
PSMD4	5710	proteasome (prosome, macropain) 26S subunit, non-ATPase, 4 (PSMD4), transcript variant 2, mRNA.	-0.06	10.86	7.38E-07	1.51E-06	magenta
PTGES2	80142	prostaglandin E synthase 2 (PTGES2), transcript variant 1, mRNA.	0.03	10.01	8.70E-03	1.25E-02	magenta
PTPRCAP	5790	protein tyrosine phosphatase, receptor type, C-associated protein (PTPRCAP), mRNA.	0.40	8.13	5.85E-32	4.13E-31	magenta
PUSL1	126789	pseudouridylate synthase-like 1 (PUSL1), mRNA.	0.02	10.46	9.37E-02	1.18E-01	magenta
QDPR	5860	quinoid dihydropteridine reductase (QDPR), mRNA.	0.25	7.02	6.57E-32	4.63E-31	magenta
RAB5B	5869	RAB5B, member RAS oncogene family (RAB5B), mRNA.	-0.05	5.85	1.02E-03	1.62E-03	magenta
RALY	22913	RNA binding protein, autoantigenic (hnRNP-associated with lethal yellow homolog (mouse)) (RALY), transcript variant 1, mRNA.	-0.13	7.38	1.07E-18	4.30E-18	magenta

RANGRF	29098	RAN guanine nucleotide release factor (RANGNRF), mRNA.	-0.07	5.93	1.93E-04	3.28E-04	magenta
RBM17	84991	RNA binding motif protein 17 (RBM17), mRNA.	0.02	5.90	1.08E-01	1.35E-01	magenta
RHBDD2	57414	rhomboid domain containing 2 (RHBDD2), transcript variant 2, mRNA.	-0.01	11.81	7.52E-01	7.82E-01	magenta
RHOF	54509	ras homolog gene family, member F (in filopodia) (RHOF), mRNA.	0.60	9.97	4.46E-79	3.84E-77	magenta
RNF126	55658	ring finger protein 126 (RNF126), transcript variant 2, mRNA.	0.23	8.63	1.40E-35	1.15E-34	magenta
RNH1	6050	ribonuclease/angiogenin inhibitor 1 (RNH1), transcript variant 8, mRNA.	0.37	7.63	3.64E-48	5.47E-47	magenta
RNPS1	10921	RNA binding protein S1, serine-rich domain (RNPS1), transcript variant 1, mRNA.	-0.19	5.79	6.92E-18	2.67E-17	magenta
RPL4	6124	ribosomal protein L4 (RPL4), mRNA.	0.05	9.18	9.46E-04	1.50E-03	magenta
RPL6	6128	ribosomal protein L6 (RPL6), transcript variant 2, mRNA.	0.14	8.24	8.86E-19	3.56E-18	magenta
RPS2	440589	PREDICTED: similar to ribosomal protein S2, transcript variant 3 (LOC440589), mRNA.	0.05	7.76	1.80E-05	3.37E-05	magenta
RPS29	6235	ribosomal protein S29 (RPS29), transcript variant 1, mRNA.	-0.12	7.52	1.39E-16	5.04E-16	magenta

RSL1D1	26156	ribosomal L1 domain containing 1 (RSL1D1), mRNA.	-0.09	7.07	6.43E-08	1.42E-07	magenta
RUVBL1	8607	RuvB-like 1 (E. coli) (RUVBL1), mRNA.	-0.43	6.69	1.90E-59	5.16E-58	magenta
RUVBL2	10856	RuvB-like 2 (E. coli) (RUVBL2), mRNA.	0.10	6.60	1.40E-11	3.84E-11	magenta
RWDD4	201965	RWD domain containing 4A (RWDD4A), mRNA.	0.08	8.77	1.09E-11	3.02E-11	magenta
SAAL1	113174	serum amyloid A-like 1 (SAAL1), mRNA.	0.36	9.88	1.67E-54	3.24E-53	magenta
SAMM50	25813	sorting and assembly machinery component 50 homolog (S. cerevisiae) (SAMM50), mRNA.	0.31	8.03	3.34E-32	2.40E-31	magenta
SCRN1	9805	secernin 1 (SCRN1), mRNA.	-0.06	7.93	2.05E-03	3.15E-03	magenta
SEC13	6396	SEC13 homolog (S. cerevisiae) (SEC13), mRNA.	0.08	8.65	1.23E-12	3.59E-12	magenta
SEC61A1	29927	Sec61 alpha 1 subunit (S. cerevisiae) (SEC61A1), mRNA.	-0.16	8.04	9.99E-16	3.45E-15	magenta
SH3BGRL3	83442	SH3 domain binding glutamic acid-rich protein like 3 (SH3BGRL3), mRNA.	-0.09	6.78	1.86E-05	3.46E-05	magenta
SIAH1	6477	seven in absentia homolog 1 (Drosophila) (SIAH1), transcript variant 2, mRNA.	-0.10	8.18	6.61E-11	1.75E-10	magenta
SIAH2	6478	seven in absentia homolog 2 (Drosophila) (SIAH2), mRNA.	0.01	8.93	4.85E-01	5.30E-01	magenta

SLC25A1	6576	solute carrier family 25 (mitochondrial carrier; citrate transporter), member 1 (SLC25A1), mRNA.	0.00	8.16	9.11E-01	9.25E-01	magenta
SLC25A23	79085	solute carrier family 25 (mitochondrial carrier; phosphate carrier), member 23 (SLC25A23), mRNA.	0.19	7.29	1.04E-24	5.60E-24	magenta
SLC35A4	113829	solute carrier family 35, member A4 (SLC35A4), mRNA.	-0.06	7.72	1.50E-08	3.46E-08	magenta
SNAP29	9342	synaptosomal-associated protein, 29kDa (SNAP29), mRNA.	-0.29	8.45	8.43E-52	1.42E-50	magenta
SNRNP25	79622	chromosome 16 open reading frame 33 (C16orf33), mRNA.	0.01	11.43	4.75E-01	5.20E-01	magenta
SNRPC	6631	small nuclear ribonucleoprotein polypeptide C (SNRPC), mRNA.	-0.08	7.91	8.36E-05	1.47E-04	magenta
SNX19	399979	sorting nexin 19 (SNX19), mRNA.	0.39	7.74	4.03E-55	8.11E-54	magenta
SRM	6723	spermidine synthase (SRM), mRNA.	-0.39	7.69	1.13E-47	1.66E-46	magenta
STUB1	10273	STIP1 homology and U-box containing protein 1 (STUB1), mRNA.	-0.05	8.29	1.94E-03	2.98E-03	magenta
SUMO3	6612	SMT3 suppressor of mif two 3 homolog 3 (S. cerevisiae) (SUMO3), mRNA.	0.07	10.50	2.01E-07	4.30E-07	magenta

TAF10	6881	TAF10 RNA polymerase II, TATA box binding protein (TBP)-associated factor, 30kDa (TAF10), mRNA.	0.07	8.01	2.11E-08	4.79E-08	magenta
TALDO1	6888	transaldolase 1 (TALDO1), mRNA.	0.06	9.05	2.55E-04	4.28E-04	magenta
TCHP	84260	trichoplein, keratin filament binding (TCHP), mRNA.	0.16	8.46	8.22E-16	2.85E-15	magenta
TERF2IP	54386	telomeric repeat binding factor 2, interacting protein (TERF2IP), mRNA.	-0.31	8.54	2.75E-56	5.96E-55	magenta
TFEB	7942	transcription factor EB (TFEB), mRNA.	0.08	8.18	5.15E-06	9.98E-06	magenta
THOC6	79228	THO complex 6 homolog (Drosophila) (THOC6), mRNA.	0.14	7.38	6.99E-25	3.78E-24	magenta
TMEM165	55858	transmembrane protein 165 (TMEM165), mRNA.	-0.03	8.56	4.21E-02	5.54E-02	magenta
TMEM9	252839	transmembrane protein 9 (TMEM9), mRNA.	0.19	8.65	6.21E-21	2.76E-20	magenta
TNFRSF13B	23495	tumor necrosis factor receptor superfamily, member 13B (TNFRSF13B), mRNA.	-0.03	8.49	3.37E-03	5.04E-03	magenta
TNRC6B	23112	trinucleotide repeat containing 6B (TNRC6B), transcript variant 2, mRNA.	-0.10	7.51	3.60E-07	7.55E-07	magenta
TOPBP1	11073	topoisomerase (DNA) II binding protein 1 (TOPBP1), mRNA.	0.16	8.17	1.17E-21	5.39E-21	magenta
TOR3A	64222	torsin family 3, member A (TOR3A), mRNA.	0.03	9.63	1.67E-02	2.30E-02	magenta

TRAP1	10131	TNF receptor-associated protein 1 (TRAP1), mRNA.	-0.11	9.76	9.66E-15	3.16E-14	magenta
TSTA3	7264	tissue specific transplantation antigen P35B (TSTA3), mRNA.	0.08	10.86	1.84E-15	6.22E-15	magenta
TUBB4B	10383	tubulin, beta 2C (TUBB2C), mRNA.	-0.03	9.76	9.72E-02	1.22E-01	magenta
TUBB8	56604	tubulin, beta polypeptide 4, member Q (TUBB4Q), mRNA.	0.05	6.23	5.77E-04	9.38E-04	magenta
TUBB8	347688	tubulin, beta 8 (TUBB8), mRNA.	0.02	7.34	1.62E-01	1.95E-01	magenta
TUBGCP2	10844	tubulin, gamma complex associated protein 2 (TUBGCP2), mRNA.	-0.02	9.84	4.79E-02	6.26E-02	magenta
TUFM	7284	Tu translation elongation factor, mitochondrial (TUFM), mRNA.	0.07	7.32	2.93E-05	5.36E-05	magenta
TWF2	11344	twinfilin, actin-binding protein, homolog 2 (Drosophila) (TWF2), mRNA.	-0.11	6.33	2.25E-13	6.84E-13	magenta
TXNDC12	51060	thioredoxin domain containing 12 (endoplasmic reticulum) (TXNDC12), mRNA.	0.02	8.91	1.30E-01	1.59E-01	magenta
U2AF1	7307	U2 small nuclear RNA auxiliary factor 1 (U2AF1), transcript variant a, mRNA.	0.05	10.32	2.97E-02	3.97E-02	magenta
UBE2N	7334	ubiquitin-conjugating enzyme E2N (UBC13 homolog, yeast) (UBE2N), mRNA.	-0.01	8.25	2.83E-01	3.25E-01	magenta
UCK2	7371	uridine-cytidine kinase 2 (UCK2), mRNA.	0.03	7.62	1.76E-02	2.42E-02	magenta

UNC45A	55898	unc-45 homolog A (C. elegans) (UNC45A), transcript variant 2, mRNA.	0.10	6.87	2.81E-07	5.95E-07	magenta
UQCRC1	7384	ubiquinol-cytochrome c reductase core protein I (UQCRC1), mRNA.	-0.29	8.19	8.14E-34	6.17E-33	magenta
USP48	84196	ubiquitin specific peptidase 48 (USP48), transcript variant 2, mRNA.	0.05	9.32	3.17E-05	5.78E-05	magenta
USP7	7874	ubiquitin specific peptidase 7 (herpes virus-associated) (USP7), mRNA.	0.06	10.18	1.26E-06	2.54E-06	magenta
VAC14	55697	Vac14 homolog (S. cerevisiae) (VAC14), mRNA.	0.02	6.96	4.26E-01	4.72E-01	magenta
VPS28	51160	vacuolar protein sorting 28 homolog (S. cerevisiae) (VPS28), transcript variant 2, mRNA.	0.03	7.94	2.30E-01	2.70E-01	magenta
VPS37C	55048	vacuolar protein sorting 37 homolog C (S. cerevisiae) (VPS37C), mRNA.	0.03	11.48	2.08E-01	2.47E-01	magenta
WIBG	84305	within bgcn homolog (Drosophila) (WIBG), mRNA.	0.06	7.12	8.06E-04	1.29E-03	magenta
WRNIP1	56897	Werner helicase interacting protein 1 (WRNIP1), transcript variant 2, mRNA.	-0.03	8.41	8.70E-03	1.25E-02	magenta
WWP1	11059	WW domain containing E3 ubiquitin protein ligase 1 (WWP1), mRNA.	-0.15	6.01	9.65E-18	3.70E-17	magenta

YIF1B	90522	Yip1 interacting factor homolog B (S. cerevisiae) (YIF1B), transcript variant 3, mRNA.	-0.35	7.71	3.10E-56	6.67E-55	magenta
ZDHHC12	84885	zinc finger, DHHC-type containing 12 (ZDHHC12), mRNA.	0.13	7.24	2.63E-13	7.96E-13	magenta
ZFYVE26	23503	zinc finger, FYVE domain containing 26 (ZFYVE26), mRNA.	-0.01	8.63	3.91E-01	4.36E-01	magenta
ADAM15	8751	ADAM metallopeptidase domain 15 (metargidin) (ADAM15), transcript variant 1, mRNA.	-0.05	9.41	2.19E-03	3.34E-03	midnightblue
ADCK2	90956	aarF domain containing kinase 2 (ADCK2), mRNA.	0.30	7.63	2.55E-44	3.15E-43	midnightblue
ADIPOR1	51094	adiponectin receptor 1 (ADIPOR1), mRNA.	-0.20	5.68	5.64E-20	2.41E-19	midnightblue
AKR7A2	8574	aldo-keto reductase family 7, member A2 (aflatoxin aldehyde reductase) (AKR7A2), mRNA.	0.18	8.13	2.71E-27	1.61E-26	midnightblue
APBB1IP	54518	amyloid beta (A4) precursor protein-binding, family B, member 1 interacting protein (APBB1IP), mRNA.	0.01	6.35	5.05E-01	5.50E-01	midnightblue
ARHGEF18	23370	rho/rac guanine nucleotide exchange factor (GEF) 18 (ARHGEF18), mRNA.	0.38	7.81	5.67E-43	6.59E-42	midnightblue

ATP6V0E2	155066	ATPase, H+ transporting V0 subunit e2 (ATP6V0E2), mRNA.	0.03	7.34	1.40E-01	1.71E-01	midnightblue
B4GAT1	11041	UDP-GlcNAc:betaGal beta-1,3-N-acetylglucosaminyltransferase 1 (B3GNT1), mRNA.	-0.02	8.66	1.24E-01	1.53E-01	midnightblue
BCKDK	10295	branched chain ketoacid dehydrogenase kinase (BCKDK), mRNA.	0.14	6.84	4.46E-17	1.66E-16	midnightblue
BID	637	BH3 interacting domain death agonist (BID), transcript variant 1, mRNA.	-0.04	7.39	8.06E-03	1.16E-02	midnightblue
BTBD2	55643	BTB (POZ) domain containing 2 (BTBD2), mRNA.	-0.12	7.71	6.69E-14	2.10E-13	midnightblue
C17orf53	78995	chromosome 17 open reading frame 53 (C17orf53), mRNA.	0.05	7.75	1.22E-03	1.91E-03	midnightblue
C19orf54	284325	chromosome 19 open reading frame 54 (C19orf54), mRNA.	-0.11	7.64	1.36E-06	2.74E-06	midnightblue
CALM3	808	calmodulin 3 (phosphorylase kinase, delta) (CALM3), mRNA.	-0.34	6.24	1.24E-39	1.22E-38	midnightblue
CAPN1	823	calpain 1, (mu/I) large subunit (CAPN1), mRNA.	0.14	8.23	1.31E-12	3.81E-12	midnightblue
CCDC25	55246	coiled-coil domain containing 25 (CCDC25), mRNA.	-0.02	9.23	2.09E-01	2.47E-01	midnightblue
CCDC92	80212	coiled-coil domain containing 92 (CCDC92), mRNA.	-0.06	10.23	2.29E-06	4.56E-06	midnightblue
CCND3	896	cyclin D3 (CCND3), mRNA.	0.19	9.48	1.95E-36	1.68E-35	midnightblue

CCNY	219771	cyclin Y (CCNY), transcript variant 1, mRNA.	0.02	9.38	3.75E-01	4.21E-01	midnightblue
CD81	975	CD81 molecule (CD81), mRNA.	-0.13	8.58	4.67E-19	1.91E-18	midnightblue
CENPB	1059	centromere protein B, 80kDa (CENPB), mRNA.	0.27	10.42	6.82E-43	7.90E-42	midnightblue
CENPBD1	92806	hypothetical protein MGC16385 (MGC16385), mRNA.	-0.19	6.40	9.46E-18	3.63E-17	midnightblue
CFDP1	10428	craniofacial development protein 1 (CFDP1), mRNA.	0.01	11.34	4.66E-01	5.12E-01	midnightblue
CHIC2	26511	cysteine-rich hydrophobic domain 2 (CHIC2), mRNA.	-0.24	6.69	2.76E-25	1.51E-24	midnightblue
CSNK1D	1453	casein kinase 1, delta (CSNK1D), transcript variant 1, mRNA.	0.10	7.93	1.78E-08	4.07E-08	midnightblue
CUTC	51076	cutC copper transporter homolog (E. coli) (CUTC), mRNA.	0.04	5.63	1.00E-02	1.42E-02	midnightblue
DIS3L	115752	DIS3 mitotic control homolog (S. cerevisiae)-like (DIS3L), mRNA.	-0.01	8.15	3.39E-01	3.84E-01	midnightblue
DPH3	285381	DPH3, KTI11 homolog (S. cerevisiae) (DPH3), transcript variant 1, mRNA.	-0.17	8.62	8.49E-25	4.58E-24	midnightblue
EAF2	55840	ELL associated factor 2 (EAF2), mRNA.	0.10	9.04	1.28E-11	3.53E-11	midnightblue
EHD1	10938	EH-domain containing 1 (EHD1), mRNA.	2.15	6.88	3.62E-89	6.57E-87	midnightblue
EMILIN2	84034	elastin microfibril interfacer 2 (EMILIN2), mRNA.	-0.10	8.15	1.18E-15	4.04E-15	midnightblue

ENKD1	84080	chromosome 16 open reading frame 48 (C16orf48), mRNA.	0.02	11.39	1.74E-01	2.08E-01	midnightblue
ESD	2098	esterase D/formylglutathione hydrolase (ESD), mRNA.	0.11	8.56	1.44E-08	3.31E-08	midnightblue
FAM110A	83541	chromosome 20 open reading frame 55 (C20orf55), transcript variant 2, mRNA.	-0.04	11.41	1.64E-03	2.54E-03	midnightblue
FAM65A	79567	family with sequence similarity 65, member A (FAM65A), mRNA.	0.20	7.89	1.19E-24	6.39E-24	midnightblue
FANCG	2189	Fanconi anemia, complementation group G (FANCG), mRNA.	-0.04	8.14	1.43E-04	2.46E-04	midnightblue
FN3KRP	79672	fructosamine-3-kinase-related protein (FN3KRP), mRNA.	0.81	6.81	2.22E-85	2.90E-83	midnightblue
GLTSCR2	29997	glioma tumor suppressor candidate region gene 2 (GLTSCR2), mRNA.	0.09	8.25	3.53E-13	1.06E-12	midnightblue
HES6	55502	hairy and enhancer of split 6 (Drosophila) (HES6), mRNA.	0.13	8.31	9.87E-17	3.61E-16	midnightblue
HPS6	79803	Hermansky-Pudlak syndrome 6 (HPS6), mRNA.	-0.08	8.13	5.85E-09	1.39E-08	midnightblue
IL4R	3566	interleukin 4 receptor (IL4R), transcript variant 1, mRNA.	-0.10	7.67	1.74E-15	5.91E-15	midnightblue
IMPDH1	3614	IMP (inosine monophosphate) dehydrogenase 1 (IMPDH1), transcript variant 1, mRNA.	-0.22	6.32	1.18E-27	7.14E-27	midnightblue

IRF5	3663	interferon regulatory factor 5 (IRF5), transcript variant 1, mRNA.	0.20	6.45	2.82E-34	2.19E-33	midnightblue
ISOC1	51015	isochorismatase domain containing 1 (ISOC1), mRNA.	-0.14	6.99	1.99E-15	6.71E-15	midnightblue
KATNB1	10300	katanin p80 (WD repeat containing) subunit B 1 (KATNB1), mRNA.	0.06	7.19	1.16E-05	2.20E-05	midnightblue
KCTD17	79734	potassium channel tetramerisation domain containing 17 (KCTD17), mRNA.	-0.07	5.48	5.56E-05	9.89E-05	midnightblue
KIF2C	11004	kinesin family member 2C (KIF2C), mRNA.	-0.17	7.56	3.53E-21	1.59E-20	midnightblue
LEF1	51176	lymphoid enhancer-binding factor 1 (LEF1), mRNA.	0.26	5.45	1.77E-25	9.80E-25	midnightblue
LRRC20	55222	leucine rich repeat containing 20 (LRRC20), transcript variant 3, mRNA.	-0.17	7.95	9.43E-19	3.79E-18	midnightblue
LRRC41	10489	leucine rich repeat containing 41 (LRRC41), mRNA.	0.10	6.19	1.49E-10	3.86E-10	midnightblue
MAN1B1	11253	mannosidase, alpha, class 1B, member 1 (MAN1B1), mRNA.	0.04	9.19	2.92E-02	3.91E-02	midnightblue
MANEAL	149175	mannosidase, endo-alpha-like (MANEAL), transcript variant 1, mRNA.	-0.27	7.52	5.51E-46	7.46E-45	midnightblue
MAPKAPK3	7867	mitogen-activated protein kinase-activated protein kinase 3 (MAPKAPK3), mRNA.	0.09	5.54	3.66E-06	7.18E-06	midnightblue
MFSD10	10227	tetracycline transporter-like protein (TETRAN), mRNA.	-0.14	8.99	1.52E-29	9.66E-29	midnightblue

MRPL21	219927	mitochondrial ribosomal protein L21 (MRPL21), nuclear gene encoding mitochondrial protein, transcript variant 1, mRNA.	-0.06	7.15	1.39E-04	2.38E-04	midnightblue
MTMR14	64419	myotubularin related protein 14 (MTMR14), transcript variant 1, mRNA.	-0.31	9.65	1.29E-53	2.33E-52	midnightblue
MUTYH	4595	mutY homolog (E. coli) (MUTYH), mRNA.	0.08	7.75	5.62E-10	1.41E-09	midnightblue
MXD4	10608	MAX dimerization protein 4 (MXD4), mRNA.	-0.03	10.60	2.09E-03	3.19E-03	midnightblue
MYD88	4615	myeloid differentiation primary response gene (88) (MYD88), mRNA.	0.09	6.93	3.06E-05	5.59E-05	midnightblue
MYPOP	339344	hypothetical protein LOC339344 (LOC339344), mRNA.	-0.10	8.41	8.89E-13	2.61E-12	midnightblue
NCKIPSD	51517	NCK interacting protein with SH3 domain (NCKIPSD), transcript variant 2, mRNA.	0.06	11.65	1.52E-05	2.85E-05	midnightblue
NFKBIA	4792	nuclear factor of kappa light polypeptide gene enhancer in B-cells inhibitor, alpha (NFKBIA), mRNA.	-0.03	6.05	3.67E-02	4.86E-02	midnightblue
NLRX1	79671	NLR family member X1 (NLRX1), transcript variant 2, mRNA.	0.06	8.19	6.51E-08	1.44E-07	midnightblue
NMI	9111	N-myc (and STAT) interactor (NMI), mRNA.	-0.06	5.68	4.21E-05	7.58E-05	midnightblue

PACS1	55690	phosphofurin acidic cluster sorting protein 1 (PACS1), mRNA.	0.19	9.39	7.08E-25	3.82E-24	midnightblue
PARP10	84875	poly (ADP-ribose) polymerase family, member 10 (PARP10), mRNA.	-0.32	11.67	3.64E-43	4.27E-42	midnightblue
PDCD2	5134	programmed cell death 2 (PDCD2), transcript variant 2, mRNA.	0.03	8.09	3.07E-03	4.61E-03	midnightblue
PDSS1	23590	prenyl (decaprenyl) diphosphate synthase, subunit 1 (PDSS1), mRNA.	0.10	7.66	2.50E-13	7.58E-13	midnightblue
PIP4K2A	5305	phosphatidylinositol-4-phosphate 5-kinase, type II, alpha (PIP5K2A), mRNA.	-0.06	9.34	9.18E-04	1.46E-03	midnightblue
PLGRKT	55848	chromosome 9 open reading frame 46 (C9orf46), mRNA.	-0.10	7.14	1.65E-11	4.51E-11	midnightblue
POLR1C	9533	polymerase (RNA) I polypeptide C, 30kDa (POLR1C), transcript variant 2, mRNA.	0.03	10.66	1.39E-02	1.95E-02	midnightblue
POLR3H	171568	polymerase (RNA) III (DNA directed) polypeptide H (22.9kD) (POLR3H), transcript variant 4, mRNA.	-0.02	6.14	1.58E-01	1.91E-01	midnightblue
PQLC1	80148	PQ loop repeat containing 1 (PQLC1), mRNA.	0.01	8.57	4.15E-01	4.61E-01	midnightblue
PSAT1	29968	phosphoserine aminotransferase 1 (PSAT1), transcript variant 2, mRNA.	0.12	10.07	2.30E-17	8.68E-17	midnightblue
PSMA4	5685	proteasome (prosome, macropain) subunit, alpha type, 4 (PSMA4), mRNA.	-0.18	9.80	1.72E-23	8.65E-23	midnightblue

RASSF7	8045	Ras association (RalGDS/AF-6) domain family 7 (RASSF7), mRNA.	0.06	8.74	4.80E-06	9.35E-06	midnightblue
RRNAD1	51093	chromosome 1 open reading frame 66 (C1orf66), mRNA.	-0.02	8.66	2.48E-01	2.88E-01	midnightblue
RYK	6259	RYK receptor-like tyrosine kinase (RYK), transcript variant 1, mRNA.	-0.01	8.98	4.04E-01	4.49E-01	midnightblue
SARS2	54938	seryl-tRNA synthetase 2, mitochondrial (SARS2), mRNA.	0.42	7.20	3.05E-63	1.02E-61	midnightblue
SIX5	147912	sine oculis homeobox homolog 5 (Drosophila) (SIX5), mRNA.	-0.01	11.80	2.83E-01	3.26E-01	midnightblue
SLC25A28	81894	solute carrier family 25, member 28 (SLC25A28), mRNA.	-0.02	8.02	2.24E-01	2.63E-01	midnightblue
SLC31A1	1317	solute carrier family 31 (copper transporters), member 1 (SLC31A1), mRNA.	-0.01	6.17	5.94E-01	6.36E-01	midnightblue
SLC37A4	2542	solute carrier family 37 (glycerol-6-phosphate transporter), member 4 (SLC37A4), mRNA.	0.18	8.44	3.68E-26	2.09E-25	midnightblue
SLC52A2	79581	G protein-coupled receptor 172A (GPR172A), mRNA.	-0.28	8.29	6.03E-42	6.67E-41	midnightblue
SMIM14	201895	chromosome 4 open reading frame 34 (C4orf34), mRNA.	0.11	9.19	4.18E-10	1.06E-09	midnightblue

SPSB3	90864	splA/ryanodine receptor domain and SOCS box containing 3 (SPSB3), mRNA.	0.20	6.68	8.46E-23	4.14E-22	midnightblue
SRP19	6728	signal recognition particle 19kDa (SRP19), mRNA.	0.10	7.74	7.69E-07	1.58E-06	midnightblue
STX5	6811	syntaxin 5A (STX5A), mRNA.	0.04	7.94	5.10E-04	8.33E-04	midnightblue
TGOLN2	10618	trans-golgi network protein 2 (TGOLN2), mRNA.	-0.69	10.29	7.13E-92	1.65E-89	midnightblue
TIGD5	84948	tigger transposable element derived 5 (TIGD5), mRNA.	0.12	6.68	2.29E-17	8.62E-17	midnightblue
TIMELESS	8914	timeless homolog (Drosophila) (TIMELESS), mRNA.	0.05	8.58	9.24E-06	1.76E-05	midnightblue
TNK2	10188	tyrosine kinase, non-receptor, 2 (TNK2), transcript variant 1, mRNA.	-0.24	6.33	2.00E-35	1.64E-34	midnightblue
TP53I13	90313	tumor protein p53 inducible protein 13 (TP53I13), mRNA.	-0.31	8.40	4.55E-40	4.58E-39	midnightblue
TPRG1L	127262	family with sequence similarity 79, member A (FAM79A), mRNA.	0.07	10.72	4.99E-07	1.04E-06	midnightblue
TRIB3	57761	tribbles homolog 3 (Drosophila) (TRIB3), mRNA.	-0.03	5.44	1.80E-01	2.16E-01	midnightblue
TRIM21	6737	tripartite motif-containing 21 (TRIM21), mRNA.	-0.15	8.43	1.25E-26	7.20E-26	midnightblue
TSC22D4	81628	TSC22 domain family, member 4 (TSC22D4), mRNA.	0.11	6.33	2.90E-12	8.27E-12	midnightblue
UBE2T	29089	ubiquitin-conjugating enzyme E2T (putative) (UBE2T), mRNA.	-0.08	9.60	4.97E-11	1.32E-10	midnightblue

UCHL3	7347	ubiquitin carboxyl-terminal esterase L3 (ubiquitin thiolesterase) (UCHL3), mRNA.	-0.25	9.29	6.80E-51	1.10E-49	midnightblue
WDR61	80349	WD repeat domain 61 (WDR61), mRNA.	-0.46	6.01	3.56E-40	3.62E-39	midnightblue
WRAP53	55135	WD repeat domain 79 (WDR79), mRNA.	-0.23	7.36	6.35E-17	2.34E-16	midnightblue
XYLT2	64132	xylosyltransferase II (XYLT2), mRNA.	-0.01	6.91	4.42E-01	4.88E-01	midnightblue
ZFP90	146198	zinc finger protein 90 homolog (mouse) (ZFP90), mRNA.	-0.16	8.89	9.04E-22	4.19E-21	midnightblue
AASDHPPT	60496	aminoadipate-semialdehyde dehydrogenase-phosphopantetheinyl transferase (AASDHPPT), mRNA.	-0.15	9.94	2.28E-15	7.66E-15	pink
ACOT7	11332	acyl-CoA thioesterase 7 (ACOT7), transcript variant hBACHb, mRNA.	-0.07	7.59	5.12E-05	9.14E-05	pink
ADPRHL2	54936	ADP-ribosylhydrolase like 2 (ADPRHL2), mRNA.	0.00	6.67	8.51E-01	8.73E-01	pink
AHCY	191	S-adenosylhomocysteine hydrolase (AHCY), mRNA.	-0.50	8.21	1.02E-56	2.23E-55	pink
ALG6	29929	asparagine-linked glycosylation 6 homolog (S. cerevisiae, alpha-1,3-glucosyltransferase) (ALG6), mRNA.	-0.02	9.11	2.78E-02	3.73E-02	pink

ALG8	79053	asparagine-linked glycosylation 8 homolog (S. cerevisiae, alpha-1,3-glucosyltransferase) (ALG8), transcript variant 1, mRNA.	-0.16	7.85	2.28E-21	1.04E-20	pink
ALKBH6	84964	alkB, alkylation repair homolog 6 (E. coli) (ALKBH6), transcript variant 1, mRNA.	0.01	9.10	7.34E-01	7.65E-01	pink
ALKBH7	84266	alkB, alkylation repair homolog 7 (E. coli) (ALKBH7), mRNA.	0.05	9.37	9.88E-06	1.88E-05	pink
ALOX5AP	241	arachidonate 5-lipoxygenase-activating protein (ALOX5AP), mRNA.	-0.01	6.51	3.78E-01	4.23E-01	pink
APEX1	328	APEX nuclease (multifunctional DNA repair enzyme) 1 (APEX1), transcript variant 2, mRNA.	-0.30	6.50	2.38E-46	3.30E-45	pink
API5	8539	apoptosis inhibitor 5 (API5), mRNA.	0.00	7.95	9.56E-01	9.62E-01	pink
ARHGAP30	257106	Rho GTPase activating protein 30 (ARHGAP30), transcript variant 1, mRNA.	-0.05	5.68	6.79E-04	1.09E-03	pink
ARL2	402	ADP-ribosylation factor-like 2 (ARL2), mRNA.	0.02	9.98	1.82E-01	2.17E-01	pink
ASCC3	10973	activating signal cointegrator 1 complex subunit 3 (ASCC3), transcript variant 1, mRNA.	0.05	7.84	1.94E-04	3.29E-04	pink
ASMTL	8623	acetylserotonin O-methyltransferase-like (ASMTL), mRNA.	-0.12	9.45	3.79E-19	1.56E-18	pink

ATP5G2	517	ATP synthase, H+ transporting, mitochondrial F0 complex, subunit C2 (subunit 9) (ATP5G2), nuclear gene encoding mitochondrial protein, transcript variant 2, mRNA.	0.12	8.37	1.77E-13	5.41E-13	pink
ATPIF1	93974	ATPase inhibitory factor 1 (ATPIF1), nuclear gene encoding mitochondrial protein, transcript variant 3, mRNA.	0.19	6.22	3.94E-21	1.77E-20	pink
BATF3	55509	Jun dimerization protein p21SNFT (SNFT), mRNA.	-0.07	9.48	7.72E-03	1.11E-02	pink
BCL2L10	10017	BCL2-like 10 (apoptosis facilitator) (BCL2L10), mRNA.	0.05	7.22	4.93E-04	8.08E-04	pink
BCLAF1	9774	BCL2-associated transcription factor 1 (BCLAF1), transcript variant 2, mRNA.	-0.04	5.99	4.90E-02	6.40E-02	pink
BCOR	54880	BCL6 co-repressor (BCOR), transcript variant 1, mRNA.	-0.02	9.51	3.62E-02	4.80E-02	pink
BLM	641	Bloom syndrome (BLM), mRNA.	-0.08	9.45	2.13E-11	5.81E-11	pink
BMPR2	659	bone morphogenetic protein receptor, type II (serine/threonine kinase) (BMPR2), mRNA.	-0.02	8.00	2.05E-01	2.43E-01	pink
BRK1	55845	chromosome 3 open reading frame 10 (C3orf10), mRNA.	-0.03	7.07	9.43E-03	1.35E-02	pink

BRMS1L	84312	breast cancer metastasis-suppressor 1-like (BRMS1L), mRNA.	0.02	11.63	5.36E-02	6.96E-02	pink
C12orf10	60314	chromosome 12 open reading frame 10 (C12orf10), mRNA.	-0.05	9.60	6.93E-02	8.87E-02	pink
C1orf50	79078	chromosome 1 open reading frame 50 (C1orf50), mRNA.	0.01	10.02	5.11E-01	5.56E-01	pink
C20orf27	54976	chromosome 20 open reading frame 27 (C20orf27), mRNA.	-0.16	8.54	1.20E-15	4.13E-15	pink
C6orf1	221491	chromosome 6 open reading frame 1 (C6orf1), transcript variant 1, mRNA.	0.05	9.92	1.29E-04	2.22E-04	pink
C6orf62	81688	chromosome 6 open reading frame 62 (C6orf62), mRNA.	0.74	6.14	1.51E-75	9.15E-74	pink
C7orf50	84310	hypothetical protein MGC11257 (MGC11257), mRNA.	0.11	8.28	5.73E-09	1.36E-08	pink
CAPZA2	830	capping protein (actin filament) muscle Z-line, alpha 2 (CAPZA2), mRNA.	0.07	7.05	1.80E-02	2.48E-02	pink
CASC4	113201	cancer susceptibility candidate 4 (CASC4), transcript variant 2, mRNA.	-0.03	6.82	5.36E-02	6.96E-02	pink
CBX7	23492	chromobox homolog 7 (CBX7), mRNA.	-0.44	6.33	4.21E-58	1.02E-56	pink
CCDC106	29903	coiled-coil domain containing 106 (CCDC106), mRNA.	-0.17	10.78	3.93E-23	1.95E-22	pink

CDCA4	55038	cell division cycle associated 4 (CDCA4), transcript variant 13, mRNA.	-0.25	6.50	4.05E-22	1.91E-21	pink
CDCA7L	55536	cell division cycle associated 7-like (CDCA7L), mRNA.	-0.06	6.79	8.44E-05	1.48E-04	pink
CDK19	23097	cell division cycle 2-like 6 (CDK8-like) (CDC2L6), mRNA.	0.29	8.77	1.65E-27	9.89E-27	pink
CENPN	55839	centromere protein N (CENPN), mRNA.	0.11	8.84	1.71E-08	3.93E-08	pink
CEP350	9857	centrosomal protein 350kDa (CEP350), mRNA.	0.06	8.85	8.89E-05	1.56E-04	pink
CLIC1	1192	chloride intracellular channel 1 (CLIC1), mRNA.	-0.09	8.43	1.79E-09	4.36E-09	pink
COA7	65260	chromosome 1 open reading frame 163 (C1orf163), mRNA.	-0.07	8.93	2.41E-03	3.65E-03	pink
COMMD7	149951	COMM domain containing 7 (COMMD7), mRNA.	0.00	8.40	7.89E-01	8.16E-01	pink
COPB1	1315	coatomer protein complex, subunit beta 1 (COPB1), mRNA.	0.14	7.43	5.67E-15	1.87E-14	pink
CPNE3	8895	copine III (CPNE3), mRNA.	0.03	6.76	4.04E-02	5.33E-02	pink
CPSF4	10898	cleavage and polyadenylation specific factor 4, 30kDa (CPSF4), transcript variant 1, mRNA.	0.26	8.18	4.82E-50	7.58E-49	pink
CREBRF	153222	adult retina protein (LOC153222), mRNA.	-0.03	6.28	6.19E-02	7.98E-02	pink
CSE1L	1434	CSE1 chromosome segregation 1-like (yeast) (CSE1L), mRNA.	-0.30	6.76	2.36E-40	2.42E-39	pink

DCAF7	10238	WD repeat domain 68 (WDR68), mRNA.	0.18	9.80	2.20E-23	1.10E-22	pink
DEDD2	162989	death effector domain containing 2 (DEDD2), mRNA.	-0.03	10.34	1.59E-02	2.20E-02	pink
DEF6	50619	differentially expressed in FDCP 6 homolog (mouse) (DEF6), mRNA.	0.03	7.81	1.51E-02	2.10E-02	pink
DESI2	51029	chromosome 1 open reading frame 121 (C1orf121), mRNA.	0.34	10.69	1.80E-48	2.72E-47	pink
DGKQ	1609	diacylglycerol kinase, theta 110kDa (DGKQ), mRNA.	-0.02	7.50	2.73E-01	3.16E-01	pink
DGUOK	1716	deoxyguanosine kinase (DGUOK), nuclear gene encoding mitochondrial protein, transcript variant 2, mRNA.	-0.08	5.59	1.10E-04	1.91E-04	pink
DHX15	1665	DEAH (Asp-Glu-Ala-His) box polypeptide 15 (DHX15), mRNA.	-0.07	7.59	1.52E-03	2.35E-03	pink
DMTF1	9988	cyclin D binding myb-like transcription factor 1 (DMTF1), mRNA.	-0.06	7.79	5.69E-04	9.25E-04	pink
DPCD	25911	deleted in a mouse model of primary ciliary dyskinesia (RP11-529I10.4), mRNA.	0.05	7.06	1.34E-04	2.31E-04	pink
DROSHA	29102	ribonuclease III, nuclear (RNASEN), mRNA.	0.01	7.34	2.23E-01	2.62E-01	pink
DUSP10	11221	dual specificity phosphatase 10 (DUSP10), transcript variant 1, mRNA.	-0.13	5.82	7.66E-14	2.39E-13	pink

ECI1	1632	dodecenoyl-Coenzyme A delta isomerase (3,2 trans-enoyl-Coenzyme A isomerase) (DCI), nuclear gene encoding mitochondrial protein, mRNA.	-0.01	12.25	5.57E-01	6.01E-01	pink
EIF4B	1975	eukaryotic translation initiation factor 4B (EIF4B), mRNA.	0.39	7.50	2.40E-64	8.40E-63	pink
EIF4EBP2	1979	eukaryotic translation initiation factor 4E binding protein 2 (EIF4EBP2), mRNA.	-0.03	7.77	7.06E-03	1.02E-02	pink
EMC9	51016	chromosome 14 open reading frame 122 (C14orf122), mRNA.	-0.04	8.71	1.79E-03	2.76E-03	pink
EMD	2010	emerin (Emery-Dreifuss muscular dystrophy) (EMD), mRNA.	-0.01	7.68	4.93E-01	5.38E-01	pink
EMP3	2014	epithelial membrane protein 3 (EMP3), mRNA.	0.03	8.28	1.05E-02	1.48E-02	pink
EXOSC7	23016	exosome component 7 (EXOSC7), mRNA.	-0.08	6.49	2.66E-07	5.65E-07	pink
FAM212A	389119	chromosome 3 open reading frame 54 (C3orf54), mRNA.	0.16	6.88	7.99E-19	3.22E-18	pink
FAM50A	9130	family with sequence similarity 50, member A (FAM50A), mRNA.	0.14	7.47	1.63E-07	3.51E-07	pink
FAM58A	92002	family with sequence similarity 58, member A (FAM58A), mRNA.	0.04	8.97	2.50E-02	3.39E-02	pink
FBL	2091	fibrillarin (FBL), mRNA.	0.09	9.24	8.09E-09	1.89E-08	pink

FECH	2235	ferrochelatase (protoporphyria) (FECH), nuclear gene encoding mitochondrial protein, transcript variant 2, mRNA.	0.03	8.17	4.47E-02	5.87E-02	pink
FEZ2	9637	fasciculation and elongation protein zeta 2 (zygin II) (FEZ2), transcript variant 2, mRNA.	-0.06	6.36	4.29E-05	7.72E-05	pink
FIBP	9158	fibroblast growth factor (acidic) intracellular binding protein (FIBP), transcript variant 2, mRNA.	0.58	8.01	7.06E-57	1.57E-55	pink
FOPNL	123811	chromosome 16 open reading frame 63 (C16orf63), mRNA.	0.01	6.56	2.29E-01	2.69E-01	pink
FTSJ1	24140	FtsJ homolog 1 (E. coli) (FTSJ1), transcript variant 2, mRNA.	-0.05	7.89	5.01E-07	1.04E-06	pink
GALNT11	63917	UDP-N-acetyl-alpha-D-galactosamine:polypeptide N-acetylgalactosaminyltransferase 11 (GalNAc-T11) (GALNT11), mRNA.	0.74	7.54	5.70E-90	1.16E-87	pink
GFOD2	81577	glucose-fructose oxidoreductase domain containing 2 (GFOD2), mRNA.	0.04	11.05	1.28E-04	2.21E-04	pink
GLMP	112770	chromosome 1 open reading frame 85 (C1orf85), mRNA.	-0.15	9.57	2.84E-15	9.52E-15	pink
GM2A	2760	GM2 ganglioside activator (GM2A), mRNA.	0.39	6.04	3.63E-31	2.47E-30	pink

GMDS	2762	GDP-mannose 4,6-dehydratase (GMDS), mRNA.	-0.11	6.23	4.28E-11	1.14E-10	pink
GNA13	10672	guanine nucleotide binding protein (G protein), alpha 13 (GNA13), mRNA.	-0.05	8.16	3.05E-03	4.57E-03	pink
GOLPH3	64083	golgi phosphoprotein 3 (coat-protein) (GOLPH3), mRNA.	0.13	8.58	1.43E-11	3.92E-11	pink
GSDMD	79792	gasdermin domain containing 1 (GSDMDC1), mRNA.	-0.23	8.50	4.57E-41	4.80E-40	pink
GTF3C2	2976	general transcription factor IIIC, polypeptide 2, beta 110kDa (GTF3C2), transcript variant 1, mRNA.	0.05	6.75	2.39E-04	4.03E-04	pink
HAUS7	55559	UCHL5 interacting protein (UCHL5IP), transcript variant 2, mRNA.	-0.01	12.37	4.86E-01	5.31E-01	pink
HECTD1	25831	HECT domain containing 1 (HECTD1), mRNA.	-0.20	9.21	5.29E-35	4.22E-34	pink
HNRNPR	10236	heterogeneous nuclear ribonucleoprotein R (HNRPR), mRNA.	-0.02	8.89	1.58E-01	1.91E-01	pink
HPS1	3257	Hermansky-Pudlak syndrome 1 (HPS1), transcript variant 3, mRNA.	-0.11	9.87	1.43E-14	4.64E-14	pink
HSD17B10	3028	hydroxysteroid (17-beta) dehydrogenase 10 (HSD17B10), nuclear gene encoding mitochondrial protein, transcript variant 2, mRNA.	-0.05	6.52	2.64E-03	3.98E-03	pink

IARS2	55699	isoleucyl-tRNA synthetase 2, mitochondrial (IARS2), mRNA.	-0.24	6.21	8.22E-34	6.22E-33	pink
IMMT	10989	inner membrane protein, mitochondrial (mitofilin) (IMMT), mRNA.	0.03	6.73	1.20E-01	1.49E-01	pink
INPP5E	56623	inositol polyphosphate-5-phosphatase, 72 kDa (INPP5E), mRNA.	0.00	5.81	8.43E-01	8.66E-01	pink
IVNS1ABP	10625	influenza virus NS1A binding protein (IVNS1ABP), transcript variant 2, mRNA.	-0.09	7.60	4.29E-09	1.02E-08	pink
KIDINS220	57498	kinase D-interacting substance of 220 kDa (KIDINS220), mRNA.	0.21	8.71	2.09E-25	1.15E-24	pink
KLF12	11278	Kruppel-like factor 12 (KLF12), mRNA.	-0.13	7.06	5.70E-10	1.43E-09	pink
KLHL36	79786	chromosome 16 open reading frame 44 (C16orf44), mRNA.	-0.15	5.76	2.44E-13	7.40E-13	pink
LAMTOR1	55004	chromosome 11 open reading frame 59 (C11orf59), mRNA.	0.00	11.78	9.97E-01	9.98E-01	pink
LCMT2	9836	leucine carboxyl methyltransferase 2 (LCMT2), mRNA.	0.24	7.51	1.51E-37	1.37E-36	pink
LEO1	123169	Leo1, Paf1/RNA polymerase II complex component, homolog (S. cerevisiae) (LEO1), mRNA.	0.01	8.36	4.95E-01	5.40E-01	pink
LMNA	4000	lamin A/C (LMNA), transcript variant 2, mRNA.	-0.18	6.13	9.09E-16	3.14E-15	pink

LRRC23	10233	leucine rich repeat containing 23 (LRRC23), transcript variant 2, mRNA.	0.07	7.73	4.35E-06	8.50E-06	pink
LSM14A	26065	LSM14A, SCD6 homolog A (S. cerevisiae) (LSM14A), mRNA.	0.54	8.30	1.40E-69	6.28E-68	pink
LSM2	57819	LSM2 homolog, U6 small nuclear RNA associated (S. cerevisiae) (LSM2), mRNA.	-0.02	7.80	2.43E-01	2.84E-01	pink
MCCC1	56922	methylcrotonoyl-Coenzyme A carboxylase 1 (alpha) (MCCC1), mRNA.	0.05	8.35	3.99E-03	5.92E-03	pink
MED10	84246	mediator of RNA polymerase II transcription, subunit 10 homolog (NUT2, S. cerevisiae) (MED10), mRNA.	0.13	8.91	5.94E-12	1.67E-11	pink
MED19	219541	mediator of RNA polymerase II transcription, subunit 19 homolog (S. cerevisiae) (MED19), mRNA.	-0.06	7.87	1.50E-03	2.33E-03	pink
METAP2	10988	methionyl aminopeptidase 2 (METAP2), mRNA.	-0.77	6.46	9.38E-35	7.36E-34	pink
MFF	56947	chromosome 2 open reading frame 33 (C2orf33), mRNA.	-0.23	7.40	1.44E-24	7.68E-24	pink
MFSD1	64747	major facilitator superfamily domain containing 1 (MFSD1), mRNA.	-0.03	8.42	2.18E-01	2.57E-01	pink
MICA	4276	MHC class I polypeptide-related sequence A (MICA), mRNA.	-0.49	7.00	2.20E-63	7.39E-62	pink

MICB	4277	MHC class I polypeptide-related sequence B (MICB), mRNA.	-0.10	7.31	6.67E-12	1.86E-11	pink
MIF4GD	57409	MIF4G domain containing (MIF4GD), mRNA.	-0.08	8.33	1.22E-04	2.11E-04	pink
MMGT1	93380	transmembrane protein 32 (TMEM32), mRNA.	0.03	8.11	5.57E-02	7.22E-02	pink
MOK	5891	renal tumor antigen (RAGE), mRNA.	-0.07	5.88	1.63E-04	2.78E-04	pink
MPDU1	9526	mannose-P-dolichol utilization defect 1 (MPDU1), mRNA.	-0.06	10.74	1.77E-05	3.31E-05	pink
MPND	84954	MPN domain containing (MPND), mRNA.	0.19	9.27	3.72E-28	2.28E-27	pink
MRGBP	55257	chromosome 20 open reading frame 20 (C20orf20), mRNA.	-0.02	5.65	2.22E-01	2.62E-01	pink
MRPL11	65003	mitochondrial ribosomal protein L11 (MRPL11), nuclear gene encoding mitochondrial protein, transcript variant 3, mRNA.	-0.17	7.54	7.57E-19	3.06E-18	pink
MRPL17	63875	mitochondrial ribosomal protein L17 (MRPL17), nuclear gene encoding mitochondrial protein, mRNA.	0.01	7.80	3.86E-01	4.31E-01	pink
MRPL20	55052	mitochondrial ribosomal protein L20 (MRPL20), nuclear gene encoding mitochondrial protein, mRNA.	0.12	7.75	4.33E-17	1.61E-16	pink

MRPS26	64949	mitochondrial ribosomal protein S26 (MRPS26), nuclear gene encoding mitochondrial protein, mRNA.	0.29	7.07	3.03E-28	1.86E-27	pink
MRPS7	51081	mitochondrial ribosomal protein S7 (MRPS7), nuclear gene encoding mitochondrial protein, mRNA.	0.00	7.44	9.07E-01	9.22E-01	pink
MTHFD1L	25902	methylenetetrahydrofolate dehydrogenase (NADP+ dependent) 1-like (MTHFD1L), mRNA.	0.03	8.09	3.18E-02	4.24E-02	pink
MVB12A	93343	family with sequence similarity 125, member A (FAM125A), mRNA.	-0.26	11.17	4.61E-42	5.16E-41	pink
MYCBP2	23077	MYC binding protein 2 (MYCBP2), mRNA.	0.00	6.64	8.67E-01	8.87E-01	pink
NA	8214	DiGeorge syndrome critical region gene 6 (DGCR6), mRNA.	-0.33	6.44	1.79E-43	2.12E-42	pink
NA	84163	GTF2I repeat domain containing 2 (GTF2IRD2), mRNA.	-0.20	6.44	2.10E-24	1.11E-23	pink
NA	85359	DiGeorge syndrome critical region gene 6-like (DGCR6L), mRNA.	-0.02	7.10	2.41E-01	2.81E-01	pink
NA	136319	myotrophin (MTPN), mRNA.	-0.02	8.35	2.41E-01	2.82E-01	pink
NAA20	51126	N-acetyltransferase 5 (NAT5), transcript variant 2, mRNA.	-0.15	8.51	1.45E-19	6.07E-19	pink

NAP1L1	4673	nucleosome assembly protein 1-like 1 (NAP1L1), transcript variant 2, mRNA.	0.16	9.08	5.68E-15	1.87E-14	pink
NDUFA8	4702	NADH dehydrogenase (ubiquinone) 1 alpha subcomplex, 8, 19kDa (NDUFA8), nuclear gene encoding mitochondrial protein, mRNA.	0.04	7.31	3.71E-03	5.53E-03	pink
NDUFB8	4714	NADH dehydrogenase (ubiquinone) 1 beta subcomplex, 8, 19kDa (NDUFB8), mRNA.	0.49	6.96	9.13E-48	1.36E-46	pink
NDUFS6	4726	NADH dehydrogenase (ubiquinone) Fe-S protein 6, 13kDa (NADH-coenzyme Q reductase) (NDUFS6), mRNA.	0.13	6.26	2.94E-12	8.38E-12	pink
NDUFS7	374291	NADH dehydrogenase (ubiquinone) Fe-S protein 7, 20kDa (NADH-coenzyme Q reductase) (NDUFS7), mRNA.	-0.36	5.99	4.31E-35	3.46E-34	pink
NDUFS8	4728	NADH dehydrogenase (ubiquinone) Fe-S protein 8, 23kDa (NADH-coenzyme Q reductase) (NDUFS8), mRNA.	-0.02	7.76	3.00E-01	3.44E-01	pink
NMRAL1	57407	NmrA-like family domain containing 1 (NMRAL1), mRNA.	0.11	7.87	2.39E-11	6.48E-11	pink

NNT	23530	nicotinamide nucleotide transhydrogenase (NNT), nuclear gene encoding mitochondrial protein, transcript variant 2, mRNA.	-0.01	10.87	6.45E-01	6.83E-01	pink
NOL8	55035	nucleolar protein 8 (NOL8), mRNA.	-0.14	6.74	3.57E-19	1.47E-18	pink
NOSIP	51070	nitric oxide synthase interacting protein (NOSIP), mRNA.	0.05	7.71	3.58E-03	5.35E-03	pink
NSMCE2	286053	non-SMC element 2, MMS21 homolog (S. cerevisiae) (NSMCE2), mRNA.	0.13	6.15	2.56E-17	9.59E-17	pink
NT5C	30833	5', 3'-nucleotidase, cytosolic (NT5C), mRNA.	0.01	9.25	6.37E-01	6.76E-01	pink
NUDT14	256281	nudix (nucleoside diphosphate linked moiety X)-type motif 14 (NUDT14), mRNA.	0.29	8.20	2.16E-35	1.76E-34	pink
NUP160	23279	nucleoporin 160kDa (NUP160), mRNA.	0.80	6.10	1.47E-78	1.22E-76	pink
PAN3	255967	PAN3 polyA specific ribonuclease subunit homolog (S. cerevisiae) (PAN3), mRNA.	0.25	8.17	5.98E-24	3.08E-23	pink
PCNP	57092	PEST proteolytic signal containing nuclear protein (PCNP), mRNA.	0.01	7.34	6.96E-01	7.31E-01	pink

PCYT2	653414	PREDICTED: similar to Ethanolamine-phosphate cytidylyltransferase (Phosphorylethanolamine transferase) (CTP:phosphoethanolamine cytidylyltransferase) (LOC653414), mRNA.	-0.74	9.32	1.15E-80	1.04E-78	pink
PDLIM7	9260	PDZ and LIM domain 7 (enigma) (PDLIM7), transcript variant 4, mRNA.	-0.13	7.45	5.97E-19	2.43E-18	pink
PECR	55825	peroxisomal trans-2-enoyl-CoA reductase (PECR), mRNA.	0.00	5.96	9.20E-01	9.32E-01	pink
PEX10	5192	peroxisome biogenesis factor 10 (PEX10), transcript variant 1, mRNA.	-0.03	9.71	4.85E-03	7.14E-03	pink
PEX16	9409	peroxisomal biogenesis factor 16 (PEX16), transcript variant 1, mRNA.	-0.14	6.71	4.96E-14	1.56E-13	pink
PIGU	128869	phosphatidylinositol glycan anchor biosynthesis, class U (PIGU), mRNA.	-0.09	10.11	2.76E-05	5.07E-05	pink
PIN1	5300	protein (peptidylprolyl cis/trans isomerase) NIMA-interacting 1 (PIN1), mRNA.	0.03	5.47	5.66E-02	7.33E-02	pink
PLD3	23646	phospholipase D family, member 3 (PLD3), transcript variant 1, mRNA.	0.26	8.47	1.91E-38	1.81E-37	pink
PPM1M	132160	protein phosphatase 1M (PP2C domain containing) (PPM1M), mRNA.	-0.33	8.46	9.03E-57	2.00E-55	pink

PPP1R35	221908	chromosome 7 open reading frame 47 (C7orf47), mRNA.	0.05	8.97	3.05E-05	5.58E-05	pink
PROSER1	80209	chromosome 13 open reading frame 23 (C13orf23), transcript variant 2, mRNA.	0.01	6.24	3.17E-01	3.61E-01	pink
PRPSAP2	5636	phosphoribosyl pyrophosphate synthetase-associated protein 2 (PRPSAP2), mRNA.	0.00	10.44	8.76E-01	8.95E-01	pink
PSMB10	5699	proteasome (prosome, macropain) subunit, beta type, 10 (PSMB10), mRNA.	-0.14	7.38	9.81E-23	4.78E-22	pink
PSMB7	5695	proteasome (prosome, macropain) subunit, beta type, 7 (PSMB7), mRNA.	0.00	8.75	9.24E-01	9.36E-01	pink
RAD23B	5887	RAD23 homolog B (S. cerevisiae) (RAD23B), mRNA.	-0.01	6.02	6.76E-01	7.12E-01	pink
RANBP9	10048	RAN binding protein 9 (RANBP9), mRNA.	-0.01	9.44	4.37E-01	4.84E-01	pink
RAP2A	5911	RAP2A, member of RAS oncogene family (RAP2A), mRNA.	-0.19	8.26	5.04E-27	2.96E-26	pink
RBM26	64062	RNA binding motif protein 26 (RBM26), mRNA.	0.07	8.25	5.45E-05	9.71E-05	pink
RDX	5962	radixin (RDX), mRNA.	-0.09	9.22	1.06E-11	2.94E-11	pink
RGS10	6001	regulator of G-protein signalling 10 (RGS10), transcript variant 1, mRNA.	-0.24	5.84	1.42E-36	1.23E-35	pink

RHOC	389	ras homolog gene family, member C (RHOC), transcript variant 3, mRNA.	-0.19	10.19	3.31E-31	2.25E-30	pink
RING1	6015	ring finger protein 1 (RING1), mRNA.	-0.31	8.44	1.24E-39	1.22E-38	pink
RNF167	26001	ring finger protein 167 (RNF167), mRNA.	-0.02	5.50	3.11E-01	3.55E-01	pink
RPUSD3	285367	RNA pseudouridylate synthase domain containing 3 (RPUSD3), mRNA.	-0.02	5.56	3.26E-01	3.71E-01	pink
RRM1	6240	ribonucleotide reductase M1 polypeptide (RRM1), mRNA.	-0.03	9.44	2.81E-02	3.77E-02	pink
RRM2B	50484	ribonucleotide reductase M2 B (TP53 inducible) (RRM2B), mRNA.	0.05	7.12	3.45E-04	5.72E-04	pink
RRP1B	23076	KIAA0179 (KIAA0179), mRNA.	0.00	7.97	8.92E-01	9.09E-01	pink
RRP36	88745	chromosome 6 open reading frame 153 (C6orf153), mRNA.	0.00	8.44	9.53E-01	9.60E-01	pink
RRP8	23378	KIAA0409 (KIAA0409), mRNA.	-0.04	8.55	1.02E-02	1.45E-02	pink
SDHAF2	54949	chromosome 11 open reading frame 79 (C11orf79), mRNA.	0.08	6.06	3.83E-08	8.56E-08	pink
SERTAD1	29950	SERTA domain containing 1 (SERTAD1), mRNA.	-0.02	7.40	2.95E-01	3.39E-01	pink
SET	6418	SET translocation (myeloid leukemia-associated) (SET), mRNA.	-0.06	10.45	8.60E-05	1.51E-04	pink
SF3A1	10291	splicing factor 3a, subunit 1, 120kDa (SF3A1), transcript variant 2, mRNA.	-0.13	5.72	1.18E-15	4.05E-15	pink

SGOL2	151246	shugoshin-like 2 (S. pombe) (SGOL2), mRNA.	0.06	7.97	2.71E-04	4.55E-04	pink
SH3KBP1	30011	SH3-domain kinase binding protein 1 (SH3KBP1), transcript variant 2, mRNA.	0.77	7.37	9.35E-83	9.69E-81	pink
SKAP1	8631	src kinase associated phosphoprotein 1 (SKAP1), transcript variant 1, mRNA.	-0.07	5.47	7.96E-04	1.27E-03	pink
SLC2A8	29988	solute carrier family 2, (facilitated glucose transporter) member 8 (SLC2A8), mRNA.	0.10	6.99	1.30E-07	2.82E-07	pink
SLC41A3	54946	solute carrier family 41, member 3 (SLC41A3), transcript variant 1, mRNA.	-0.06	8.93	4.55E-05	8.15E-05	pink
SMARCC1	6599	SWI/SNF related, matrix associated, actin dependent regulator of chromatin, subfamily c, member 1 (SMARCC1), mRNA.	-0.11	8.47	1.26E-16	4.57E-16	pink
SMC2	10592	structural maintenance of chromosomes 2 (SMC2), transcript variant 2, mRNA.	0.05	7.07	4.56E-04	7.49E-04	pink
SMC3	9126	structural maintenance of chromosomes 3 (SMC3), mRNA.	0.10	5.49	6.94E-09	1.63E-08	pink
SNF8	11267	SNF8, ESCRT-II complex subunit, homolog (S. cerevisiae) (SNF8), mRNA.	-0.04	6.46	1.22E-02	1.73E-02	pink

SPG11	80208	KIAA1840 (KIAA1840), mRNA.	-0.32	8.71	1.66E-41	1.78E-40	pink
SRPK1	6732	SFRS protein kinase 1 (SRPK1), mRNA.	0.01	6.52	4.18E-01	4.64E-01	pink
SRRM1	10250	serine/arginine repetitive matrix 1 (SRRM1), mRNA.	-0.16	6.21	1.66E-15	5.65E-15	pink
SRXN1	140809	sulfiredoxin 1 homolog (S. cerevisiae) (SRXN1), mRNA.	0.01	9.34	6.04E-01	6.46E-01	pink
SSBP1	6742	single-stranded DNA binding protein 1 (SSBP1), mRNA.	0.00	6.98	7.63E-01	7.92E-01	pink
SSU72	29101	SSU72 RNA polymerase II CTD phosphatase homolog (S. cerevisiae) (SSU72), mRNA.	-0.24	6.70	1.30E-41	1.40E-40	pink
STAU1	6780	staufen, RNA binding protein, homolog 1 (Drosophila) (STAU1), transcript variant T4, mRNA.	-0.32	10.25	3.04E-61	9.03E-60	pink
STRA13	201254	stimulated by retinoic acid 13 homolog (mouse) (STRA13), mRNA.	-0.04	6.58	2.20E-02	3.00E-02	pink
STRAP	11171	serine/threonine kinase receptor associated protein (STRAP), mRNA.	0.05	7.85	2.43E-06	4.83E-06	pink
STX11	8676	syntaxin 11 (STX11), mRNA.	-0.09	7.53	1.78E-08	4.08E-08	pink
SZRD1	26099	chromosome 1 open reading frame 144 (C1orf144), mRNA.	0.10	7.86	5.33E-12	1.50E-11	pink
TACO1	51204	coiled-coil domain containing 44 (CCDC44), mRNA.	0.26	6.15	1.50E-21	6.88E-21	pink
TARS	6897	threonyl-tRNA synthetase (TARS), mRNA.	0.32	7.38	1.82E-39	1.78E-38	pink

TBC1D22A	25771	TBC1 domain family, member 22A (TBC1D22A), mRNA.	-0.06	7.68	7.42E-05	1.31E-04	pink
TCEAL3	85012	transcription elongation factor A (SII)-like 3 (TCEAL3), transcript variant 2, mRNA.	0.06	8.62	5.33E-04	8.70E-04	pink
TDRD7	23424	tudor domain containing 7 (TDRD7), mRNA.	0.19	7.07	5.57E-19	2.28E-18	pink
THYN1	29087	thymocyte nuclear protein 1 (THYN1), transcript variant 1, mRNA.	-0.02	5.58	1.03E-01	1.28E-01	pink
TIAL1	7073	TIA1 cytotoxic granule-associated RNA binding protein-like 1 (TIAL1), transcript variant 2, mRNA.	-0.02	6.96	3.63E-01	4.09E-01	pink
TIMM10	26519	translocase of inner mitochondrial membrane 10 homolog (yeast) (TIMM10), mRNA.	0.03	7.09	1.03E-02	1.46E-02	pink
TINF2	26277	TERF1 (TRF1)-interacting nuclear factor 2 (TINF2), mRNA.	0.14	7.68	1.27E-15	4.35E-15	pink
TM9SF4	9777	transmembrane 9 superfamily protein member 4 (TM9SF4), mRNA.	0.18	8.77	8.13E-30	5.24E-29	pink
TMED1	11018	transmembrane emp24 protein transport domain containing 1 (TMED1), mRNA.	0.13	9.73	2.35E-17	8.81E-17	pink
TMEM194A	23306	KIAA0286 protein (KIAA0286), mRNA.	-0.07	7.42	2.26E-07	4.81E-07	pink
TMEM205	374882	MBC3205 (UNQ501), mRNA.	0.08	6.87	1.91E-06	3.83E-06	pink

TMEM209	84928	hypothetical protein FLJ14803 (FLJ14803), mRNA.	-0.06	7.59	4.58E-04	7.53E-04	pink
TMEM245	23731	chromosome 9 open reading frame 5 (C9orf5), mRNA.	-0.02	6.93	1.90E-01	2.27E-01	pink
TP53I3	9540	tumor protein p53 inducible protein 3 (TP53I3), transcript variant 2, mRNA.	-0.17	7.89	1.24E-24	6.63E-24	pink
TPGS2	25941	chromosome 18 open reading frame 10 (C18orf10), mRNA.	0.26	7.02	8.38E-33	6.14E-32	pink
TRAPPC3	27095	trafficking protein particle complex 3 (TRAPPC3), mRNA.	0.05	8.95	8.59E-04	1.37E-03	pink
TSPAN17	26262	tetraspanin 17 (TSPAN17), transcript variant 1, mRNA.	0.03	7.35	7.00E-02	8.96E-02	pink
TSSC4	10078	tumor suppressing subtransferable candidate 4 (TSSC4), mRNA.	-0.01	5.49	5.35E-01	5.80E-01	pink
TTC14	151613	tetratricopeptide repeat domain 14 (TTC14), transcript variant 2, mRNA.	-0.13	7.05	9.13E-16	3.15E-15	pink
TTC3	7267	tetratricopeptide repeat domain 3 (TTC3), transcript variant 2, mRNA.	-0.06	8.59	8.71E-07	1.78E-06	pink
TYMS	7298	thymidylate synthetase (TYMS), mRNA.	-0.01	8.15	6.31E-01	6.70E-01	pink
UBE2M	9040	ubiquitin-conjugating enzyme E2M (UBC12 homolog, yeast) (UBE2M), mRNA.	-0.14	7.87	4.08E-14	1.29E-13	pink
UBR7	55148	chromosome 14 open reading frame 130 (C14orf130), transcript variant 1, mRNA.	0.21	9.42	2.26E-37	2.03E-36	pink

UHMK1	127933	U2AF homology motif (UHM) kinase 1 (UHMK1), mRNA.	0.12	10.44	1.04E-13	3.20E-13	pink
UROS	7390	uroporphyrinogen III synthase (congenital erythropoietic porphyria) (UROS), mRNA.	0.02	6.11	3.71E-01	4.16E-01	pink
USP13	8975	ubiquitin specific peptidase 13 (isopeptidase T-3) (USP13), mRNA.	0.09	6.16	6.91E-12	1.93E-11	pink
USP14	9097	ubiquitin specific peptidase 14 (tRNA-guanine transglycosylase) (USP14), transcript variant 1, mRNA.	0.01	7.47	6.54E-01	6.92E-01	pink
VDAC1	7416	voltage-dependent anion channel 1 (VDAC1), mRNA.	-0.03	9.81	4.60E-02	6.03E-02	pink
WDR54	84058	WD repeat domain 54 (WDR54), mRNA.	-0.36	7.86	1.96E-55	4.02E-54	pink
XPC	7508	xeroderma pigmentosum, complementation group C (XPC), mRNA.	0.01	6.92	2.32E-01	2.72E-01	pink
XXYLT1	152002	chromosome 3 open reading frame 21 (C3orf21), mRNA.	-0.21	9.34	7.41E-34	5.62E-33	pink
YIF1A	10897	Yip1 interacting factor homolog A (S. cerevisiae) (YIF1A), mRNA.	0.11	8.95	1.91E-10	4.93E-10	pink
YTHDC1	91746	YTH domain containing 1 (YTHDC1), transcript variant 2, mRNA.	0.10	8.40	5.65E-12	1.59E-11	pink
YTHDF2	51441	YTH domain family, member 2 (YTHDF2), mRNA.	0.07	6.59	1.82E-06	3.63E-06	pink

YWHAB	7529	tyrosine 3-monooxygenase/tryptophan 5-monooxygenase activation protein, beta polypeptide (YWHAB), transcript variant 2, mRNA.	-0.12	7.26	9.96E-17	3.64E-16	pink
ZFAND2A	90637	zinc finger, AN1-type domain 2A (ZFAND2A), mRNA.	0.15	7.53	1.27E-16	4.61E-16	pink
ZNF511	118472	zinc finger protein 511 (ZNF511), mRNA.	0.01	7.51	4.51E-01	4.98E-01	pink
ZNF558	148156	zinc finger protein 558 (ZNF558), mRNA.	-0.05	9.18	1.23E-02	1.74E-02	pink
ZNF593	51042	zinc finger protein 593 (ZNF593), mRNA.	0.11	8.12	2.80E-13	8.46E-13	pink
ZNF653	115950	zinc finger protein 653 (ZNF653), mRNA.	-0.14	7.38	5.38E-16	1.88E-15	pink
ZNF700	90592	zinc finger protein 700 (ZNF700), mRNA.	-0.01	7.89	6.70E-01	7.07E-01	pink
ZNF787	126208	zinc finger protein 787 (ZNF787), mRNA.	0.02	7.95	2.39E-01	2.79E-01	pink
A4GALT	53947	alpha 1,4-galactosyltransferase (globotriaosylceramide synthase) (A4GALT), mRNA.	-0.07	9.02	8.99E-06	1.72E-05	purple
ACSM3	6296	acyl-CoA synthetase medium-chain family member 3 (ACSM3), transcript variant 1, mRNA.	0.03	12.30	2.70E-02	3.65E-02	purple
ADIRF	10974	chromosome 10 open reading frame 116 (C10orf116), mRNA.	-0.19	8.36	1.26E-24	6.76E-24	purple

AK4	205	adenylate kinase 3-like 1 (AK3L1), nuclear gene encoding mitochondrial protein, transcript variant 7, mRNA.	-0.04	8.28	8.98E-03	1.29E-02	purple
ALPL	249	alkaline phosphatase, liver/bone/kidney (ALPL), mRNA.	0.13	7.53	5.74E-14	1.80E-13	purple
AMICA1	120425	adhesion molecule, interacts with CXADR antigen 1 (AMICA1), mRNA.	-0.05	10.35	1.00E-04	1.75E-04	purple
ANKRD13A	88455	ankyrin repeat domain 13A (ANKRD13A), mRNA.	-0.13	6.96	4.64E-19	1.90E-18	purple
ANO10	55129	transmembrane protein 16K (TMEM16K), mRNA.	-0.03	9.58	1.34E-02	1.88E-02	purple
ANXA11	311	annexin A11 (ANXA11), transcript variant b, mRNA.	-0.15	7.28	1.67E-21	7.65E-21	purple
ARHGAP10	79658	Rho GTPase activating protein 10 (ARHGAP10), mRNA.	0.05	7.53	1.28E-05	2.41E-05	purple
ARHGAP24	83478	Rho GTPase activating protein 24 (ARHGAP24), transcript variant 2, mRNA.	-0.01	6.06	5.06E-01	5.51E-01	purple
ARPC1A	10552	actin related protein 2/3 complex, subunit 1A, 41kDa (ARPC1A), mRNA.	-0.06	7.62	3.75E-03	5.59E-03	purple
ASRGL1	80150	asparaginase like 1 (ASRGL1), mRNA.	0.00	10.41	7.87E-01	8.15E-01	purple
ATP1B1	481	ATPase, Na+/K+ transporting, beta 1 polypeptide (ATP1B1), transcript variant 1, mRNA.	0.57	9.58	4.93E-56	1.04E-54	purple

BAIAP2L1	55971	BAI1-associated protein 2-like 1 (BAIAP2L1), mRNA.	-0.09	6.65	3.66E-13	1.10E-12	purple
BCAR1	9564	breast cancer anti-estrogen resistance 1 (BCAR1), mRNA.	-0.36	6.77	1.10E-31	7.65E-31	purple
BHLHE22	27319	basic helix-loop-helix domain containing, class B, 5 (BHLHB5), mRNA.	-0.10	9.31	7.22E-15	2.37E-14	purple
BMPR1A	283155	PREDICTED: hypothetical LOC283155 (LOC283155), mRNA.	-0.10	7.60	3.50E-12	9.95E-12	purple
C1orf106	55765	chromosome 1 open reading frame 106 (C1orf106), mRNA.	-0.10	8.31	4.28E-10	1.08E-09	purple
C2orf88	84281	hypothetical protein MGC13057 (MGC13057), transcript variant 2, mRNA.	0.01	8.73	7.70E-01	7.99E-01	purple
C3orf14	57415	chromosome 3 open reading frame 14 (C3orf14), mRNA.	0.04	8.29	6.14E-03	8.95E-03	purple
CACNB4	785	calcium channel, voltage-dependent, beta 4 subunit (CACNB4), transcript variant 1, mRNA.	0.23	6.32	4.61E-31	3.10E-30	purple
CASK	8573	calcium/calmodulin-dependent serine protein kinase (MAGUK family) (CASK), mRNA.	1.14	6.37	1.17E-68	4.89E-67	purple
CCDC126	90693	coiled-coil domain containing 126 (CCDC126), mRNA.	0.07	9.25	7.75E-04	1.24E-03	purple
CCDC64	92558	coiled-coil domain containing 64 (CCDC64), mRNA.	0.02	6.56	3.89E-01	4.34E-01	purple

CCND2	894	cyclin D2 (CCND2), mRNA.	0.28	8.52	4.64E-38	4.34E-37	purple
CD82	3732	CD82 molecule (CD82), transcript variant 1, mRNA.	-0.47	8.94	2.21E-58	5.49E-57	purple
CD9	928	CD9 molecule (CD9), mRNA.	-0.20	7.31	6.31E-25	3.41E-24	purple
CDC42EP4	23580	CDC42 effector protein (Rho GTPase binding) 4 (CDC42EP4), mRNA.	0.05	5.81	1.59E-04	2.72E-04	purple
CDH2	1000	cadherin 2, type 1, N-cadherin (neuronal) (CDH2), mRNA.	0.02	9.33	8.23E-02	1.04E-01	purple
CDK2AP1	8099	CDK2-associated protein 1 (CDK2AP1), mRNA.	0.04	6.18	2.71E-02	3.65E-02	purple
CEBPB	1051	CCAAT/enhancer binding protein (C/EBP), beta (CEBPB), mRNA.	-0.18	5.41	2.33E-17	8.75E-17	purple
CHDH	55349	choline dehydrogenase (CHDH), mRNA.	0.01	5.54	6.46E-01	6.84E-01	purple
COL5A1	1289	collagen, type V, alpha 1 (COL5A1), mRNA.	-0.12	8.27	2.62E-12	7.50E-12	purple
CPXM1	56265	carboxypeptidase X (M14 family), member 1 (CPXM1), mRNA.	-0.10	6.64	8.26E-11	2.17E-10	purple
CRYL1	51084	crystallin, lambda 1 (CRYL1), mRNA.	0.10	8.54	5.18E-10	1.30E-09	purple
CRYM	1428	crystallin, mu (CRYM), transcript variant 2, mRNA.	0.03	8.41	8.27E-02	1.05E-01	purple
CSRP2	1466	cysteine and glycine-rich protein 2 (CSRP2), mRNA.	-0.36	7.15	4.88E-49	7.41E-48	purple
CST3	1471	cystatin C (amyloid angiopathy and cerebral hemorrhage) (CST3), mRNA.	0.01	7.81	4.51E-01	4.97E-01	purple

CTNNA2	1496	catenin (cadherin-associated protein), alpha 2 (CTNNA2), mRNA.	0.02	5.99	3.64E-01	4.10E-01	purple
DAAM2	23500	dishevelled associated activator of morphogenesis 2 (DAAM2), mRNA.	-0.05	8.50	1.05E-03	1.66E-03	purple
DACT1	51339	dapper, antagonist of beta-catenin, homolog 1 (Xenopus laevis) (DACT1), transcript variant 2, mRNA.	-0.01	10.29	4.24E-01	4.70E-01	purple
DBNDD1	79007	dysbindin (dystrobrevin binding protein 1) domain containing 1 (DBNDD1), transcript variant 2, mRNA.	-0.06	5.83	2.50E-03	3.79E-03	purple
DLL1	28514	delta-like 1 (Drosophila) (DLL1), mRNA.	-0.07	9.41	1.05E-04	1.82E-04	purple
DNAJC12	56521	DnaJ (Hsp40) homolog, subfamily C, member 12 (DNAJC12), transcript variant 2, mRNA.	0.16	7.71	6.64E-20	2.82E-19	purple
EOMES	8320	eomesodermin homolog (Xenopus laevis) (EOMES), mRNA.	0.05	6.91	4.23E-03	6.27E-03	purple
EPB41L5	57669	erythrocyte membrane protein band 4.1 like 5 (EPB41L5), mRNA.	0.04	12.00	2.46E-02	3.34E-02	purple
EPDR1	54749	ependymin related protein 1 (zebrafish) (EPDR1), mRNA.	0.35	6.79	1.86E-28	1.15E-27	purple
EPHB1	2047	EPH receptor B1 (EPHB1), mRNA.	-0.14	8.27	1.24E-17	4.74E-17	purple

ETV5	2119	ets variant gene 5 (ets-related molecule) (ETV5), mRNA.	0.06	8.42	3.91E-05	7.06E-05	purple
F12	2161	coagulation factor XII (Hageman factor) (F12), mRNA.	-0.23	6.20	1.10E-32	8.04E-32	purple
FAM46A	55603	family with sequence similarity 46, member A (FAM46A), mRNA.	0.08	6.11	5.38E-05	9.59E-05	purple
FGGY	55277	hypothetical protein FLJ10986 (FLJ10986), mRNA.	-0.06	6.03	2.09E-05	3.87E-05	purple
FJX1	24147	four jointed box 1 (Drosophila) (FJX1), mRNA.	-0.09	8.28	2.39E-04	4.03E-04	purple
FOXA3	3171	forkhead box A3 (FOXA3), mRNA.	0.01	7.74	5.76E-01	6.19E-01	purple
GABARAPL1	23710	GABA(A) receptor-associated protein like 1 (GABARAPL1), mRNA.	0.30	6.53	2.30E-37	2.07E-36	purple
GADD45A	1647	growth arrest and DNA-damage-inducible, alpha (GADD45A), mRNA.	-0.10	8.35	7.13E-17	2.62E-16	purple
GAS2	2620	growth arrest-specific 2 (GAS2), transcript variant 2, mRNA.	-0.01	9.70	5.94E-01	6.36E-01	purple
GCHFR	2644	GTP cyclohydrolase I feedback regulator (GCHFR), mRNA.	-0.17	8.60	1.41E-16	5.08E-16	purple
GCNT3	9245	glucosaminyl (N-acetyl) transferase 3, mucin type (GCNT3), mRNA.	-0.10	5.83	8.33E-11	2.19E-10	purple
GPC4	2239	glypican 4 (GPC4), mRNA.	-0.02	8.22	2.14E-01	2.53E-01	purple

GRHPR	9380	glyoxylate reductase/hydroxypyruvate reductase (GRHPR), mRNA.	0.29	6.60	9.42E-42	1.03E-40	purple
GRM8	2918	glutamate receptor, metabotropic 8 (GRM8), mRNA.	-0.07	9.47	1.24E-06	2.50E-06	purple
GSTM4	2948	glutathione S-transferase M4 (GSTM4), transcript variant 1, mRNA.	0.06	6.50	1.22E-03	1.91E-03	purple
HACD1	9200	protein tyrosine phosphatase-like (proline instead of catalytic arginine), member A (PTPLA), mRNA.	0.08	9.83	2.22E-07	4.74E-07	purple
HBD	3045	hemoglobin, delta (HBD), mRNA.	0.00	6.43	9.83E-01	9.86E-01	purple
HES5	388585	hairy and enhancer of split 5 (Drosophila) (HES5), mRNA.	0.01	5.87	3.11E-01	3.56E-01	purple
HEY1	23462	hairy/enhancer-of-split related with YRPW motif 1 (HEY1), transcript variant 1, mRNA.	0.03	8.23	1.87E-01	2.23E-01	purple
HNF1B	6928	transcription factor 2, hepatic; LF-B3; variant hepatic nuclear factor (TCF2), transcript variant a, mRNA.	-0.04	7.84	7.52E-02	9.58E-02	purple
HOOK1	51361	hook homolog 1 (Drosophila) (HOOK1), mRNA.	0.04	7.79	6.96E-05	1.23E-04	purple
HOXB2	3212	homeobox B2 (HOXB2), mRNA.	-0.01	8.47	7.88E-01	8.15E-01	purple
HOXB4	3214	homeobox B4 (HOXB4), mRNA.	0.02	10.34	5.79E-02	7.49E-02	purple
HOXB7	3217	homeobox B7 (HOXB7), mRNA.	-0.12	7.51	8.01E-14	2.49E-13	purple
HRSP12	10247	heat-responsive protein 12 (HRSP12), mRNA.	0.00	7.81	6.79E-01	7.14E-01	purple

HVCN1	84329	hydrogen voltage-gated channel 1 (HVCN1), transcript variant 1, mRNA.	0.00	5.86	9.66E-01	9.72E-01	purple
IL17RB	55540	interleukin 17 receptor B (IL17RB), mRNA.	0.04	6.61	1.48E-02	2.06E-02	purple
INPP5A	3632	inositol polyphosphate-5-phosphatase, 40kDa (INPP5A), mRNA.	0.05	7.82	9.39E-06	1.79E-05	purple
IPCEF1	26034	phosphoinositide-binding protein PIP3-E (PIP3-E), mRNA.	-0.01	12.31	3.61E-01	4.06E-01	purple
KIAA0226L	80183	chromosome 13 open reading frame 18 (C13orf18), mRNA.	0.03	9.08	3.84E-02	5.08E-02	purple
LAD1	3898	ladinin 1 (LAD1), mRNA.	0.02	10.09	1.15E-01	1.43E-01	purple
LFNG	3955	LFNG O-fucosylpeptide 3-beta-N-acetylglucosaminyltransferase (LFNG), transcript variant 1, mRNA.	-0.22	9.00	1.36E-44	1.69E-43	purple
LGALS14	56891	lectin, galactoside-binding, soluble, 14 (LGALS14), transcript variant 2, mRNA.	-0.14	9.74	3.09E-15	1.03E-14	purple
LGALS3BP	3959	lectin, galactoside-binding, soluble, 3 binding protein (LGALS3BP), mRNA.	-0.46	7.48	3.99E-73	2.06E-71	purple
LIN7A	8825	lin-7 homolog A (C. elegans) (LIN7A), mRNA.	0.00	10.28	6.34E-01	6.73E-01	purple
LMO3	55885	LIM domain only 3 (rhombotin-like 2) (LMO3), transcript variant 2, mRNA.	0.00	6.71	9.03E-01	9.19E-01	purple

MAP1B	4131	microtubule-associated protein 1B (MAP1B), transcript variant 1, mRNA.	0.05	10.23	7.38E-04	1.18E-03	purple
MBD4	8930	methyl-CpG binding domain protein 4 (MBD4), mRNA.	0.10	10.06	1.23E-11	3.38E-11	purple
MDK	4192	midkine (neurite growth-promoting factor 2) (MDK), transcript variant 2, mRNA.	-0.15	10.50	2.93E-19	1.21E-18	purple
MEF2C	4208	MADS box transcription enhancer factor 2, polypeptide C (myocyte enhancer factor 2C) (MEF2C), mRNA.	0.06	8.03	9.27E-05	1.62E-04	purple
MGST3	4259	microsomal glutathione S-transferase 3 (MGST3), mRNA.	0.06	10.45	9.15E-04	1.46E-03	purple
MMP7	4316	matrix metallopeptidase 7 (matrilysin, uterine) (MMP7), mRNA.	-0.15	8.79	2.21E-21	1.00E-20	purple
MNX1	3110	homeobox HB9 (HLXB9), mRNA.	0.08	6.67	2.00E-06	4.00E-06	purple
MPC2	25874	brain protein 44 (BRP44), mRNA.	-0.06	7.30	6.51E-07	1.34E-06	purple
MPP1	4354	membrane protein, palmitoylated 1, 55kDa (MPP1), mRNA.	0.07	9.24	5.20E-06	1.01E-05	purple
MSRB1	51734	selenoprotein X, 1 (SEPX1), mRNA.	-0.09	7.64	1.04E-08	2.41E-08	purple
MT1G	4495	metallothionein 1G (MT1G), mRNA.	-0.09	6.89	9.18E-10	2.27E-09	purple
MUC13	56667	mucin 13, cell surface associated (MUC13), mRNA.	1.61	9.14	7.38E-93	1.97E-90	purple
MYH10	4628	myosin, heavy chain 10, non-muscle (MYH10), mRNA.	0.00	8.84	9.65E-01	9.70E-01	purple
MYO6	4646	myosin VI (MYO6), mRNA.	0.08	9.01	4.28E-07	8.94E-07	purple

NA	26872	six transmembrane epithelial antigen of the prostate 1 (STEAP1), mRNA.	-0.35	7.00	6.35E-44	7.63E-43	purple
NA	441519	cancer/testis antigen CT45-3 (CT45-3), mRNA.	0.22	8.26	1.50E-31	1.04E-30	purple
NA	388325	DTFT5783 (UNQ5783), mRNA.	-0.16	7.65	5.01E-23	2.47E-22	purple
NA	140851	chromosome 20 open reading frame 127 (C20orf127), mRNA.	0.07	7.77	1.50E-06	3.02E-06	purple
NA	29970	schwannomin interacting protein 1 (SCHIP1), mRNA.	-0.03	10.88	3.09E-01	3.54E-01	purple
NREP	9315	chromosome 5 open reading frame 13 (C5orf13), mRNA.	-0.20	5.72	2.51E-24	1.31E-23	purple
NRGN	4900	neurogranin (protein kinase C substrate, RC3) (NRGN), mRNA.	-0.10	7.48	1.65E-12	4.78E-12	purple
NRIP1	8204	nuclear receptor interacting protein 1 (NRIP1), mRNA.	0.11	5.65	6.29E-09	1.49E-08	purple
NT5C3B	115024	5'-nucleotidase, cytosolic III-like (NT5C3L), mRNA.	-0.05	11.11	6.31E-04	1.02E-03	purple
NUMB	8650	numb homolog (Drosophila) (NUMB), transcript variant 1, mRNA.	0.09	7.70	1.20E-12	3.51E-12	purple
PARD6G	84552	par-6 partitioning defective 6 homolog gamma (C. elegans) (PARD6G), mRNA.	0.41	5.46	3.38E-30	2.20E-29	purple

PCBD1	5092	pterin-4 alpha-carbinolamine dehydratase/dimerization cofactor of hepatocyte nuclear factor 1 alpha (TCF1) (PCBD1), mRNA.	0.03	10.22	1.59E-01	1.92E-01	purple
PHLDA1	22822	pleckstrin homology-like domain, family A, member 1 (PHLDA1), mRNA.	-0.18	7.70	4.25E-20	1.83E-19	purple
PLA1A	51365	phospholipase A1 member A (PLA1A), mRNA.	-0.02	9.02	2.66E-01	3.09E-01	purple
PLCL2	23228	phospholipase C-like 2 (PLCL2), mRNA.	-0.06	8.30	1.74E-05	3.24E-05	purple
PLOD2	5352	procollagen-lysine, 2-oxoglutarate 5-dioxygenase 2 (PLOD2), transcript variant 1, mRNA.	-0.03	7.36	2.82E-02	3.78E-02	purple
PMEPA1	56937	transmembrane, prostate androgen induced RNA (TMEPAI), transcript variant 4, mRNA.	-0.08	7.44	6.64E-06	1.28E-05	purple
PPIC	5480	peptidylprolyl isomerase C (cyclophilin C) (PPIC), mRNA.	0.29	7.70	3.43E-36	2.92E-35	purple
PRKCDBP	112464	protein kinase C, delta binding protein (PRKCDBP), mRNA.	-0.01	9.91	2.80E-01	3.23E-01	purple
RAB27A	5873	RAB27A, member RAS oncogene family (RAB27A), transcript variant 3, mRNA.	-0.27	9.97	2.72E-40	2.77E-39	purple
RAB31	11031	RAB31, member RAS oncogene family (RAB31), mRNA.	-0.23	8.15	2.74E-49	4.21E-48	purple

RAB38	23682	RAB38, member RAS oncogene family (RAB38), mRNA.	-0.16	7.91	1.26E-20	5.56E-20	purple
RAB40B	10966	RAB40B, member RAS oncogene family (RAB40B), mRNA.	-0.13	8.20	3.52E-21	1.59E-20	purple
RASGRP1	10125	RAS guanyl releasing protein 1 (calcium and DAG-regulated) (RASGRP1), mRNA.	-0.03	7.69	1.81E-02	2.49E-02	purple
RBPMS2	348093	RNA binding protein with multiple splicing 2 (RBPMS2), mRNA.	-0.01	7.37	7.12E-01	7.46E-01	purple
RENBP	5973	renin binding protein (RENBP), mRNA.	-0.02	6.25	9.23E-02	1.16E-01	purple
RHOU	58480	ras homolog gene family, member U (RHOU), mRNA.	0.07	8.97	4.61E-04	7.56E-04	purple
RIMS3	9783	regulating synaptic membrane exocytosis 3 (RIMS3), mRNA.	0.06	10.39	8.75E-05	1.53E-04	purple
RTKN	6242	rhotekin (RTKN), transcript variant 1, mRNA.	-0.15	9.18	2.16E-24	1.14E-23	purple
S100A4	6275	S100 calcium binding protein A4 (S100A4), transcript variant 1, mRNA.	0.14	6.65	4.98E-22	2.33E-21	purple
S100A6	6277	S100 calcium binding protein A6 (S100A6), mRNA.	0.14	8.29	3.18E-10	8.08E-10	purple
SASH1	23328	SAM and SH3 domain containing 1 (SASH1), mRNA.	0.12	9.64	1.26E-10	3.27E-10	purple
SCPEP1	59342	serine carboxypeptidase 1 (SCPEP1), mRNA.	0.04	8.93	1.46E-02	2.04E-02	purple
SDC4	6385	syndecan 4 (SDC4), mRNA.	-0.24	7.15	4.80E-40	4.82E-39	purple

SERPINE2	5270	serpin peptidase inhibitor, clade E (nexin, plasminogen activator inhibitor type 1), member 2 (SERPINE2), mRNA.	0.51	7.33	1.10E-63	3.76E-62	purple
SGPP1	81537	sphingosine-1-phosphate phosphatase 1 (SGPP1), mRNA.	-0.08	9.60	2.77E-05	5.08E-05	purple
SH2D2A	9047	SH2 domain protein 2A (SH2D2A), mRNA.	-0.04	7.07	1.27E-02	1.78E-02	purple
SIT1	27240	signaling threshold regulating transmembrane adaptor 1 (SIT1), mRNA.	0.02	6.31	1.91E-01	2.27E-01	purple
SNX10	29887	sorting nexin 10 (SNX10), mRNA.	0.02	10.60	1.62E-01	1.96E-01	purple
SORL1	6653	sortilin-related receptor, L(DLR class) A repeats-containing (SORL1), mRNA.	-0.05	7.84	3.73E-03	5.56E-03	purple
SPRY2	10253	sprouty homolog 2 (Drosophila) (SPRY2), mRNA.	0.05	8.76	4.70E-03	6.93E-03	purple
SSBP2	23635	single-stranded DNA binding protein 2 (SSBP2), mRNA.	-0.13	6.24	6.48E-11	1.72E-10	purple
ST8SIA5	29906	ST8 alpha-N-acetyl-neuraminide alpha-2,8-sialyltransferase 5 (ST8SIA5), mRNA.	0.05	10.53	1.08E-05	2.05E-05	purple
STARD5	80765	START domain containing 5 (STARD5), mRNA.	0.02	7.13	1.36E-01	1.66E-01	purple
STC2	8614	stanniocalcin 2 (STC2), mRNA.	0.20	6.09	7.46E-18	2.88E-17	purple
STK3	6788	serine/threonine kinase 3 (STE20 homolog, yeast) (STK3), mRNA.	0.29	7.39	1.39E-39	1.37E-38	purple

SVIL	6840	supervillin (SVIL), transcript variant 2, mRNA.	0.30	6.23	1.44E-33	1.08E-32	purple
SYT11	23208	synaptotagmin XI (SYT11), mRNA.	-0.02	6.62	2.20E-01	2.59E-01	purple
TBX15	6913	T-box 15 (TBX15), mRNA.	-0.11	5.36	2.54E-08	5.76E-08	purple
TCN2	6948	transcobalamin II; macrocytic anemia (TCN2), mRNA.	-0.06	8.94	5.41E-05	9.64E-05	purple
TIMD4	91937	T-cell immunoglobulin and mucin domain containing 4 (TIMD4), mRNA.	0.06	6.85	6.42E-04	1.04E-03	purple
TM6SF1	53346	transmembrane 6 superfamily member 1 (TM6SF1), mRNA.	0.06	7.91	6.86E-04	1.10E-03	purple
TMEM119	338773	transmembrane protein 119 (TMEM119), mRNA.	0.08	6.64	2.61E-05	4.79E-05	purple
TMEM123	114908	transmembrane protein 123 (TMEM123), mRNA.	0.10	8.66	8.26E-08	1.81E-07	purple
TMEM230	29058	chromosome 20 open reading frame 30 (C20orf30), transcript variant 2, mRNA.	0.17	9.39	4.36E-26	2.47E-25	purple
TMEM51	55092	transmembrane protein 51 (TMEM51), mRNA.	0.28	8.04	1.61E-29	1.02E-28	purple
TNFRSF21	27242	tumor necrosis factor receptor superfamily, member 21 (TNFRSF21), mRNA.	0.09	9.82	2.46E-05	4.52E-05	purple
TNFSF12	8742	tumor necrosis factor (ligand) superfamily, member 12 (TNFSF12), mRNA.	0.18	5.98	1.87E-22	8.88E-22	purple

TOX	9760	thymus high mobility group box protein TOX (TOX), mRNA.	-0.01	8.72	1.99E-01	2.37E-01	purple
UBD	10537	ubiquitin D (UBD), mRNA.	0.18	6.19	5.18E-26	2.92E-25	purple
UPP1	7378	uridine phosphorylase 1 (UPP1), transcript variant 2, mRNA.	-0.01	6.88	3.99E-01	4.45E-01	purple
VCAN	1462	versican (VCAN), mRNA.	0.17	6.65	1.29E-20	5.68E-20	purple
YES1	7525	v-yes-1 Yamaguchi sarcoma viral oncogene homolog 1 (YES1), mRNA.	0.15	7.85	3.15E-18	1.23E-17	purple
ZNF503	84858	zinc finger protein 503 (ZNF503), mRNA.	-0.02	6.30	1.73E-01	2.08E-01	purple
MARCH3	115123	membrane-associated ring finger (C3HC4) 3 (MARCH3), mRNA.	-0.12	9.07	8.23E-15	2.70E-14	red
SEPT3	55964	septin 3 (SEPT3), transcript variant A, mRNA.	0.41	7.11	2.37E-51	3.92E-50	red
AAK1	388957	similar to BMP2 inducible kinase (DKFZp686K16132), mRNA.	-0.12	7.97	1.08E-19	4.53E-19	red
ACY3	91703	aspartoacylase (aminocyclase) 3 (ACY3), mRNA.	0.28	9.28	7.85E-35	6.20E-34	red
ADAR	103	adenosine deaminase, RNA-specific (ADAR), transcript variant 3, mRNA.	-0.03	6.83	6.15E-02	7.93E-02	red
ADI1	55256	acireductone dioxygenase 1 (ADI1), mRNA.	-0.01	5.97	3.36E-01	3.81E-01	red
ADORA2A	135	adenosine A2a receptor (ADORA2A), mRNA.	0.05	6.11	1.12E-04	1.94E-04	red

AGPAT9	84803	lung cancer metastasis-associated protein (MAG1), mRNA.	0.02	5.39	3.13E-01	3.57E-01	red
AIF1	199	allograft inflammatory factor 1 (AIF1), transcript variant 1, mRNA.	0.02	12.71	2.63E-01	3.05E-01	red
ALDH7A1	501	aldehyde dehydrogenase 7 family, member A1 (ALDH7A1), mRNA.	-0.14	7.32	1.96E-20	8.57E-20	red
ALOX5	240	arachidonate 5-lipoxygenase (ALOX5), mRNA.	0.17	7.11	3.93E-24	2.04E-23	red
AMER1	139285	family with sequence similarity 123B (FAM123B), mRNA.	0.05	9.86	1.25E-04	2.16E-04	red
ANAPC13	25847	anaphase promoting complex subunit 13 (ANAPC13), mRNA.	-0.02	6.98	2.93E-01	3.37E-01	red
APOBEC3G	60489	apolipoprotein B mRNA editing enzyme, catalytic polypeptide-like 3G (APOBEC3G), mRNA.	0.11	6.22	8.31E-08	1.82E-07	red
APP	351	amyloid beta (A4) precursor protein (peptidase nexin-II, Alzheimer disease) (APP), transcript variant 2, mRNA.	-0.23	7.70	1.56E-26	8.97E-26	red
ARHGDIB	397	Rho GDP dissociation inhibitor (GDI) beta (ARHGDIB), mRNA.	-0.18	9.14	1.41E-26	8.12E-26	red
ARL2BP	23568	ADP-ribosylation factor-like 2 binding protein (ARL2BP), mRNA.	-0.06	8.99	1.73E-03	2.68E-03	red

ARMCX1	51309	armadillo repeat containing, X-linked 1 (ARMCX1), mRNA.	-0.02	9.44	1.87E-01	2.23E-01	red
ASPHD2	57168	aspartate beta-hydroxylase domain containing 2 (ASPHD2), mRNA.	0.00	7.55	7.72E-01	8.01E-01	red
ATF3	467	activating transcription factor 3 (ATF3), transcript variant 1, mRNA.	0.01	11.40	4.41E-01	4.88E-01	red
ATP6V1A	523	ATPase, H+ transporting, lysosomal 70kDa, V1 subunit A (ATP6V1A), mRNA.	0.09	6.74	5.31E-11	1.41E-10	red
BATF	10538	basic leucine zipper transcription factor, ATF-like (BATF), mRNA.	-0.17	6.55	2.55E-36	2.19E-35	red
BBS2	583	Bardet-Biedl syndrome 2 (BBS2), mRNA.	0.03	6.96	1.52E-02	2.11E-02	red
BCL11A	53335	B-cell CLL/lymphoma 11A (zinc finger protein) (BCL11A), transcript variant 1, mRNA.	0.22	5.37	2.70E-15	9.06E-15	red
BCL2L1	598	BCL2-like 1 (BCL2L1), nuclear gene encoding mitochondrial protein, transcript variant 2, mRNA.	-0.82	7.35	1.09E-73	5.80E-72	red
BIN2	51411	bridging integrator 2 (BIN2), mRNA.	-0.02	9.19	2.20E-01	2.59E-01	red
BLK	640	B lymphoid tyrosine kinase (BLK), mRNA.	-0.33	6.40	5.91E-37	5.21E-36	red
BSPRY	54836	B-box and SPRY domain containing (BSPRY), mRNA.	-0.25	6.99	4.60E-38	4.32E-37	red

BST2	684	bone marrow stromal cell antigen 2 (BST2), mRNA.	-0.09	7.64	6.06E-08	1.34E-07	red
C12orf57	113246	chromosome 12 open reading frame 57 (C12orf57), mRNA.	0.00	8.35	8.74E-01	8.93E-01	red
C19orf66	55337	hypothetical protein FLJ11286 (FLJ11286), mRNA.	0.06	7.97	6.17E-04	9.98E-04	red
C1orf162	128346	chromosome 1 open reading frame 162 (C1orf162), mRNA.	-0.04	9.84	1.54E-03	2.38E-03	red
C1orf53	388722	chromosome 1 open reading frame 53 (C1orf53), mRNA.	0.39	7.15	6.99E-47	9.89E-46	red
C22orf39	128977	hypothetical protein LOC128977 (LOC128977), mRNA.	-0.01	8.55	6.08E-01	6.49E-01	red
C5orf30	90355	chromosome 5 open reading frame 30 (C5orf30), mRNA.	-0.17	7.55	4.15E-27	2.45E-26	red
C9orf78	51759	chromosome 9 open reading frame 78 (C9orf78), mRNA.	0.11	10.53	1.08E-15	3.73E-15	red
CAB39L	81617	calcium binding protein 39-like (CAB39L), transcript variant 2, mRNA.	0.03	7.99	1.29E-01	1.58E-01	red
CAMK2D	817	calcium/calmodulin-dependent protein kinase (CaM kinase) II delta (CAMK2D), transcript variant 4, mRNA.	0.21	8.57	1.45E-22	6.98E-22	red

CAMKK2	10645	calcium/calmodulin-dependent protein kinase kinase 2, beta (CAMKK2), transcript variant 6, mRNA.	0.06	8.31	1.02E-04	1.78E-04	red
CASP1	834	caspase 1, apoptosis-related cysteine peptidase (interleukin 1, beta, convertase) (CASP1), transcript variant gamma, mRNA.	0.06	9.52	8.64E-05	1.51E-04	red
CASP3	836	caspase 3, apoptosis-related cysteine peptidase (CASP3), transcript variant alpha, mRNA.	-0.06	9.17	2.18E-03	3.33E-03	red
CCNE2	9134	cyclin E2 (CCNE2), transcript variant 1, mRNA.	0.07	8.16	5.25E-05	9.36E-05	red
CD38	952	CD38 molecule (CD38), mRNA.	0.05	6.55	1.35E-03	2.10E-03	red
CD72	971	CD72 molecule (CD72), mRNA.	0.11	6.32	4.54E-08	1.01E-07	red
CD99	4267	CD99 molecule (CD99), mRNA.	-0.02	8.38	1.45E-01	1.76E-01	red
CDK5R1	8851	cyclin-dependent kinase 5, regulatory subunit 1 (p35) (CDK5R1), mRNA.	0.07	9.66	2.03E-06	4.06E-06	red
CDKN2C	1031	cyclin-dependent kinase inhibitor 2C (p18, inhibits CDK4) (CDKN2C), transcript variant 2, mRNA.	0.58	9.86	3.28E-60	9.14E-59	red
CELSR3	1951	cadherin, EGF LAG seven-pass G-type receptor 3 (flamingo homolog, Drosophila) (CELSR3), mRNA.	0.12	9.54	2.30E-18	9.03E-18	red
CHMP5	51510	chromatin modifying protein 5 (CHMP5), mRNA.	-0.06	6.89	3.94E-06	7.73E-06	red

CKB	1152	creatine kinase, brain (CKB), mRNA.	-0.26	5.91	1.87E-33	1.39E-32	red
CLINT1	9685	clathrin interactor 1 (CLINT1), mRNA.	-0.06	6.55	1.56E-04	2.67E-04	red
CMTM7	112616	CKLF-like MARVEL transmembrane domain containing 7 (CMTM7), transcript variant 1, mRNA.	0.13	6.99	5.40E-15	1.79E-14	red
COL4A3BP	10087	collagen, type IV, alpha 3 (Goodpasture antigen) binding protein (COL4A3BP), transcript variant 2, mRNA.	0.20	6.02	1.25E-24	6.67E-24	red
CORO2A	7464	coronin, actin binding protein, 2A (CORO2A), transcript variant 1, mRNA.	0.00	10.99	9.15E-01	9.28E-01	red
CPEB4	80315	cytoplasmic polyadenylation element binding protein 4 (CPEB4), mRNA.	-0.07	10.84	3.22E-08	7.24E-08	red
CSNK2A2	1459	casein kinase 2, alpha prime polypeptide (CSNK2A2), mRNA.	-0.08	7.57	6.63E-08	1.46E-07	red
CSRNP1	64651	AXIN1 up-regulated 1 (AXUD1), mRNA.	0.11	7.89	2.00E-13	6.08E-13	red
CXorf38	159013	chromosome X open reading frame 38 (CXorf38), mRNA.	0.18	7.81	1.85E-15	6.24E-15	red
CYB5B	80777	cytochrome b5 type B (outer mitochondrial membrane) (CYB5B), mRNA.	-0.17	8.27	5.10E-22	2.39E-21	red

CYFIP1	23191	cytoplasmic FMR1 interacting protein 1 (CYFIP1), transcript variant 2, mRNA.	0.17	7.19	1.02E-22	4.95E-22	red
CYP2J2	1573	cytochrome P450, family 2, subfamily J, polypeptide 2 (CYP2J2), mRNA.	0.05	6.01	2.90E-03	4.35E-03	red
DAD1	1603	defender against cell death 1 (DAD1), mRNA.	0.07	10.53	2.58E-06	5.11E-06	red
DAPP1	27071	dual adaptor of phosphotyrosine and 3-phosphoinositides (DAPP1), mRNA.	0.26	9.18	5.12E-42	5.70E-41	red
DDT	1652	D-dopachrome tautomerase (DDT), mRNA.	0.18	8.46	1.76E-18	6.97E-18	red
DDX60	55601	hypothetical protein FLJ20035 (FLJ20035), mRNA.	0.02	11.98	3.18E-01	3.62E-01	red
DEGS1	8560	degenerative spermatocyte homolog 1, lipid desaturase (Drosophila) (DEGS1), transcript variant 1, mRNA.	-0.27	7.21	1.01E-37	9.26E-37	red
DENND3	22898	DENN/MADD domain containing 3 (DENND3), mRNA.	0.13	6.60	1.92E-17	7.26E-17	red
DESI1	27351	DNA segment, Chr 15, Wayne State University 75, expressed (D15Wsu75e), mRNA.	-0.05	10.15	2.08E-05	3.85E-05	red
DFNA5	1687	deafness, autosomal dominant 5 (DFNA5), mRNA.	0.41	7.53	7.35E-35	5.82E-34	red
DHRS7	51635	dehydrogenase/reductase (SDR family) member 7 (DHRS7), mRNA.	0.00	10.16	6.75E-01	7.11E-01	red

DHRS9	10170	dehydrogenase/reductase (SDR family) member 9 (DHRS9), transcript variant 2, mRNA.	-0.08	7.51	6.43E-06	1.24E-05	red
DHX58	79132	likely ortholog of mouse D11lgp2 (LGP2), mRNA.	0.40	5.54	1.89E-26	1.08E-25	red
DLL3	10683	delta-like 3 (Drosophila) (DLL3), transcript variant 2, mRNA.	-0.05	6.58	1.24E-05	2.35E-05	red
DNAJB6	10049	DnaJ (Hsp40) homolog, subfamily B, member 6 (DNAJB6), transcript variant 1, mRNA.	0.02	5.83	2.93E-01	3.37E-01	red
DUSP5	1847	dual specificity phosphatase 5 (DUSP5), mRNA.	-0.45	6.53	4.79E-60	1.32E-58	red
E2F3	1871	E2F transcription factor 3 (E2F3), mRNA.	-0.56	10.67	6.18E-86	8.48E-84	red
ECE2	84291	hypothetical protein MGC2408 (MGC2408), mRNA.	-0.04	8.45	7.30E-03	1.06E-02	red
EIF2AK2	5610	eukaryotic translation initiation factor 2-alpha kinase 2 (EIF2AK2), mRNA.	-0.13	7.94	2.39E-12	6.85E-12	red
ELK1	2002	ELK1, member of ETS oncogene family (ELK1), mRNA.	0.58	9.11	3.24E-73	1.69E-71	red
EPSTI1	94240	epithelial stromal interaction 1 (breast) (EPSTI1), transcript variant 1, mRNA.	0.13	9.49	7.81E-20	3.31E-19	red
FAM102A	399665	family with sequence similarity 102, member A (FAM102A), transcript variant 2, mRNA.	-0.06	6.86	1.56E-04	2.67E-04	red

FAM107B	83641	family with sequence similarity 107, member B (FAM107B), mRNA.	-0.04	10.68	2.87E-03	4.31E-03	red
FAM83D	81610	family with sequence similarity 83, member D (FAM83D), mRNA.	0.02	8.78	1.09E-01	1.36E-01	red
FARS2	10667	phenylalanyl-tRNA synthetase 2, mitochondrial (FARS2), nuclear gene encoding mitochondrial protein, mRNA.	0.01	11.58	2.90E-01	3.34E-01	red
FBXO6	26270	F-box protein 6 (FBXO6), mRNA.	-0.14	6.02	4.72E-14	1.49E-13	red
FERMT2	10979	pleckstrin homology domain containing, family C (with FERM domain) member 1 (PLEKHC1), mRNA.	-0.13	7.97	2.01E-16	7.22E-16	red
FGL2	10875	fibrinogen-like 2 (FGL2), mRNA.	-0.05	5.49	2.43E-04	4.09E-04	red
FHL3	2275	four and a half LIM domains 3 (FHL3), mRNA.	-0.09	9.75	3.17E-13	9.57E-13	red
FLOT2	2319	flotillin 2 (FLOT2), mRNA.	-0.09	7.78	2.52E-07	5.36E-07	red
FOXO3	2309	forkhead box O3A (FOXO3A), transcript variant 1, mRNA.	-0.21	7.51	1.03E-23	5.27E-23	red
FYN	2534	FYN oncogene related to SRC, FGR, YES (FYN), transcript variant 1, mRNA.	-0.16	7.18	4.59E-21	2.05E-20	red
FZD6	8323	frizzled homolog 6 (Drosophila) (FZD6), mRNA.	-0.37	6.58	3.32E-53	5.89E-52	red

GBP1	2633	guanylate binding protein 1, interferon-inducible, 67kDa (GBP1), mRNA.	-0.11	7.54	3.32E-14	1.06E-13	red
GBP2	2634	guanylate binding protein 2, interferon-inducible (GBP2), mRNA.	0.03	6.98	4.44E-02	5.84E-02	red
GBP4	115361	guanylate binding protein 4 (GBP4), mRNA.	0.03	9.44	2.77E-02	3.73E-02	red
GBP5	115362	guanylate binding protein 5 (GBP5), mRNA.	-0.03	8.55	9.06E-02	1.14E-01	red
GINM1	116254	chromosome 6 open reading frame 72 (C6orf72), mRNA.	0.07	8.28	1.76E-08	4.04E-08	red
GJB2	2706	gap junction protein, beta 2, 26kDa (GJB2), mRNA.	0.04	10.07	2.08E-02	2.85E-02	red
GK	2710	glycerol kinase (GK), transcript variant 2, mRNA.	0.08	7.92	1.91E-07	4.09E-07	red
GLDC	2731	glycine dehydrogenase (decarboxylating) (GLDC), mRNA.	0.01	6.67	6.47E-01	6.85E-01	red
GLYATL2	219970	glycine-N-acyltransferase-like 2 (GLYATL2), mRNA.	-0.07	8.53	7.27E-06	1.39E-05	red
GMPR	2766	guanosine monophosphate reductase (GMPR), mRNA.	0.09	8.37	1.33E-06	2.68E-06	red
GNA15	2769	guanine nucleotide binding protein (G protein), alpha 15 (Gq class) (GNA15), mRNA.	0.03	11.34	4.26E-02	5.61E-02	red
GNPDA1	10007	glucosamine-6-phosphate deaminase 1 (GNPDA1), mRNA.	-0.03	8.13	1.38E-01	1.68E-01	red

GNS	2799	glucosamine (N-acetyl)-6-sulfatase (Sanfilippo disease IIID) (GNS), mRNA.	0.07	8.79	1.32E-04	2.28E-04	red
GPER1	2852	G protein-coupled receptor 30 (GPR30), transcript variant 2, mRNA.	-0.01	6.96	3.24E-01	3.69E-01	red
GPNMB	10457	glycoprotein (transmembrane) nmb (GPNMB), transcript variant 1, mRNA.	0.01	6.76	4.76E-01	5.21E-01	red
GPR180	160897	G protein-coupled receptor 180 (GPR180), mRNA.	-0.09	6.16	3.41E-05	6.19E-05	red
GPR63	81491	G protein-coupled receptor 63 (GPR63), mRNA.	-0.11	7.97	2.04E-13	6.20E-13	red
GTF3A	2971	general transcription factor IIIA (GTF3A), mRNA.	-0.09	7.18	4.75E-07	9.89E-07	red
H2AFY	9555	H2A histone family, member Y (H2AFY), transcript variant 1, mRNA.	-0.05	8.48	2.26E-04	3.81E-04	red
HEBP2	23593	heme binding protein 2 (HEBP2), mRNA.	-0.04	8.08	1.59E-03	2.47E-03	red
HELZ2	85441	peroxisomal proliferator-activated receptor A interacting complex 285 (PRIC285), transcript variant 2, mRNA.	-0.02	6.67	1.76E-01	2.11E-01	red
HES4	57801	hairy and enhancer of split 4 (Drosophila) (HES4), mRNA.	0.60	7.94	1.74E-77	1.21E-75	red
HNRNPA0	10949	heterogeneous nuclear ribonucleoprotein A0 (HNRPA0), mRNA.	-0.06	9.73	1.19E-03	1.87E-03	red

IDS	3423	iduronate 2-sulfatase (Hunter syndrome) (IDS), transcript variant 1, mRNA.	0.05	6.99	2.78E-03	4.18E-03	red
IFI27	3429	interferon, alpha-inducible protein 27 (IFI27), mRNA.	0.50	6.07	4.36E-47	6.23E-46	red
IFI35	3430	interferon-induced protein 35 (IFI35), mRNA.	-0.04	10.53	1.73E-04	2.95E-04	red
IFI44	10561	interferon-induced protein 44 (IFI44), mRNA.	0.05	8.53	3.81E-03	5.67E-03	red
IFI44L	10964	interferon-induced protein 44-like (IFI44L), mRNA.	0.06	10.56	6.93E-08	1.53E-07	red
IFIH1	64135	interferon induced with helicase C domain 1 (IFIH1), mRNA.	0.02	5.83	1.02E-01	1.28E-01	red
IFIT1	3434	interferon-induced protein with tetratricopeptide repeats 1 (IFIT1), transcript variant 2, mRNA.	-0.13	7.77	6.04E-12	1.69E-11	red
IFIT2	3433	interferon-induced protein with tetratricopeptide repeats 2 (IFIT2), mRNA.	0.06	9.41	4.11E-05	7.41E-05	red
IFITM1	8519	interferon induced transmembrane protein 1 (9-27) (IFITM1), mRNA.	0.01	6.73	4.78E-01	5.24E-01	red
IFITM2	10581	interferon induced transmembrane protein 2 (1-8D) (IFITM2), mRNA.	-0.30	7.96	3.69E-31	2.50E-30	red
IFITM3	10410	interferon induced transmembrane protein 3 (1-8U) (IFITM3), mRNA.	0.01	9.02	6.21E-01	6.61E-01	red
IGF2BP3	10643	insulin-like growth factor 2 mRNA binding protein 3 (IGF2BP3), mRNA.	0.01	10.12	6.74E-01	7.10E-01	red

IL13RA1	3597	interleukin 13 receptor, alpha 1 (IL13RA1), mRNA.	-0.04	8.94	1.73E-02	2.39E-02	red
IL6	3569	interleukin 6 (interferon, beta 2) (IL6), mRNA.	0.13	7.21	8.24E-15	2.70E-14	red
INPP5D	3635	inositol polyphosphate-5-phosphatase, 145kDa (INPP5D), transcript variant 1, mRNA.	-0.05	8.55	1.30E-04	2.24E-04	red
INTS12	57117	integrator complex subunit 12 (INTS12), mRNA.	-0.10	6.32	1.64E-07	3.54E-07	red
IQCG	84223	IQ motif containing G (IQCG), mRNA.	-0.01	6.71	5.19E-01	5.63E-01	red
IRF4	3662	interferon regulatory factor 4 (IRF4), mRNA.	-0.05	7.58	1.43E-03	2.23E-03	red
IRF7	3665	interferon regulatory factor 7 (IRF7), transcript variant a, mRNA.	0.02	6.85	3.93E-01	4.39E-01	red
IRF9	10379	interferon-stimulated transcription factor 3, gamma 48kDa (ISGF3G), mRNA.	-0.07	7.61	1.86E-04	3.16E-04	red
ISG15	9636	ISG15 ubiquitin-like modifier (ISG15), mRNA.	0.00	9.89	9.03E-01	9.19E-01	red
JADE2	23338	PHD finger protein 15 (PHF15), mRNA.	-0.01	8.29	3.22E-01	3.66E-01	red
KCNK12	56660	potassium channel, subfamily K, member 12 (KCNK12), mRNA.	-0.13	6.73	8.07E-11	2.12E-10	red
KIFAP3	22920	kinesin-associated protein 3 (KIFAP3), mRNA.	-0.13	8.78	1.90E-15	6.41E-15	red
KLHL14	57565	kelch-like 14 (Drosophila) (KLHL14), mRNA.	0.05	8.31	3.74E-04	6.18E-04	red
KLHL6	89857	kelch-like 6 (Drosophila) (KLHL6), mRNA.	-0.03	6.52	1.80E-02	2.47E-02	red

L1TD1	54596	LINE-1 type transposase domain containing 1 (L1TD1), mRNA.	-0.05	7.11	2.27E-02	3.09E-02	red
LAG3	3902	lymphocyte-activation gene 3 (LAG3), mRNA.	0.00	8.28	9.05E-01	9.20E-01	red
LAMP5	24141	chromosome 20 open reading frame 103 (C20orf103), mRNA.	0.09	7.82	3.04E-10	7.73E-10	red
LAP3	51056	leucine aminopeptidase 3 (LAP3), mRNA.	-0.15	6.06	2.00E-22	9.51E-22	red
LAPTM4B	55353	lysosomal associated protein transmembrane 4 beta (LAPTM4B), mRNA.	0.16	6.37	1.45E-24	7.70E-24	red
LARGE	9215	like-glycosyltransferase (LARGE), transcript variant 1, mRNA.	0.30	8.57	3.09E-46	4.23E-45	red
LARP6	55323	La ribonucleoprotein domain family, member 6 (LARP6), transcript variant 1, mRNA.	-0.19	9.32	1.51E-27	9.07E-27	red
LPAR6	10161	purinergic receptor P2Y, G-protein coupled, 5 (P2RY5), mRNA.	-0.02	8.55	1.64E-01	1.98E-01	red
LRRC8C	84230	leucine rich repeat containing 8 family, member C (LRRC8C), mRNA.	-0.04	5.69	1.14E-02	1.60E-02	red
LSAMP	4045	limbic system-associated membrane protein (LSAMP), mRNA.	-0.09	8.03	3.84E-05	6.93E-05	red
LXN	56925	latexin (LXN), mRNA.	0.10	7.64	7.60E-08	1.67E-07	red
LY6E	4061	lymphocyte antigen 6 complex, locus E (LY6E), mRNA.	0.14	6.36	2.09E-16	7.47E-16	red

MAPRE2	10982	microtubule-associated protein, RP/EB family, member 2 (MAPRE2), mRNA.	0.11	8.94	1.79E-16	6.42E-16	red
MARK1	4139	MAP/microtubule affinity-regulating kinase 1 (MARK1), mRNA.	0.03	6.52	9.09E-02	1.14E-01	red
MB21D2	151963	chromosome 3 open reading frame 59 (C3orf59), mRNA.	-0.11	8.09	2.67E-14	8.50E-14	red
MEIS2	4212	Meis homeobox 2 (MEIS2), transcript variant c, mRNA.	0.14	7.54	3.78E-16	1.33E-15	red
MGEA5	10724	meningioma expressed antigen 5 (hyaluronidase) (MGEA5), mRNA.	-0.15	9.52	6.60E-21	2.93E-20	red
MID1IP1	58526	MID1 interacting protein 1 (gastrulation specific G12 homolog (zebrafish)) (MID1IP1), mRNA.	-0.23	7.61	2.97E-36	2.54E-35	red
MLLT11	10962	myeloid/lymphoid or mixed-lineage leukemia (trithorax homolog, Drosophila); translocated to, 11 (MLLT11), mRNA.	-0.06	7.34	3.42E-03	5.11E-03	red
MNDA	4332	myeloid cell nuclear differentiation antigen (MNDA), mRNA.	0.30	8.19	2.52E-35	2.04E-34	red
MOCOS	55034	molybdenum cofactor sulfurase (MOCOS), mRNA.	-0.24	6.23	4.77E-31	3.21E-30	red

MRPL44	65080	mitochondrial ribosomal protein L44 (MRPL44), nuclear gene encoding mitochondrial protein, mRNA.	-0.20	7.69	1.70E-33	1.27E-32	red
MSI2	124540	musashi homolog 2 (Drosophila) (MSI2), transcript variant 1, mRNA.	0.07	9.39	2.43E-10	6.25E-10	red
MX1	4599	myxovirus (influenza virus) resistance 1, interferon-inducible protein p78 (mouse) (MX1), mRNA.	0.36	8.49	9.91E-46	1.32E-44	red
MYB	4602	v-myb myeloblastosis viral oncogene homolog (avian) (MYB), mRNA.	0.12	7.46	7.73E-11	2.04E-10	red
MYO5A	4644	myosin VA (heavy chain 12, myoxin) (MYO5A), mRNA.	-0.16	8.22	2.75E-15	9.21E-15	red
NA	648	BMI1 polycomb ring finger oncogene (BMI1), mRNA.	0.13	8.28	2.52E-14	8.05E-14	red
NAGK	55577	N-acetylglucosamine kinase (NAGK), mRNA.	0.10	7.80	2.05E-09	4.98E-09	red
NCOA1	8648	nuclear receptor coactivator 1 (NCOA1), transcript variant 1, mRNA.	-0.22	11.40	3.55E-36	3.01E-35	red
NDUFAF6	137682	chromosome 8 open reading frame 38 (C8orf38), mRNA.	-0.09	7.85	5.62E-07	1.16E-06	red
NEIL2	252969	nei like 2 (E. coli) (NEIL2), mRNA.	0.11	7.44	1.01E-09	2.48E-09	red
NEUROG2	63973	neurogenin 2 (NEUROG2), mRNA.	0.17	7.56	1.80E-18	7.12E-18	red

NFAT5	10725	nuclear factor of activated T-cells 5, tonicity-responsive (NFAT5), transcript variant 5, mRNA.	-0.03	6.88	3.77E-01	4.22E-01	red
NGFRAP1	27018	nerve growth factor receptor (TNFRSF16) associated protein 1 (NGFRAP1), transcript variant 3, mRNA.	-0.06	9.45	1.21E-06	2.45E-06	red
NKIRAS1	28512	NFKB inhibitor interacting Ras-like 1 (NKIRAS1), mRNA.	0.36	6.20	1.83E-42	2.09E-41	red
NME7	29922	non-metastatic cells 7, protein expressed in (nucleoside-diphosphate kinase) (NME7), transcript variant 1, mRNA.	-0.08	8.45	2.12E-09	5.14E-09	red
NSF	4905	PREDICTED: N-ethylmaleimide-sensitive factor (NSF), mRNA.	-0.16	7.15	1.68E-24	8.88E-24	red
NSG1	27065	DNA segment on chromosome 4 (unique) 234 expressed sequence (D4S234E), mRNA.	0.03	11.29	3.22E-02	4.30E-02	red
NT5C3A	51251	5'-nucleotidase, cytosolic III (NT5C3), transcript variant 2, mRNA.	-0.04	9.42	2.24E-02	3.06E-02	red
NT5DC1	221294	5'-nucleotidase domain containing 1 (NT5DC1), mRNA.	-0.16	8.65	2.42E-24	1.27E-23	red
NT5E	4907	5'-nucleotidase, ecto (CD73) (NT5E), mRNA.	0.12	8.52	1.14E-15	3.91E-15	red
OAS1	4938	2',5'-oligoadenylate synthetase 1, 40/46kDa (OAS1), transcript variant 2, mRNA.	0.47	9.38	9.82E-56	2.04E-54	red

OXCT2	64064	3-oxoacid CoA transferase 2 (OXCT2), mRNA.	0.94	6.94	5.85E-76	3.66E-74	red
OXR1	55074	oxidation resistance 1 (OXR1), mRNA.	-0.06	8.28	2.03E-05	3.75E-05	red
P4HA2	8974	procollagen-proline, 2-oxoglutarate 4-dioxygenase (proline 4-hydroxylase), alpha polypeptide II (P4HA2), transcript variant 1, mRNA.	0.01	8.73	3.37E-01	3.82E-01	red
PABPC4	400455	PREDICTED: hypothetical LOC400455 (LOC400455), mRNA.	-0.05	6.71	1.53E-03	2.37E-03	red
PAK2	5062	p21 (CDKN1A)-activated kinase 2 (PAK2), mRNA.	-0.22	8.09	6.20E-28	3.78E-27	red
PARP12	64761	poly (ADP-ribose) polymerase family, member 12 (PARP12), mRNA.	0.11	6.88	6.07E-09	1.44E-08	red
PARP14	54625	poly (ADP-ribose) polymerase family, member 14 (PARP14), mRNA.	0.11	7.94	5.69E-18	2.21E-17	red
PARP9	83666	poly (ADP-ribose) polymerase family, member 9 (PARP9), mRNA.	0.06	5.76	2.47E-06	4.91E-06	red
PBX3	5090	pre-B-cell leukemia homeobox 3 (PBX3), mRNA.	0.00	6.71	7.82E-01	8.10E-01	red
PCED1B	91523	family with sequence similarity 113, member B (FAM113B), mRNA.	0.10	6.69	1.10E-10	2.87E-10	red

PDE4B	5142	phosphodiesterase 4B, cAMP-specific (phosphodiesterase E4 dunce homolog, Drosophila) (PDE4B), transcript variant b, mRNA.	0.02	7.39	1.24E-01	1.53E-01	red
PDE6B	5158	phosphodiesterase 6B, cGMP-specific, rod, beta (congenital stationary night blindness 3, autosomal dominant) (PDE6B), mRNA.	-0.12	9.28	2.96E-15	9.90E-15	red
PDK3	5165	pyruvate dehydrogenase kinase, isozyme 3 (PDK3), mRNA.	0.03	6.52	5.90E-02	7.62E-02	red
PDPR	55066	pyruvate dehydrogenase phosphatase regulatory subunit (PDPR), mRNA.	-0.22	9.56	2.28E-38	2.15E-37	red
PELO	53918	pelota homolog (Drosophila) (PELO), mRNA.	-0.03	6.97	2.59E-02	3.50E-02	red
PERP	64065	PERP, TP53 apoptosis effector (PERP), mRNA.	0.08	8.31	2.46E-07	5.22E-07	red
PHF10	55274	PHD finger protein 10 (PHF10), transcript variant 2, mRNA.	0.07	7.54	1.09E-03	1.71E-03	red
PHF11	51131	PHD finger protein 11 (PHF11), transcript variant 2, mRNA.	-0.03	8.27	6.13E-02	7.90E-02	red
PIAS2	9063	protein inhibitor of activated STAT, 2 (PIAS2), transcript variant alpha, mRNA.	0.13	8.44	1.43E-16	5.15E-16	red

PIP5K1B	8395	phosphatidylinositol-4-phosphate 5-kinase, type I, beta (PIP5K1B), transcript variant 2, mRNA.	-0.07	10.62	7.35E-06	1.41E-05	red
PKP2	5318	plakophilin 2 (PKP2), transcript variant 2b, mRNA.	-0.02	10.02	9.96E-02	1.25E-01	red
PLA2G16	11145	HRAS-like suppressor 3 (HRASLS3), mRNA.	-0.22	7.53	2.08E-37	1.87E-36	red
PLAGL2	5326	pleiomorphic adenoma gene-like 2 (PLAGL2), mRNA.	-0.03	7.44	1.68E-01	2.02E-01	red
PLEKHF2	79666	pleckstrin homology domain containing, family F (with FYVE domain) member 2 (PLEKHF2), mRNA.	-0.01	10.48	2.46E-01	2.86E-01	red
PLSCR1	5359	phospholipid scramblase 1 (PLSCR1), mRNA.	0.03	10.81	1.11E-03	1.75E-03	red
PLTP	5360	phospholipid transfer protein (PLTP), transcript variant 2, mRNA.	0.03	8.99	3.85E-02	5.10E-02	red
PMM2	5373	phosphomannomutase 2 (PMM2), mRNA.	-0.03	6.40	1.49E-01	1.81E-01	red
POLE3	54107	polymerase (DNA directed), epsilon 3 (p17 subunit) (POLE3), mRNA.	0.02	8.72	1.45E-01	1.76E-01	red
POP1	10940	processing of precursor 1, ribonuclease P/MRP subunit (S. cerevisiae) (POP1), mRNA.	0.05	7.09	1.67E-02	2.31E-02	red

PPP1R11	6992	protein phosphatase 1, regulatory (inhibitor) subunit 11 (PPP1R11), mRNA.	0.08	11.00	3.39E-04	5.63E-04	red
PPP1R16B	26051	protein phosphatase 1, regulatory (inhibitor) subunit 16B (PPP1R16B), mRNA.	-0.08	8.48	4.30E-07	8.97E-07	red
PRADC1	84279	chromosome 2 open reading frame 7 (C2orf7), mRNA.	-0.11	11.45	3.44E-16	1.21E-15	red
PRICKLE1	144165	prickle homolog 1 (Drosophila) (PRICKLE1), mRNA.	-0.07	8.18	6.91E-05	1.22E-04	red
RAB13	5872	RAB13, member RAS oncogene family (RAB13), mRNA.	-0.02	8.12	1.86E-01	2.22E-01	red
RALGPS2	55103	Ral GEF with PH domain and SH3 binding motif 2 (RALGPS2), transcript variant 2, mRNA.	-0.11	8.38	3.83E-11	1.02E-10	red
RAP1GDS1	5910	RAP1, GTP-GDP dissociation stimulator 1 (RAP1GDS1), mRNA.	-0.01	11.56	6.32E-01	6.71E-01	red
RARRES3	5920	retinoic acid receptor responder (tazarotene induced) 3 (RARRES3), mRNA.	-0.09	6.93	5.78E-14	1.82E-13	red
RASA1	5921	RAS p21 protein activator (GTPase activating protein) 1 (RASA1), transcript variant 1, mRNA.	-0.08	7.48	1.80E-07	3.87E-07	red

RBCK1	10616	RanBP-type and C3HC4-type zinc finger containing 1 (RBCK1), transcript variant 2, mRNA.	-0.06	5.70	7.84E-04	1.26E-03	red
RBM18	92400	RNA binding motif protein 18 (RBM18), mRNA.	-0.01	6.29	5.17E-01	5.62E-01	red
RCN2	5955	reticulocalbin 2, EF-hand calcium binding domain (RCN2), mRNA.	-0.46	5.92	3.83E-46	5.21E-45	red
RRS1	23212	RRS1 ribosome biogenesis regulator homolog (S. cerevisiae) (RRS1), mRNA.	-0.03	6.27	1.52E-02	2.12E-02	red
RTN4IP1	84816	reticulon 4 interacting protein 1 (RTN4IP1), nuclear gene encoding mitochondrial protein, mRNA.	0.06	7.50	3.05E-03	4.57E-03	red
SALL2	6297	sal-like 2 (Drosophila) (SALL2), mRNA.	-0.06	6.10	5.88E-05	1.05E-04	red
SAMD9L	219285	sterile alpha motif domain containing 9-like (SAMD9L), mRNA.	0.05	8.93	3.84E-04	6.35E-04	red
SAT1	6303	spermidine/spermine N1-acetyltransferase 1 (SAT1), mRNA.	-0.04	8.09	1.21E-03	1.90E-03	red
SEC22B	9554	SEC22 vesicle trafficking protein homolog B (S. cerevisiae) (SEC22B), mRNA.	-0.03	5.61	1.32E-01	1.62E-01	red
SIDT1	54847	SID1 transmembrane family, member 1 (SIDT1), mRNA.	0.02	5.70	3.16E-01	3.60E-01	red

SIRPA	140885	signal-regulatory protein alpha (SIRPA), transcript variant 1, mRNA.	0.05	8.00	2.58E-03	3.90E-03	red
SLC2A3	6515	solute carrier family 2 (facilitated glucose transporter), member 3 (SLC2A3), mRNA.	0.02	10.87	5.28E-02	6.87E-02	red
SLC9A3R1	9368	solute carrier family 9 (sodium/hydrogen exchanger), member 3 regulator 1 (SLC9A3R1), mRNA.	-0.17	6.76	3.72E-24	1.94E-23	red
SMAP1	60682	stromal membrane-associated protein 1 (SMAP1), transcript variant 1, mRNA.	-0.06	7.71	4.66E-03	6.87E-03	red
SMARCA2	6595	SWI/SNF related, matrix associated, actin dependent regulator of chromatin, subfamily a, member 2 (SMARCA2), transcript variant 2, mRNA.	-0.02	8.23	2.45E-01	2.86E-01	red
SP110	3431	SP110 nuclear body protein (SP110), transcript variant b, mRNA.	0.24	7.38	1.31E-31	9.10E-31	red
SPA17	53340	sperm autoantigenic protein 17 (SPA17), mRNA.	-0.22	7.75	1.94E-26	1.11E-25	red
SPAG6	9576	sperm associated antigen 6 (SPAG6), transcript variant 1, mRNA.	-0.03	6.71	3.16E-02	4.22E-02	red
SPIRE1	56907	spire homolog 1 (Drosophila) (SPIRE1), mRNA.	0.04	10.44	2.56E-03	3.88E-03	red

ST3GAL6	10402	ST3 beta-galactoside alpha-2,3-sialyltransferase 6 (ST3GAL6), mRNA.	-0.02	8.18	1.06E-01	1.31E-01	red
STAP1	26228	BCR downstream signaling 1 (BRDG1), mRNA.	-0.29	6.72	1.60E-37	1.45E-36	red
STAT1	6772	signal transducer and activator of transcription 1, 91kDa (STAT1), transcript variant beta, mRNA.	0.20	5.98	5.85E-19	2.38E-18	red
STAT2	6773	signal transducer and activator of transcription 2, 113kDa (STAT2), mRNA.	-0.16	8.49	1.42E-18	5.65E-18	red
STK4	6789	serine/threonine kinase 4 (STK4), mRNA.	-0.10	6.73	4.36E-11	1.16E-10	red
STRADB	55437	amyotrophic lateral sclerosis 2 (juvenile) chromosome region, candidate 2 (ALS2CR2), mRNA.	-0.12	6.08	4.22E-14	1.33E-13	red
SUSD1	64420	sushi domain containing 1 (SUSD1), mRNA.	-0.08	7.43	2.25E-11	6.11E-11	red
SYBU	55638	hypothetical protein FLJ20366 (FLJ20366), mRNA.	-0.07	7.83	3.74E-05	6.77E-05	red
SYNM	23336	desmuslin (DMN), transcript variant B, mRNA.	0.13	6.77	8.32E-11	2.19E-10	red
TEAD4	7004	TEA domain family member 4 (TEAD4), transcript variant 2, mRNA.	-0.11	10.04	1.37E-12	3.98E-12	red
THAP10	56906	THAP domain containing 10 (THAP10), mRNA.	0.02	10.70	1.48E-02	2.06E-02	red

TLR7	51284	toll-like receptor 7 (TLR7), mRNA.	0.23	6.52	7.40E-27	4.31E-26	red
TM2D3	80213	TM2 domain containing 3 (TM2D3), transcript variant 2, mRNA.	-0.05	8.18	5.18E-03	7.60E-03	red
TM9SF2	9375	transmembrane 9 superfamily member 2 (TM9SF2), mRNA.	-0.02	7.44	1.58E-01	1.91E-01	red
TMEM140	55281	transmembrane protein 140 (TMEM140), mRNA.	-0.07	7.76	1.59E-05	2.97E-05	red
TMEM237	65062	amyotrophic lateral sclerosis 2 (juvenile) chromosome region, candidate 4 (ALS2CR4), transcript variant 2, mRNA.	0.01	7.41	4.71E-01	5.17E-01	red
TMEM8B	51754	chromosome 9 open reading frame 127 (C9orf127), transcript variant 3, mRNA.	0.18	7.96	5.11E-26	2.88E-25	red
TNFSF10	8743	tumor necrosis factor (ligand) superfamily, member 10 (TNFSF10), mRNA.	-0.01	7.17	4.85E-01	5.30E-01	red
TP53INP1	94241	tumor protein p53 inducible nuclear protein 1 (TP53INP1), mRNA.	0.11	6.00	4.60E-10	1.16E-09	red
TRA2B	6434	splicing factor, arginine/serine-rich 10 (transformer 2 homolog, Drosophila) (SFRS10), mRNA.	0.06	5.93	9.65E-05	1.68E-04	red
TRAM2	9697	translocation associated membrane protein 2 (TRAM2), mRNA.	0.01	7.87	1.99E-01	2.37E-01	red

TRERF1	55809	transcriptional regulating factor 1 (TRERF1), mRNA.	-0.02	7.17	2.56E-01	2.98E-01	red
TRIM69	140691	tripartite motif-containing 69 (TRIM69), transcript variant a, mRNA.	-0.03	6.49	5.05E-02	6.59E-02	red
TST	7263	thiosulfate sulfurtransferase (rhodanese) (TST), nuclear gene encoding mitochondrial protein, mRNA.	-0.14	6.99	4.56E-18	1.78E-17	red
TUBB2A	Na	tubulin, beta polypeptide (TUBB), mRNA.	2.90	9.15	8.82E-140	4.48E-136	red
TUBB2B	347733	tubulin, beta 2B (TUBB2B), mRNA.	0.17	9.67	3.52E-24	1.84E-23	red
UBE2E1	7324	ubiquitin-conjugating enzyme E2E 1 (UBC4/5 homolog, yeast) (UBE2E1), transcript variant 2, mRNA.	-0.01	7.81	6.53E-01	6.91E-01	red
UBE2F	140739	ubiquitin-conjugating enzyme E2F (putative) (UBE2F), mRNA.	0.03	8.16	2.33E-02	3.17E-02	red
UCHL1	7345	ubiquitin carboxyl-terminal esterase L1 (ubiquitin thiolesterase) (UCHL1), mRNA.	0.04	8.47	2.39E-03	3.63E-03	red
UFSP2	55325	chromosome 4 open reading frame 20 (C4orf20), mRNA.	0.04	9.65	1.97E-03	3.02E-03	red
UHRF1	29128	ubiquitin-like, containing PHD and RING finger domains, 1 (UHRF1), transcript variant 1, mRNA.	0.02	7.51	1.58E-01	1.92E-01	red

VKORC1L1	154807	vitamin K epoxide reductase complex, subunit 1-like 1 (VKORC1L1), mRNA.	-0.01	7.09	5.36E-01	5.81E-01	red
VPREB3	29802	pre-B lymphocyte gene 3 (VPREB3), mRNA.	-0.18	7.30	1.41E-14	4.59E-14	red
WBP5	51186	WW domain binding protein 5 (WBP5), transcript variant 4, mRNA.	-0.17	9.20	4.94E-25	2.68E-24	red
WDFY1	57590	WD repeat and FYVE domain containing 1 (WDFY1), mRNA.	-0.26	7.21	1.09E-27	6.59E-27	red
WNT10A	80326	wingless-type MMTV integration site family, member 10A (WNT10A), mRNA.	0.59	6.99	3.11E-55	6.29E-54	red
XAF1	54739	XIAP associated factor-1 (XAF1), transcript variant 1, mRNA.	-0.06	7.24	9.43E-05	1.65E-04	red
XPNPEP1	7511	X-prolyl aminopeptidase (aminopeptidase P) 1, soluble (XPNPEP1), mRNA.	-0.27	7.42	3.42E-36	2.91E-35	red
ZBTB32	27033	zinc finger and BTB domain containing 32 (ZBTB32), mRNA.	-0.07	7.05	4.42E-06	8.62E-06	red
ZC2HC1A	51101	chromosome 8 open reading frame 70 (C8orf70), mRNA.	-0.02	5.46	2.16E-01	2.55E-01	red
ZNF22	7570	zinc finger protein 22 (KOX 15) (ZNF22), mRNA.	0.02	8.87	4.06E-01	4.51E-01	red
ZNF362	149076	FLJ25476 protein (FLJ25476), mRNA.	-0.18	8.27	8.20E-21	3.63E-20	red
ZYX	7791	zyxin (ZYX), transcript variant 2, mRNA.	-0.12	8.91	7.35E-14	2.29E-13	red

ACTL6A	86	actin-like 6A (ACTL6A), transcript variant 3, mRNA.	0.06	6.63	5.10E-04	8.33E-04	salmon
ADNP2	22850	zinc finger protein 508 (ZNF508), mRNA.	0.02	7.59	8.37E-02	1.06E-01	salmon
AKIP1	56672	chromosome 11 open reading frame 17 (C11orf17), transcript variant 1, mRNA.	0.04	8.73	1.15E-03	1.80E-03	salmon
ANKRD13C	81573	ankyrin repeat domain 13C (ANKRD13C), mRNA.	0.16	8.83	1.22E-20	5.38E-20	salmon
ANKRD46	157567	ankyrin repeat domain 46 (ANKRD46), mRNA.	0.08	9.17	9.19E-11	2.41E-10	salmon
AP3M1	26985	adaptor-related protein complex 3, mu 1 subunit (AP3M1), transcript variant 1, mRNA.	-0.26	5.88	4.68E-38	4.38E-37	salmon
APOBEC3H	164668	apolipoprotein B mRNA editing enzyme, catalytic polypeptide-like 3H (APOBEC3H), mRNA.	-0.29	7.66	1.11E-41	1.20E-40	salmon
ARL6IP1	23204	ADP-ribosylation factor-like 6 interacting protein 1 (ARL6IP1), mRNA.	0.11	7.25	9.72E-12	2.70E-11	salmon
ASF1B	55723	ASF1 anti-silencing function 1 homolog B (S. cerevisiae) (ASF1B), mRNA.	-0.16	6.41	2.19E-16	7.80E-16	salmon
ATAD1	84896	ATPase family, AAA domain containing 1 (ATAD1), mRNA.	0.88	7.93	2.30E-71	1.11E-69	salmon

BAZ1A	11177	bromodomain adjacent to zinc finger domain, 1A (BAZ1A), transcript variant 2, mRNA.	-0.06	6.14	8.56E-05	1.50E-04	salmon
CARHSP1	23589	calcium regulated heat stable protein 1, 24kDa (CARHSP1), transcript variant 2, mRNA.	0.00	9.01	7.62E-01	7.92E-01	salmon
CBX3	11335	chromobox homolog 3 (HP1 gamma homolog, Drosophila) (CBX3), transcript variant 2, mRNA.	-0.02	8.37	2.28E-01	2.68E-01	salmon
CCDC69	26112	coiled-coil domain containing 69 (CCDC69), mRNA.	-0.38	7.87	3.27E-57	7.52E-56	salmon
CCT8	10694	chaperonin containing TCP1, subunit 8 (theta) (CCT8), mRNA.	-0.10	8.06	4.26E-09	1.02E-08	salmon
CD63	967	CD63 antigen (melanoma 1 antigen) (CD63), mRNA.	0.05	6.63	1.22E-03	1.92E-03	salmon
CDK9	1025	cyclin-dependent kinase 9 (CDC2-related kinase) (CDK9), mRNA.	-0.06	7.91	1.40E-06	2.83E-06	salmon
CENPK	64105	centromere protein K (CENPK), mRNA.	-0.08	8.23	1.87E-05	3.48E-05	salmon
CEPT1	10390	choline/ethanolamine phosphotransferase 1 (CEPT1), transcript variant 1, mRNA.	-0.17	8.60	1.28E-27	7.72E-27	salmon
CES2	8824	carboxylesterase 2 (intestine, liver) (CES2), transcript variant 2, mRNA.	-0.13	8.94	8.70E-16	3.01E-15	salmon

CHCHD6	84303	coiled-coil-helix-coiled-coil-helix domain containing 6 (CHCHD6), mRNA.	-0.02	8.51	1.41E-01	1.72E-01	salmon
CHORDC1	26973	cysteine and histidine-rich domain (CHORD)-containing 1 (CHORDC1), mRNA.	0.02	8.09	5.63E-02	7.29E-02	salmon
CHST2	9435	carbohydrate (N-acetylglucosamine-6-O) sulfotransferase 2 (CHST2), mRNA.	0.02	6.00	2.94E-01	3.38E-01	salmon
CLK1	1195	CDC-like kinase 1 (CLK1), transcript variant 1, mRNA.	0.14	9.86	3.93E-25	2.14E-24	salmon
CNBP	7555	CCHC-type zinc finger, nucleic acid binding protein (CNBP), mRNA.	-0.01	7.73	5.96E-01	6.38E-01	salmon
CNOT7	29883	CCR4-NOT transcription complex, subunit 7 (CNOT7), transcript variant 1, mRNA.	0.15	8.48	1.24E-09	3.05E-09	salmon
COMMD3	23412	COMM domain containing 3 (COMMD3), mRNA.	-0.16	8.35	7.65E-19	3.09E-18	salmon
CRAT	1384	carnitine acetyltransferase (CRAT), nuclear gene encoding mitochondrial protein, transcript variant 1, mRNA.	-0.07	9.58	8.79E-07	1.80E-06	salmon
CREBBP	1387	CREB binding protein (Rubinstein-Taybi syndrome) (CREBBP), transcript variant 2, mRNA.	-0.01	6.01	7.69E-01	7.98E-01	salmon
CRIP1	1396	cysteine-rich protein 1 (intestinal) (CRIP1), mRNA.	-0.06	5.49	8.74E-05	1.53E-04	salmon

CTPS1	1503	CTP synthase (CTPS), mRNA.	0.02	9.47	1.25E-01	1.54E-01	salmon
CUEDC2	79004	CUE domain containing 2 (CUEDC2), mRNA.	0.04	7.18	3.59E-02	4.77E-02	salmon
CUTA	51596	cutA divalent cation tolerance homolog (E. coli) (CUTA), transcript variant 3, mRNA.	0.13	10.43	9.53E-11	2.49E-10	salmon
DDX21	9188	DEAD (Asp-Glu-Ala-Asp) box polypeptide 21 (DDX21), mRNA.	0.06	5.63	1.01E-05	1.92E-05	salmon
DDX3X	1654	DEAD (Asp-Glu-Ala-Asp) box polypeptide 3, X-linked (DDX3X), mRNA.	-0.02	10.06	3.03E-01	3.47E-01	salmon
DEK	7913	DEK oncogene (DNA binding) (DEK), mRNA.	-0.28	6.80	1.14E-44	1.43E-43	salmon
DEXI	28955	dexamethasone-induced transcript (DEXI), mRNA.	0.05	8.19	3.59E-05	6.51E-05	salmon
DKC1	1736	dyskeratosis congenita 1, dyskerin (DKC1), mRNA.	0.24	6.32	9.09E-34	6.86E-33	salmon
DUSP28	285193	dual specificity phosphatase 28 (DUSP28), mRNA.	-0.06	7.86	7.81E-05	1.38E-04	salmon
EDARADD	128178	EDAR-associated death domain (EDARADD), transcript variant B, mRNA.	-0.06	6.32	5.83E-03	8.50E-03	salmon
EDRF1	26098	chromosome 10 open reading frame 137 (C10orf137), mRNA.	-0.02	11.04	2.45E-01	2.86E-01	salmon
EIF4ENIF1	56478	eukaryotic translation initiation factor 4E nuclear import factor 1 (EIF4ENIF1), mRNA.	-0.01	8.20	3.15E-01	3.59E-01	salmon

ELMSAN1	91748	chromosome 14 open reading frame 43 (C14orf43), transcript variant 2, mRNA.	0.03	7.85	7.53E-03	1.09E-02	salmon
F8A1	8263	coagulation factor VIII-associated (intronic transcript) 1 (F8A1), mRNA.	-0.01	8.12	2.22E-01	2.62E-01	salmon
FAM3A	60343	family with sequence similarity 3, member A (FAM3A), mRNA.	0.01	11.07	7.16E-01	7.49E-01	salmon
FASTKD1	79675	FAST kinase domains 1 (FASTKD1), mRNA.	-0.07	7.01	7.88E-04	1.26E-03	salmon
FOXRED1	55572	FAD-dependent oxidoreductase domain containing 1 (FOXRED1), mRNA.	-0.04	9.25	6.72E-03	9.77E-03	salmon
FXYD5	53827	FXYD domain containing ion transport regulator 5 (FXYD5), transcript variant 1, mRNA.	-0.13	8.24	6.32E-13	1.87E-12	salmon
GAMT	2593	guanidinoacetate N-methyltransferase (GAMT), transcript variant 1, mRNA.	0.05	9.24	2.06E-03	3.16E-03	salmon
GBE1	2632	glucan (1,4-alpha-), branching enzyme 1 (glycogen branching enzyme, Andersen disease, glycogen storage disease type IV) (GBE1), mRNA.	0.01	8.81	2.18E-01	2.57E-01	salmon
GFPT1	2673	glutamine-fructose-6-phosphate transaminase 1 (GFPT1), mRNA.	0.16	9.10	4.08E-16	1.43E-15	salmon
GLG1	2734	golgi apparatus protein 1 (GLG1), mRNA.	0.03	8.39	3.05E-02	4.08E-02	salmon

GMFB	2764	glia maturation factor, beta (GMFB), mRNA.	-0.05	6.39	1.11E-03	1.75E-03	salmon
GSKIP	51527	chromosome 14 open reading frame 129 (C14orf129), mRNA.	-0.10	8.49	1.43E-10	3.70E-10	salmon
HAGH	3029	hydroxyacylglutathione hydrolase (HAGH), transcript variant 2, mRNA.	0.04	5.61	3.30E-02	4.40E-02	salmon
HCST	10870	hematopoietic cell signal transducer (HCST), transcript variant 2, mRNA.	0.17	8.53	1.20E-14	3.90E-14	salmon
HIATL1	84641	hippocampus abundant transcript-like 1 (HIATL1), mRNA.	-0.06	8.92	2.37E-05	4.38E-05	salmon
HMGCL	3155	3-hydroxymethyl-3-methylglutaryl-Coenzyme A lyase (hydroxymethylglutaricaciduria) (HMGCL), mRNA.	-0.14	8.26	5.09E-20	2.18E-19	salmon
HRASLS2	54979	HRAS-like suppressor 2 (HRASLS2), mRNA.	0.01	10.30	7.44E-01	7.74E-01	salmon
HSP90AA1	3320	heat shock protein 90kDa alpha (cytosolic), class A member 1 (HSP90AA1), transcript variant 2, mRNA.	-0.15	7.96	1.90E-18	7.54E-18	salmon
HYLS1	219844	hydrolethalus syndrome 1 (HYLS1), mRNA.	-0.05	8.89	3.03E-04	5.05E-04	salmon
ICAM3	3385	intercellular adhesion molecule 3 (ICAM3), mRNA.	-0.08	6.21	3.02E-08	6.81E-08	salmon
KCTD10	83892	potassium channel tetramerisation domain containing 10 (KCTD10), mRNA.	-0.11	5.47	7.88E-09	1.85E-08	salmon

KDM6A	7403	ubiquitously transcribed tetratricopeptide repeat, X chromosome (UTX), mRNA.	1.36	6.03	1.20E-71	5.85E-70	salmon
KLHDC3	116138	kelch domain containing 3 (KLHDC3), mRNA.	-0.10	6.08	2.11E-06	4.21E-06	salmon
KLHDC4	54758	kelch domain containing 4 (KLHDC4), mRNA.	0.07	6.78	3.71E-04	6.14E-04	salmon
LAMP2	3920	lysosomal-associated membrane protein 2 (LAMP2), transcript variant LAMP2B, mRNA.	-0.05	8.16	9.53E-04	1.51E-03	salmon
LARP7	51574	La ribonucleoprotein domain family, member 7 (LARP7), transcript variant 1, mRNA.	0.05	5.95	1.91E-03	2.93E-03	salmon
LCMT1	51451	leucine carboxyl methyltransferase 1 (LCMT1), transcript variant 1, mRNA.	-0.11	7.21	8.72E-11	2.29E-10	salmon
LGALS1	3956	lectin, galactoside-binding, soluble, 1 (galectin 1) (LGALS1), mRNA.	-0.08	9.45	7.79E-08	1.71E-07	salmon
LINC00467	84791	chromosome 1 open reading frame 97 (C1orf97), mRNA.	0.14	8.35	2.78E-10	7.10E-10	salmon
MAPK3	5595	mitogen-activated protein kinase 3 (MAPK3), transcript variant 1, mRNA.	0.00	12.06	8.81E-01	8.99E-01	salmon

MARS2	92935	methionyl-tRNA synthetase 2, mitochondrial (MARS2), nuclear gene encoding mitochondrial protein, mRNA.	-0.22	7.39	1.52E-25	8.44E-25	salmon
MCEE	84693	methylmalonyl CoA epimerase (MCEE), mRNA.	0.09	7.22	9.91E-06	1.89E-05	salmon
MRPS27	23107	mitochondrial ribosomal protein S27 (MRPS27), nuclear gene encoding mitochondrial protein, mRNA.	0.10	6.67	4.75E-11	1.26E-10	salmon
MTERF3	51001	MTERF domain containing 1 (MTERFD1), mRNA.	0.02	6.52	1.18E-01	1.46E-01	salmon
MYADM	91663	myeloid-associated differentiation marker (MYADM), transcript variant 4, mRNA.	0.60	6.00	8.73E-55	1.71E-53	salmon
MYL6B	140465	myosin, light chain 6B, alkali, smooth muscle and non-muscle (MYL6B), mRNA.	0.11	7.17	2.83E-10	7.22E-10	salmon
NA	445329	sulfotransferase family, cytosolic, 1A, phenol-preferring, member 4 (SULT1A4), transcript variant 1, mRNA.	-0.25	6.99	2.12E-34	1.65E-33	salmon
NA	79897	ribonuclease P 21kDa subunit (RPP21), mRNA.	0.11	6.73	4.24E-09	1.01E-08	salmon
NA	117584	ring finger and FYVE-like domain containing 1 (RFFL), transcript variant 2, mRNA.	0.03	9.29	1.32E-03	2.07E-03	salmon

NACC2	138151	BTB (POZ) domain containing 14A (BTBD14A), mRNA.	0.00	5.75	7.68E-01	7.97E-01	salmon
NAPRT	93100	nicotinate phosphoribosyltransferase domain containing 1 (NAPRT1), mRNA.	-0.11	5.91	3.41E-11	9.17E-11	salmon
NDUFB10	4716	NADH dehydrogenase (ubiquinone) 1 beta subcomplex, 10, 22kDa (NDUFB10), mRNA.	0.00	9.16	8.57E-01	8.78E-01	salmon
NIFK	84365	MKI67 (FHA domain) interacting nucleolar phosphoprotein (MKI67IP), mRNA.	0.02	8.32	1.60E-01	1.94E-01	salmon
NINJ2	4815	ninjurin 2 (NINJ2), mRNA.	0.06	7.26	2.95E-06	5.82E-06	salmon
NMRK1	54981	chromosome 9 open reading frame 95 (C9orf95), mRNA.	-0.17	10.71	1.22E-29	7.79E-29	salmon
NOP56	10528	nucleolar protein 5A (56kDa with KKE/D repeat) (NOL5A), mRNA.	-0.02	10.29	1.77E-01	2.12E-01	salmon
NOP58	51602	nucleolar protein NOP5/NOP58 (NOP5/NOP58), mRNA.	0.09	7.94	4.03E-04	6.65E-04	salmon
NPRL2	10641	tumor suppressor candidate 4 (TUSC4), mRNA.	0.12	8.94	2.27E-18	8.93E-18	salmon
NSMCE1	197370	non-SMC element 1 homolog (S. cerevisiae) (NSMCE1), mRNA.	0.15	6.85	1.96E-15	6.61E-15	salmon
NUDT1	4521	nudix (nucleoside diphosphate linked moiety X)-type motif 1 (NUDT1), transcript variant 3B, mRNA.	0.06	10.69	1.13E-05	2.15E-05	salmon

NUDT18	79873	nudix (nucleoside diphosphate linked moiety X)-type motif 18 (NUDT18), mRNA.	-0.02	7.70	1.82E-01	2.18E-01	salmon
NUP107	57122	nucleoporin 107kDa (NUP107), mRNA.	0.00	6.23	9.98E-01	9.98E-01	salmon
NUP153	9972	nucleoporin 153kDa (NUP153), mRNA.	0.12	5.53	1.90E-12	5.46E-12	salmon
NUP205	23165	nucleoporin 205kDa (NUP205), mRNA.	-0.24	10.01	1.48E-30	9.76E-30	salmon
ODC1	4953	ornithine decarboxylase 1 (ODC1), mRNA.	0.07	6.24	4.10E-04	6.76E-04	salmon
OMA1	115209	OMA1 homolog, zinc metallopeptidase (S. cerevisiae) (OMA1), mRNA.	0.27	6.34	5.11E-35	4.09E-34	salmon
OTUD4	54726	OTU domain containing 4 (OTUD4), transcript variant 1, mRNA.	-0.08	9.52	3.87E-08	8.65E-08	salmon
PAPD4	167153	PAP associated domain containing 4 (PAPD4), mRNA.	-0.22	5.99	1.22E-23	6.20E-23	salmon
PGRMC2	10424	progesterone receptor membrane component 2 (PGRMC2), mRNA.	0.14	5.77	6.68E-11	1.77E-10	salmon
PIGN	23556	phosphatidylinositol glycan anchor biosynthesis, class N (PIGN), transcript variant 2, mRNA.	-0.15	7.00	1.94E-18	7.66E-18	salmon
PLA2G4A	5321	phospholipase A2, group IVA (cytosolic, calcium-dependent) (PLA2G4A), mRNA.	0.04	8.22	8.99E-03	1.29E-02	salmon

PNISR	25957	chromosome 6 open reading frame 111 (C6orf111), mRNA.	-0.04	7.04	8.43E-05	1.48E-04	salmon
PNN	5411	pinin, desmosome associated protein (PNN), mRNA.	-0.08	6.23	2.68E-08	6.06E-08	salmon
POLR2J	5439	polymerase (RNA) II (DNA directed) polypeptide J, 13.3kDa (POLR2J), mRNA.	-0.75	6.97	4.18E-58	1.01E-56	salmon
POLR3B	55703	polymerase (RNA) III (DNA directed) polypeptide B (POLR3B), mRNA.	-0.03	11.88	1.46E-02	2.04E-02	salmon
POLR3GL	84265	polymerase (RNA) III (DNA directed) polypeptide G (32kD)-like (POLR3GL), mRNA.	-0.04	8.66	4.60E-03	6.79E-03	salmon
PPIG	9360	peptidylprolyl isomerase G (cyclophilin G) (PPIG), mRNA.	0.01	11.83	6.79E-01	7.14E-01	salmon
PPP1CC	5501	protein phosphatase 1, catalytic subunit, gamma isoform (PPP1CC), mRNA.	0.13	8.42	7.29E-14	2.28E-13	salmon
PPP4C	5531	protein phosphatase 4 (formerly X), catalytic subunit (PPP4C), mRNA.	0.06	8.35	6.09E-04	9.87E-04	salmon
PRKAR1A	5573	protein kinase, cAMP-dependent, regulatory, type I, alpha (tissue specific extinguisher 1) (PRKAR1A), transcript variant 3, mRNA.	-0.07	8.54	3.25E-05	5.91E-05	salmon
PSMC6	5706	proteasome (prosome, macropain) 26S subunit, ATPase, 6 (PSMC6), mRNA.	0.87	8.15	7.87E-65	2.83E-63	salmon

PSMD10	5716	proteasome (prosome, macropain) 26S subunit, non-ATPase, 10 (PSMD10), transcript variant 2, mRNA.	-0.02	9.83	5.51E-02	7.15E-02	salmon
PSME3	10197	proteasome (prosome, macropain) activator subunit 3 (PA28 gamma; Ki) (PSME3), transcript variant 2, mRNA.	0.01	7.66	4.72E-01	5.17E-01	salmon
PSRC1	84722	proline/serine-rich coiled-coil 1 (PSRC1), transcript variant 1, mRNA.	0.27	6.79	1.95E-32	1.41E-31	salmon
PTPN12	5782	protein tyrosine phosphatase, non-receptor type 12 (PTPN12), mRNA.	-0.15	8.76	2.57E-16	9.15E-16	salmon
RABEP1	9135	rabaptin, RAB GTPase binding effector protein 1 (RABEP1), mRNA.	-0.08	8.21	1.00E-08	2.33E-08	salmon
RINL	126432	FLJ45909 protein (FLJ45909), mRNA.	0.02	6.15	2.33E-01	2.73E-01	salmon
RNF113A	7737	ring finger protein 113A (RNF113A), mRNA.	-0.02	8.31	1.63E-01	1.96E-01	salmon
RNF145	153830	hypothetical protein FLJ31951 (FLJ31951), mRNA.	-0.08	5.84	1.21E-08	2.81E-08	salmon
RPS29	731786	PREDICTED: similar to 60S ribosomal protein L32 (LOC731786), mRNA.	0.11	7.51	9.52E-09	2.22E-08	salmon
RRAS	6237	related RAS viral (r-ras) oncogene homolog (RRAS), mRNA.	-0.02	6.63	9.76E-02	1.22E-01	salmon

RWDD2A	112611	RWD domain containing 2 (RWDD2), mRNA.	-0.08	6.33	3.54E-08	7.94E-08	salmon
SEC22A	26984	SEC22 vesicle trafficking protein homolog A (S. cerevisiae) (SEC22A), mRNA.	-0.03	9.30	4.77E-02	6.23E-02	salmon
SEPW1	6415	selenoprotein W, 1 (SEPW1), mRNA.	-0.04	6.06	2.01E-02	2.75E-02	salmon
SLC20A1	6574	solute carrier family 20 (phosphate transporter), member 1 (SLC20A1), mRNA.	0.20	7.52	1.41E-31	9.80E-31	salmon
SLC25A14	9016	solute carrier family 25 (mitochondrial carrier, brain), member 14 (SLC25A14), nuclear gene encoding mitochondrial protein, transcript variant long, mRNA.	-0.05	8.05	1.22E-04	2.10E-04	salmon
SLC27A3	11000	solute carrier family 27 (fatty acid transporter), member 3 (SLC27A3), mRNA.	-0.07	7.90	1.01E-08	2.34E-08	salmon
SNRPA1	6627	small nuclear ribonucleoprotein polypeptide A' (SNRPA1), mRNA.	0.11	9.98	8.71E-10	2.16E-09	salmon
SOCS4	122809	suppressor of cytokine signaling 4 (SOCS4), transcript variant 1, mRNA.	0.10	8.49	2.11E-12	6.07E-12	salmon
SQRDL	58472	sulfide quinone reductase-like (yeast) (SQRDL), mRNA.	0.24	8.69	7.34E-38	6.84E-37	salmon

SSB	6741	Sjogren syndrome antigen B (autoantigen La) (SSB), mRNA.	-0.17	8.59	3.47E-32	2.48E-31	salmon
SURF1	6834	surfeit 1 (SURF1), nuclear gene encoding mitochondrial protein, mRNA.	-0.32	6.03	4.39E-44	5.35E-43	salmon
TAF1B	9014	TATA box binding protein (TBP)-associated factor, RNA polymerase I, B, 63kDa (TAF1B), mRNA.	0.26	9.13	3.38E-37	3.01E-36	salmon
TAF2	6873	TAF2 RNA polymerase II, TATA box binding protein (TBP)-associated factor, 150kDa (TAF2), mRNA.	-0.21	10.24	1.63E-16	5.87E-16	salmon
TAF7	6879	TAF7 RNA polymerase II, TATA box binding protein (TBP)-associated factor, 55kDa (TAF7), mRNA.	-0.04	8.44	3.66E-03	5.46E-03	salmon
TCEA2	6919	transcription elongation factor A (SII), 2 (TCEA2), transcript variant 1, mRNA.	0.26	9.62	5.18E-41	5.42E-40	salmon
TFB2M	64216	transcription factor B2, mitochondrial (TFB2M), mRNA.	0.05	7.85	9.18E-02	1.16E-01	salmon
TLN1	7094	talin 1 (TLN1), mRNA.	-0.15	7.02	8.25E-16	2.86E-15	salmon
TM7SF2	7108	transmembrane 7 superfamily member 2 (TM7SF2), mRNA.	-0.17	7.63	5.63E-19	2.30E-18	salmon
TMEM179B	374395	similar to RIKEN cDNA 1810059G22 (LOC374395), mRNA.	-0.08	6.80	5.70E-06	1.10E-05	salmon

TRAPPC2L	51693	trafficking protein particle complex 2-like (TRAPPC2L), mRNA.	-0.05	6.13	3.01E-04	5.03E-04	salmon
TRAPPC6A	79090	trafficking protein particle complex 6A (TRAPPC6A), mRNA.	-0.03	8.91	5.20E-02	6.76E-02	salmon
TSPAN4	7106	tetraspanin 4 (TSPAN4), transcript variant 4, mRNA.	-0.12	9.32	6.08E-09	1.44E-08	salmon
UBLCP1	134510	ubiquitin-like domain containing CTD phosphatase 1 (UBLCP1), mRNA.	0.07	9.30	3.88E-09	9.29E-09	salmon
VAMP8	8673	vesicle-associated membrane protein 8 (endobrevin) (VAMP8), mRNA.	-0.09	8.21	1.05E-14	3.44E-14	salmon
VKORC1	79001	vitamin K epoxide reductase complex, subunit 1 (VKORC1), transcript variant 2, mRNA.	-0.07	7.39	4.66E-05	8.34E-05	salmon
WDR12	55759	WD repeat domain 12 (WDR12), mRNA.	0.03	8.30	2.56E-02	3.47E-02	salmon
ZFAND5	7763	zinc finger, AN1-type domain 5 (ZFAND5), mRNA.	0.01	7.39	3.21E-01	3.66E-01	salmon
ZNF239	8187	zinc finger protein 239 (ZNF239), mRNA.	-0.06	9.09	3.95E-04	6.53E-04	salmon
ZNF358	140467	zinc finger protein 358 (ZNF358), mRNA.	-0.27	9.95	8.19E-46	1.10E-44	salmon
ZWINT	11130	ZW10 interactor (ZWINT), transcript variant 2, mRNA.	-0.02	11.03	7.23E-02	9.24E-02	salmon
AARS	16	alanyl-tRNA synthetase (AARS), mRNA.	-0.31	8.18	1.17E-45	1.56E-44	tan

ABCB9	23457	ATP-binding cassette, sub-family B (MDR/TAP), member 9 (ABCB9), transcript variant 1, mRNA.	-0.32	7.45	9.87E-39	9.46E-38	tan
ACAT2	39	acetyl-Coenzyme A acetyltransferase 2 (acetoacetyl Coenzyme A thiolase) (ACAT2), mRNA.	0.11	6.94	1.10E-11	3.02E-11	tan
ACKR1	2532	Duffy blood group, chemokine receptor (DARC), mRNA.	-0.09	9.46	6.24E-08	1.38E-07	tan
ACTN1	87	actinin, alpha 1 (ACTN1), mRNA.	-0.02	9.01	4.23E-01	4.69E-01	tan
AIM2	9447	absent in melanoma 2 (AIM2), mRNA.	-0.16	7.64	5.73E-24	2.96E-23	tan
ANG	283	angiogenin, ribonuclease, RNase A family, 5 (ANG), mRNA.	0.04	8.44	4.65E-03	6.86E-03	tan
ANTXR2	118429	anthrax toxin receptor 2 (ANTXR2), mRNA.	0.00	9.01	8.90E-01	9.07E-01	tan
ARCN1	372	archain 1 (ARCN1), mRNA.	-0.17	8.88	9.33E-15	3.05E-14	tan
ARFGAP3	26286	ADP-ribosylation factor GTPase activating protein 3 (ARFGAP3), mRNA.	0.07	6.90	2.50E-06	4.96E-06	tan
ARL1	400	ADP-ribosylation factor-like 1 (ARL1), mRNA.	0.00	7.33	8.93E-01	9.09E-01	tan
ASNS	440	asparagine synthetase (ASNS), transcript variant 1, mRNA.	0.01	7.21	3.57E-01	4.03E-01	tan
ATF6	22926	activating transcription factor 6 (ATF6), mRNA.	0.14	6.66	3.05E-13	9.22E-13	tan

ATP6V0A2	23545	ATPase, H+ transporting, lysosomal V0 subunit a2 (ATP6V0A2), mRNA.	0.11	9.78	1.52E-14	4.90E-14	tan
B4GALT3	8703	UDP-Gal:betaGlcNAc beta 1,4- galactosyltransferase, polypeptide 3 (B4GALT3), mRNA.	-0.13	6.09	1.29E-17	4.91E-17	tan
BTN3A2	11118	butyrophilin, subfamily 3, member A2 (BTN3A2), mRNA.	0.21	7.62	1.18E-27	7.18E-27	tan
CAMK1G	57172	calcium/calmodulin-dependent protein kinase IG (CAMK1G), mRNA.	-0.01	6.43	5.87E-01	6.29E-01	tan
CARD9	64170	caspase recruitment domain family, member 9 (CARD9), mRNA.	0.12	6.61	1.18E-10	3.06E-10	tan
CDK12	51755	Cdc2-related kinase, arginine/serine-rich (CRKRS), mRNA.	0.12	7.24	5.05E-12	1.42E-11	tan
CHKA	1119	choline kinase alpha (CHKA), transcript variant 1, mRNA.	0.63	5.40	7.66E-55	1.51E-53	tan
CKAP4	10970	cytoskeleton-associated protein 4 (CKAP4), mRNA.	-0.02	7.96	2.32E-01	2.72E-01	tan
COPA	1314	coatomer protein complex, subunit alpha (COPA), mRNA.	0.00	9.94	8.53E-01	8.74E-01	tan
COPB2	9276	coatomer protein complex, subunit beta 2 (beta prime) (COPB2), mRNA.	0.24	5.92	5.80E-22	2.71E-21	tan
COPG1	22820	coatomer protein complex, subunit gamma (COPG), mRNA.	0.13	9.31	8.32E-12	2.31E-11	tan

CRELD1	78987	cysteine-rich with EGF-like domains 1 (CRELD1), transcript variant 1, mRNA.	0.08	7.35	4.24E-05	7.63E-05	tan
CRELD2	79174	cysteine-rich with EGF-like domains 2 (CRELD2), mRNA.	-0.04	7.35	4.15E-04	6.84E-04	tan
CSTB	1476	cystatin B (stefin B) (CSTB), mRNA.	-0.05	7.59	5.80E-03	8.46E-03	tan
CTH	1491	cystathionase (cystathionine gamma-lyase) (CTH), transcript variant 1, mRNA.	0.02	9.29	1.31E-01	1.60E-01	tan
CYB5A	1528	cytochrome b5 type A (microsomal) (CYB5A), transcript variant 1, mRNA.	0.09	9.93	4.84E-10	1.22E-09	tan
DDOST	1650	dolichyl-diphosphooligosaccharide-protein glycosyltransferase (DDOST), mRNA.	0.05	7.42	6.63E-06	1.28E-05	tan
DECR2	26063	2,4-dienoyl CoA reductase 2, peroxisomal (DECR2), mRNA.	-0.02	7.30	1.27E-01	1.56E-01	tan
DERL2	51009	Der1-like domain family, member 2 (DERL2), mRNA.	-0.04	10.16	6.33E-03	9.22E-03	tan
DNAJB11	51726	DnaJ (Hsp40) homolog, subfamily B, member 11 (DNAJB11), mRNA.	-0.04	7.39	1.19E-03	1.87E-03	tan
DNAJB9	4189	DnaJ (Hsp40) homolog, subfamily B, member 9 (DNAJB9), mRNA.	0.03	7.14	4.05E-02	5.35E-02	tan
DNAJC3	5611	DnaJ (Hsp40) homolog, subfamily C, member 3 (DNAJC3), mRNA.	-0.27	6.64	6.66E-52	1.13E-50	tan

DPAGT1	1798	dolichyl-phosphate (UDP-N-acetylglucosamine) N-acetylglucosaminephosphotransferase 1 (GlcNAc-1-P transferase) (DPAGT1), transcript variant 2, mRNA.	0.12	8.96	1.93E-17	7.30E-17	tan
DUSP14	11072	dual specificity phosphatase 14 (DUSP14), mRNA.	-0.20	9.98	3.14E-39	3.06E-38	tan
EDEM2	55741	ER degradation enhancer, mannosidase alpha-like 2 (EDEM2), mRNA.	0.12	5.89	7.98E-11	2.10E-10	tan
ELL2	22936	elongation factor, RNA polymerase II, 2 (ELL2), mRNA.	-0.42	7.10	3.56E-62	1.11E-60	tan
EPHX2	2053	epoxide hydrolase 2, cytoplasmic (EPHX2), mRNA.	0.12	7.62	1.71E-11	4.67E-11	tan
ERGIC1	57222	endoplasmic reticulum-golgi intermediate compartment (ERGIC) 1 (ERGIC1), transcript variant 1, mRNA.	0.49	5.99	1.23E-42	1.42E-41	tan
ERLEC1	27248	chromosome 2 open reading frame 30 (C2orf30), mRNA.	-0.13	7.69	9.90E-08	2.16E-07	tan
EVI2B	2124	ecotropic viral integration site 2B (EVI2B), mRNA.	-0.03	9.16	8.12E-03	1.17E-02	tan
FADS1	3992	fatty acid desaturase 1 (FADS1), mRNA.	0.29	7.19	6.97E-35	5.53E-34	tan
FAIM3	9214	Fas apoptotic inhibitory molecule 3 (FAIM3), mRNA.	-0.03	5.68	5.07E-02	6.61E-02	tan
FAM214A	56204	KIAA1370 (KIAA1370), mRNA.	-0.11	6.76	2.05E-14	6.56E-14	tan

FAM46C	54855	family with sequence similarity 46, member C (FAM46C), mRNA.	-0.11	8.17	2.75E-10	7.03E-10	tan
FICD	11153	Huntingtin interacting protein E (HYPE), mRNA.	-0.03	8.02	7.25E-02	9.26E-02	tan
FKBP11	51303	FK506 binding protein 11, 19 kDa (FKBP11), mRNA.	0.02	5.80	1.74E-01	2.09E-01	tan
FKBP2	2286	FK506 binding protein 2, 13kDa (FKBP2), transcript variant 1, mRNA.	0.26	6.13	3.18E-30	2.07E-29	tan
FNDC3B	64778	fibronectin type III domain containing 3B (FNDC3B), mRNA.	0.04	6.65	1.27E-03	1.98E-03	tan
FRMD6	122786	FERM domain containing 6 (FRMD6), transcript variant 1, mRNA.	0.05	6.43	2.01E-02	2.76E-02	tan
GALM	130589	galactose mutarotase (aldose 1-epimerase) (GALM), mRNA.	0.29	8.94	1.94E-33	1.44E-32	tan
GARS	2617	glycyl-tRNA synthetase (GARS), mRNA.	0.03	7.45	2.68E-02	3.62E-02	tan
GMPPA	29926	GDP-mannose pyrophosphorylase A (GMPPA), transcript variant 1, mRNA.	0.01	10.83	5.89E-01	6.31E-01	tan
GMPPB	29925	GDP-mannose pyrophosphorylase B (GMPPB), transcript variant 1, mRNA.	-0.04	6.83	4.04E-02	5.34E-02	tan
GORASP2	26003	golgi reassembly stacking protein 2, 55kDa (GORASP2), mRNA.	0.07	9.61	3.66E-04	6.06E-04	tan

GPX4	2879	glutathione peroxidase 4 (phospholipid hydroperoxidase) (GPX4), transcript variant 3, mRNA.	-0.06	7.50	7.12E-06	1.37E-05	tan
GSTT1	2952	glutathione S-transferase theta 1 (GSTT1), mRNA.	-0.12	7.28	1.51E-06	3.03E-06	tan
H3F3B	3021	H3 histone, family 3B (H3.3B) (H3F3B), mRNA.	-0.12	8.96	2.77E-16	9.81E-16	tan
HENMT1	113802	chromosome 1 open reading frame 59 (C1orf59), mRNA.	0.20	6.61	3.10E-23	1.55E-22	tan
HERPUD1	9709	homocysteine-inducible, endoplasmic reticulum stress-inducible, ubiquitin-like domain member 1 (HERPUD1), transcript variant 2, mRNA.	0.10	7.01	1.51E-14	4.88E-14	tan
HIP1R	728014	PREDICTED: similar to huntingtin interacting protein 1 related (LOC728014), mRNA.	0.00	6.75	8.80E-01	8.98E-01	tan
HLA-DRB1	3123	major histocompatibility complex, class II, DR beta 1 (HLA-DRB1), mRNA.	-0.09	11.56	8.43E-10	2.09E-09	tan
HSPA5	3309	heat shock 70kDa protein 5 (glucose-regulated protein, 78kDa) (HSPA5), mRNA.	0.03	10.50	4.19E-02	5.52E-02	tan
HYOU1	10525	hypoxia up-regulated 1 (HYOU1), mRNA.	-0.25	9.16	5.39E-31	3.61E-30	tan

IGJ	3512	immunoglobulin J polypeptide, linker protein for immunoglobulin alpha and mu polypeptides (IGJ), mRNA.	0.15	8.17	5.05E-16	1.77E-15	tan
IMMP2L	83943	IMP2 inner mitochondrial membrane peptidase-like (S. cerevisiae) (IMMP2L), mRNA.	0.00	5.63	7.44E-01	7.75E-01	tan
ITGA4	3676	integrin, alpha 4 (antigen CD49D, alpha 4 subunit of VLA-4 receptor) (ITGA4), mRNA.	-0.06	6.79	3.00E-04	5.02E-04	tan
ITM2C	81618	integral membrane protein 2C (ITM2C), transcript variant 1, mRNA.	0.27	6.97	2.03E-24	1.07E-23	tan
KCND2	3751	potassium voltage-gated channel, Shal-related subfamily, member 2 (KCND2), mRNA.	0.05	9.27	4.15E-04	6.85E-04	tan
KCNN3	3782	potassium intermediate/small conductance calcium-activated channel, subfamily N, member 3 (KCNN3), transcript variant 2, mRNA.	0.08	8.54	1.40E-04	2.39E-04	tan
KCTD5	54442	potassium channel tetramerisation domain containing 5 (KCTD5), mRNA.	-0.25	6.50	1.49E-31	1.03E-30	tan
KIAA0125	9834	KIAA0125 (KIAA0125), mRNA.	0.15	9.50	1.98E-25	1.09E-24	tan
KIAA1549L	120196	chromosome 11 open reading frame 69 (C11orf69), mRNA.	-0.49	9.38	3.25E-75	1.92E-73	tan

KLHL42	57542	kelch domain containing 5 (KLHDC5), mRNA.	0.12	5.35	4.33E-07	9.05E-07	tan
LHX2	9355	LIM homeobox 2 (LHX2), mRNA.	-0.01	6.38	6.61E-01	6.98E-01	tan
LRP10	26020	low density lipoprotein receptor-related protein 10 (LRP10), mRNA.	-0.22	10.37	8.20E-35	6.45E-34	tan
LRRFIP2	9209	leucine rich repeat (in FLII) interacting protein 2 (LRRFIP2), transcript variant 2, mRNA.	-0.11	7.01	4.18E-12	1.18E-11	tan
MAGED1	9500	melanoma antigen family D, 1 (MAGED1), transcript variant 3, mRNA.	0.03	7.25	5.05E-02	6.58E-02	tan
MAN2A1	4124	mannosidase, alpha, class 2A, member 1 (MAN2A1), mRNA.	0.00	9.11	7.51E-01	7.82E-01	tan
MANF	7873	arginine-rich, mutated in early stage tumors (ARMET), mRNA.	0.14	9.55	3.45E-23	1.71E-22	tan
MAP3K7CL	56911	chromosome 21 open reading frame 7 (C21orf7), mRNA.	-0.08	9.64	5.48E-15	1.81E-14	tan
MAPKAPK2	9261	mitogen-activated protein kinase-activated protein kinase 2 (MAPKAPK2), transcript variant 2, mRNA.	-0.03	6.99	4.71E-02	6.16E-02	tan
MIS12	79003	MIS12, MIND kinetochore complex component, homolog (yeast) (MIS12), mRNA.	-0.06	9.81	5.09E-09	1.21E-08	tan

MRPS31	10240	mitochondrial ribosomal protein S31 (MRPS31), nuclear gene encoding mitochondrial protein, mRNA.	0.90	9.36	1.03E-70	4.81E-69	tan
MYDGF	56005	chromosome 19 open reading frame 10 (C19orf10), mRNA.	-0.03	7.44	1.81E-01	2.16E-01	tan
NA	51237	proapoptotic caspase adaptor protein (PACAP), mRNA.	-0.28	7.79	4.41E-45	5.64E-44	tan
NA	284040	CMT1A duplicated region transcript 4 (CDRT4), mRNA.	0.15	6.81	2.60E-18	1.02E-17	tan
NA	2081	endoplasmic reticulum to nucleus signalling 1 (ERN1), transcript variant 2, mRNA.	-0.11	8.85	3.25E-15	1.08E-14	tan
NANS	54187	N-acetylneuraminic acid synthase (sialic acid synthase) (NANS), mRNA.	0.00	6.34	9.97E-01	9.97E-01	tan
NAPSA	9476	napsin A aspartic peptidase (NAPSA), mRNA.	0.02	9.67	1.31E-01	1.60E-01	tan
NECAP2	55707	NECAP endocytosis associated 2 (NECAP2), mRNA.	0.05	9.07	4.74E-04	7.78E-04	tan
NUCB1	4924	nucleobindin 1 (NUCB1), mRNA.	0.10	8.44	3.87E-08	8.65E-08	tan
OAF	220323	OAF homolog (Drosophila) (OAF), mRNA.	-0.27	5.76	7.89E-37	6.93E-36	tan
P4HB	5034	procollagen-proline, 2-oxoglutarate 4-dioxygenase (proline 4-hydroxylase), beta polypeptide (P4HB), mRNA.	0.08	10.02	8.27E-09	1.94E-08	tan

PAM	5066	peptidylglycine alpha-amidating monooxygenase (PAM), transcript variant 3, mRNA.	0.02	7.18	1.96E-01	2.33E-01	tan
PDCD1	5133	programmed cell death 1 (PDCD1), mRNA.	0.00	9.48	6.46E-01	6.84E-01	tan
PDIA4	9601	protein disulfide isomerase family A, member 4 (PDIA4), mRNA.	-0.03	7.50	2.98E-02	3.99E-02	tan
PDIA5	10954	protein disulfide isomerase family A, member 5 (PDIA5), mRNA.	-0.10	9.24	3.29E-16	1.17E-15	tan
PHGDH	26227	phosphoglycerate dehydrogenase (PHGDH), mRNA.	-0.08	8.41	7.70E-13	2.28E-12	tan
PIM2	11040	pim-2 oncogene (PIM2), mRNA.	0.05	8.73	5.71E-07	1.18E-06	tan
PNOC	5368	prepronociceptin (PNOC), mRNA.	0.17	7.76	3.02E-28	1.85E-27	tan
POP5	51367	processing of precursor 5, ribonuclease P/MRP subunit (S. cerevisiae) (POP5), transcript variant 1, mRNA.	-0.22	6.03	3.68E-20	1.59E-19	tan
PPAPDC1B	84513	phosphatidic acid phosphatase type 2 domain containing 1B (PPAPDC1B), mRNA.	-0.26	9.29	5.19E-29	3.25E-28	tan
PPIB	5479	peptidylprolyl isomerase B (cyclophilin B) (PPIB), mRNA.	-0.09	11.79	6.83E-11	1.81E-10	tan
PPP3CC	5533	protein phosphatase 3 (formerly 2B), catalytic subunit, gamma isoform (PPP3CC), mRNA.	-0.15	8.36	1.01E-18	4.06E-18	tan

PPP3R1	5534	protein phosphatase 3 (formerly 2B), regulatory subunit B, alpha isoform (PPP3R1), mRNA.	0.01	8.08	3.66E-01	4.11E-01	tan
PRRT3	285368	proline-rich transmembrane protein 3 (PRRT3), mRNA.	0.04	9.10	1.39E-01	1.69E-01	tan
PSTPIP2	9050	proline-serine-threonine phosphatase interacting protein 2 (PSTPIP2), mRNA.	-0.10	7.48	2.37E-10	6.08E-10	tan
PTGER4	5734	prostaglandin E receptor 4 (subtype EP4) (PTGER4), mRNA.	0.01	10.98	3.44E-01	3.89E-01	tan
PTPN13	5783	protein tyrosine phosphatase, non-receptor type 13 (APO-1/CD95 (Fas)-associated phosphatase) (PTPN13), transcript variant 4, mRNA.	0.75	9.12	1.09E-80	1.01E-78	tan
RAB30	27314	RAB30, member RAS oncogene family (RAB30), mRNA.	0.02	7.01	2.35E-01	2.75E-01	tan
RABAC1	10567	Rab acceptor 1 (prenylated) (RABAC1), mRNA.	0.01	9.34	4.99E-01	5.44E-01	tan
RALA	5898	v-ral simian leukemia viral oncogene homolog A (ras related) (RALA), mRNA.	0.05	9.13	1.68E-03	2.60E-03	tan
RCN1	728913	PREDICTED: similar to Reticulocalbin-1 precursor (LOC728913), mRNA.	-0.01	5.79	4.50E-01	4.96E-01	tan

RHOQ	23433	ras homolog gene family, member Q (RHOQ), mRNA.	0.01	9.48	7.99E-01	8.26E-01	tan
RNF103	7844	ring finger protein 103 (RNF103), mRNA.	-0.05	6.47	1.05E-03	1.66E-03	tan
RPN1	6184	ribophorin I (RPN1), mRNA.	0.37	10.98	9.10E-50	1.42E-48	tan
SCFD1	23256	sec1 family domain containing 1 (SCFD1), transcript variant 2, mRNA.	0.02	11.75	4.57E-01	5.03E-01	tan
SDF2L1	23753	stromal cell-derived factor 2-like 1 (SDF2L1), mRNA.	-0.07	9.09	5.03E-04	8.23E-04	tan
SEC24C	9632	SEC24 related gene family, member C (S. cerevisiae) (SEC24C), transcript variant 2, mRNA.	0.24	7.41	1.34E-27	8.08E-27	tan
SEC31A	22872	SEC31 homolog A (S. cerevisiae) (SEC31A), transcript variant 2, mRNA.	0.08	8.35	1.46E-07	3.16E-07	tan
SELM	140606	selenoprotein M (SELM), mRNA.	0.80	7.34	4.64E-102	1.96E-99	tan
SEMA4A	64218	sema domain, immunoglobulin domain (Ig), transmembrane domain (TM) and short cytoplasmic domain, (semaphorin) 4A (SEMA4A), mRNA.	0.13	6.41	1.31E-13	4.03E-13	tan
SHMT2	6472	serine hydroxymethyltransferase 2 (mitochondrial) (SHMT2), mRNA.	0.32	6.34	9.98E-48	1.48E-46	tan

SIL1	64374	SIL1 homolog, endoplasmic reticulum chaperone (S. cerevisiae) (SIL1), transcript variant 2, mRNA.	-0.23	6.74	4.20E-26	2.38E-25	tan
SLAIN1	122060	SLAIN motif family, member 1 (SLAIN1), transcript variant 1, mRNA.	0.05	7.65	2.82E-04	4.73E-04	tan
SLC35B1	10237	solute carrier family 35, member B1 (SLC35B1), mRNA.	0.10	6.18	6.52E-07	1.34E-06	tan
SLC38A10	124565	hypothetical protein MGC15523 (MGC15523), transcript variant 2, mRNA.	0.03	7.37	3.73E-03	5.55E-03	tan
SLC39A14	23516	solute carrier family 39 (zinc transporter), member 14 (SLC39A14), mRNA.	-0.04	10.01	9.45E-03	1.35E-02	tan
SLC7A1	6541	solute carrier family 7 (cationic amino acid transporter, y+ system), member 1 (SLC7A1), mRNA.	-0.18	8.22	2.51E-22	1.19E-21	tan
SLC7A5	8140	solute carrier family 7 (cationic amino acid transporter, y+ system), member 5 (SLC7A5), mRNA.	-0.06	8.49	1.51E-08	3.47E-08	tan
SMCO4	56935	chromosome 11 open reading frame 75 (C11orf75), mRNA.	0.02	7.36	3.99E-01	4.45E-01	tan
SPCS2	9789	signal peptidase complex subunit 2 homolog (S. cerevisiae) (SPCS2), mRNA.	0.01	6.98	7.63E-01	7.92E-01	tan

SPCS3	60559	signal peptidase complex subunit 3 homolog (S. cerevisiae) (SPCS3), mRNA.	-0.22	8.78	1.88E-37	1.70E-36	tan
SPRED1	161742	sprouty-related, EVH1 domain containing 1 (SPRED1), mRNA.	-0.01	10.36	5.84E-01	6.26E-01	tan
SPTY2D1	144108	SPT2, Suppressor of Ty, domain containing 1 (S. cerevisiae) (SPTY2D1), mRNA.	-0.08	8.09	8.94E-07	1.83E-06	tan
SRPR	6734	signal recognition particle receptor ('docking protein') (SRPR), mRNA.	-0.02	7.67	1.55E-01	1.88E-01	tan
ST6GALNAC4	27090	ST6 (alpha-N-acetyl-neuraminyl-2,3-beta-galactosyl-1, 3)-N-acetylgalactosaminide alpha-2,6-sialyltransferase 4 (ST6GALNAC4), transcript variant 2, mRNA.	-0.19	7.70	3.00E-20	1.30E-19	tan
SURF4	6836	surfeit 4 (SURF4), mRNA.	0.01	12.40	4.28E-01	4.74E-01	tan
SYVN1	84447	synovial apoptosis inhibitor 1, synoviolin (SYVN1), transcript variant 1, mRNA.	-0.01	7.25	5.42E-01	5.86E-01	tan
TLR9	54106	toll-like receptor 9 (TLR9), transcript variant A, mRNA.	-0.21	5.62	4.73E-25	2.57E-24	tan
TM9SF1	10548	transmembrane 9 superfamily member 1 (TM9SF1), transcript variant 1, mRNA.	-0.02	7.71	2.21E-01	2.60E-01	tan

TMED5	50999	transmembrane emp24 protein transport domain containing 5 (TMED5), mRNA.	0.01	7.57	5.48E-01	5.92E-01	tan
TMED9	54732	transmembrane emp24 protein transport domain containing 9 (TMED9), mRNA.	0.05	10.66	1.16E-04	2.00E-04	tan
TMEM5	10329	transmembrane protein 5 (TMEM5), mRNA.	-0.13	8.03	3.29E-19	1.36E-18	tan
TMEM59	9528	transmembrane protein 59 (TMEM59), mRNA.	-0.02	6.34	2.67E-01	3.09E-01	tan
TSPAN12	23554	tetraspanin 12 (TSPAN12), mRNA.	0.27	8.81	1.08E-40	1.12E-39	tan
TXNDC11	51061	thioredoxin domain containing 11 (TXNDC11), mRNA.	-0.04	9.55	2.89E-02	3.88E-02	tan
UBALD2	283991	family with sequence similarity 100, member B (FAM100B), mRNA.	0.02	8.59	1.22E-01	1.51E-01	tan
VPS37B	79720	vacuolar protein sorting 37 homolog B (S. cerevisiae) (VPS37B), mRNA.	-0.13	8.72	2.94E-11	7.93E-11	tan
WARS	7453	tryptophanyl-tRNA synthetase (WARS), transcript variant 3, mRNA.	0.08	6.84	4.49E-07	9.35E-07	tan
WDR33	55339	WD repeat domain 33 (WDR33), transcript variant 2, mRNA.	-0.25	6.47	7.62E-35	6.02E-34	tan
WIPI1	55062	WD repeat domain, phosphoinositide interacting 1 (WIPI1), mRNA.	-0.03	8.27	1.03E-01	1.28E-01	tan
XBP1	7494	X-box binding protein 1 (XBP1), transcript variant 1, mRNA.	0.10	5.81	3.23E-11	8.67E-11	tan

AAED1	195827	chromosome 9 open reading frame 21 (C9orf21), mRNA.	-0.13	8.52	3.55E-09	8.51E-09	turquoise
AAMDC	28971	chromosome 11 open reading frame 67 (C11orf67), mRNA.	0.07	6.25	1.21E-03	1.89E-03	turquoise
ABCA6	23460	ATP-binding cassette, sub-family A (ABC1), member 6 (ABCA6), mRNA.	0.12	9.62	5.72E-14	1.80E-13	turquoise
ABCB10	23456	ATP-binding cassette, sub-family B (MDR/TAP), member 10 (ABCB10), nuclear gene encoding mitochondrial protein, mRNA.	0.24	9.06	1.70E-40	1.75E-39	turquoise
ABCC4	10257	ATP-binding cassette, sub-family C (CFTR/MRP), member 4 (ABCC4), mRNA.	0.02	7.26	1.90E-01	2.27E-01	turquoise
ABHD6	57406	abhydrolase domain containing 6 (ABHD6), mRNA.	0.13	8.36	2.26E-12	6.49E-12	turquoise
ABI1	10006	abl-interactor 1 (ABI1), transcript variant 1, mRNA.	-0.01	9.93	6.40E-01	6.79E-01	turquoise
ABI3	51225	ABI gene family, member 3 (ABI3), mRNA.	-0.05	9.52	1.78E-03	2.75E-03	turquoise
ACADM	34	acyl-Coenzyme A dehydrogenase, C-4 to C-12 straight chain (ACADM), nuclear gene encoding mitochondrial protein, mRNA.	0.03	7.82	7.29E-02	9.30E-02	turquoise
ACKR3	57007	chemokine (C-X-C motif) receptor 7 (CXCR7), transcript variant 2, mRNA.	-0.01	7.81	4.03E-01	4.49E-01	turquoise

ACO1	48	aconitase 1, soluble (ACO1), mRNA.	0.05	7.06	6.69E-04	1.08E-03	turquoise
ACOT4	122970	acyl-CoA thioesterase 4 (ACOT4), mRNA.	0.04	5.77	9.26E-03	1.32E-02	turquoise
ACOX3	8310	acyl-Coenzyme A oxidase 3, pristanoyl (ACOX3), mRNA.	-0.06	5.39	1.07E-04	1.86E-04	turquoise
ACP6	51205	acid phosphatase 6, lysophosphatidic (ACP6), mRNA.	-0.18	8.34	4.67E-24	2.42E-23	turquoise
ACSL4	2182	acyl-CoA synthetase long-chain family member 4 (ACSL4), transcript variant 1, mRNA.	0.19	6.82	1.62E-22	7.75E-22	turquoise
ADAM19	8728	ADAM metallopeptidase domain 19 (meltrin beta) (ADAM19), transcript variant 2, mRNA.	-0.11	8.15	3.55E-10	9.02E-10	turquoise
ADAM8	101	ADAM metallopeptidase domain 8 (ADAM8), mRNA.	-0.10	7.77	8.88E-12	2.47E-11	turquoise
ADAP1	11033	centaurin, alpha 1 (CENTA1), mRNA.	-0.16	6.41	1.17E-16	4.26E-16	turquoise
ADCK3	56997	chaperone, ABC1 activity of bc1 complex homolog (S. pombe) (CABC1), nuclear gene encoding mitochondrial protein, mRNA.	-0.06	9.46	2.23E-06	4.44E-06	turquoise
ADD1	118	adducin 1 (alpha) (ADD1), transcript variant 1, mRNA.	0.04	7.85	3.53E-03	5.27E-03	turquoise
ADIPOR2	79602	adiponectin receptor 2 (ADIPOR2), mRNA.	0.06	10.23	6.35E-04	1.03E-03	turquoise

ADO	84890	chromosome 10 open reading frame 22 (C10orf22), mRNA.	-0.16	9.05	7.55E-19	3.06E-18	turquoise
ADORA2B	136	adenosine A2b receptor (ADORA2B), mRNA.	0.10	8.50	5.07E-13	1.51E-12	turquoise
AEN	64782	interferon stimulated exonuclease gene 20kDa-like 1 (ISG20L1), mRNA.	0.00	7.16	9.35E-01	9.45E-01	turquoise
AHNAK	79026	AHNAK nucleoprotein (desmoyokin) (AHNAK), transcript variant 1, mRNA.	-0.07	7.65	3.45E-07	7.25E-07	turquoise
AHSA1	10598	AHA1, activator of heat shock 90kDa protein ATPase homolog 1 (yeast) (AHSA1), mRNA.	0.12	9.89	3.79E-13	1.14E-12	turquoise
AICDA	57379	activation-induced cytidine deaminase (AICDA), mRNA.	-0.02	6.87	2.63E-01	3.05E-01	turquoise
AIM1	202	absent in melanoma 1 (AIM1), mRNA.	0.21	8.28	8.90E-20	3.76E-19	turquoise
AKAP11	11215	A kinase (PRKA) anchor protein 11 (AKAP11), transcript variant 1, mRNA.	0.20	5.95	2.62E-17	9.82E-17	turquoise
AKAP13	11214	A kinase (PRKA) anchor protein 13 (AKAP13), transcript variant 1, mRNA.	0.06	10.08	9.38E-04	1.49E-03	turquoise
AKAP7	9465	A kinase (PRKA) anchor protein 7 (AKAP7), transcript variant gamma, mRNA.	0.07	7.32	5.22E-06	1.01E-05	turquoise
AKIRIN1	79647	chromosome 1 open reading frame 108 (C1orf108), mRNA.	0.01	8.45	5.35E-01	5.80E-01	turquoise

AKIRIN2	55122	chromosome 6 open reading frame 166 (C6orf166), mRNA.	-0.26	9.95	3.94E-39	3.80E-38	turquoise
ALDH5A1	7915	aldehyde dehydrogenase 5 family, member A1 (succinate-semialdehyde dehydrogenase) (ALDH5A1), nuclear gene encoding mitochondrial protein, transcript variant 1, mRNA.	-0.10	7.01	1.19E-09	2.93E-09	turquoise
AMMECR1	9949	Alport syndrome, mental retardation, midface hypoplasia and elliptocytosis chromosomal region, gene 1 (AMMECR1), transcript variant 1, mRNA.	0.03	9.47	8.71E-02	1.10E-01	turquoise
ANGPTL6	83854	angiopoietin-like 6 (ANGPTL6), mRNA.	-0.09	6.53	1.91E-07	4.10E-07	turquoise
ANKRD37	353322	ankyrin repeat domain 37 (ANKRD37), mRNA.	-0.05	5.87	2.05E-03	3.14E-03	turquoise
ANXA2R	389289	similar to annexin II receptor (AXIIR), mRNA.	0.21	7.29	6.51E-29	4.06E-28	turquoise
ANXA5	308	annexin A5 (ANXA5), mRNA.	-0.06	6.67	6.00E-05	1.06E-04	turquoise
AP3M2	10947	adaptor-related protein complex 3, mu 2 subunit (AP3M2), mRNA.	-0.25	6.89	2.15E-27	1.28E-26	turquoise
AP3S1	1176	adaptor-related protein complex 3, sigma 1 subunit (AP3S1), transcript variant 1, mRNA.	0.49	6.75	4.20E-54	7.96E-53	turquoise

APAF1	317	apoptotic peptidase activating factor 1 (APAF1), transcript variant 3, mRNA.	-0.39	9.04	1.02E-52	1.77E-51	turquoise
AQP9	366	aquaporin 9 (AQP9), mRNA.	-0.09	8.23	7.60E-10	1.89E-09	turquoise
ARHGAP1	392	Rho GTPase activating protein 1 (ARHGAP1), mRNA.	0.08	9.10	4.68E-06	9.12E-06	turquoise
ARHGAP15	55843	Rho GTPase activating protein 15 (ARHGAP15), mRNA.	0.03	6.15	7.02E-02	8.98E-02	turquoise
ARHGAP18	93663	Rho GTPase activating protein 18 (ARHGAP18), mRNA.	0.02	6.23	2.38E-01	2.78E-01	turquoise
ARHGAP21	57584	Rho GTPase activating protein 21 (ARHGAP21), mRNA.	0.00	9.75	8.51E-01	8.73E-01	turquoise
ARHGAP9	64333	Rho GTPase activating protein 9 (ARHGAP9), transcript variant 2, mRNA.	-0.26	8.24	2.58E-43	3.04E-42	turquoise
ARHGEF2	9181	rho/rac guanine nucleotide exchange factor (GEF) 2 (ARHGEF2), mRNA.	-0.10	7.47	3.07E-13	9.27E-13	turquoise
ARHGEF3	50650	Rho guanine nucleotide exchange factor (GEF) 3 (ARHGEF3), mRNA.	-0.03	7.04	9.99E-02	1.25E-01	turquoise
ARHGEF6	9459	Rac/Cdc42 guanine nucleotide exchange factor (GEF) 6 (ARHGEF6), mRNA.	-0.01	5.21	5.52E-01	5.96E-01	turquoise

ARID1A	8289	AT rich interactive domain 1A (SWI-like) (ARID1A), transcript variant 2, mRNA.	0.02	7.60	3.70E-01	4.15E-01	turquoise
ARID3A	1820	AT rich interactive domain 3A (BRIGHT-like) (ARID3A), mRNA.	0.19	7.20	6.59E-35	5.24E-34	turquoise
ARL5A	26225	ADP-ribosylation factor-like 5A (ARL5A), transcript variant 2, mRNA.	-0.19	8.15	6.85E-20	2.91E-19	turquoise
ARPC5L	81873	actin related protein 2/3 complex, subunit 5-like (ARPC5L), mRNA.	0.01	8.77	4.67E-01	5.12E-01	turquoise
ASAP1	50807	development and differentiation enhancing factor 1 (DDEF1), mRNA.	0.15	8.16	1.70E-18	6.75E-18	turquoise
ASCL1	429	achaete-scute complex homolog 1 (Drosophila) (ASCL1), mRNA.	-0.09	9.06	6.62E-14	2.08E-13	turquoise
ASF1A	25842	ASF1 anti-silencing function 1 homolog A (S. cerevisiae) (ASF1A), mRNA.	-0.41	6.65	1.30E-55	2.68E-54	turquoise
ATF5	22809	activating transcription factor 5 (ATF5), mRNA.	-0.18	8.00	3.71E-36	3.13E-35	turquoise
ATL3	25923	DKFZP564J0863 protein (DKFZP564J0863), mRNA.	0.05	6.07	7.34E-04	1.18E-03	turquoise
ATP11C	286410	ATPase, Class VI, type 11C (ATP11C), transcript variant 2, mRNA.	-0.01	9.31	5.80E-01	6.23E-01	turquoise
ATP1A1	476	ATPase, Na+/K+ transporting, alpha 1 polypeptide (ATP1A1), transcript variant 1, mRNA.	0.19	8.31	2.98E-15	9.95E-15	turquoise

ATP6V0A1	535	ATPase, H+ transporting, lysosomal V0 subunit a1 (ATP6V0A1), mRNA.	-0.05	6.82	1.02E-03	1.61E-03	turquoise
ATXN1	6310	ataxin 1 (ATXN1), mRNA.	-0.01	8.98	4.70E-01	5.15E-01	turquoise
AUH	549	AU RNA binding protein/enoyl-Coenzyme A hydratase (AUH), nuclear gene encoding mitochondrial protein, mRNA.	0.08	7.93	5.19E-05	9.26E-05	turquoise
AZIN1	51582	antizyme inhibitor 1 (AZIN1), transcript variant 1, mRNA.	0.71	7.10	8.19E-59	2.15E-57	turquoise
BCAR3	8412	breast cancer anti-estrogen resistance 3 (BCAR3), mRNA.	-0.05	8.74	8.49E-03	1.22E-02	turquoise
BCCIP	56647	BRCA2 and CDKN1A interacting protein (BCCIP), transcript variant B, mRNA.	-0.03	7.54	2.96E-02	3.96E-02	turquoise
BCDIN3D	144233	hypothetical protein LOC144233 (LOC144233), mRNA.	0.02	11.12	3.50E-01	3.95E-01	turquoise
BCKDHA	593	branched chain keto acid dehydrogenase E1, alpha polypeptide (BCKDHA), mRNA.	0.10	7.29	2.52E-06	4.99E-06	turquoise
BEND6	221336	chromosome 6 open reading frame 65 (C6orf65), mRNA.	-0.11	8.76	3.80E-12	1.08E-11	turquoise
BET1L	51272	blocked early in transport 1 homolog (S. cerevisiae)-like (BET1L), mRNA.	-0.16	8.32	2.09E-17	7.89E-17	turquoise

BIK	638	BCL2-interacting killer (apoptosis-inducing) (BIK), mRNA.	0.03	11.64	2.49E-02	3.37E-02	turquoise
BIN1	274	bridging integrator 1 (BIN1), transcript variant 6, mRNA.	-0.17	8.77	1.90E-30	1.24E-29	turquoise
BLMH	642	bleomycin hydrolase (BLMH), mRNA.	-0.09	9.28	2.92E-08	6.59E-08	turquoise
BLNK	29760	B-cell linker (BLNK), mRNA.	-0.07	7.95	1.18E-07	2.56E-07	turquoise
BMF	90427	Bcl2 modifying factor (BMF), transcript variant 2, mRNA.	0.18	5.53	2.08E-16	7.44E-16	turquoise
BMP2K	55589	BMP2 inducible kinase (BMP2K), transcript variant 2, mRNA.	0.02	8.75	1.22E-01	1.50E-01	turquoise
BMP6	654	bone morphogenetic protein 6 (BMP6), mRNA.	-0.04	8.74	3.41E-04	5.66E-04	turquoise
BNIP3L	665	BCL2/adenovirus E1B 19kDa interacting protein 3-like (BNIP3L), mRNA.	0.09	7.39	7.23E-09	1.70E-08	turquoise
BRCA1	672	breast cancer 1, early onset (BRCA1), transcript variant BRCA1-delta14-17, mRNA.	0.06	8.84	2.48E-05	4.55E-05	turquoise
BRD3	8019	bromodomain containing 3 (BRD3), mRNA.	-0.02	6.17	1.53E-01	1.86E-01	turquoise
BTBD6	90135	BTB (POZ) domain containing 6 (BTBD6), mRNA.	0.25	10.39	6.48E-35	5.15E-34	turquoise
BTG1	694	B-cell translocation gene 1, anti-proliferative (BTG1), mRNA.	-0.03	7.97	5.30E-02	6.90E-02	turquoise
BTG2	7832	BTG family, member 2 (BTG2), mRNA.	0.04	9.61	2.43E-02	3.30E-02	turquoise

BTG3	10950	BTG family, member 3 (BTG3), mRNA.	0.10	7.68	4.48E-12	1.26E-11	turquoise
BTK	695	Bruton agammaglobulinemia tyrosine kinase (BTK), mRNA.	0.40	5.92	2.93E-46	4.02E-45	turquoise
BTLA	151888	B and T lymphocyte associated (BTLA), mRNA.	0.02	6.45	7.78E-02	9.89E-02	turquoise
BUB3	9184	BUB3 budding uninhibited by benzimidazoles 3 homolog (yeast) (BUB3), transcript variant 1, mRNA.	0.14	7.52	2.46E-17	9.24E-17	turquoise
C10orf99	387695	chromosome 10 open reading frame 99 (C10orf99), mRNA.	0.01	11.69	5.13E-01	5.58E-01	turquoise
C11orf74	119710	chromosome 11 open reading frame 74 (C11orf74), mRNA.	0.50	8.28	7.09E-54	1.32E-52	turquoise
C12orf45	121053	chromosome 12 open reading frame 45 (C12orf45), mRNA.	-0.08	7.71	8.06E-07	1.65E-06	turquoise
C12orf5	57103	chromosome 12 open reading frame 5 (C12orf5), mRNA.	0.02	8.35	2.48E-01	2.89E-01	turquoise
C12orf75	387882	hypothetical protein (LOC387882), mRNA.	0.08	11.29	1.85E-09	4.50E-09	turquoise
C16orf87	388272	similar to RIKEN cDNA 4921524J17 (LOC388272), mRNA.	0.15	7.52	1.41E-18	5.60E-18	turquoise
C18orf8	29919	chromosome 18 open reading frame 8 (C18orf8), mRNA.	0.12	7.71	7.97E-09	1.87E-08	turquoise
C1GALT1C1	29071	C1GALT1-specific chaperone 1 (C1GALT1C1), transcript variant 1, mRNA.	0.06	5.32	7.57E-04	1.21E-03	turquoise

C1orf54	79630	chromosome 1 open reading frame 54 (C1orf54), mRNA.	0.08	8.29	8.70E-14	2.70E-13	turquoise
C4orf32	132720	chromosome 4 open reading frame 32 (C4orf32), mRNA.	0.11	9.31	1.46E-14	4.73E-14	turquoise
C5orf15	56951	chromosome 5 open reading frame 15 (C5orf15), mRNA.	0.15	9.21	3.66E-16	1.29E-15	turquoise
CA2	760	carbonic anhydrase II (CA2), mRNA.	0.31	8.94	2.76E-45	3.57E-44	turquoise
CABLES1	91768	Cdk5 and Abl enzyme substrate 1 (CABLES1), mRNA.	-0.61	7.70	1.51E-78	1.23E-76	turquoise
CACNA1E	777	calcium channel, voltage-dependent, R type, alpha 1E subunit (CACNA1E), mRNA.	0.25	10.03	8.64E-43	9.99E-42	turquoise
CADM1	23705	cell adhesion molecule 1 (CADM1), mRNA.	0.21	7.39	1.38E-24	7.36E-24	turquoise
CALD1	800	caldesmon 1 (CALD1), transcript variant 5, mRNA.	-0.05	7.24	1.58E-06	3.17E-06	turquoise
CALM1	801	calmodulin 1 (phosphorylase kinase, delta) (CALM1), mRNA.	0.03	5.64	7.40E-02	9.43E-02	turquoise
CAMLG	819	calcium modulating ligand (CAMLG), mRNA.	0.09	10.58	6.13E-10	1.53E-09	turquoise
CAMP	820	cathelicidin antimicrobial peptide (CAMP), mRNA.	0.18	7.20	8.27E-20	3.50E-19	turquoise
CAPG	822	capping protein (actin filament), gelsolin-like (CAPG), mRNA.	0.56	6.90	4.65E-63	1.52E-61	turquoise

CASP4	837	caspase 4, apoptosis-related cysteine peptidase (CASP4), transcript variant alpha, mRNA.	-0.23	7.52	2.14E-26	1.23E-25	turquoise
CASP6	839	caspase 6, apoptosis-related cysteine peptidase (CASP6), transcript variant alpha, mRNA.	-0.30	10.07	3.64E-53	6.41E-52	turquoise
CAST	831	calpastatin (CAST), transcript variant 7, mRNA.	0.23	6.98	1.91E-31	1.31E-30	turquoise
CAV1	857	caveolin 1, caveolae protein, 22kDa (CAV1), mRNA.	0.18	8.36	2.21E-20	9.59E-20	turquoise
CBX2	84733	chromobox homolog 2 (Pc class homolog, Drosophila) (CBX2), transcript variant 1, mRNA.	0.04	9.61	2.56E-02	3.46E-02	turquoise
CBX6	23466	chromobox homolog 6 (CBX6), mRNA.	-0.12	8.37	7.72E-18	2.97E-17	turquoise
CCDC102A	92922	coiled-coil domain containing 102A (CCDC102A), mRNA.	-0.01	7.73	3.63E-01	4.08E-01	turquoise
CCDC109B	55013	coiled-coil domain containing 109B (CCDC109B), mRNA.	0.01	8.28	5.05E-01	5.51E-01	turquoise
CCDC117	150275	coiled-coil domain containing 117 (CCDC117), mRNA.	0.00	11.86	7.75E-01	8.04E-01	turquoise
CCDC28A	25901	coiled-coil domain containing 28A (CCDC28A), mRNA.	-0.01	7.80	4.17E-01	4.63E-01	turquoise
CCDC34	91057	coiled-coil domain containing 34 (CCDC34), transcript variant 1, mRNA.	-0.38	9.15	1.30E-57	3.03E-56	turquoise

CCDC6	8030	coiled-coil domain containing 6 (CCDC6), mRNA.	0.06	6.90	1.97E-04	3.33E-04	turquoise
CCDC71	64925	coiled-coil domain containing 71 (CCDC71), mRNA.	0.05	10.01	1.70E-02	2.35E-02	turquoise
CCL22	6367	chemokine (C-C motif) ligand 22 (CCL22), mRNA.	-0.05	6.23	2.58E-03	3.90E-03	turquoise
CCL3	6348	chemokine (C-C motif) ligand 3 (CCL3), mRNA.	-0.06	7.90	1.08E-07	2.35E-07	turquoise
CCL3L1	728830	PREDICTED: similar to Small inducible cytokine A3-like 1 precursor (Tonsillar lymphocyte LD78 beta protein) (LD78-beta(1-70)) (G0/G1 switch regulatory protein 19-2) (G0S19-2 protein) (PAT 464.2) (LOC728830), mRNA.	-0.04	7.59	3.76E-02	4.99E-02	turquoise
CCL5	6352	chemokine (C-C motif) ligand 5 (CCL5), mRNA.	-0.04	7.04	3.80E-02	5.03E-02	turquoise
CCNA1	8900	cyclin A1 (CCNA1), mRNA.	-0.06	9.39	2.79E-07	5.90E-07	turquoise
CCNDBP1	23582	cyclin D-type binding-protein 1 (CCNDBP1), transcript variant 2, mRNA.	0.07	11.23	8.79E-14	2.72E-13	turquoise
CCT2	10576	chaperonin containing TCP1, subunit 2 (beta) (CCT2), mRNA.	0.18	5.60	4.06E-14	1.29E-13	turquoise
CCT5	22948	chaperonin containing TCP1, subunit 5 (epsilon) (CCT5), mRNA.	0.14	7.87	2.18E-12	6.26E-12	turquoise
CD160	11126	CD160 molecule (CD160), mRNA.	-0.05	10.96	8.40E-05	1.48E-04	turquoise
CD19	930	CD19 molecule (CD19), mRNA.	0.02	9.68	1.22E-01	1.50E-01	turquoise

CD200	4345	CD200 molecule (CD200), transcript variant 1, mRNA.	0.02	11.94	1.78E-01	2.13E-01	turquoise
CD22	933	CD22 molecule (CD22), mRNA.	0.02	11.97	2.54E-01	2.96E-01	turquoise
CD300A	11314	CD300a molecule (CD300A), mRNA.	-0.12	5.66	5.62E-17	2.08E-16	turquoise
CD37	951	CD37 molecule (CD37), transcript variant 2, mRNA.	0.44	7.78	2.37E-55	4.86E-54	turquoise
CD40	958	CD40 molecule, TNF receptor superfamily member 5 (CD40), transcript variant 1, mRNA.	-0.03	7.03	8.27E-02	1.05E-01	turquoise
CD44	960	CD44 molecule (Indian blood group) (CD44), transcript variant 2, mRNA.	0.01	6.79	5.53E-01	5.97E-01	turquoise
CD48	962	CD48 molecule (CD48), mRNA.	-0.04	6.89	1.21E-03	1.90E-03	turquoise
CD53	963	CD53 molecule (CD53), transcript variant 1, mRNA.	-0.47	6.46	3.62E-45	4.65E-44	turquoise
CD59	966	CD59 molecule, complement regulatory protein (CD59), transcript variant 2, mRNA.	-0.06	8.08	1.36E-07	2.94E-07	turquoise
CD70	970	CD70 molecule (CD70), mRNA.	-0.04	9.66	4.12E-03	6.11E-03	turquoise
CD80	941	CD80 molecule (CD80), mRNA.	0.10	6.96	2.30E-09	5.56E-09	turquoise
CD83	9308	CD83 molecule (CD83), transcript variant 2, mRNA.	0.03	7.99	9.51E-02	1.19E-01	turquoise
CD84	8832	CD84 molecule (CD84), mRNA.	0.14	7.16	3.17E-15	1.06E-14	turquoise
CD99L2	83692	CD99 molecule-like 2 (CD99L2), transcript variant 1, mRNA.	-0.10	8.92	1.90E-14	6.11E-14	turquoise
CDC34	997	cell division cycle 34 homolog (S. cerevisiae) (CDC34), mRNA.	-0.13	7.13	2.08E-10	5.36E-10	turquoise

CDC37L1	55664	cell division cycle 37 homolog (S. cerevisiae)-like 1 (CDC37L1), mRNA.	0.53	6.15	1.72E-47	2.49E-46	turquoise
CDK6	1021	cyclin-dependent kinase 6 (CDK6), mRNA.	-0.01	8.14	5.26E-01	5.70E-01	turquoise
CDS2	8760	CDP-diacylglycerol synthase (phosphatidate cytidylyltransferase) 2 (CDS2), mRNA.	-0.08	9.98	2.31E-07	4.92E-07	turquoise
CEBPD	1052	CCAAT/enhancer binding protein (C/EBP), delta (CEBPD), mRNA.	0.03	8.27	1.12E-01	1.39E-01	turquoise
CELF2	10659	CUG triplet repeat, RNA binding protein 2 (CUGBP2), transcript variant 3, mRNA.	0.01	7.07	4.60E-01	5.06E-01	turquoise
CEP70	80321	centrosomal protein 70kDa (CEP70), mRNA.	-0.05	6.85	6.86E-05	1.21E-04	turquoise
CERK	64781	ceramide kinase (CERK), transcript variant 1, mRNA.	-0.04	8.57	1.47E-02	2.04E-02	turquoise
CERS6	253782	LAG1 homolog, ceramide synthase 6 (S. cerevisiae) (LASS6), mRNA.	0.03	7.61	1.53E-02	2.13E-02	turquoise
CH25H	9023	cholesterol 25-hydroxylase (CH25H), mRNA.	-0.07	6.64	6.01E-05	1.07E-04	turquoise
CHD9	80205	chromodomain helicase DNA binding protein 9 (CHD9), mRNA.	0.04	7.15	7.38E-03	1.07E-02	turquoise
CHPT1	56994	choline phosphotransferase 1 (CHPT1), mRNA.	0.07	10.89	1.13E-06	2.29E-06	turquoise
CHST4	10164	carbohydrate (N-acetylglucosamine 6-O) sulfotransferase 4 (CHST4), mRNA.	-0.12	8.20	7.21E-16	2.51E-15	turquoise

CHSY1	22856	carbohydrate (chondroitin) synthase 1 (CHSY1), mRNA.	-0.02	9.84	2.44E-01	2.85E-01	turquoise
CIRH1A	84916	cirrhosis, autosomal recessive 1A (cirhin) (CIRH1A), mRNA.	-0.01	7.37	3.55E-01	4.00E-01	turquoise
CITED2	10370	Cbp/p300-interacting transactivator, with Glu/Asp-rich carboxy-terminal domain, 2 (CITED2), mRNA.	0.25	9.28	1.85E-44	2.30E-43	turquoise
CLASP1	23332	cytoplasmic linker associated protein 1 (CLASP1), mRNA.	-0.10	10.43	1.74E-14	5.60E-14	turquoise
CLC	1178	Charcot-Leyden crystal protein (CLC), mRNA.	-0.42	7.06	3.99E-68	1.63E-66	turquoise
CLDND1	56650	claudin domain containing 1 (CLDND1), transcript variant 2, mRNA.	-0.11	8.84	2.87E-11	7.74E-11	turquoise
CLECL1	160365	dendritic cell-associated lectin-1 (DCAL1), mRNA.	-0.02	8.22	1.22E-01	1.51E-01	turquoise
CLIP2	7461	CAP-GLY domain containing linker protein 2 (CLIP2), transcript variant 1, mRNA.	0.01	6.80	4.22E-01	4.68E-01	turquoise
CMC2	56942	chromosome 16 open reading frame 61 (C16orf61), mRNA.	0.02	6.09	1.25E-01	1.54E-01	turquoise
CMTM6	54918	CKLF-like MARVEL transmembrane domain containing 6 (CMTM6), mRNA.	0.13	8.31	6.50E-16	2.27E-15	turquoise
CNDP2	55748	CNDP dipeptidase 2 (metallopeptidase M20 family) (CNDP2), mRNA.	-0.09	6.50	3.54E-07	7.44E-07	turquoise

CNKSR3	154043	CNKSR family member 3 (CNKSR3), mRNA.	0.04	9.09	4.50E-04	7.40E-04	turquoise
CNNM3	26505	cyclin M3 (CNNM3), transcript variant 1, mRNA.	-0.32	6.82	7.11E-46	9.55E-45	turquoise
CNR1	1268	cannabinoid receptor 1 (brain) (CNR1), transcript variant 1, mRNA.	0.00	10.24	9.09E-01	9.24E-01	turquoise
CNST	163882	chromosome 1 open reading frame 71 (C1orf71), mRNA.	-0.20	8.96	7.96E-35	6.28E-34	turquoise
COCH	1690	coagulation factor C homolog, cochlin (Limulus polyphemus) (COCH), mRNA.	-0.08	10.01	5.29E-11	1.41E-10	turquoise
COL24A1	255631	collagen, type XXIV, alpha 1 (COL24A1), mRNA.	-0.04	6.45	1.27E-02	1.78E-02	turquoise
COQ10B	80219	coenzyme Q10 homolog B (S. cerevisiae) (COQ10B), mRNA.	0.06	6.84	8.42E-04	1.34E-03	turquoise
COQ2	27235	coenzyme Q2 homolog, prenyltransferase (yeast) (COQ2), mRNA.	-0.04	8.00	2.12E-03	3.23E-03	turquoise
CORO1C	23603	coronin, actin binding protein, 1C (CORO1C), mRNA.	0.04	8.39	2.16E-04	3.65E-04	turquoise
COX20	116228	family with sequence similarity 36, member A (FAM36A), mRNA.	0.01	5.58	7.21E-01	7.53E-01	turquoise
CPD	1362	carboxypeptidase D (CPD), mRNA.	-0.15	8.00	1.83E-13	5.60E-13	turquoise
CPNE1	8904	copine I (CPNE1), transcript variant 1, mRNA.	-0.19	10.84	3.79E-34	2.90E-33	turquoise
CPNE8	144402	copine VIII (CPNE8), mRNA.	-0.08	7.80	3.63E-08	8.14E-08	turquoise

CPOX	1371	coproporphyrinogen oxidase (CPOX), mRNA.	0.09	6.11	3.69E-07	7.73E-07	turquoise
CR2	1380	complement component (3d/Epstein Barr virus) receptor 2 (CR2), transcript variant 2, mRNA.	0.14	9.81	9.59E-22	4.44E-21	turquoise
CREB3L2	64764	cAMP responsive element binding protein 3-like 2 (CREB3L2), mRNA.	0.05	9.30	7.35E-04	1.18E-03	turquoise
CRIPT	9419	cysteine-rich PDZ-binding protein (CRIPT), mRNA.	0.00	6.23	8.97E-01	9.13E-01	turquoise
CSK	1445	c-src tyrosine kinase (CSK), mRNA.	0.06	7.04	1.35E-03	2.11E-03	turquoise
CTBS	1486	chitobiase, di-N-acetyl- (CTBS), mRNA.	-0.13	6.22	5.24E-16	1.84E-15	turquoise
CTDSP1	58190	CTD (carboxy-terminal domain, RNA polymerase II, polypeptide A) small phosphatase 1 (CTDSP1), transcript variant 1, mRNA.	-0.08	5.85	7.03E-07	1.45E-06	turquoise
CTHRC1	115908	collagen triple helix repeat containing 1 (CTHRC1), mRNA.	-0.09	5.70	1.52E-07	3.27E-07	turquoise
CTNNAL1	8727	catenin (cadherin-associated protein), alpha-like 1 (CTNNAL1), mRNA.	-0.08	6.66	2.89E-08	6.51E-08	turquoise
CTSZ	1522	cathepsin Z (CTSZ), mRNA.	0.34	7.40	6.84E-40	6.81E-39	turquoise
CWC27	10283	serologically defined colon cancer antigen 10 (SDCCAG10), mRNA.	-0.24	8.01	4.08E-21	1.83E-20	turquoise

CXCL10	3627	chemokine (C-X-C motif) ligand 10 (CXCL10), mRNA.	-0.02	9.83	2.47E-01	2.88E-01	turquoise
CXCL12	6387	chemokine (C-X-C motif) ligand 12 (stromal cell-derived factor 1) (CXCL12), transcript variant 1, mRNA.	-0.02	12.66	2.41E-01	2.81E-01	turquoise
CXCL13	10563	chemokine (C-X-C motif) ligand 13 (B-cell chemoattractant) (CXCL13), mRNA.	-0.06	7.48	6.06E-09	1.44E-08	turquoise
CXCR3	2833	chemokine (C-X-C motif) receptor 3 (CXCR3), mRNA.	-0.01	5.49	3.88E-01	4.33E-01	turquoise
CXCR4	7852	chemokine (C-X-C motif) receptor 4 (CXCR4), transcript variant 2, mRNA.	-0.07	10.89	9.88E-07	2.01E-06	turquoise
CXCR5	643	Burkitt lymphoma receptor 1, GTP binding protein (chemokine (C-X-C motif) receptor 5) (BLR1), transcript variant 1, mRNA.	0.05	7.08	1.78E-03	2.75E-03	turquoise
CXorf57	55086	chromosome X open reading frame 57 (CXorf57), mRNA.	-0.12	6.07	2.20E-13	6.67E-13	turquoise
CXXC4	80319	CXXC finger 4 (CXXC4), mRNA.	-0.04	5.67	8.61E-03	1.24E-02	turquoise
CYB561A3	220002	cytochrome b, ascorbate dependent 3 (CYBASC3), mRNA.	0.01	6.06	4.88E-01	5.33E-01	turquoise
CYBB	1536	cytochrome b-245, beta polypeptide (chronic granulomatous disease) (CYBB), mRNA.	-0.12	7.60	2.28E-23	1.14E-22	turquoise

CYBRD1	79901	cytochrome b reductase 1 (CYBRD1), mRNA.	0.06	10.77	9.21E-11	2.41E-10	turquoise
CYP1B1	1545	cytochrome P450, family 1, subfamily B, polypeptide 1 (CYP1B1), mRNA.	-0.14	9.25	4.19E-17	1.56E-16	turquoise
CYP26A1	1592	cytochrome P450, family 26, subfamily A, polypeptide 1 (CYP26A1), transcript variant 1, mRNA.	0.02	8.89	1.84E-01	2.20E-01	turquoise
CYP2R1	120227	cytochrome P450, family 2, subfamily R, polypeptide 1 (CYP2R1), mRNA.	0.05	8.90	6.84E-03	9.93E-03	turquoise
CYSLTR1	10800	cysteinyl leukotriene receptor 1 (CYSLTR1), mRNA.	-0.08	8.66	1.04E-05	1.97E-05	turquoise
CYTH1	9267	pleckstrin homology, Sec7 and coiled-coil domains 1(cytohesin 1) (PSCD1), transcript variant 1, mRNA.	-0.01	11.91	4.61E-01	5.07E-01	turquoise
CYTIP	9595	pleckstrin homology, Sec7 and coiled-coil domains, binding protein (PSCDBP), mRNA.	-0.07	7.13	2.88E-04	4.82E-04	turquoise
DAAM1	23002	dishevelled associated activator of morphogenesis 1 (DAAM1), mRNA.	-0.02	7.90	1.73E-01	2.08E-01	turquoise
DARS	1615	aspartyl-tRNA synthetase (DARS), mRNA.	0.00	9.54	9.17E-01	9.30E-01	turquoise
DBN1	1627	drebrin 1 (DBN1), transcript variant 2, mRNA.	0.03	10.78	2.30E-02	3.13E-02	turquoise

DCTPP1	79077	XTP3-transactivated protein A (XTP3TPA), mRNA.	0.05	7.16	7.63E-03	1.10E-02	turquoise
DCUN1D5	84259	DCN1, defective in cullin neddylation 1, domain containing 5 (S. cerevisiae) (DCUN1D5), mRNA.	-0.08	7.78	3.67E-07	7.70E-07	turquoise
DDA1	79016	chromosome 19 open reading frame 58 (C19orf58), mRNA.	0.25	7.52	1.10E-16	4.00E-16	turquoise
DDB2	1643	damage-specific DNA binding protein 2, 48kDa (DDB2), mRNA.	0.37	8.33	1.23E-48	1.87E-47	turquoise
DDIT4	54541	DNA-damage-inducible transcript 4 (DDIT4), mRNA.	-0.40	6.60	7.73E-47	1.09E-45	turquoise
DDR2	4921	discoidin domain receptor family, member 2 (DDR2), transcript variant 2, mRNA.	0.08	5.80	1.62E-06	3.25E-06	turquoise
DDX1	1653	DEAD (Asp-Glu-Ala-Asp) box polypeptide 1 (DDX1), mRNA.	0.04	5.56	1.21E-02	1.71E-02	turquoise
DDX18	8886	DEAD (Asp-Glu-Ala-Asp) box polypeptide 18 (DDX18), mRNA.	0.05	8.75	1.96E-03	3.02E-03	turquoise
DDX5	1655	DEAD (Asp-Glu-Ala-Asp) box polypeptide 5 (DDX5), mRNA.	-0.02	11.59	6.17E-01	6.58E-01	turquoise
DDX59	83479	DEAD (Asp-Glu-Ala-Asp) box polypeptide 59 (DDX59), transcript variant 1, mRNA.	-0.38	7.80	3.40E-41	3.59E-40	turquoise

DENND1B	54530	hypothetical protein FLJ20054 (FLJ20054), mRNA.	0.02	5.45	2.09E-01	2.47E-01	turquoise
DENND5B	160518	hypothetical protein MGC24039 (MGC24039), mRNA.	0.01	8.74	2.44E-01	2.85E-01	turquoise
DERA	51071	2-deoxyribose-5-phosphate aldolase homolog (C. elegans) (DERA), mRNA.	0.00	8.25	8.51E-01	8.73E-01	turquoise
DGCR8	54487	DiGeorge syndrome critical region gene 8 (DGCR8), mRNA.	-0.12	7.44	7.76E-17	2.85E-16	turquoise
DGKD	8527	diacylglycerol kinase, delta 130kDa (DGKD), transcript variant 1, mRNA.	-0.09	7.96	1.02E-02	1.45E-02	turquoise
DHCR24	1718	24-dehydrocholesterol reductase (DHCR24), mRNA.	-0.08	7.15	1.91E-07	4.09E-07	turquoise
DHRS3	9249	dehydrogenase/reductase (SDR family) member 3 (DHRS3), mRNA.	0.05	7.46	9.79E-04	1.55E-03	turquoise
DIMT1	27292	DIM1 dimethyladenosine transferase 1-like (S. cerevisiae) (DIMT1L), mRNA.	-0.01	8.67	5.49E-01	5.93E-01	turquoise
DNAJA1	3301	DnaJ (Hsp40) homolog, subfamily A, member 1 (DNAJA1), mRNA.	0.32	7.12	6.67E-32	4.69E-31	turquoise
DNAJB6	387820	PREDICTED: similar to DnaJ (Hsp40) homolog, subfamily B, member 6 isoform a (LOC387820), mRNA.	-0.16	7.52	1.38E-18	5.51E-18	turquoise

DNAJC5B	85479	DnaJ (Hsp40) homolog, subfamily C, member 5 beta (DNAJC5B), mRNA.	-0.04	6.66	2.09E-02	2.85E-02	turquoise
DNASE1L3	1776	deoxyribonuclease I-like 3 (DNASE1L3), mRNA.	-0.03	9.53	6.49E-03	9.44E-03	turquoise
DOCK7	85440	dedicator of cytokinesis 7 (DOCK7), mRNA.	0.17	11.51	2.98E-26	1.70E-25	turquoise
DPYSL2	1808	dihydropyrimidinase-like 2 (DPYSL2), mRNA.	-0.18	9.35	7.00E-32	4.92E-31	turquoise
DRAM1	55332	damage-regulated autophagy modulator (DRAM), mRNA.	-0.23	10.40	6.15E-36	5.14E-35	turquoise
DSE	29940	dermatan sulfate epimerase (DSE), transcript variant 2, mRNA.	-0.09	8.04	2.31E-12	6.61E-12	turquoise
DSTN	11034	destrin (actin depolymerizing factor) (DSTN), transcript variant 2, mRNA.	0.11	7.13	2.29E-12	6.54E-12	turquoise
DTL	51514	denticleless homolog (Drosophila) (DTL), mRNA.	0.05	7.89	1.60E-04	2.73E-04	turquoise
DUSP11	8446	dual specificity phosphatase 11 (RNA/RNP complex 1-interacting) (DUSP11), mRNA.	0.05	8.46	4.79E-05	8.57E-05	turquoise
DUSP2	1844	dual specificity phosphatase 2 (DUSP2), mRNA.	-0.09	9.18	1.91E-10	4.91E-10	turquoise
DUSP22	56940	dual specificity phosphatase 22 (DUSP22), mRNA.	-0.36	8.44	1.52E-32	1.10E-31	turquoise
DUSP4	1846	dual specificity phosphatase 4 (DUSP4), transcript variant 1, mRNA.	-0.06	9.70	7.16E-06	1.37E-05	turquoise

DYNC1H1	1778	dynein, cytoplasmic 1, heavy chain 1 (DYNC1H1), mRNA.	-0.01	8.22	7.18E-01	7.51E-01	turquoise
DYNLL1	8655	dynein, cytoplasmic, light polypeptide 1 (DNCL1), mRNA.	0.02	7.81	3.20E-01	3.65E-01	turquoise
DYRK2	8445	dual-specificity tyrosine-(Y)-phosphorylation regulated kinase 2 (DYRK2), transcript variant 2, mRNA.	0.01	12.29	3.09E-01	3.53E-01	turquoise
DYRK3	8444	dual-specificity tyrosine-(Y)-phosphorylation regulated kinase 3 (DYRK3), transcript variant 2, mRNA.	0.05	10.84	3.07E-03	4.61E-03	turquoise
E2F2	1870	E2F transcription factor 2 (E2F2), mRNA.	-0.02	9.80	6.55E-02	8.42E-02	turquoise
E2F5	1875	E2F transcription factor 5, p130-binding (E2F5), mRNA.	-0.02	11.61	1.20E-01	1.48E-01	turquoise
E2F7	144455	E2F transcription factor 7 (E2F7), mRNA.	-0.01	7.30	3.96E-01	4.42E-01	turquoise
EBF1	1879	early B-cell factor 1 (EBF1), mRNA.	-0.02	7.88	7.98E-02	1.01E-01	turquoise
ECHS1	1892	enoyl Coenzyme A hydratase, short chain, 1, mitochondrial (ECHS1), nuclear gene encoding mitochondrial protein, mRNA.	0.09	7.11	5.46E-09	1.30E-08	turquoise
EDEM1	9695	ER degradation enhancer, mannosidase alpha-like 1 (EDEM1), mRNA.	-0.09	8.81	2.22E-10	5.71E-10	turquoise

EDN1	1906	endothelin 1 (EDN1), mRNA.	0.18	10.86	5.17E-23	2.54E-22	turquoise
EED	8726	embryonic ectoderm development (EED), transcript variant 2, mRNA.	-0.11	7.60	2.61E-11	7.06E-11	turquoise
EEF1A2	1917	eukaryotic translation elongation factor 1 alpha 2 (EEF1A2), mRNA.	0.00	7.31	8.44E-01	8.66E-01	turquoise
EEF1E1	9521	eukaryotic translation elongation factor 1 epsilon 1 (EEF1E1), mRNA.	-0.10	7.56	1.59E-07	3.42E-07	turquoise
EFR3A	23167	KIAA0143 protein (KIAA0143), mRNA.	-0.21	9.50	7.45E-42	8.17E-41	turquoise
EGFL6	25975	EGF-like-domain, multiple 6 (EGFL6), mRNA.	0.09	7.53	1.71E-09	4.16E-09	turquoise
EGR2	1959	early growth response 2 (Krox-20 homolog, Drosophila) (EGR2), mRNA.	-0.04	8.28	1.04E-04	1.81E-04	turquoise
EHBP1	23301	EH domain binding protein 1 (EHBP1), mRNA.	-0.01	7.77	5.49E-01	5.93E-01	turquoise
EHD4	30844	EH-domain containing 4 (EHD4), mRNA.	0.12	8.20	1.32E-08	3.04E-08	turquoise
ELL3	80237	elongation factor RNA polymerase II-like 3 (ELL3), mRNA.	-0.04	6.44	2.72E-02	3.67E-02	turquoise
ELOVL6	79071	ELOVL family member 6, elongation of long chain fatty acids (FEN1/Elo2, SUR4/Elo3-like, yeast) (ELOVL6), mRNA.	0.12	11.05	1.76E-14	5.67E-14	turquoise

ELP3	55140	elongation protein 3 homolog (S. cerevisiae) (ELP3), mRNA.	0.05	9.63	1.28E-04	2.21E-04	turquoise
EMG1	10436	EMG1 nucleolar protein homolog (S. cerevisiae) (EMG1), mRNA.	-0.01	5.51	3.68E-01	4.14E-01	turquoise
ENC1	8507	ectodermal-neural cortex (with BTB-like domain) (ENC1), mRNA.	0.02	10.62	8.13E-02	1.03E-01	turquoise
ENPP2	5168	ectonucleotide pyrophosphatase/phosphodiesterase 2 (autotaxin) (ENPP2), transcript variant 1, mRNA.	0.03	8.03	9.75E-02	1.22E-01	turquoise
ENPP4	22875	ectonucleotide pyrophosphatase/phosphodiesterase 4 (putative function) (ENPP4), mRNA.	-0.14	9.06	6.49E-17	2.39E-16	turquoise
ENPP5	59084	ectonucleotide pyrophosphatase/phosphodiesterase 5 (putative function) (ENPP5), mRNA.	0.21	7.48	3.99E-49	6.12E-48	turquoise
ENTPD1	953	ectonucleoside triphosphate diphosphohydrolase 1 (ENTPD1), mRNA.	-0.03	8.84	3.65E-02	4.84E-02	turquoise
EPAS1	2034	endothelial PAS domain protein 1 (EPAS1), mRNA.	-0.04	5.91	3.81E-03	5.66E-03	turquoise
EPB41L2	2037	erythrocyte membrane protein band 4.1-like 2 (EPB41L2), mRNA.	-0.01	8.24	6.19E-01	6.59E-01	turquoise
EPS8	2059	epidermal growth factor receptor pathway substrate 8 (EPS8), mRNA.	0.11	6.74	4.92E-06	9.57E-06	turquoise

ERAP2	64167	leukocyte-derived arginine aminopeptidase (LRAP), mRNA.	-0.07	7.94	1.04E-04	1.81E-04	turquoise
ERCC6L	54821	FLJ20105 protein (FLJ20105), transcript variant 2, mRNA.	0.02	6.28	2.34E-01	2.74E-01	turquoise
ERMP1	79956	KIAA1815 (KIAA1815), mRNA.	-0.05	7.03	8.01E-04	1.28E-03	turquoise
ETS1	2113	v-ets erythroblastosis virus E26 oncogene homolog 1 (avian) (ETS1), mRNA.	-0.18	6.79	2.19E-20	9.55E-20	turquoise
ETV4	2118	ets variant gene 4 (E1A enhancer binding protein, E1AF) (ETV4), transcript variant 2, mRNA.	-0.08	7.99	1.82E-08	4.17E-08	turquoise
EVL	51466	Enah/Vasp-like (EVL), mRNA.	0.07	7.18	1.75E-06	3.51E-06	turquoise
EZR	7430	villin 2 (ezrin) (VIL2), mRNA.	0.01	5.78	3.45E-01	3.90E-01	turquoise
F13A1	2162	coagulation factor XIII, A1 polypeptide (F13A1), mRNA.	-0.09	6.86	3.07E-09	7.37E-09	turquoise
FAIM	55179	Fas apoptotic inhibitory molecule (FAIM), transcript variant 1, mRNA.	0.01	8.46	4.68E-01	5.13E-01	turquoise
FAM129C	199786	family with sequence similarity 129, member C (FAM129C), mRNA.	0.21	9.23	1.15E-42	1.33E-41	turquoise
FAM133B	257415	hypothetical protein MGC40405 (MGC40405), transcript variant 2, mRNA.	-0.03	7.14	6.93E-02	8.87E-02	turquoise
FAM160B1	57700	KIAA1600 (KIAA1600), mRNA.	0.04	10.97	1.11E-03	1.75E-03	turquoise

FAM174B	400451	hypothetical gene supported by AK075564; BC060873 (LOC400451), mRNA.	-0.49	8.79	1.02E-80	9.59E-79	turquoise
FAM20B	9917	family with sequence similarity 20, member B (FAM20B), mRNA.	0.00	9.00	8.47E-01	8.69E-01	turquoise
FAM216A	29902	chromosome 12 open reading frame 24 (C12orf24), mRNA.	-0.04	6.92	1.28E-03	2.00E-03	turquoise
FAM57A	79850	family with sequence similarity 57, member A (FAM57A), mRNA.	-0.20	7.01	1.23E-30	8.12E-30	turquoise
FAM60A	58516	family with sequence similarity 60, member A (FAM60A), mRNA.	0.25	7.41	1.79E-22	8.54E-22	turquoise
FAM81A	145773	family with sequence similarity 81, member A (FAM81A), mRNA.	0.12	7.78	7.97E-15	2.62E-14	turquoise
FAM89B	23625	family with sequence similarity 89, member B (FAM89B), mRNA.	0.01	6.87	5.80E-01	6.23E-01	turquoise
FANCD2	2177	Fanconi anemia, complementation group D2 (FANCD2), transcript variant 2, mRNA.	0.00	12.31	8.07E-01	8.32E-01	turquoise
FAR2	55711	male sterility domain containing 1 (MLSTD1), mRNA.	-0.14	5.76	1.25E-16	4.54E-16	turquoise
FASN	2194	fatty acid synthase (FASN), mRNA.	0.02	8.47	1.39E-01	1.69E-01	turquoise
FBXO32	114907	F-box protein 32 (FBXO32), transcript variant 1, mRNA.	0.14	5.71	5.17E-13	1.54E-12	turquoise
FBXO33	254170	F-box protein 33 (FBXO33), mRNA.	-0.34	7.15	9.78E-42	1.06E-40	turquoise

FBXW7	55294	F-box and WD repeat domain containing 7 (FBXW7), transcript variant 2, mRNA.	0.63	5.85	1.29E-62	4.08E-61	turquoise
FCER2	2208	Fc fragment of IgE, low affinity II, receptor for (CD23) (FCER2), mRNA.	-0.41	6.36	2.03E-45	2.66E-44	turquoise
FCGR2B	2213	Fc fragment of IgG, low affinity IIb, receptor (CD32) (FCGR2B), transcript variant 3, mRNA.	-0.34	7.93	4.91E-51	7.99E-50	turquoise
FCHSD2	9873	FCH and double SH3 domains 2 (FCHSD2), mRNA.	0.00	7.63	9.94E-01	9.95E-01	turquoise
FCRLB	127943	Fc receptor-like B (FCRLB), mRNA.	-0.02	8.49	1.11E-01	1.38E-01	turquoise
FGR	2268	Gardner-Rasheed feline sarcoma viral (v-fgr) oncogene homolog (FGR), transcript variant 1, mRNA.	0.02	6.98	3.18E-01	3.62E-01	turquoise
FHL2	2274	four and a half LIM domains 2 (FHL2), transcript variant 4, mRNA.	-0.30	6.97	1.05E-36	9.19E-36	turquoise
FILIP1L	11259	downregulated in ovarian cancer 1 (DOC1), transcript variant 1, mRNA.	0.06	9.65	2.02E-06	4.03E-06	turquoise
FNBP1	23048	formin binding protein 1 (FNBP1), mRNA.	-0.01	7.60	3.70E-01	4.16E-01	turquoise
FOXN2	3344	forkhead box N2 (FOXN2), mRNA.	-0.08	9.46	1.39E-05	2.62E-05	turquoise
FRAT2	23401	frequently rearranged in advanced T-cell lymphomas 2 (FRAT2), mRNA.	-0.02	6.72	1.87E-01	2.24E-01	turquoise

FRMD3	257019	FERM domain containing 3 (FRMD3), mRNA.	0.17	8.16	4.68E-15	1.55E-14	turquoise
FSTL3	10272	follistatin-like 3 (secreted glycoprotein) (FSTL3), mRNA.	-0.07	6.40	1.88E-06	3.76E-06	turquoise
FUCA1	2517	fucosidase, alpha-L- 1, tissue (FUCA1), mRNA.	-0.04	8.09	1.07E-03	1.69E-03	turquoise
FUNDC1	139341	FUN14 domain containing 1 (FUNDC1), mRNA.	-0.13	7.82	8.51E-07	1.74E-06	turquoise
FUT7	2529	fucosyltransferase 7 (alpha (1,3) fucosyltransferase) (FUT7), mRNA.	0.11	6.50	8.79E-10	2.18E-09	turquoise
GABARAPL2	11345	GABA(A) receptor-associated protein-like 2 (GABARAPL2), mRNA.	0.01	9.22	3.58E-01	4.03E-01	turquoise
GADD45B	4616	growth arrest and DNA-damage-inducible, beta (GADD45B), mRNA.	0.09	10.35	7.34E-09	1.72E-08	turquoise
GALK1	2584	galactokinase 1 (GALK1), mRNA.	-0.01	10.41	5.42E-01	5.86E-01	turquoise
GAS7	8522	growth arrest-specific 7 (GAS7), transcript variant c, mRNA.	-0.07	5.56	2.18E-04	3.68E-04	turquoise
GCH1	2643	GTP cyclohydrolase 1 (dopa-responsive dystonia) (GCH1), transcript variant 2, mRNA.	0.00	9.10	8.81E-01	8.99E-01	turquoise
GCLC	2729	glutamate-cysteine ligase, catalytic subunit (GCLC), mRNA.	0.08	8.01	1.64E-06	3.29E-06	turquoise

GCNT2	2651	glucosaminyl (N-acetyl) transferase 2, I-branching enzyme (I blood group) (GCNT2), transcript variant 1, mRNA.	-0.16	9.00	1.75E-24	9.27E-24	turquoise
GFM1	85476	G elongation factor, mitochondrial 1 (GFM1), nuclear gene encoding mitochondrial protein, mRNA.	0.36	8.28	3.32E-57	7.59E-56	turquoise
GGA2	23062	golgi associated, gamma adaptin ear containing, ARF binding protein 2 (GGA2), mRNA.	-0.09	6.20	3.68E-09	8.81E-09	turquoise
GINS2	51659	GINS complex subunit 2 (Psf2 homolog) (GINS2), mRNA.	0.00	12.17	7.69E-01	7.98E-01	turquoise
GINS3	64785	GINS complex subunit 3 (Psf3 homolog) (GINS3), mRNA.	0.05	6.70	1.41E-03	2.20E-03	turquoise
GINS4	84296	GINS complex subunit 4 (Sld5 homolog) (GINS4), mRNA.	0.01	7.59	6.76E-01	7.11E-01	turquoise
GLIPR2	152007	chromosome 9 open reading frame 19 (C9orf19), mRNA.	0.68	9.75	5.13E-83	5.43E-81	turquoise
GLMN	11146	glomulin, FKBP associated protein (GLMN), mRNA.	0.07	9.57	4.06E-05	7.32E-05	turquoise
GLO1	2739	glyoxalase I (GLO1), mRNA.	-0.13	5.70	8.64E-14	2.68E-13	turquoise
GLUD1	2746	glutamate dehydrogenase 1 (GLUD1), mRNA.	0.11	7.78	3.97E-10	1.01E-09	turquoise
GNA11	2767	guanine nucleotide binding protein (G protein), alpha 11 (Gq class) (GNA11), mRNA.	0.05	8.59	4.38E-05	7.85E-05	turquoise

GNE	10020	glucosamine (UDP-N-acetyl)-2-epimerase/N-acetylmannosamine kinase (GNE), mRNA.	0.40	7.52	1.82E-42	2.07E-41	turquoise
GNG7	2788	guanine nucleotide binding protein (G protein), gamma 7 (GNG7), mRNA.	-0.06	7.98	6.96E-07	1.43E-06	turquoise
GNL2	29889	guanine nucleotide binding protein-like 2 (nucleolar) (GNL2), mRNA.	0.05	8.92	5.16E-03	7.58E-03	turquoise
GOLGA7	51125	golgi autoantigen, golgin subfamily a, 7 (GOLGA7), transcript variant 2, mRNA.	-0.03	7.54	7.93E-02	1.01E-01	turquoise
GOT2	2806	glutamic-oxaloacetic transaminase 2, mitochondrial (aspartate aminotransferase 2) (GOT2), nuclear gene encoding mitochondrial protein, mRNA.	-0.01	10.59	4.85E-01	5.30E-01	turquoise
GPR132	29933	G protein-coupled receptor 132 (GPR132), mRNA.	0.00	8.19	8.29E-01	8.52E-01	turquoise
GPR137B	7107	G protein-coupled receptor 137B (GPR137B), mRNA.	-0.02	7.11	3.11E-01	3.55E-01	turquoise
GPR55	9290	G protein-coupled receptor 55 (GPR55), mRNA.	0.10	7.98	5.76E-08	1.28E-07	turquoise
GPT2	84706	glutamic pyruvate transaminase (alanine aminotransferase) 2 (GPT2), mRNA.	-0.06	7.08	6.32E-04	1.02E-03	turquoise
GRAMD3	65983	GRAM domain containing 3 (GRAMD3), mRNA.	-0.06	6.38	6.45E-06	1.24E-05	turquoise

GRAMD4	23151	death-inducing-protein (DIP), mRNA.	-0.09	9.29	6.24E-08	1.38E-07	turquoise
GSK3B	2932	glycogen synthase kinase 3 beta (GSK3B), mRNA.	0.03	8.74	8.75E-02	1.11E-01	turquoise
GTF2E2	2961	general transcription factor IIE, polypeptide 2, beta 34kDa (GTF2E2), mRNA.	-0.28	6.82	1.28E-41	1.39E-40	turquoise
GTF2F2	2963	general transcription factor IIF, polypeptide 2 (30kD subunit) (GTF2F2), mRNA.	-0.05	7.01	1.94E-04	3.29E-04	turquoise
GTPBP4	23560	GTP binding protein 4 (GTPBP4), mRNA.	-0.07	6.01	9.29E-05	1.62E-04	turquoise
GUSB	2990	glucuronidase, beta (GUSB), mRNA.	-0.28	7.00	3.35E-35	2.71E-34	turquoise
H1FX	8971	H1 histone family, member X (H1FX), mRNA.	-0.03	7.20	4.33E-02	5.70E-02	turquoise
H2AFZ	3015	H2A histone family, member Z (H2AFZ), mRNA.	-0.10	8.41	6.50E-12	1.82E-11	turquoise
HACD3	51495	protein tyrosine phosphatase-like A domain containing 1 (PTPLAD1), mRNA.	-0.05	10.29	1.03E-04	1.79E-04	turquoise
HAGHL	84264	hydroxyacylglutathione hydrolase-like (HAGHL), transcript variant 2, mRNA.	-0.02	9.80	2.75E-01	3.17E-01	turquoise
HCFC1R1	54985	host cell factor C1 regulator 1 (XPO1 dependent) (HCFC1R1), transcript variant 1, mRNA.	0.03	9.12	2.57E-03	3.89E-03	turquoise
HDAC9	9734	histone deacetylase 9 (HDAC9), transcript variant 3, mRNA.	0.02	11.10	1.01E-01	1.26E-01	turquoise

HERC1	8925	hect (homologous to the E6-AP (UBE3A) carboxyl terminus) domain and RCC1 (CHC1)-like domain (RLD) 1 (HERC1), mRNA.	0.03	8.54	1.61E-01	1.94E-01	turquoise
HILPDA	29923	hypoxia-inducible protein 2 (HIG2), mRNA.	-0.39	6.12	3.93E-51	6.46E-50	turquoise
HIPK2	28996	homeodomain interacting protein kinase 2 (HIPK2), mRNA.	-0.13	8.76	1.59E-20	6.96E-20	turquoise
HIVEP1	3096	human immunodeficiency virus type I enhancer binding protein 1 (HIVEP1), mRNA.	-0.08	10.33	1.04E-05	1.98E-05	turquoise
HK2	3099	hexokinase 2 (HK2), mRNA.	0.10	7.74	6.17E-08	1.36E-07	turquoise
HLA-DMB	3109	major histocompatibility complex, class II, DM beta (HLA-DMB), mRNA.	0.05	8.47	5.69E-03	8.32E-03	turquoise
HLA-DOB	3112	major histocompatibility complex, class II, DO beta (HLA-DOB), mRNA.	0.14	6.30	1.70E-14	5.49E-14	turquoise
HMCES	56941	chromosome 3 open reading frame 37 (C3orf37), transcript variant 2, mRNA.	0.00	6.34	9.86E-01	9.88E-01	turquoise
HMGCS1	3157	3-hydroxy-3-methylglutaryl-Coenzyme A synthase 1 (soluble) (HMGCS1), mRNA.	-0.01	9.67	3.77E-01	4.22E-01	turquoise
HMOX1	3162	heme oxygenase (decycling) 1 (HMOX1), mRNA.	-0.01	8.90	4.55E-01	5.01E-01	turquoise

HPCAL1	3241	hippocalcin-like 1 (HPCAL1), transcript variant 2, mRNA.	0.05	6.44	9.60E-03	1.37E-02	turquoise
HPRT1	3251	hypoxanthine phosphoribosyltransferase 1 (Lesch-Nyhan syndrome) (HPRT1), mRNA.	0.06	11.24	1.64E-05	3.06E-05	turquoise
HSD17B12	51144	hydroxysteroid (17-beta) dehydrogenase 12 (HSD17B12), mRNA.	-0.10	8.25	8.46E-14	2.63E-13	turquoise
HSD17B4	3295	hydroxysteroid (17-beta) dehydrogenase 4 (HSD17B4), mRNA.	-0.17	7.32	9.39E-31	6.23E-30	turquoise
HSPA4L	22824	heat shock 70kDa protein 4-like (HSPA4L), mRNA.	0.06	7.90	3.70E-06	7.25E-06	turquoise
HSPH1	10808	heat shock 105kDa/110kDa protein 1 (HSPH1), mRNA.	-0.08	7.47	1.84E-10	4.75E-10	turquoise
ICOSLG	23308	inducible T-cell co-stimulator ligand (ICOSLG), mRNA.	-0.15	9.07	1.50E-28	9.28E-28	turquoise
ID2	3398	inhibitor of DNA binding 2, dominant negative helix-loop-helix protein (ID2), mRNA.	0.01	6.88	2.94E-01	3.38E-01	turquoise
ID3	3399	inhibitor of DNA binding 3, dominant negative helix-loop-helix protein (ID3), mRNA.	-0.07	8.31	3.51E-06	6.91E-06	turquoise
IER3	8870	immediate early response 3 (IER3), mRNA.	-0.21	8.21	4.56E-23	2.25E-22	turquoise
IER5	51278	immediate early response 5 (IER5), mRNA.	-0.08	7.22	2.82E-08	6.36E-08	turquoise
IFI16	3428	interferon, gamma-inducible protein 16 (IFI16), mRNA.	0.01	8.00	5.53E-01	5.97E-01	turquoise

IFI30	10437	interferon, gamma-inducible protein 30 (IFI30), mRNA.	-0.17	9.34	7.72E-23	3.79E-22	turquoise
IFNAR2	3455	interferon (alpha, beta and omega) receptor 2 (IFNAR2), transcript variant 2, mRNA.	-0.04	9.86	1.01E-02	1.44E-02	turquoise
IGFLR1	79713	transmembrane protein 149 (TMEM149), mRNA.	0.02	8.39	1.63E-01	1.97E-01	turquoise
IL12A	3592	interleukin 12A (natural killer cell stimulatory factor 1, cytotoxic lymphocyte maturation factor 1, p35) (IL12A), mRNA.	-0.18	7.66	1.28E-33	9.61E-33	turquoise
IL15	3600	interleukin 15 (IL15), transcript variant 3, mRNA.	0.07	9.02	8.43E-08	1.85E-07	turquoise
IL1A	3552	interleukin 1, alpha (IL1A), mRNA.	-0.37	8.57	5.17E-55	1.03E-53	turquoise
IL1B	3553	interleukin 1, beta (IL1B), mRNA.	-0.04	8.65	5.03E-02	6.55E-02	turquoise
IL21R	50615	interleukin 21 receptor (IL21R), transcript variant 2, mRNA.	0.01	10.18	3.94E-01	4.40E-01	turquoise
IL4I1	259307	interleukin 4 induced 1 (IL4I1), transcript variant 1, mRNA.	0.38	7.12	2.94E-51	4.85E-50	turquoise
IMPA2	3613	inositol(myo)-1(or 4)-monophosphatase 2 (IMPA2), mRNA.	0.09	9.23	5.77E-07	1.19E-06	turquoise
INF2	64423	chromosome 14 open reading frame 173 (C14orf173), transcript variant 1, mRNA.	-0.06	8.53	4.44E-07	9.25E-07	turquoise
ING2	3622	inhibitor of growth family, member 2 (ING2), mRNA.	-0.16	8.97	1.08E-33	8.11E-33	turquoise

INIP	58493	chromosome 9 open reading frame 80 (C9orf80), mRNA.	-0.18	6.32	8.77E-20	3.71E-19	turquoise
INPP1	3628	inositol polyphosphate-1-phosphatase (INPP1), mRNA.	-0.26	6.85	8.19E-38	7.59E-37	turquoise
INSIG1	3638	insulin induced gene 1 (INSIG1), transcript variant 2, mRNA.	-0.15	5.73	8.65E-21	3.83E-20	turquoise
IQCB1	9657	IQ motif containing B1 (IQCB1), transcript variant 3, mRNA.	0.08	7.73	1.68E-04	2.86E-04	turquoise
IQGAP1	8826	IQ motif containing GTPase activating protein 1 (IQGAP1), mRNA.	0.01	9.22	5.74E-01	6.18E-01	turquoise
IQGAP2	10788	IQ motif containing GTPase activating protein 2 (IQGAP2), mRNA.	0.03	12.12	2.11E-02	2.89E-02	turquoise
IQGAP3	128239	IQ motif containing GTPase activating protein 3 (IQGAP3), mRNA.	0.00	8.99	9.11E-01	9.25E-01	turquoise
IRAK3	11213	interleukin-1 receptor-associated kinase 3 (IRAK3), mRNA.	0.31	6.22	1.07E-46	1.49E-45	turquoise
IRF1	3659	interferon regulatory factor 1 (IRF1), mRNA.	-0.08	10.74	9.44E-12	2.62E-11	turquoise
IRF2BP2	359948	interferon regulatory factor 2 binding protein 2 (IRF2BP2), transcript variant 2, mRNA.	0.24	7.66	5.22E-34	3.98E-33	turquoise
ISG20	3669	interferon stimulated exonuclease gene 20kDa (ISG20), mRNA.	-0.37	6.47	2.10E-51	3.49E-50	turquoise

ITGAE	3682	integrin, alpha E (antigen CD103, human mucosal lymphocyte antigen 1; alpha polypeptide) (ITGAE), mRNA.	-0.18	7.60	3.17E-22	1.50E-21	turquoise
ITGB7	3695	integrin, beta 7 (ITGB7), mRNA.	0.33	6.05	2.66E-45	3.46E-44	turquoise
ITPKA	3706	inositol 1,4,5-trisphosphate 3-kinase A (ITPKA), mRNA.	0.03	6.67	5.86E-02	7.57E-02	turquoise
ITPR1	3708	inositol 1,4,5-triphosphate receptor, type 1 (ITPR1), mRNA.	-0.02	9.80	2.15E-01	2.54E-01	turquoise
ITPR2	3709	inositol 1,4,5-triphosphate receptor, type 2 (ITPR2), mRNA.	0.07	10.53	1.25E-05	2.36E-05	turquoise
JADE3	9767	PHD finger protein 16 (PHF16), transcript variant 2, mRNA.	-0.02	5.50	2.24E-01	2.63E-01	turquoise
JAM3	83700	junctional adhesion molecule 3 (JAM3), mRNA.	-0.04	10.36	2.17E-02	2.96E-02	turquoise
JARID2	3720	jumonji, AT rich interactive domain 2 (JARID2), mRNA.	0.04	5.97	9.02E-03	1.29E-02	turquoise
JDP2	122953	jun dimerization protein 2 (JDP2), mRNA.	0.05	7.42	3.10E-04	5.17E-04	turquoise
JKAMP	51528	chromosome 14 open reading frame 100 (C14orf100), mRNA.	-0.11	6.61	1.47E-14	4.77E-14	turquoise
JMJD1C	221037	jumonji domain containing 1C (JMJD1C), transcript variant 2, mRNA.	0.05	9.73	9.73E-03	1.38E-02	turquoise

KANK1	23189	ankyrin repeat domain 15 (ANKRD15), transcript variant 1, mRNA.	-0.03	8.44	3.92E-02	5.19E-02	turquoise
KCNK1	3775	potassium channel, subfamily K, member 1 (KCNK1), mRNA.	-0.08	7.77	7.17E-09	1.69E-08	turquoise
KCNN4	3783	potassium intermediate/small conductance calcium-activated channel, subfamily N, member 4 (KCNN4), mRNA.	-0.09	5.77	2.57E-07	5.45E-07	turquoise
KCTD12	115207	potassium channel tetramerisation domain containing 12 (KCTD12), mRNA.	-0.10	7.07	8.64E-05	1.51E-04	turquoise
KCTD13	253980	potassium channel tetramerisation domain containing 13 (KCTD13), mRNA.	-0.02	10.16	1.71E-01	2.06E-01	turquoise
KDM5B	10765	jumonji, AT rich interactive domain 1B (JARID1B), mRNA.	0.04	10.12	1.80E-03	2.78E-03	turquoise
KIAA0020	9933	KIAA0020 (KIAA0020), mRNA.	-0.27	8.65	5.14E-41	5.39E-40	turquoise
KIAA0513	9764	KIAA0513 (KIAA0513), mRNA.	0.15	7.50	2.86E-17	1.07E-16	turquoise
KIAA1324L	222223	KIAA1324-like (KIAA1324L), mRNA.	0.51	9.89	4.57E-84	5.65E-82	turquoise
KIF13B	23303	kinesin family member 13B (KIF13B), mRNA.	0.20	7.74	4.66E-21	2.08E-20	turquoise
KLF13	51621	Kruppel-like factor 13 (KLF13), mRNA.	-0.19	10.73	9.78E-25	5.26E-24	turquoise
KLF2	10365	Kruppel-like factor 2 (lung) (KLF2), mRNA.	-0.08	7.33	1.21E-05	2.29E-05	turquoise

KLF6	1316	Kruppel-like factor 6 (KLF6), transcript variant 2, mRNA.	0.16	8.02	1.49E-13	4.56E-13	turquoise
KLF9	687	Kruppel-like factor 9 (KLF9), mRNA.	-0.02	8.09	7.77E-02	9.87E-02	turquoise
KLHDC2	23588	kelch domain containing 2 (KLHDC2), mRNA.	-0.09	6.98	6.62E-05	1.17E-04	turquoise
KLHL9	55958	kelch-like 9 (Drosophila) (KLHL9), mRNA.	-0.31	7.82	2.95E-42	3.34E-41	turquoise
LACTB2	51110	lactamase, beta 2 (LACTB2), mRNA.	0.52	6.23	1.03E-49	1.59E-48	turquoise
LAMP3	27074	lysosomal-associated membrane protein 3 (LAMP3), mRNA.	-0.09	8.40	1.17E-08	2.73E-08	turquoise
LAPTM5	7805	lysosomal associated multispanning membrane protein 5 (LAPTM5), mRNA.	0.02	8.97	3.12E-02	4.16E-02	turquoise
LARP1B	55132	La ribonucleoprotein domain family, member 2 (LARP2), transcript variant 3, mRNA.	-0.07	6.58	4.97E-06	9.67E-06	turquoise
LASP1	3927	LIM and SH3 protein 1 (LASP1), mRNA.	0.27	8.80	3.72E-40	3.78E-39	turquoise
LAT2	7462	linker for activation of T cells family, member 2 (LAT2), transcript variant 3, mRNA.	-0.18	5.49	5.91E-19	2.41E-18	turquoise
LCK	3932	lymphocyte-specific protein tyrosine kinase (LCK), mRNA.	-0.17	8.73	1.24E-14	4.02E-14	turquoise
LDLR	3949	low density lipoprotein receptor (familial hypercholesterolemia) (LDLR), mRNA.	1.01	7.11	1.96E-92	4.98E-90	turquoise
LGMN	5641	legumain (LGMN), transcript variant 1, mRNA.	0.12	7.20	1.96E-15	6.62E-15	turquoise

LGR4	55366	leucine-rich repeat-containing G protein-coupled receptor 4 (LGR4), mRNA.	0.16	8.37	7.39E-21	3.28E-20	turquoise
LIMK1	3984	LIM domain kinase 1 (LIMK1), mRNA.	-0.03	8.20	6.09E-02	7.86E-02	turquoise
LMBR1	64327	limb region 1 homolog (mouse) (LMBR1), mRNA.	-0.03	6.79	9.45E-03	1.35E-02	turquoise
LMO2	4005	LIM domain only 2 (rhombotin-like 1) (LMO2), mRNA.	-0.01	8.54	4.43E-01	4.90E-01	turquoise
LMO4	8543	LIM domain only 4 (LMO4), mRNA.	-0.10	7.75	3.08E-13	9.30E-13	turquoise
LONRF1	91694	LON peptidase N-terminal domain and ring finger 1 (LONRF1), mRNA.	-0.14	5.74	2.03E-12	5.85E-12	turquoise
LPCAT1	79888	acyltransferase like 2 (AYTL2), mRNA.	-0.11	9.01	8.53E-14	2.65E-13	turquoise
LPGAT1	9926	lysophosphatidylglycerol acyltransferase 1 (LPGAT1), mRNA.	-0.12	9.43	4.30E-20	1.85E-19	turquoise
LPIN1	23175	lipin 1 (LPIN1), mRNA.	-0.21	9.41	3.68E-33	2.72E-32	turquoise
LPIN2	9663	lipin 2 (LPIN2), mRNA.	-0.19	8.43	4.91E-35	3.94E-34	turquoise
LPXN	9404	leupaxin (LPXN), mRNA.	0.05	6.24	9.50E-05	1.66E-04	turquoise
LRIG1	26018	leucine-rich repeats and immunoglobulin-like domains 1 (LRIG1), mRNA.	-0.08	6.67	5.00E-12	1.41E-11	turquoise
LRMP	4033	lymphoid-restricted membrane protein (LRMP), mRNA.	-0.01	10.91	5.89E-01	6.31E-01	turquoise
LRRC1	55227	leucine rich repeat containing 1 (LRRC1), mRNA.	0.05	7.03	1.73E-03	2.67E-03	turquoise
LRRC16A	55604	leucine rich repeat containing 16 (LRRC16), mRNA.	-0.07	8.77	2.80E-04	4.69E-04	turquoise
LRRK1	79705	leucine-rich repeat kinase 1 (LRRK1), mRNA.	-0.10	9.34	4.43E-16	1.56E-15	turquoise

LTA	4049	lymphotoxin alpha (TNF superfamily, member 1) (LTA), mRNA.	0.29	6.09	1.36E-38	1.30E-37	turquoise
LTB	4050	lymphotoxin beta (TNF superfamily, member 3) (LTB), transcript variant 2, mRNA.	-0.07	6.89	1.76E-05	3.29E-05	turquoise
LY86	9450	lymphocyte antigen 86 (LY86), mRNA.	-0.01	6.51	6.90E-01	7.25E-01	turquoise
LYAR	55646	hypothetical protein FLJ20425 (LYAR), mRNA.	0.04	7.58	9.27E-03	1.32E-02	turquoise
LYL1	4066	lymphoblastic leukemia derived sequence 1 (LYL1), mRNA.	-0.09	8.98	3.10E-06	6.11E-06	turquoise
LYN	4067	v-yes-1 Yamaguchi sarcoma viral related oncogene homolog (LYN), mRNA.	0.02	8.89	1.01E-01	1.26E-01	turquoise
LYPD6B	130576	hypothetical protein LOC130576 (LOC130576), mRNA.	-0.14	7.62	2.37E-17	8.90E-17	turquoise
LYRM5	144363	LYR motif containing 5 (LYRM5), mRNA.	0.05	8.53	1.33E-04	2.29E-04	turquoise
LYSMD2	256586	LysM, putative peptidoglycan-binding, domain containing 2 (LYSMD2), mRNA.	-0.07	8.69	1.15E-07	2.49E-07	turquoise
MAEA	730744	PREDICTED: similar to macrophage erythroblast attacher (LOC730744), mRNA.	0.04	9.07	3.46E-04	5.75E-04	turquoise
MANBA	4126	mannosidase, beta A, lysosomal (MANBA), mRNA.	-0.01	7.82	4.63E-01	5.09E-01	turquoise

MAP2K1	5604	mitogen-activated protein kinase kinase 1 (MAP2K1), mRNA.	0.14	7.65	8.28E-17	3.03E-16	turquoise
MAP3K6	9064	mitogen-activated protein kinase kinase kinase 6 (MAP3K6), mRNA.	-0.13	7.74	1.15E-16	4.17E-16	turquoise
MAP3K7	6885	mitogen-activated protein kinase kinase kinase 7 (MAP3K7), transcript variant B, mRNA.	0.00	9.91	7.83E-01	8.11E-01	turquoise
MAP3K8	1326	mitogen-activated protein kinase kinase kinase 8 (MAP3K8), mRNA.	0.08	7.83	6.50E-05	1.15E-04	turquoise
MAP4K1	11184	mitogen-activated protein kinase kinase kinase kinase 1 (MAP4K1), transcript variant 2, mRNA.	0.58	5.65	3.91E-40	3.97E-39	turquoise
MAPK1	5594	mitogen-activated protein kinase 1 (MAPK1), transcript variant 2, mRNA.	0.35	6.22	2.79E-41	2.96E-40	turquoise
MAPK6	5597	mitogen-activated protein kinase 6 (MAPK6), mRNA.	-0.04	8.49	2.29E-03	3.48E-03	turquoise
MARCKS	4082	myristoylated alanine-rich protein kinase C substrate (MARCKS), mRNA.	0.01	7.40	6.61E-01	6.98E-01	turquoise
MARCKSL1	65108	MARCKS-like 1 (MARCKSL1), mRNA.	0.08	6.33	5.56E-10	1.39E-09	turquoise
MB21D1	115004	chromosome 6 open reading frame 150 (C6orf150), mRNA.	-0.10	7.26	2.90E-12	8.27E-12	turquoise

MCM3	4172	MCM3 minichromosome maintenance deficient 3 (S. cerevisiae) (MCM3), mRNA.	0.01	12.05	6.56E-01	6.94E-01	turquoise
MCM4	4173	MCM4 minichromosome maintenance deficient 4 (S. cerevisiae) (MCM4), transcript variant 1, mRNA.	-0.05	6.50	2.78E-02	3.74E-02	turquoise
MCTP1	79772	multiple C2 domains, transmembrane 1 (MCTP1), transcript variant S, mRNA.	-0.63	6.50	4.88E-69	2.12E-67	turquoise
MDH1	4190	malate dehydrogenase 1, NAD (soluble) (MDH1), mRNA.	0.44	9.80	9.73E-59	2.48E-57	turquoise
ME1	4199	malic enzyme 1, NADP(+)-dependent, cytosolic (ME1), mRNA.	0.07	10.42	1.22E-05	2.31E-05	turquoise
MED30	90390	thyroid hormone receptor associated protein 6 (THRAP6), mRNA.	-0.21	6.69	6.79E-33	4.99E-32	turquoise
MERTK	10461	c-mer proto-oncogene tyrosine kinase (MERTK), mRNA.	-0.03	9.41	2.50E-02	3.39E-02	turquoise
METTL7A	25840	methyltransferase like 7A (METTL7A), mRNA.	-0.52	7.72	4.78E-61	1.39E-59	turquoise
MFSD6	54842	FLJ20160 protein (FLJ20160), mRNA.	-0.10	7.07	2.65E-11	7.17E-11	turquoise
MIB1	57534	mindbomb homolog 1 (Drosophila) (MIB1), mRNA.	0.02	8.05	1.99E-01	2.37E-01	turquoise

MIR600HG	81571	PREDICTED: chromosome 9 open reading frame 45 (C9orf45), misc RNA.	0.10	6.72	1.29E-08	2.98E-08	turquoise
MKLN1	4289	muskelin 1, intracellular mediator containing kelch motifs (MKLN1), mRNA.	0.06	7.41	9.39E-05	1.64E-04	turquoise
MLEC	9761	KIAA0152 (KIAA0152), mRNA.	0.45	5.88	1.09E-57	2.57E-56	turquoise
MMD	23531	monocyte to macrophage differentiation-associated (MMD), mRNA.	-0.07	9.52	6.07E-06	1.17E-05	turquoise
MOB3B	79817	MOB1, Mps One Binder kinase activator-like 2B (yeast) (MOBKL2B), mRNA.	-0.09	7.92	2.80E-09	6.73E-09	turquoise
MOXD1	26002	monooxygenase, DBH-like 1 (MOXD1), transcript variant 2, mRNA.	0.22	5.88	1.79E-20	7.81E-20	turquoise
MPHOSPH10	10199	M-phase phosphoprotein 10 (U3 small nucleolar ribonucleoprotein) (MPHOSPH10), mRNA.	-0.64	7.83	3.61E-86	5.23E-84	turquoise
MPP6	51678	membrane protein, palmitoylated 6 (MAGUK p55 subfamily member 6) (MPP6), mRNA.	-0.19	7.74	1.18E-24	6.32E-24	turquoise
MRPS30	10884	mitochondrial ribosomal protein S30 (MRPS30), nuclear gene encoding mitochondrial protein, mRNA.	-0.07	8.75	3.50E-07	7.36E-07	turquoise

MRPS6	64968	mitochondrial ribosomal protein S6 (MRPS6), nuclear gene encoding mitochondrial protein, mRNA.	-0.08	7.98	6.80E-08	1.50E-07	turquoise
MSH6	2956	mutS homolog 6 (E. coli) (MSH6), mRNA.	0.06	7.16	1.12E-06	2.28E-06	turquoise
MSL3	10943	male-specific lethal 3-like 1 (Drosophila) (MSL3L1), transcript variant 2, mRNA.	-0.10	8.75	2.61E-10	6.70E-10	turquoise
MT1A	4489	metallothionein 1A (MT1A), mRNA.	0.14	7.18	2.08E-18	8.21E-18	turquoise
MT1F	4494	metallothionein 1F (MT1F), mRNA.	0.10	8.57	6.93E-09	1.63E-08	turquoise
MT1X	4501	metallothionein 1X (MT1X), mRNA.	-0.03	8.58	4.81E-02	6.28E-02	turquoise
MT2A	4502	metallothionein 2A (MT2A), mRNA.	-0.12	7.70	1.68E-18	6.68E-18	turquoise
MTX2	10651	metaxin 2 (MTX2), transcript variant 1, mRNA.	0.02	9.26	2.97E-01	3.41E-01	turquoise
MTX3	345778	metaxin 3 (MTX3), mRNA.	0.19	8.41	1.24E-25	6.89E-25	turquoise
MYC	4609	v-myc myelocytomatosis viral oncogene homolog (avian) (MYC), mRNA.	0.04	8.55	1.33E-03	2.08E-03	turquoise
MYH11	4629	myosin, heavy chain 11, smooth muscle (MYH11), transcript variant SM2B, mRNA.	0.31	10.85	6.76E-51	1.09E-49	turquoise
MYL12A	10627	myosin regulatory light chain MRCL3 (MRCL3), mRNA.	0.22	6.46	2.86E-28	1.76E-27	turquoise
MYO1D	4642	myosin ID (MYO1D), mRNA.	0.01	8.19	6.29E-01	6.68E-01	turquoise
MYO1E	4643	myosin IE (MYO1E), mRNA.	0.03	8.73	3.78E-02	5.01E-02	turquoise
MYO5C	55930	myosin VC (MYO5C), mRNA.	0.00	8.62	7.33E-01	7.64E-01	turquoise

N4BP2	55728	Nedd4 binding protein 2 (N4BP2), mRNA.	-0.02	7.06	1.14E-01	1.42E-01	turquoise
N4BP2L2	88523	CG016 (LOC88523), mRNA.	0.05	9.25	2.08E-05	3.85E-05	turquoise
N6AMT1	642897	PREDICTED: similar to N6-DNA methyltransferase A (LOC642897), mRNA.	-0.17	6.33	3.16E-14	1.00E-13	turquoise
NA	5026	purinergic receptor P2X, ligand-gated ion channel, 5 (P2RX5), transcript variant 3, mRNA.	0.17	9.72	6.63E-27	3.87E-26	turquoise
NA	5460	POU domain, class 5, transcription factor 1 (POU5F1), transcript variant 1, mRNA.	-0.20	9.39	1.10E-25	6.13E-25	turquoise
NA	201562	protein tyrosine phosphatase-like (proline instead of catalytic arginine), member b (PTPLB), mRNA.	-0.17	7.12	1.30E-18	5.18E-18	turquoise
NA	115352	Fc receptor-like 3 (FCRL3), transcript variant 2, mRNA.	0.14	7.79	5.19E-18	2.02E-17	turquoise
NA	6351	chemokine (C-C motif) ligand 4 (CCL4), transcript variant 1, mRNA.	0.08	11.63	1.68E-09	4.09E-09	turquoise
NA	4207	MADS box transcription enhancer factor 2, polypeptide B (myocyte enhancer factor 2B) (MEF2B), mRNA.	-0.07	6.31	8.50E-05	1.49E-04	turquoise
NA	730422	PREDICTED: similar to chemokine (C-C motif) ligand 3-like 3 (LOC730422), mRNA.	-0.04	8.11	3.19E-04	5.32E-04	turquoise

NA	5032	purinergic receptor P2Y, G-protein coupled, 11 (P2RY11), mRNA.	0.05	11.45	9.35E-04	1.48E-03	turquoise
NA	90925	PREDICTED: hypothetical protein LOC90925 (LOC90925), misc RNA.	-0.02	7.57	2.83E-01	3.25E-01	turquoise
NA	2744	glutaminase (GLS), mRNA.	0.01	7.21	4.18E-01	4.64E-01	turquoise
NA	30851	Tax1 (human T-cell leukemia virus type I) binding protein 3 (TAX1BP3), mRNA.	-0.01	6.89	5.50E-01	5.94E-01	turquoise
NA	56342	peter pan homolog (Drosophila) (PPAN), mRNA.	0.01	6.10	5.60E-01	6.03E-01	turquoise
NA	3336	heat shock 10kDa protein 1 (chaperonin 10) (HSPE1), mRNA.	0.01	10.72	5.73E-01	6.17E-01	turquoise
NA	3310	heat shock 70kDa protein 6 (HSP70B') (HSPA6), mRNA.	0.01	5.97	6.32E-01	6.71E-01	turquoise
NAAA	27163	N-acylsphingosine amidohydrolase (acid ceramidase)-like (ASAHL), transcript variant 2, mRNA.	-0.18	8.82	1.54E-36	1.33E-35	turquoise
NAB1	4664	NGFI-A binding protein 1 (EGR1 binding protein 1) (NAB1), mRNA.	-0.03	6.89	4.61E-02	6.04E-02	turquoise
NABP1	64859	oligonucleotide/oligosaccharide-binding fold containing 2A (OBFC2A), mRNA.	-0.02	8.53	1.58E-01	1.91E-01	turquoise
NAE1	8883	amyloid beta precursor protein binding protein 1 (APPBP1), transcript variant 1, mRNA.	0.14	6.21	2.84E-19	1.17E-18	turquoise

NAMPT	10135	pre-B-cell colony enhancing factor 1 (PBEF1), mRNA.	0.69	8.09	1.10E-74	6.35E-73	turquoise
NARFL	64428	nuclear prelamin A recognition factor-like (NARFL), mRNA.	0.00	7.50	9.17E-01	9.30E-01	turquoise
NCALD	83988	neurocalcin delta (NCALD), transcript variant 1, mRNA.	-0.04	9.30	3.65E-04	6.05E-04	turquoise
NCF4	4689	neutrophil cytosolic factor 4, 40kDa (NCF4), transcript variant 2, mRNA.	0.03	6.39	1.40E-01	1.71E-01	turquoise
NCK2	729030	PREDICTED: similar to NCK adaptor protein 2 (LOC729030), mRNA.	0.08	5.79	1.34E-07	2.89E-07	turquoise
NCOA3	8202	nuclear receptor coactivator 3 (NCOA3), transcript variant 2, mRNA.	-0.08	8.20	1.38E-11	3.79E-11	turquoise
NCOA7	135112	nuclear receptor coactivator 7 (NCOA7), mRNA.	-0.15	10.06	2.22E-20	9.64E-20	turquoise
NCR3	259197	natural cytotoxicity triggering receptor 3 (NCR3), mRNA.	0.08	8.36	7.37E-04	1.18E-03	turquoise
NDUFAF4	29078	chromosome 6 open reading frame 66 (C6orf66), mRNA.	-0.31	8.34	1.20E-52	2.08E-51	turquoise
NEK6	10783	NIMA (never in mitosis gene a)-related kinase 6 (NEK6), mRNA.	-0.47	8.62	2.10E-48	3.16E-47	turquoise
NEXN	91624	nexilin (F actin binding protein) (NEXN), mRNA.	-0.03	7.12	1.25E-01	1.54E-01	turquoise

NFATC1	4772	nuclear factor of activated T-cells, cytoplasmic, calcineurin-dependent 1 (NFATC1), transcript variant 1, mRNA.	0.14	8.60	3.82E-12	1.08E-11	turquoise
NFE2L3	9603	nuclear factor (erythroid-derived 2)-like 3 (NFE2L3), mRNA.	-0.08	9.61	1.45E-07	3.12E-07	turquoise
NFIL3	4783	nuclear factor, interleukin 3 regulated (NFIL3), mRNA.	0.11	6.88	4.35E-12	1.23E-11	turquoise
NFKB2	4791	nuclear factor of kappa light polypeptide gene enhancer in B-cells 2 (p49/p100) (NFKB2), transcript variant 3, mRNA.	0.02	5.91	4.30E-01	4.76E-01	turquoise
NFKBIZ	64332	nuclear factor of kappa light polypeptide gene enhancer in B-cells inhibitor, zeta (NFKBIZ), transcript variant 2, mRNA.	-0.09	8.41	1.04E-08	2.42E-08	turquoise
NGLY1	55768	N-glycanase 1 (NGLY1), mRNA.	0.07	7.01	9.56E-07	1.95E-06	turquoise
NHLRC3	387921	similar to RIKEN cDNA 8030451K01 (LOC387921), transcript variant 1, mRNA.	0.01	7.31	6.72E-01	7.08E-01	turquoise
NINJ1	4814	ninjurin 1 (NINJ1), mRNA.	0.00	5.60	9.56E-01	9.63E-01	turquoise
NIP7	51388	nuclear import 7 homolog (S. cerevisiae) (NIP7), mRNA.	0.08	8.36	6.33E-13	1.88E-12	turquoise

NIPA1	123606	non imprinted in Prader-Willi/Angelman syndrome 1 (NIPA1), mRNA.	-0.14	6.97	1.67E-19	6.96E-19	turquoise
NIPA2	81614	non imprinted in Prader-Willi/Angelman syndrome 2 (NIPA2), transcript variant 1, mRNA.	-0.12	5.76	1.51E-08	3.46E-08	turquoise
NLRP7	199713	NLR family, pyrin domain containing 7 (NLRP7), transcript variant 1, mRNA.	1.69	6.97	5.13E-85	6.51E-83	turquoise
NOC3L	64318	nucleolar complex associated 3 homolog (S. cerevisiae) (NOC3L), mRNA.	-0.03	8.14	2.80E-02	3.76E-02	turquoise
NOD2	64127	nucleotide-binding oligomerization domain containing 2 (NOD2), mRNA.	-0.05	5.84	6.88E-03	9.98E-03	turquoise
NPC1	4864	Niemann-Pick disease, type C1 (NPC1), mRNA.	-0.09	6.38	2.98E-10	7.60E-10	turquoise
NSUN2	54888	NOL1/NOP2/Sun domain family, member 2 (NSUN2), mRNA.	-0.21	7.03	1.38E-24	7.35E-24	turquoise
NT5C2	22978	5'-nucleotidase, cytosolic II (NT5C2), mRNA.	0.21	7.73	1.78E-23	8.94E-23	turquoise
NTAN1	123803	N-terminal asparagine amidase (NTAN1), mRNA.	0.00	7.17	9.84E-01	9.87E-01	turquoise
NUB1	401433	PREDICTED: hypothetical gene supported by AK127717 (LOC401433), misc RNA.	0.05	7.76	1.29E-02	1.82E-02	turquoise
NUP88	4927	nucleoporin 88kDa (NUP88), mRNA.	0.07	8.34	8.00E-06	1.53E-05	turquoise

OGFRL1	79627	opioid growth factor receptor-like 1 (OGFRL1), mRNA.	-0.11	6.37	1.17E-09	2.88E-09	turquoise
OPTN	10133	optineurin (OPTN), transcript variant 1, mRNA.	0.08	9.22	4.19E-09	1.00E-08	turquoise
ORC1	4998	origin recognition complex, subunit 1-like (yeast) (ORC1L), mRNA.	-0.03	9.74	5.99E-03	8.73E-03	turquoise
P2RY10	27334	purinergic receptor P2Y, G-protein coupled, 10 (P2RY10), transcript variant 1, mRNA.	0.03	6.84	1.38E-01	1.69E-01	turquoise
P2RY8	286530	purinergic receptor P2Y, G-protein coupled, 8 (P2RY8), mRNA.	0.03	8.49	1.67E-02	2.32E-02	turquoise
P4HA1	5033	procollagen-proline, 2-oxoglutarate 4-dioxygenase (proline 4-hydroxylase), alpha polypeptide I (P4HA1), transcript variant 1, mRNA.	-0.13	10.03	3.63E-17	1.35E-16	turquoise
PAFAH1B1	5048	platelet-activating factor acetylhydrolase, isoform Ib, alpha subunit 45kDa (PAFAH1B1), mRNA.	0.09	9.80	3.56E-08	7.98E-08	turquoise
PAIP2	51247	poly(A) binding protein interacting protein 2 (PAIP2), transcript variant 2, mRNA.	0.02	6.82	1.38E-01	1.68E-01	turquoise
PAK1IP1	55003	PAK1 interacting protein 1 (PAK1IP1), mRNA.	0.11	9.05	2.44E-07	5.19E-07	turquoise

PAQR4	124222	progestin and adipoQ receptor family member IV (PAQR4), mRNA.	0.40	7.11	4.51E-49	6.87E-48	turquoise
PAQR8	85315	progestin and adipoQ receptor family member VIII (PAQR8), mRNA.	0.13	6.61	1.20E-16	4.35E-16	turquoise
PARD6A	50855	par-6 partitioning defective 6 homolog alpha (C. elegans) (PARD6A), transcript variant 2, mRNA.	0.59	8.40	1.01E-73	5.47E-72	turquoise
PARM1	25849	DKFZP564O0823 protein (DKFZP564O0823), mRNA.	-0.89	9.18	2.32E-59	6.26E-58	turquoise
PARP15	165631	poly (ADP-ribose) polymerase family, member 15 (PARP15), mRNA.	-0.02	8.07	6.16E-02	7.94E-02	turquoise
PARP4	143	poly (ADP-ribose) polymerase family, member 4 (PARP4), mRNA.	0.03	7.11	5.80E-02	7.49E-02	turquoise
PCMT1	5110	protein-L-isoaspartate (D-aspartate) O-methyltransferase (PCMT1), mRNA.	0.19	7.60	7.24E-33	5.31E-32	turquoise
PCSK7	9159	proprotein convertase subtilisin/kexin type 7 (PCSK7), mRNA.	0.11	10.31	6.63E-15	2.18E-14	turquoise
PDCD2L	84306	programmed cell death 2-like (PDCD2L), mRNA.	0.10	9.10	2.09E-12	6.01E-12	turquoise
PDCD4	27250	programmed cell death 4 (neoplastic transformation inhibitor) (PDCD4), transcript variant 1, mRNA.	-0.04	10.10	1.56E-02	2.17E-02	turquoise

PDE7A	5150	phosphodiesterase 7A (PDE7A), transcript variant 1, mRNA.	-0.12	7.85	1.56E-12	4.52E-12	turquoise
PDLIM1	9124	PDZ and LIM domain 1 (elfin) (PDLIM1), mRNA.	0.01	7.48	2.00E-01	2.37E-01	turquoise
PEA15	8682	phosphoprotein enriched in astrocytes 15 (PEA15), mRNA.	0.04	8.39	2.77E-02	3.73E-02	turquoise
PECAM1	5175	platelet/endothelial cell adhesion molecule (CD31 antigen) (PECAM1), mRNA.	-0.13	10.35	2.34E-19	9.70E-19	turquoise
PER2	8864	period homolog 2 (Drosophila) (PER2), mRNA.	0.08	9.35	2.52E-05	4.63E-05	turquoise
PEX5	5830	peroxisomal biogenesis factor 5 (PEX5), mRNA.	0.68	6.88	8.44E-51	1.35E-49	turquoise
PFN2	5217	profilin 2 (PFN2), transcript variant 2, mRNA.	-0.07	7.78	7.28E-07	1.50E-06	turquoise
PHACTR1	221692	phosphatase and actin regulator 1 (PHACTR1), mRNA.	0.08	7.30	1.68E-05	3.14E-05	turquoise
PHACTR3	116154	phosphatase and actin regulator 3 (PHACTR3), transcript variant 1, mRNA.	0.20	9.82	6.34E-40	6.32E-39	turquoise
PHF21A	51317	PHD finger protein 21A (PHF21A), mRNA.	0.10	9.43	6.53E-15	2.15E-14	turquoise
PHLDA3	23612	pleckstrin homology-like domain, family A, member 3 (PHLDA3), mRNA.	-0.44	5.78	9.29E-54	1.70E-52	turquoise
PIGV	55650	phosphatidylinositol glycan anchor biosynthesis, class V (PIGV), mRNA.	-0.05	7.18	7.38E-03	1.07E-02	turquoise

PIK3CD	5293	phosphoinositide-3-kinase, catalytic, delta polypeptide (PIK3CD), mRNA.	-0.04	9.84	1.25E-02	1.76E-02	turquoise
PIK3IP1	113791	HGFL gene (MGC17330), mRNA.	-0.10	5.59	1.28E-08	2.97E-08	turquoise
PIK3R1	5295	phosphoinositide-3-kinase, regulatory subunit 1 (p85 alpha) (PIK3R1), transcript variant 1, mRNA.	0.05	10.47	4.16E-03	6.17E-03	turquoise
PIK3R6	146850	chromosome 17 open reading frame 38 (C17orf38), mRNA.	0.09	8.55	4.57E-10	1.15E-09	turquoise
PILRA	29992	paired immunoglobin-like type 2 receptor alpha (PILRA), transcript variant 1, mRNA.	0.31	9.32	2.47E-43	2.92E-42	turquoise
PIM1	5292	pim-1 oncogene (PIM1), mRNA.	0.00	6.44	7.80E-01	8.09E-01	turquoise
PIP5K1C	23396	phosphatidylinositol-4-phosphate 5-kinase, type I, gamma (PIP5K1C), mRNA.	0.24	9.01	1.13E-36	9.83E-36	turquoise
PKP4	8502	plakophilin 4 (PKP4), transcript variant 2, mRNA.	-0.07	7.23	5.20E-04	8.50E-04	turquoise
PLAC8	51316	placenta-specific 8 (PLAC8), mRNA.	0.67	8.60	1.64E-78	1.32E-76	turquoise
PLCG2	5336	phospholipase C, gamma 2 (phosphatidylinositol-specific) (PLCG2), mRNA.	0.00	8.20	9.90E-01	9.92E-01	turquoise
PLEK	5341	pleckstrin (PLEK), mRNA.	0.01	9.30	3.37E-01	3.82E-01	turquoise

PLEKHA1	59338	pleckstrin homology domain containing, family A (phosphoinositide binding specific) member 1 (PLEKHA1), transcript variant 2, mRNA.	-0.34	7.23	2.95E-45	3.81E-44	turquoise
PLEKHH3	79990	pleckstrin homology domain containing, family H (with MyTH4 domain) member 3 (PLEKHH3), mRNA.	0.01	6.14	5.97E-01	6.39E-01	turquoise
PLEKHO1	51177	pleckstrin homology domain containing, family O member 1 (PLEKHO1), mRNA.	-0.12	6.77	7.59E-17	2.79E-16	turquoise
PLIN2	123	adipose differentiation-related protein (ADFP), mRNA.	-0.32	6.97	2.72E-50	4.31E-49	turquoise
PMM1	5372	phosphomannomutase 1 (PMM1), mRNA.	-0.02	7.91	9.36E-02	1.18E-01	turquoise
PNP	4860	nucleoside phosphorylase (NP), mRNA.	0.04	7.94	6.75E-02	8.66E-02	turquoise
PNPLA8	50640	patatin-like phospholipase domain containing 8 (PNPLA8), mRNA.	-0.17	6.02	1.45E-24	7.73E-24	turquoise
POLB	5423	polymerase (DNA directed), beta (POLB), mRNA.	0.05	8.03	1.55E-02	2.15E-02	turquoise
POLR2C	5432	polymerase (RNA) II (DNA directed) polypeptide C, 33kDa (POLR2C), mRNA.	0.35	7.84	3.83E-53	6.73E-52	turquoise

POLR3K	51728	polymerase (RNA) III (DNA directed) polypeptide K, 12.3 kDa (POLR3K), mRNA.	-0.01	7.05	4.97E-01	5.42E-01	turquoise
PPFIBP2	8495	PTPRF interacting protein, binding protein 2 (liprin beta 2) (PPFIBP2), mRNA.	-0.24	6.44	1.27E-28	7.87E-28	turquoise
PPP1R15A	23645	protein phosphatase 1, regulatory (inhibitor) subunit 15A (PPP1R15A), mRNA.	0.63	8.73	2.13E-51	3.53E-50	turquoise
PPP2R3C	55012	protein phosphatase 2 (formerly 2A), regulatory subunit B", gamma (PPP2R3C), mRNA.	0.32	9.54	2.61E-45	3.40E-44	turquoise
PPP2R5E	5529	protein phosphatase 2, regulatory subunit B', epsilon isoform (PPP2R5E), mRNA.	0.26	8.45	1.55E-38	1.47E-37	turquoise
PPP4R1	9989	protein phosphatase 4, regulatory subunit 1 (PPP4R1), transcript variant 1, mRNA.	0.09	8.00	1.22E-07	2.65E-07	turquoise
PRDM1	639	PR domain containing 1, with ZNF domain (PRDM1), transcript variant 2, mRNA.	0.53	8.06	4.86E-67	1.88E-65	turquoise
PRDX4	10549	peroxiredoxin 4 (PRDX4), mRNA.	-0.05	9.74	2.78E-05	5.09E-05	turquoise
PRDX6	9588	peroxiredoxin 6 (PRDX6), mRNA.	0.01	5.87	5.42E-01	5.86E-01	turquoise
PRKAB1	5564	protein kinase, AMP-activated, beta 1 non-catalytic subunit (PRKAB1), mRNA.	0.02	7.65	2.81E-01	3.24E-01	turquoise

PRKCD	5580	protein kinase C, delta (PRKCD), transcript variant 2, mRNA.	-0.01	8.75	4.74E-01	5.19E-01	turquoise
PRKCE	5581	protein kinase C, epsilon (PRKCE), mRNA.	0.03	8.67	1.70E-02	2.35E-02	turquoise
PRMT1	3276	protein arginine methyltransferase 1 (PRMT1), transcript variant 1, mRNA.	-0.13	8.40	1.39E-17	5.30E-17	turquoise
PRMT6	55170	protein arginine methyltransferase 6 (PRMT6), mRNA.	0.00	6.19	9.17E-01	9.30E-01	turquoise
PRR5	55615	proline rich 5 (renal) (PRR5), transcript variant 2, mRNA.	0.15	9.17	1.46E-17	5.56E-17	turquoise
PSEN2	5664	presenilin 2 (Alzheimer disease 4) (PSEN2), transcript variant 2, mRNA.	0.14	7.41	5.44E-10	1.37E-09	turquoise
PTAFR	5724	platelet-activating factor receptor (PTAFR), mRNA.	0.06	8.15	2.88E-04	4.81E-04	turquoise
PTK2B	2185	PTK2B protein tyrosine kinase 2 beta (PTK2B), transcript variant 4, mRNA.	0.00	6.57	9.82E-01	9.85E-01	turquoise
PTPN1	5770	protein tyrosine phosphatase, non-receptor type 1 (PTPN1), mRNA.	0.23	8.56	7.49E-24	3.84E-23	turquoise
PTPN22	26191	protein tyrosine phosphatase, non-receptor type 22 (lymphoid) (PTPN22), transcript variant 1, mRNA.	-0.11	8.74	2.70E-16	9.59E-16	turquoise
PTPN6	5777	protein tyrosine phosphatase, non-receptor type 6 (PTPN6), transcript variant 1, mRNA.	0.09	7.01	3.35E-05	6.10E-05	turquoise

PYHIN1	149628	pyrin and HIN domain family, member 1 (PYHIN1), transcript variant b1, mRNA.	-0.20	7.55	1.10E-35	9.13E-35	turquoise
RAB11A	8766	RAB11A, member RAS oncogene family (RAB11A), mRNA.	0.16	5.67	9.23E-11	2.42E-10	turquoise
RAB2B	84932	RAB2B, member RAS oncogene family (RAB2B), mRNA.	-0.06	7.91	1.15E-03	1.81E-03	turquoise
RAB33A	9363	RAB33A, member RAS oncogene family (RAB33A), mRNA.	-0.08	8.65	1.33E-07	2.88E-07	turquoise
RAB3IP	117177	RAB3A interacting protein (rabin3) (RAB3IP), transcript variant beta 1, mRNA.	-0.01	6.18	5.44E-01	5.88E-01	turquoise
RAB9A	9367	RAB9A, member RAS oncogene family (RAB9A), mRNA.	0.07	7.31	6.20E-04	1.00E-03	turquoise
RABGGTB	5876	Rab geranylgeranyltransferase, beta subunit (RABGGTB), mRNA.	0.02	9.22	2.23E-01	2.62E-01	turquoise
RAP2C	57826	RAP2C, member of RAS oncogene family (RAP2C), mRNA.	-0.03	8.71	4.01E-02	5.29E-02	turquoise
RARA	5914	retinoic acid receptor, alpha (RARA), transcript variant 1, mRNA.	-0.29	7.44	1.03E-53	1.87E-52	turquoise
RASD1	51655	RAS, dexamethasone-induced 1 (RASD1), mRNA.	0.08	7.14	5.24E-05	9.34E-05	turquoise

RASGRP3	25780	RAS guanyl releasing protein 3 (calcium and DAG-regulated) (RASGRP3), mRNA.	0.09	5.79	4.25E-07	8.89E-07	turquoise
RASL11A	387496	RAS-like, family 11, member A (RASL11A), mRNA.	0.96	7.33	2.35E-88	3.98E-86	turquoise
RASSF2	9770	Ras association (RalGDS/AF-6) domain family 2 (RASSF2), transcript variant 1, mRNA.	0.18	7.43	2.06E-23	1.03E-22	turquoise
RASSF4	83937	Ras association (RalGDS/AF-6) domain family 4 (RASSF4), mRNA.	0.15	8.14	1.01E-18	4.06E-18	turquoise
RBPJ	3516	recombination signal binding protein for immunoglobulin kappa J region (RBPJ), transcript variant 2, mRNA.	0.18	6.44	3.20E-24	1.67E-23	turquoise
RCSD1	92241	RCSD domain containing 1 (RCSD1), mRNA.	0.04	7.24	4.66E-04	7.65E-04	turquoise
RDH11	51109	retinol dehydrogenase 11 (all-trans/9-cis/11-cis) (RDH11), mRNA.	-0.03	8.43	1.77E-02	2.44E-02	turquoise
REC8	9985	REC8-like 1 (yeast) (REC8L1), transcript variant 2, mRNA.	0.07	7.16	9.15E-04	1.46E-03	turquoise
REEP5	7905	receptor accessory protein 5 (REEP5), mRNA.	-0.04	7.13	3.19E-03	4.78E-03	turquoise
RERE	473	arginine-glutamic acid dipeptide (RE) repeats (RERE), transcript variant 3, mRNA.	-0.01	7.53	5.20E-01	5.65E-01	turquoise

RETSAT	54884	retinol saturase (all-trans-retinol 13,14-reductase) (RETSAT), mRNA.	0.01	8.78	2.20E-01	2.60E-01	turquoise
RFTN1	23180	raftlin, lipid raft linker 1 (RFTN1), mRNA.	0.01	7.86	4.65E-01	5.11E-01	turquoise
RFX5	5993	regulatory factor X, 5 (influences HLA class II expression) (RFX5), transcript variant 2, mRNA.	-0.07	10.10	6.61E-05	1.17E-04	turquoise
RGCC	28984	response gene to complement 32 (RGC32), mRNA.	-0.33	9.85	5.91E-55	1.17E-53	turquoise
RGL1	23179	ral guanine nucleotide dissociation stimulator-like 1 (RGL1), mRNA.	-0.57	8.67	2.58E-72	1.30E-70	turquoise
RGS1	5996	regulator of G-protein signalling 1 (RGS1), mRNA.	-0.49	6.94	4.84E-62	1.48E-60	turquoise
RGS14	10636	regulator of G-protein signalling 14 (RGS14), mRNA.	0.13	6.85	9.65E-16	3.33E-15	turquoise
RGS16	6004	regulator of G-protein signalling 16 (RGS16), mRNA.	-0.01	8.99	4.66E-01	5.12E-01	turquoise
RGS2	5997	regulator of G-protein signalling 2, 24kDa (RGS2), mRNA.	-0.09	7.86	3.68E-11	9.86E-11	turquoise
RGS20	8601	regulator of G-protein signalling 20 (RGS20), transcript variant 1, mRNA.	-0.07	5.52	3.16E-05	5.77E-05	turquoise
RHOB	388	ras homolog gene family, member B (RHOB), mRNA.	-0.01	7.09	5.14E-01	5.59E-01	turquoise
RHOBTB3	22836	Rho-related BTB domain containing 3 (RHOBTB3), mRNA.	-0.07	7.75	1.44E-04	2.47E-04	turquoise

RHOG	391	ras homolog gene family, member G (rho G) (RHOG), mRNA.	0.05	8.92	4.47E-05	8.03E-05	turquoise
RHOH	399	ras homolog gene family, member H (RHOH), mRNA.	-0.24	9.80	6.40E-22	2.98E-21	turquoise
RIC8A	60626	resistance to inhibitors of cholinesterase 8 homolog A (C. elegans) (RIC8A), mRNA.	0.03	10.75	2.58E-02	3.48E-02	turquoise
RIPK2	8767	receptor-interacting serine-threonine kinase 2 (RIPK2), mRNA.	0.04	10.73	3.18E-03	4.77E-03	turquoise
RITA1	84934	chromosome 12 open reading frame 52 (C12orf52), mRNA.	0.16	9.65	2.65E-37	2.37E-36	turquoise
RMI2	116028	chromosome 16 open reading frame 75 (C16orf75), mRNA.	0.03	6.61	2.25E-01	2.65E-01	turquoise
RNASET2	8635	ribonuclease T2 (RNASET2), mRNA.	-0.20	8.11	3.77E-33	2.78E-32	turquoise
RND3	390	Rho family GTPase 3 (RND3), mRNA.	-0.04	7.67	3.67E-03	5.47E-03	turquoise
RNF114	55905	zinc finger protein 313 (ZNF313), mRNA.	-0.02	7.12	2.58E-01	3.00E-01	turquoise
RNF122	79845	ring finger protein 122 (RNF122), mRNA.	-0.12	7.58	2.03E-18	8.00E-18	turquoise
RNF14	9604	ring finger protein 14 (RNF14), transcript variant 2, mRNA.	-0.11	6.12	5.74E-10	1.44E-09	turquoise
RNGTT	8732	RNA guanylyltransferase and 5'-phosphatase (RNGTT), mRNA.	-0.07	10.15	6.52E-06	1.26E-05	turquoise
RPF2	84154	brix domain containing 1 (BXDC1), mRNA.	-0.41	9.12	1.81E-42	2.07E-41	turquoise

RPL39L	116832	ribosomal protein L39-like (RPL39L), mRNA.	0.10	7.35	1.04E-10	2.71E-10	turquoise
RRAGC	64121	Ras-related GTP binding C (RRAGC), mRNA.	0.04	11.64	3.89E-03	5.77E-03	turquoise
RRP15	51018	ribosomal RNA processing 15 homolog (S. cerevisiae) (RRP15), mRNA.	-0.19	8.26	2.68E-21	1.21E-20	turquoise
RRP9	9136	RRP9, small subunit (SSU) processome component, homolog (yeast) (RRP9), mRNA.	-0.01	7.23	3.37E-01	3.82E-01	turquoise
RSBN1	54665	round spermatid basic protein 1 (RSBN1), mRNA.	0.02	6.34	2.36E-01	2.76E-01	turquoise
RTN2	6253	reticulon 2 (RTN2), transcript variant 3, mRNA.	-0.06	6.48	4.97E-04	8.15E-04	turquoise
RTN4	57142	reticulon 4 (RTN4), transcript variant 3, mRNA.	-0.07	7.42	1.70E-06	3.42E-06	turquoise
RUNX1	861	runt-related transcription factor 1 (acute myeloid leukemia 1; aml1 oncogene) (RUNX1), transcript variant 1, mRNA.	-0.01	6.68	4.94E-01	5.39E-01	turquoise
RUSC1	23623	RUN and SH3 domain containing 1 (RUSC1), mRNA.	-0.04	9.67	1.19E-04	2.06E-04	turquoise
RXRA	6256	retinoid X receptor, alpha (RXRA), mRNA.	-0.13	10.44	1.33E-20	5.84E-20	turquoise
RYBP	23429	RING1 and YY1 binding protein (RYBP), mRNA.	-0.13	9.27	2.86E-08	6.46E-08	turquoise

S100A10	6281	S100 calcium binding protein A10 (annexin II ligand, calpactin I, light polypeptide (p11)) (S100A10), mRNA.	-0.01	8.17	3.62E-01	4.08E-01	turquoise
S100A11	6282	S100 calcium binding protein A11 (S100A11), mRNA.	-0.04	9.97	5.84E-04	9.49E-04	turquoise
S1PR4	8698	endothelial differentiation, lysophosphatidic acid G-protein-coupled receptor, 6 (EDG6), mRNA.	-0.02	8.33	1.38E-01	1.68E-01	turquoise
SAMD4A	23034	sterile alpha motif domain containing 4A (SAMD4A), mRNA.	0.11	10.36	8.06E-19	3.25E-18	turquoise
SAMSN1	64092	SAM domain, SH3 domain and nuclear localization signals 1 (SAMSN1), mRNA.	0.13	10.79	1.10E-21	5.09E-21	turquoise
SAP30	8819	Sin3A-associated protein, 30kDa (SAP30), mRNA.	-0.14	6.16	2.51E-14	8.00E-14	turquoise
SASH3	54440	chromosome X open reading frame 9 (CXorf9), mRNA.	-0.12	7.61	1.52E-15	5.18E-15	turquoise
SC5D	6309	sterol-C5-desaturase (ERG3 delta-5-desaturase homolog, S. cerevisiae)-like (SC5DL), transcript variant 2, mRNA.	0.19	11.74	5.66E-27	3.31E-26	turquoise
SCAMP1	9522	secretory carrier membrane protein 1 (SCAMP1), mRNA.	0.07	8.59	9.59E-07	1.96E-06	turquoise
SCARB2	950	scavenger receptor class B, member 2 (SCARB2), mRNA.	0.00	5.80	9.42E-01	9.51E-01	turquoise
SCD	6319	stearoyl-CoA desaturase (delta-9-desaturase) (SCD), mRNA.	-0.16	10.52	1.72E-19	7.19E-19	turquoise

SCML1	6322	sex comb on midleg-like 1 (Drosophila) (SCML1), transcript variant 3, mRNA.	0.03	6.81	5.94E-02	7.66E-02	turquoise
SCYL3	57147	SCY1-like 3 (S. cerevisiae) (SCYL3), transcript variant 1, mRNA.	-0.05	6.01	1.07E-04	1.86E-04	turquoise
SDSL	113675	serine dehydratase-like (SDSL), mRNA.	-0.05	6.64	6.40E-04	1.03E-03	turquoise
SEC11A	23478	SEC11 homolog A (S. cerevisiae) (SEC11A), mRNA.	-0.14	5.83	4.76E-17	1.76E-16	turquoise
SEL1L3	23231	KIAA0746 protein (KIAA0746), mRNA.	0.01	5.46	3.78E-01	4.23E-01	turquoise
SELL	6402	selectin L (lymphocyte adhesion molecule 1) (SELL), mRNA.	0.10	7.31	1.07E-07	2.33E-07	turquoise
SELT	51714	selenoprotein T (SELT), mRNA.	0.06	7.09	2.34E-04	3.94E-04	turquoise
SEMA4D	10507	sema domain, immunoglobulin domain (Ig), transmembrane domain (TM) and short cytoplasmic domain, (semaphorin) 4D (SEMA4D), mRNA.	-0.12	7.67	2.18E-20	9.51E-20	turquoise
SEPHS2	22928	selenophosphate synthetase 2 (SEPHS2), mRNA.	0.12	10.29	2.37E-20	1.03E-19	turquoise
SERINC2	347735	serine incorporator 2 (SERINC2), mRNA.	0.09	9.55	1.10E-16	3.99E-16	turquoise
SERINC3	10955	serine incorporator 3 (SERINC3), transcript variant 2, mRNA.	0.00	9.55	9.85E-01	9.87E-01	turquoise
SERTAD2	9792	SERTA domain containing 2 (SERTAD2), mRNA.	0.01	10.83	3.49E-01	3.94E-01	turquoise

SERTAD3	29946	SERTA domain containing 3 (SERTAD3), transcript variant 2, mRNA.	-0.01	6.62	5.10E-01	5.55E-01	turquoise
SESN1	27244	sestrin 1 (SESN1), mRNA.	0.01	12.82	4.59E-01	5.05E-01	turquoise
SETBP1	26040	SET binding protein 1 (SETBP1), mRNA.	0.10	9.40	1.63E-08	3.75E-08	turquoise
SETD6	79918	SET domain containing 6 (SETD6), mRNA.	-0.09	6.03	7.98E-08	1.75E-07	turquoise
SFT2D1	113402	SFT2 domain containing 1 (SFT2D1), mRNA.	0.07	6.98	8.32E-06	1.59E-05	turquoise
SGK1	6446	serum/glucocorticoid regulated kinase (SGK), mRNA.	-0.12	9.96	1.00E-13	3.10E-13	turquoise
SH2B2	10603	SH2B adaptor protein 2 (SH2B2), mRNA.	-0.20	8.59	3.15E-35	2.55E-34	turquoise
SH2B3	10019	SH2B adaptor protein 3 (SH2B3), mRNA.	-0.07	8.46	9.21E-04	1.46E-03	turquoise
SH2D3C	10044	SH2 domain containing 3C (SH2D3C), transcript variant 2, mRNA.	0.01	6.99	6.19E-01	6.60E-01	turquoise
SH3PXD2A	9644	SH3 and PX domains 2A (SH3PXD2A), mRNA.	-0.05	8.26	1.04E-04	1.80E-04	turquoise
SHROOM3	57619	shroom family member 3 (SHROOM3), mRNA.	-0.02	8.76	2.39E-01	2.79E-01	turquoise
SIDT2	51092	SID1 transmembrane family, member 2 (SIDT2), mRNA.	0.02	7.26	2.50E-01	2.90E-01	turquoise
SKIDA1	730417	PREDICTED: hypothetical protein LOC730417 (LOC730417), mRNA.	0.06	7.99	3.54E-02	4.70E-02	turquoise

SLAMF1	6504	signaling lymphocytic activation molecule family member 1 (SLAMF1), mRNA.	0.04	8.87	2.58E-02	3.48E-02	turquoise
SLAMF7	57823	SLAM family member 7 (SLAMF7), mRNA.	-0.09	8.84	3.61E-11	9.66E-11	turquoise
SLBP	7884	stem-loop (histone) binding protein (SLBP), mRNA.	-0.05	7.61	1.21E-03	1.90E-03	turquoise
SLC15A3	51296	solute carrier family 15, member 3 (SLC15A3), mRNA.	-0.48	6.46	1.33E-52	2.29E-51	turquoise
SLC15A4	121260	solute carrier family 15, member 4 (SLC15A4), mRNA.	-0.27	5.43	3.98E-25	2.17E-24	turquoise
SLC16A10	117247	solute carrier family 16, member 10 (aromatic amino acid transporter) (SLC16A10), mRNA.	-0.02	6.79	1.25E-01	1.54E-01	turquoise
SLC16A3	9123	solute carrier family 16, member 3 (monocarboxylic acid transporter 4) (SLC16A3), transcript variant 3, mRNA.	0.01	8.55	7.38E-01	7.69E-01	turquoise
SLC16A9	220963	solute carrier family 16, member 9 (monocarboxylic acid transporter 9) (SLC16A9), mRNA.	-0.07	9.08	3.65E-06	7.17E-06	turquoise
SLC18B1	116843	chromosome 6 open reading frame 192 (C6orf192), mRNA.	0.31	8.72	2.61E-35	2.11E-34	turquoise

SLC1A1	6505	solute carrier family 1 (neuronal/epithelial high affinity glutamate transporter, system Xag), member 1 (SLC1A1), mRNA.	-0.52	6.07	5.41E-54	1.02E-52	turquoise
SLC1A4	6509	solute carrier family 1 (glutamate/neutral amino acid transporter), member 4 (SLC1A4), mRNA.	0.70	8.77	9.64E-59	2.47E-57	turquoise
SLC25A4	291	solute carrier family 25 (mitochondrial carrier; adenine nucleotide translocator), member 4 (SLC25A4), nuclear gene encoding mitochondrial protein, mRNA.	-0.17	8.77	8.63E-24	4.41E-23	turquoise
SLC27A2	11001	solute carrier family 27 (fatty acid transporter), member 2 (SLC27A2), mRNA.	-0.11	8.18	2.33E-14	7.44E-14	turquoise
SLC2A5	6518	solute carrier family 2 (facilitated glucose/fructose transporter), member 5 (SLC2A5), mRNA.	-0.19	7.61	1.19E-33	8.98E-33	turquoise
SLC35D2	11046	solute carrier family 35, member D2 (SLC35D2), mRNA.	-0.08	7.90	2.85E-12	8.14E-12	turquoise
SLC35E3	55508	solute carrier family 35, member E3 (SLC35E3), mRNA.	0.04	6.66	5.34E-04	8.72E-04	turquoise

SLC35F2	54733	solute carrier family 35, member F2 (SLC35F2), mRNA.	0.02	7.43	2.27E-01	2.67E-01	turquoise
SLC37A2	729049	PREDICTED: similar to T-box 1 isoform C (LOC729049), mRNA.	0.61	6.09	1.60E-58	4.04E-57	turquoise
SLC38A1	81539	solute carrier family 38, member 1 (SLC38A1), transcript variant 2, mRNA.	0.03	6.11	2.28E-02	3.10E-02	turquoise
SLC38A2	54407	solute carrier family 38, member 2 (SLC38A2), mRNA.	0.78	9.32	3.02E-90	6.39E-88	turquoise
SLC41A2	84102	solute carrier family 41, member 2 (SLC41A2), mRNA.	0.06	7.91	2.52E-04	4.24E-04	turquoise
SLC43A2	124935	solute carrier family 43, member 2 (SLC43A2), mRNA.	-0.02	7.18	1.73E-01	2.07E-01	turquoise
SLC44A1	23446	solute carrier family 44, member 1 (SLC44A1), mRNA.	0.10	6.89	1.99E-14	6.40E-14	turquoise
SLC44A2	57153	solute carrier family 44, member 2 (SLC44A2), mRNA.	-0.04	6.08	2.96E-02	3.97E-02	turquoise
SLC45A3	85414	solute carrier family 45, member 3 (SLC45A3), mRNA.	-0.07	10.70	2.45E-07	5.21E-07	turquoise
SLC6A4	6532	solute carrier family 6 (neurotransmitter transporter, serotonin), member 4 (SLC6A4), mRNA.	-0.17	7.89	1.84E-22	8.76E-22	turquoise

SLC7A7	9056	solute carrier family 7 (cationic amino acid transporter, y+ system), member 7 (SLC7A7), mRNA.	0.00	5.61	7.89E-01	8.16E-01	turquoise
SLC8A3	6547	solute carrier family 8 (sodium-calcium exchanger), member 3 (SLC8A3), transcript variant b, mRNA.	-0.10	8.17	8.21E-11	2.16E-10	turquoise
SLCO4A1	28231	solute carrier organic anion transporter family, member 4A1 (SLCO4A1), mRNA.	-0.04	11.15	1.18E-04	2.04E-04	turquoise
SLCO5A1	81796	solute carrier organic anion transporter family, member 5A1 (SLCO5A1), mRNA.	-0.06	8.58	2.64E-03	3.98E-03	turquoise
SLFN12	55106	schlafen family member 12 (SLFN12), mRNA.	0.11	8.57	1.12E-18	4.49E-18	turquoise
SMAD4	4089	SMAD family member 4 (SMAD4), mRNA.	-0.07	9.17	7.08E-05	1.25E-04	turquoise
SMAD5	4090	SMAD family member 5 (SMAD5), transcript variant 3, mRNA.	0.26	6.12	9.91E-30	6.36E-29	turquoise
SMAP2	64744	stromal membrane-associated protein 1-like (SMAP1L), mRNA.	0.03	8.51	1.04E-01	1.30E-01	turquoise
SMC6	79677	structural maintenance of chromosomes 6 (SMC6), mRNA.	-0.63	9.32	3.21E-74	1.79E-72	turquoise
SMIM3	85027	MSTP150 (MST150), mRNA.	-0.12	9.15	5.51E-22	2.57E-21	turquoise

SMYD3	64754	SET and MYND domain containing 3 (SMYD3), mRNA.	-0.05	6.02	8.14E-05	1.43E-04	turquoise
SNRNP70	6625	small nuclear ribonucleoprotein 70kDa polypeptide (RNP antigen) (SNRP70), transcript variant 2, mRNA.	0.07	9.53	3.37E-06	6.63E-06	turquoise
SNX2	6643	sorting nexin 2 (SNX2), mRNA.	-0.12	6.91	4.60E-18	1.79E-17	turquoise
SNX25	83891	sorting nexin 25 (SNX25), mRNA.	0.10	6.81	1.59E-07	3.42E-07	turquoise
SNX4	8723	sorting nexin 4 (SNX4), mRNA.	0.08	9.91	3.45E-05	6.26E-05	turquoise
SNX8	29886	sorting nexin 8 (SNX8), mRNA.	-0.01	7.69	5.76E-01	6.19E-01	turquoise
SOCS1	8651	suppressor of cytokine signaling 1 (SOCS1), mRNA.	0.03	7.67	4.66E-02	6.10E-02	turquoise
SOCS2	8835	suppressor of cytokine signaling 2 (SOCS2), mRNA.	0.01	10.74	4.23E-01	4.69E-01	turquoise
SOGA1	140710	chromosome 20 open reading frame 117 (C20orf117), transcript variant 2, mRNA.	-0.13	8.16	2.31E-17	8.70E-17	turquoise
SORT1	6272	sortilin 1 (SORT1), mRNA.	0.13	6.96	3.93E-12	1.11E-11	turquoise
SOX4	6659	SRY (sex determining region Y)-box 4 (SOX4), mRNA.	0.00	8.16	9.95E-01	9.96E-01	turquoise
SOX9	6662	SRY (sex determining region Y)-box 9 (campomelic dysplasia, autosomal sex-reversal) (SOX9), mRNA.	0.09	8.77	1.84E-12	5.31E-12	turquoise
SPATS2L	26010	DNA polymerase-transactivated protein 6 (DNAPTP6), mRNA.	-0.21	8.10	1.83E-22	8.70E-22	turquoise

SPC25	57405	SPC25, NDC80 kinetochore complex component, homolog (S. cerevisiae) (SPC25), mRNA.	0.00	5.39	9.34E-01	9.44E-01	turquoise
SPIB	6689	Spi-B transcription factor (Spi-1/PU.1 related) (SPIB), mRNA.	-0.05	9.84	2.29E-03	3.48E-03	turquoise
SPR	6697	sepiapterin reductase (7,8-dihydrobiopterin:NADP+ oxidoreductase) (SPR), mRNA.	-0.10	8.01	1.85E-12	5.34E-12	turquoise
SQSTM1	8878	sequestosome 1 (SQSTM1), mRNA.	0.14	6.49	4.32E-13	1.29E-12	turquoise
SRC	6714	v-src sarcoma (Schmidt-Ruppin A-2) viral oncogene homolog (avian) (SRC), transcript variant 1, mRNA.	0.07	7.68	1.45E-06	2.93E-06	turquoise
SREBF1	6720	sterol regulatory element binding transcription factor 1 (SREBF1), transcript variant 1, mRNA.	-0.40	6.71	8.34E-45	1.05E-43	turquoise
SRGN	5552	serglycin (SRGN), mRNA.	-0.07	7.25	4.21E-03	6.24E-03	turquoise
SRI	6717	sorcin (SRI), transcript variant 1, mRNA.	-0.17	7.17	1.54E-23	7.77E-23	turquoise
SRPRB	58477	signal recognition particle receptor, B subunit (SRPRB), mRNA.	-0.05	6.33	6.95E-04	1.12E-03	turquoise
ST13	6767	suppression of tumorigenicity 13 (colon carcinoma) (Hsp70 interacting protein) (ST13), mRNA.	-0.04	10.67	1.02E-02	1.44E-02	turquoise

ST3GAL1	6482	ST3 beta-galactoside alpha-2,3-sialyltransferase 1 (ST3GAL1), transcript variant 2, mRNA.	0.20	5.79	1.24E-22	5.99E-22	turquoise
ST3GAL5	8869	ST3 beta-galactoside alpha-2,3-sialyltransferase 5 (ST3GAL5), transcript variant 1, mRNA.	0.11	5.54	2.96E-10	7.55E-10	turquoise
ST6GAL1	6480	ST6 beta-galactosamide alpha-2,6-sialyltranferase 1 (ST6GAL1), transcript variant 3, mRNA.	-0.05	7.55	2.08E-03	3.19E-03	turquoise
ST8SIA4	7903	ST8 alpha-N-acetyl-neuraminide alpha-2,8-sialyltransferase 4 (ST8SIA4), transcript variant 2, mRNA.	-0.03	9.78	1.88E-02	2.58E-02	turquoise
STAG3	10734	stromal antigen 3 (STAG3), mRNA.	-0.03	7.45	1.73E-01	2.08E-01	turquoise
STAMBPL1	57559	STAM binding protein-like 1 (STAMBPL1), mRNA.	-0.17	9.66	7.20E-23	3.54E-22	turquoise
STIM1	6786	stromal interaction molecule 1 (STIM1), mRNA.	0.01	5.92	3.72E-01	4.18E-01	turquoise
STIP1	10963	stress-induced-phosphoprotein 1 (Hsp70/Hsp90-organizing protein) (STIP1), mRNA.	0.07	8.60	1.98E-04	3.35E-04	turquoise
STK17B	9262	serine/threonine kinase 17b (STK17B), mRNA.	0.01	12.13	4.11E-01	4.57E-01	turquoise

STK24	8428	serine/threonine kinase 24 (STE20 homolog, yeast) (STK24), transcript variant 2, mRNA.	-0.51	8.59	2.16E-66	8.11E-65	turquoise
STK38	11329	serine/threonine kinase 38 (STK38), mRNA.	0.01	7.98	4.35E-01	4.82E-01	turquoise
STK38L	23012	serine/threonine kinase 38 like (STK38L), mRNA.	-0.05	9.15	1.82E-03	2.80E-03	turquoise
STMN1	3925	stathmin 1/oncoprotein 18 (STMN1), transcript variant 1, mRNA.	0.40	9.69	6.66E-57	1.49E-55	turquoise
STOM	2040	stomatin (STOM), transcript variant 2, mRNA.	0.03	6.73	1.32E-02	1.85E-02	turquoise
STRBP	55342	spermatid perinuclear RNA binding protein (STRBP), mRNA.	-0.01	6.50	7.32E-01	7.64E-01	turquoise
STX7	8417	syntaxin 7 (STX7), mRNA.	-0.02	7.73	2.67E-01	3.09E-01	turquoise
SUCLG2	8801	succinate-CoA ligase, GDP-forming, beta subunit (SUCLG2), mRNA.	0.48	7.08	5.59E-84	6.75E-82	turquoise
SYNPO2L	79933	synaptopodin 2-like (SYNPO2L), mRNA.	-0.12	9.73	6.71E-19	2.72E-18	turquoise
SYTL3	94120	synaptotagmin-like 3 (SYTL3), mRNA.	0.09	7.16	1.75E-08	4.00E-08	turquoise
TANK	10010	TRAF family member-associated NFKB activator (TANK), transcript variant 1, mRNA.	0.10	9.34	8.13E-10	2.02E-09	turquoise
TAP1	6890	transporter 1, ATP-binding cassette, sub-family B (MDR/TAP) (TAP1), mRNA.	-0.05	8.36	7.32E-04	1.18E-03	turquoise

TBC1D22B	55633	TBC1 domain family, member 22B (TBC1D22B), mRNA.	-0.06	8.52	5.84E-08	1.29E-07	turquoise
TBC1D9	23158	TBC1 domain family, member 9 (with GRAM domain) (TBC1D9), mRNA.	-0.10	8.88	4.48E-13	1.34E-12	turquoise
TBCD	6904	tubulin folding cofactor D (TBCD), mRNA.	0.01	5.48	3.28E-01	3.72E-01	turquoise
TCF4	6925	transcription factor 4 (TCF4), mRNA.	0.00	8.80	9.26E-01	9.37E-01	turquoise
TDG	6996	thymine-DNA glycosylase (TDG), mRNA.	0.02	8.68	6.50E-02	8.36E-02	turquoise
TESK2	10420	testis-specific kinase 2 (TESK2), mRNA.	-0.63	6.78	2.20E-72	1.11E-70	turquoise
TEX9	374618	testis expressed sequence 9 (TEX9), mRNA.	0.08	8.66	4.96E-06	9.64E-06	turquoise
TFPI2	7980	tissue factor pathway inhibitor 2 (TFPI2), mRNA.	-0.23	8.26	1.53E-37	1.39E-36	turquoise
TGFB1I1	7041	transforming growth factor beta 1 induced transcript 1 (TGFB1I1), transcript variant 1, mRNA.	-0.03	7.34	4.02E-02	5.31E-02	turquoise
TGFBR3	7049	transforming growth factor, beta receptor III (TGFBR3), mRNA.	-0.03	8.52	1.34E-01	1.64E-01	turquoise
THAP11	57215	THAP domain containing 11 (THAP11), mRNA.	0.03	7.61	9.54E-02	1.20E-01	turquoise
THOP1	7064	thimet oligopeptidase 1 (THOP1), mRNA.	0.08	8.73	3.14E-07	6.62E-07	turquoise
TIPARP	25976	TCDD-inducible poly(ADP-ribose) polymerase (TIPARP), mRNA.	0.04	6.35	1.09E-02	1.54E-02	turquoise

TJP2	9414	tight junction protein 2 (zona occludens 2) (TJP2), transcript variant 1, mRNA.	-0.24	7.42	1.37E-41	1.47E-40	turquoise
TK1	7083	thymidine kinase 1, soluble (TK1), mRNA.	-0.01	6.21	6.30E-01	6.70E-01	turquoise
TLE1	7088	transducin-like enhancer of split 1 (E(sp1) homolog, Drosophila) (TLE1), mRNA.	0.04	5.95	8.37E-04	1.34E-03	turquoise
TLR10	81793	toll-like receptor 10 (TLR10), transcript variant 2, mRNA.	-0.11	5.78	1.18E-08	2.74E-08	turquoise
TM7SF3	51768	transmembrane 7 superfamily member 3 (TM7SF3), mRNA.	0.01	11.91	5.75E-01	6.18E-01	turquoise
TMC6	11322	transmembrane channel-like 6 (TMC6), mRNA.	0.02	7.28	3.10E-01	3.55E-01	turquoise
TMC8	147138	transmembrane channel-like 8 (TMC8), mRNA.	-0.34	9.12	3.82E-58	9.32E-57	turquoise
TMCO3	55002	transmembrane and coiled-coil domains 3 (TMCO3), mRNA.	0.48	10.71	6.78E-69	2.87E-67	turquoise
TMEM120A	83862	transmembrane protein induced by tumor necrosis factor alpha (TMPIT), mRNA.	0.50	7.12	3.00E-71	1.44E-69	turquoise
TMEM126A	84233	transmembrane protein 126A (TMEM126A), mRNA.	-0.17	6.99	2.89E-25	1.58E-24	turquoise
TMEM138	51524	transmembrane protein 138 (TMEM138), mRNA.	-0.24	6.56	7.13E-42	7.84E-41	turquoise
TMEM14C	51522	transmembrane protein 14C (TMEM14C), mRNA.	0.54	6.84	9.43E-57	2.08E-55	turquoise

TMEM173	340061	transmembrane protein 173 (TMEM173), mRNA.	-0.03	8.11	5.45E-03	7.98E-03	turquoise
TMEM177	80775	transmembrane protein 177 (TMEM177), mRNA.	0.04	8.29	2.05E-03	3.14E-03	turquoise
TMEM2	23670	transmembrane protein 2 (TMEM2), mRNA.	-0.02	10.46	1.37E-01	1.67E-01	turquoise
TMEM201	199953	hypothetical protein LOC199953 (RP13-15M17.2), mRNA.	-0.08	8.70	6.49E-06	1.25E-05	turquoise
TMEM217	221468	chromosome 6 open reading frame 128 (C6orf128), mRNA.	-0.04	7.57	1.97E-03	3.02E-03	turquoise
TMEM243	79161	chromosome 7 open reading frame 23 (C7orf23), mRNA.	-0.05	7.06	7.42E-04	1.19E-03	turquoise
TMEM41B	440026	transmembrane protein 41B (TMEM41B), mRNA.	-0.18	6.45	1.06E-26	6.13E-26	turquoise
TMEM44	93109	transmembrane protein 44 (TMEM44), transcript variant 2, mRNA.	-0.27	6.47	1.17E-19	4.92E-19	turquoise
TMEM97	27346	transmembrane protein 97 (TMEM97), mRNA.	-0.12	6.89	3.66E-11	9.79E-11	turquoise
TMOD1	7111	tropomodulin 1 (TMOD1), mRNA.	-0.07	9.33	1.61E-05	3.02E-05	turquoise
TMPO	7112	thymopoietin (TMPO), transcript variant 3, mRNA.	-0.19	7.63	6.48E-27	3.79E-26	turquoise
TMSB15B	286527	hypothetical protein MGC39900 (MGC39900), mRNA.	0.07	8.74	1.89E-05	3.52E-05	turquoise
TMUB1	83590	transmembrane and ubiquitin-like domain containing 1 (TMUB1), mRNA.	-0.31	7.13	4.13E-40	4.16E-39	turquoise

TMX1	81542	thioredoxin domain containing 1 (TXNDC1), mRNA.	-0.15	7.90	2.16E-18	8.50E-18	turquoise
TNF	7124	tumor necrosis factor (TNF superfamily, member 2) (TNF), mRNA.	-0.05	10.58	6.88E-07	1.42E-06	turquoise
TNFAIP3	7128	tumor necrosis factor, alpha-induced protein 3 (TNFAIP3), mRNA.	0.14	8.58	1.09E-13	3.34E-13	turquoise
TNFRSF17	608	tumor necrosis factor receptor superfamily, member 17 (TNFRSF17), mRNA.	-0.04	6.64	1.56E-02	2.17E-02	turquoise
TNFSF11	8600	tumor necrosis factor (ligand) superfamily, member 11 (TNFSF11), transcript variant 1, mRNA.	0.33	6.53	2.63E-31	1.80E-30	turquoise
TNFSF13B	10673	tumor necrosis factor (ligand) superfamily, member 13b (TNFSF13B), mRNA.	0.03	8.93	2.67E-03	4.02E-03	turquoise
TNFSF14	8740	tumor necrosis factor (ligand) superfamily, member 14 (TNFSF14), transcript variant 1, mRNA.	0.57	8.44	1.36E-64	4.82E-63	turquoise
TNFSF9	8744	tumor necrosis factor (ligand) superfamily, member 9 (TNFSF9), mRNA.	0.04	10.18	8.25E-04	1.32E-03	turquoise
TNIP2	79155	TNFAIP3 interacting protein 2 (TNIP2), mRNA.	-0.18	5.76	8.27E-22	3.84E-21	turquoise

TNPO2	30000	transportin 2 (importin 3, karyopherin beta 2b) (TNPO2), mRNA.	-0.37	7.06	1.69E-61	5.10E-60	turquoise
TNS3	64759	tensin 3 (TNS3), mRNA.	0.35	6.94	2.82E-37	2.52E-36	turquoise
TP53BP2	7159	tumor protein p53 binding protein, 2 (TP53BP2), transcript variant 1, mRNA.	0.02	10.44	9.00E-02	1.14E-01	turquoise
TP63	8626	tumor protein p73-like (TP73L), mRNA.	0.07	8.18	1.97E-07	4.22E-07	turquoise
TPST2	8459	tyrosylprotein sulfotransferase 2 (TPST2), transcript variant 1, mRNA.	-0.13	7.12	2.53E-16	9.02E-16	turquoise
TRAF1	7185	TNF receptor-associated factor 1 (TRAF1), mRNA.	-0.13	7.85	2.51E-15	8.44E-15	turquoise
TRAF3IP3	80342	TRAF3 interacting protein 3 (TRAF3IP3), mRNA.	0.03	10.76	9.45E-02	1.19E-01	turquoise
TRAFD1	10906	TRAF-type zinc finger domain containing 1 (TRAFD1), mRNA.	0.05	9.03	2.63E-03	3.96E-03	turquoise
TRAPPC2	6399	trafficking protein particle complex 2 (TRAPPC2), transcript variant 1, mRNA.	-0.28	7.44	2.74E-31	1.86E-30	turquoise
TRIAP1	51499	TP53 regulated inhibitor of apoptosis 1 (TRIAP1), mRNA.	-0.06	8.52	4.47E-02	5.87E-02	turquoise
TRIB1	10221	tribbles homolog 1 (Drosophila) (TRIB1), mRNA.	-0.05	6.31	1.54E-02	2.14E-02	turquoise
TRIM8	81603	tripartite motif-containing 8 (TRIM8), mRNA.	-0.05	8.45	2.75E-04	4.61E-04	turquoise

TRIOBP	11078	TRIO and F-actin binding protein (TRIOBP), transcript variant 6, mRNA.	0.06	5.99	1.18E-05	2.23E-05	turquoise
TRIP6	7205	thyroid hormone receptor interactor 6 (TRIP6), mRNA.	0.11	7.68	4.41E-10	1.11E-09	turquoise
TRMT11	60487	tRNA methyltransferase 11 homolog (S. cerevisiae) (TRMT11), mRNA.	0.01	5.98	6.19E-01	6.60E-01	turquoise
TSC22D3	1831	TSC22 domain family, member 3 (TSC22D3), transcript variant 2, mRNA.	-0.06	7.66	3.78E-04	6.25E-04	turquoise
TSR1	55720	TSR1, 20S rRNA accumulation, homolog (S. cerevisiae) (TSR1), mRNA.	0.03	8.24	5.31E-02	6.90E-02	turquoise
TTC19	54902	tetratricopeptide repeat domain 19 (TTC19), mRNA.	0.02	7.74	1.02E-01	1.27E-01	turquoise
TTC32	130502	tetratricopeptide repeat domain 32 (TTC32), mRNA.	-0.02	8.32	2.62E-01	3.04E-01	turquoise
TTC38	55020	hypothetical protein FLJ20699 (FLJ20699), mRNA.	0.13	8.07	1.35E-17	5.17E-17	turquoise
TTC39C	125488	chromosome 18 open reading frame 17 (C18orf17), mRNA.	0.07	8.07	2.75E-04	4.61E-04	turquoise
TTC5	91875	tetratricopeptide repeat domain 5 (TTC5), mRNA.	-0.16	8.85	5.12E-24	2.64E-23	turquoise
TXNIP	10628	thioredoxin interacting protein (TXNIP), mRNA.	-0.18	6.36	1.27E-15	4.36E-15	turquoise
TXNRD1	7296	thioredoxin reductase 1 (TXNRD1), transcript variant 1, mRNA.	-0.09	9.81	2.99E-09	7.20E-09	turquoise

TYMSOS	494514	chromosome 18 open reading frame 56 (C18orf56), mRNA.	-0.01	7.27	3.50E-01	3.95E-01	turquoise
TYW3	127253	tRNA-yW synthesizing protein 3 homolog (S. cerevisiae) (TYW3), mRNA.	-0.06	7.60	3.68E-07	7.71E-07	turquoise
U2AF2	11338	U2 small nuclear RNA auxiliary factor 2 (U2AF2), transcript variant 2, mRNA.	0.04	7.02	1.80E-02	2.48E-02	turquoise
UAP1L1	91373	UDP-N-acteylglucosamine pyrophosphorylase 1-like 1 (UAP1L1), mRNA.	0.59	8.03	5.07E-76	3.22E-74	turquoise
UBASH3B	84959	Cbl-interacting protein Sts-1 (STS-1), mRNA.	0.56	9.27	7.42E-75	4.33E-73	turquoise
UBE2E2	7325	ubiquitin-conjugating enzyme E2E 2 (UBC4/5 homolog, yeast) (UBE2E2), mRNA.	0.11	7.42	5.15E-12	1.45E-11	turquoise
UBE2G1	7326	ubiquitin-conjugating enzyme E2G 1 (UBC7 homolog, yeast) (UBE2G1), mRNA.	-0.07	6.66	7.47E-09	1.75E-08	turquoise
UBE2Q2	92912	ubiquitin-conjugating enzyme E2Q (putative) 2 (UBE2Q2), mRNA.	-0.11	9.32	2.20E-22	1.04E-21	turquoise
UBE2S	731049	PREDICTED: similar to Ubiquitin-conjugating enzyme E2S (Ubiquitin-conjugating enzyme E2-24 kDa) (Ubiquitin-protein ligase) (Ubiquitin carrier protein) (E2-EPF5) (LOC731049), mRNA.	0.00	8.65	7.25E-01	7.57E-01	turquoise

UBL3	5412	ubiquitin-like 3 (UBL3), mRNA.	0.00	9.48	8.14E-01	8.39E-01	turquoise
UGCG	7357	UDP-glucose ceramide glucosyltransferase (UGCG), mRNA.	0.06	9.06	1.61E-05	3.02E-05	turquoise
UGDH	7358	UDP-glucose dehydrogenase (UGDH), mRNA.	-0.07	8.15	1.36E-06	2.75E-06	turquoise
UGT2B11	10720	UDP glucuronosyltransferase 2 family, polypeptide B11 (UGT2B11), mRNA.	0.00	7.19	7.88E-01	8.15E-01	turquoise
UGT2B17	7367	UDP glucuronosyltransferase 2 family, polypeptide B17 (UGT2B17), mRNA.	0.04	6.59	9.30E-04	1.48E-03	turquoise
UGT2B7	7364	UDP glucuronosyltransferase 2 family, polypeptide B7 (UGT2B7), mRNA.	0.07	10.26	1.88E-03	2.90E-03	turquoise
UNG	7374	uracil-DNA glycosylase (UNG), transcript variant 2, mRNA.	-0.25	5.98	1.10E-22	5.34E-22	turquoise
USP9X	8239	ubiquitin specific peptidase 9, X-linked (USP9X), transcript variant 4, mRNA.	-0.66	6.19	3.22E-61	9.50E-60	turquoise
UVRAG	7405	UV radiation resistance associated gene (UVRAG), mRNA.	0.04	7.07	5.03E-03	7.39E-03	turquoise
VAMP5	10791	vesicle-associated membrane protein 5 (myobrevin) (VAMP5), mRNA.	0.00	8.54	7.62E-01	7.92E-01	turquoise
VASH2	79805	vasohibin 2 (VASH2), mRNA.	-0.13	7.80	1.54E-17	5.85E-17	turquoise

VASP	7408	vasodilator-stimulated phosphoprotein (VASP), transcript variant 2, mRNA.	0.06	6.64	1.15E-04	2.00E-04	turquoise
VCL	7414	vinculin (VCL), transcript variant 1, mRNA.	0.01	10.52	3.29E-01	3.73E-01	turquoise
VIMP	55829	selenoprotein S (SELS), transcript variant 2, mRNA.	0.01	7.43	1.77E-01	2.12E-01	turquoise
VPS41	27072	vacuolar protein sorting 41 homolog (S. cerevisiae) (VPS41), transcript variant 2, mRNA.	0.05	6.82	1.72E-02	2.37E-02	turquoise
VSTM2L	128434	chromosome 20 open reading frame 102 (C20orf102), mRNA.	-0.07	8.15	1.37E-09	3.36E-09	turquoise
WASL	8976	Wiskott-Aldrich syndrome-like (WASL), mRNA.	0.44	7.29	4.06E-40	4.10E-39	turquoise
WDR41	55255	WD repeat domain 41 (WDR41), mRNA.	0.02	7.41	7.76E-02	9.87E-02	turquoise
WFS1	7466	Wolfram syndrome 1 (wolframin) (WFS1), mRNA.	-0.05	6.85	3.29E-03	4.93E-03	turquoise
XPR1	9213	xenotropic and polytropic retrovirus receptor (XPR1), mRNA.	0.01	10.68	3.89E-01	4.34E-01	turquoise
YBX3	8531	cold shock domain protein A (CSDA), mRNA.	0.02	8.98	1.23E-01	1.52E-01	turquoise
YPEL5	51646	yippee-like 5 (Drosophila) (YPEL5), mRNA.	-0.08	7.59	3.94E-11	1.05E-10	turquoise
ZBED1	9189	zinc finger, BED-type containing 1 (ZBED1), mRNA.	0.12	5.72	1.06E-16	3.86E-16	turquoise
ZBED2	79413	zinc finger, BED-type containing 2 (ZBED2), mRNA.	-0.14	9.35	6.05E-20	2.58E-19	turquoise

ZBTB20	26137	zinc finger and BTB domain containing 20 (ZBTB20), mRNA.	-0.15	9.31	2.13E-19	8.83E-19	turquoise
ZCCHC7	84186	zinc finger, CCHC domain containing 7 (ZCCHC7), mRNA.	-0.08	7.20	1.48E-04	2.54E-04	turquoise
ZDHHC14	79683	zinc finger, DHHC-type containing 14 (ZDHHC14), transcript variant 1, mRNA.	0.21	7.00	4.70E-27	2.76E-26	turquoise
ZFP36L1	677	zinc finger protein 36, C3H type-like 1 (ZFP36L1), mRNA.	-0.17	6.79	5.97E-29	3.74E-28	turquoise
ZFP91	80829	zinc finger protein 91 homolog (mouse) (ZFP91), transcript variant 1, mRNA.	0.35	9.41	3.77E-41	3.97E-40	turquoise
ZFPM1	161882	zinc finger protein, multitype 1 (ZFPM1), mRNA.	1.17	8.53	3.03E-98	1.10E-95	turquoise
ZMYM6NB	9204	zinc finger, MYM-type 6 (ZMYM6), mRNA.	-0.46	6.95	1.93E-57	4.47E-56	turquoise
ZNF143	7702	zinc finger protein 143 (ZNF143), mRNA.	-0.19	9.11	6.85E-30	4.42E-29	turquoise
ZNF148	7707	zinc finger protein 148 (ZNF148), mRNA.	0.23	8.20	2.31E-19	9.58E-19	turquoise
ZNF281	23528	zinc finger protein 281 (ZNF281), mRNA.	-0.01	8.25	5.35E-01	5.80E-01	turquoise
ZNF296	162979	zinc finger protein 342 (ZNF342), mRNA.	-0.10	8.60	3.00E-17	1.12E-16	turquoise
ZNF318	24149	zinc finger protein 318 (ZNF318), mRNA.	-0.01	7.69	2.61E-01	3.03E-01	turquoise
ZNF573	126231	zinc finger protein 573 (ZNF573), mRNA.	-0.30	10.65	5.20E-56	1.10E-54	turquoise
ZNF827	152485	hypothetical protein LOC152485 (LOC152485), mRNA.	-0.07	7.57	5.35E-07	1.11E-06	turquoise

ZNF84	7637	zinc finger protein 84 (ZNF84), mRNA.	0.22	7.73	4.20E-27	2.48E-26	turquoise
ZNFX1	57169	zinc finger, NFX1-type containing 1 (ZNFX1), mRNA.	-0.04	9.74	1.57E-04	2.68E-04	turquoise
ZRANB2	9406	zinc finger, RAN-binding domain containing 2 (ZRANB2), transcript variant 1, mRNA.	-0.12	8.17	1.57E-13	4.80E-13	turquoise
6-Sep	23157	septin 6 (SEPT6), transcript variant I, mRNA.	-0.35	7.68	1.20E-47	1.77E-46	yellow
ACAP1	9744	centaurin, beta 1 (CENTB1), mRNA.	-0.01	10.83	7.18E-01	7.51E-01	yellow
ACAT1	38	acetyl-Coenzyme A acetyltransferase 1 (acetoacetyl Coenzyme A thiolase) (ACAT1), nuclear gene encoding mitochondrial protein, mRNA.	0.01	7.40	4.43E-01	4.89E-01	yellow
ACIN1	22985	apoptotic chromatin condensation inducer 1 (ACIN1), mRNA.	-0.12	9.20	8.14E-15	2.67E-14	yellow
ACSS1	84532	acyl-CoA synthetase short-chain family member 1 (ACSS1), nuclear gene encoding mitochondrial protein, mRNA.	-0.04	10.58	9.45E-04	1.50E-03	yellow
ACTN4	81	actinin, alpha 4 (ACTN4), mRNA.	-0.10	5.64	1.97E-07	4.22E-07	yellow
ADAP2	55803	centaurin, alpha 2 (CENTA2), mRNA.	-0.03	7.52	7.42E-03	1.07E-02	yellow
ADRM1	11047	adhesion regulating molecule 1 (ADRM1), transcript variant 1, mRNA.	0.04	8.06	3.97E-03	5.90E-03	yellow

AFG3L2	10939	AFG3 ATPase family gene 3-like 2 (yeast) (AFG3L2), nuclear gene encoding mitochondrial protein, mRNA.	-0.14	7.98	4.24E-21	1.90E-20	yellow
AKNA	80709	AT-hook transcription factor (AKNA), mRNA.	0.02	8.61	2.55E-02	3.46E-02	yellow
ALDH2	217	aldehyde dehydrogenase 2 family (mitochondrial) (ALDH2), nuclear gene encoding mitochondrial protein, mRNA.	-0.01	9.41	2.93E-01	3.37E-01	yellow
ALDH3A2	224	aldehyde dehydrogenase 3 family, member A2 (ALDH3A2), transcript variant 1, mRNA.	-0.26	6.69	1.26E-37	1.15E-36	yellow
ALDH9A1	223	aldehyde dehydrogenase 9 family, member A1 (ALDH9A1), mRNA.	-0.04	7.68	5.83E-03	8.50E-03	yellow
ALDOC	230	aldolase C, fructose-bisphosphate (ALDOC), mRNA.	-0.07	8.19	1.89E-03	2.91E-03	yellow
ALG13	55849	asparagine-linked glycosylation 13 homolog (S. cerevisiae) (ALG13), mRNA.	-0.05	7.03	5.55E-03	8.12E-03	yellow
AMOT	154796	angiomotin (AMOT), mRNA.	0.02	12.29	2.62E-01	3.04E-01	yellow
ANAPC5	51433	anaphase promoting complex subunit 5 (ANAPC5), mRNA.	-0.08	7.13	5.14E-08	1.14E-07	yellow
AP2A1	160	adaptor-related protein complex 2, alpha 1 subunit (AP2A1), transcript variant 1, mRNA.	0.00	8.38	9.73E-01	9.77E-01	yellow

AP3D1	8943	adaptor-related protein complex 3, delta 1 subunit (AP3D1), transcript variant 2, mRNA.	0.04	5.81	9.69E-03	1.38E-02	yellow
APEH	327	N-acylaminoacyl-peptide hydrolase (APEH), mRNA.	-0.10	5.58	4.39E-09	1.05E-08	yellow
APMAP	57136	chromosome 20 open reading frame 3 (C20orf3), mRNA.	0.25	7.35	6.41E-30	4.14E-29	yellow
APOBEC3C	27350	apolipoprotein B mRNA editing enzyme, catalytic polypeptide-like 3C (APOBEC3C), mRNA.	-0.19	6.00	1.68E-28	1.04E-27	yellow
ARHGDIA	396	Rho GDP dissociation inhibitor (GDI) alpha (ARHGDIA), mRNA.	0.08	7.05	7.79E-06	1.49E-05	yellow
ATAD3A	55210	ATPase family, AAA domain containing 3A (ATAD3A), mRNA.	-0.10	8.82	3.08E-05	5.62E-05	yellow
ATN1	1822	atrophin 1 (ATN1), transcript variant 1, mRNA.	0.09	8.73	2.27E-05	4.19E-05	yellow
ATP13A1	57130	ATPase type 13A1 (ATP13A1), mRNA.	-0.02	7.55	2.49E-01	2.90E-01	yellow
AXIN1	8312	axin 1 (AXIN1), transcript variant 2, mRNA.	0.04	7.51	1.23E-02	1.74E-02	yellow
B4GALT1	2683	UDP-Gal:betaGlcNAc beta 1,4- galactosyltransferase, polypeptide 1 (B4GALT1), mRNA.	-0.03	7.37	1.24E-02	1.74E-02	yellow
BAX	581	BCL2-associated X protein (BAX), transcript variant beta, mRNA.	0.17	8.11	1.20E-22	5.78E-22	yellow

BBX	56987	bobby sox homolog (Drosophila) (BBX), mRNA.	0.04	10.22	1.81E-04	3.08E-04	yellow
BCL3	602	B-cell CLL/lymphoma 3 (BCL3), mRNA.	-0.29	7.14	1.77E-45	2.34E-44	yellow
BIRC3	330	baculoviral IAP repeat-containing 3 (BIRC3), transcript variant 1, mRNA.	-0.13	8.89	8.25E-24	4.22E-23	yellow
BLVRA	644	biliverdin reductase A (BLVRA), mRNA.	-0.14	6.27	2.91E-18	1.14E-17	yellow
BNIP3	664	BCL2/adenovirus E1B 19kDa interacting protein 3 (BNIP3), nuclear gene encoding mitochondrial protein, mRNA.	0.07	7.61	1.30E-07	2.82E-07	yellow
BSG	682	basigin (Ok blood group) (BSG), transcript variant 2, mRNA.	-0.03	5.53	7.64E-02	9.72E-02	yellow
C10orf76	79591	chromosome 10 open reading frame 76 (C10orf76), mRNA.	0.02	9.22	2.58E-01	3.00E-01	yellow
C15orf57	90416	coiled-coil domain containing 32 (CCDC32), transcript variant 1, mRNA.	0.07	8.07	1.08E-07	2.35E-07	yellow
C17orf62	79415	chromosome 17 open reading frame 62 (C17orf62), mRNA.	0.06	6.51	6.18E-04	1.00E-03	yellow
C2orf49	79074	chromosome 2 open reading frame 49 (C2orf49), mRNA.	0.10	11.00	3.35E-14	1.07E-13	yellow
C7orf26	79034	chromosome 7 open reading frame 26 (C7orf26), mRNA.	-0.08	6.92	1.47E-06	2.97E-06	yellow
CABIN1	23523	calcineurin binding protein 1 (CABIN1), mRNA.	0.00	7.12	9.20E-01	9.32E-01	yellow

CAD	790	carbamoyl-phosphate synthetase 2, aspartate transcarbamylase, and dihydroorotase (CAD), mRNA.	0.00	9.16	8.24E-01	8.48E-01	yellow
CALR	811	calreticulin (CALR), mRNA.	-0.06	8.18	1.36E-05	2.56E-05	yellow
CANT1	124583	calcium activated nucleotidase 1 (CANT1), mRNA.	0.33	8.68	4.86E-43	5.66E-42	yellow
CARM1	10498	coactivator-associated arginine methyltransferase 1 (CARM1), mRNA.	0.04	6.92	1.53E-02	2.13E-02	yellow
CCAR2	57805	KIAA1967 (KIAA1967), transcript variant 1, mRNA.	0.01	7.51	6.00E-01	6.42E-01	yellow
CD86	942	CD86 molecule (CD86), transcript variant 2, mRNA.	2.16	7.20	2.75E-108	1.74E-105	yellow
CDC25B	994	cell division cycle 25 homolog B (S. pombe) (CDC25B), transcript variant 3, mRNA.	-1.00	8.48	2.85E-88	4.67E-86	yellow
CDCA8	55143	cell division cycle associated 8 (CDCA8), mRNA.	0.12	8.38	1.81E-11	4.93E-11	yellow
CDK5RAP3	80279	CDK5 regulatory subunit associated protein 3 (CDK5RAP3), transcript variant 1, mRNA.	0.00	7.59	8.77E-01	8.96E-01	yellow
CHERP	10523	calcium homeostasis endoplasmic reticulum protein (CHERP), mRNA.	-0.01	9.70	3.92E-01	4.37E-01	yellow
CHMP1A	5119	procollagen (type III) N-endopeptidase (PCOLN3), mRNA.	-0.08	7.20	1.95E-11	5.32E-11	yellow

CHPF	79586	chondroitin polymerizing factor (CHPF), mRNA.	-0.05	9.57	2.50E-06	4.96E-06	yellow
CHTF18	63922	CTF18, chromosome transmission fidelity factor 18 homolog (S. cerevisiae) (CHTF18), mRNA.	0.04	11.46	2.15E-03	3.28E-03	yellow
CHURC1	91612	churchill domain containing 1 (CHURC1), mRNA.	-0.07	7.33	4.16E-06	8.12E-06	yellow
CIRBP	1153	cold inducible RNA binding protein (CIRBP), mRNA.	-0.22	7.54	1.31E-29	8.35E-29	yellow
CLPTM1	1209	cleft lip and palate associated transmembrane protein 1 (CLPTM1), mRNA.	-0.32	6.12	2.68E-40	2.75E-39	yellow
CLUH	23277	KIAA0664 (KIAA0664), mRNA.	-0.03	7.99	2.56E-02	3.47E-02	yellow
CNEP1R1	255919	chromosome 16 open reading frame 69 (C16orf69), mRNA.	-0.85	7.45	2.62E-78	2.05E-76	yellow
CNOT3	4849	CCR4-NOT transcription complex, subunit 3 (CNOT3), mRNA.	0.27	7.50	4.41E-30	2.86E-29	yellow
CNPPD1	27013	chromosome 2 open reading frame 24 (C2orf24), mRNA.	-0.01	7.53	3.28E-01	3.72E-01	yellow
COL9A2	1298	collagen, type IX, alpha 2 (COL9A2), mRNA.	0.17	6.00	1.62E-21	7.43E-21	yellow
COQ5	84274	coenzyme Q5 homolog, methyltransferase (S. cerevisiae) (COQ5), mRNA.	0.04	8.65	2.65E-02	3.58E-02	yellow
CORO7	79585	coronin 7 (CORO7), mRNA.	0.01	8.07	3.45E-01	3.90E-01	yellow

COTL1	23406	coactosin-like 1 (Dictyostelium) (COTL1), mRNA.	-0.02	10.30	1.88E-01	2.24E-01	yellow
CSRP1	1465	cysteine and glycine-rich protein 1 (CSRP1), mRNA.	0.00	8.79	8.66E-01	8.87E-01	yellow
CTDNEP1	23399	dullard homolog (Xenopus laevis) (DULLARD), mRNA.	-0.16	11.17	1.96E-22	9.32E-22	yellow
CYB5R2	51700	cytochrome b5 reductase 2 (CYB5R2), mRNA.	-0.05	10.67	4.25E-05	7.64E-05	yellow
DDX24	57062	DEAD (Asp-Glu-Ala-Asp) box polypeptide 24 (DDX24), mRNA.	-0.11	8.12	1.82E-14	5.85E-14	yellow
DDX56	54606	DEAD (Asp-Glu-Ala-Asp) box polypeptide 56 (DDX56), mRNA.	0.19	8.29	2.74E-25	1.51E-24	yellow
DECR1	1666	2,4-dienoyl CoA reductase 1, mitochondrial (DECR1), nuclear gene encoding mitochondrial protein, mRNA.	-0.12	7.68	1.94E-08	4.43E-08	yellow
DEF8	54849	hypothetical protein FLJ20186 (FLJ20186), transcript variant 1, mRNA.	-0.03	7.83	1.48E-02	2.06E-02	yellow
DHX30	22907	DEAH (Asp-Glu-Ala-His) box polypeptide 30 (DHX30), transcript variant 2, mRNA.	0.02	5.38	3.18E-01	3.62E-01	yellow
DHX32	55760	DEAH (Asp-Glu-Ala-His) box polypeptide 32 (DHX32), mRNA.	-0.12	7.45	3.24E-10	8.24E-10	yellow
DHX38	9785	DEAH (Asp-Glu-Ala-His) box polypeptide 38 (DHX38), mRNA.	0.09	6.80	1.11E-05	2.11E-05	yellow

DLGAP4	22839	discs, large (Drosophila) homolog-associated protein 4 (DLGAP4), transcript variant 3, mRNA.	-0.08	8.07	1.52E-03	2.36E-03	yellow
DNM2	1785	dynamin 2 (DNM2), transcript variant 2, mRNA.	0.44	8.26	8.70E-59	2.26E-57	yellow
DPH5	51611	DPH5 homolog (S. cerevisiae) (DPH5), transcript variant 2, mRNA.	-0.03	8.27	2.41E-02	3.28E-02	yellow
DPP9	91039	dipeptidyl-peptidase 9 (DPP9), mRNA.	0.19	7.32	2.32E-31	1.59E-30	yellow
ECI2	10455	peroxisomal D3,D2-enoyl-CoA isomerase (PECI), transcript variant 2, mRNA.	-0.04	6.05	5.23E-03	7.67E-03	yellow
EFTUD2	9343	elongation factor Tu GTP binding domain containing 2 (EFTUD2), mRNA.	-0.07	7.16	3.56E-05	6.45E-05	yellow
EIF1B	10289	eukaryotic translation initiation factor 1B (EIF1B), mRNA.	0.21	7.86	2.25E-25	1.24E-24	yellow
EIF2S3	1968	eukaryotic translation initiation factor 2, subunit 3 gamma, 52kDa (EIF2S3), mRNA.	0.25	6.70	1.59E-40	1.64E-39	yellow
EIF3B	8662	eukaryotic translation initiation factor 3, subunit 9 eta, 116kDa (EIF3S9), transcript variant 1, mRNA.	-0.09	7.70	9.85E-10	2.43E-09	yellow
EIF3D	8664	eukaryotic translation initiation factor 3, subunit 7 zeta, 66/67kDa (EIF3S7), mRNA.	-0.07	6.87	4.54E-05	8.13E-05	yellow

EIF4G1	1981	eukaryotic translation initiation factor 4 gamma, 1 (EIF4G1), transcript variant 5, mRNA.	0.03	11.21	1.70E-02	2.35E-02	yellow
ELAC2	60528	elaC homolog 2 (E. coli) (ELAC2), mRNA.	0.09	8.11	4.83E-09	1.15E-08	yellow
EMID1	129080	EMI domain containing 1 (EMID1), mRNA.	0.06	7.25	3.38E-04	5.62E-04	yellow
ENG	2022	endoglin (Osler-Rendu-Weber syndrome 1) (ENG), mRNA.	-0.04	6.35	4.66E-03	6.87E-03	yellow
ENOPH1	58478	enolase-phosphatase 1 (ENOPH1), mRNA.	0.12	7.15	7.71E-11	2.04E-10	yellow
EP300	2033	E1A binding protein p300 (EP300), mRNA.	-0.05	8.21	1.35E-04	2.33E-04	yellow
EP400	57634	E1A binding protein p400 (EP400), mRNA.	0.25	10.63	2.05E-31	1.41E-30	yellow
ERAL1	26284	Era G-protein-like 1 (E. coli) (ERAL1), mRNA.	0.07	7.42	6.78E-09	1.60E-08	yellow
ESRRA	2101	estrogen-related receptor alpha (ESRRA), mRNA.	-0.03	5.89	4.50E-02	5.91E-02	yellow
ESYT1	23344	family with sequence similarity 62 (C2 domain containing), member A (FAM62A), mRNA.	-0.05	8.25	5.30E-03	7.77E-03	yellow
EXOSC3	51010	exosome component 3 (EXOSC3), transcript variant 2, mRNA.	0.11	6.42	1.58E-11	4.33E-11	yellow
FAM26F	441168	hypothetical protein LOC441168 (LOC441168), mRNA.	-0.55	8.02	6.11E-58	1.46E-56	yellow

FBXW4	6468	F-box and WD repeat domain containing 4 (FBXW4), mRNA.	-0.18	9.88	1.69E-33	1.26E-32	yellow
FERMT3	83706	UNC-112 related protein 2 (URP2), transcript variant URP2LF, mRNA.	0.01	9.23	3.03E-01	3.47E-01	yellow
FHOD1	29109	formin homology 2 domain containing 1 (FHOD1), mRNA.	1.04	9.22	2.05E-90	4.52E-88	yellow
FLNA	2316	filamin A, alpha (actin binding protein 280) (FLNA), mRNA.	-0.11	7.51	1.41E-20	6.19E-20	yellow
FTH1	2495	ferritin, heavy polypeptide 1 (FTH1), mRNA.	-0.27	6.71	1.11E-22	5.39E-22	yellow
G6PD	2539	glucose-6-phosphate dehydrogenase (G6PD), nuclear gene encoding mitochondrial protein, transcript variant 2, mRNA.	-0.12	7.16	1.57E-15	5.34E-15	yellow
GAD1	2571	glutamate decarboxylase 1 (brain, 67kDa) (GAD1), transcript variant GAD25, mRNA.	0.59	7.89	3.73E-48	5.58E-47	yellow
GBF1	8729	golgi-specific brefeldin A resistance factor 1 (GBF1), mRNA.	0.04	10.77	6.48E-03	9.43E-03	yellow
GCNT1	2650	glucosaminyl (N-acetyl) transferase 1, core 2 (beta-1,6-N-acetylglucosaminyltransferase) (GCNT1), mRNA.	-0.55	6.15	2.41E-39	2.35E-38	yellow
GDPD5	81544	glycerophosphodiester phosphodiesterase domain containing 5 (GDPD5), mRNA.	-0.09	10.38	6.48E-14	2.03E-13	yellow

GLA	2717	galactosidase, alpha (GLA), mRNA.	0.03	10.49	1.32E-02	1.85E-02	yellow
GLIPR1	11010	GLI pathogenesis-related 1 (glioma) (GLIPR1), mRNA.	-0.03	9.83	2.02E-02	2.76E-02	yellow
GLRX5	51218	glutaredoxin 5 homolog (S. cerevisiae) (GLRX5), mRNA.	0.01	12.58	3.54E-01	3.99E-01	yellow
GLT8D1	55830	glycosyltransferase 8 domain containing 1 (GLT8D1), transcript variant 1, mRNA.	0.08	7.88	2.75E-10	7.03E-10	yellow
GMIP	51291	GEM interacting protein (GMIP), mRNA.	0.85	6.23	2.93E-74	1.67E-72	yellow
GNAI2	2771	guanine nucleotide binding protein (G protein), alpha inhibiting activity polypeptide 2 (GNAI2), mRNA.	0.04	6.64	5.31E-02	6.90E-02	yellow
GOLGA3	2802	golgi autoantigen, golgin subfamily a, 3 (GOLGA3), mRNA.	-0.08	9.55	4.98E-06	9.67E-06	yellow
GRINA	2907	glutamate receptor, ionotropic, N-methyl D-asparate-associated protein 1 (glutamate binding) (GRINA), transcript variant 1, mRNA.	-0.01	6.95	3.58E-01	4.04E-01	yellow
GRSF1	2926	G-rich RNA sequence binding factor 1 (GRSF1), mRNA.	-0.01	7.81	6.44E-01	6.83E-01	yellow
H2AFX	729063	PREDICTED: similar to H2A histone family, member X (LOC729063), mRNA.	0.03	9.13	1.58E-01	1.91E-01	yellow
HACL1	26061	2-hydroxyacyl-CoA lyase 1 (HACL1), mRNA.	0.07	11.44	5.76E-08	1.28E-07	yellow

HELZ	9931	helicase with zinc finger (HELZ), mRNA.	0.06	7.75	7.15E-03	1.04E-02	yellow
HIGD1A	25994	HIG1 domain family, member 1A (HIGD1A), mRNA.	-0.20	9.26	8.94E-30	5.75E-29	yellow
HJURP	55355	hypothetical protein DKFZp762E1312 (DKFZp762E1312), mRNA.	-0.09	9.30	5.07E-13	1.51E-12	yellow
HMGN5	79366	nucleosomal binding protein 1 (NSBP1), mRNA.	0.06	8.24	5.53E-04	9.02E-04	yellow
HN1	51155	hematological and neurological expressed 1 (HN1), transcript variant 3, mRNA.	1.03	7.98	6.80E-96	2.30E-93	yellow
HSP90AB1	3326	heat shock protein 90kDa alpha (cytosolic), class B member 1 (HSP90AB1), mRNA.	-0.17	7.70	7.08E-31	4.72E-30	yellow
ING1	3621	inhibitor of growth family, member 1 (ING1), transcript variant 2, mRNA.	0.00	10.78	7.38E-01	7.69E-01	yellow
INTS3	65123	integrator complex subunit 3 (INTS3), mRNA.	-0.04	7.66	1.90E-03	2.93E-03	yellow
INTS5	80789	integrator complex subunit 5 (INTS5), mRNA.	-0.01	5.64	3.66E-01	4.12E-01	yellow
IPO13	9670	importin 13 (IPO13), mRNA.	1.70	7.18	3.90E-114	4.95E-111	yellow
IPO4	79711	importin 4 (IPO4), mRNA.	-0.03	6.55	3.53E-02	4.69E-02	yellow
IQSEC1	9922	IQ motif and Sec7 domain 1 (IQSEC1), mRNA.	0.01	7.81	7.13E-01	7.47E-01	yellow
IRAK1	3654	interleukin-1 receptor-associated kinase 1 (IRAK1), transcript variant 2, mRNA.	-0.07	6.98	2.99E-05	5.47E-05	yellow

IRF3	3661	interferon regulatory factor 3 (IRF3), mRNA.	-0.04	7.18	1.12E-02	1.59E-02	yellow
ISOC2	79763	isochorismatase domain containing 2 (ISOC2), mRNA.	0.03	8.32	1.98E-02	2.72E-02	yellow
ITFG2	55846	integrin alpha FG-GAP repeat containing 2 (ITFG2), mRNA.	0.13	9.38	3.66E-14	1.16E-13	yellow
ITFG3	83986	integrin alpha FG-GAP repeat containing 3 (ITFG3), mRNA.	0.00	7.69	7.81E-01	8.10E-01	yellow
KANK2	25959	ankyrin repeat domain 25 (ANKRD25), mRNA.	-0.08	5.92	4.89E-06	9.51E-06	yellow
KANSL1	284058	KIAA1267 (KIAA1267), mRNA.	0.16	7.25	1.71E-26	9.83E-26	yellow
KAT2B	8850	p300/CBP-associated factor (PCAF), mRNA.	-0.12	8.70	8.35E-13	2.46E-12	yellow
KCTD20	222658	potassium channel tetramerisation domain containing 20 (KCTD20), mRNA.	0.01	9.02	5.56E-01	6.00E-01	yellow
KIAA0100	9703	KIAA0100 (KIAA0100), mRNA.	-0.05	8.79	2.93E-05	5.37E-05	yellow
KIAA2013	90231	KIAA2013 (KIAA2013), mRNA.	0.01	9.49	5.57E-01	6.00E-01	yellow
KIF21A	55605	kinesin family member 21A (KIF21A), mRNA.	-0.34	8.79	5.66E-24	2.92E-23	yellow
KIF4A	24137	kinesin family member 4A (KIF4A), mRNA.	0.01	8.09	4.38E-01	4.84E-01	yellow
LACTB	114294	lactamase, beta (LACTB), nuclear gene encoding mitochondrial protein, transcript variant 1, mRNA.	-0.10	11.28	2.60E-13	7.88E-13	yellow

LARP4B	23185	La ribonucleoprotein domain family, member 5 (LARP5), mRNA.	0.05	8.66	1.24E-02	1.75E-02	yellow
LGALS9	3965	lectin, galactoside-binding, soluble, 9 (galectin 9) (LGALS9), transcript variant short, mRNA.	0.10	6.48	1.07E-09	2.63E-09	yellow
LIG1	3978	ligase I, DNA, ATP-dependent (LIG1), mRNA.	0.12	6.77	3.02E-12	8.61E-12	yellow
LMF2	91289	transmembrane protein 112B (TMEM112B), mRNA.	0.09	7.51	1.53E-09	3.75E-09	yellow
LONP1	9361	lon peptidase 1, mitochondrial (LONP1), nuclear gene encoding mitochondrial protein, mRNA.	-0.04	6.92	1.18E-02	1.67E-02	yellow
LSS	4047	lanosterol synthase (2,3-oxidosqualene-lanosterol cyclase) (LSS), transcript variant 1, mRNA.	0.10	8.23	7.43E-10	1.85E-09	yellow
LYRM1	57149	LYR motif containing 1 (LYRM1), mRNA.	-0.04	9.15	5.06E-04	8.27E-04	yellow
MAN2C1	4123	mannosidase, alpha, class 2C, member 1 (MAN2C1), mRNA.	0.09	7.91	4.10E-09	9.80E-09	yellow
MAP1S	55201	microtubule-associated protein 1S (MAP1S), mRNA.	0.02	9.40	3.56E-01	4.02E-01	yellow
MAP3K5	4217	mitogen-activated protein kinase kinase kinase 5 (MAP3K5), mRNA.	-0.09	7.49	2.93E-07	6.17E-07	yellow

MBTPS1	8720	membrane-bound transcription factor peptidase, site 1 (MBTPS1), transcript variant 1, mRNA.	1.08	8.63	4.76E-92	1.15E-89	yellow
MCM2	4171	MCM2 minichromosome maintenance deficient 2, mitotin (S. cerevisiae) (MCM2), mRNA.	0.20	8.41	6.77E-24	3.48E-23	yellow
MDH2	4191	malate dehydrogenase 2, NAD (mitochondrial) (MDH2), mRNA.	-0.13	8.99	1.42E-16	5.14E-16	yellow
MED24	9862	thyroid hormone receptor associated protein 4 (THRAP4), transcript variant 2, mRNA.	0.08	9.35	7.43E-11	1.96E-10	yellow
MED25	81857	mediator of RNA polymerase II transcription, subunit 25 homolog (S. cerevisiae) (MED25), mRNA.	0.00	8.66	8.64E-01	8.85E-01	yellow
MED28	80306	mediator of RNA polymerase II transcription, subunit 28 homolog (S. cerevisiae) (MED28), mRNA.	0.06	11.17	5.54E-04	9.03E-04	yellow
MESDC1	59274	mesoderm development candidate 1 (MESDC1), mRNA.	0.02	10.91	1.06E-01	1.32E-01	yellow
MFHAS1	9258	malignant fibrous histiocytoma amplified sequence 1 (MFHAS1), mRNA.	-0.05	10.76	1.93E-04	3.27E-04	yellow

MGAT1	4245	mannosyl (alpha-1,3-)-glycoprotein beta-1,2-N-acetylglucosaminyltransferase (MGAT1), mRNA.	-0.04	8.68	6.17E-03	8.99E-03	yellow
MGLL	11343	monoglyceride lipase (MGLL), transcript variant 2, mRNA.	0.32	8.09	4.51E-57	1.02E-55	yellow
MITD1	129531	MIT, microtubule interacting and transport, domain containing 1 (MITD1), mRNA.	-0.23	8.99	2.76E-27	1.64E-26	yellow
MKNK2	2872	MAP kinase interacting serine/threonine kinase 2 (MKNK2), transcript variant 2, mRNA.	0.90	8.48	7.41E-74	4.04E-72	yellow
MLF2	8079	myeloid leukemia factor 2 (MLF2), mRNA.	-0.21	6.39	2.18E-32	1.58E-31	yellow
MOV10	4343	Mov10, Moloney leukemia virus 10, homolog (mouse) (MOV10), mRNA.	0.09	8.20	2.14E-07	4.57E-07	yellow
MPRIP	23164	myosin phosphatase-Rho interacting protein (M-RIP), transcript variant 1, mRNA.	-0.03	8.74	4.58E-02	6.00E-02	yellow
MRPL1	65008	mitochondrial ribosomal protein L1 (MRPL1), nuclear gene encoding mitochondrial protein, mRNA.	0.00	11.10	9.35E-01	9.45E-01	yellow
MRPL2	51069	mitochondrial ribosomal protein L2 (MRPL2), nuclear gene encoding mitochondrial protein, mRNA.	0.05	8.26	7.86E-03	1.13E-02	yellow

MRPL32	64983	mitochondrial ribosomal protein L32 (MRPL32), nuclear gene encoding mitochondrial protein, mRNA.	-0.07	6.37	5.25E-04	8.58E-04	yellow
MRPL38	64978	mitochondrial ribosomal protein L38 (MRPL38), nuclear gene encoding mitochondrial protein, mRNA.	-0.09	9.21	1.02E-08	2.37E-08	yellow
MRPL43	84545	mitochondrial ribosomal protein L43 (MRPL43), nuclear gene encoding mitochondrial protein, transcript variant 4, mRNA.	-0.22	6.15	8.03E-26	4.50E-25	yellow
MSN	4478	moesin (MSN), mRNA.	0.33	9.56	1.23E-51	2.06E-50	yellow
MTA1	9112	metastasis associated 1 (MTA1), mRNA.	-0.24	6.51	3.16E-32	2.27E-31	yellow
MTA2	9219	metastasis associated 1 family, member 2 (MTA2), mRNA.	-0.03	7.35	4.14E-02	5.45E-02	yellow
MTF1	4520	metal-regulatory transcription factor 1 (MTF1), mRNA.	0.05	8.06	8.76E-03	1.26E-02	yellow
MTHFD1	4522	methylenetetrahydrofolate dehydrogenase (NADP+ dependent) 1, methenyltetrahydrofolate cyclohydrolase, formyltetrahydrofolate synthetase (MTHFD1), mRNA.	-0.02	8.02	1.49E-01	1.81E-01	yellow

MTHFD2	10797	methylenetetrahydrofolate dehydrogenase (NADP+ dependent) 2, methenyltetrahydrofolate cyclohydrolase (MTHFD2), nuclear gene encoding mitochondrial protein, transcript variant 1, mRNA.	0.16	8.72	1.33E-29	8.50E-29	yellow
MTOR	2475	FK506 binding protein 12-rapamycin associated protein 1 (FRAP1), mRNA.	0.03	7.22	1.89E-02	2.60E-02	yellow
MYO1C	4641	myosin IC (MYO1C), transcript variant 2, mRNA.	0.00	9.00	8.78E-01	8.97E-01	yellow
MYO1G	64005	myosin IG (MYO1G), mRNA.	-0.18	7.52	1.30E-17	4.98E-17	yellow
N4BP2L2	10443	phosphonoformate immuno-associated protein 5 (PFAAP5), mRNA.	0.01	9.84	3.79E-01	4.24E-01	yellow
NA	7919	HLA-B associated transcript 1 (BAT1), transcript variant 1, mRNA.	0.15	6.48	4.85E-21	2.16E-20	yellow
NA	51523	CXXC finger 5 (CXXC5), mRNA.	-0.05	9.66	1.62E-04	2.76E-04	yellow
NA	8106	poly(A) binding protein, nuclear 1 (PABPN1), mRNA.	-0.05	7.18	3.42E-03	5.11E-03	yellow

NA	2073	excision repair cross-complementing rodent repair deficiency, complementation group 5 (xeroderma pigmentosum, complementation group G (Cockayne syndrome)) (ERCC5), mRNA.	0.01	10.61	3.32E-01	3.77E-01	yellow
NABP2	79035	oligonucleotide/oligosaccharide-binding fold containing 2B (OBFC2B), mRNA.	0.12	5.61	8.38E-13	2.47E-12	yellow
NADSYN1	55191	NAD synthetase 1 (NADSYN1), mRNA.	0.03	6.91	8.11E-02	1.03E-01	yellow
NCAPD3	23310	non-SMC condensin II complex, subunit D3 (NCAPD3), mRNA.	0.14	8.02	1.46E-22	7.00E-22	yellow
NCOA5	57727	nuclear receptor coactivator 5 (NCOA5), mRNA.	-0.04	6.75	2.35E-02	3.20E-02	yellow
NCOR2	9612	nuclear receptor co-repressor 2 (NCOR2), transcript variant 2, mRNA.	0.14	5.86	3.47E-15	1.16E-14	yellow
NDFIP2	54602	Nedd4 family interacting protein 2 (NDFIP2), mRNA.	-0.16	7.93	4.59E-27	2.70E-26	yellow
NDRG3	57446	NDRG family member 3 (NDRG3), transcript variant 1, mRNA.	0.07	6.81	2.73E-05	5.01E-05	yellow

NDUFA6	4700	NADH dehydrogenase (ubiquinone) 1 alpha subcomplex, 6, 14kDa (NDUFA6), nuclear gene encoding mitochondrial protein, mRNA.	0.27	8.77	2.05E-45	2.70E-44	yellow
NDUFB5	4711	NADH dehydrogenase (ubiquinone) 1 beta subcomplex, 5, 16kDa (NDUFB5), nuclear gene encoding mitochondrial protein, mRNA.	0.01	6.10	6.74E-01	7.10E-01	yellow
NELFB	25920	PREDICTED: cofactor of BRCA1 (COBRA1), mRNA.	-0.06	7.21	8.89E-05	1.56E-04	yellow
NMT2	9397	N-myristoyltransferase 2 (NMT2), mRNA.	0.98	6.28	1.25E-60	3.56E-59	yellow
NOC2L	26155	nucleolar complex associated 2 homolog (S. cerevisiae) (NOC2L), mRNA.	0.10	6.45	1.96E-09	4.75E-09	yellow
NPC2	10577	Niemann-Pick disease, type C2 (NPC2), mRNA.	0.09	6.13	2.16E-09	5.23E-09	yellow
NPLOC4	55666	nuclear protein localization 4 homolog (S. cerevisiae) (NPLOC4), mRNA.	0.01	9.13	6.77E-01	7.13E-01	yellow
NQO1	1728	NAD(P)H dehydrogenase, quinone 1 (NQO1), transcript variant 2, mRNA.	-0.12	8.91	1.41E-08	3.25E-08	yellow
NRBP1	29959	nuclear receptor binding protein 1 (NRBP1), mRNA.	0.23	9.05	5.20E-35	4.15E-34	yellow

NRROS	375387	leucine rich repeat containing 33 (LRRC33), mRNA.	0.05	7.04	2.22E-04	3.76E-04	yellow
NT5DC2	64943	5'-nucleotidase domain containing 2 (NT5DC2), mRNA.	-0.11	7.74	1.21E-11	3.33E-11	yellow
NUP188	23511	nucleoporin 188kDa (NUP188), mRNA.	-0.07	9.77	9.23E-10	2.28E-09	yellow
NUP210	23225	nucleoporin 210kDa (NUP210), mRNA.	0.02	8.75	1.37E-01	1.67E-01	yellow
NUP214	8021	nucleoporin 214kDa (NUP214), mRNA.	0.26	8.84	4.12E-40	4.16E-39	yellow
NUP37	79023	nucleoporin 37kDa (NUP37), mRNA.	0.05	10.62	2.91E-02	3.90E-02	yellow
NUP93	9688	nucleoporin 93kDa (NUP93), mRNA.	0.39	7.37	1.07E-55	2.21E-54	yellow
NXF1	10482	nuclear RNA export factor 1 (NXF1), transcript variant 2, mRNA.	-0.09	8.70	1.79E-12	5.16E-12	yellow
OGDH	4967	oxoglutarate (alpha-ketoglutarate) dehydrogenase (lipoamide) (OGDH), nuclear gene encoding mitochondrial protein, transcript variant 1, mRNA.	-0.07	8.51	5.92E-06	1.14E-05	yellow
PA2G4	5036	proliferation-associated 2G4, 38kDa (PA2G4), mRNA.	-0.09	7.58	1.07E-06	2.18E-06	yellow
PARVG	64098	parvin, gamma (PARVG), mRNA.	0.20	6.85	3.41E-16	1.20E-15	yellow
PCCB	5096	propionyl Coenzyme A carboxylase, beta polypeptide (PCCB), mRNA.	-0.06	5.67	4.90E-03	7.21E-03	yellow
PCID2	55795	PCI domain containing 2 (PCID2), mRNA.	-0.02	9.16	1.83E-01	2.18E-01	yellow

PDHB	5162	pyruvate dehydrogenase (lipoamide) beta (PDHB), mRNA.	0.06	10.43	3.93E-05	7.09E-05	yellow
PEF1	553115	penta-EF-hand domain containing 1 (PEF1), mRNA.	-0.06	8.79	1.42E-06	2.86E-06	yellow
PELP1	27043	proline, glutamic acid and leucine rich protein 1 (PELP1), mRNA.	0.08	7.51	8.93E-07	1.83E-06	yellow
PFAS	5198	phosphoribosylformylglycinamidine synthase (FGAR amidotransferase) (PFAS), mRNA.	0.03	7.61	7.37E-02	9.40E-02	yellow
PFKFB4	5210	6-phosphofructo-2-kinase/fructose-2,6-biphosphatase 4 (PFKFB4), mRNA.	0.63	8.36	7.69E-78	5.66E-76	yellow
PFKL	5211	phosphofructokinase, liver (PFKL), transcript variant 2, mRNA.	0.12	6.78	2.29E-10	5.89E-10	yellow
PFKP	5214	phosphofructokinase, platelet (PFKP), mRNA.	0.01	7.03	6.21E-01	6.61E-01	yellow
PGRMC1	10857	progesterone receptor membrane component 1 (PGRMC1), mRNA.	-0.12	10.01	1.02E-21	4.73E-21	yellow
PHYH	5264	phytanoyl-CoA 2-hydroxylase (PHYH), transcript variant 2, mRNA.	-0.03	7.73	3.60E-02	4.78E-02	yellow
PITPNM1	9600	phosphatidylinositol transfer protein, membrane-associated 1 (PITPNM1), mRNA.	-0.46	10.31	3.25E-78	2.46E-76	yellow
PITRM1	10531	pitrilysin metallopeptidase 1 (PITRM1), mRNA.	-0.14	7.98	1.98E-15	6.67E-15	yellow

PKM	5315	pyruvate kinase, muscle (PKM2), transcript variant 2, mRNA.	0.36	8.28	1.88E-44	2.33E-43	yellow
PKN1	5585	protein kinase N1 (PKN1), transcript variant 2, mRNA.	0.03	8.52	6.26E-02	8.06E-02	yellow
PLA2G4C	8605	phospholipase A2, group IVC (cytosolic, calcium-independent) (PLA2G4C), mRNA.	0.00	12.42	7.86E-01	8.14E-01	yellow
PLCB2	5330	phospholipase C, beta 2 (PLCB2), mRNA.	-0.10	8.15	8.05E-08	1.77E-07	yellow
PLCXD1	55344	phosphatidylinositol-specific phospholipase C, X domain containing 1 (PLCXD1), mRNA.	-0.07	8.06	3.35E-04	5.56E-04	yellow
PLEKHO2	80301	pleckstrin homology domain containing, family Q member 1 (PLEKHQ1), mRNA.	0.13	9.50	5.41E-19	2.21E-18	yellow
PMPCB	9512	peptidase (mitochondrial processing) beta (PMPCB), nuclear gene encoding mitochondrial protein, mRNA.	-0.05	9.41	8.78E-07	1.80E-06	yellow
PNPLA6	10908	patatin-like phospholipase domain containing 6 (PNPLA6), mRNA.	-0.02	6.68	4.36E-01	4.83E-01	yellow
PNPO	55163	pyridoxamine 5'-phosphate oxidase (PNPO), mRNA.	0.17	8.02	4.92E-22	2.31E-21	yellow
POLD1	5424	polymerase (DNA directed), delta 1, catalytic subunit 125kDa (POLD1), mRNA.	-0.59	9.33	2.60E-83	2.80E-81	yellow

POLR1D	51082	polymerase (RNA) I polypeptide D, 16kDa (POLR1D), transcript variant 1, mRNA.	0.05	9.89	1.45E-02	2.02E-02	yellow
POLRMT	5442	polymerase (RNA) mitochondrial (DNA directed) (POLRMT), nuclear gene encoding mitochondrial protein, mRNA.	0.03	8.57	2.48E-02	3.36E-02	yellow
PPA1	5464	pyrophosphatase (inorganic) 1 (PPA1), mRNA.	0.01	8.26	6.21E-01	6.61E-01	yellow
PPME1	51400	protein phosphatase methylesterase 1 (PPME1), mRNA.	0.05	9.58	4.09E-06	8.00E-06	yellow
PPP1R10	5514	protein phosphatase 1, regulatory (inhibitor) subunit 10 (PPP1R10), mRNA.	0.48	5.84	7.51E-54	1.38E-52	yellow
PPP2R1A	5518	protein phosphatase 2 (formerly 2A), regulatory subunit A , alpha isoform (PPP2R1A), mRNA.	0.00	7.72	7.97E-01	8.23E-01	yellow
PPP2R3B	28227	protein phosphatase 2 (formerly 2A), regulatory subunit B", beta (PPP2R3B), transcript variant 2, mRNA.	-0.11	7.32	9.16E-11	2.40E-10	yellow
PPP6R1	22870	SAPS domain family, member 1 (SAPS1), mRNA.	-0.02	8.64	2.98E-01	3.43E-01	yellow
PREB	10113	prolactin regulatory element binding (PREB), mRNA.	-0.07	9.30	5.99E-06	1.16E-05	yellow
PRKX	5613	protein kinase, X-linked (PRKX), mRNA.	0.01	9.59	1.71E-01	2.05E-01	yellow

PRPF3	9129	PRP3 pre-mRNA processing factor 3 homolog (S. cerevisiae) (PRPF3), mRNA.	0.11	8.03	9.13E-10	2.26E-09	yellow
PRPF31	26121	PRP31 pre-mRNA processing factor 31 homolog (S. cerevisiae) (PRPF31), mRNA.	0.08	8.66	8.03E-09	1.88E-08	yellow
PRPF8	10594	PRP8 pre-mRNA processing factor 8 homolog (S. cerevisiae) (PRPF8), mRNA.	0.06	8.07	3.36E-05	6.11E-05	yellow
PRRC2A	7916	HLA-B associated transcript 2 (BAT2), transcript variant 2, mRNA.	0.04	8.88	4.47E-03	6.62E-03	yellow
PSMD1	5707	proteasome (prosome, macropain) 26S subunit, non-ATPase, 1 (PSMD1), mRNA.	0.11	8.96	1.44E-13	4.42E-13	yellow
PSMD2	5708	proteasome (prosome, macropain) 26S subunit, non-ATPase, 2 (PSMD2), mRNA.	0.10	7.40	1.11E-10	2.89E-10	yellow
PSMF1	9491	proteasome (prosome, macropain) inhibitor subunit 1 (PI31) (PSMF1), transcript variant 1, mRNA.	-0.18	8.04	8.64E-33	6.32E-32	yellow
PTGR1	22949	leukotriene B4 12-hydroxydehydrogenase (LTB4DH), mRNA.	0.15	7.71	7.41E-13	2.19E-12	yellow
PTMS	5763	parathymosin (PTMS), mRNA.	0.10	8.37	2.03E-08	4.62E-08	yellow
PTOV1	53635	prostate tumor overexpressed gene 1 (PTOV1), mRNA.	0.12	6.18	1.32E-14	4.28E-14	yellow

PTPN23	25930	protein tyrosine phosphatase, non-receptor type 23 (PTPN23), mRNA.	-0.06	9.07	5.40E-05	9.61E-05	yellow
PYCR1	5831	pyrroline-5-carboxylate reductase 1 (PYCR1), transcript variant 1, mRNA.	-0.01	7.79	3.49E-01	3.94E-01	yellow
PYCR2	29920	pyrroline-5-carboxylate reductase family, member 2 (PYCR2), mRNA.	0.01	7.33	7.18E-01	7.51E-01	yellow
PYGB	5834	phosphorylase, glycogen; brain (PYGB), mRNA.	0.03	7.63	7.55E-02	9.62E-02	yellow
PYGO2	90780	pygopus homolog 2 (Drosophila) (PYGO2), mRNA.	0.01	10.11	7.36E-01	7.68E-01	yellow
R3HDM4	91300	chromosome 19 open reading frame 22 (C19orf22), mRNA.	0.01	7.36	7.10E-01	7.44E-01	yellow
RAB20	55647	RAB20, member RAS oncogene family (RAB20), mRNA.	-0.30	8.96	5.80E-32	4.10E-31	yellow
RAB5C	5878	RAB5C, member RAS oncogene family (RAB5C), transcript variant 1, mRNA.	-0.04	7.43	2.21E-02	3.01E-02	yellow
RABL6	55684	chromosome 9 open reading frame 86 (C9orf86), mRNA.	0.05	9.62	4.54E-03	6.71E-03	yellow
RAD23A	5886	RAD23 homolog A (S. cerevisiae) (RAD23A), mRNA.	-0.09	6.79	8.37E-04	1.34E-03	yellow
RAPGEF6	51735	Rap guanine nucleotide exchange factor (GEF) 6 (RAPGEF6), mRNA.	0.07	9.57	2.35E-07	5.01E-07	yellow

RAVER1	125950	ribonucleoprotein, PTB-binding 1 (RAVER1), mRNA.	-0.08	6.78	2.19E-07	4.68E-07	yellow
RBM10	8241	RNA binding motif protein 10 (RBM10), transcript variant 1, mRNA.	-0.06	8.50	1.00E-02	1.43E-02	yellow
RBM42	79171	hypothetical protein MGC10433 (MGC10433), mRNA.	0.31	6.43	1.66E-34	1.30E-33	yellow
RCC1	751867	regulator of chromosome condensation 1 (SNHG3-RCC1), transcript variant 1, mRNA.	-0.14	6.04	5.31E-12	1.49E-11	yellow
RECQL4	9401	RecQ protein-like 4 (RECQL4), mRNA.	0.20	8.33	1.96E-13	5.98E-13	yellow
RFX1	5989	regulatory factor X, 1 (influences HLA class II expression) (RFX1), mRNA.	-0.06	7.33	2.22E-07	4.73E-07	yellow
RGS19	10287	regulator of G-protein signalling 19 (RGS19), transcript variant 2, mRNA.	-0.16	6.79	2.74E-27	1.63E-26	yellow
RHOT2	89941	ras homolog gene family, member T2 (RHOT2), mRNA.	-0.19	8.27	5.56E-36	4.66E-35	yellow
RNF144A	9781	ring finger protein 144 (RNF144), mRNA.	-0.04	9.36	6.82E-02	8.75E-02	yellow
RNF40	9810	ring finger protein 40 (RNF40), mRNA.	-0.01	6.36	7.28E-01	7.60E-01	yellow
RORA	6095	RAR-related orphan receptor A (RORA), transcript variant 2, mRNA.	0.07	7.82	1.13E-04	1.95E-04	yellow
RPL29	6159	ribosomal protein L29 (RPL29), mRNA.	0.06	8.10	2.07E-05	3.83E-05	yellow

RPL9	6133	ribosomal protein L9 (RPL9), transcript variant 2, mRNA.	-0.06	8.67	1.08E-06	2.19E-06	yellow
RPS6KA4	8986	ribosomal protein S6 kinase, 90kDa, polypeptide 4 (RPS6KA4), transcript variant 1, mRNA.	0.03	9.03	1.94E-03	2.98E-03	yellow
RPUSD1	113000	RNA pseudouridylate synthase domain containing 1 (RPUSD1), mRNA.	0.21	7.43	1.08E-22	5.25E-22	yellow
RRP12	23223	ribosomal RNA processing 12 homolog (S. cerevisiae) (RRP12), mRNA.	-0.04	8.98	1.40E-02	1.95E-02	yellow
SAC3D1	29901	SAC3 domain containing 1 (SAC3D1), mRNA.	0.04	7.14	6.02E-02	7.77E-02	yellow
SAFB2	9667	scaffold attachment factor B2 (SAFB2), mRNA.	-0.09	9.30	1.64E-12	4.76E-12	yellow
SAMD1	90378	sterile alpha motif domain containing 1 (SAMD1), mRNA.	-0.31	7.49	4.18E-54	7.95E-53	yellow
SAP30L	79685	SAP30-like (SAP30L), mRNA.	-0.04	9.14	2.55E-03	3.86E-03	yellow
SCAF4	57466	splicing factor, arginine/serine-rich 15 (SFRS15), mRNA.	0.50	6.39	1.22E-51	2.05E-50	yellow
SCO1	6341	SCO cytochrome oxidase deficient homolog 1 (yeast) (SCO1), nuclear gene encoding mitochondrial protein, mRNA.	0.05	10.53	1.03E-06	2.09E-06	yellow
SDF4	51150	stromal cell derived factor 4 (SDF4), mRNA.	-0.13	8.38	4.80E-16	1.68E-15	yellow

SEMA4B	10509	sema domain, immunoglobulin domain (Ig), transmembrane domain (TM) and short cytoplasmic domain, (semaphorin) 4B (SEMA4B), transcript variant 1, mRNA.	-0.20	6.75	1.09E-31	7.59E-31	yellow
SEPN1	57190	selenoprotein N, 1 (SEPN1), transcript variant 2, mRNA.	-0.03	9.92	4.83E-03	7.10E-03	yellow
SETD1A	9739	SET domain containing 1A (SETD1A), mRNA.	-0.07	8.86	5.03E-10	1.26E-09	yellow
SF3A2	8175	splicing factor 3a, subunit 2, 66kDa (SF3A2), mRNA.	-0.05	6.19	1.40E-03	2.18E-03	yellow
SF3B4	10262	splicing factor 3b, subunit 4, 49kDa (SF3B4), mRNA.	0.10	8.47	4.03E-08	8.99E-08	yellow
SH3BP1	23616	SH3-domain binding protein 1 (SH3BP1), mRNA.	-0.15	7.00	1.44E-17	5.49E-17	yellow
SH3TC1	54436	SH3 domain and tetratricopeptide repeats 1 (SH3TC1), mRNA.	-0.04	9.31	2.12E-03	3.24E-03	yellow
SHC1	6464	SHC (Src homology 2 domain containing) transforming protein 1 (SHC1), transcript variant 2, mRNA.	-0.08	7.65	2.02E-08	4.60E-08	yellow
SIGMAR1	10280	opioid receptor, sigma 1 (OPRS1), transcript variant 3, mRNA.	-0.08	7.27	4.33E-09	1.03E-08	yellow
SKP2	6502	S-phase kinase-associated protein 2 (p45) (SKP2), transcript variant 2, mRNA.	0.35	7.08	6.51E-41	6.80E-40	yellow

SLC17A5	26503	solute carrier family 17 (anion/sugar transporter), member 5 (SLC17A5), mRNA.	0.04	7.49	2.73E-02	3.67E-02	yellow
SLC17A9	63910	chromosome 20 open reading frame 59 (C20orf59), mRNA.	0.02	8.45	1.50E-01	1.82E-01	yellow
SLC1A5	6510	solute carrier family 1 (neutral amino acid transporter), member 5 (SLC1A5), mRNA.	0.13	7.02	6.14E-12	1.72E-11	yellow
SLC25A22	79751	solute carrier family 25 (mitochondrial carrier: glutamate), member 22 (SLC25A22), mRNA.	0.01	10.62	6.60E-01	6.97E-01	yellow
SLC25A39	51629	solute carrier family 25, member 39 (SLC25A39), mRNA.	-0.12	7.79	2.68E-08	6.07E-08	yellow
SLC25A6	293	solute carrier family 25 (mitochondrial carrier; adenine nucleotide translocator), member 6 (SLC25A6), mRNA.	-0.18	7.75	1.83E-33	1.37E-32	yellow
SLC29A1	2030	solute carrier family 29 (nucleoside transporters), member 1 (SLC29A1), nuclear gene encoding mitochondrial protein, transcript variant 3, mRNA.	-0.10	5.55	7.64E-08	1.68E-07	yellow

SLC2A6	11182	solute carrier family 2 (facilitated glucose transporter), member 6 (SLC2A6), mRNA.	-0.02	9.72	7.46E-02	9.51E-02	yellow
SLC38A5	92745	solute carrier family 38, member 5 (SLC38A5), mRNA.	0.13	6.00	1.67E-16	5.99E-16	yellow
SLC4A2	6522	solute carrier family 4, anion exchanger, member 2 (erythrocyte membrane protein band 3-like 1) (SLC4A2), mRNA.	-0.01	7.32	4.37E-01	4.84E-01	yellow
SMAD3	4088	SMAD family member 3 (SMAD3), mRNA.	0.21	7.18	4.63E-23	2.28E-22	yellow
SMARCB1	6598	SWI/SNF related, matrix associated, actin dependent regulator of chromatin, subfamily b, member 1 (SMARCB1), transcript variant 1, mRNA.	0.05	8.08	3.03E-03	4.55E-03	yellow
SMARCC2	6601	SWI/SNF related, matrix associated, actin dependent regulator of chromatin, subfamily c, member 2 (SMARCC2), transcript variant 2, mRNA.	-0.04	10.51	4.04E-02	5.33E-02	yellow
SMG5	23381	Smg-5 homolog, nonsense mediated mRNA decay factor (C. elegans) (SMG5), mRNA.	0.09	9.86	2.74E-08	6.20E-08	yellow

SMG7	9887	Smg-7 homolog, nonsense mediated mRNA decay factor (C. elegans) (SMG7), transcript variant 3, mRNA.	-0.10	9.46	2.68E-12	7.64E-12	yellow
SMIM19	114926	chromosome 8 open reading frame 40 (C8orf40), mRNA.	-0.17	6.69	4.25E-27	2.50E-26	yellow
SNX11	29916	sorting nexin 11 (SNX11), transcript variant 2, mRNA.	-0.22	5.73	3.25E-22	1.53E-21	yellow
SNX3	8724	sorting nexin 3 (SNX3), mRNA.	-0.08	11.57	4.80E-05	8.58E-05	yellow
SOX18	54345	SRY (sex determining region Y)-box 18 (SOX18), mRNA.	0.00	7.97	8.90E-01	9.07E-01	yellow
SPTAN1	6709	spectrin, alpha, non-erythrocytic 1 (alpha-fodrin) (SPTAN1), mRNA.	-0.39	9.60	5.19E-44	6.29E-43	yellow
SREBF2	6721	sterol regulatory element binding transcription factor 2 (SREBF2), mRNA.	0.26	7.24	1.61E-35	1.32E-34	yellow
SRRM2	23524	serine/arginine repetitive matrix 2 (SRRM2), mRNA.	-0.05	7.73	9.39E-03	1.34E-02	yellow
SRRT	51593	ARS2 protein (ARS2), transcript variant 1, mRNA.	0.01	7.55	4.47E-01	4.94E-01	yellow
SSRP1	6749	structure specific recognition protein 1 (SSRP1), mRNA.	-0.15	7.41	2.51E-19	1.04E-18	yellow
STAT5A	6776	signal transducer and activator of transcription 5A (STAT5A), mRNA.	0.05	7.30	1.23E-03	1.93E-03	yellow
STXBP2	6813	syntaxin binding protein 2 (STXBP2), mRNA.	0.02	7.74	3.28E-01	3.73E-01	yellow
SUPT5H	6829	suppressor of Ty 5 homolog (S. cerevisiae) (SUPT5H), mRNA.	-0.09	9.77	4.43E-13	1.33E-12	yellow

SUSD6	9766	KIAA0247 (KIAA0247), mRNA.	0.02	8.56	1.69E-01	2.03E-01	yellow
SYNGR1	9145	synaptogyrin 1 (SYNGR1), transcript variant 1c, mRNA.	0.00	8.38	8.18E-01	8.43E-01	yellow
TACC3	10460	transforming, acidic coiled-coil containing protein 3 (TACC3), mRNA.	0.06	9.40	1.06E-07	2.32E-07	yellow
TAPBP	6892	TAP binding protein (tapasin) (TAPBP), transcript variant 1, mRNA.	-0.05	8.82	8.93E-05	1.56E-04	yellow
TAX1BP1	8887	Tax1 (human T-cell leukemia virus type I) binding protein 1 (TAX1BP1), transcript variant 1, mRNA.	-0.02	6.68	2.74E-01	3.17E-01	yellow
TBC1D10C	374403	TBC1 domain family, member 10C (TBC1D10C), mRNA.	-0.10	6.98	3.00E-11	8.07E-11	yellow
TBCB	1155	tubulin folding cofactor B (TBCB), mRNA.	0.02	8.91	2.33E-01	2.73E-01	yellow
TBCE	6905	tubulin folding cofactor E (TBCE), transcript variant 1, mRNA.	0.06	8.17	1.66E-07	3.57E-07	yellow
TCEAL4	79921	transcription elongation factor A (SII)-like 4 (TCEAL4), transcript variant 4, mRNA.	0.06	8.68	4.13E-04	6.81E-04	yellow
TCEAL8	90843	transcription elongation factor A (SII)-like 8 (TCEAL8), transcript variant 1, mRNA.	0.26	7.97	2.33E-34	1.81E-33	yellow

TCIRG1	10312	T-cell, immune regulator 1, ATPase, H+ transporting, lysosomal V0 subunit A3 (TCIRG1), transcript variant 1, mRNA.	0.10	8.79	8.09E-11	2.13E-10	yellow
TCTN1	79600	tectonic (FLJ21127), mRNA.	-0.16	7.29	1.66E-21	7.58E-21	yellow
TESK1	7016	testis-specific kinase 1 (TESK1), mRNA.	-0.01	8.30	2.36E-01	2.76E-01	yellow
TIMM8A	1678	translocase of inner mitochondrial membrane 8 homolog A (yeast) (TIMM8A), nuclear gene encoding mitochondrial protein, mRNA.	-0.07	6.28	3.55E-08	7.96E-08	yellow
TMEM109	79073	transmembrane protein 109 (TMEM109), mRNA.	-0.05	7.19	3.19E-07	6.73E-07	yellow
TMEM126B	55863	transmembrane protein 126B (TMEM126B), mRNA.	0.04	6.24	1.33E-02	1.87E-02	yellow
TMEM214	54867	hypothetical protein FLJ20254 (FLJ20254), mRNA.	-0.08	6.84	2.56E-09	6.18E-09	yellow
TMEM241	85019	chromosome 18 open reading frame 45 (C18orf45), mRNA.	-0.13	6.28	7.97E-19	3.22E-18	yellow
TMEM8A	58986	transmembrane protein 8 (five membrane-spanning domains) (TMEM8), mRNA.	0.05	10.12	6.95E-05	1.23E-04	yellow
TMEM99	147184	transmembrane protein 99 (TMEM99), mRNA.	0.03	8.96	2.61E-03	3.95E-03	yellow

TNFRSF8	943	tumor necrosis factor receptor superfamily, member 8 (TNFRSF8), transcript variant 2, mRNA.	-0.28	7.75	1.30E-53	2.33E-52	yellow
TNRC6A	27327	trinucleotide repeat containing 6A (TNRC6A), mRNA.	0.11	7.64	6.38E-10	1.59E-09	yellow
TPR	7175	translocated promoter region (to activated MET oncogene) (TPR), mRNA.	-0.09	6.95	2.21E-06	4.40E-06	yellow
TPX2	22974	TPX2, microtubule-associated, homolog (Xenopus laevis) (TPX2), mRNA.	0.01	10.43	2.65E-01	3.07E-01	yellow
TRAPPC12	51112	tetratricopeptide repeat domain 15 (TTC15), mRNA.	-0.24	8.17	3.54E-17	1.32E-16	yellow
TRIM28	10155	tripartite motif-containing 28 (TRIM28), mRNA.	-0.08	9.95	1.40E-05	2.64E-05	yellow
TRIM56	81844	tripartite motif-containing 56 (TRIM56), mRNA.	0.27	6.01	1.23E-39	1.22E-38	yellow
TROAP	10024	trophinin associated protein (tastin) (TROAP), mRNA.	-0.10	8.40	1.50E-14	4.83E-14	yellow
TRPV2	51393	transient receptor potential cation channel, subfamily V, member 2 (TRPV2), mRNA.	-0.01	8.90	5.94E-01	6.36E-01	yellow
TSR3	115939	chromosome 16 open reading frame 42 (C16orf42), mRNA.	-0.08	10.43	4.07E-10	1.03E-09	yellow
TTLL12	23170	tubulin tyrosine ligase-like family, member 12 (TTLL12), mRNA.	0.34	7.56	1.85E-44	2.30E-43	yellow

TXNDC15	79770	chromosome 5 open reading frame 14 (C5orf14), mRNA.	0.29	6.42	4.29E-45	5.50E-44	yellow
UBA1	7317	ubiquitin-activating enzyme E1 (A1S9T and BN75 temperature sensitivity complementing) (UBE1), transcript variant 2, mRNA.	-0.14	8.55	1.70E-22	8.12E-22	yellow
UBAP2L	9898	ubiquitin associated protein 2-like (UBAP2L), mRNA.	0.00	6.37	9.44E-01	9.52E-01	yellow
UBE2Z	65264	ubiquitin-conjugating enzyme E2Z (putative) (UBE2Z), mRNA.	0.15	7.92	1.22E-17	4.66E-17	yellow
UNC119	9094	unc-119 homolog (C. elegans) (UNC119), transcript variant 1, mRNA.	-0.45	8.38	5.70E-42	6.33E-41	yellow
UPF1	5976	UPF1 regulator of nonsense transcripts homolog (yeast) (UPF1), mRNA.	-0.22	8.43	4.42E-20	1.90E-19	yellow
UPF3A	65110	UPF3 regulator of nonsense transcripts homolog A (yeast) (UPF3A), transcript variant 1, mRNA.	-0.32	7.98	9.07E-33	6.62E-32	yellow
UQCC1	55245	chromosome 20 open reading frame 44 (C20orf44), transcript variant 3, mRNA.	0.06	11.20	1.58E-04	2.70E-04	yellow
UROD	7389	uroporphyrinogen decarboxylase (UROD), mRNA.	-0.06	6.84	5.65E-05	1.00E-04	yellow
USF2	7392	upstream transcription factor 2, c-fos interacting (USF2), transcript variant 1, mRNA.	0.05	8.43	2.14E-03	3.27E-03	yellow

USP5	8078	ubiquitin specific peptidase 5 (isopeptidase T) (USP5), mRNA.	0.33	7.33	2.32E-33	1.72E-32	yellow
VARS	7407	valyl-tRNA synthetase (VARS), mRNA.	0.11	7.89	7.22E-16	2.51E-15	yellow
VEZT	55591	vezatin, adherens junctions transmembrane protein (VEZT), mRNA.	0.02	10.03	4.67E-02	6.11E-02	yellow
VPS35	55737	vacuolar protein sorting 35 homolog (S. cerevisiae) (VPS35), mRNA.	-0.03	9.94	5.64E-03	8.25E-03	yellow
VPS51	738	chromosome 11 open reading frame2 (C11orf2), mRNA.	-0.02	8.17	3.13E-01	3.57E-01	yellow
WASF2	10163	WAS protein family, member 2 (WASF2), mRNA.	0.17	6.02	4.09E-22	1.92E-21	yellow
WDR6	11180	WD repeat domain 6 (WDR6), mRNA.	-0.22	8.69	1.27E-35	1.05E-34	yellow
WDR82	80335	transmembrane protein 113 (TMEM113), mRNA.	0.27	7.44	3.86E-25	2.11E-24	yellow
WDSUB1	151525	WD repeat, sterile alpha motif and U-box domain containing 1 (WDSUB1), mRNA.	0.06	7.48	9.82E-06	1.87E-05	yellow
XAB2	56949	XPA binding protein 2 (XAB2), mRNA.	0.03	6.06	2.70E-02	3.64E-02	yellow
YEATS2	55689	YEATS domain containing 2 (YEATS2), mRNA.	0.18	8.94	1.84E-27	1.10E-26	yellow
ZC3H14	79882	zinc finger CCCH-type containing 14 (ZC3H14), transcript variant 1, mRNA.	0.02	8.30	1.48E-01	1.80E-01	yellow

ZFP36	7538	zinc finger protein 36, C3H type, homolog (mouse) (ZFP36), mRNA.	-0.09	7.94	1.89E-08	4.31E-08	yellow
ZFYVE21	79038	zinc finger, FYVE domain containing 21 (ZFYVE21), mRNA.	0.08	8.53	3.07E-06	6.05E-06	yellow
ZNF277	11179	zinc finger protein 277 pseudogene (ZNF277P), mRNA.	0.17	6.53	8.06E-24	4.13E-23	yellow
ZNF444	55311	zinc finger protein 444 (ZNF444), mRNA.	-0.06	8.05	4.58E-07	9.54E-07	yellow
ZNF581	51545	zinc finger protein 581 (ZNF581), mRNA.	0.14	8.17	1.43E-25	7.95E-25	yellow
ZNF586	54807	zinc finger protein 586 (ZNF586), transcript variant 2, mRNA.	-0.36	7.82	1.11E-38	1.06E-37	yellow
ZNF816	125893	zinc finger protein 816A (ZNF816A), mRNA.	0.07	9.95	8.96E-07	1.83E-06	yellow

Table S3. Association between differentially expressed genes and three ICS response miRs using linear mixed model.

**Dependent_variable(Gene Entrenz ID)**	**Symbol**	**Definition**	**Independent_variable**	**beta**	**pvalue**	**p.adjust**	**Reactome Pathway**
51649	MRPS23	mitochondrial ribosomal protein S23 (MRPS23), nuclear gene encoding mitochondrial protein.	hsa.miR.339.3p	-0.033	9.80E-07	8.28E-03	R-HSA-392499~Metabolism of proteins
5688	PSMA7	proteasome (prosome, macropain) subunit, alpha type, 7 (PSMA7).	hsa.miR.339.3p	-0.032	7.67E-06	2.22E-02	R-HSA-1169091~Activation of NF-kappaB in B cells
1211	CLTA	clathrin, light chain (Lca) (CLTA), transcript variant 1.	hsa.miR.339.3p	-0.026	6.59E-06	2.22E-02	R-HSA-1280218~Adaptive Immune System

29093	MRPL22	mitochondrial ribosomal protein L22 (MRPL22), nuclear gene encoding mitochondrial protein, transcript variant 1.	hsa.miR.339.3p	-0.031	4.86E-06	2.22E-02	R-HSA-392499~Metabolism of proteins
2995	GYPC	glycophorin C (Gerbich blood group) (GYPC), transcript variant 1.	hsa.miR.339.3p	-0.038	1.70E-05	3.51E-02	R-HSA-109582~Hemostasis
84337	ELOF1	elongation factor 1 homolog (S. cerevisiae) (ELOF1).	hsa.miR.339.3p	-0.033	1.56E-05	3.51E-02	Na
126328	NDUFA11	NADH dehydrogenase (ubiquinone) 1 alpha subcomplex, 11, 14.7kDa (NDUFA11).	hsa.miR.339.3p	-0.03	2.09E-05	3.78E-02	R-HSA-1428517~The citric acid (TCA) cycle and respiratory electron transport
55658	RNF126	ring finger protein 126 (RNF126), transcript variant 1.	hsa.miR.339.3p	-0.029	3.55E-05	4.67E-02	R-HSA-1280218~Adaptive Immune System
1978	EIF4EBP1	eukaryotic translation initiation factor 4E binding protein 1 (EIF4EBP1).	hsa.miR.339.3p	-0.038	2.96E-05	4.67E-02	R-HSA-165159~MTOR signalling
10572	SIVA1	SIVA1, apoptosis-inducing factor (SIVA1), transcript variant 2.	hsa.miR.339.3p	-0.036	4.95E-05	5.50E-02	Na
51719	CAB39	calcium binding protein 39 (CAB39).	hsa.miR.339.3p	0.022	6.43E-05	5.81E-02	R-HSA-165159~MTOR signalling,R-HSA-168249~Innate Immune System
29093	MRPL22	mitochondrial ribosomal protein L22 (MRPL22), nuclear gene encoding mitochondrial protein, transcript variant 1.	hsa.miR.432.5p	-0.024	6.03E-05	5.81E-02	R-HSA-392499~Metabolism of proteins
6789	STK4	serine/threonine kinase 4 (STK4).	hsa.miR.339.3p	0.022	9.58E-05	6.45E-02	R-HSA-2028269~Signaling by Hippo
10856	RUVBL2	RuvB-like 2 (E. coli) (RUVBL2).	hsa.miR.339.3p	-0.022	9.82E-05	6.45E-02	R-HSA-3214847~HATs acetylate histones

27072	VPS41	vacuolar protein sorting 41 homolog (S. cerevisiae) (VPS41), transcript variant 2.	hsa.miR.339.3p	0.019	9.04E-05	6.45E-02	R-HSA-5663205~Infectious disease,R-HSA-9679506~SARS-CoV Infections
25953	PNKD	paroxysmal nonkinesiogenic dyskinesia (PNKD), transcript variant 1.	hsa.miR.339.3p	-0.037	8.03E-05	6.45E-02	Na
727877	LOC727877	PREDICTED: similar to Cyclin-L2 (Paneth cell-enhanced expression protein), transcript variant 2 (LOC727877).	hsa.miR.339.3p	-0.035	9.10E-05	6.45E-02	Na
9470	EIF4E2	eukaryotic translation initiation factor 4E family member 2 (EIF4E2).	hsa.miR.339.3p	-0.019	1.21E-04	7.30E-02	R-HSA-1280215~Cytokine Signaling in Immune system
29066	ZC3H7A	zinc finger CCCH-type containing 7A (ZC3H7A).	hsa.miR.339.3p	0.021	1.19E-04	7.30E-02	Na
1236	CCR7	chemokine (C-C motif) receptor 7 (CCR7).	hsa.miR.432.5p	0.068	1.27E-04	7.36E-02	R-HSA-380108~Chemokine receptors bind chemokines
54512	EXOSC4	exosome component 4 (EXOSC4).	hsa.miR.339.3p	-0.026	1.53E-04	8.19E-02	R-HSA-2262752~Cellular responses to stress
23385	NCSTN	nicastrin (NCSTN).	hsa.miR.339.3p	0.026	1.77E-04	8.97E-02	R-HSA-168249~Innate Immune System,R-HSA-157118~Signaling by NOTCH
6182	MRPL12	mitochondrial ribosomal protein L12 (MRPL12), nuclear gene encoding mitochondrial protein.	hsa.miR.339.3p	-0.026	1.93E-04	9.30E-02	R-HSA-392499~Metabolism of proteins
727877	LOC727877	PREDICTED: similar to Cyclin-L2 (Paneth cell-enhanced expression protein), transcript variant 2 (LOC727877).	hsa.miR.432.5p	-0.029	2.05E-04	9.54E-02	Na

Table S4. Details of genes from Black and Magenta modules.

IlluminaID	SYMBOL	Definition	EntrenzID	Module
ILMN_1794386	IL2RG	interleukin 2 receptor, gamma (severe combined immunodeficiency) (IL2RG), mRNA.	3561	magenta
ILMN_1651262	HNRNPAB	heterogeneous nuclear ribonucleoprotein A/B (HNRPAB), transcript variant 1, mRNA.	3182	black
ILMN_1651987	CCDC167	chromosome 6 open reading frame 129 (C6orf129), mRNA.	154467	black
ILMN_1654016	MYL12B	myosin regulatory light chain MRLC2 (MRLC2), mRNA.	103910	black
ILMN_1654609	EPB41L4A-AS1	TIGA1 (TIGA1), mRNA.	114915	black
ILMN_1655046	NUTF2	nuclear transport factor 2 (NUTF2), mRNA.	10204	black
ILMN_1655154	PTBP1	polypyrimidine tract binding protein 1 (PTBP1), transcript variant 2, mRNA.	5725	black
ILMN_1655340	RNF181	hypothetical protein LOC51255 (LOC51255), mRNA.	51255	black
ILMN_1656297	YBEY	chromosome 21 open reading frame 57 (C21orf57), transcript variant 1, mRNA.	54059	black
ILMN_1656899	CIB1	calcium and integrin binding 1 (calmyrin) (CIB1), mRNA.	10519	black
ILMN_1658065	EBP	emopamil binding protein (sterol isomerase) (EBP), mRNA.	10682	black
ILMN_1658351	FIS1	fission 1 (mitochondrial outer membrane) homolog (S. cerevisiae) (FIS1), mRNA.	51024	black
ILMN_1658486	MRPL54	mitochondrial ribosomal protein L54 (MRPL54), nuclear gene encoding mitochondrial protein, mRNA.	116541	black
ILMN_1658802	KRTCAP2	keratinocyte associated protein 2 (KRTCAP2), mRNA.	200185	black
ILMN_1659343	NA	bolA homolog 2B (E. coli) (BOLA2B), mRNA.	654483	black
ILMN_1659725	EXOSC5	exosome component 5 (EXOSC5), mRNA.	56915	black
ILMN_1660436	HSPA1B	heat shock 70kDa protein 1B (HSPA1B), mRNA.	3304	black
ILMN_1661347	IMP4	IMP4, U3 small nucleolar ribonucleoprotein, homolog (yeast) (IMP4), mRNA.	92856	black
ILMN_1661490	PFDN6	prefoldin subunit 6 (PFDN6), mRNA.	10471	black
ILMN_1661717	TFDP1	transcription factor Dp-1 (TFDP1), mRNA.	7027	black
ILMN_1662417	LRPPRC	leucine-rich PPR-motif containing (LRPPRC), mRNA.	10128	black
ILMN_1663921	TMEM141	transmembrane protein 141 (TMEM141), mRNA.	85014	black

ILMN_1664010	ELF1	E74-like factor 1 (ets domain transcription factor) (ELF1), mRNA.	1997	black
ILMN_1664429	ROMO1	chromosome 20 open reading frame 52 (C20orf52), nuclear gene encoding mitochondrial protein, mRNA.	140823	black
ILMN_1664614	FAU	Finkel-Biskis-Reilly murine sarcoma virus (FBR-MuSV) ubiquitously expressed (fox derived); ribosomal protein S30 (FAU), mRNA.	2197	black
ILMN_1666326	NDUFB9	NADH dehydrogenase (ubiquinone) 1 beta subcomplex, 9, 22kDa (NDUFB9), mRNA.	4715	black
ILMN_1666471	UQCRQ	ubiquinol-cytochrome c reductase, complex III subunit VII, 9.5kDa (UQCRQ), nuclear gene encoding mitochondrial protein, mRNA.	27089	black
ILMN_1666967	MPC1	brain protein 44-like (BRP44L), mRNA.	51660	black
ILMN_1667050	PRPS1	phosphoribosyl pyrophosphate synthetase 1 (PRPS1), mRNA.	5631	black
ILMN_1667257	SDHB	succinate dehydrogenase complex, subunit B, iron sulfur (Ip) (SDHB), mRNA.	6390	black
ILMN_1667716	TMEM101	transmembrane protein 101 (TMEM101), mRNA.	84336	black
ILMN_1669456	NME3	non-metastatic cells 3, protein expressed in (NME3), mRNA.	4832	black
ILMN_1669550	MAD2L2	MAD2 mitotic arrest deficient-like 2 (yeast) (MAD2L2), mRNA.	10459	black
ILMN_1670609	ATOX1	ATX1 antioxidant protein 1 homolog (yeast) (ATOX1), mRNA.	475	black
ILMN_1670809	NRM	nurim (nuclear envelope membrane protein) (NRM), mRNA.	11270	black
ILMN_1671237	GNGT2	guanine nucleotide binding protein (G protein), gamma transducing activity polypeptide 2 (GNGT2), mRNA.	2793	black
ILMN_1671374	C19orf53	chromosome 19 open reading frame 53 (C19orf53), mRNA.	28974	black
ILMN_1672149	CHCHD1	coiled-coil-helix-coiled-coil-helix domain containing 1 (CHCHD1), mRNA.	118487	black
ILMN_1672389	CRYZ	crystallin, zeta (quinone reductase) (CRYZ), mRNA.	1429	black
ILMN_1673323	DPM3	dolichyl-phosphate mannosyltransferase polypeptide 3 (DPM3), transcript variant 1, mRNA.	54344	black
ILMN_1673991	ATIC	5-aminoimidazole-4-carboxamide ribonucleotide formyltransferase/IMP cyclohydrolase (ATIC), mRNA.	471	black

ILMN_1674243	TFRC	transferrin receptor (p90, CD71) (TFRC), mRNA.	7037	black
ILMN_1674609	CLTB	clathrin, light chain (Lcb) (CLTB), transcript variant 1, mRNA.	1212	black
ILMN_1675239	NDUFA7	NADH dehydrogenase (ubiquinone) 1 alpha subcomplex, 7, 14.5kDa (NDUFA7), mRNA.	4701	black
ILMN_1676393	ATP5G1	ATP synthase, H+ transporting, mitochondrial F0 complex, subunit C1 (subunit 9) (ATP5G1), nuclear gene encoding mitochondrial protein, transcript variant 2, mRNA.	516	black
ILMN_1676611	PHPT1	phosphohistidine phosphatase 1 (PHPT1), mRNA.	29085	black
ILMN_1678165	LSM7	LSM7 homolog, U6 small nuclear RNA associated (S. cerevisiae) (LSM7), mRNA.	51690	black
ILMN_1678308	ATP6V1F	ATPase, H+ transporting, lysosomal 14kDa, V1 subunit F (ATP6V1F), mRNA.	9296	black
ILMN_1679809	GSTP1	glutathione S-transferase pi (GSTP1), mRNA.	2950	black
ILMN_1679841	SIVA1	SIVA1, apoptosis-inducing factor (SIVA1), transcript variant 1, mRNA.	10572	black
ILMN_1680403	SSR4	signal sequence receptor, delta (translocon-associated protein delta) (SSR4), mRNA.	6748	black
ILMN_1681437	DCXR	dicarbonyl/L-xylulose reductase (DCXR), mRNA.	51181	black
ILMN_1681617	NA	actin related protein 2/3 complex, subunit 3, 21kDa (ARPC3), mRNA.	10094	black
ILMN_1682299	NDUFA11	NADH dehydrogenase (ubiquinone) 1 alpha subcomplex, 11, 14.7kDa (NDUFA11), mRNA.	126328	black
ILMN_1682316	TRIM33	tripartite motif-containing 33 (TRIM33), transcript variant a, mRNA.	51592	black
ILMN_1682332	GYPC	glycophorin C (Gerbich blood group) (GYPC), transcript variant 1, mRNA.	2995	black
ILMN_1683883	NA	aminoacylase 1 (ACY1), mRNA.	95	black
ILMN_1684446	SPAG7	sperm associated antigen 7 (SPAG7), mRNA.	9552	black
ILMN_1685088	RPL36	ribosomal protein L36 (RPL36), transcript variant 1, mRNA.	25873	black
ILMN_1687359	MRPS23	mitochondrial ribosomal protein S23 (MRPS23), nuclear gene encoding mitochondrial protein, mRNA.	51649	black
ILMN_1688318	FRG1B	similar to FRG1 protein (FSHD region gene 1 protein) (MGC72104), mRNA.	284802	black

ILMN_1688702	PJA2	praja 2, RING-H2 motif containing (PJA2), mRNA.	9867	black
ILMN_1689389	SF3B5	splicing factor 3b, subunit 5, 10kDa (SF3B5), mRNA.	83443	black
ILMN_1690217	BFSP2	beaded filament structural protein 2, phakinin (BFSP2), mRNA.	8419	black
ILMN_1690802	TRMT112	hypothetical protein HSPC152 (HSPC152), mRNA.	51504	black
ILMN_1691379	UBL5	ubiquitin-like 5 (UBL5), transcript variant 1, mRNA.	59286	black
ILMN_1692398	CNTNAP1	contactin associated protein 1 (CNTNAP1), mRNA.	8506	black
ILMN_1692486	ZNRD1	zinc ribbon domain containing 1 (ZNRD1), transcript variant a, mRNA.	30834	black
ILMN_1693227	ZC3H7A	zinc finger CCCH-type containing 7A (ZC3H7A), mRNA.	29066	black
ILMN_1693685	LINC00116	PREDICTED: LOC205251 (LOC205251), misc RNA.	205251	black
ILMN_1696544	EDF1	endothelial differentiation-related factor 1 (EDF1), transcript variant alpha, mRNA.	8721	black
ILMN_1696568	ATP2C1	ATPase, Ca++ transporting, type 2C, member 1 (ATP2C1), transcript variant 1, mRNA.	27032	black
ILMN_1697820	HINT2	histidine triad nucleotide binding protein 2 (HINT2), mRNA.	84681	black
ILMN_1698766	PYCARD	PYD and CARD domain containing (PYCARD), transcript variant 1, mRNA.	29108	black
ILMN_1700306	OCIAD2	OCIA domain containing 2 (OCIAD2), transcript variant 1, mRNA.	132299	black
ILMN_1700419	TMEM208	HSPC171 protein (HSPC171), mRNA.	29100	black
ILMN_1700955	TCTEX1D2	hypothetical protein MGC33212 (MGC33212), mRNA.	255758	black
ILMN_1701134	PTEN	phosphatase and tensin homolog (mutated in multiple advanced cancers 1) (PTEN), mRNA.	5728	black
ILMN_1702059	RPS19BP1	ribosomal protein S19 binding protein 1 (RPS19BP1), mRNA.	91582	black
ILMN_1702828	CHMP4A	chromatin modifying protein 4A (CHMP4A), mRNA.	29082	black
ILMN_1703697	LANCL1	LanC lantibiotic synthetase component C-like 1 (bacterial) (LANCL1), mRNA.	10314	black
ILMN_1704024	TMEM160	transmembrane protein 160 (TMEM160), mRNA.	54958	black
ILMN_1704404	PSMD13	proteasome (prosome, macropain) 26S subunit, non-ATPase, 13 (PSMD13), transcript variant 1, mRNA.	5719	black
ILMN_1704785	FUOM	chromosome 10 open reading frame 125 (C10orf125), mRNA.	282969	black

ILMN_1704891	SMARCD2	SWI/SNF related, matrix associated, actin dependent regulator of chromatin, subfamily d, member 2 (SMARCD2), mRNA.	6603	black
ILMN_1705464	MRPL41	mitochondrial ribosomal protein L41 (MRPL41), nuclear gene encoding mitochondrial protein, mRNA.	64975	black
ILMN_1707783	TMA7	coiled-coil domain containing 72 (CCDC72), mRNA.	51372	black
ILMN_1707810	RPS5	ribosomal protein S5 (RPS5), mRNA.	6193	black
ILMN_1708151	LAGE3	L antigen family, member 3 (LAGE3), mRNA.	8270	black
ILMN_1709451	TFPT	TCF3 (E2A) fusion partner (in childhood Leukemia) (TFPT), mRNA.	29844	black
ILMN_1709626	IFI27L1	family with sequence similarity 14, member B (FAM14B), mRNA.	122509	black
ILMN_1710758	RNF20	ring finger protein 20 (RNF20), mRNA.	56254	black
ILMN_1710873	ZNF330	zinc finger protein 330 (ZNF330), mRNA.	27309	black
ILMN_1710979	ANKRD39	ankyrin repeat domain 39 (ANKRD39), mRNA.	51239	black
ILMN_1711617	GMFG	glia maturation factor, gamma (GMFG), mRNA.	9535	black
ILMN_1711810	PNKD	paroxysmal nonkinesiogenic dyskinesia (PNKD), transcript variant 1, mRNA.	25953	black
ILMN_1714495	NDUFB2	NADH dehydrogenase (ubiquinone) 1 beta subcomplex, 2, 8kDa (NDUFB2), nuclear gene encoding mitochondrial protein, mRNA.	4708	black
ILMN_1715324	HSD17B8	hydroxysteroid (17-beta) dehydrogenase 8 (HSD17B8), mRNA.	7923	black
ILMN_1716093	KRT10	keratin 10 (epidermolytic hyperkeratosis; keratosis palmaris et plantaris) (KRT10), mRNA.	3858	black
ILMN_1716169	MIF	macrophage migration inhibitory factor (glycosylation-inhibiting factor) (MIF), mRNA.	4282	black
ILMN_1716733	MYOM2	myomesin (M-protein) 2, 165kDa (MYOM2), mRNA.	9172	black
ILMN_1716913	TRAPPC1	trafficking protein particle complex 1 (TRAPPC1), mRNA.	58485	black
ILMN_1717714	CDKN2A	cyclin-dependent kinase inhibitor 2A (melanoma, p16, inhibits CDK4) (CDKN2A), transcript variant 1, mRNA.	1029	black
ILMN_1718672	NHP2	nucleolar protein family A, member 2 (H/ACA small nucleolar RNPs) (NOLA2), transcript variant 1, mRNA.	55651	black

ILMN_1720422	G3BP2	GTPase activating protein (SH3 domain) binding protein 2 (G3BP2), transcript variant 1, mRNA.	9908	black
ILMN_1720442	NCBP2	nuclear cap binding protein subunit 2, 20kDa (NCBP2), transcript variant 2, mRNA.	22916	black
ILMN_1720542	POLR2I	polymerase (RNA) II (DNA directed) polypeptide I, 14.5kDa (POLR2I), mRNA.	5438	black
ILMN_1721977	NAA10	ARD1 homolog A, N-acetyltransferase (S. cerevisiae) (ARD1A), mRNA.	8260	black
ILMN_1722102	ANAPC11	APC11 anaphase promoting complex subunit 11 homolog (yeast) (ANAPC11), transcript variant 1, mRNA.	51529	black
ILMN_1722491	APRT	adenine phosphoribosyltransferase (APRT), transcript variant 1, mRNA.	353	black
ILMN_1722905	MRPS11	mitochondrial ribosomal protein S11 (MRPS11), nuclear gene encoding mitochondrial protein, transcript variant 1, mRNA.	64963	black
ILMN_1723580	RBMX	RNA binding motif protein, X-linked (RBMX), mRNA.	27316	black
ILMN_1724700	RIOK3	RIO kinase 3 (yeast) (RIOK3), transcript variant 2, mRNA.	8780	black
ILMN_1725241	GSTK1	glutathione S-transferase kappa 1 (GSTK1), mRNA.	373156	black
ILMN_1725366	SLC27A5	solute carrier family 27 (fatty acid transporter), member 5 (SLC27A5), mRNA.	10998	black
ILMN_1725705	CLPP	ClpP caseinolytic peptidase, ATP-dependent, proteolytic subunit homolog (E. coli) (CLPP), nuclear gene encoding mitochondrial protein, mRNA.	8192	black
ILMN_1726603	ATP5I	ATP synthase, H+ transporting, mitochondrial F0 complex, subunit E (ATP5I), nuclear gene encoding mitochondrial protein, mRNA.	521	black
ILMN_1726884	NARF	nuclear prelamin A recognition factor (NARF), transcript variant 1, mRNA.	26502	black
ILMN_1727004	MRPL14	mitochondrial ribosomal protein L14 (MRPL14), nuclear gene encoding mitochondrial protein, mRNA.	64928	black
ILMN_1727740	SYNCRIP	synaptotagmin binding, cytoplasmic RNA interacting protein (SYNCRIP), mRNA.	10492	black
ILMN_1730391	MRPS18A	mitochondrial ribosomal protein S18A (MRPS18A), nuclear gene encoding mitochondrial protein, mRNA.	55168	black

ILMN_1730433	CD2AP	CD2-associated protein (CD2AP), mRNA.	23607	black
ILMN_1732555	B4GALT6	UDP-Gal:betaGlcNAc beta 1,4-galactosyltransferase, polypeptide 6 (B4GALT6), mRNA.	9331	black
ILMN_1732750	COA4	coiled-coil-helix-coiled-coil-helix domain containing 8 (CHCHD8), mRNA.	51287	black
ILMN_1732985	PHF20L1	PHD finger protein 20-like 1 (PHF20L1), transcript variant 1, mRNA.	51105	black
ILMN_1733927	TCEB2	transcription elongation factor B (SIII), polypeptide 2 (18kDa, elongin B) (TCEB2), transcript variant 2, mRNA.	6923	black
ILMN_1733956	IARS	isoleucyl-tRNA synthetase (IARS), transcript variant short, mRNA.	3376	black
ILMN_1733960	NAA38	LSM domain containing 1 (LSMD1), mRNA.	84316	black
ILMN_1735909	TRPT1	tRNA phosphotransferase 1 (TRPT1), transcript variant 1, mRNA.	83707	black
ILMN_1736389	SEC61B	Sec61 beta subunit (SEC61B), mRNA.	10952	black
ILMN_1736752	COMTD1	catechol-O-methyltransferase domain containing 1 (COMTD1), mRNA.	118881	black
ILMN_1737236	COX5B	cytochrome c oxidase subunit Vb (COX5B), mRNA.	1329	black
ILMN_1737358	TMEM256	chromosome 17 open reading frame 61 (C17orf61), mRNA.	254863	black
ILMN_1738326	EIF4E2	eukaryotic translation initiation factor 4E family member 2 (EIF4E2), mRNA.	9470	black
ILMN_1738529	BCS1L	BCS1-like (yeast) (BCS1L), nuclear gene encoding mitochondrial protein, transcript variant 2, mRNA.	617	black
ILMN_1738938	TIMM8B	translocase of inner mitochondrial membrane 8 homolog B (yeast) (TIMM8B), mRNA.	26521	black
ILMN_1739345	LBHD1	chromosome 11 open reading frame 48 (C11orf48), mRNA.	79081	black
ILMN_1740319	IFI27L2	family with sequence similarity 14, member A (FAM14A), mRNA.	83982	black
ILMN_1741491	ZNHIT1	zinc finger, HIT type 1 (ZNHIT1), mRNA.	10467	black
ILMN_1743049	PWP1	PWP1 homolog (S. cerevisiae) (PWP1), mRNA.	11137	black
ILMN_1743711	XPOT	PREDICTED: similar to Exportin-T (tRNA exportin) (Exportin(tRNA)) (LOC441228), mRNA.	441228	black
ILMN_1744604	CYBA	cytochrome b-245, alpha polypeptide (CYBA), mRNA.	1535	black

ILMN_1744628	FDX1L	similar to RIKEN cDNA B230118G17 gene (MGC19604), transcript variant 1, mRNA.	112812	black
ILMN_1744647	CAND1	cullin-associated and neddylation-dissociated 1 (CAND1), mRNA.	55832	black
ILMN_1745053	MSRB2	methionine sulfoxide reductase B2 (MSRB2), mRNA.	22921	black
ILMN_1745885	POLR2F	polymerase (RNA) II (DNA directed) polypeptide F (POLR2F), mRNA.	5435	black
ILMN_1746175	TNFSF4	tumor necrosis factor (ligand) superfamily, member 4 (tax-transcriptionally activated glycoprotein 1, 34kDa) (TNFSF4), mRNA.	7292	black
ILMN_1746241	SDHC	succinate dehydrogenase complex, subunit C, integral membrane protein, 15kDa (SDHC), nuclear gene encoding mitochondrial protein, mRNA.	6391	black
ILMN_1746408	MIDN	midnolin (MIDN), mRNA.	90007	black
ILMN_1746598	NA	sodium channel modifier 1 (SCNM1), transcript variant 2, mRNA.	79005	black
ILMN_1747195	PSMB8	proteasome (prosome, macropain) subunit, beta type, 8 (large multifunctional peptidase 7) (PSMB8), transcript variant 2, mRNA.	5696	black
ILMN_1747935	GOLGB1	golgi autoantigen, golgin subfamily b, macrogolgin (with transmembrane signal), 1 (GOLGB1), mRNA.	2804	black
ILMN_1749014	ACLY	ATP citrate lyase (ACLY), transcript variant 1, mRNA.	47	black
ILMN_1749662	GPX1	glutathione peroxidase 1 (GPX1), transcript variant 1, mRNA.	2876	black
ILMN_1749709	NDUFB11	NADH dehydrogenase (ubiquinone) 1 beta subcomplex, 11, 17.3kDa (NDUFB11), mRNA.	54539	black
ILMN_1750143	ATP5J2	ATP synthase, H+ transporting, mitochondrial F0 complex, subunit F2 (ATP5J2), nuclear gene encoding mitochondrial protein, transcript variant 3, mRNA.	9551	black
ILMN_1750658	HAX1	HCLS1 associated protein X-1 (HAX1), transcript variant 2, mRNA.	10456	black
ILMN_1751368	HNRNPD	heterogeneous nuclear ribonucleoprotein D (AU-rich element RNA binding protein 1, 37kDa) (HNRPD), transcript variant 4, mRNA.	3184	black
ILMN_1751743	XRCC1	X-ray repair complementing defective repair in Chinese hamster cells 1 (XRCC1), mRNA.	7515	black

ILMN_1752340	ARF5	ADP-ribosylation factor 5 (ARF5), mRNA.	381	black
ILMN_1752631	CGGBP1	CGG triplet repeat binding protein 1 (CGGBP1), transcript variant 1, mRNA.	8545	black
ILMN_1753393	OSGEP	O-sialoglycoprotein endopeptidase (OSGEP), mRNA.	55644	black
ILMN_1753862	SRP54	signal recognition particle 54kDa (SRP54), mRNA.	6729	black
ILMN_1754544	PSMA7	proteasome (prosome, macropain) subunit, alpha type, 7 (PSMA7), mRNA.	5688	black
ILMN_1756352	LAMTOR2	mitogen-activated protein-binding protein-interacting protein (MAPBPIP), mRNA.	28956	black
ILMN_1757914	WDR83OS	chromosome 19 open reading frame 56 (C19orf56), mRNA.	51398	black
ILMN_1758398	GUK1	guanylate kinase 1 (GUK1), mRNA.	2987	black
ILMN_1759184	C19orf48	chromosome 19 open reading frame 48 (C19orf48), mRNA.	84798	black
ILMN_1760027	WAS	Wiskott-Aldrich syndrome (eczema-thrombocytopenia) (WAS), mRNA.	7454	black
ILMN_1760849	NETO2	neuropilin (NRP) and tolloid (TLL)-like 2 (NETO2), mRNA.	81831	black
ILMN_1761147	GABPB1	GA binding protein transcription factor, beta subunit 2 (GABPB2), transcript variant gamma-2, mRNA.	2553	black
ILMN_1761242	COMMD1	copper metabolism (Murr1) domain containing 1 (COMMD1), mRNA.	150684	black
ILMN_1761728	ACP5	acid phosphatase 5, tartrate resistant (ACP5), mRNA.	54	black
ILMN_1762281	DCTN3	dynactin 3 (p22) (DCTN3), transcript variant 1, mRNA.	11258	black
ILMN_1762654	PAFAH1B3	platelet-activating factor acetylhydrolase, isoform Ib, gamma subunit 29kDa (PAFAH1B3), mRNA.	5050	black
ILMN_1763147	NDUFB6	NADH dehydrogenase (ubiquinone) 1 beta subcomplex, 6, 17kDa (NDUFB6), nuclear gene encoding mitochondrial protein, transcript variant 2, mRNA.	4712	black
ILMN_1763379	NA	kelch-like 23 (Drosophila) (KLHL23), mRNA.	151230	black
ILMN_1763884	NA	mitochondria-associated protein involved in granulocyte-macrophage colony-stimulating factor signal transduction (Magmas), nuclear gene encoding mitochondrial protein, mRNA.	51025	black
ILMN_1764230	GNPTG	N-acetylglucosamine-1-phosphate transferase, gamma subunit (GNPTG), mRNA.	84572	black

ILMN_1765684	C19orf70	hypothetical protein P117 (P117), mRNA.	125988	black
ILMN_1765858	CAB39	calcium binding protein 39 (CAB39), mRNA.	51719	black
ILMN_1767123	NDUFA2	NADH dehydrogenase (ubiquinone) 1 alpha subcomplex, 2, 8kDa (NDUFA2), mRNA.	4695	black
ILMN_1767139	NDUFA13	NADH dehydrogenase (ubiquinone) 1 alpha subcomplex, 13 (NDUFA13), mRNA.	51079	black
ILMN_1767549	BLOC1S1	biogenesis of lysosome-related organelles complex-1, subunit 1 (BLOC1S1), mRNA.	2647	black
ILMN_1768197	PTBP3	ROD1 regulator of differentiation 1 (S. pombe) (ROD1), mRNA.	9991	black
ILMN_1768712	NUDT8	nudix (nucleoside diphosphate linked moiety X)-type motif 8 (NUDT8), mRNA.	254552	black
ILMN_1769343	DNPH1	chromosome 6 open reading frame 108 (C6orf108), transcript variant 1, mRNA.	10591	black
ILMN_1770885	COX17	COX17 cytochrome c oxidase assembly homolog (S. cerevisiae) (COX17), nuclear gene encoding mitochondrial protein, mRNA.	10063	black
ILMN_1771003	HAUS4	chromosome 14 open reading frame 94 (C14orf94), mRNA.	54930	black
ILMN_1771019	MTMR4	myotubularin related protein 4 (MTMR4), mRNA.	9110	black
ILMN_1771734	GMPS	guanine monphosphate synthetase (GMPS), mRNA.	8833	black
ILMN_1772796	DYNLL2	dynein, light chain, LC8-type 2 (DYNLL2), mRNA.	140735	black
ILMN_1772981	EPN1	epsin 1 (EPN1), mRNA.	29924	black
ILMN_1773751	HRAS	v-Ha-ras Harvey rat sarcoma viral oncogene homolog (HRAS), transcript variant 1, mRNA.	3265	black
ILMN_1773760	PAICS	phosphoribosylaminoimidazole carboxylase, phosphoribosylaminoimidazole succinocarboxamide synthetase (PAICS), transcript variant 3, mRNA.	10606	black
ILMN_1773780	FAM173A	chromosome 16 open reading frame 24 (C16orf24), mRNA.	65990	black
ILMN_1774334	HIGD2A	HIG1 domain family, member 2A (HIGD2A), mRNA.	192286	black
ILMN_1774890	LAS1L	LAS1-like (S. cerevisiae) (LAS1L), mRNA.	81887	black

ILMN_1774990	C16orf13	hypothetical protein MGC13114 (MGC13114), transcript variant 7, mRNA.	84326	black
ILMN_1775243	RPL35	PREDICTED: similar to 60S ribosomal protein L35, transcript variant 5 (LOC441246), mRNA.	441246	black
ILMN_1775672	SOD2	superoxide dismutase 2, mitochondrial (SOD2), nuclear gene encoding mitochondrial protein, transcript variant 2, mRNA.	6648	black
ILMN_1777721	MAPRE1	microtubule-associated protein, RP/EB family, member 1 (MAPRE1), mRNA.	22919	black
ILMN_1777991	DYRK4	dual-specificity tyrosine-(Y)-phosphorylation regulated kinase 4 (DYRK4), mRNA.	8798	black
ILMN_1778347	NUDT2	nudix (nucleoside diphosphate linked moiety X)-type motif 2 (NUDT2), transcript variant 2, mRNA.	318	black
ILMN_1779735	LAMTOR4	similar to CG14977-PA (LOC389541), mRNA.	389541	black
ILMN_1779751	C7orf55	hypothetical protein HSPC268 (HSPC268), mRNA.	154791	black
ILMN_1779813	FAM96B	family with sequence similarity 96, member B (FAM96B), mRNA.	51647	black
ILMN_1780127	MRPS34	mitochondrial ribosomal protein S34 (MRPS34), nuclear gene encoding mitochondrial protein, mRNA.	65993	black
ILMN_1780533	RNASE6	ribonuclease, RNase A family, k6 (RNASE6), mRNA.	6039	black
ILMN_1780977	PVRIG	poliovirus receptor related immunoglobulin domain containing (PVRIG), mRNA.	79037	black
ILMN_1781290	RHOA	ras homolog gene family, member A (RHOA), mRNA.	387	black
ILMN_1781638	HDDC3	HD domain containing 3 (HDDC3), mRNA.	374659	black
ILMN_1781986	UQCR10	ubiquinol-cytochrome c reductase complex (7.2 kD) (UCRC), transcript variant 2, mRNA.	29796	black
ILMN_1783636	COX6A1	cytochrome c oxidase subunit VIa polypeptide 1 (COX6A1), nuclear gene encoding mitochondrial protein, mRNA.	1337	black
ILMN_1783681	MRPL34	mitochondrial ribosomal protein L34 (MRPL34), nuclear gene encoding mitochondrial protein, mRNA.	64981	black
ILMN_1783843	MIIP	invasion inhibitory protein 45 (IIP45), transcript variant 1, mRNA.	60672	black

ILMN_1784641	NDUFA3	NADH dehydrogenase (ubiquinone) 1 alpha subcomplex, 3, 9kDa (NDUFA3), mRNA.	4696	black
ILMN_1785175	SWAP70	SWAP-70 protein (SWAP70), mRNA.	23075	black
ILMN_1785570	SUSD3	sushi domain containing 3 (SUSD3), mRNA.	203328	black
ILMN_1787705	ATP6V1B2	ATPase, H+ transporting, lysosomal 56/58kDa, V1 subunit B2 (ATP6V1B2), mRNA.	526	black
ILMN_1788547	GCLM	glutamate-cysteine ligase, modifier subunit (GCLM), mRNA.	2730	black
ILMN_1789074	HSPA1A	heat shock 70kDa protein 1A (HSPA1A), mRNA.	3303	black
ILMN_1789136	NA	small EDRK-rich factor 2 (SERF2), mRNA.	10169	black
ILMN_1789614	TPT1	tumor protein, translationally-controlled 1 (TPT1), mRNA.	7178	black
ILMN_1790461	UQCC2	chromosome 6 open reading frame 125 (C6orf125), mRNA.	84300	black
ILMN_1794230	SCAND1	SCAN domain containing 1 (SCAND1), transcript variant 1, mRNA.	51282	black
ILMN_1794522	EIF5A	eukaryotic translation initiation factor 5A (EIF5A), mRNA.	1984	black
ILMN_1795341	SRSF1	splicing factor, arginine/serine-rich 1 (splicing factor 2, alternate splicing factor) (SFRS1), transcript variant 2, mRNA.	6426	black
ILMN_1795639	MGMT	O-6-methylguanine-DNA methyltransferase (MGMT), mRNA.	4255	black
ILMN_1796595	C5orf22	chromosome 5 open reading frame 22 (C5orf22), mRNA.	55322	black
ILMN_1797530	CHCHD5	coiled-coil-helix-coiled-coil-helix domain containing 5 (CHCHD5), mRNA.	84269	black
ILMN_1797828	DDRGK1	chromosome 20 open reading frame 116 (C20orf116), mRNA.	65992	black
ILMN_1798827	SRBD1	S1 RNA binding domain 1 (SRBD1), mRNA.	55133	black
ILMN_1798886	NUDT21	nudix (nucleoside diphosphate linked moiety X)-type motif 21 (NUDT21), mRNA.	11051	black
ILMN_1799015	PXMP2	peroxisomal membrane protein 2, 22kDa (PXMP2), mRNA.	5827	black
ILMN_1800197	MRPL36	mitochondrial ribosomal protein L36 (MRPL36), nuclear gene encoding mitochondrial protein, mRNA.	64979	black
ILMN_1800602	GCA	grancalcin, EF-hand calcium binding protein (GCA), mRNA.	25801	black

ILMN_1801119	BCL2	B-cell CLL/lymphoma 2 (BCL2), nuclear gene encoding mitochondrial protein, transcript variant alpha, mRNA.	596	black
ILMN_1802553	NA	mitochondrial ribosomal protein S24 (MRPS24), nuclear gene encoding mitochondrial protein, mRNA.	64951	black
ILMN_1802627	PSMG3	chromosome 7 open reading frame 48 (C7orf48), mRNA.	84262	black
ILMN_1804248	FDPS	farnesyl diphosphate synthase (farnesyl pyrophosphate synthetase, dimethylallyltranstransferase, geranyltranstransferase) (FDPS), mRNA.	2224	black
ILMN_1804656	COX14	chromosome 12 open reading frame 62 (C12orf62), mRNA.	84987	black
ILMN_1804679	KAT8	MYST histone acetyltransferase 1 (MYST1), mRNA.	84148	black
ILMN_1805922	EBPL	emopamil binding protein-like (EBPL), mRNA.	84650	black
ILMN_1806123	MRPL23	mitochondrial ribosomal protein L23 (MRPL23), nuclear gene encoding mitochondrial protein, mRNA.	6150	black
ILMN_1807240	RPS9	ribosomal protein S9 (RPS9), mRNA.	6203	black
ILMN_1808196	GSTO1	glutathione S-transferase omega 1 (GSTO1), mRNA.	9446	black
ILMN_1809013	MYL6	myosin, light chain 6, alkali, smooth muscle and non-muscle (MYL6), transcript variant 2, mRNA.	4637	black
ILMN_1809495	COX8A	cytochrome c oxidase subunit 8A (ubiquitous) (COX8A), mRNA.	1351	black
ILMN_1810625	UFC1	ubiquitin-fold modifier conjugating enzyme 1 (UFC1), mRNA.	51506	black
ILMN_1810680	NA	bolA homolog 2 (E. coli) (BOLA2), transcript variant 2, mRNA.	552900	black
ILMN_1810901	RNASEH2A	ribonuclease H2, subunit A (RNASEH2A), mRNA.	10535	black
ILMN_1811327	MRPL27	mitochondrial ribosomal protein L27 (MRPL27), nuclear gene encoding mitochondrial protein, transcript variant 1, mRNA.	51264	black
ILMN_1812638	ATP5L	ATP synthase, H+ transporting, mitochondrial F0 complex, subunit G (ATP5L), nuclear gene encoding mitochondrial protein, mRNA.	10632	black
ILMN_1813260	TIMM17B	translocase of inner mitochondrial membrane 17 homolog B (yeast) (TIMM17B), mRNA.	10245	black

ILMN_1813604	NDUFB7	NADH dehydrogenase (ubiquinone) 1 beta subcomplex, 7, 18kDa (NDUFB7), nuclear gene encoding mitochondrial protein, mRNA.	4713	black
ILMN_1813682	MRPL53	mitochondrial ribosomal protein L53 (MRPL53), nuclear gene encoding mitochondrial protein, mRNA.	116540	black
ILMN_1813817	MRPL55	mitochondrial ribosomal protein L55 (MRPL55), nuclear gene encoding mitochondrial protein, transcript variant 8, mRNA.	128308	black
ILMN_1815024	PRDX5	peroxiredoxin 5 (PRDX5), nuclear gene encoding mitochondrial protein, transcript variant 1, mRNA.	25824	black
ILMN_1815115	CYC1	cytochrome c-1 (CYC1), mRNA.	1537	black
ILMN_1815134	PI4K2B	phosphatidylinositol 4-kinase type 2 beta (PI4K2B), mRNA.	55300	black
ILMN_1815479	NOP10	nucleolar protein family A, member 3 (H/ACA small nucleolar RNPs) (NOLA3), mRNA.	55505	black
ILMN_1815689	CD46	CD46 molecule, complement regulatory protein (CD46), transcript variant d, mRNA.	4179	black
ILMN_1343294	ACTB	actin, beta (ACTB), mRNA.	60	magenta
ILMN_1652478	NA	NODAL modulator 2 (NOMO2), transcript variant 2, mRNA.	283820	magenta
ILMN_1652918	RHOF	ras homolog gene family, member F (in filopodia) (RHOF), mRNA.	54509	magenta
ILMN_1653599	ATP5D	ATP synthase, H+ transporting, mitochondrial F1 complex, delta subunit (ATP5D), nuclear gene encoding mitochondrial protein, transcript variant 2, mRNA.	513	magenta
ILMN_1656111	MYLIP	myosin regulatory light chain interacting protein (MYLIP), mRNA.	29116	magenta
ILMN_1656540	RUVBL1	RuvB-like 1 (E. coli) (RUVBL1), mRNA.	8607	magenta
ILMN_1657550	MVD	mevalonate (diphospho) decarboxylase (MVD), mRNA.	4597	magenta
ILMN_1657898	MTFP1	mitochondrial protein 18 kDa (MTP18), nuclear gene encoding mitochondrial protein, transcript variant 1, mRNA.	51537	magenta
ILMN_1657983	TERF2IP	telomeric repeat binding factor 2, interacting protein (TERF2IP), mRNA.	54386	magenta
ILMN_1658003	YIF1B	Yip1 interacting factor homolog B (S. cerevisiae) (YIF1B), transcript variant 3, mRNA.	90522	magenta
ILMN_1658456	SNX19	sorting nexin 19 (SNX19), mRNA.	399979	magenta

ILMN_1658472	APH1A	anterior pharynx defective 1 homolog A (C. elegans) (APH1A), transcript variant 2, mRNA.	51107	magenta
ILMN_1658678	SAAL1	serum amyloid A-like 1 (SAAL1), mRNA.	113174	magenta
ILMN_1658928	GNB2	guanine nucleotide binding protein (G protein), beta polypeptide 2 (GNB2), mRNA.	2783	magenta
ILMN_1659270	OTP	orthopedia homeobox (OTP), mRNA.	23440	magenta
ILMN_1659857	SNAP29	synaptosomal-associated protein, 29kDa (SNAP29), mRNA.	9342	magenta
ILMN_1660880	RNH1	ribonuclease/angiogenin inhibitor 1 (RNH1), transcript variant 8, mRNA.	6050	magenta
ILMN_1661337	SRM	spermidine synthase (SRM), mRNA.	6723	magenta
ILMN_1661439	FLOT1	flotillin 1 (FLOT1), mRNA.	10211	magenta
ILMN_1661594	C2orf42	chromosome 2 open reading frame 42 (C2orf42), mRNA.	54980	magenta
ILMN_1664098	FASTK	Fas-activated serine/threonine kinase (FASTK), transcript variant 1, mRNA.	10922	magenta
ILMN_1666194	IFRD2	interferon-related developmental regulator 2 (IFRD2), mRNA.	7866	magenta
ILMN_1667213	DFFA	DNA fragmentation factor, 45kDa, alpha polypeptide (DFFA), transcript variant 1, mRNA.	1676	magenta
ILMN_1669070	MIPEP	mitochondrial intermediate peptidase (MIPEP), nuclear gene encoding mitochondrial protein, mRNA.	4285	magenta
ILMN_1669572	RNF126	ring finger protein 126 (RNF126), transcript variant 2, mRNA.	55658	magenta
ILMN_1669718	PSENEN	presenilin enhancer 2 homolog (C. elegans) (PSENEN), mRNA.	55851	magenta
ILMN_1671054	HLA-A	major histocompatibility complex, class I, A (HLA-A), mRNA.	3105	magenta
ILMN_1671191	UQCRC1	ubiquinol-cytochrome c reductase core protein I (UQCRC1), mRNA.	7384	magenta
ILMN_1671583	MKRN1	makorin, ring finger protein, 1 (MKRN1), mRNA.	23608	magenta
ILMN_1671932	SAMM50	sorting and assembly machinery component 50 homolog (S. cerevisiae) (SAMM50), mRNA.	25813	magenta
ILMN_1672417	PTPRCAP	protein tyrosine phosphatase, receptor type, C-associated protein (PTPRCAP), mRNA.	5790	magenta
ILMN_1672443	QDPR	quinoid dihydropteridine reductase (QDPR), mRNA.	5860	magenta
ILMN_1673026	CHCHD3	coiled-coil-helix-coiled-coil-helix domain containing 3 (CHCHD3), mRNA.	54927	magenta

ILMN_1678052	C19orf24	chromosome 19 open reading frame 24 (C19orf24), mRNA.	55009	magenta
ILMN_1679880	THOC6	THO complex 6 homolog (Drosophila) (THOC6), mRNA.	79228	magenta
ILMN_1679949	SLC25A23	solute carrier family 25 (mitochondrial carrier; phosphate carrier), member 23 (SLC25A23), mRNA.	79085	magenta
ILMN_1681802	GRK6	G protein-coupled receptor kinase 6 (GRK6), transcript variant 2, mRNA.	2870	magenta
ILMN_1681998	AP2B1	adaptor-related protein complex 2, beta 1 subunit (AP2B1), transcript variant 1, mRNA.	163	magenta
ILMN_1683660	EIF3H	eukaryotic translation initiation factor 3, subunit 3 gamma, 40kDa (EIF3S3), mRNA.	8667	magenta
ILMN_1684217	AURKB	aurora kinase B (AURKB), mRNA.	9212	magenta
ILMN_1684929	TOPBP1	topoisomerase (DNA) II binding protein 1 (TOPBP1), mRNA.	11073	magenta
ILMN_1685725	ILDR1	immunoglobulin-like domain containing receptor 1 (ILDR1), mRNA.	286676	magenta
ILMN_1686748	TMEM9	transmembrane protein 9 (TMEM9), mRNA.	252839	magenta
ILMN_1688959	CD27	CD27 molecule (CD27), mRNA.	939	magenta
ILMN_1689001	CDK4	cyclin-dependent kinase 4 (CDK4), mRNA.	1019	magenta
ILMN_1689110	NOB1	NIN1/RPN12 binding protein 1 homolog (S. cerevisiae) (NOB1), mRNA.	28987	magenta
ILMN_1689342	NUBP1	nucleotide binding protein 1 (MinD homolog, E. coli) (NUBP1), mRNA.	4682	magenta
ILMN_1689446	EIF3G	eukaryotic translation initiation factor 3, subunit 4 delta, 44kDa (EIF3S4), mRNA.	8666	magenta
ILMN_1689800	MRTO4	mRNA turnover 4 homolog (S. cerevisiae) (MRTO4), mRNA.	51154	magenta
ILMN_1690494	RPL6	ribosomal protein L6 (RPL6), transcript variant 2, mRNA.	6128	magenta
ILMN_1690610	RALY	RNA binding protein, autoantigenic (hnRNP-associated with lethal yellow homolog (mouse)) (RALY), transcript variant 1, mRNA.	22913	magenta
ILMN_1690653	CDK2AP2	CDK2-associated protein 2 (CDK2AP2), mRNA.	10263	magenta
ILMN_1691090	MPV17	MpV17 mitochondrial inner membrane protein (MPV17), nuclear gene encoding mitochondrial protein, mRNA.	4358	magenta
ILMN_1691795	C19orf43	chromosome 19 open reading frame 43 (C19orf43), mRNA.	79002	magenta

ILMN_1691843	RNPS1	RNA binding protein S1, serine-rich domain (RNPS1), transcript variant 1, mRNA.	10921	magenta
ILMN_1692092	WWP1	WW domain containing E3 ubiquitin protein ligase 1 (WWP1), mRNA.	11059	magenta
ILMN_1693430	NME1-NME2	NM23-LV (NME1-NME2), mRNA.	654364	magenta
ILMN_1694742	RPS29	ribosomal protein S29 (RPS29), transcript variant 1, mRNA.	6235	magenta
ILMN_1694950	MRPL28	mitochondrial ribosomal protein L28 (MRPL28), nuclear gene encoding mitochondrial protein, mRNA.	10573	magenta
ILMN_1695420	CLTA	clathrin, light chain (Lca) (CLTA), transcript variant 1, mRNA.	1211	magenta
ILMN_1696640	TCHP	trichoplein, keratin filament binding (TCHP), mRNA.	84260	magenta
ILMN_1696952	SEC61A1	Sec61 alpha 1 subunit (S. cerevisiae) (SEC61A1), mRNA.	29927	magenta
ILMN_1697777	TSTA3	tissue specific transplantation antigen P35B (TSTA3), mRNA.	7264	magenta
ILMN_1698491	MBD3	methyl-CpG binding domain protein 3 (MBD3), mRNA.	53615	magenta
ILMN_1699598	AP2M1	adaptor-related protein complex 2, mu 1 subunit (AP2M1), transcript variant 2, mRNA.	1173	magenta
ILMN_1699603	MRPL12	mitochondrial ribosomal protein L12 (MRPL12), nuclear gene encoding mitochondrial protein, mRNA.	6182	magenta
ILMN_1699737	TRAP1	TNF receptor-associated protein 1 (TRAP1), mRNA.	10131	magenta
ILMN_1703305	TWF2	twinfilin, actin-binding protein, homolog 2 (Drosophila) (TWF2), mRNA.	11344	magenta
ILMN_1703370	ZDHHC12	zinc finger, DHHC-type containing 12 (ZDHHC12), mRNA.	84885	magenta
ILMN_1704055	NA	hypothetical protein HSPC111 (HSPC111), mRNA.	51491	magenta
ILMN_1704253	C6orf106	chromosome 6 open reading frame 106 (C6orf106), transcript variant 2, mRNA.	64771	magenta
ILMN_1705117	SEC13	SEC13 homolog (S. cerevisiae) (SEC13), mRNA.	6396	magenta
ILMN_1705364	BAG6	HLA-B associated transcript 3 (BAT3), transcript variant 1, mRNA.	7917	magenta
ILMN_1707137	C17orf97	hypothetical gene supported by AK128660 (LOC400566), mRNA.	400566	magenta
ILMN_1708660	RWDD4	RWD domain containing 4A (RWDD4A), mRNA.	201965	magenta
ILMN_1708808	RUVBL2	RuvB-like 2 (E. coli) (RUVBL2), mRNA.	10856	magenta

ILMN_1711543	C14orf169	chromosome 14 open reading frame 169 (C14orf169), mRNA.	79697	magenta
ILMN_1711627	SIAH1	seven in absentia homolog 1 (Drosophila) (SIAH1), transcript variant 2, mRNA.	6477	magenta
ILMN_1713749	CORO1A	coronin, actin binding protein, 1A (CORO1A), mRNA.	11151	magenta
ILMN_1713985	MAF1	MAF1 homolog (S. cerevisiae) (MAF1), mRNA.	84232	magenta
ILMN_1715698	NA	similar to DNA segment, Chr 11, Brigham & Womens Genetics 0434 expressed (MGC71993), mRNA.	440400	magenta
ILMN_1715896	PMVK	phosphomevalonate kinase (PMVK), mRNA.	10654	magenta
ILMN_1717154	AQR	aquarius homolog (mouse) (AQR), mRNA.	9716	magenta
ILMN_1719471	MSH3	mutS homolog 3 (E. coli) (MSH3), mRNA.	4437	magenta
ILMN_1719906	HADH	hydroxyacyl-Coenzyme A dehydrogenase (HADH), nuclear gene encoding mitochondrial protein, mRNA.	3033	magenta
ILMN_1720319	SLC35A4	solute carrier family 35, member A4 (SLC35A4), mRNA.	113829	magenta
ILMN_1721093	TAF10	TAF10 RNA polymerase II, TATA box binding protein (TBP)-associated factor, 30kDa (TAF10), mRNA.	6881	magenta
ILMN_1721337	MRPS18B	mitochondrial ribosomal protein S18B (MRPS18B), nuclear gene encoding mitochondrial protein, mRNA.	28973	magenta
ILMN_1722065	COPG2	coatomer protein complex, subunit gamma 2 (COPG2), mRNA.	26958	magenta
ILMN_1723185	ELOF1	elongation factor 1 homolog (S. cerevisiae) (ELOF1), mRNA.	84337	magenta
ILMN_1723729	RSL1D1	ribosomal L1 domain containing 1 (RSL1D1), mRNA.	26156	magenta
ILMN_1724145	CBX4	chromobox homolog 4 (Pc class homolog, Drosophila) (CBX4), mRNA.	8535	magenta
ILMN_1725642	SUMO3	SMT3 suppressor of mif two 3 homolog 3 (S. cerevisiae) (SUMO3), mRNA.	6612	magenta
ILMN_1726434	UNC45A	unc-45 homolog A (C. elegans) (UNC45A), transcript variant 2, mRNA.	55898	magenta
ILMN_1726466	HDHD3	haloacid dehalogenase-like hydrolase domain containing 3 (HDHD3), mRNA.	81932	magenta
ILMN_1726786	TNRC6B	trinucleotide repeat containing 6B (TNRC6B), transcript variant 2, mRNA.	23112	magenta
ILMN_1727444	PAGR1	chromosome 16 open reading frame 53 (C16orf53), mRNA.	79447	magenta

ILMN_1728047	AKR1A1	aldo-keto reductase family 1, member A1 (aldehyde reductase) (AKR1A1), transcript variant 1, mRNA.	10327	magenta
ILMN_1728355	PSMD4	proteasome (prosome, macropain) 26S subunit, non-ATPase, 4 (PSMD4), transcript variant 2, mRNA.	5710	magenta
ILMN_1729319	USP7	ubiquitin specific peptidase 7 (herpes virus-associated) (USP7), mRNA.	7874	magenta
ILMN_1731851	OXA1L	oxidase (cytochrome c) assembly 1-like (OXA1L), mRNA.	5018	magenta
ILMN_1733616	TFEB	transcription factor EB (TFEB), mRNA.	7942	magenta
ILMN_1733799	FAM195B	hypothetical protein LOC348262 (LOC348262), mRNA.	348262	magenta
ILMN_1733947	FKBP8	FK506 binding protein 8, 38kDa (FKBP8), mRNA.	23770	magenta
ILMN_1736548	PHACTR4	phosphatase and actin regulator 4 (PHACTR4), transcript variant 1, mRNA.	65979	magenta
ILMN_1737074	RPS2	PREDICTED: similar to ribosomal protein S2, transcript variant 3 (LOC440589), mRNA.	440589	magenta
ILMN_1737163	SH3BGRL3	SH3 domain binding glutamic acid-rich protein like 3 (SH3BGRL3), mRNA.	83442	magenta
ILMN_1737344	DDX41	DEAD (Asp-Glu-Ala-Asp) box polypeptide 41 (DDX41), mRNA.	51428	magenta
ILMN_1738103	COPE	coatomer protein complex, subunit epsilon (COPE), transcript variant 2, mRNA.	11316	magenta
ILMN_1738369	TUFM	Tu translation elongation factor, mitochondrial (TUFM), mRNA.	7284	magenta
ILMN_1738572	USP48	ubiquitin specific peptidase 48 (USP48), transcript variant 2, mRNA.	84196	magenta
ILMN_1738652	BAD	BCL2-antagonist of cell death (BAD), transcript variant 2, mRNA.	572	magenta
ILMN_1738767	PLP2	proteolipid protein 2 (colonic epithelium-enriched) (PLP2), mRNA.	5355	magenta
ILMN_1738784	PPP2R5A	protein phosphatase 2, regulatory subunit B', alpha isoform (PPP2R5A), mRNA.	5525	magenta
ILMN_1740737	DCPS	decapping enzyme, scavenger (DCPS), mRNA.	28960	magenta
ILMN_1740976	NONO	non-POU domain containing, octamer-binding (NONO), mRNA.	4841	magenta
ILMN_1741997	SNRPC	small nuclear ribonucleoprotein polypeptide C (SNRPC), mRNA.	6631	magenta
ILMN_1743582	NUDT22	nudix (nucleoside diphosphate linked moiety X)-type motif 22 (NUDT22), mRNA.	84304	magenta
ILMN_1745271	EXOSC4	exosome component 4 (EXOSC4), mRNA.	54512	magenta

ILMN_1745620	KRCC1	lysine-rich coiled-coil 1 (KRCC1), mRNA.	51315	magenta
ILMN_1745760	RANGRF	RAN guanine nucleotide release factor (RANGNRF), mRNA.	29098	magenta
ILMN_1746588	TALDO1	transaldolase 1 (TALDO1), mRNA.	6888	magenta
ILMN_1747419	PCGF2	polycomb group ring finger 2 (PCGF2), mRNA.	7703	magenta
ILMN_1749405	KIAA1191	KIAA1191 (KIAA1191), transcript variant 3, mRNA.	57179	magenta
ILMN_1750100	TUBB8	tubulin, beta polypeptide 4, member Q (TUBB4Q), mRNA.	56604	magenta
ILMN_1750130	GSPT1	G1 to S phase transition 1 (GSPT1), mRNA.	2935	magenta
ILMN_1751431	WIBG	within bgcn homolog (Drosophila) (WIBG), mRNA.	84305	magenta
ILMN_1751561	CAMK1D	calcium/calmodulin-dependent protein kinase ID (CAMK1D), transcript variant 2, mRNA.	57118	magenta
ILMN_1751753	IDH2	isocitrate dehydrogenase 2 (NADP+), mitochondrial (IDH2), mRNA.	3418	magenta
ILMN_1752285	RPL4	ribosomal protein L4 (RPL4), mRNA.	6124	magenta
ILMN_1752423	MAP2K2	mitogen-activated protein kinase kinase 2 (MAP2K2), mRNA.	5605	magenta
ILMN_1752451	CTSH	cathepsin H (CTSH), transcript variant 2, mRNA.	1512	magenta
ILMN_1752582	RAB5B	RAB5B, member RAS oncogene family (RAB5B), mRNA.	5869	magenta
ILMN_1752967	DHPS	deoxyhypusine synthase (DHPS), transcript variant 3, mRNA.	1725	magenta
ILMN_1755321	AAAS	achalasia, adrenocortical insufficiency, alacrimia (Allgrove, triple-A) (AAAS), mRNA.	8086	magenta
ILMN_1756126	STUB1	STIP1 homology and U-box containing protein 1 (STUB1), mRNA.	10273	magenta
ILMN_1756439	SCRN1	secernin 1 (SCRN1), mRNA.	9805	magenta
ILMN_1758640	NDUFA10	PREDICTED: similar to NADH dehydrogenase (ubiquinone) 1 alpha subcomplex, 10, 42kDa precursor (LOC732160), mRNA.	732160	magenta
ILMN_1759075	TNFRSF13B	tumor necrosis factor receptor superfamily, member 13B (TNFRSF13B), mRNA.	23495	magenta
ILMN_1759341	MAN2B1	mannosidase, alpha, class 2B, member 1 (MAN2B1), mRNA.	4125	magenta
ILMN_1760708	NA	crystallin, beta B2 (CRYBB2), mRNA.	1415	magenta
ILMN_1762316	CPSF3L	cleavage and polyadenylation specific factor 3-like (CPSF3L), mRNA.	54973	magenta

ILMN_1762615	FAM175B	KIAA0157 (KIAA0157), mRNA.	23172	magenta
ILMN_1763705	WRNIP1	Werner helicase interacting protein 1 (WRNIP1), transcript variant 2, mRNA.	56897	magenta
ILMN_1763824	PTGES2	prostaglandin E synthase 2 (PTGES2), transcript variant 1, mRNA.	80142	magenta
ILMN_1765257	CINP	cyclin-dependent kinase 2-interacting protein (CINP), mRNA.	51550	magenta
ILMN_1765258	HLA-E	major histocompatibility complex, class I, E (HLA-E), mRNA.	3133	magenta
ILMN_1765621	HDGF	hepatoma-derived growth factor (high-mobility group protein 1-like) (HDGF), mRNA.	3068	magenta
ILMN_1767324	EIF4EBP1	eukaryotic translation initiation factor 4E binding protein 1 (EIF4EBP1), mRNA.	1978	magenta
ILMN_1767365	PAK1	p21/Cdc42/Rac1-activated kinase 1 (STE20 homolog, yeast) (PAK1), mRNA.	5058	magenta
ILMN_1768181	TOR3A	torsin family 3, member A (TOR3A), mRNA.	64222	magenta
ILMN_1768662	UCK2	uridine-cytidine kinase 2 (UCK2), mRNA.	7371	magenta
ILMN_1768773	NA	PREDICTED: similar to cytochrome P450 monooxygenase CYP2T1 (LOC731986), mRNA.	731986	magenta
ILMN_1768867	AP3B1	adaptor-related protein complex 3, beta 1 subunit (AP3B1), mRNA.	8546	magenta
ILMN_1769191	GNAS	GNAS complex locus (GNAS), transcript variant 1, mRNA.	2778	magenta
ILMN_1770244	CBX1	chromobox homolog 1 (HP1 beta homolog Drosophila) (CBX1), mRNA.	10951	magenta
ILMN_1770641	KLHL3	kelch-like 3 (Drosophila) (KLHL3), mRNA.	26249	magenta
ILMN_1770817	CAPNS1	calpain, small subunit 1 (CAPNS1), transcript variant 2, mRNA.	826	magenta
ILMN_1772113	U2AF1	U2 small nuclear RNA auxiliary factor 1 (U2AF1), transcript variant a, mRNA.	7307	magenta
ILMN_1772527	ATG101	chromosome 12 open reading frame 44 (C12orf44), mRNA.	60673	magenta
ILMN_1772798	ARPP19	cyclic AMP phosphoprotein, 19 kD (ARPP-19), mRNA.	10776	magenta
ILMN_1773228	DLST	dihydrolipoamide S-succinyltransferase (E2 component of 2-oxo-glutarate complex) (DLST), mRNA.	1743	magenta
ILMN_1773313	NA	upregulated during skeletal muscle growth 5 homolog (mouse) (USMG5), mRNA.	84833	magenta
ILMN_1773935	TMEM165	transmembrane protein 165 (TMEM165), mRNA.	55858	magenta
ILMN_1774079	NUDC	nuclear distribution gene C homolog (A. nidulans) (NUDC), mRNA.	10726	magenta

ILMN_1774432	DTD1	D-tyrosyl-tRNA deacylase 1 homolog (S. cerevisiae) (DTD1), mRNA.	92675	magenta
ILMN_1775074	TUBGCP2	tubulin, gamma complex associated protein 2 (TUBGCP2), mRNA.	10844	magenta
ILMN_1776147	C21orf59	chromosome 21 open reading frame 59 (C21orf59), mRNA.	56683	magenta
ILMN_1776577	DSCC1	defective in sister chromatid cohesion homolog 1 (S. cerevisiae) (DCC1), mRNA.	79075	magenta
ILMN_1777584	KARS	lysyl-tRNA synthetase (KARS), mRNA.	3735	magenta
ILMN_1777794	PRKCSH	protein kinase C substrate 80K-H (PRKCSH), transcript variant 1, mRNA.	5589	magenta
ILMN_1778255	FARSA	phenylalanyl-tRNA synthetase, alpha subunit (FARSA), mRNA.	2193	magenta
ILMN_1780315	PUSL1	pseudouridylate synthase-like 1 (PUSL1), mRNA.	126789	magenta
ILMN_1780769	TUBB4B	tubulin, beta 2C (TUBB2C), mRNA.	10383	magenta
ILMN_1781906	RBM17	RNA binding motif protein 17 (RBM17), mRNA.	84991	magenta
ILMN_1782543	EEF1D	eukaryotic translation elongation factor 1 delta (guanine nucleotide exchange protein) (EEF1D), transcript variant 2, mRNA.	1936	magenta
ILMN_1782618	C9orf16	chromosome 9 open reading frame 16 (C9orf16), mRNA.	79095	magenta
ILMN_1783753	TXNDC12	thioredoxin domain containing 12 (endoplasmic reticulum) (TXNDC12), mRNA.	51060	magenta
ILMN_1786212	TUBB8	tubulin, beta 8 (TUBB8), mRNA.	347688	magenta
ILMN_1787410	EIF6	integrin beta 4 binding protein (ITGB4BP), transcript variant 4, mRNA.	3692	magenta
ILMN_1789233	VPS37C	vacuolar protein sorting 37 homolog C (S. cerevisiae) (VPS37C), mRNA.	55048	magenta
ILMN_1790797	VPS28	vacuolar protein sorting 28 homolog (S. cerevisiae) (VPS28), transcript variant 2, mRNA.	51160	magenta
ILMN_1792748	CPS1	carbamoyl-phosphate synthetase 1, mitochondrial (CPS1), mRNA.	1373	magenta
ILMN_1793651	UBE2N	ubiquitin-conjugating enzyme E2N (UBC13 homolog, yeast) (UBE2N), mRNA.	7334	magenta
ILMN_1795826	ATP6V0D1	ATPase, H+ transporting, lysosomal 38kDa, V0 subunit d1 (ATP6V0D1), mRNA.	9114	magenta
ILMN_1796430	PSMD3	proteasome (prosome, macropain) 26S subunit, non-ATPase, 3 (PSMD3), mRNA.	5709	magenta

ILMN_1797005	PGLS	6-phosphogluconolactonase (PGLS), mRNA.	25796	magenta
ILMN_1797522	DUSP3	dual specificity phosphatase 3 (vaccinia virus phosphatase VH1-related) (DUSP3), mRNA.	1845	magenta
ILMN_1798061	ZFYVE26	zinc finger, FYVE domain containing 26 (ZFYVE26), mRNA.	23503	magenta
ILMN_1799024	VAC14	Vac14 homolog (S. cerevisiae) (VAC14), mRNA.	55697	magenta
ILMN_1799951	LYST	lysosomal trafficking regulator (LYST), transcript variant 1, mRNA.	1130	magenta
ILMN_1800461	CSNK2B	casein kinase 2, beta polypeptide (CSNK2B), mRNA.	1460	magenta
ILMN_1800976	NFATC3	nuclear factor of activated T-cells, cytoplasmic, calcineurin-dependent 3 (NFATC3), transcript variant 2, mRNA.	4775	magenta
ILMN_1801118	SNRNP25	chromosome 16 open reading frame 33 (C16orf33), mRNA.	79622	magenta
ILMN_1801313	SIAH2	seven in absentia homolog 2 (Drosophila) (SIAH2), mRNA.	6478	magenta
ILMN_1801913	PPIH	peptidylprolyl isomerase H (cyclophilin H) (PPIH), mRNA.	10465	magenta
ILMN_1802252	GAPDH	glyceraldehyde-3-phosphate dehydrogenase (GAPDH), mRNA.	2597	magenta
ILMN_1802706	IDH3G	isocitrate dehydrogenase 3 (NAD+) gamma (IDH3G), nuclear gene encoding mitochondrial protein, transcript variant 1, mRNA.	3421	magenta
ILMN_1803277	MVP	major vault protein (MVP), transcript variant 2, mRNA.	9961	magenta
ILMN_1803772	POLD4	polymerase (DNA-directed), delta 4 (POLD4), mRNA.	57804	magenta
ILMN_1805990	BAK1	BCL2-antagonist/killer 1 (BAK1), mRNA.	578	magenta
ILMN_1806605	FAHD2B	PREDICTED: similar to fumarylacetoacetate hydrolase domain containing 2A (LOC731002), mRNA.	731002	magenta
ILMN_1806937	BABAM1	HSPC142 protein (HSPC142), transcript variant 1, mRNA.	29086	magenta
ILMN_1807201	FAM104A	family with sequence similarity 104, member A (FAM104A), mRNA.	84923	magenta
ILMN_1809437	RHBDD2	rhomboid domain containing 2 (RHBDD2), transcript variant 2, mRNA.	57414	magenta
ILMN_1809818	PRCC	papillary renal cell carcinoma (translocation-associated) (PRCC), transcript variant 1, mRNA.	5546	magenta
ILMN_1811775	CCDC124	coiled-coil domain containing 124 (CCDC124), mRNA.	115098	magenta

ILMN_1813671	SLC25A1	solute carrier family 25 (mitochondrial carrier; citrate transporter), member 1 (SLC25A1), mRNA.	6576	magenta
ILMN_1814213	PQLC3	PQ loop repeat containing 3 (PQLC3), mRNA.	130814	magenta
ILMN_1814589	KIF22	kinesin family member 22 (KIF22), mRNA.	3835	magenta
ILMN_1815169	MCM5	MCM5 minichromosome maintenance deficient 5, cell division cycle 46 (S. cerevisiae) (MCM5), mRNA.	4174	magenta
ILMN_1815402	LPAR5	G protein-coupled receptor 92 (GPR92), mRNA.	57121	magenta

Table S5. List of Unique validated gene targets of three ICS response associated miRs.

**mature_mirna_id**	**target_symbol**	**target_entrez**
hsa-miR-28-5p	CALM3	808
hsa-miR-28-5p	CCND1	595
hsa-miR-28-5p	CCNT1	904
hsa-miR-28-5p	CHD1	1105
hsa-miR-28-5p	EGFR	1956
hsa-miR-28-5p	FKBP5	2289
hsa-miR-28-5p	GNB1	2782
hsa-miR-28-5p	GNG2	54331
hsa-miR-28-5p	H2BC7	8343
hsa-miR-28-5p	IGF1R	3480
hsa-miR-28-5p	MAPK1	5594
hsa-miR-28-5p	POLR2A	5430
hsa-miR-28-5p	POU2F1	5451
hsa-miR-28-5p	PPP5C	5536
hsa-miR-28-5p	PRMT1	3276
hsa-miR-28-5p	SMC3	9126
hsa-miR-28-5p	SRF	6722
hsa-miR-28-5p	TNRC6B	23112
hsa-miR-28-5p	XPO1	7514
hsa-miR-28-5p	ZNF217	7764
hsa-miR-28-5p	AAAS	8086
hsa-miR-28-5p	AAR2	25980
hsa-miR-28-5p	ABI2	10152
hsa-miR-28-5p	ACBD4	79777
hsa-miR-28-5p	ACE	1636
hsa-miR-28-5p	ACTB	60

hsa-miR-28-5p	ACTR2	10097
hsa-miR-28-5p	ACVR1B	91
hsa-miR-28-5p	ACVR2B	93
hsa-miR-28-5p	ADA2	51816
hsa-miR-28-5p	ADGRV1	84059
hsa-miR-28-5p	ADSS2	159
hsa-miR-28-5p	AGFG1	3267
hsa-miR-28-5p	AGPAT3	56894
hsa-miR-28-5p	AGPAT4	56895
hsa-miR-28-5p	AGPS	8540
hsa-miR-28-5p	AGRN	375790
hsa-miR-28-5p	AHDC1	27245
hsa-miR-28-5p	AHNAK	79026
hsa-miR-28-5p	AKAP11	11215
hsa-miR-28-5p	AL096711.2	NA
hsa-miR-28-5p	ALKBH5	54890
hsa-miR-28-5p	ANAPC2	29882
hsa-miR-28-5p	ANKRD52	283373
hsa-miR-28-5p	ANKZF1	55139
hsa-miR-28-5p	ANXA11	311
hsa-miR-28-5p	AP1B1	162
hsa-miR-28-5p	AP2B1	163
hsa-miR-28-5p	APEX1	328
hsa-miR-28-5p	APMAP	57136
hsa-miR-28-5p	APOBEC3C	27350
hsa-miR-28-5p	APOL3	80833
hsa-miR-28-5p	APOOL	139322
hsa-miR-28-5p	ARF3	377
hsa-miR-28-5p	ARFGEF2	10564
hsa-miR-28-5p	ARGLU1	55082
hsa-miR-28-5p	ARHGAP42	143872
hsa-miR-28-5p	ARHGEF9	23229
hsa-miR-28-5p	ARPP19	10776
hsa-miR-28-5p	ASB1	51665
hsa-miR-28-5p	ASF1A	25842
hsa-miR-28-5p	ASH1L	55870
hsa-miR-28-5p	ATG9A	79065
hsa-miR-28-5p	ATMIN	23300
hsa-miR-28-5p	ATN1	1822
hsa-miR-28-5p	ATP13A2	23400

hsa-miR-28-5p	ATP2A2	488
hsa-miR-28-5p	ATP9A	10079
hsa-miR-28-5p	ATRAID	51374
hsa-miR-28-5p	B4GALNT4	338707
hsa-miR-28-5p	B4GALT1	2683
hsa-miR-28-5p	BAG1	573
hsa-miR-28-5p	BAZ2A	11176
hsa-miR-28-5p	BBS10	79738
hsa-miR-28-5p	BCL11B	64919
hsa-miR-28-5p	BCL9	607
hsa-miR-28-5p	BCOR	54880
hsa-miR-28-5p	BECN1	8678
hsa-miR-28-5p	BHLHE40	8553
hsa-miR-28-5p	BIRC6	57448
hsa-miR-28-5p	BIVM	54841
hsa-miR-28-5p	BLCAP	10904
hsa-miR-28-5p	BLMH	642
hsa-miR-28-5p	BMPR2	659
hsa-miR-28-5p	BRD4	23476
hsa-miR-28-5p	BST2	684
hsa-miR-28-5p	BTBD7	55727
hsa-miR-28-5p	BTG1	694
hsa-miR-28-5p	BTG2	7832
hsa-miR-28-5p	BTLA	151888
hsa-miR-28-5p	BTRC	8945
hsa-miR-28-5p	C16orf72	29035
hsa-miR-28-5p	C16orf74	404550
hsa-miR-28-5p	C1QTNF1	114897
hsa-miR-28-5p	C5orf63	401207
hsa-miR-28-5p	C6orf62	81688
hsa-miR-28-5p	CAMK2G	818
hsa-miR-28-5p	CAMK2N1	55450
hsa-miR-28-5p	CAND1	55832
hsa-miR-28-5p	CASP2	835
hsa-miR-28-5p	CASTOR2	729438
hsa-miR-28-5p	CBL	867
hsa-miR-28-5p	CBX5	23468
hsa-miR-28-5p	CCDC6	8030
hsa-miR-28-5p	CCNB1	891
hsa-miR-28-5p	CCND2	894

hsa-miR-28-5p	CCND3	896
hsa-miR-28-5p	CCT3	7203
hsa-miR-28-5p	CD276	80381
hsa-miR-28-5p	CD47	961
hsa-miR-28-5p	CD52	1043
hsa-miR-28-5p	CD99L2	83692
hsa-miR-28-5p	CDCA7L	55536
hsa-miR-28-5p	CDK4	1019
hsa-miR-28-5p	CDKN1A	1026
hsa-miR-28-5p	CDS1	1040
hsa-miR-28-5p	CELF1	10658
hsa-miR-28-5p	CELF2	10659
hsa-miR-28-5p	CENPB	1059
hsa-miR-28-5p	CENPF	1063
hsa-miR-28-5p	CENPJ	55835
hsa-miR-28-5p	CENPN	55839
hsa-miR-28-5p	CENPV	201161
hsa-miR-28-5p	CEP162	22832
hsa-miR-28-5p	CFL2	1073
hsa-miR-28-5p	CHERP	10523
hsa-miR-28-5p	CHP1	11261
hsa-miR-28-5p	CHST3	9469
hsa-miR-28-5p	CISH	1154
hsa-miR-28-5p	CLNS1A	1207
hsa-miR-28-5p	CLPTM1L	81037
hsa-miR-28-5p	CMC4	100272147
hsa-miR-28-5p	CMTM4	146223
hsa-miR-28-5p	CNN3	1266
hsa-miR-28-5p	CNOT2	4848
hsa-miR-28-5p	CNOT6	57472
hsa-miR-28-5p	CNOT6L	246175
hsa-miR-28-5p	COA7	65260
hsa-miR-28-5p	COG7	91949
hsa-miR-28-5p	COL1A1	1277
hsa-miR-28-5p	COL1A2	1278
hsa-miR-28-5p	COL6A2	1292
hsa-miR-28-5p	COPB2	9276
hsa-miR-28-5p	CPE	1363
hsa-miR-28-5p	CPSF3	51692
hsa-miR-28-5p	CPSF7	79869

hsa-miR-28-5p	CRISPLD2	83716
hsa-miR-28-5p	CRKL	1399
hsa-miR-28-5p	CRTC3	64784
hsa-miR-28-5p	CRYBG1	202
hsa-miR-28-5p	CS	1431
hsa-miR-28-5p	CSE1L	1434
hsa-miR-28-5p	CSNK1D	1453
hsa-miR-28-5p	CSNK1G1	53944
hsa-miR-28-5p	CSTF2T	23283
hsa-miR-28-5p	CTDNEP1	23399
hsa-miR-28-5p	CTIF	9811
hsa-miR-28-5p	CTNNB1	1499
hsa-miR-28-5p	CTNND1	1500
hsa-miR-28-5p	CTTNBP2	83992
hsa-miR-28-5p	CWC27	10283
hsa-miR-28-5p	CXCL5	6374
hsa-miR-28-5p	CYB5B	80777
hsa-miR-28-5p	CYP1B1	1545
hsa-miR-28-5p	D2HGDH	728294
hsa-miR-28-5p	DAAM2	23500
hsa-miR-28-5p	DCAF10	79269
hsa-miR-28-5p	DCP2	167227
hsa-miR-28-5p	DCTN5	84516
hsa-miR-28-5p	DDAH1	23576
hsa-miR-28-5p	DDB1	1642
hsa-miR-28-5p	DDIT4	54541
hsa-miR-28-5p	DDR1	780
hsa-miR-28-5p	DDT	100037417
hsa-miR-28-5p	DDTL	100037417
hsa-miR-28-5p	DDX21	9188
hsa-miR-28-5p	DDX56	54606
hsa-miR-28-5p	DENND4B	9909
hsa-miR-28-5p	DGCR2	9993
hsa-miR-28-5p	DGCR8	54487
hsa-miR-28-5p	DHX16	8449
hsa-miR-28-5p	DHX33	56919
hsa-miR-28-5p	DIAPH1	1729
hsa-miR-28-5p	DICER1	23405
hsa-miR-28-5p	DIP2C	22982
hsa-miR-28-5p	DIPK1B	138311

hsa-miR-28-5p	DLG2	1740
hsa-miR-28-5p	DLG4	1742
hsa-miR-28-5p	DNAH10	196385
hsa-miR-28-5p	DNAJA4	55466
hsa-miR-28-5p	DNAJB14	79982
hsa-miR-28-5p	DNAJB4	11080
hsa-miR-28-5p	DNAJB6	10049
hsa-miR-28-5p	DNMBP	23268
hsa-miR-28-5p	DOCK6	57572
hsa-miR-28-5p	DOK3	79930
hsa-miR-28-5p	DRAXIN	374946
hsa-miR-28-5p	DST	667
hsa-miR-28-5p	DUSP18	150290
hsa-miR-28-5p	DUSP8	1850
hsa-miR-28-5p	DYNC1H1	1778
hsa-miR-28-5p	DYNC1LI2	1783
hsa-miR-28-5p	DYNLL2	140735
hsa-miR-28-5p	DYRK1B	9149
hsa-miR-28-5p	DYRK2	8445
hsa-miR-28-5p	DYRK4	8798
hsa-miR-28-5p	E2F3	1871
hsa-miR-28-5p	E2F6	1876
hsa-miR-28-5p	EDA2R	60401
hsa-miR-28-5p	EEF1D	1936
hsa-miR-28-5p	EEF2	1938
hsa-miR-28-5p	EHD2	30846
hsa-miR-28-5p	EIF2AK1	27102
hsa-miR-28-5p	EIF4A2	1974
hsa-miR-28-5p	EIF4B	1975
hsa-miR-28-5p	EIF4EBP2	1979
hsa-miR-28-5p	EIF4G1	1981
hsa-miR-28-5p	EIF4G2	1982
hsa-miR-28-5p	EMC10	284361
hsa-miR-28-5p	EME2	197342
hsa-miR-28-5p	EMILIN2	84034
hsa-miR-28-5p	EMP3	2014
hsa-miR-28-5p	EN2	2020
hsa-miR-28-5p	ENO2	2026
hsa-miR-28-5p	ENPP5	59084
hsa-miR-28-5p	ENTPD4	9583

hsa-miR-28-5p	EPDR1	54749
hsa-miR-28-5p	EPG5	57724
hsa-miR-28-5p	EPHA4	2043
hsa-miR-28-5p	ERAP1	51752
hsa-miR-28-5p	ERG28	11161
hsa-miR-28-5p	ERI2	112479
hsa-miR-28-5p	ETNK1	55500
hsa-miR-28-5p	EYA3	2140
hsa-miR-28-5p	EZR	7430
hsa-miR-28-5p	FAM102A	399665
hsa-miR-28-5p	FAM168A	23201
hsa-miR-28-5p	FAM171B	165215
hsa-miR-28-5p	FAM174C	55009
hsa-miR-28-5p	FAM189B	10712
hsa-miR-28-5p	FAM193B	54540
hsa-miR-28-5p	FAM210A	125228
hsa-miR-28-5p	FAM217B	63939
hsa-miR-28-5p	FAM53C	51307
hsa-miR-28-5p	FAR1	84188
hsa-miR-28-5p	FARP2	9855
hsa-miR-28-5p	FARSA	2193
hsa-miR-28-5p	FASTKD5	60493
hsa-miR-28-5p	FAT4	79633
hsa-miR-28-5p	FBN1	2200
hsa-miR-28-5p	FBXL19	54620
hsa-miR-28-5p	FBXL5	26234
hsa-miR-28-5p	FBXO25	26260
hsa-miR-28-5p	FBXO44	93611
hsa-miR-28-5p	FBXW2	26190
hsa-miR-28-5p	FGF1	2246
hsa-miR-28-5p	FLT1	2321
hsa-miR-28-5p	FNIP1	96459
hsa-miR-28-5p	FOXJ3	22887
hsa-miR-28-5p	FOXK1	221937
hsa-miR-28-5p	FRS2	10818
hsa-miR-28-5p	FTL	2512
hsa-miR-28-5p	FTSJ1	24140
hsa-miR-28-5p	G3BP1	10146
hsa-miR-28-5p	GABBR1	2550
hsa-miR-28-5p	GABPA	2551

hsa-miR-28-5p	GABPB2	126626
hsa-miR-28-5p	GABRA1	2554
hsa-miR-28-5p	GALNT1	2589
hsa-miR-28-5p	GAREM2	150946
hsa-miR-28-5p	GATM	2628
hsa-miR-28-5p	GEMIN4	50628
hsa-miR-28-5p	GFI1	2672
hsa-miR-28-5p	GIGYF1	64599
hsa-miR-28-5p	GIT1	28964
hsa-miR-28-5p	GLUL	2752
hsa-miR-28-5p	GNA12	2768
hsa-miR-28-5p	GNAS	2778
hsa-miR-28-5p	GNS	2799
hsa-miR-28-5p	GPHN	10243
hsa-miR-28-5p	GPR107	57720
hsa-miR-28-5p	GPR137C	283554
hsa-miR-28-5p	GPX7	2882
hsa-miR-28-5p	GRK3	157
hsa-miR-28-5p	GRK6	2870
hsa-miR-28-5p	GSK3A	2931
hsa-miR-28-5p	GSPT2	23708
hsa-miR-28-5p	GTF3C4	9329
hsa-miR-28-5p	GUCY1A2	2977
hsa-miR-28-5p	H1-10	8971
hsa-miR-28-5p	H1-2	3006
hsa-miR-28-5p	H1-4	3008
hsa-miR-28-5p	H1-5	3009
hsa-miR-28-5p	HACE1	57531
hsa-miR-28-5p	HCFC2	29915
hsa-miR-28-5p	HCN2	610
hsa-miR-28-5p	HDAC8	55869
hsa-miR-28-5p	HDGF	3068
hsa-miR-28-5p	HEXB	3074
hsa-miR-28-5p	HEXD	284004
hsa-miR-28-5p	HEY1	23462
hsa-miR-28-5p	HIPK1	204851
hsa-miR-28-5p	HMX3	340784
hsa-miR-28-5p	HNRNPA3	220988
hsa-miR-28-5p	HNRNPC	3183
hsa-miR-28-5p	HNRNPH2	3188

hsa-miR-28-5p	HNRNPK	3190
hsa-miR-28-5p	HNRNPU	3192
hsa-miR-28-5p	HOXA5	3202
hsa-miR-28-5p	HPS4	89781
hsa-miR-28-5p	HSP90B1	7184
hsa-miR-28-5p	HSPA14	51182
hsa-miR-28-5p	HSPA1A	3303
hsa-miR-28-5p	HSPA1B	3304
hsa-miR-28-5p	HSPG2	3339
hsa-miR-28-5p	HYI	81888
hsa-miR-28-5p	ICE2	79664
hsa-miR-28-5p	ICOSLG	23308
hsa-miR-28-5p	IDE	3416
hsa-miR-28-5p	IER2	9592
hsa-miR-28-5p	IER3	8870
hsa-miR-28-5p	IER5	51278
hsa-miR-28-5p	IFNAR2	3455
hsa-miR-28-5p	IGF1	3479
hsa-miR-28-5p	IGF2BP1	10642
hsa-miR-28-5p	IKBKB	3551
hsa-miR-28-5p	IL2RG	3561
hsa-miR-28-5p	IL34	146433
hsa-miR-28-5p	IMPDH1	3614
hsa-miR-28-5p	IPO9	55705
hsa-miR-28-5p	IQGAP1	8826
hsa-miR-28-5p	IQSEC2	23096
hsa-miR-28-5p	IRS4	8471
hsa-miR-28-5p	ISYNA1	51477
hsa-miR-28-5p	ITM2C	81618
hsa-miR-28-5p	JAGN1	84522
hsa-miR-28-5p	JAM2	58494
hsa-miR-28-5p	KANK4	163782
hsa-miR-28-5p	KAT6A	7994
hsa-miR-28-5p	KAT7	11143
hsa-miR-28-5p	KCMF1	56888
hsa-miR-28-5p	KCNH2	3757
hsa-miR-28-5p	KCNK17	89822
hsa-miR-28-5p	KCTD10	83892
hsa-miR-28-5p	KDM5C	8242
hsa-miR-28-5p	KDM6B	23135

hsa-miR-28-5p	KHNYN	23351
hsa-miR-28-5p	KIAA0100	9703
hsa-miR-28-5p	KIF1B	23095
hsa-miR-28-5p	KIF5A	3798
hsa-miR-28-5p	KIF5C	3800
hsa-miR-28-5p	KLF3	51274
hsa-miR-28-5p	KLF6	1316
hsa-miR-28-5p	KLF7	8609
hsa-miR-28-5p	KLHDC3	116138
hsa-miR-28-5p	KLHL11	55175
hsa-miR-28-5p	KLHL12	59349
hsa-miR-28-5p	KLHL13	90293
hsa-miR-28-5p	KLHL22	84861
hsa-miR-28-5p	KMT2A	4297
hsa-miR-28-5p	KMT2B	9757
hsa-miR-28-5p	KMT2D	8085
hsa-miR-28-5p	KPNA4	3840
hsa-miR-28-5p	KRBA1	84626
hsa-miR-28-5p	KRCC1	51315
hsa-miR-28-5p	KRT80	144501
hsa-miR-28-5p	LAMC1	3915
hsa-miR-28-5p	LAMP2	3920
hsa-miR-28-5p	LENG8	114823
hsa-miR-28-5p	LHFPL2	10184
hsa-miR-28-5p	LIFR	3977
hsa-miR-28-5p	LIMA1	51474
hsa-miR-28-5p	LINC00346	283487
hsa-miR-28-5p	LMO7	4008
hsa-miR-28-5p	LNPEP	4012
hsa-miR-28-5p	LNPK	80856
hsa-miR-28-5p	LONRF2	164832
hsa-miR-28-5p	LRP8	7804
hsa-miR-28-5p	LRRC4B	94030
hsa-miR-28-5p	LTA4H	4048
hsa-miR-28-5p	LYPD3	27076
hsa-miR-28-5p	LZTS2	84445
hsa-miR-28-5p	MAD2L1	4085
hsa-miR-28-5p	MAL2	114569
hsa-miR-28-5p	MAML1	9794
hsa-miR-28-5p	MANEA	79694

hsa-miR-28-5p	MAP1A	4130
hsa-miR-28-5p	MAP1B	4131
hsa-miR-28-5p	MAP2K3	5606
hsa-miR-28-5p	MAP3K9	4293
hsa-miR-28-5p	MAPKBP1	23005
hsa-miR-28-5p	MAPRE1	22919
hsa-miR-28-5p	MARCHF6	10299
hsa-miR-28-5p	MARCKSL1	65108
hsa-miR-28-5p	MAST4	375449
hsa-miR-28-5p	MAZ	4150
hsa-miR-28-5p	MCM4	4173
hsa-miR-28-5p	MCM7	4176
hsa-miR-28-5p	MCUR1	63933
hsa-miR-28-5p	MED17	9440
hsa-miR-28-5p	MED22	6837
hsa-miR-28-5p	MEF2C	4208
hsa-miR-28-5p	MEF2D	4209
hsa-miR-28-5p	MEIS1	4211
hsa-miR-28-5p	MELTF	4241
hsa-miR-28-5p	METTL14	57721
hsa-miR-28-5p	MGST2	4258
hsa-miR-28-5p	MIDN	90007
hsa-miR-28-5p	MIGA1	374986
hsa-miR-28-5p	MKLN1	4289
hsa-miR-28-5p	MLEC	9761
hsa-miR-28-5p	MLH1	4292
hsa-miR-28-5p	MOGS	7841
hsa-miR-28-5p	MORF4L1	10933
hsa-miR-28-5p	MORF4L2	9643
hsa-miR-28-5p	MPEG1	219972
hsa-miR-28-5p	MPL	4352
hsa-miR-28-5p	MRPS27	23107
hsa-miR-28-5p	MSI2	124540
hsa-miR-28-5p	MSL1	339287
hsa-miR-28-5p	MSMO1	6307
hsa-miR-28-5p	MSN	4478
hsa-miR-28-5p	MT-ATP6	4508
hsa-miR-28-5p	MT-ND1	4535
hsa-miR-28-5p	MT-ND4L	4539
hsa-miR-28-5p	MYH15	22989

hsa-miR-28-5p	MYLK	4638
hsa-miR-28-5p	MYO18A	399687
hsa-miR-28-5p	MYO1B	4430
hsa-miR-28-5p	N4BP1	9683
hsa-miR-28-5p	NA	NA
hsa-miR-28-5p	NAB2	4665
hsa-miR-28-5p	NACC2	138151
hsa-miR-28-5p	NAP1L1	4673
hsa-miR-28-5p	NAP1L2	4674
hsa-miR-28-5p	NAT8L	339983
hsa-miR-28-5p	NAV1	89796
hsa-miR-28-5p	NCDN	23154
hsa-miR-28-5p	NCF4	4689
hsa-miR-28-5p	NCL	4691
hsa-miR-28-5p	NDST1	3340
hsa-miR-28-5p	NECAP2	55707
hsa-miR-28-5p	NEFL	4747
hsa-miR-28-5p	NF1	4763
hsa-miR-28-5p	NFE2L1	4779
hsa-miR-28-5p	NFIC	4782
hsa-miR-28-5p	NFYC	4802
hsa-miR-28-5p	NKIRAS2	28511
hsa-miR-28-5p	NONO	4841
hsa-miR-28-5p	NOP2	4839
hsa-miR-28-5p	NOP53	29997
hsa-miR-28-5p	NOS1AP	9722
hsa-miR-28-5p	NOTCH2	4853
hsa-miR-28-5p	NOTCH3	4854
hsa-miR-28-5p	NPC2	10577
hsa-miR-28-5p	NR2C2	7182
hsa-miR-28-5p	NR2F6	2063
hsa-miR-28-5p	NRBP1	29959
hsa-miR-28-5p	NRF1	4899
hsa-miR-28-5p	NRN1	51299
hsa-miR-28-5p	NSD1	64324
hsa-miR-28-5p	NSD3	54904
hsa-miR-28-5p	NSUN7	79730
hsa-miR-28-5p	NT5C3B	115024
hsa-miR-28-5p	NTMT1	28989
hsa-miR-28-5p	NUDCD3	23386

hsa-miR-28-5p	NUFIP2	57532
hsa-miR-28-5p	NUP188	23511
hsa-miR-28-5p	NUSAP1	51203
hsa-miR-28-5p	OAS2	4939
hsa-miR-28-5p	OAZ1	4946
hsa-miR-28-5p	OAZ2	4947
hsa-miR-28-5p	OLA1	29789
hsa-miR-28-5p	OLFM1	10439
hsa-miR-28-5p	OTUB1	55611
hsa-miR-28-5p	PALB2	79728
hsa-miR-28-5p	PAQR5	54852
hsa-miR-28-5p	PAX6	5080
hsa-miR-28-5p	PCGF5	84333
hsa-miR-28-5p	PCLAF	9768
hsa-miR-28-5p	PCSK9	255738
hsa-miR-28-5p	PCYOX1	51449
hsa-miR-28-5p	PDIA6	10130
hsa-miR-28-5p	PDK3	5165
hsa-miR-28-5p	PER2	8864
hsa-miR-28-5p	PGK1	5230
hsa-miR-28-5p	PGM1	5236
hsa-miR-28-5p	PHF10	55274
hsa-miR-28-5p	PHF21A	51317
hsa-miR-28-5p	PHLDB2	90102
hsa-miR-28-5p	PIANP	196500
hsa-miR-28-5p	PIAS1	8554
hsa-miR-28-5p	PITRM1	10531
hsa-miR-28-5p	PLAC8	51316
hsa-miR-28-5p	PLCD3	113026
hsa-miR-28-5p	PLEKHG1	57480
hsa-miR-28-5p	PLEKHG5	57449
hsa-miR-28-5p	PLIN5	440503
hsa-miR-28-5p	PLPPR4	9890
hsa-miR-28-5p	PNRC1	10957
hsa-miR-28-5p	PODXL	5420
hsa-miR-28-5p	POLK	51426
hsa-miR-28-5p	POM121C	100101267
hsa-miR-28-5p	POSTN	10631
hsa-miR-28-5p	PPP1R16B	26051
hsa-miR-28-5p	PPP2CB	5516

hsa-miR-28-5p	PPP2R1B	5519
hsa-miR-28-5p	PPP2R5E	5529
hsa-miR-28-5p	PRDM10	56980
hsa-miR-28-5p	PRDM2	7799
hsa-miR-28-5p	PRELID3B	51012
hsa-miR-28-5p	PRKAA1	5562
hsa-miR-28-5p	PRKD2	25865
hsa-miR-28-5p	PRKDC	5591
hsa-miR-28-5p	PROSER1	80209
hsa-miR-28-5p	PRPF38B	55119
hsa-miR-28-5p	PRRC2A	7916
hsa-miR-28-5p	PRRC2B	84726
hsa-miR-28-5p	PRSS16	10279
hsa-miR-28-5p	PSENEN	55851
hsa-miR-28-5p	PSMC5	5705
hsa-miR-28-5p	PSMF1	9491
hsa-miR-28-5p	PTBP3	9991
hsa-miR-28-5p	PTEN	5728
hsa-miR-28-5p	PTOV1	53635
hsa-miR-28-5p	PTPN13	5783
hsa-miR-28-5p	PTPRJ	5795
hsa-miR-28-5p	PUDP	8226
hsa-miR-28-5p	PURB	5814
hsa-miR-28-5p	PYGO2	90780
hsa-miR-28-5p	QRICH1	54870
hsa-miR-28-5p	QSOX1	5768
hsa-miR-28-5p	R3HDM4	91300
hsa-miR-28-5p	RAB11FIP2	22841
hsa-miR-28-5p	RAB11FIP5	26056
hsa-miR-28-5p	RAB14	51552
hsa-miR-28-5p	RAB18	22931
hsa-miR-28-5p	RAB30	27314
hsa-miR-28-5p	RAB36	9609
hsa-miR-28-5p	RAB3B	5865
hsa-miR-28-5p	RABL6	55684
hsa-miR-28-5p	RACK1	10399
hsa-miR-28-5p	RANBP2	5903
hsa-miR-28-5p	RAP1B	5908
hsa-miR-28-5p	RAP2C	57826
hsa-miR-28-5p	RBM28	55131

hsa-miR-28-5p	RBPJ	3516
hsa-miR-28-5p	RC3H1	149041
hsa-miR-28-5p	RC3H2	54542
hsa-miR-28-5p	RCN2	5955
hsa-miR-28-5p	RCOR1	23186
hsa-miR-28-5p	RECQL5	9400
hsa-miR-28-5p	REG1B	5968
hsa-miR-28-5p	REST	5978
hsa-miR-28-5p	RETREG2	79137
hsa-miR-28-5p	RETREG3	162427
hsa-miR-28-5p	RGS20	8601
hsa-miR-28-5p	RGS4	5999
hsa-miR-28-5p	RHOF	54509
hsa-miR-28-5p	RIC8A	60626
hsa-miR-28-5p	RIPK1	8737
hsa-miR-28-5p	RNF11	26994
hsa-miR-28-5p	RNF141	50862
hsa-miR-28-5p	RNF144B	255488
hsa-miR-28-5p	RNF165	494470
hsa-miR-28-5p	RNPEPL1	57140
hsa-miR-28-5p	RPL22	6146
hsa-miR-28-5p	RPL27A	6157
hsa-miR-28-5p	RPL9	6133
hsa-miR-28-5p	RPRD1A	55197
hsa-miR-28-5p	RPS20	6224
hsa-miR-28-5p	RWDD1	51389
hsa-miR-28-5p	SCAMP3	10067
hsa-miR-28-5p	SCD	6319
hsa-miR-28-5p	SEC24A	10802
hsa-miR-28-5p	SEC24C	9632
hsa-miR-28-5p	SEMA4C	54910
hsa-miR-28-5p	SENP2	59343
hsa-miR-28-5p	SERINC3	10955
hsa-miR-28-5p	SESN1	27244
hsa-miR-28-5p	SESN3	143686
hsa-miR-28-5p	SETBP1	26040
hsa-miR-28-5p	SETD5	55209
hsa-miR-28-5p	SETD7	80854
hsa-miR-28-5p	SF3A2	8175
hsa-miR-28-5p	SFT2D2	375035

hsa-miR-28-5p	SH3PXD2A	9644
hsa-miR-28-5p	SHFL	55337
hsa-miR-28-5p	SHOC2	8036
hsa-miR-28-5p	SHTN1	57698
hsa-miR-28-5p	SIGMAR1	10280
hsa-miR-28-5p	SIK1	150094
hsa-miR-28-5p	SIX5	147912
hsa-miR-28-5p	SKA1	220134
hsa-miR-28-5p	SLC16A10	117247
hsa-miR-28-5p	SLC16A3	9123
hsa-miR-28-5p	SLC1A2	6506
hsa-miR-28-5p	SLC1A5	6510
hsa-miR-28-5p	SLC2A13	114134
hsa-miR-28-5p	SLC35A5	55032
hsa-miR-28-5p	SLC39A14	23516
hsa-miR-28-5p	SLC44A1	23446
hsa-miR-28-5p	SLC6A1	6529
hsa-miR-28-5p	SLC7A5	8140
hsa-miR-28-5p	SLC7A6	9057
hsa-miR-28-5p	SLF1	84250
hsa-miR-28-5p	SLIT2	9353
hsa-miR-28-5p	SMAD1	4086
hsa-miR-28-5p	SMAD4	4089
hsa-miR-28-5p	SMAP1	60682
hsa-miR-28-5p	SMARCC2	6601
hsa-miR-28-5p	SMCP	4184
hsa-miR-28-5p	SMIM12	113444
hsa-miR-28-5p	SMYD1	150572
hsa-miR-28-5p	SNX1	6642
hsa-miR-28-5p	SNX16	64089
hsa-miR-28-5p	SNX2	6643
hsa-miR-28-5p	SNX29	92017
hsa-miR-28-5p	SOAT1	6646
hsa-miR-28-5p	SOGA1	140710
hsa-miR-28-5p	SON	6651
hsa-miR-28-5p	SORT1	6272
hsa-miR-28-5p	SPCS3	60559
hsa-miR-28-5p	SPEN	23013
hsa-miR-28-5p	SPRY4	81848
hsa-miR-28-5p	SPTBN1	6711

hsa-miR-28-5p	SRD5A1	6715
hsa-miR-28-5p	SREBF2	6721
hsa-miR-28-5p	SRPK2	6733
hsa-miR-28-5p	SRPRA	6734
hsa-miR-28-5p	SRRM2	23524
hsa-miR-28-5p	SSRP1	6749
hsa-miR-28-5p	ST14	6768
hsa-miR-28-5p	ST7	7982
hsa-miR-28-5p	STAT5B	6777
hsa-miR-28-5p	STAU1	6780
hsa-miR-28-5p	STK3	6788
hsa-miR-28-5p	STOX2	56977
hsa-miR-28-5p	STRN4	29888
hsa-miR-28-5p	STX12	23673
hsa-miR-28-5p	SUMO3	6612
hsa-miR-28-5p	SYBU	55638
hsa-miR-28-5p	SYMPK	8189
hsa-miR-28-5p	SYNJ2	8871
hsa-miR-28-5p	TAOK1	57551
hsa-miR-28-5p	TAOK2	9344
hsa-miR-28-5p	TAP2	6891
hsa-miR-28-5p	TASOR2	54906
hsa-miR-28-5p	TATDN2	9797
hsa-miR-28-5p	TAX1BP1	8887
hsa-miR-28-5p	TBC1D20	128637
hsa-miR-28-5p	TBC1D9	23158
hsa-miR-28-5p	TBRG4	9238
hsa-miR-28-5p	TCEAL3	85012
hsa-miR-28-5p	TCF4	6925
hsa-miR-28-5p	TEAD1	7003
hsa-miR-28-5p	TEAD3	7005
hsa-miR-28-5p	TENT4A	11044
hsa-miR-28-5p	TENT5A	55603
hsa-miR-28-5p	TERF2IP	54386
hsa-miR-28-5p	TEX261	113419
hsa-miR-28-5p	TGOLN2	10618
hsa-miR-28-5p	THRA	7067
hsa-miR-28-5p	TLK1	9874
hsa-miR-28-5p	TMCO6	55374
hsa-miR-28-5p	TMED4	222068

hsa-miR-28-5p	TMED9	54732
hsa-miR-28-5p	TMEM104	54868
hsa-miR-28-5p	TMEM107	84314
hsa-miR-28-5p	TMEM109	79073
hsa-miR-28-5p	TMEM127	55654
hsa-miR-28-5p	TMEM140	55281
hsa-miR-28-5p	TMEM167A	153339
hsa-miR-28-5p	TMEM167B	56900
hsa-miR-28-5p	TMEM214	54867
hsa-miR-28-5p	TMEM258	746
hsa-miR-28-5p	TMEM41A	90407
hsa-miR-28-5p	TMEM63A	9725
hsa-miR-28-5p	TMPO	7112
hsa-miR-28-5p	TMX3	54495
hsa-miR-28-5p	TMX4	56255
hsa-miR-28-5p	TNFRSF10B	8795
hsa-miR-28-5p	TNIP2	79155
hsa-miR-28-5p	TNKS	8658
hsa-miR-28-5p	TNPO1	3842
hsa-miR-28-5p	TNPO2	30000
hsa-miR-28-5p	TNS1	7145
hsa-miR-28-5p	TOLLIP	54472
hsa-miR-28-5p	TOR1AIP2	163590
hsa-miR-28-5p	TP53	7157
hsa-miR-28-5p	TP53BP2	7159
hsa-miR-28-5p	TPM4	7171
hsa-miR-28-5p	TRAM2	9697
hsa-miR-28-5p	TRIM23	373
hsa-miR-28-5p	TRIM44	54765
hsa-miR-28-5p	TRIM65	201292
hsa-miR-28-5p	TRIO	7204
hsa-miR-28-5p	TRIOBP	11078
hsa-miR-28-5p	TRPS1	7227
hsa-miR-28-5p	TSC22D1	8848
hsa-miR-28-5p	TSPYL4	23270
hsa-miR-28-5p	TTLL4	9654
hsa-miR-28-5p	TUB	7275
hsa-miR-28-5p	TUBA1B	10376
hsa-miR-28-5p	TUBB	203068
hsa-miR-28-5p	TUBB2A	7280

hsa-miR-28-5p	TUBB4A	10382
hsa-miR-28-5p	TUFM	7284
hsa-miR-28-5p	TULP4	56995
hsa-miR-28-5p	TVP23C	201158
hsa-miR-28-5p	TXLNA	200081
hsa-miR-28-5p	UBA6	55236
hsa-miR-28-5p	UBALD1	124402
hsa-miR-28-5p	UBE2H	7328
hsa-miR-28-5p	UBE2Q2	92912
hsa-miR-28-5p	UBE2Z	65264
hsa-miR-28-5p	UBFD1	56061
hsa-miR-28-5p	UBR4	23352
hsa-miR-28-5p	UMPS	7372
hsa-miR-28-5p	UNC13A	23025
hsa-miR-28-5p	UNG	7374
hsa-miR-28-5p	USP1	7398
hsa-miR-28-5p	USP22	23326
hsa-miR-28-5p	USP9X	8239
hsa-miR-28-5p	VAT1L	57687
hsa-miR-28-5p	VCAN	1462
hsa-miR-28-5p	VEZF1	7716
hsa-miR-28-5p	VKORC1	79001
hsa-miR-28-5p	VPS37B	79720
hsa-miR-28-5p	VPS8	23355
hsa-miR-28-5p	VTI1A	143187
hsa-miR-28-5p	VWF	7450
hsa-miR-28-5p	WAC	51322
hsa-miR-28-5p	WDR6	11180
hsa-miR-28-5p	WEE1	7465
hsa-miR-28-5p	WNK1	65125
hsa-miR-28-5p	WSB2	55884
hsa-miR-28-5p	WTAP	9589
hsa-miR-28-5p	XXYLT1	152002
hsa-miR-28-5p	YIPF6	286451
hsa-miR-28-5p	YWHAE	7531
hsa-miR-28-5p	YWHAH	7533
hsa-miR-28-5p	ZBTB18	10472
hsa-miR-28-5p	ZBTB37	84614
hsa-miR-28-5p	ZBTB44	29068
hsa-miR-28-5p	ZBTB47	92999

hsa-miR-28-5p	ZBTB7A	51341
hsa-miR-28-5p	ZBTB7B	51043
hsa-miR-28-5p	ZC3H11A	9877
hsa-miR-28-5p	ZCCHC3	85364
hsa-miR-28-5p	ZDHHC18	84243
hsa-miR-28-5p	ZFP82	284406
hsa-miR-28-5p	ZFP91	80829
hsa-miR-28-5p	ZMAT3	64393
hsa-miR-28-5p	ZMYM6	9204
hsa-miR-28-5p	ZNF106	64397
hsa-miR-28-5p	ZNF14	7561
hsa-miR-28-5p	ZNF175	7728
hsa-miR-28-5p	ZNF207	7756
hsa-miR-28-5p	ZNF281	23528
hsa-miR-28-5p	ZNF444	55311
hsa-miR-28-5p	ZNF451	26036
hsa-miR-28-5p	ZNF496	84838
hsa-miR-28-5p	ZNF629	23361
hsa-miR-28-5p	ZNF655	79027
hsa-miR-28-5p	ZNF768	79724
hsa-miR-28-5p	ZSWIM1	90204
hsa-miR-28-5p	ZSWIM8	23053
hsa-miR-339-3p	H2BC21	8349
hsa-miR-339-3p	H4C2	8366
hsa-miR-339-3p	HDAC1	3065
hsa-miR-339-3p	TNRC6A	27327
hsa-miR-339-3p	ACTN1	87
hsa-miR-339-3p	ADA	100
hsa-miR-339-3p	ADORA2A	135
hsa-miR-339-3p	ADRB1	153
hsa-miR-339-3p	AKIRIN1	79647
hsa-miR-339-3p	ALG3	10195
hsa-miR-339-3p	ANKRD40	91369
hsa-miR-339-3p	ARHGAP17	55114
hsa-miR-339-3p	ARID2	196528
hsa-miR-339-3p	ARPC3	10094
hsa-miR-339-3p	ATP5F1B	506
hsa-miR-339-3p	BDNF	627
hsa-miR-339-3p	BICRA	29998
hsa-miR-339-3p	BRD4	23476
hsa-miR-339-3p	BTG2	7832
hsa-miR-339-3p	C1orf21	81563

hsa-miR-339-3p	CBX2	84733
hsa-miR-339-3p	CCKAR	886
hsa-miR-339-3p	CCKBR	887
hsa-miR-339-3p	CCN1	3491
hsa-miR-339-3p	CDH23	64072
hsa-miR-339-3p	CELF1	10658
hsa-miR-339-3p	CELSR2	1952
hsa-miR-339-3p	CHSY1	22856
hsa-miR-339-3p	CKAP4	10970
hsa-miR-339-3p	CLN6	54982
hsa-miR-339-3p	CLPTM1	1209
hsa-miR-339-3p	CMAS	55907
hsa-miR-339-3p	COL1A1	1277
hsa-miR-339-3p	COL3A1	1281
hsa-miR-339-3p	COL4A2	1284
hsa-miR-339-3p	CRHR2	1395
hsa-miR-339-3p	CSNK1A1	1452
hsa-miR-339-3p	CTNND1	1500
hsa-miR-339-3p	CXCR4	7852
hsa-miR-339-3p	DDB1	1642
hsa-miR-339-3p	DDX3X	1654
hsa-miR-339-3p	DDX6	1656
hsa-miR-339-3p	DHRS13	147015
hsa-miR-339-3p	DICER1	23405
hsa-miR-339-3p	DUSP14	11072
hsa-miR-339-3p	EEF1D	1936
hsa-miR-339-3p	EGR1	1958
hsa-miR-339-3p	EHD2	30846
hsa-miR-339-3p	EIF5A2	56648
hsa-miR-339-3p	EZR	7430
hsa-miR-339-3p	FBN2	2201
hsa-miR-339-3p	FJX1	24147
hsa-miR-339-3p	FLG	2312
hsa-miR-339-3p	FOXO1	2308
hsa-miR-339-3p	FTSJ1	24140
hsa-miR-339-3p	FUT11	170384
hsa-miR-339-3p	FZD2	2535
hsa-miR-339-3p	GABRA6	2559
hsa-miR-339-3p	GARS1	2617
hsa-miR-339-3p	GNB1L	54584
hsa-miR-339-3p	H2AC11	8969
hsa-miR-339-3p	HECTD4	283450
hsa-miR-339-3p	HERC2	8924

hsa-miR-339-3p	HEYL	26508
hsa-miR-339-3p	HNRNPA2B1	3181
hsa-miR-339-3p	HNRNPU	3192
hsa-miR-339-3p	HTR2C	3358
hsa-miR-339-3p	ID3	3399
hsa-miR-339-3p	IGF2	3481
hsa-miR-339-3p	IGSF9B	22997
hsa-miR-339-3p	INSIG1	3638
hsa-miR-339-3p	IPO9	55705
hsa-miR-339-3p	KDM3B	51780
hsa-miR-339-3p	KLHL15	80311
hsa-miR-339-3p	KMT2C	58508
hsa-miR-339-3p	KMT2D	8085
hsa-miR-339-3p	KMT5A	387893
hsa-miR-339-3p	LIG1	3978
hsa-miR-339-3p	LOXL2	4017
hsa-miR-339-3p	LRRFIP1	9208
hsa-miR-339-3p	MANBA	4126
hsa-miR-339-3p	MAOA	4128
hsa-miR-339-3p	MBD3	53615
hsa-miR-339-3p	MCL1	4170
hsa-miR-339-3p	MEX3D	399664
hsa-miR-339-3p	MOCOS	55034
hsa-miR-339-3p	MTA2	9219
hsa-miR-339-3p	NA	NA
hsa-miR-339-3p	NBAS	51594
hsa-miR-339-3p	NEDD8	4738
hsa-miR-339-3p	NFKB1	4790
hsa-miR-339-3p	NME4	4833
hsa-miR-339-3p	NR3C1	2908
hsa-miR-339-3p	NTRK3	4916
hsa-miR-339-3p	NUFIP2	57532
hsa-miR-339-3p	OAZ1	4946
hsa-miR-339-3p	PABPC1	26986
hsa-miR-339-3p	PAPPA2	60676
hsa-miR-339-3p	PATZ1	23598
hsa-miR-339-3p	PAWR	5074
hsa-miR-339-3p	PELI3	246330
hsa-miR-339-3p	PHLDA2	7262
hsa-miR-339-3p	PITPNM3	83394
hsa-miR-339-3p	PLOD1	5351
hsa-miR-339-3p	POMC	5443
hsa-miR-339-3p	POU4F1	5457

hsa-miR-339-3p	PPFIBP2	8495
hsa-miR-339-3p	PRPSAP1	5635
hsa-miR-339-3p	PTPN1	5770
hsa-miR-339-3p	PUS7	54517
hsa-miR-339-3p	RABL6	55684
hsa-miR-339-3p	RANGAP1	5905
hsa-miR-339-3p	RBM23	55147
hsa-miR-339-3p	RGS2	5997
hsa-miR-339-3p	RLIM	51132
hsa-miR-339-3p	RNF138	51444
hsa-miR-339-3p	RNF169	254225
hsa-miR-339-3p	RPL10A	4736
hsa-miR-339-3p	RPS5	6193
hsa-miR-339-3p	RTL10	79680
hsa-miR-339-3p	SAMD1	90378
hsa-miR-339-3p	SAR1A	56681
hsa-miR-339-3p	SCD	6319
hsa-miR-339-3p	SEC16A	9919
hsa-miR-339-3p	SEMA7A	8482
hsa-miR-339-3p	SHOX2	6474
hsa-miR-339-3p	SLC16A1	6566
hsa-miR-339-3p	SLC20A1	6574
hsa-miR-339-3p	SLC39A14	23516
hsa-miR-339-3p	SLC6A2	6530
hsa-miR-339-3p	SLC9A1	6548
hsa-miR-339-3p	SLFN5	162394
hsa-miR-339-3p	SP2	6668
hsa-miR-339-3p	SRM	6723
hsa-miR-339-3p	STIP1	10963
hsa-miR-339-3p	SYNCRIP	10492
hsa-miR-339-3p	SYNE2	23224
hsa-miR-339-3p	SYT9	143425
hsa-miR-339-3p	TAOK2	9344
hsa-miR-339-3p	TBCD	6904
hsa-miR-339-3p	TBCEL	219899
hsa-miR-339-3p	TEAD1	7003
hsa-miR-339-3p	TET2	54790
hsa-miR-339-3p	THOC5	8563
hsa-miR-339-3p	TLN1	7094
hsa-miR-339-3p	TMEM121	80757
hsa-miR-339-3p	TMEM250	90120
hsa-miR-339-3p	TRA2B	6434
hsa-miR-339-3p	TUB	7275

hsa-miR-339-3p	TUBB	203068
hsa-miR-339-3p	TUBB6	84617
hsa-miR-339-3p	TXNRD1	7296
hsa-miR-339-3p	UBE2G1	7326
hsa-miR-339-3p	UNC45B	146862
hsa-miR-339-3p	UQCC2	84300
hsa-miR-339-3p	USP25	29761
hsa-miR-339-3p	UVSSA	57654
hsa-miR-339-3p	VCL	7414
hsa-miR-339-3p	WDR6	11180
hsa-miR-339-3p	WDTC1	23038
hsa-miR-339-3p	XAB2	56949
hsa-miR-339-3p	YAP1	10413
hsa-miR-339-3p	YWHAE	7531
hsa-miR-339-3p	ZBTB10	65986
hsa-miR-339-3p	ZCCHC3	85364
hsa-miR-339-3p	ZFP36	7538
hsa-miR-339-3p	ZFP91	80829
hsa-miR-339-3p	ZNF317	57693
hsa-miR-339-3p	ZNF718	255403
hsa-miR-432-5p	AKT3	10000
hsa-miR-432-5p	CALM2	805
hsa-miR-432-5p	CREB1	1385
hsa-miR-432-5p	ERBB4	2066
hsa-miR-432-5p	GNG2	54331
hsa-miR-432-5p	H2BC4	8347
hsa-miR-432-5p	H2BC5	3017
hsa-miR-432-5p	IGF1R	3480
hsa-miR-432-5p	MMP2	4313
hsa-miR-432-5p	NCOA1	8648
hsa-miR-432-5p	NRIP1	8204
hsa-miR-432-5p	POLR2A	5430
hsa-miR-432-5p	PPP5C	5536
hsa-miR-432-5p	SHC1	6464
hsa-miR-432-5p	ZNF217	7764
hsa-miR-432-5p	ABCC5	10057
hsa-miR-432-5p	ACTN4	81
hsa-miR-432-5p	ADAMTS17	170691
hsa-miR-432-5p	ADAR	103
hsa-miR-432-5p	ADGRL2	23266
hsa-miR-432-5p	AHNAK	79026
hsa-miR-432-5p	AKAP12	9590
hsa-miR-432-5p	AKAP2	11217

hsa-miR-432-5p	AL096711.2	NA
hsa-miR-432-5p	ANKRD11	29123
hsa-miR-432-5p	ANKRD13C	81573
hsa-miR-432-5p	ANKRD17	26057
hsa-miR-432-5p	ANLN	54443
hsa-miR-432-5p	ANO5	203859
hsa-miR-432-5p	AP1S1	1174
hsa-miR-432-5p	AP2A2	161
hsa-miR-432-5p	AP2B1	163
hsa-miR-432-5p	APC	324
hsa-miR-432-5p	APLP1	333
hsa-miR-432-5p	ARHGAP32	9743
hsa-miR-432-5p	ARHGEF4	50649
hsa-miR-432-5p	ARID1A	8289
hsa-miR-432-5p	ARID1B	57492
hsa-miR-432-5p	ARID4B	51742
hsa-miR-432-5p	ARID5B	84159
hsa-miR-432-5p	ASAP1	50807
hsa-miR-432-5p	ASH1L	55870
hsa-miR-432-5p	ATL1	51062
hsa-miR-432-5p	ATM	472
hsa-miR-432-5p	ATP2A2	488
hsa-miR-432-5p	AXL	558
hsa-miR-432-5p	AZIN1	51582
hsa-miR-432-5p	BBS4	585
hsa-miR-432-5p	BBX	56987
hsa-miR-432-5p	BDKRB2	624
hsa-miR-432-5p	BDP1	55814
hsa-miR-432-5p	BHLHE40	8553
hsa-miR-432-5p	BLCAP	10904
hsa-miR-432-5p	BTG1	694
hsa-miR-432-5p	BTG2	7832
hsa-miR-432-5p	C10orf105	414152
hsa-miR-432-5p	C16orf72	29035
hsa-miR-432-5p	C1orf198	84886
hsa-miR-432-5p	CACNA2D1	781
hsa-miR-432-5p	CASKIN1	57524
hsa-miR-432-5p	CAVIN1	284119
hsa-miR-432-5p	CAVIN2	8436
hsa-miR-432-5p	CBX3	11335
hsa-miR-432-5p	CCDC47	57003
hsa-miR-432-5p	CCDC88A	55704
hsa-miR-432-5p	CCN1	3491

hsa-miR-432-5p	CCNI	10983
hsa-miR-432-5p	CCP110	9738
hsa-miR-432-5p	CCSER2	54462
hsa-miR-432-5p	CDC14B	8555
hsa-miR-432-5p	CDC73	79577
hsa-miR-432-5p	CDCA7L	55536
hsa-miR-432-5p	CDH10	1008
hsa-miR-432-5p	CDH13	1012
hsa-miR-432-5p	CDH2	1000
hsa-miR-432-5p	CDK5R1	8851
hsa-miR-432-5p	CDR2	1039
hsa-miR-432-5p	CEMIP	57214
hsa-miR-432-5p	CENPA	1058
hsa-miR-432-5p	CENPC	1060
hsa-miR-432-5p	CETP	1071
hsa-miR-432-5p	CHAC1	79094
hsa-miR-432-5p	CHGB	1114
hsa-miR-432-5p	CHMP1B	57132
hsa-miR-432-5p	CHMP3	51652
hsa-miR-432-5p	CLIP2	7461
hsa-miR-432-5p	CLUH	23277
hsa-miR-432-5p	CNOT2	4848
hsa-miR-432-5p	CNST	163882
hsa-miR-432-5p	CNTNAP1	8506
hsa-miR-432-5p	COL12A1	1303
hsa-miR-432-5p	COL1A2	1278
hsa-miR-432-5p	COL5A1	1289
hsa-miR-432-5p	COL6A1	1291
hsa-miR-432-5p	COL6A3	1293
hsa-miR-432-5p	CPD	1362
hsa-miR-432-5p	CPEB2	132864
hsa-miR-432-5p	CPEB4	80315
hsa-miR-432-5p	CPSF3	51692
hsa-miR-432-5p	CPT1A	1374
hsa-miR-432-5p	CREBZF	58487
hsa-miR-432-5p	CSDE1	7812
hsa-miR-432-5p	CSNK1G2	1455
hsa-miR-432-5p	CTDSP2	10106
hsa-miR-432-5p	CTNNA1	1495
hsa-miR-432-5p	DCTN2	10540
hsa-miR-432-5p	DENND6A	201627
hsa-miR-432-5p	DHX16	8449
hsa-miR-432-5p	DIDO1	11083

hsa-miR-432-5p	DIPK1A	388650
hsa-miR-432-5p	DIPK2A	205428
hsa-miR-432-5p	DLC1	10395
hsa-miR-432-5p	DLG5	9231
hsa-miR-432-5p	DNAJB4	11080
hsa-miR-432-5p	DNAJB6	10049
hsa-miR-432-5p	DNAJC6	9829
hsa-miR-432-5p	DNER	92737
hsa-miR-432-5p	DNM1L	10059
hsa-miR-432-5p	DNTTIP2	30836
hsa-miR-432-5p	DPM2	8818
hsa-miR-432-5p	DR1	1810
hsa-miR-432-5p	DSEL	92126
hsa-miR-432-5p	DST	667
hsa-miR-432-5p	DSTYK	25778
hsa-miR-432-5p	DUSP16	80824
hsa-miR-432-5p	DYNC1H1	1778
hsa-miR-432-5p	ECHDC1	55862
hsa-miR-432-5p	EDEM3	80267
hsa-miR-432-5p	EDIL3	10085
hsa-miR-432-5p	EFHD2	79180
hsa-miR-432-5p	EIF4A1	1973
hsa-miR-432-5p	EIF4G3	8672
hsa-miR-432-5p	ENAH	55740
hsa-miR-432-5p	ENC1	8507
hsa-miR-432-5p	EPAS1	2034
hsa-miR-432-5p	ERO1B	56605
hsa-miR-432-5p	ESPL1	9700
hsa-miR-432-5p	ETNK1	55500
hsa-miR-432-5p	EWSR1	2130
hsa-miR-432-5p	EXOSC10	5394
hsa-miR-432-5p	FAM160B1	57700
hsa-miR-432-5p	FAM171A1	221061
hsa-miR-432-5p	FAM171B	165215
hsa-miR-432-5p	FAR1	84188
hsa-miR-432-5p	FBN1	2200
hsa-miR-432-5p	FBXL3	26224
hsa-miR-432-5p	FLNA	2316
hsa-miR-432-5p	FMN1	342184
hsa-miR-432-5p	FN1	2335
hsa-miR-432-5p	FNDC3A	22862
hsa-miR-432-5p	FNDC3B	64778
hsa-miR-432-5p	FRS2	10818

hsa-miR-432-5p	FSCN1	6624
hsa-miR-432-5p	FSTL1	11167
hsa-miR-432-5p	FUT8	2530
hsa-miR-432-5p	FZR1	51343
hsa-miR-432-5p	G3BP1	10146
hsa-miR-432-5p	GABARAPL2	11345
hsa-miR-432-5p	GABRB2	2561
hsa-miR-432-5p	GABRB3	2562
hsa-miR-432-5p	GALNT2	2590
hsa-miR-432-5p	GAPVD1	26130
hsa-miR-432-5p	GAS1	2619
hsa-miR-432-5p	GDA	9615
hsa-miR-432-5p	GDF5OS	554250
hsa-miR-432-5p	GFPT1	2673
hsa-miR-432-5p	GGT5	2687
hsa-miR-432-5p	GJD2	57369
hsa-miR-432-5p	GMFB	2764
hsa-miR-432-5p	GNA12	2768
hsa-miR-432-5p	GNAS	2778
hsa-miR-432-5p	GOLGA7	51125
hsa-miR-432-5p	GOLIM4	27333
hsa-miR-432-5p	GRIA2	2891
hsa-miR-432-5p	GRIN2A	2903
hsa-miR-432-5p	GRIPAP1	56850
hsa-miR-432-5p	GSKIP	51527
hsa-miR-432-5p	GUCY1B1	2983
hsa-miR-432-5p	H1-0	3005
hsa-miR-432-5p	HEATR5A	25938
hsa-miR-432-5p	HIPK2	28996
hsa-miR-432-5p	HMGB2	3148
hsa-miR-432-5p	HOOK3	84376
hsa-miR-432-5p	HSD17B4	3295
hsa-miR-432-5p	HSP90B1	7184
hsa-miR-432-5p	HSPA8	3312
hsa-miR-432-5p	HUWE1	10075
hsa-miR-432-5p	HYOU1	10525
hsa-miR-432-5p	IAPP	3375
hsa-miR-432-5p	ID2	3398
hsa-miR-432-5p	ID4	3400
hsa-miR-432-5p	IDH3A	3419
hsa-miR-432-5p	IGSF8	93185
hsa-miR-432-5p	IL6ST	3572
hsa-miR-432-5p	IMPDH2	3615

hsa-miR-432-5p	ING5	84289
hsa-miR-432-5p	INS	3630
hsa-miR-432-5p	IPO7	10527
hsa-miR-432-5p	IRF2BP2	359948
hsa-miR-432-5p	ITGA11	22801
hsa-miR-432-5p	ITGB8	3696
hsa-miR-432-5p	ITM2C	81618
hsa-miR-432-5p	ITPRIPL1	150771
hsa-miR-432-5p	ITSN1	6453
hsa-miR-432-5p	JAK1	3716
hsa-miR-432-5p	KARS1	3735
hsa-miR-432-5p	KCNB1	3745
hsa-miR-432-5p	KCNC2	3747
hsa-miR-432-5p	KCNK16	83795
hsa-miR-432-5p	KCNMA1	3778
hsa-miR-432-5p	KCNMB1	3779
hsa-miR-432-5p	KCTD10	83892
hsa-miR-432-5p	KCTD3	51133
hsa-miR-432-5p	KDM2B	84678
hsa-miR-432-5p	KDM4C	23081
hsa-miR-432-5p	KIAA0319L	79932
hsa-miR-432-5p	KIAA1671	85379
hsa-miR-432-5p	KIF21A	55605
hsa-miR-432-5p	KIF3B	9371
hsa-miR-432-5p	KLHL28	54813
hsa-miR-432-5p	KTN1	3895
hsa-miR-432-5p	LANCL1	10314
hsa-miR-432-5p	LARP1	23367
hsa-miR-432-5p	LASP1	3927
hsa-miR-432-5p	LGI3	203190
hsa-miR-432-5p	LIF	3976
hsa-miR-432-5p	LOX	4015
hsa-miR-432-5p	LRBA	987
hsa-miR-432-5p	LRP11	84918
hsa-miR-432-5p	LRPPRC	10128
hsa-miR-432-5p	LRRC1	55227
hsa-miR-432-5p	LSAMP	4045
hsa-miR-432-5p	LTBP1	4052
hsa-miR-432-5p	LYRM7	90624
hsa-miR-432-5p	M6PR	4074
hsa-miR-432-5p	MAN1A2	10905
hsa-miR-432-5p	MAP1A	4130
hsa-miR-432-5p	MAP1B	4131

hsa-miR-432-5p	MAP2	4133
hsa-miR-432-5p	MAP4	4134
hsa-miR-432-5p	MAPK8IP3	23162
hsa-miR-432-5p	MAPRE1	22919
hsa-miR-432-5p	MARCHF6	10299
hsa-miR-432-5p	MARCKS	4082
hsa-miR-432-5p	MATN3	4148
hsa-miR-432-5p	MBNL2	10150
hsa-miR-432-5p	MDH1	4190
hsa-miR-432-5p	MDM2	4193
hsa-miR-432-5p	MECP2	4204
hsa-miR-432-5p	MEDAG	84935
hsa-miR-432-5p	MEGF9	1955
hsa-miR-432-5p	MGAT1	4245
hsa-miR-432-5p	MICU1	10367
hsa-miR-432-5p	MICU3	286097
hsa-miR-432-5p	MLLT6	4302
hsa-miR-432-5p	MME	4311
hsa-miR-432-5p	MOGAT1	116255
hsa-miR-432-5p	MRC2	9902
hsa-miR-432-5p	MROH7	374977
hsa-miR-432-5p	MRPL34	64981
hsa-miR-432-5p	MSN	4478
hsa-miR-432-5p	MTMR10	54893
hsa-miR-432-5p	MTPN	136319
hsa-miR-432-5p	MTRNR2L10	100463488
hsa-miR-432-5p	MTRNR2L12	100462981
hsa-miR-432-5p	MYH9	4627
hsa-miR-432-5p	MYO1B	4430
hsa-miR-432-5p	NA	NA
hsa-miR-432-5p	NAP1L5	266812
hsa-miR-432-5p	NAPB	63908
hsa-miR-432-5p	NBAS	51594
hsa-miR-432-5p	NCAPD3	23310
hsa-miR-432-5p	NECAB1	64168
hsa-miR-432-5p	NES	10763
hsa-miR-432-5p	NET1	10276
hsa-miR-432-5p	NF1	4763
hsa-miR-432-5p	NFE2L1	4779
hsa-miR-432-5p	NFIC	4782
hsa-miR-432-5p	NMRAL1	57407
hsa-miR-432-5p	NNMT	4837
hsa-miR-432-5p	NPTX2	4885

hsa-miR-432-5p	NR1D1	9572
hsa-miR-432-5p	NR2F2	7026
hsa-miR-432-5p	NRG3	10718
hsa-miR-432-5p	NUCB2	4925
hsa-miR-432-5p	NUP62	23636
hsa-miR-432-5p	OGT	8473
hsa-miR-432-5p	OLIG1	116448
hsa-miR-432-5p	OLIG2	10215
hsa-miR-432-5p	OPCML	4978
hsa-miR-432-5p	OR2C3	81472
hsa-miR-432-5p	OR6A2	8590
hsa-miR-432-5p	OSBPL10	114884
hsa-miR-432-5p	OTUD4	54726
hsa-miR-432-5p	P3H1	64175
hsa-miR-432-5p	P4HB	5034
hsa-miR-432-5p	PALLD	23022
hsa-miR-432-5p	PALM2AKAP2	445815
hsa-miR-432-5p	PBRM1	55193
hsa-miR-432-5p	PBXIP1	57326
hsa-miR-432-5p	PCDH11X	27328
hsa-miR-432-5p	PCDH9	5101
hsa-miR-432-5p	PCLO	27445
hsa-miR-432-5p	PCSK2	5126
hsa-miR-432-5p	PDCD7	10081
hsa-miR-432-5p	PDCL	5082
hsa-miR-432-5p	PDE4DIP	9659
hsa-miR-432-5p	PDIA4	9601
hsa-miR-432-5p	PDS5B	23047
hsa-miR-432-5p	PEG10	23089
hsa-miR-432-5p	PFKFB2	5208
hsa-miR-432-5p	PFKFB3	5209
hsa-miR-432-5p	PFN1	5216
hsa-miR-432-5p	PGK1	5230
hsa-miR-432-5p	PGRMC2	10424
hsa-miR-432-5p	PHF13	148479
hsa-miR-432-5p	PHLDB2	90102
hsa-miR-432-5p	PIGT	51604
hsa-miR-432-5p	PIK3CB	5291
hsa-miR-432-5p	PITPNM1	9600
hsa-miR-432-5p	PLA2G12A	81579
hsa-miR-432-5p	PLAT	5327
hsa-miR-432-5p	PLEC	5339
hsa-miR-432-5p	PNISR	25957

hsa-miR-432-5p	PNMA8A	55228
hsa-miR-432-5p	PNPLA2	57104
hsa-miR-432-5p	PNPLA8	50640
hsa-miR-432-5p	POLA1	5422
hsa-miR-432-5p	PON2	5445
hsa-miR-432-5p	PPDPF	79144
hsa-miR-432-5p	PRKAB1	5564
hsa-miR-432-5p	PRKCH	5583
hsa-miR-432-5p	PRKX	5613
hsa-miR-432-5p	PRR5-ARHGAP8	553158
hsa-miR-432-5p	PRRC2B	84726
hsa-miR-432-5p	PSAP	5660
hsa-miR-432-5p	PSD3	23362
hsa-miR-432-5p	PTBP2	58155
hsa-miR-432-5p	PTBP3	9991
hsa-miR-432-5p	PTHLH	5744
hsa-miR-432-5p	PTPN3	5774
hsa-miR-432-5p	RAB11A	8766
hsa-miR-432-5p	RAB2A	5862
hsa-miR-432-5p	RAB3GAP1	22930
hsa-miR-432-5p	RAD50	10111
hsa-miR-432-5p	RAF1	5894
hsa-miR-432-5p	RAPH1	65059
hsa-miR-432-5p	RASA1	5921
hsa-miR-432-5p	RBFOX1	54715
hsa-miR-432-5p	RBFOX2	23543
hsa-miR-432-5p	RBM26	64062
hsa-miR-432-5p	RCOR1	23186
hsa-miR-432-5p	RESF1	55196
hsa-miR-432-5p	RFK	55312
hsa-miR-432-5p	RGS7BP	401190
hsa-miR-432-5p	RHOB	388
hsa-miR-432-5p	RHOBTB2	23221
hsa-miR-432-5p	RHOBTB3	22836
hsa-miR-432-5p	RNF103-CHMP3	100526767
hsa-miR-432-5p	RNF141	50862
hsa-miR-432-5p	RNF213	57674
hsa-miR-432-5p	RPLP0	6175
hsa-miR-432-5p	RTN3	10313
hsa-miR-432-5p	SACS	26278
hsa-miR-432-5p	SAE1	10055
hsa-miR-432-5p	SATB1	6304
hsa-miR-432-5p	SBDS	51119

hsa-miR-432-5p	SCD	6319
hsa-miR-432-5p	SCG2	7857
hsa-miR-432-5p	SDE2	163859
hsa-miR-432-5p	SEC14L4	284904
hsa-miR-432-5p	SEC24B	10427
hsa-miR-432-5p	SEPTIN2	4735
hsa-miR-432-5p	SERP1	27230
hsa-miR-432-5p	SERPINE2	5270
hsa-miR-432-5p	SERPINH1	871
hsa-miR-432-5p	SESN3	143686
hsa-miR-432-5p	SETBP1	26040
hsa-miR-432-5p	SETX	23064
hsa-miR-432-5p	SH3PXD2B	285590
hsa-miR-432-5p	SIN3B	23309
hsa-miR-432-5p	SIPA1L2	57568
hsa-miR-432-5p	SLBP	7884
hsa-miR-432-5p	SLC1A1	6505
hsa-miR-432-5p	SLC1A2	6506
hsa-miR-432-5p	SLC24A2	25769
hsa-miR-432-5p	SLC2A13	114134
hsa-miR-432-5p	SLC4A4	8671
hsa-miR-432-5p	SLC8A1	6546
hsa-miR-432-5p	SLC9A6	10479
hsa-miR-432-5p	SLITRK5	26050
hsa-miR-432-5p	SMARCA5	8467
hsa-miR-432-5p	SMU1	55234
hsa-miR-432-5p	SNRNP200	23020
hsa-miR-432-5p	SNRPD2	6633
hsa-miR-432-5p	SNU13	4809
hsa-miR-432-5p	SOBP	55084
hsa-miR-432-5p	SOCS5	9655
hsa-miR-432-5p	SOCS7	30837
hsa-miR-432-5p	SOD2	6648
hsa-miR-432-5p	SORCS1	114815
hsa-miR-432-5p	SORT1	6272
hsa-miR-432-5p	SOX9	6662
hsa-miR-432-5p	SP3	6670
hsa-miR-432-5p	SPANXN1	494118
hsa-miR-432-5p	SPANXN5	494197
hsa-miR-432-5p	SPARC	6678
hsa-miR-432-5p	SPCS3	60559
hsa-miR-432-5p	SPTBN1	6711
hsa-miR-432-5p	SRRM2	23524

hsa-miR-432-5p	STAMBP	10617
hsa-miR-432-5p	STC1	6781
hsa-miR-432-5p	STEAP2	261729
hsa-miR-432-5p	STIP1	10963
hsa-miR-432-5p	STK10	6793
hsa-miR-432-5p	STRIP1	85369
hsa-miR-432-5p	SUB1	10923
hsa-miR-432-5p	SUMO3	6612
hsa-miR-432-5p	SURF4	6836
hsa-miR-432-5p	SYN3	8224
hsa-miR-432-5p	SYNCRIP	10492
hsa-miR-432-5p	SYT4	6860
hsa-miR-432-5p	TAF9B	51616
hsa-miR-432-5p	TAGLN	6876
hsa-miR-432-5p	TAOK1	57551
hsa-miR-432-5p	TFPI	7035
hsa-miR-432-5p	TFRC	7037
hsa-miR-432-5p	THAP4	51078
hsa-miR-432-5p	THBS2	7058
hsa-miR-432-5p	TIMP3	7078
hsa-miR-432-5p	TLN1	7094
hsa-miR-432-5p	TMEFF1	8577
hsa-miR-432-5p	TMPRSS2	7113
hsa-miR-432-5p	TMTC1	83857
hsa-miR-432-5p	TMX4	56255
hsa-miR-432-5p	TNFAIP1	7126
hsa-miR-432-5p	TPP2	7174
hsa-miR-432-5p	TRAPPC2	6399
hsa-miR-432-5p	TSC22D1	8848
hsa-miR-432-5p	TSC22D2	9819
hsa-miR-432-5p	TUBB	203068
hsa-miR-432-5p	TUBB2A	7280
hsa-miR-432-5p	UBAP1	51271
hsa-miR-432-5p	UBE3A	7337
hsa-miR-432-5p	UBL3	5412
hsa-miR-432-5p	UBR5	51366
hsa-miR-432-5p	UBTF	7343
hsa-miR-432-5p	UGP2	7360
hsa-miR-432-5p	UGT8	7368
hsa-miR-432-5p	UNC13A	23025
hsa-miR-432-5p	UNC5B	219699
hsa-miR-432-5p	UNC79	57578
hsa-miR-432-5p	USP53	54532

hsa-miR-432-5p	USP9Y	8287
hsa-miR-432-5p	VCL	7414
hsa-miR-432-5p	VGLL3	389136
hsa-miR-432-5p	VPS13D	55187
hsa-miR-432-5p	VPS26A	9559
hsa-miR-432-5p	VPS29	51699
hsa-miR-432-5p	VPS72	6944
hsa-miR-432-5p	WARS2	10352
hsa-miR-432-5p	WDFY3	23001
hsa-miR-432-5p	WSB1	26118
hsa-miR-432-5p	XRCC6	2547
hsa-miR-432-5p	YARS1	8565
hsa-miR-432-5p	YTHDC1	91746
hsa-miR-432-5p	ZBTB20	26137
hsa-miR-432-5p	ZBTB38	253461
hsa-miR-432-5p	ZC3H3	23144
hsa-miR-432-5p	ZC3H6	376940
hsa-miR-432-5p	ZC3H7A	29066
hsa-miR-432-5p	ZCCHC14	23174
hsa-miR-432-5p	ZEB1	6935
hsa-miR-432-5p	ZFC3H1	196441
hsa-miR-432-5p	ZFP91	80829
hsa-miR-432-5p	ZFX	7543
hsa-miR-432-5p	ZFYVE26	23503
hsa-miR-432-5p	ZMAT1	84460
hsa-miR-432-5p	ZMPSTE24	10269
hsa-miR-432-5p	ZNF281	23528
hsa-miR-432-5p	ZNF33A	7581
hsa-miR-432-5p	ZNF518A	9849
hsa-miR-432-5p	ZNF605	100289635
hsa-miR-432-5p	ZNF638	27332
hsa-miR-432-5p	ZNF644	84146
hsa-miR-432-5p	ZNF652	22834
hsa-miR-432-5p	ZNF784	147808
